# Electrochemical Hofmann Rearrangement for Modular Synthesis of Unsymmetrical Ureas and Late‐Stage Modification of Drugs

**DOI:** 10.1002/advs.74920

**Published:** 2026-03-25

**Authors:** Xue‐Qing Mou, Xiao Zha, Yu‐Rui Jian, Shao‐Xing Zhong, Xin‐Yu Fu, Sheng‐Yang Xu, Wei Xiang, Bao‐Dong Cui, Yun Zhang, Yong‐Zheng Chen

**Affiliations:** ^1^ Guizhou Provincial Key Laboratory of Innovation and Manufacturing for Pharmaceuticals School of Pharmacy Zunyi Medical University Zunyi P. R. China; ^2^ School of Pharmacy Key Laboratory of Basic Pharmacology of Ministry of Education and Joint International Research Laboratory of Ethnomedicine of Ministry of Education Zunyi Medical University Zunyi P. R. China

**Keywords:** electrosynthesis, Hofmann rearrangement, N‐acylureas, unsymmetrical ureas

## Abstract

Due to the poor compatibility of amines under oxidative conditions and the low chemoselectivity between two different amides, the Hofmann rearrangement of amides involving amine or other amide nucleophiles poses significant challenges. Herein, we present a versatile electrochemical reaction that achieves highly chemoselective Hofmann rearrangement of two different amides with the addition of only sodium iodide, enabling the efficient synthesis of single *N*‐acylureas. Additionally, under similar electrochemical conditions, both primary and secondary amines can also efficiently react with amides to produce unsymmetrical ureas. This electrosynthetic method exhibits good functional group tolerance and has been successfully employed to synthesize five herbicides, as well as to facilitate the late‐stage functionalization of over twenty amine‐containing drugs. Furthermore, mechanism studies indicated that the use of NaI guaranteed the feasibility and compatibility of the reaction.

## Graphical abstract

An electrochemical Hofmann rearrangement of amide involving amine or other amide nucleophiles has been developed. The use of NaI guarantees the feasibility and compatibility of the electrosynthesis protocol, which enables highly chemoselective synthesis of unsymmetrical N‐acylureas from two different amides, as well as the production of unsymmetrical ureas from amines, including various amine‐containing drugs.

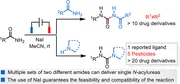

## 1 Introduction

**SCHEME 1:**
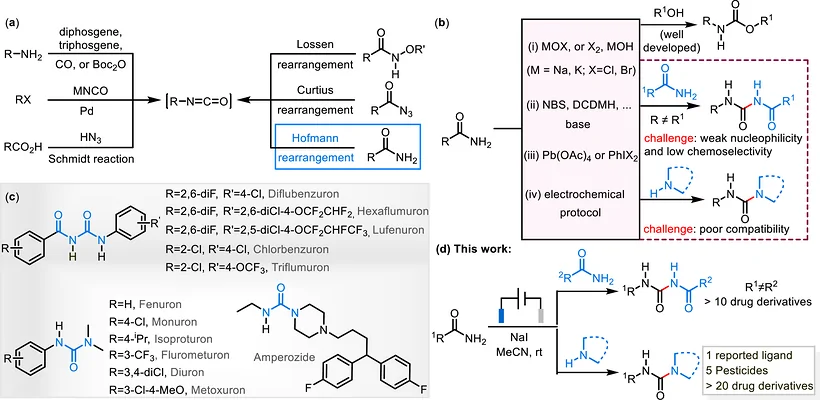
(a) Preparation of isocyanates. (b) Overview of the Hofmann rearrangement. (c) Representative pharmaceuticals and agrochemicals featuring unsymmetrical urea. (d) This work: electrochemical Hofmann rearrangement for modular synthesis of unsymmetrical ureas and late‐stage modification of amine‐containing drugs.

Isocyanates are crucial synthetic intermediates because they can be easily converted into amines [1, 2], ureas [3, 4, 5], amides [6, 7], and carbamates [8, 9], all of which are widely utilized in synthetic and pharmaceutical chemistry. Consequently, the preparation of isocyanates has attracted considerable attention and led to the advancement of various synthetic methods for their production (Scheme 1a). These strategies include the treatment of primary amines with phosgenacid chlorides [10, 11], diphosgene [12, 13], triphosgene [14, 15], di‐tertbutyl dicarbonate (Boc_2_O) [16, 17], carbon monoxide [18, 19], or carbon dioxide [20, 21]; palladium‐catalyzed cross‐coupling of aryl halides with metal cyanates [22]; the Schmidt reaction of carboxylic acids with azides [23]; the Lossen rearrangement involving hydroxamic acids [24, 25]; and the Curtius rearrangement of acyl azides [26, 27]. Despite these developments, many of these methods involve toxic, corrosive, or unstable starting materials. In terms of stability and the easy accessibility of substrates, the Hofmann rearrangement of amides [28, 29] offers distinct advantages for both laboratory and industrial applications.

In the well‐developed Hofmann rearrangement reactions, most procedures predominantly utilize stoichiometric amounts of corrosive reagents (i.e., Br_2_) [30, 31, 32], or other oxidants (i.e., *N*‐haloacylamide, lead tetraacetate, and high‐valent iodine reagents) (Scheme 1b) [33, 34, 35, 36]. These methods typically produce large amounts of waste, complicating purification processes and raising environmental concerns. Additionally, such transformations frequently suffer from poor functional group compatibility, which restricts their applicability in the synthesis of diversely functionalized molecules and the late‐stage modification of drugs.

Organic electrosynthesis employs electrons to drive chemical transformations, thereby eliminating the need for stoichiometric amounts of redox reagents. Importantly, it allows for the regulation of reaction activity and selectivity by manipulating electric current and potential, enabling the achievement of synthetically challenging transformations [37, 38, 39, 40, 41, 42, 43, 44, 45, 46, 47, 48]. Unarguably, electrosynthesis is gradually becoming an important green technology to improve the Hofmann rearrangement and has led to several impressive works under this topic [49, 50, 51, 52, 53, 54]. However, similar to traditional oxidative strategies for these transformations, recent advancements in the electrochemical Hofmann rearrangement primarily focus on the formation of carbamates through the nucleophilic attack of alcohol on in situ‐generated isocyanates. Probably due to the poor compatibility of oxidative‐labile amines and the low chemoselectivity between two different amides, the electrochemical Hofmann reactions of amides with amine or other amide nucleophiles toward the synthesis of *N*‐alkyl unsymmetric ureas and *N*‐acyl ureas pose significant challenges.

*N*‐alkyl‐substituted unsymmetric ureas and *N*‐acylureas are important scaffolds and key pharmacophores in pharmaceuticals [55, 56, 57, 58] and agrochemicals [59, 60]. For example, pesticides such as Diflubenzuron, Hexaflumuron, Lufenuron, Chlorbenzuron, and Triflumuron contain *N*‐acyl urea structures, while herbicides like Fenuron, Monuron, Isoproturon, Flurometuron, Diuron, and Metoxuron, along with medicinal compounds such as Amperozide, Regorafenib, and Cariprazine, feature *N*‐alkyl unsymmetric urea motifs (Scheme 1c). Furthermore, unsymmetric ureas can also serve as versatile organic catalysts [61, 62, 63, 64] or ligands [65, 66, 67, 68, 69]. However, the preparation of these unsymmetric ureas often involves precious metals like palladium, hazardous reagents, cumbersome procedures, or low selectivity [70, 71, 72, 73]. Therefore, it is highly desirable to harness the easily controllable characteristics of organic electrosynthesis to further expand the applications of the Hofmann rearrangement reaction for accessing novel unsymmetric urea molecules.

This study presents a mild electrochemical technique that efficiently achieves the Hofmann rearrangement between two amides by simply adding sodium iodide as both the supporting electrolyte and iodine source. Multiple sets of two different amides can undergo highly selective Hofmann rearrangement reactions to generate single *N*‐acylureas, including more than 10 drug derivatives. Notably, many examples could not be prepared easily by existing routes (e.g., amines reacting with α‐oxoisocyanates) due to difficulties in obtaining the starting materials, underscoring the value of the chemoselective Hofmann rearrangement of two different amides. Under similar conditions, amine nucleophiles are well‐tolerated in this reaction, allowing for the synthesis of a variety of ureas, including five herbicides and a previously reported ligand molecule [66]. Moreover, this method successfully enables the late‐stage functionalization of over twenty complex amine‐containing drug molecules (Scheme 1d). These advantages provide a complementary approach to the impressive protocols recently developed by Lei [74], Chen [75], and Ton [76]. for the synthesis of unsymmetric ureas, which further advance the development of pharmaceuticals and agrochemicals as well as asymmetric catalysis.

## 2 Results and Discussion

We commenced the optimization of the electrochemical Hofmann rearrangement using Levetiracetam and *p*‐Toluamide as the model substrates (Table 1). Through systematic investigation, we identified the optimal reaction conditions: employing a graphite rod as the anode, a platinum plate as the cathode, sodium iodide (2 equiv.) as the supporting electrolyte in acetonitrile with a constant current of 10 mA at room temperature under an air atmosphere for 8 h. Under these conditions, *N*‐acylurea product Pab was obtained in 61% isolated yield, while only trace amounts of *N*‐acylureas Pbb were produced, and *N*‐acylureas Paa and Pba were not detected (entry 1). Further screening of alternative reaction solvents, including hexafluoroisopropanol, methanol, water, and dichloromethane, showed that only the use of dichloromethane could deliver the target product, but in a lower yield (entries 2 and 3). Additionally, we evaluated several commonly used electrolytes, including *^n^* Bu_4_NBF_4_, *^n^* Bu_4_NPF_6_, *^n^* Bu_4_NOAc, and a variety of halide salts (*^n^* Bu_4_NBr, *^n^* Bu_4_NCl, *^n^* Bu_4_NI, NH_4_I, KI, NaCl, and NaBr). It was observed that a dramatic reduction in the yield of the desired compound Pab when using *^n^* Bu_4_NI, KI, or NaBr, while the other tested electrolytes failed to produce the target compound (entries 4–6). These results indicated that sodium iodide, which has been infrequently utilized in the existing literature, played a crucial role in this organic electrosynthesis. Based on these results, we further optimized additional reaction parameters, including electrode materials, reaction current, and sodium iodide loading; however, none of these alternatives outperformed the original conditions (entries 7–14). Controlled experiments revealed that (1) conducting the reaction under an argon atmosphere did not further enhance the yield, whereas exposure to an oxygen environment slightly inhibited product formation; (2) electricity played a key role in the reaction; and (3) compound Pbb was obtained with a yield of 78% in the absence of Levetiracetam, while compound Paa was not detected without *p*‐Toluamide (for detailed screening information, please see Table S2).

**TABLE 1:** Optimization of electrochemical Hofmann rearrangement involving two different amides.^a^ *^.^*

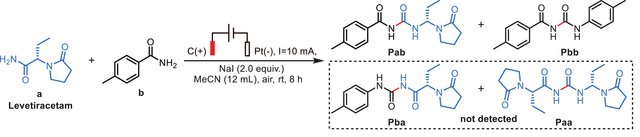			

Entry	Deviation from the standard conditions	Yield of Pab (%)^b^	Yield of Pbb (%)^b^
1	None	61	trace
2	MeCN → MeOH, H_2_O, or HFIP	not detected	not detected
3	MeCN → DCM	37	trace
4	NaI → ^n^Bu_4_NBF_4_, ^n^Bu_4_NPF_6,_^n^Bu_4_NOAc, ^n^Bu_4_NCl, ^n^Bu_4_NBr, NH_4_I, NaCl	not detected	not detected
5	NaI → ^n^Bu_4_NI, NaBr	18‐22	trace
6	NaI → KI	32	15
7	C (+) / Pt (‐) → C (+)/Ni (‐), C (+)/Cu (‐), Pt (‐) / Pt (+), C (‐) / C (+)	28‐48	trace
8	NaI (2.0 → 0.5 equiv.)	25	trace
9	NaI (2.0 → 1.0 equiv.)	46	trace
10	NaI (2.0 → 3.0 equiv.)	54	trace
11	10 mA →8 mA	35	trace
12	10 mA →12 mA	43	trace
13	8 h → 6 h	49	trace
14	8 h → 10 h	54	9
15	Air → Ar	55	trace
16	Air → O_2_	48	12
17	No electricity	not detected	not detected
18	without a, 8 mA, 6 h instead of 10 mA, 8 h	—	78%

^a^ Without further notification, the reactions were performed with Levetiracetam a (0.3 mmol, 1.0 equiv.), p‐Toluamide b (0.45 mmol, 1.5 equiv.), NaI (0.6 mmol, 2.0 equiv.) in MeCN (12 mL) within an undivided cell (a three‐necked flask equipped with a graphite rod and a platinum plate), while maintaining a constant current of 10 mA under air at room temperature for 8 h;

^b^ Isolated yields.

**SCHEME 2:**
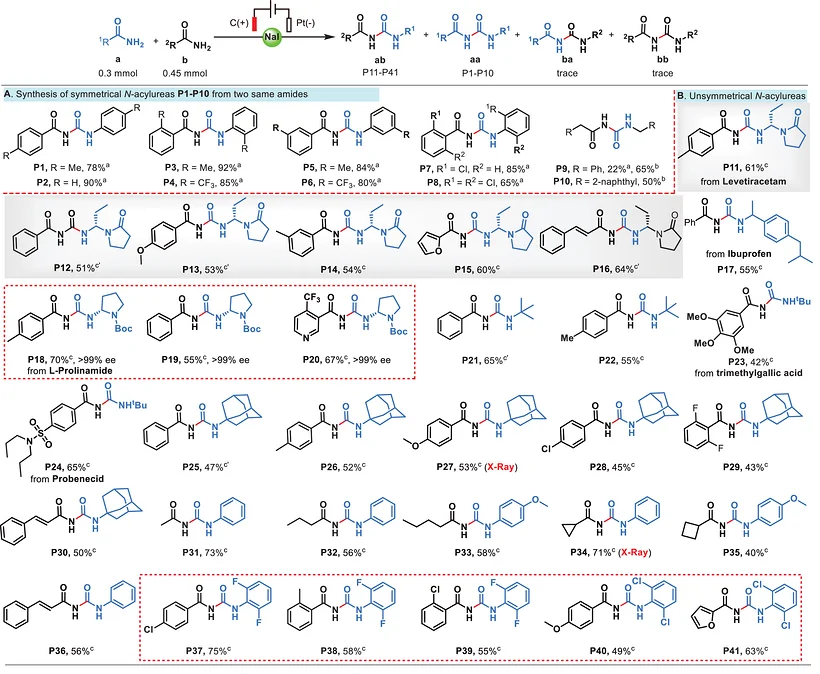
Substrate scope for the synthesis of *N*‐acylureas. ^[a]^Reaction condition: amide (0.3 mmol), NaI (0.6 mmol), C(+)/Pt(‐), 8 mA, MeCN (12 mL), air, rt, 4–6 h, isolated yield; ^[b]^Reaction condition: amide(0.3 mmol), NaBr(0.9 mmol), C(+)/Pt(‐), 12 mA, MeCN:H_2_O (12 mL: mL), air, rt, 8 h, isolated yield;^[c]^Reaction condition: amide a (0.3 mmol), amide b (0.45 mmol), NaI (0.6 mmol), C(+)/Pt(‐), 10 mA, MeCN (12 mL), air, rt, 6–8 h, isolated yield; ^[c’]^ Replace with 2.5 equivalents of amide b.

With the optimized reaction conditions in hand, we first investigate the substrate scope for the synthesis of symmetrical *N*‐acylureas (Scheme 2A). As exemplified by **P1‐P10**, this reaction worked well over structurally diverse aromatic carboxamides. Overall, the reaction systems were relatively clean, with few by‐products observed. The relatively low yields for certain amides could be attributed to incomplete consumption of the starting materials. Notably, the obvious effect of electronic properties and substituent positions on the aromatic ring of the amides was not observed. Various substituents on the aromatic ring of carboxamides, including halogens, alkyl, and trifluoromethyl‌, proved compatible with the reaction, affording the corresponding symmetrical *N*‐acylureas in 65%–92% yields. When the phenyl ring of the aromatic carboxamides was replaced with an alkyl group, the corresponding *N*‐acylureas could be obtained, albeit with low yields. Encouragingly, further investigation revealed that improved reaction conditions (**Condition B**) could significantly enhance the yields of these related products.

Next, we investigated the substrate scope of the electrochemical Hofmann rearrangement involving two different amides. The possibility of forming four types of *N*‐acylureas within the reaction system poses a primary challenge for this study: how to achieve highly selective synthesis of a single compound. Fortunately, this electrosynthesis method showed unparalleled advantages in selectively discriminating different amides. As summarized in Scheme 2B, multiple sets of two different amides could undergo highly selective Hofmann rearrangement reactions to generate single *N*‐acylureas. When Levetiracetam, prolinamide, pivalic amide, or adamantane‐1‐carboxamide was subjected to this transformation with aromatic carboxamides, the latter mainly acted only as nucleophiles to react with the isocyanates formed in situ from aliphatic carboxamides, resulting in the formation of the corresponding compounds **P11**‐**P29**. Such an electrosynthesis protocol enabled the preparation of various derivatives of Levetiracetam, trimethylgallic acid, probenecid, ibuprofen, and flumetnincam, as well as prolinamide. Notably, when enantiopure amides (the carbonyl group directly attached to the stereocenter) were employed, complete retention of configuration in the resulting products was observed (**P18‐P20**). Specifically, the combination of adamantane‐1‐carboxamide and acrylamide selectively generated product **P30** with a yield of 50%. In contrast, a change in reactivity was observed when formamide, butyramide, pentanamide, cyclopropyl carboxamide, cyclobutyl carboxamide, or acrylamide reacted with aromatic carboxamides. In these cases, aromatic carboxamides initially formed isocyanates, which then underwent nucleophilic attack by aliphatic carboxamides to afford the corresponding unsymmetrical *N*‐acylureas **P31**‐**P36** in 40%‐73% yields. Meanwhile, the structures of unsymmetrical *N*‐acylureas were confirmed by X‐ray crystallography of compounds **P27** and **P34** (CCDC 2443330, 2443332). Furthermore, the use of two structurally similar aromatic carboxamides also exhibited good chemical selectivity, allowing for the synthesis of single unsymmetrical *N*‐acylureas **P37‐P41** with moderate to high yields.

**SCHEME 3:**
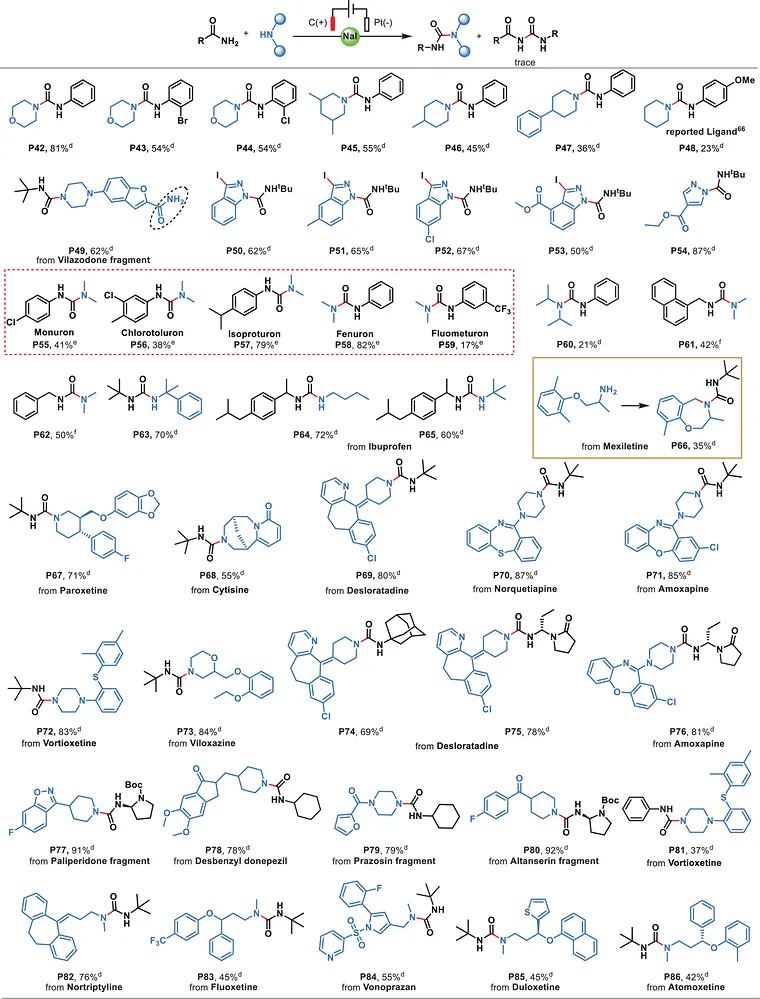
Substrate scope for unsymmetrical ureas and late‐stage modification of amine‐containing drugs. ^[d]^Reaction condition: NaI (2.0 equiv.), C(+)/Pt(‐),10 mA, MeCN (12 mL), air, rt, 6 h, isolated yield; ^[e]^Reaction condition: NaI (0.5 equiv.), C(+)/Pt(‐), 8 mA, MeCN (12 mL), Ar, rt, 10 h, isolated yield; ^[f]^Reaction condition: NaBr (3.0 equiv.), C(+)/Pt(‐), 20 mA, Na_2_CO_3_ (3.0 equiv.), MeCN:H_2_O (12 mL:1 mL), air, rt, 12 h, isolated yield.

Inspired by the excellent reactivity and efficiency demonstrated in the synthesis of *N*‐acylureas, we further investigated the involvement of amine nucleophiles in the electrochemical Hofmann rearrangement reaction. As shown in Scheme 3, under organic electrosynthetic conditions with sodium iodide as the electrolyte, cyclic amines such as morpholine, piperidine, and piperazine could react with aromatic carboxamides to generate unsymmetric ureas P42‐P49. It is worth noting that compound P48 served as an excellent ligand in an existing palladium‐catalyzed reaction [66]. In the case of the Vilazodone fragment's reaction with pivalic amide, the amide group in the biologically active substrate remained intact and delivered product P49 with a yield of 62%. When using indazole as the amine source, a tandem Hofmann rearrangement and C‐H iodination at the C3 position of indazole could occur, producing compounds P50‐P53 in 50% to 67% yields. Ethyl pyrazole‐4‐carboxylate also proved to be amenable to this reaction with pivalic amide to afford the corresponding unsymmetrical urea P54 in 87% yield. Beyond cyclic amines, linear primary and secondary amines were found to effectively participate in the reaction with both aromatic and aliphatic carboxamides. For instance, as exemplified by P55‐P59, the reaction of dimethylamine with aromatic carboxamides produced various herbicides, including Monuron, Chlorotoluron, Isoproturon, Fenuron, and Fluometuron, in 17%–82% yields. Additionally, diisopropylamine was able to react with benzamide to generate product P60, albeit at a low yield. By using improved reaction conditions (condition F, with NaBr as the electrolyte, extra addition of Na_2_CO_3_, and increased current), dimethylamine could react well with aliphatic carboxamides to afford compounds P61 and P62 in moderate yields. Moreover, n‐butylamine and tert‐butylamine effectively reacted with amides (including those derived from ibuprofen) to produce unsymmetric ureas P63‐65 with yields of 60% to 72%. Interestingly, employing metilethine as the amine source led to the synthesis of unsymmetrical urea P66 via a tandem Hofmann rearrangement and benzylic C‐H amination. To further illustrate the applicability of this protocol in pharmaceutical synthesis, a range of amine‐containing drugs (Mexiletine, Paroxetine, Cytisine, Desloratadine, Norquetiapine, Amoxapine, Vortioxetine, Viloxazine, Nortriptyline, Fluoxetine, Vonoprazan, Duloxetine, and Atomoxetine) as well as drug fragments (such as Paliperidone, Desbenzyl donepezil, Prazosin, and Altanserin) were evaluated and successfully converted to the corresponding unsymmetrical ureas P67‐P86 in moderate to excellent yields.

**SCHEME 4:**
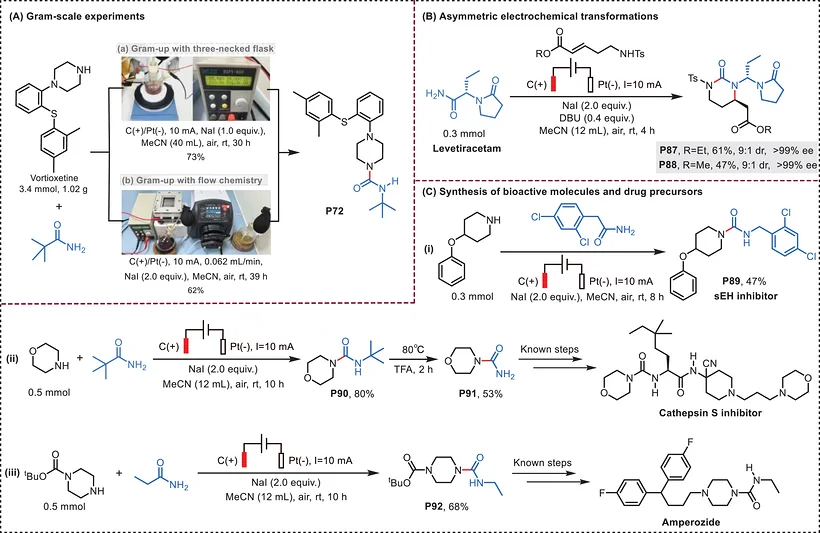
(A) Gram‐scale experiments. (B) Asymmetric electrochemical transformations. (C) Synthesis of bioactive molecules and drug precursors.

To further demonstrate the practical applicability of the electrochemical protocol, gram‐scale experiments, asymmetric electrochemical transformations, and the synthesis of bioactive molecules and drug precursors were conducted. As shown in Scheme 4Aa, 1.02 g of Vortioxetine could react efficiently with pivalic amide in a three‐necked flask by prolonging the reaction time, affording the compound **P72** in 73% yield. This easy setup and procedure offered a convenient approach for the preparation of unsymmetric ureas. In a continuous‐flow reactor (Scheme 4Ab), the reaction proceeded smoothly with moderate yields, highlighting its potential for larger‐scale production. Notably, under electrochemical conditions in the presence of DBU, enantiomerically pure Levetiracetam reacted with aminoalkyl *α,β*‐unsaturated esters to furnish six‐membered cyclic ureas in moderate yields with high diastereo‐ and enantioselectivity via a rearrangement/[4+2] annulation sequence. (Scheme 4B). Finally, this methodology enabled the synthesis of the soluble epoxide hydrolase (sEH) inhibitor **P89** [77] and provided the key precursors **P91** and **P92** for the cathepsin inhibitor [78] and Amperozide [79], respectively (Scheme 4C).

**SCHEME 5:**
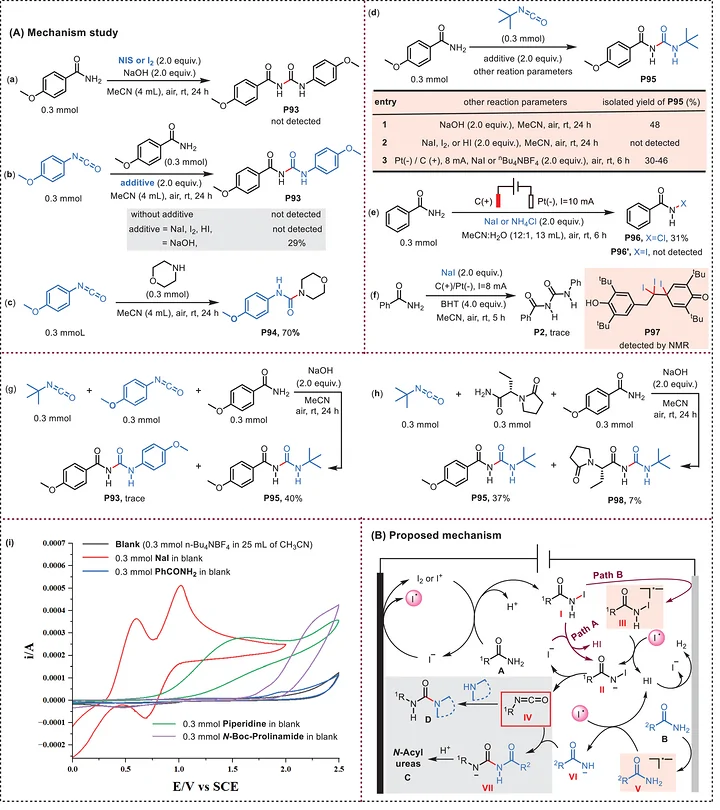
(A) Mechanism study. (B) Proposed mechanism study.

For mechanistic understanding, we conducted the following control experiments and cyclic voltammetry analyses: (1) The use of NIS or I_2_ as oxidant could not facilitate the Hofmann rearrangement of 4‐methoxybenzamide to form compound P93 (Scheme 5Aa). (2) When reacting 4‐methoxyphenyl isocyanate with 4‐methoxybenzamide, either without any additives or in the presence of NaI, I_2_, or HI (which might be present in the electrochemical system to activate amides or isocyanates), no product P93 was generated. However, the extra addition of NaOH led to the formation of compound P93 with a yield of 29% (Scheme 5Ab). These results suggested that the base played a crucial role in promoting the reaction between amides and isocyanates. (3) When morpholine was employed as a nucleophile to react with 4‐methoxy isocyanate, the target product P94 could be generated in 70% isolated yield without the addition of NaOH (Scheme 5Ac). This observation demonstrated that amines were strong nucleophiles capable of directly reacting with isocyanates in the absence of a base. (4) The investigation into the reaction feasibility of tert‐butyl isocyanate with 4‐methoxybenzamide showed that only under either electrochemical conditions or in the presence of NaOH could compound P95 be produced (Scheme 5Ad), suggesting that the electrochemical process may function as a base in the reaction. (5) Although *N*‐iodobenzamide was not observed, probably due to its high reactivity and instability under these conditions, the formation of *N*‐chlorobenzamide (P96) suggested that a haloamide may be a necessary intermediate in the electrochemical process (Scheme 5Ae). (6) Addition of the radical scavenger BHT under standard conditions dramatically inhibited the reaction, producing only trace amounts of the expected *N*‐acylurea P2 and, concurrently, iodide‐radical adduct P97 (Scheme 5Af). This control experiment indicated that the iodide radical might participate in the organic electrosynthesis. (7) The reaction of tert‐butyl isocyanate, 4‐methoxyphenyl isocyanate, and 4‐methoxybenzamide in the presence of NaOH led to the formation of compound P95 with a yield of 40%, while only trace amounts of compound P93 were produced. This finding suggested that tert‐butyl isocyanate was more reactive than aryl isocyanate when reacting with amides under basic conditions (Scheme 5Ag). (8) When Levetiracetam and 4‐methoxybenzamide simultaneously compete for reaction with tert‐butyl isocyanate, the yield of compound P95 (derived from 4‐methoxybenzamide) was higher than that of compound P97 (derived from Levetiracetam), which revealed that under basic conditions, aryl amides were more readily reactive with isocyanates (Scheme 5Ah). (9) Cyclic voltammetry analyses showed two oxidation peaks at 0.6 and 1.0 V for sodium iodide, which were characteristic of iodide anion oxidation and were lower than the oxidation peaks of amides or amines (Scheme 5Ai). These findings indicated that the iodide anion was preferentially oxidized during the electrochemical process, which not only facilitated subsequent transformations but also protected other amides and amine nucleophiles from electrooxidative degradation, ensuring the feasibility and good compatibility of the reaction.

On the basis of mechanism studies and previous relevant research [30, 31, 32, 33, 34, 35, 36], a plausible reaction pathway for the electrochemical Hofmann rearrangement toward the synthesis of unsymmetrical ureas was proposed (Scheme 5B). In the anode, iodide anion was first oxidized to form I^·^, I_2_, or I^+^, and both I^2^ and I^+^ could then respectively react with amide **A** to produce compound **I**. Two possible pathways might give rise to the nitrogen anion **II** from the compound I. (a) The iodide anion acted as a base, facilitating the formation of anion **II**. (b) Compound **I** was cathodically reduced to generate anionic radical **III**, which was then deprotonated by the iodine radical to afford nitrogen anion **II**. The newly‐formed species **II** underwent rearrangement to form isocyanate **IV**. In addition, amide **B** underwent cathodic reduction in the cathode and subsequent deprotonation by iodine radical to produce a more nucleophilic nitrogen anion **VI**, which further performed a nucleophilic attack on isocyanate **IV** to access intermediate **VII**. Finally, subsequent protonation of intermediate **VII** produced unsymmetrical *N*‐acylureas. When strong nucleophiles (i.e., amines) were present in the reaction system, they reacted directly with the isocyanate generated in situ to deliver unsymmetrical ureas. Throughout the reaction, anodic oxidation enabled the production of I^·^, I_2_, or I^+^ from Iodide anion, while cathodic reduction generated hydrogen gas from protons and thereby facilitated the deprotonation of certain amides. These processes worked cooperatively to regulate both the activity and selectivity of the two different amides, ultimately producing a single unsymmetrical *N*‐acylurea. Importantly, the relatively low potential of the iodide anion was critical for the good compatibility of this reaction with various amine nucleophiles.

## 3 Conclusions

In summary, we developed a practical electrochemical Hofmann rearrangement reaction involving amine or other amide nucleophiles using sodium iodide as the supporting electrolyte. When only one type of amide was present in the reaction system, a variety of corresponding symmetric *N*‐acylureas could be efficiently generated. Importantly, multiple sets of two different amides could selectively produce single unsymmetric *N*‐acylureas, overcoming the challenges of chemoselectivity control typically encountered in existing methods. In addition, oxidative‐labile amines were well‐tolerated in this electrosynthesis protocol, enabling the efficient synthesis of various unsymmetric ureas, including five herbicides and over twenty derivatives of amine‐containing drugs. The reaction was successfully scaled up to the gram scale with satisfactory yields using both continuous flow and three‐neck flask setups, thereby further demonstrating its practicality. Furthermore, mechanism studies indicated that the use of NaI played a vital role in guaranteeing the feasibility and compatibility of the reaction. Thus, this method offers a supplementary strategy for the efficient and highly selective synthesis of unsymmetric ureas and will further advance the development and application of methodologies based on Hofmann rearrangement.

## Funding

The Natural Science Foundation of China (22161056), the Guizhou Science and Technology Department (QKHJC‐ZK [2025]‐053, QKHJCVZD‐2026‐002, QKHPTRC‐GCC [2022]032‐1, QKHRCPTGCC‐2023‐003, 2024YJSKYJJ318, 2024 YJSKYJJ345), the Zunyi Science and Technology Department (ZSKH‐2025‐254), and the Graduate Education Innovation Program of Zunyi Medical University (ZYK226).

## Conflicts of Interest

The authors declare no conflict of interest.

## Supporting information




**Supporting File**: advs74920‐sup‐0001‐SuppMat.pdf.

## Data Availability

The data that supports the findings of this study are available in the supplementary material of this article

## References

[1] J. H. Seo and H. M. Ko , “Transition‐Metal‐Free Synthesis of Aromatic Amines via the Reaction of Benzynes with Isocyanates,” Tetrahedron Letters 59 (2018): 671–674, 10.1016/j.tetlet.2018.01.022.

[2] Y. Meng , E. Gnanamani , and R. N. Zare , “Catalyst‐Free Decarboxylative Amination of Carboxylic Acids in Water Microdroplets,” Journal of the American Chemical Society 145 (2023): 32–36, 10.1021/jacs.2c12236. 36566437

[3] X. Huang , T. Zhuang , P. A. Kates , H. Gao , X. Chen , and J. T. Groves , “Alkyl Isocyanates via Manganese‐Catalyzed C–H Activation for the Preparation of Substituted Ureas,” Journal of the American Chemical Society 139 (2017): 15407–15413, 10.1021/jacs.7b07658. 28976738

[4] L. Mistry , K. Mapesa , T. W. Bousfield , and J. E. Camp , “Synthesis of Ureas in the Bio‐alternative Solvent Cyrene,” Green Chemistry 19 (2017): 2123–2128, 10.1039/C7GC00908A.

[5] Y. Yamanashi , M. Xu , S. A. Kawashima , and M. Kanai , “Induced Bioconjugation via on‐Demand Isocyanate Formation,” Journal of the American Chemical Society 147 (2025): 27232–27237, 10.1021/jacs.5c11603. 40770739

[6] X. Wang , M. Nakajima , E. Serrano , and R. Martin , “Alkyl Bromides as Mild Hydride Sources in Ni‐Catalyzed Hydroamidation of Alkynes with Isocyanates,” Journal of the American Chemical Society 138 (2016): 15531–15534, 10.1021/jacs.6b10351. 27934006

[7] J. S. Derasp and A. M. Beauchemin , “Rhodium‐Catalyzed Synthesis of Amides from Functionalized Blocked Isocyanates,” ACS Catalysis 9 (2019): 8104–8109, 10.1021/acscatal.9b02641.

[8] S. Sun , B. Gao , J. Chen , K. B. Sharpless , and J. Dong , “Fluorosulfuryl Isocyanate Enabled SuFEx Ligation of Alcohols and Amines,” Angewandte Chemie International Edition 60 (2021): 21195–21199, 10.1002/anie.202105583. PMC9881234 34259368

[9] T. M. Townsend , W. H. Bernskoetter , N. Hazari , and B. Q. Mercado , “Dehydrogenative Synthesis of Carbamates from Formamides and Alcohols Using a Pincer‐Supported Iron Catalyst,” ACS Catalysis 11 (2021): 10614–10624, 10.1021/acscatal.1c02718.

[10] J. S. Nowick , N. A. Powell , T. M. Nguyen , and G. Noronha , “An Improved Method for the Synthesis of Enantiomerically Pure Amino Acid Ester Isocyanates,” The Journal of Organic Chemistry 57 (1992): 7364–7366, 10.1021/jo00052a069.

[11] J. S. Nowick , D. L. Holmes , G. Noronha , E. M. Smith , T. M. Nguyen , and S.‐L. Huang , “Synthesis of Peptide Isocyanates and Isothiocyanates,” The Journal of Organic Chemistry 61 (1996): 3929–3934, 10.1021/jo960038n. 11667260

[12] K. Kurita and Y. Iwakura , “Trichloromethyl Chloroformate as a Phosgene Equivalent: 3‐isocyanatopropanoyl Chloride,” Organic Syntheses 59 (1979): 195.

[13] S. T. Sigurdsson , B. Seeger , U. Kutzke , and F. Eckstein , “A Mild and Simple Method for the Preparation of Isocyanates from Aliphatic Amines Using Trichloromethyl Chloroformate. Synthesis of an Isocyanate Containing an Activated Disulfide,” The Journal of Organic Chemistry 61 (1996): 3883–3884, 10.1021/jo9521920. 11667245

[14] J. H. Tsai , L. R. Takaoka , N. A. Powell , and J. S. Nowick , “Synthesis of Amino Acid Ester Isocyanates: Methyl (S)‐2‐Isocyanato‐3‐Phenylpropanoate: Benzenepropanoic Acid, α‐Isocyanato‐, Methyl Ester,(S),” Organic Syntheses 78 (2002): 220.

[15] Y. C. Charalambides and S. C. Moratti , “Comparison of Base‐Promoted and Self‐Catalyzed Conditions in the Synthesis of Isocyanates from Amines Using Triphosgene,” Synthetic Communications 37 (2007): 1037–1044, 10.1080/00397910601055156.

[16] H.‐J. Knölker , T. Braxmeier , and G. Schlechtingen , “A Novel Method for the Synthesis of Isocyanates under Mild Conditions,” Angewandte Chemie, International Edition in English 34 (1995): 2497.

[17] S. Lemouzy , A. Delavarde , F. Lamaty , X. Bantreil , J. Pinaud , and S. Caillol , “Lignin‐based Bisguaiacol Diisocyanate: a Green Route for the Synthesis of Biobased Polyurethanes,” Green Chemistry 25 (2023): 4833–4839, 10.1039/D3GC00704A.

[18] F. M. Miloserdov and V. V. Grushin , “Palladium‐Catalyzed Aromatic Azidocarbonylation,” Angewandte Chemie International Edition 51 (2012): 3668–3672, 10.1002/anie.201200078. 22374705

[19] F. M. Miloserdov , C. L. McMullin , M. M. Belmonte , et al., “The Challenge of Palladium‐Catalyzed Aromatic Azidocarbonylation: from Mechanistic and Catalyst Deactivation Studies to a Highly Efficient Process,” Organometallics 33 (2014): 736–752, 10.1021/om401126m.

[20] D. Saylik , M. J. Horvath , P. S. Elmes , W. R. Jackson , C. G. Lovel , and K. Moody , “Preparation of Isocyanates from Primary Amines and Carbon Dioxide Using Mitsunobu Chemistry 1,” The Journal of Organic Chemistry 64 (1999): 3940–3946, 10.1021/jo982362j.

[21] Q. Zhuo , J. Yang , Z. Mo , et al., “Dinitrogen Cleavage and Functionalization with Carbon Dioxide in a Dititanium Dihydride Framework,” Journal of the American Chemical Society 144 (2022): 6972–6980, 10.1021/jacs.2c01851. 35380823

[22] E. V. Vinogradova , B. P. Fors , and S. L. Buchwald , “Palladium‐Catalyzed Cross‐Coupling of Aryl Chlorides and Triflates with Sodium Cyanate: a Practical Synthesis of Unsymmetrical Ureas,” Journal of the American Chemical Society 134 (2012): 11132–11135, 10.1021/ja305212v. PMC3472423 22716197

[23] E. F. V. Scriven and K. Turnbull , “Azides: Their Preparation and Synthetic Uses,” Chemical Reviews 88 (1988): 297–368.

[24] T. Mukaiyama and H. Nohira , “Dehydration Reactions of Hydroxamic Acids. A New Method for the Preparation of Isocyanates,” The Journal of Organic Chemistry 26 (1961): 782–784, 10.1021/jo01062a033.

[25] P. Spieß , J. Brześkiewicz , and N. Maulide , “Deprotective Lossen Rearrangement: a Direct and General Transformation of Nms‐amides to Unsymmetrical Ureas,” Chemical Science 15 (2024): 15799–15803. 10.1039/d4sc04974h PMC11385062 39268216

[26] A. K. Ghosh , A. Sarkar , and M. Brindisi , “The Curtius Rearrangement: Mechanistic Insight and Recent Applications in Natural Product Syntheses,” Organic & Biomolecular Chemistry 16 (2018): 2006–2027. 10.1039/c8ob00138c PMC5864567 29479624

[27] J. P. Barbor , K. N. Flesch , M. Chan , H. R. Ang , and B. M. Stoltz , “Enantioselective Spirocyclization of Pd‐Enolates and Isocyanates,” Angewandte Chemie International Edition 64 (2025): 202502583, 10.1002/anie.202502583. PMC12124958 40148236

[28] A. W. Hofmann , “Ueber die Einwirkung des Broms in Alkalischer Lösung auf Amide,” Berichte der deutschen chemischen Gesellschaft 14 (1881): 2725–2736, 10.1002/cber.188101402242.

[29] W. Hofmann and B. Dtsch , “Ueber die Einwirkung des Broms in Alkalischer Lösung auf Amide,” Berichte der deutschen chemischen Gesellschaft 15 (1882): 762–775, 10.1002/cber.188201501166.

[30] J. S. Amato , C. Bagner , R. J. Cvetovich , S. Gomolka , F. W. Hartner , and R. Reamer , “Development of the Hofmann Rearrangement of N α‐Tosylasparagine through Calorimetric and NMR Analysis,” The Journal of Organic Chemistry 63 (1998): 9533–9534, 10.1021/jo980799l.

[31] P. Gogoi and D. Konwar , “An Efficient Modification of the Hofmann Rearrangement: Synthesis of Methyl Carbamates,” Tetrahedron Letters 48 (2007): 531–533, 10.1016/j.tetlet.2006.11.134.

[32] D. Pradip , “Recent Advances in the Hofmann Rearrangement and Its Application to Natural Product Synthesis,” Current Organic Chemistry 23 (2019): 2402–2435.

[33] H. E. Baumgarten , H. L. Smith , and A. Staklis , “Reactions of Amines. XVIII. Oxidative Rearrangement of Amides with Lead Tetraacetate,” The Journal of Organic Chemistry 40 (1975): 3554–3561, 10.1021/jo00912a019.

[34] H. Senanayake , L. E. Fredenburgh , R. A. Reamer , R. D. Larsen , T. R. Verhoeven , and P. J. Reider , “Nature of N‐Bromosuccinimide in Basic Media: the True Oxidizing Species in the Hofmann Rearrangement,” Journal of the American Chemical Society 116 (1994): 7947–7948, 10.1021/ja00096a082.

[35] X. Huang , M. Seid , and J. W. Keillor , “A Mild and Efficient Modified Hofmann Rearrangement,” The Journal of Organic Chemistry 62 (1997): 7495–7496, 10.1021/jo9708553. 11671873

[36] N. Okamoto , Y. Miwa , H. Minami , K. Takeda , and R. Yanada , “Concise One‐Pot Tandem Synthesis of Indoles and Isoquinolines from Amides,” Angewandte Chemie International Edition 48 (2009): 9693–9696, 10.1002/anie.200904960. 19899177

[37] J. Yoshida , K. Kataoka , R. Horcajada , and A. Nagaki , “Modern Strategies in Electroorganic Synthesis,” Chemical Reviews 108 (2008): 2265–2299, 10.1021/cr0680843. 18564879

[38] M. Yan , Y. Kawamata , and P. S. Baran , “Synthetic Organic Electrochemical Methods Since 2000: on the Verge of a Renaissance,” Chemical Reviews 117 (2017): 13230–13319, 10.1021/acs.chemrev.7b00397. PMC5786875 28991454

[39] P. F. Ma , Z.‐R. Liu , S.‐S. Xu , et al., “Recent Advances in Organic Electrosynthesis Employing Transition Metal Complexes as Electrocatalysts,” Science Bulletin 66 (2021): 2412–2429, 10.1016/j.scib.2021.07.011. 36654127

[40] M.‐J. Luo , Q. Xiao , and J.‐H. Li , “Electro‐/Photocatalytic Alkene‐derived Radical Cation Chemistry: Recent Advances in Synthetic Applications,” Chemical Society Reviews 51 (2022): 7206–7237, 10.1039/D2CS00013J. 35880555

[41] A. Malapit , M. B. Prater , J. R. Cabrera‐Pardo , et al., “Advances on the Merger of Electrochemistry and Transition Metal Catalysis for Organic Synthesis,” Chemical Reviews 122 (2022): 3180–3218, 10.1021/acs.chemrev.1c00614. PMC9714963 34797053

[42] J. Yount and D. G. Piercey , “Electrochemical Synthesis of High‐Nitrogen Materials and Energetic Materials,” Chemical Reviews 122 (2022): 8809–8840, 10.1021/acs.chemrev.1c00935. 35290022

[43] J. Zhong , C. Ding , H. Kim , T. McCallum , and K.‐Y. Ye , “Alternating Current Electrolysis: a Photoredox Catalysis Mimic and Beyond,” Green Synthesis and Catalysis 3 (2022): 4–10.

[44] J. Rein , S. B. Zacate , K. Mao , and S. Lin , “A Tutorial on Asymmetric Electrocatalysis,” Chemical Society Reviews 52 (2023): 8106–8125, 10.1039/D3CS00511A. PMC10842033 37910160

[45] Y. Wang , S. Dana , H. Long , et al., “Electrochemical Late‐Stage Functionalization,” Chemical Reviews 123 (2023): 11269–11335, 10.1021/acs.chemrev.3c00158. PMC10571048 37751573

[46] L. Zeng , J. Wang , D. Wang , H. Yi , and A. Lei , “Comprehensive Comparisons between Directing and Alternating Current Electrolysis in Organic Synthesis,” Angewandte Chemie International Edition 62 (2023): 202309620, 10.1002/anie.202309620. 37606535

[47] W. Zeng , Y. Wang , C. Peng , and Y. Qiu , “Organo‐mediator Enabled Electrochemical Transformations,” Chemical Society Reviews 54 (2025): 4468–4501, 10.1039/D4CS01142B. 40151968

[48] X. Hu , C. Zheng , X. Song , B. Hasimujiang , M. Chen , and Z. Ruan , “Manganese‐Catalyzed Electrochemical Diazidation of Dehydroalanine Peptides,” Advanced Science 12 (2025): 2502711. 10.1002/advs.202502711 PMC12302629 40349176

[49] T. Shono , Y. Matsumura , S. Yamane , and S. Kashimura , “The Hofmann Rearrangement Induced by Electroorganic Method,” Chemistry Letters 11 (1982): 565–568, 10.1246/cl.1982.565.

[50] Y. Matsumura , Y. Satoh , T. Maki , and O. Onomura , “The Electrochemically Induced Hofmann Rearrangement and Its Comparison with the Classic Hofmann Rearrangement,” Electrochimica Acta 45 (2000): 3011–3020, 10.1016/S0013-4686(00)00380-7.

[51] L. Li , M. Xue , X. Yan , W. Liu , K. Xu , and S. Zhang , “Electrochemical Hofmann Rearrangement Mediated by NaBr: Practical Access to Bioactive Carbamates,” Organic & Biomolecular Chemistry 16 (2018): 4615–4618, 10.1039/C8OB01059E. 29900466

[52] T. Cantin , A. B. Charette , T. Poisson , and P. Jubault , “Synthesis of Cyclopropylamines through an Electro‐induced Hofmann Rearrangement,” Synthesis 55 (2023): 2943–2950.

[53] B. K. Malviya , C. Bottecchia , K. Stone , et al., “Multigram Electrochemical Hofmann Rearrangement Using a Spinning Three‐Dimensional Anode,” Organic Process Research & Development 27 (2023): 2183–2191, 10.1021/acs.oprd.3c00332.

[54] M. Wang and P. Wilson , “An Electrochemical Hofmann Rearrangement on Acrylamide Copolymers,” Polymer Chemistry 14 (2023): 3057–3062, 10.1039/D3PY00594A.

[55] J. L. Archibald , D. R. Beardsley , G. M. F. Bisset , et al., “Antihypertensive Ureidopiperidines,” Journal of Medicinal Chemistry 23 (1980): 857–861, 10.1021/jm00182a009. 7401114

[56] T. Klabunde , K. U. Wendt , D. Kadereit , et al., “Acyl Ureas as Human Liver Glycogen Phosphorylase Inhibitors for the Treatment of Type 2 Diabetes,” Journal of Medicinal Chemistry 48 (2005): 6178–6193, 10.1021/jm049034y. 16190745

[57] W. M. Kazmierski , R. Hamatake , M. Duan , et al., “Discovery of Novel Urea‐Based Hepatitis C Protease Inhibitors with High Potency against Protease‐Inhibitor‐Resistant Mutants,” Journal of Medicinal Chemistry 55 (2012): 3021–3026, 10.1021/jm201278q. 22471376

[58] K. Ghosh and M. Brindisi , “Urea Derivatives in Modern Drug Discovery and Medicinal Chemistry,” Journal of Medicinal Chemistry 63 (2020): 2751–2788, 10.1021/acs.jmedchem.9b01541. PMC7266097 31789518

[59] Z. Li , Z. Wu , and F. Luo , “Synthesis and Antifungal Activities of Alkyl N‐(1,2,3‐Thiadiazole‐4‐Carbonyl) Carbamates and S‐Alkyl N‐(1,2,3‐Thiadiazole‐4‐Carbonyl) Carbamothioates,” Journal of Agricultural and Food Chemistry 53 (2005): 3872–3876, 10.1021/jf0501746. 15884810

[60] J.‐F. Zhang , J.‐Y. Xu , B.‐L. Wang , et al., “Synthesis and Insecticidal Activities of Novel Anthranilic Diamides Containing Acylthiourea and Acylurea,” Journal of Agricultural and Food Chemistry 60 (2012): 7565–7572, 10.1021/jf302446c. 22812664

[61] S. H. McCooey and S. J. Connon , “Urea‐ and Thiourea‐Substituted Cinchona Alkaloid Derivatives as Highly Efficient Bifunctional Organocatalysts for the Asymmetric Addition of Malonate to Nitroalkenes: Inversion of Configuration at C9 Dramatically Improves Catalyst Performance,” Angewandte Chemie International Edition 44 (2005): 6367–6370, 10.1002/anie.200501721. 16136619

[62] M. S. Manna and S. Mukherjee , “Organocatalytic Enantioselective Formal C(sp 2)–H Alkylation,” Journal of the American Chemical Society 137 (2015): 130–133, 10.1021/ja5117556. 25551319

[63] S. C. Mallojjala , R. Sarkar , R. W. Karugu , et al., “Mechanism and Origin of Remote Stereocontrol in the Organocatalytic Enantioselective Formal C(sp 2)–H Alkylation Using Nitroalkanes as Alkylating Agents,” Journal of the American Chemical Society 144 (2022): 17399–17406, 10.1021/jacs.2c02941. PMC10322726 36108139

[64] A. Kondoh , R. Ojima , S. Ishikawa , and M. Terada , “Construction of 1,3‐Nonadjacent Stereogenic Centers through Enantioselective Addition of α‐Thioacetamides to α‐Substituted Vinyl Sulfones Catalyzed by Chiral Strong Brønsted Base,” Advanced Science 11 (2024): 2308020, 10.1002/advs.202308020. PMC10916638 38126668

[65] Y. Kuninobu , H. Ida , M. Nishi , and M. Kanai , “A Meta‐selective C–H Borylation Directed by a Secondary Interaction between Ligand and Substrate,” Nature Chemistry 7 (2015): 712–717, 10.1038/nchem.2322. 26291942

[66] K. E. Houghtling , A. M. Canfield , and S. M. Paradine , “Convergent Synthesis of Dihydrobenzofurans via Urea Ligand‐Enabled Heteroannulation of 2‐Bromophenols with 1,3‐Dienes,” Organic Letters 24 (2022): 5787–5790, 10.1021/acs.orglett.2c02301. PMC9380016 35904546

[67] J. Vaith , D. Rodina , G. C. Spaulding , and S. M. Paradine , “Pd‐Catalyzed Heteroannulation Using N ‐Arylureas as a Sterically Undemanding Ligand Platform,” Journal of the American Chemical Society 144 (2022): 6667–6673, 10.1021/jacs.2c01019. PMC9026275 35380831

[68] X. Xie , S. Dong , K. Hong , J. Huang , and X. Xu , “Catalytic Asymmetric Difluoroalkylation Using in Situ Generated Difluoroenol Species as the Privileged Synthon,” Advanced Science 11 (2024): 2307520, 10.1002/advs.202307520. PMC11005710 38318687

[69] S. M. Paradine , “Ureas and Their Derivatives as Ligands in Transition Metal Catalysis,” Tetrahedron 184 (2025): 134781, 10.1016/j.tet.2025.134781. PMC12201964 40583974

[70] K. Bjerglund , A. T. Lindhardt , and T. Skrydstrup , “Palladium‐Catalyzed N ‐Acylation of Monosubstituted Ureas Using Near‐Stoichiometric Carbon Monoxide,” The Journal of Organic Chemistry 77 (2012): 3793–3799, 10.1021/jo3000767. 22458554

[71] B. Chen , J.‐B. Peng , J. Ying , X. Qi , and X.‐F. Wu , “A Palladium‐Catalyzed Domino Procedure for the Synthesis of Unsymmetrical Ureas,” Advanced Synthesis & Catalysis 360 (2018): 2820–2824, 10.1002/adsc.201800496.

[72] S. Y. Kim and H. N. Lim , “Methyl Pyruvate Oxime as a Carbonyl Synthon: Synthesis of Ureas, Carbamates, Thiocarbamates, and Anilides,” Organic Letters 26 (2024): 3850–3854, 10.1021/acs.orglett.4c01007. 38683648

[73] C. Zhou , D. Singh , and B. A. Arndtsen , “A Versatile Carbonylative Approach to Ureas and Carbamates through Light Activated Nickel Catalyzed Formation of Aliphatic Isocyanates,” Angewandte Chemie International Edition 64 (2025): 202423519, 10.1002/anie.202423519. 39945527

[74] J. Wang , S. Wang , Z. Wei , et al., “Synchronous Recognition of Amines in Oxidative Carbonylation toward Unsymmetrical Ureas,” Science 386 (2024): 776–782, 10.1126/science.adl0149. 39541452

[75] X. Zhao , J. Wang , D. Guo , et al., “A Novel Electrochemical Hofmann‐type Rearrangement Enables Facile Access to α‐oxoisocyanates for the Synthesis of N ‐carbamoylacetamides,” Green Chemistry 27 (2025): 2751–2759, 10.1039/D4GC05807K.

[76] L. Song , Y. Meng , T. Zhao , et al., “Unified and Green Oxidation of Amides and Aldehydes for the Hofmann and Curtius Rearrangements,” Green Chemistry 26 (2024): 428–438, 10.1039/D3GC04355J.

[77] A. B. Eldrup , F. Soleymanzadeh , N. A. Farrow , A. Kukulka , and S. De Lombaert , “Optimization of Piperidyl‐ureas as Inhibitors of Soluble Epoxide Hydrolase,” Bioorganic & Medicinal Chemistry Letters 20 (2010): 571–575, 10.1016/j.bmcl.2009.11.091. 19969453

[78] J. C. Lorenz , C. A. Busacca , X. Feng , et al., “Large‐Scale Asymmetric Synthesis of a Cathepsin S Inhibitor,” The Journal of Organic Chemistry 75 (2010): 1155, 10.1021/jo9022809. 20102230

[79] J. Li , D. Peng , and D. Luo , “Zinc Carbonyl Reagent and its Preparation Method and Application, CN Pat. CN119241572 A”, 2025, https://pss‐system.cponline.cnipa.gov.cn/retrieveList?prevPageTit=changgui.

