# Supplementary material for: Electrochemical Hofmann Rearrangement for Modular Synthesis of Unsymmetrical Ureas and Late‐Stage Modification of Drugs
Source: Adv Sci (Weinh). 2026 Mar 25;13(31):e74920. doi: 10.1002/advs.74920 (PMC13587983; doi:10.1002/advs.74920)
Supplement: Supplementary file 1 [file ADVS-13-e74920-s001.pdf]

Supporting Information

Electrochemical Hofmann Rearrangement for Modular Synthesis of Unsymmetrical Ureas and Late-Stage Modification of Drugs

Xue-Qing Mou,[+,a,b] Xiao Zha,[+,a] Yu-Rui Jian,[a] Shao-Xing Zhong,[a] Xin-Yu Fu,[a] Sheng-Yang Xu,[a] Wei Xiang,[a] Bao-Dong Cui,[a,b] Yun Zhang,[a,b] Yong-Zheng Chen*[,a,b]

[a] Dr. X.-Q. Mou, X. Zha, Y.-R. Jian, S.-X. Zhong, X. -Y. Fu, S -Y. Xu, W. Xiang, Dr. B.-D. Cui, Dr. Y. Zhang, Prof. Dr. Y.-Z. Chen
Guizhou Provincial Key Laboratory of Innovation and Manufacturing for Pharmaceuticals, School of Pharmacy
Zunyi Medical University
No. 6 West Xuefu Rd. Zunyi 563006 (China)
E-mail: mouxq@zmu.edu.cn; yzchen@zmu.edu.cn

[b] Dr. X.-Q. Mou, Dr. B.-D. Cui, Dr. Y. Zhang, Prof. Dr. Y.-Z. Chen
Key Laboratory of Basic Pharmacology of Ministry of Education and Joint International Research Laboratory of Ethnomedicine of Ministry of Education Zunyi Medical University
No. 6 West Xuefu Rd. Zunyi 563006 (China)

[+] These authors contributed equally to this work.

Contents

1. General information S1

2. Optimization of the reaction conditions S1

3. General procedures and characterization of products S4

    3.1 General procedures of Hofmann rearrangement reaction S4

    3.2 Characterization datas of products S6

4. Gram-scale reaction of P72 .... S36

5. Electrochemical continuous-flow experiment S37

6. Synthetic applications S39

7. Mechanism studies S43

    7.1 Cyclic voltammetry S43

    7.2 Controlled experiments S44

8. X-ray crystallographic data S50

9. Referances S52

10. HPLC data S53

11. NMR spectra S57

1. General information

All commercial reagents and solvents were purchased from commercial suppliers and used as received unless otherwise indicated. Analytical thin layer chromatography (TLC) was performed on 0.25 mm silica gel F254 plates and column chromatography was purified using 300-400 mesh silica gel. The instrument for electrolysis is dual display potentiostat HSPY-36-03 (made in China). The anode: a graphite rod (ϕ 6 mm), and the cathode: a platinum plate (15 mm×15 mm×0.3 mm) (from Gaoshi Ruilian Optoelectronic Technology Co., Ltd.). Cyclic voltammograms were obtained on a CHI660E instrument (from Shanghai Chenhua Instrument Co., Ltd.). The NMR spectra were recorded on a Bruker AVANCE NEO 400 and reported in parts per million (ppm), coupling constant (J) was reported in hertz unit (Hz). Data were reported as chemical shifts in ppm relative to $CDCl_3$ (7.26 ppm) or DMSO-d_6 (2.50 ppm) for ^1H and $CDCl_3$ (77.16 ppm) or DMSO-d_6 (39.52 ppm) for ^{13}C respectively. Multiplicities are recorded as s (singlet), d (doublet), t (triplet), q (quartet), dd (doublet of doublets), m (multiplet). High-resolution mass spectra (HRMS) were recorded by ESI ionization sources. The continuous-flow equipment was purchased from the EZONE (model: PER-10). X-ray crystallographic data was obtained with a diffractometer Rigaku Oxford Diffraction Supernova Dual Source. The enantiomeric excess (ee) values of chiral compounds were determined by chiral HPLC, using AD-H chiral column eluted with a mixture of hexane and isopropyl alcohol.

2. Optimization of the reaction conditions

Table S1. Optimization for synthesis of unsymmetric *N*-acylurea reaction conditions.[a]

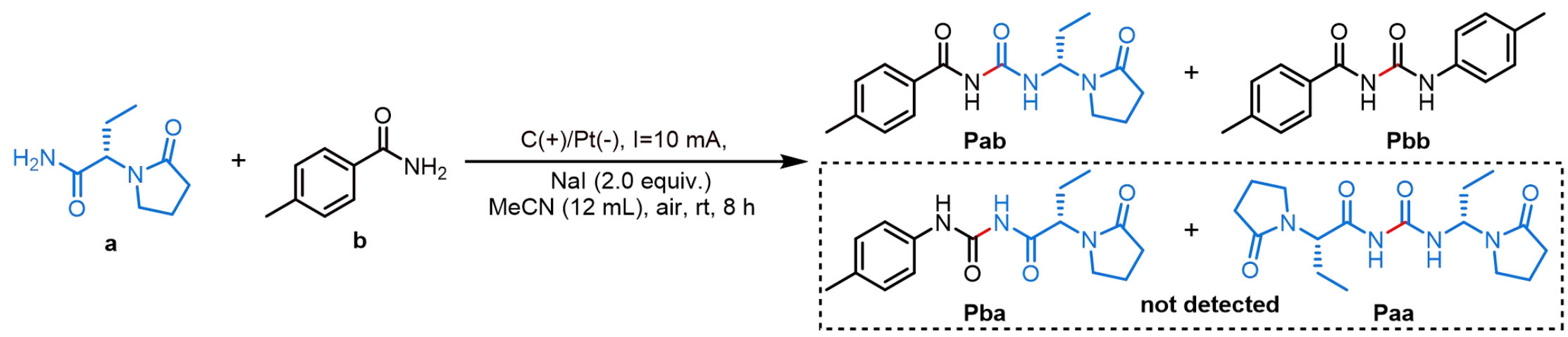

Entry	Deviation from standard conditions	Yield of **Pab** %[b]	Yield of **Pbb** %[b]
1	None	61	trace
2	MeOH, H_2O, HFIP instead of MeCN	n.d.	n.d.
3	DCM instead of MeCN	37	trace
4	$^{n}Bu_4NBF_4$ (2.0 equiv.), $^{n}Bu_4NCl$ (2.0 equiv.), $^{n}Bu_4NBr$ (2.0 equiv.), $^{n}Bu_4NOAc$ (2.0 equiv.) instead of NaI	n.d.	n.d.

5	^{n}Bu$_4$NI (2.0 equiv.) instead of NaI	18	trace
6	NH_4I (2.0 equiv.) instead of NaI	n.d.	n.d.
7	KI (2.0 equiv.) instead of NaI	32	15
8	NaBr (2.0 equiv.) instead of NaI	22	trace
9	NaCl (2.0 equiv.) instead of NaI	n.d.	n.d.
10	Cu (-) / C (+) instead of Pt (-) / C (+)	28	trace
11	Ni (-) / C (+) instead of Pt (-) / C (+)	43	trace
12	Pt (-) / Pt (+) instead of Pt (-) / C (+)	48	trace
13	C (-) / C (+) instead of Pt (-) / C (+)	40	trace
14	NaI (0.5 equiv.) instead of NaI (2.0 equiv.)	26	trace
15	NaI (1.0 equiv.) instead of NaI (2.0 equiv.)	45	trace
16	NaI (3.0 equiv.) instead of NaI (2.0 equiv.)	54	trace
17	8 mA instead of 10 mA	34	trace
18	12 mA instead of 10 mA	43	trace
19	6 h instead of 8 h	49	trace
20	10 h instead of 8 h	54	9
21	Ar instead of air	55	trace
22	O_2 instead of air	48	12
23	**b** (1.0 equiv.) instead of (1.5 equiv.)	39	trace
24	**b** (2.0 equiv.) instead of (1.5 equiv.)	56	12
25	**b** (1.0 equiv.), Without **a**	--	67
26	**b** (1.0 equiv.); 8 mA, 6 h instead of 10 mA, 8 h; Without **a**	--	78
27[c]	**a** (1.0 equiv.), Without **b**	--	--
28	No electricity	n.d.	n.d.

[a] Without further notification, the reactions were performed with **a** (51.1 mg, 0.3 mmol, 1.0 equiv.), **b** (60.8 mg, 0.45 mmol, 1.5 equiv.), sodium iodide (89.9 mg, 0.6 mmol, 2.0 equiv.), in MeCN (12 mL) within an undivided cell (a three-necked flask equipped with a graphite rod and a platinum plate), while maintaining a constant current of 10 mA (J = 4.4 mA/cm^2) under air at room temperature for 8 hours; [b] Isolated yields; [c]**Paa** not detected; n.d.: not detected.

Table S2. Optimization for synthesis of *N*-acylurea reaction conditions.[a]

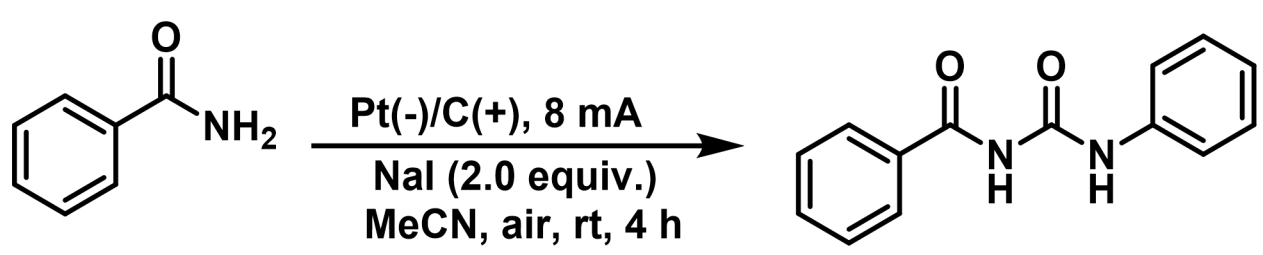

Entry	Deviation from standard conditions	Yield of **P2** %[b]
1	None	90
2	DCM, MeOH, H_2O, HFIP instead of MeCN	not detected
3	MeCN/DCM=5:1 instead of MeCN	67
4	MeCN/THF=5:1 instead of MeCN	70
5	MeCN/MeOH=5:1 instead of MeCN	not detected
6	^{n}Bu$_4$NBF$_4$ (2.0 equiv.), ^{n}Bu$_4$NCl (2.0 equiv.), ^{n}Bu$_4$NBr (2.0 equiv.), ^{n}Bu$_4$NOAc (2.0 equiv.) instead of NaI	not detected
7	^{n}Bu$_4$NI (2.0 equiv.), NH_4I (2.0 equiv.) instead of NaI	not detected
8	KI (2.0 equiv.) instead of NaI	65
9	NaBr (2.0 equiv.) instead of NaI	65

10	NaCl (2.0 equiv.) instead of NaI	not detected
11	Ni (-) / C (+) instead of Pt (-) / C (+)	75
12	Pt (-) / Carbon cloth (+) instead of Pt (-) / C (+)	58
13	Pt (-) / Carbon felt (+) instead of Pt (-) / C (+)	63
14	Pt (-) / Pt (+) instead of Pt (-) / C (+)	72
15	C (-) / C (+) instead of Pt (-) / C (+)	61
16	NaI (0.5 equiv.) instead of NaI (2.0 equiv.)	43
17	NaI (1.0 equiv.) instead of NaI (2.0 equiv.)	67
18	4 mA instead of 8 mA	45
19	6 mA instead of 8 mA	88
20	10 mA instead of 8 mA	70
21	3 h instead of 4 h	72
22	5 h instead of 4 h	85
23	Ar instead of air	86
24	O_2 instead of air	69
25	No electricity	not detected

[a] Without further notification, the reactions were performed with benzamide (36.3 mg, 0.3 mmol, 1.0 equiv.), sodium iodide (89.9 mg, 0.6 mmol, 2.0 equiv.), in MeCN (12 mL) within an undivided cell (a three-necked flask equipped with a graphite rod and a platinum plate), while maintaining a constant current of 8 mA (J = 3.6 mA/cm^2) under air at room temperature for 4 hours; [b] Isolated yields.

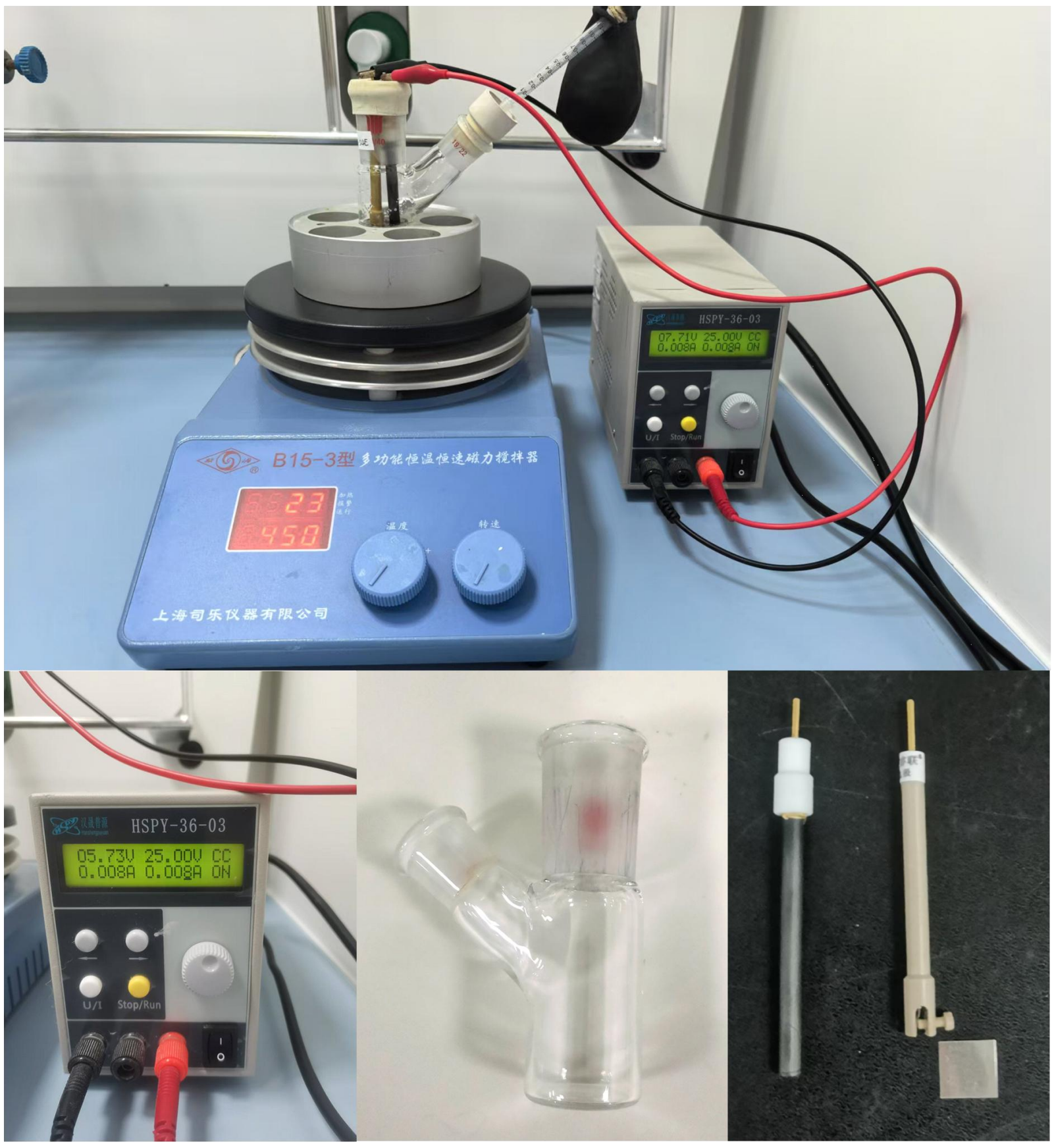

Figure S1. Details of the reaction equipment: A graphite rod (ϕ 6 mm, about 20 mm immersion depth in solution) anode electrode and a platinum plate (15 mm × 15 mm × 0.3 mm) cathode electrode. The immersed surface area of the platinum plate was about 2.25 cm^2 and the distance between the electrodes was about 5 mm.

3. General procedures and characterization of products

3.1 General procedures of Hofmann rearrangement reaction

General procedure A for *N*-acylurea synthesis:

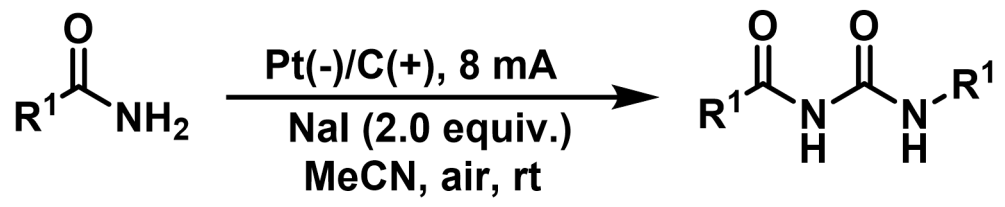

Amides (0.3 mmol, 1.0 equiv.), sodium iodide (0.6 mmol, 2.0 equiv.), and MeCN (12 mL) were added to 25 mL three-neck flask with a teflon-coated stir bar. A graphite rod was used as the anode, and a platinum plate was used as the cathode. The resulting mixture was stirred and electrolyzed with constant current 8 mA (J = 3.6 mA/cm^2) under air at room temperature until complete consumption of the amide (monitored by TLC). Then, transferred to a round-bottomed flask, concentrated *in vacuo*. The solutions were extracted with ethyl acetate and saturated sodium bisulfite, and the aqueous phase was extracted twice with ethyl acetate. The organic solution was dried over anhydrous sodium sulfate, filtered, and concentrated *in vacuo*, the residue was purified by flash chromatography on silica gel to obtain the products (**P1-P8**).

General procedure B for *N*-acylurea synthesis:

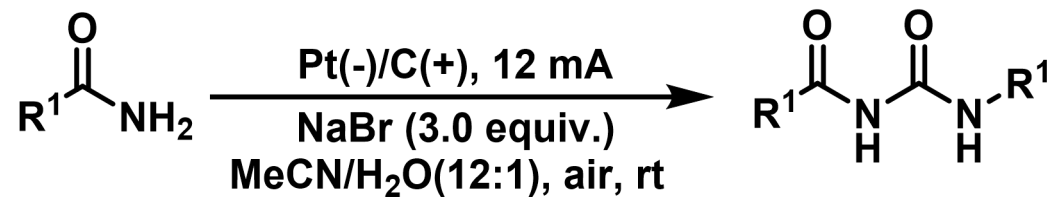

Amides (0.3 mmol, 1.0 equiv.), sodium bromide (0.9 mmol, 3.0 equiv.), and MeCN/H_2O (12:1, 13 mL) were added to 25 mL three-neck flask with a teflon-coated stir bar. A graphite rod was used as the anode, and a platinum plate was used as the cathode. The resulting mixture was stirred and electrolyzed with constant current 12 mA (J = 5.3 mA/cm^2) under air at room temperature until complete consumption of the amide (monitored by TLC). Then, transferred to a round-bottomed flask, concentrated *in vacuo*. The solutions were extracted with ethyl acetate and saturated sodium bisulfite, and the aqueous phase was extracted twice with ethyl acetate. The organic solution was dried over anhydrous sodium sulfate, filtered, and concentrated *in vacuo*, the residue was purified by flash chromatography on silica gel to obtain the products (**P9, P10**).

General procedure C for *N*-acylurea synthesis:

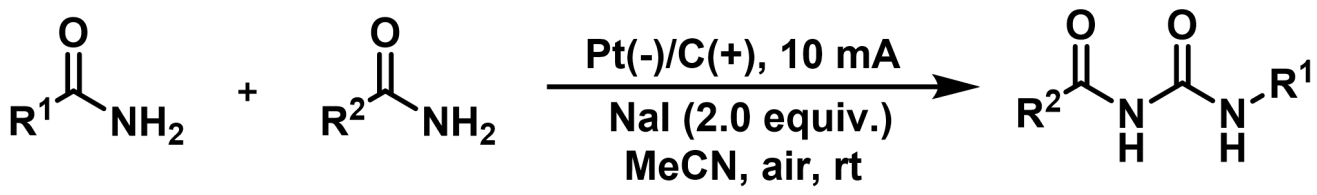

Amide (R^1CONH_2, 0.3 mmol, 1.0 equiv.), amide (R^2CONH_2, 0.45 mmol, 1.5 equiv.), sodium iodide (0.6 mmol, 2.0 equiv.), and MeCN (12 mL) were added to 25 mL three-neck flask with a teflon-coated stir bar. A graphite rod was used as the anode, and a platinum plate was used as the cathode. The resulting mixture was stirred and electrolyzed with constant current 10 mA (J = 4.4 mA/cm^2) under air at room temperature until complete consumption of the amide (monitored by TLC). Then, transferred to a round-bottomed flask, concentrated *in vacuo*. The solutions were extracted with ethyl acetate and saturated sodium bisulfite, and the aqueous phase was extracted twice with ethyl acetate. The organic solution was dried over anhydrous sodium sulfate, filtered, and concentrated *in vacuo*, the residue was purified by flash chromatography on silica gel to obtain the products (**P11-P41**).

General procedure D for unsymmetric ureas synthesis:

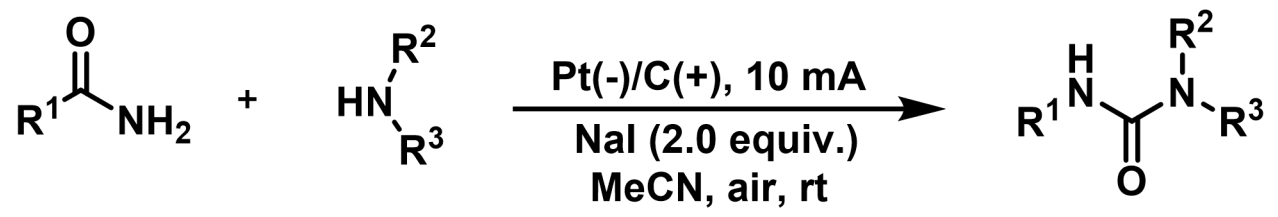

Amides, Primary or secondary amines, sodium iodide (0.6 mmol, 2.0 equiv.), and MeCN (12 mL) were added to 25 mL three-neck flask with a teflon-coated stir bar. A graphite rod was used as the anode, and a platinum plate was used as the cathode. The resulting mixture was stirred and electrolyzed with constant current 10 mA (J = 4.4 mA/cm^2) under air at room temperature until complete consumption of the amide (monitored by TLC). Then, transferred to a round-bottomed flask, concentrated *in vacuo*. The solutions were extracted with ethyl acetate and saturated sodium bisulfite, and the aqueous phase was extracted twice with ethyl acetate. The organic solution was dried over anhydrous sodium sulfate, filtered, and concentrated *in vacuo*, the residue was purified by flash chromatography on silica gel to obtain the products (**P42-P54, P60, P63-P86**).

Note: The feed ratio of amide and primary or secondary amine varies partially depending on the price of the substrate and the yield of the reaction. **Procedure D_1:** amides (0.3 mmol, 1.0 equiv.), primary or secondary amines (1.5 mmol, 5.0 equiv.); **procedure D_2:** amides (0.6 mmol, 2.0 equiv.), primary or secondary amines (0.3 mmol, 1.0 equiv.).

General procedure E for unsymmetric ureas synthesis:

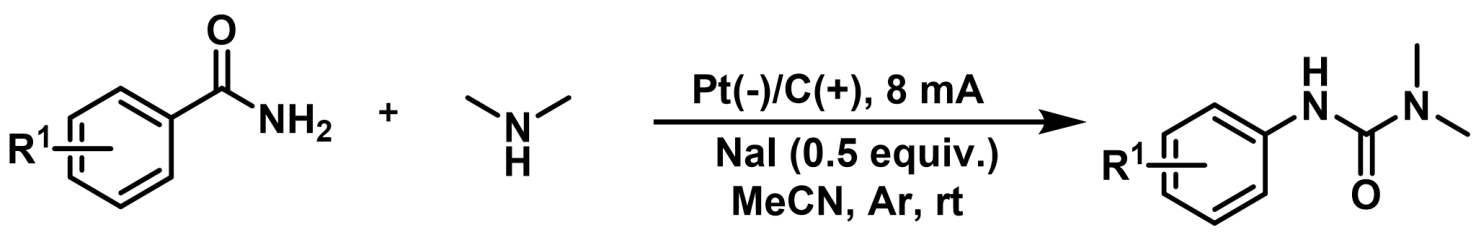

Amides (0.3 mmol, 1.0 equiv.), dimethylamine (2.0 mmol, 6.7 equiv., 2.0 M solution in THF), sodium iodide (0.15 mmol, 0.5 equiv.), and MeCN (12 mL) were added to 25 mL three-neck flask with a teflon-coated stir bar. A graphite rod was used as the anode, and a platinum plate was used as the cathode. The flask was sealed with a septum, evacuated, and backfilled under argon gas flow (this procedure was repeated three times). The resulting mixture was stirred and electrolyzed with constant current 8 mA (J = 3.6 mA/cm^2) at room temperature until complete consumption of the amide (monitored by TLC). Then, transferred to a round-bottomed flask, concentrated *in vacuo*. The solutions were extracted with ethyl acetate and saturated sodium bisulfite, and the aqueous phase was extracted twice with ethyl acetate. The organic solution was dried over anhydrous sodium sulfate, filtered, and concentrated *in vacuo*, the residue was purified by flash chromatography on silica gel to obtain the products (**P55-P59**).

General procedure F for unsymmetric ureas synthesis:

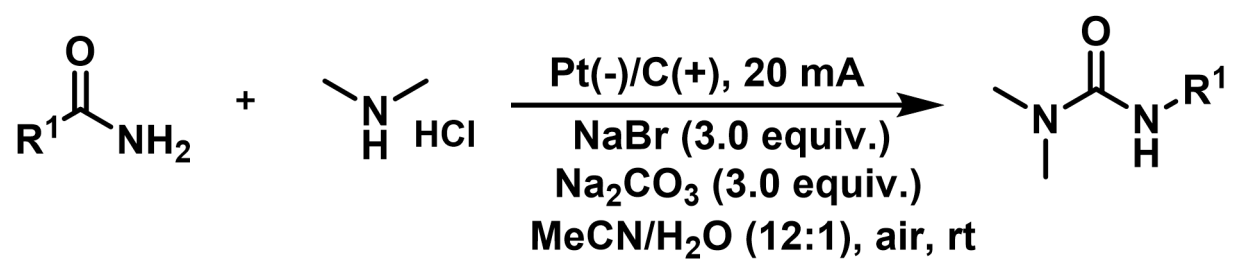

Amides (0.3 mmol, 1.0 equiv.), dimethylamine hydrochloride (1.2 mmol, 4.0 equiv.), sodium bromide (0.9 mmol, 3.0 equiv.), Na_2CO_3 (0.9 mmol, 3.0 equiv.), and MeCN/H_2O (12:1, 13 mL) were added to 25 mL three-neck flask with a teflon-coated stir bar. A graphite rod was used as the anode, and a platinum plate was used as the cathode. The resulting mixture was stirred and electrolyzed with constant current 20 mA (J = 8.9 mA/cm^2) under air at room temperature until complete consumption of the amide (monitored by TLC). Then, transferred to a round-bottomed flask, concentrated *in vacuo*. The solutions were extracted with ethyl acetate and saturated sodium bisulfite, and the aqueous phase was extracted twice with ethyl acetate. The organic solution was dried over anhydrous sodium sulfate, filtered, and concentrated *in vacuo*, the residue was purified by flash chromatography on silica gel to obtain the products (**P61, P62**).

3.2 Characterization datas of products

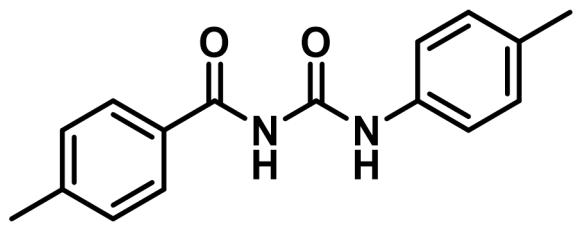

***4-methyl-N-(p-tolylcarbamoyl)benzamide* (P1)**

Compound P1 was prepared following the general procedure A (flash chromatography for purification: petroleum ether/ethyl acetate = 16:1−4:1) in 78% yield (31.4 mg) as a white solid, mp: 220.1-222.3 °C.

Reaction time: 6 h

^{1}H NMR (400 MHz, DMSO-d_6) δ 10.93 (s, 1H), 10.80 (s, 1H), 7.93 (d, J = 8.1 Hz, 2H), 7.46 (d, J = 8.2 Hz, 2H), 7.34 (d, J = 8.1 Hz, 2H), 7.16 (d, J = 8.2 Hz, 2H), 2.38 (s, 3H), 2.27 (s, 3H).

^{13}C NMR (101 MHz, DMSO-d_6) δ 168.6, 151.2, 143.5, 135.1, 132.8, 129.5, 129.4, 129.2, 128.4, 119.9, 21.1, 20.5.

The analytical data are in accordance with reported literature.[1]

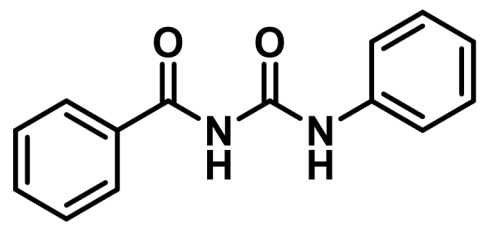

***N-(phenylcarbamoyl)benzamide* (P2)**

Compound P2 was prepared following the general procedure A (flash chromatography for purification: petroleum ether/ethyl acetate = 16:1−4:1) in 90% yield (32.4 mg) as a white solid, mp: 201.3-202.5 °C.

Reaction time: 4 h

^{1}H NMR (400 MHz, DMSO-d_6) δ 11.09 (s, 1H), 10.88 (s, 1H), 8.12 – 8.02 (m, 2H), 7.70 (t, J = 7.4 Hz, 1H), 7.65 – 7.53 (m, 4H), 7.40 (t, J = 7.9 Hz, 2H), 7.15 (t, J = 7.4 Hz, 1H).

^{13}C NMR (101 MHz, DMSO-d_6) δ 168.7, 151.1, 137.6, 133.1, 132.3, 129.0, 128.6, 128.3, 123.8, 119.9.

The analytical data are in accordance with reported literature.[1]

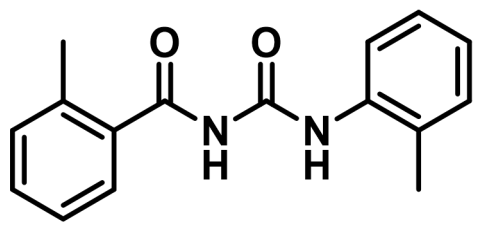

***2-methyl-N-(o-tolylcarbamoyl)benzamide* (P3)**

Compound P3 was prepared following the general procedure A (flash chromatography for purification: petroleum ether/ethyl acetate = 16:1−4:1) in 92% yield (37.0 mg) as a white solid, mp: 182.8-184.4 °C.

Reaction time: 4 h

^{1}H NMR (400 MHz, DMSO-d_6) δ 11.07 (s, 1H), 10.68 (s, 1H), 8.01 (d, J = 8.0 Hz, 1H), 7.52 (d, J = 7.4 Hz, 1H), 7.43 (t, J = 7.4 Hz, 1H), 7.26 (m, 4H), 7.05 (t, J = 7.5 Hz, 1H), 2.42 (s, 3H), 2.31 (s, 3H).

^{13}C NMR (101 MHz, DMSO-d_6) δ 171.8, 150.9, 136.1, 135.9, 134.3, 130.9, 130.8, 130.4, 127.9, 127.4, 126.5, 125.7, 123.9, 120.8, 19.5, 17.7.

HRMS（ESI, Q-TOF）m/z: $[M+Na]^+$ Calcd for $NaC_{16}H_{16}O_2N_2$, 291.1104; Found: 291.1111.

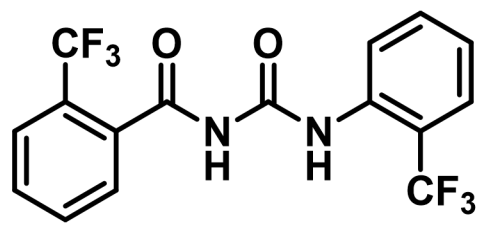

***2-(trifluoromethyl)-N-((2-(trifluoromethyl)phenyl)carbamoyl)benzamide* (P4)**

Compound P4 was prepared following the general procedure A (flash chromatography for purification: petroleum ether/ethyl acetate = 16:1−4:1) in 85% yield (48.0 mg) as a white solid, mp: 129.7-130.6 ℃.

Reaction time: 2.5 h

^{1}H NMR (400 MHz, DMSO-d_6) δ 11.66 (s, 1H), 10.86 (s, 1H), 8.16 (d, J = 8.2 Hz, 1H), 7.86 (d, J = 7.6 Hz, 1H), 7.83 – 7.65 (m, 5H), 7.36 (t, J = 7.7 Hz, 1H).

^{13}C NMR (101 MHz, DMSO-d_6) δ 170.2, 150.9, 134.7 (d, J = 1.7 Hz), 133.43 (d, J = 1.9 Hz), 133.35, 132.6, 131.1, 128.7, 126.5 (d, J = 4.6 Hz), 126.2 (d, J = 4.6 Hz), 126.0 (d, J = 31.8 Hz), 125.1, 125.0, 123.9 (d, J = 272.9 Hz), 123.7 (d, J = 273.4 Hz), 120.3 (d, J = 29.5 Hz).

^{19}F NMR (376 MHz, DMSO-d_6) δ -57.6 (s, 3F), -60.2 (s, 3F).

HRMS（ESI, Q-TOF）m/z: $[M+Na]^+$ Calcd for $NaC_{16}H_{10}O_2F_6N_2$, 399.0539; Found: 399.0562.

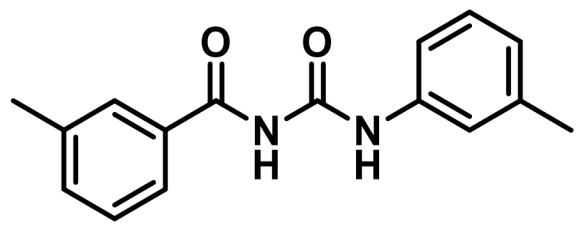

***3-methyl-N-(m-tolylcarbamoyl)benzamide* (P5)**

Compound P5 was prepared following the general procedure A (flash chromatography for purification: petroleum ether/ethyl acetate = 16:1−4:1) in 84% yield (33.8 mg) as a white solid, mp: 155.4-157.0 ℃.

Reaction time: 6 h

^{1}H NMR (400 MHz, $CDCl_3$) δ 11.03 (s, 1H), 10.25 (s, 1H), 7.90 (d, J = 7.4 Hz, 1H), 7.85 (s, 1H), 7.47 – 7.31 (m, 4H), 7.28 – 7.18 (m, 1H), 6.95 (d, J = 7.5 Hz, 1H), 2.39 (s, 3H), 2.34 (s, 3H).

^{13}C NMR (101 MHz, $CDCl_3$) δ 169.2, 152.5, 138.9, 138.8, 137.3, 134.1, 132.3, 128.9, 128.7, 125.31, 125.25, 121.1, 117.7, 21.6, 21.4.

HRMS（ESI, Q-TOF）m/z: $[M+Na]^+$ Calcd for $NaC_{16}H_{16}O_2N_2$, 291.1104; Found: 291.1108.

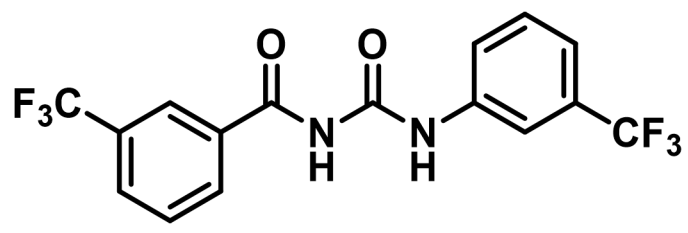

***3-(trifluoromethyl)-N-((3-(trifluoromethyl)phenyl)carbamoyl)benzamide* (P6)**

Compound P6 was prepared following the general procedure A (flash chromatography for purification: petroleum ether/ethyl acetate = 16:1−4:1) in 80% yield (45.2 mg) as a white solid, mp: 179.7-181.5 ℃.

Reaction time: 4 h

^{1}H NMR (400 MHz, DMSO-d_6) δ 11.57 (s, 1H), 11.20 (s, 1H), 8.39 (s, 1H), 8.31 (d, J = 7.9 Hz, 1H), 8.19 (d, J = 8.2 Hz, 1H), 7.99 (d, J = 7.8 Hz, 1H), 7.81 – 7.64 (m, 3H), 7.34 (t, J = 7.6 Hz, 1H).

^{13}C NMR (101 MHz, DMSO-d_6) δ 168.0, 151.4, 134.78, 134.75 (d, J = 1.8 Hz), 133.3, 132.9, 132.6, 129.9, 129.6 (q, J = 3.5 Hz), 129.4 (d, J = 32.4 Hz), 126.1 (q, J = 5.6 Hz), 125.3 (d, J = 3.8 Hz), 124.7, 123.9 (q, J = 272.9 Hz), 123.8 (q, J = 272.4 Hz), 119.8 (q, J = 29.4 Hz).

^{19}F NMR (376 MHz, DMSO-*d_6*) δ -60.0 (s, 3F), -61.0 (s, 3F).
HRMS（ESI, Q-TOF）m/z: $[M+H]^+$ Calcd for $C_{16}H_{11}O_2F_6N_2$, 377.0719; Found: 377.0725.

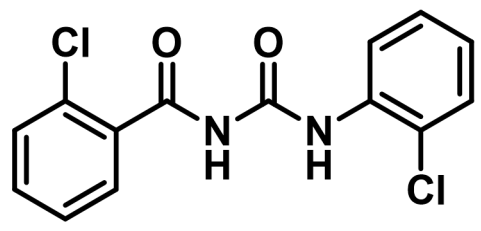

2-chloro-N-((2-chlorophenyl)carbamoyl)benzamide **(P7)**
Compound P7 was prepared following the general procedure A (flash chromatography for purification: petroleum ether/ethyl acetate = 16:1−4:1) in 85% yield (39.4 mg) as a white solid, mp: 205.1-207.4 °C.
Reaction time: 5 h
^{1}H NMR (400 MHz, DMSO-*d_6*) δ 11.51 (s, 1H), 11.04 (s, 1H), 8.30 (d, *J* = 8.2 Hz, 1H), 7.66 (d, *J* = 7.7 Hz, 1H), 7.62 – 7.51 (m, 3H), 7.49 – 7.42 (m, 1H), 7.38 (t, *J* = 7.8 Hz, 1H), 7.16 (t, *J* = 7.0 Hz, 1H).
^{13}C NMR (101 MHz, DMSO-*d_6*) δ 169.0, 150.4, 134.6, 134.4, 132.2, 129.9, 129.8, 129.4, 129.3, 128.0, 127.3, 124.9, 122.4, 121.4.
HRMS（ESI, Q-TOF）m/z: $[M+Na]^+$ Calcd for $NaC_{14}H_{10}O_2Cl_2N_2$, 331.0012; Found: 331.0017.

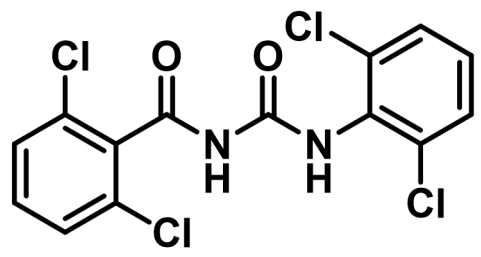

2,6-dichloro-N-((2,6-dichlorophenyl)carbamoyl)benzamide **(P8)**
Compound P8 was prepared following the general procedure A (flash chromatography for purification: petroleum ether/ethyl acetate = 16:1−4:1) in 65% yield (36.9 mg) as a white solid, mp: 205.3-207.6 °C.
Reaction time: 6 h
^{1}H NMR (400 MHz, DMSO-*d_6*) δ 11.65 (s, 1H), 9.97 (s, 1H), 7.66 – 7.46 (m, 5H), 7.42 – 7.35 (m, 1H).
^{13}C NMR (101 MHz,DMSO-*d_6*) δ 165.9, 150.6, 134.6, 133.8, 132.2, 130.7, 129.5, 128.5, 128.3.
HRMS（ESI, Q-TOF）m/z: $[M+Na]^+$ Calcd for $NaC_{14}H_8O_2Cl_4N_2$, 398.9232; Found: 398.9238.

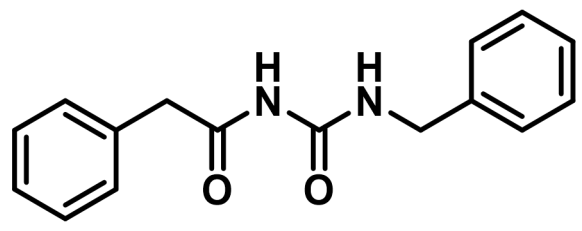

N-(benzylcarbamoyl)-2-phenylacetamide **(P9)**
Compound P9 was prepared following the general procedure B (flash chromatography for purification: petroleum ether/ethyl acetate = 10:1−2:1) in 65% yield (26.2 mg) as a white solid, mp: 184.6-186.2 °C.
Reaction time: 10 h
^{1}H NMR (400 MHz, DMSO-*d_6*) δ 10.64 (s, 1H), 8.69 (s, 1H), 7.42 – 7.10 (m, 10H), 4.34 (d, *J* = 6.0 Hz, 2H), 3.62 (s, 2H).
^{13}C NMR (101 MHz, DMSO-*d_6*) δ 172.9, 153.4, 139.2, 134.7, 129.3, 128.43, 128.41, 127.2, 127.0, 126.9, 42.6, 42.5.

The analytical data are in accordance with reported literature.[2]

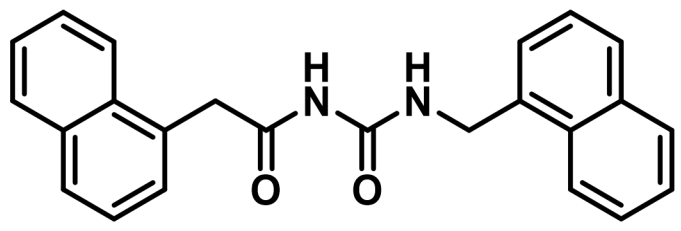

2-(naphthalen-1-yl)-N-((naphthalen-1-ylmethyl)carbamoyl)acetamide **(P10)**

Compound P10 was prepared following the general procedure B (flash chromatography for purification: petroleum ether/ethyl acetate = 16:1−4:1) in 50% yield (27.7 mg) as a white solid, mp: 152.1-153.9 °C.

Reaction time: 8 h

^{1}H NMR (400 MHz, $CDCl_3$) δ 9.59 (s, 1H), 8.88 (t, J = 4.8 Hz, 1H), 8.04 – 7.97 (m, 2H), 7.90 – 7.77 (m, 4H), 7.54 – 7.48 (m, 4H), 7.46 – 7.37 (m, 4H), 4.94 (d, J = 5.6 Hz, 2H), 4.12 (s, 2H).

^{13}C NMR (101 MHz, $CDCl_3$) δ 173.0, 154.3, 134.1, 133.9, 133.1, 132.1, 131.3, 129.6, 129.0, 128.91, 128.87, 128.7, 128.6, 126.9, 126.7, 126.2, 126.0, 125.7, 125.5, 123.7, 123.3, 41.8, 41.7.

HRMS（ESI, Q-TOF）m/z: [M+Na]$^+$ Calcd for $NaC_{24}H_{20}O_2N_2$, 391.1417; Found: 391.1423.

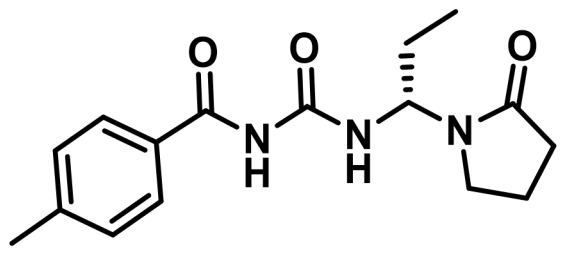

(S)-4-methyl-N-((1-(2-oxopyrrolidin-1-yl)propyl)carbamoyl)benzamide **(P11)**

Compound P11 was prepared following the general procedure C (flash chromatography for purification: petroleum ether/ethyl acetate = 1:1−1:5) in 61% yield (55.5 mg) as a white solid, mp: 128.5-130.2 °C.

Reaction time: 8 h

^{1}H NMR (400 MHz, $CDCl_3$) δ 9.60 (s, 1H), 9.30 (d, J = 7.5 Hz, 1H), 7.90 (d, J = 8.2 Hz, 2H), 7.37 (d, J = 8.0 Hz, 2H), 5.81 (q, J = 7.6 Hz, 1H), 3.39 (t, J = 7.0 Hz, 2H), 2.47 – 2.36 (m, 5H), 2.09 – 1.96 (m, 2H), 1.87 – 1.76 (m, 2H), 0.98 (t, J = 7.4 Hz, 3H).

^{13}C NMR (101 MHz, $CDCl_3$) δ 174.9, 168.5, 162.2, 144.4, 129.9, 129.1, 128.1, 59.9, 42.8, 31.5, 25.9, 21.8, 18.3, 10.0.

HRMS（ESI, Q-TOF）m/z: [M+Na]$^+$ Calcd for $NaC_{16}H_{21}O_3N_3$, 326.1475; Found: 326.1480.

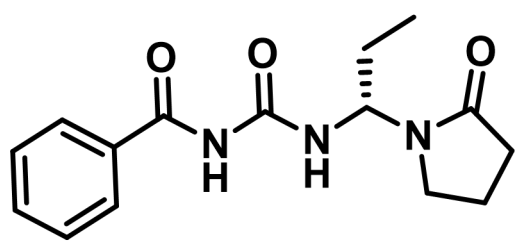

(S)-N-((1-(2-oxopyrrolidin-1-yl)propyl)carbamoyl)benzamide **(P12)**

Compound P12 was prepared following the general procedure C (R^2CONH_2, 0.75 mmol, flash chromatography for purification: petroleum ether/ethyl acetate = 1:1−1:5) in 51% yield (44.3 mg) as a white solid, mp: 126.1-127.9 °C.

Reaction time: 8 h

^{1}H NMR (400 MHz, $CDCl_3$) δ 10.39 (s, 1H), 9.37 (d, J = 7.5 Hz, 1H), 8.14 – 8.05 (m, 2H), 7.67 – 7.54 (m, 3H), 5.91 – 5.79 (m, 1H), 3.43 – 3.31 (m, 2H), 2.39 (t, J = 8.0 Hz, 2H), 2.08 – 1.93 (m, 2H), 1.90 – 1.73 (m, 2H), 0.96 (t, J = 7.4 Hz, 3H).

^{13}C NMR (101 MHz, $CDCl_3$) δ 174.8, 168.9, 154.3, 133.5, 131.8, 129.2, 128.2, 59.8, 42.6, 31.4, 25.9, 18.2, 9.9.
HRMS（ESI, Q-TOF）m/z: $[M+Na]^+$ Calcd for $NaC_{15}H_{19}O_3N_3$, 312.1319; Found: 312.1321.

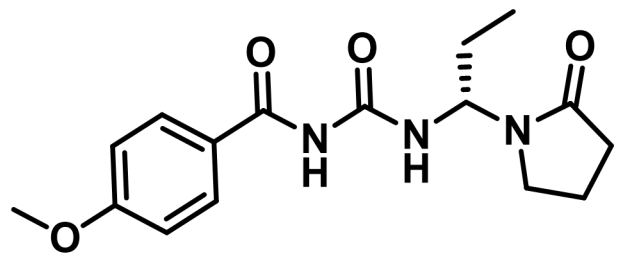

(S)-4-methoxy-N-((1-(2-oxopyrrolidin-1-yl)propyl)carbamoyl)benzamide **(P13)**
Compound P13 was prepared following the general procedure C (R^2CONH_2, 0.75 mmol, flash chromatography for purification: petroleum ether/ethyl acetate = 1:1−1:5) in 53% yield (50.75 mg) as a white solid, mp: 122.4-123.9 ℃.
Reaction time: 8 h
^{1}H NMR (400 MHz, $CDCl_3$) δ 10.16 (s, 1H), 9.40 (d, J = 7.5 Hz, 1H), 8.06 (d, J = 8.5 Hz, 2H), 7.10 (d, J = 8.5 Hz, 2H), 5.92 – 5.81 (m, 1H), 3.89 (s, 3H), 3.43 – 3.33 (m, 2H), 2.38 (t, J = 8.0 Hz, 2H), 2.02 – 1.97 (m, 2H), 1.86 – 1.72 (m, 2H), 0.97 (t, J = 7.3 Hz, 3H).
^{13}C NMR (101 MHz, $CDCl_3$) δ 174.8, 168.3, 163.9, 154.5, 130.4, 124.0, 114.5, 59.8, 55.7, 42.5, 31.5, 25.9, 18.2, 10.0.
HRMS（ESI, Q-TOF）m/z: $[M+Na]^+$ Calcd for $NaC_{16}H_{21}O_4N_3$, 342.1424; Found: 342.1427.

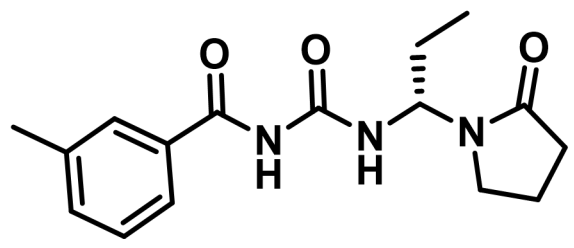

(S)-3-methyl-N-((1-(2-oxopyrrolidin-1-yl)propyl)carbamoyl)benzamide **(P14)**
Compound P14 was prepared following the general procedure C (flash chromatography for purification: petroleum ether/ethyl acetate = 1:1−1:5) in 54% yield (49.2 mg) as a white solid, mp: 80.0-82.1 ℃.
Reaction time: 8 h
^{1}H NMR (400 MHz, $CDCl_3$) δ 9.95 (s, 1H), 9.34 (d, J = 7.8 Hz, 1H), 7.86 (d, J = 7.8 Hz, 1H), 7.80 (s, 1H), 7.50 (t, J = 7.7 Hz, 1H), 7.40 (d, J = 7.6 Hz, 1H), 5.81 (q, J = 7.6 Hz, 1H), 3.38 (t, J = 7.0 Hz, 2H), 2.46 – 2.35 (m, 5H), 2.07 – 1.94 (m, 2H), 1.91 – 1.74 (m, 2H), 0.96 (t, J = 7.4 Hz, 3H).
^{13}C NMR (101 MHz, $CDCl_3$) δ 174.8, 169.0, 154.0, 138.8, 134.2, 131.9, 129.2, 128.7, 125.1, 59.9, 42.9, 31.4, 25.9, 21.5, 18.2, 10.0.
HRMS（ESI, Q-TOF）m/z: $[M+Na]^+$ Calcd for $NaC_{16}H_{21}O_3N_3$, 326.1475; Found: 326.1484.

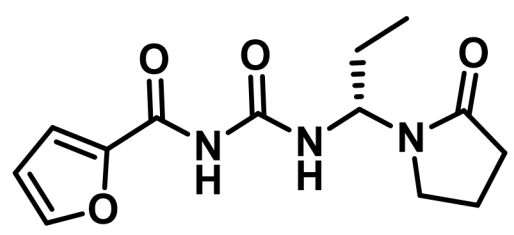

(S)-N-((1-(2-oxopyrrolidin-1-yl)propyl)carbamoyl)furan-2-carboxamide **(P15)**
Compound P15 was prepared following the general procedure C (flash chromatography for purification: petroleum ether/ethyl acetate = 1:1−1:5) in 60% yield (50.3 mg) as a white solid, mp: 126.3-129.3 ℃.
Reaction time: 8 h

^{1}H NMR (400 MHz, $CDCl_3$) δ 10.10 (s, 1H), 9.02 (d, *J* = 7.5 Hz, 1H), 7.73 (d, *J* = 1.0 Hz, 1H), 7.55 (d, *J* = 3.5 Hz, 1H), 6.65 – 6.55 (m, 1H), 5.83 (q, *J* = 7.6 Hz, 1H), 3.34 (t, *J* = 7.0 Hz, 2H), 2.41 – 2.33 (m, 2H), 2.05 – 1.93 (m, 2H), 1.86 – 1.69 (m, 2H), 0.95 (t, *J* = 7.4 Hz, 3H).
^{13}C NMR (101 MHz, $CDCl_3$) δ 174.7, 159.1, 153.7, 147.2, 145.4, 118.5, 113.1, 59.6, 42.3, 31.4, 25.8, 18.2, 9.9.
HRMS（ESI, Q-TOF）m/z: $[M+Na]^+$ Calcd for $NaC_{13}H_{17}O_4N_3$, 302.1111; Found: 302.1120.

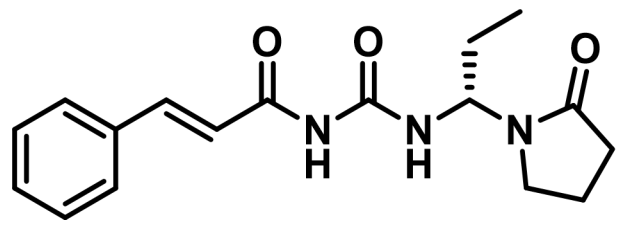

(S)-N-((1-(2-oxopyrrolidin-1-yl)propyl)carbamoyl)cinnamamide **(P16)**
Compound P16 was prepared following the general procedure C (R^2CONH_2, 0.75 mmol, flash chromatography for purification: petroleum ether/ethyl acetate = 1:1−1:5) in 64% yield (60.6 mg) as a yellow oil.
Reaction time: 8 h
^{1}H NMR (400 MHz, $CDCl_3$) δ 10.70 (s, 1H), 9.19 (d, *J* = 6.4 Hz, 1H), 7.76 (d, *J* = 15.6 Hz, 1H), 7.70 (d, *J* = 6.8 Hz, 2H), 7.52 – 7.32 (m, 3H), 6.69 (d, *J* = 15.6 Hz, 1H), 5.84 (dd, *J* = 13.5, 6.4 Hz, 1H), 3.36 (t, *J* = 6.2 Hz, 2H), 2.36 (d, *J* = 5.7 Hz, 2H), 2.11 – 1.92 (m, 2H), 1.90 – 1.69 (m, 2H), 0.99 (t, *J* = 6.8 Hz, 3H).
^{13}C NMR (101 MHz, $CDCl_3$) δ 174.9, 167.7, 155.0, 145.5, 134.1, 130.8, 129.1, 129.0, 119.1, 59.8, 42.4, 31.4, 25.8, 18.2, 9.9.
HRMS（ESI, Q-TOF）m/z: $[M+Na]^+$ Calcd for $C_{17}H_{21}N_3NaO_3$, 338.1475; Found: 338.1485.

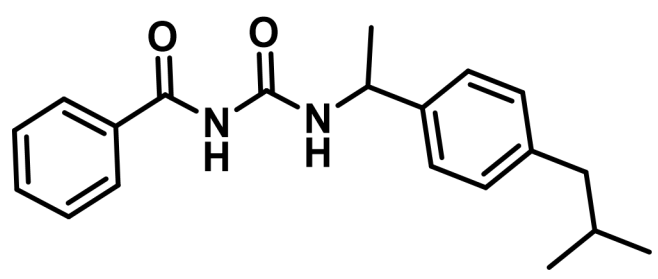

N-((1-(4-isobutylphenyl)ethyl)carbamoyl)benzamide **(P17)**
Compound P17 was prepared following the general procedure C (flash chromatography for purification: petroleum ether/ethyl acetate = 15:1−3:1) in 55% yield (53.5 mg) as a white solid, mp: 144.5-146.2 ℃.
Reaction time: 8 h
^{1}H NMR (400 MHz, $CDCl_3$) δ 10.26 (s, 1H), 9.26 (d, *J* = 7.7 Hz, 1H), 8.01 (d, *J* = 7.5 Hz, 2H), 7.57 (t, *J* = 7.4 Hz, 1H), 7.43 (t, *J* = 7.8 Hz, 2H), 7.29 (d, *J* = 8.0 Hz, 2H), 7.13 (d, *J* = 8.0 Hz, 2H), 5.17 – 5.03 (m, 1H), 2.46 (d, *J* = 7.2 Hz, 2H), 1.93 – 1.79 (m, 1H), 1.58 (d, *J* = 6.9 Hz, 3H), 0.90 (d, *J* = 6.6 Hz, 6H).
^{13}C NMR (101 MHz, $CDCl_3$) δ 168.7, 154.1, 140.8, 140.7, 133.0, 132.5, 129.5, 128.7, 128.2, 125.9, 49.7, 45.2, 30.3, 22.9, 22.5.
HRMS（ESI, Q-TOF）m/z: $[M+Na]^+$ Calcd for $NaC_{20}H_{24}O_2N_2$, 347.1730; Found: 347.1734.

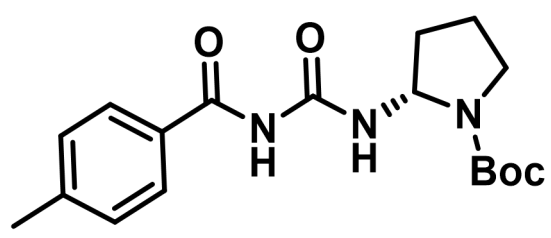

tert-butyl (S)-2-(3-(4-methylbenzoyl)ureido)pyrrolidine-1-carboxylate **(P18)**

Compound P18 was prepared following the general procedure C (flash chromatography for purification: petroleum ether/ethyl acetate = 5:1−1:1) in 70% yield (73.0 mg) as a white solid, mp: 178.7-179.3 ℃.

Reaction time: 8 h

^{1}H NMR (400 MHz, $CDCl_3$) δ 9.69 (s, 1H), 8.97 (d, J = 18.2 Hz, 1H), 7.89 (d, J = 6.3 Hz, 2H), 7.28 (d, J = 7.8 Hz, 2H), 5.74 (d, J = 44.1 Hz, 1H), 3.59 – 3.23 (m, 2H), 2.41 (s, 3H), 2.14 – 1.83 (m, 4H), 1.39 (s, 9H).

^{13}C NMR (101 MHz, $CDCl_3$) δ 168.2, 154.1, 153.3, 144.0, 129.7, 129.6, 128.1, 80.3, 64.6, 46.1, 34.1, 28.5, 22.4, 21.7.

HRMS（ESI, Q-TOF）m/z: $[M+Na]^+$ Calcd for $NaC_{18}H_{25}O_4N_3$, 370.1737; Found: 370.1745.

HPLC (>99% ee, AD-H, 4.6 mm × 250 mm, particle size 5 μm, hexane/isopropanol = 85/15, flow 1.0 mL/min, detection at 210 nm) retention time = 14.34 min (minor) and 18.64 min (major).

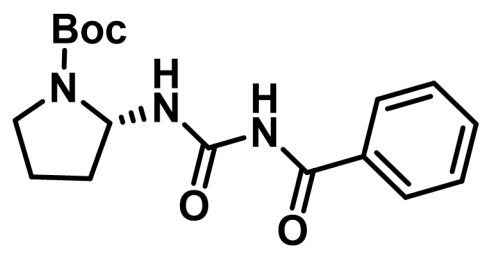

tert-butyl (S)-2-(3-benzoylureido)pyrrolidine-1-carboxylate **(P19)**

Compound P19 was prepared following the general procedure C (flash chromatography for purification: petroleum ether/ethyl acetate = 5:1−1:1) in 55% yield (55.0 mg) as a white solid, mp: 88.6-90.9 ℃.

Reaction time: 8 h

^{1}H NMR (400 MHz, $CDCl_3$) δ 9.89 (s, 1H), 8.96 (d, J = 20.3 Hz, 1H), 8.00 (s, 2H), 7.58 (t, J = 7.1 Hz, 1H), 7.48 (t, J = 7.4 Hz, 2H), 5.87 – 5.45 (m, 1H), 3.61 – 3.25 (m, 2H), 2.13 – 1.92 (m, 4H), 1.46 – 1.36 (m, 9H).

^{13}C NMR (101 MHz, $CDCl_3$) δ 168.4, 154.1, 153.3, 133.2, 132.4, 128.8, 128.1, 80.3, 64.6, 46.1, 34.0, 28.5, 22.5.

HRMS（ESI, Q-TOF）m/z: $[M+Na]^+$ Calcd for $NaC_{17}H_{23}O_4N_3$, 356.1581; Found: 356.1590.

HPLC (>99% ee, AD-H, 4.6 mm × 250 mm, particle size 5 μm, hexane/isopropanol = 85/15, flow 1.0 mL/min, detection at 210 nm) retention time = 11.48 min (minor) and 14.97 min (major).

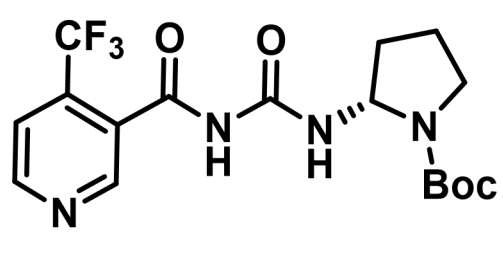

tert-butyl (S)-2-(3-(4-(trifluoromethyl)nicotinoyl)ureido)pyrrolidine-1-carboxylate **(P20)**

Compound P20 was prepared following the general procedure C (flash chromatography for purification: petroleum ether/ethyl acetate = 5:1−1:1) in 67% yield (80.9 mg) as a white solid, mp: 139.5-141.4 ℃.

Reaction time: 8 h

^{1}H NMR (400 MHz, $CDCl_3$) δ 10.77 (s, 1H), 8.95 – 8.80 (m, 2H), 8.50 (d, J = 5.1 Hz, 1H), 7.62 (d, J = 5.1 Hz, 1H), 5.41 (d, J = 61.3 Hz, 1H), 3.51 – 3.11 (m, 2H), 2.01 – 1.72 (m, 4H), 1.34 (s, 9H).

^{13}C NMR (101 MHz, $CDCl_3$) δ 166.7, 153.8, 152.8, 152.6, 149.2, 135.9 (q, J = 34.5 Hz), 128.0, 122.1 (q, J = 274.8 Hz), 120.2, 80.3, 64.6, 46.0, 33.5, 28.3, 22.4.

^{19}F NMR (377 MHz, $CDCl_3$) δ -61.3 (s, 3F).
HRMS（ESI, Q-TOF）m/z: $[M+Na]^+$ Calcd for $NaC_{17}H_{21}O_4F_3N_4$, 425.1407; Found: 425.1414.
HPLC (>99% ee, AD-H, 4.6 mm × 250 mm, particle size 5 μm, hexane/isopropanol = 85/15, flow 1.0 mL/min, detection at 210 nm) retention time = 11.09 min (minor) and 16.65 min (major).

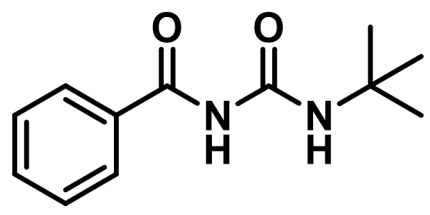

N-(tert-butylcarbamoyl)benzamide **(P21)**
Compound P21 was prepared following the general procedure C (R^2CONH_2, 0.75 mmol, flash chromatography for purification: petroleum ether/ethyl acetate = 15:1−3:1) in 65% yield (43.0 mg) as a white solid, mp: 112.5-114.1 °C.
Reaction time: 6 h
^{1}H NMR (400 MHz, $CDCl_3$) δ 10.33 (s, 1H), 8.94 (s, 1H), 8.09 (d, *J* = 7.5 Hz, 2H), 7.57 (t, *J* = 7.4 Hz, 1H), 7.46 (t, *J* = 7.6 Hz, 2H), 1.44 (s, 9H).
^{13}C NMR (101 MHz, $CDCl_3$) δ 168.8, 153.8, 132.9, 132.8, 128.7, 128.2, 51.0, 29.0.
The analytical data are in accordance with reported literature.[3]

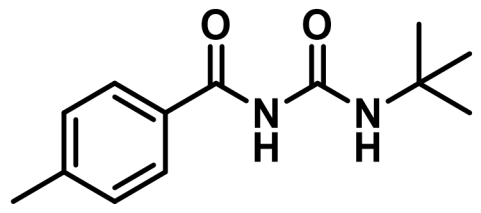

N-(tert-butylcarbamoyl)-4-methylbenzamide **(P22)**
Compound P22 was prepared following the general procedure C (flash chromatography for purification: petroleum ether/ethyl acetate = 15:1−3:1) in 55% yield (38.7 mg) as a white solid, mp: 175.9-187.2 °C.
Reaction time: 6 h
^{1}H NMR (400 MHz, $CDCl_3$) δ 9.90 (s, 1H), 8.91 (s, 1H), 7.95 (d, *J* = 8.2 Hz, 2H), 7.25 (d, *J* = 5.7 Hz, 2H), 2.41 (s, 3H), 1.44 (s, 9H).
^{13}C NMR (101 MHz, $CDCl_3$) δ 168.6, 153.6, 143.7, 129.9, 129.5, 128.1, 51.0, 29.0, 21.7.
HRMS（ESI, Q-TOF）m/z: $[M+Na]^+$ Calcd for $C_{13}H_{18}N_2NaO_2$, 257.1260; Found: 257.1269.

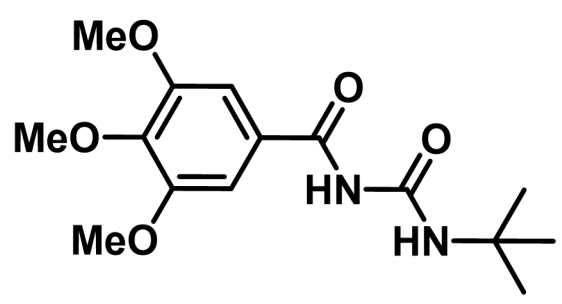

N-(tert-butylcarbamoyl)-3,4,5-trimethoxybenzamide **(P23)**
Compound P23 was prepared following the general procedure C (flash chromatography for purification: petroleum ether/ethyl acetate = 10:1−3:1) in 42% yield (39.1 mg) as a white solid, mp: 53.6-55.5 °C.
Reaction time: 6 h
^{1}H NMR (400 MHz, $CDCl_3$) δ 9.09 (s, 1H), 8.71 (s, 1H), 7.09 (s, 2H), 3.91 (s, 6H), 3.89 (s, 3H), 1.35 (s, 9H).
^{13}C NMR (101 MHz, $CDCl_3$) δ 168.2, 153.4, 152.8, 142.2, 128.0, 105.2, 61.1, 56.5, 51.0, 28.9.
HRMS（ESI, Q-TOF）m/z: $[M+Na]^+$ Calcd for $NaC_{15}H_{22}O_5N_2$, 333.1421; Found: 333.1427.

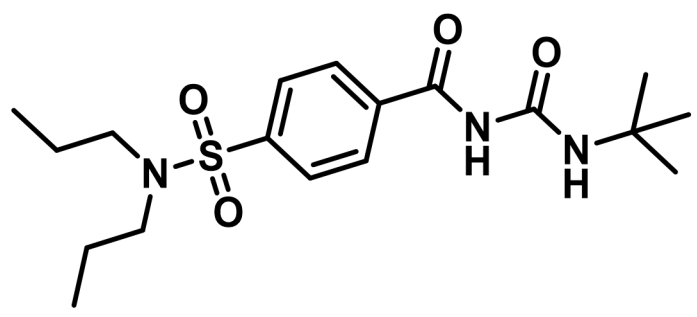

N-(tert-butylcarbamoyl)-4-(N,N-dipropylsulfamoyl)benzamide **(P24)**

Compound P24 was prepared following the general procedure C (flash chromatography for purification: petroleum ether/ethyl acetate = 15:1−3:1) in 65% yield (74.8 mg) as a white solid, mp: 175.1-177.9 ℃.

Reaction time: 6 h

^{1}H NMR (400 MHz, $CDCl_3$) δ 10.81 (s, 1H), 8.88 (s, 1H), 8.21 (d, *J* = 8.6 Hz, 2H), 7.87 (d, *J* = 8.6 Hz, 2H), 3.12 – 3.05 (m, 4H), 1.58 – 1.49 (m, 4H), 1.42 (s, 9H), 0.86 (t, *J* = 7.4 Hz, 6H).

^{13}C NMR (101 MHz, $CDCl_3$) δ 167.6, 153.7, 144.1, 136.2, 129.1, 127.2, 51.2, 50.1, 29.0, 22.1, 11.3.

HRMS（ESI, Q-TOF）m/z: $[M+H]^+$ Calcd for $C_{18}H_{30}O_4N_3S$, 384.1952; Found: 384.1960.

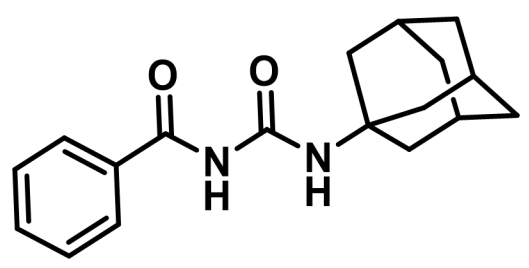

N-(((3s,5s,7s)-adamantan-1-yl)carbamoyl)benzamide **(P25)**

Compound P25 was prepared following the general procedure C (R^2CONH_2, 0.75 mmol, flash chromatography for purification: petroleum ether/ethyl acetate = 15:1−3:1) in 47% yield (42.1 mg) as a white solid, mp: 212.8-215.3 ℃.

Reaction time: 6 h

^{1}H NMR (400 MHz, $CDCl_3$) δ 10.21 (s, 1H), 8.85 (s, 1H), 8.05 (d, *J* = 7.5 Hz, 2H), 7.56 (t, *J* = 7.4 Hz, 1H), 7.45 (t, *J* = 7.6 Hz, 2H), 2.19 – 2.01 (m, 9H), 1.76 – 1.67 (m, 6H).

^{13}C NMR (101 MHz, $CDCl_3$) δ 168.9, 153.3, 132.9, 132.8, 128.6, 128.2, 51.6, 41.8, 36.5, 29.6.

The analytical data are in accordance with reported literature.[4]

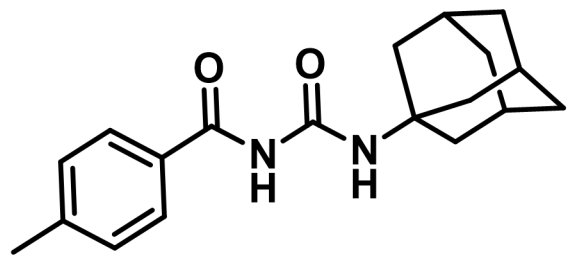

N-(((3s,5s,7s)-adamantan-1-yl)carbamoyl)-4-methylbenzamide **(P26)**

Compound P26 was prepared following the general procedure C (flash chromatography for purification: petroleum ether/ethyl acetate = 15:1−3:1) in 52% yield (48.7 mg) as a white solid, mp: 226.3-228.6 ℃.

Reaction time: 6 h

^{1}H NMR (400 MHz, $CDCl_3$) δ 9.62 (s, 1H), 8.77 (s, 1H), 7.87 (d, *J* = 7.4 Hz, 2H), 7.25 (d, *J* = 6.6 Hz, 2H), 2.39 (s, 3H), 2.07 (s, 9H), 1.70 (s, 6H).

^{13}C NMR (101 MHz, $CDCl_3$) δ 168.6, 152.9, 143.6, 130.1, 129.5, 128.1, 51.6, 41.8, 36.5, 29.6, 21.7.

HRMS（ESI, Q-TOF）m/z: $[M+Na]^+$ Calcd for $NaC_{19}H_{24}O_2N_2$, 335.1730; Found: 335.1732.

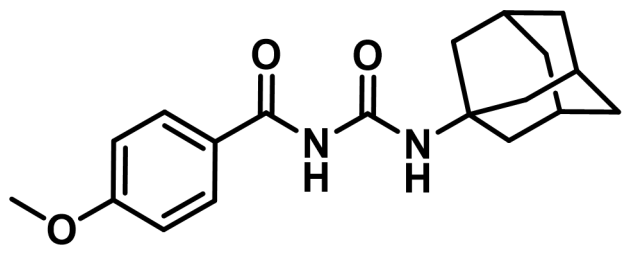

N-(((3s,5s,7s)-adamantan-1-yl)carbamoyl)-4-methoxybenzamide **(P27)**

Compound P27 was prepared following the general procedure C (flash chromatography for purification: petroleum ether/ethyl acetate = 15:1−3:1) in 53% yield (52.2 mg) as a white solid, mp: 218.4-219.6 °C.

Reaction time: 6 h

^{1}H NMR (400 MHz, $CDCl_3$) δ 10.22 (s, 1H), 8.93 (s, 1H), 8.07 (d, J = 8.9 Hz, 2H), 6.92 (d, J = 8.9 Hz, 2H), 3.84 (s, 3H), 2.10 (s, 9H), 1.72 (s, 6H).

^{13}C NMR (101 MHz, $CDCl_3$) δ 168.2, 163.3, 153.5, 130.3, 125.1, 113.9, 55.5, 51.5, 41.8, 36.5, 29.6.

HRMS（ESI, Q-TOF）m/z: $[M+Na]^+$ Calcd for $NaC_{19}H_{24}O_3N_2$, 351.1679; Found: 351.1692.

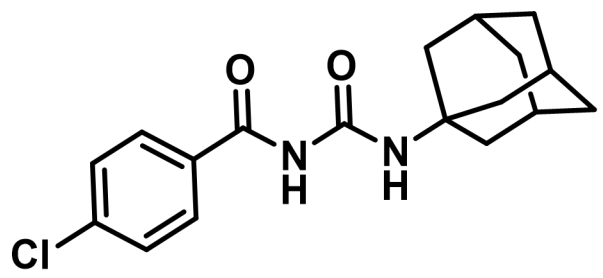

N-(((3s,5s,7s)-adamantan-1-yl)carbamoyl)-4-chlorobenzamide **(P28)**

Compound P28 was prepared following the general procedure C (flash chromatography for purification: petroleum ether/ethyl acetate = 15:1−3:1) in 45% yield (44.9 mg) as a white solid, mp: 227.2-229.1 °C.

Reaction time: 6 h

^{1}H NMR (400 MHz, $CDCl_3$) δ 10.17 (s, 1H), 8.76 (s, 1H), 7.98 (d, J = 8.2 Hz, 2H), 7.42 (d, J = 8.3 Hz, 2H), 2.09 (d, J = 23.9 Hz, 9H), 1.71 (s, 6H).

^{13}C NMR (101 MHz, $CDCl_3$) δ 167.9, 153.1, 139.3, 131.4, 129.7, 129.0, 51.8, 41.8, 36.5, 29.6.

HRMS（ESI, Q-TOF）m/z: $[M+Na]^+$ Calcd for $NaC_{18}H_{21}O_2ClN_2$, 355.1184; Found: 355.1189.

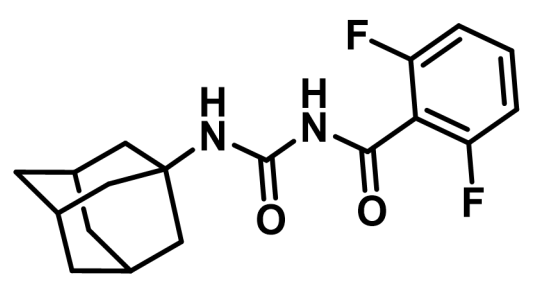

N-(((3s,5s,7s)-adamantan-1-yl)carbamoyl)-2,6-difluorobenzamide **(P29)**

Compound P29 was prepared following the general procedure C (flash chromatography for purification: petroleum ether/ethyl acetate = 15:1−4:1) in 43% yield (43.1 mg) as a white solid, mp: 236.1-237.9 °C.

Reaction time: 6 h

^{1}H NMR (400 MHz, $CDCl_3$) δ 10.28 (s, 1H), 8.24 (s, 1H), 7.47 – 7.35 (m, 1H), 6.96 (t, J = 8.2 Hz, 2H), 2.02 (s, 3H), 1.89 (s, 6H), 1.67 – 1.56 (m, 6H).

^{13}C NMR (101 MHz, $CDCl_3$) δ 162.3, 160.0 (dd, J = 254.4, 6.5 Hz), 152.4, 132.5 (t, J = 10.4 Hz), 113.6 (t, J = 19.5 Hz), 112.1 (d, J = 25.0 Hz), 51.8, 41.4, 36.4, 29.5.

^{19}F NMR (377 MHz, $CDCl_3$) δ -111.4 (s, 2F).

HRMS（ESI, Q-TOF）m/z: $[M+Na]^+$ Calcd for $NaC_{18}H_{20}O_2F_2N_2$, 357.1385; Found: 357.1393.

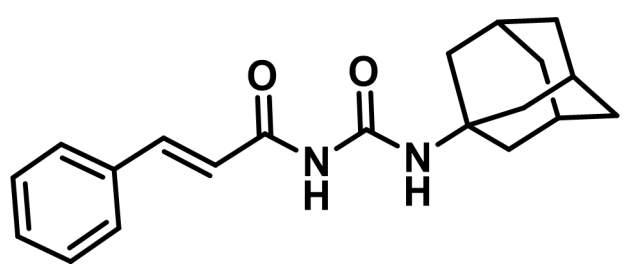

***N-(((3s,5s,7s)-adamantan-1-yl)carbamoyl)cinnamamide* (P30)**

Compound P30 was prepared following the general procedure C (flash chromatography for purification: petroleum ether/ethyl acetate = 15:1−3:1) in 50% yield (48.7 mg) as a white solid, mp: 234.8-236.9 ℃.

Reaction time: 8 h

^{1}H NMR (400 MHz, $CDCl_3$) δ 10.89 (s, 1H), 8.70 (s, 1H), 7.72 (d, *J* = 15.7 Hz, 1H), 7.57 (d, *J* = 6.2 Hz, 2H), 7.43 – 7.30 (m, 3H), 6.76 (d, *J* = 15.7 Hz, 1H), 2.14 (s, 9H), 1.75 (s, 6H).

^{13}C NMR (101 MHz, $CDCl_3$) δ 167.4, 154.3, 143.7, 134.6, 130.5, 129.0, 128.3, 120.3, 51.7, 41.9, 36.5, 29.6.

HRMS（ESI, Q-TOF）m/z: $[M+Na]^+$ Calcd for $NaC_{20}H_{24}O_2N_2$, 347.1730; Found: 347.1739.

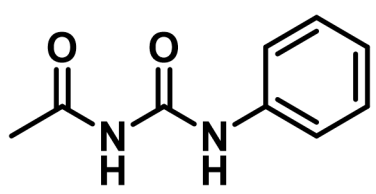

***N-(phenylcarbamoyl)acetamide* (P31)**

Compound P31 was prepared following the general procedure C (flash chromatography for purification: petroleum ether/ethyl acetate = 15:1−3:1) in 73% yield (39.0 mg) as a white solid, mp: 176.5-177.9 ℃.

Reaction time: 6 h

^{1}H NMR (400 MHz, $CDCl_3$) δ 10.69 (s, 1H), 10.39 (s, 1H), 7.53 (d, *J* = 7.8 Hz, 2H), 7.34 (t, *J* = 7.9 Hz, 2H), 7.13 (t, *J* = 7.4 Hz, 1H), 2.24 (s, 3H).

^{13}C NMR (101 MHz, $CDCl_3$) δ 173.1, 152.6, 137.1, 129.1, 124.6, 120.4, 24.2.

The analytical data are in accordance with reported literature.[5]

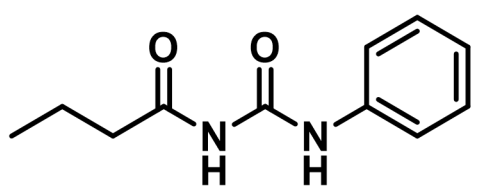

***N-(phenylcarbamoyl)butyramide* (P32)**

Compound P32 was prepared following the general procedure C (flash chromatography for purification: petroleum ether/ethyl acetate = 15:1−3:1) in 56% yield (34.7 mg) as a white solid, mp: 90.4-92.1 ℃.

Reaction time: 6 h

^{1}H NMR (400 MHz, $CDCl_3$) δ 10.71 (s, 1H), 9.98 (s, 1H), 7.54 (d, *J* = 8.0 Hz, 2H), 7.34 (t, *J* = 7.8 Hz, 2H), 7.13 (t, *J* = 7.4 Hz, 1H), 2.43 (t, *J* = 7.5 Hz, 2H), 1.79 – 1.72 (m, 2H), 1.02 (t, *J* = 7.4 Hz, 3H).

^{13}C NMR (101 MHz, $CDCl_3$) δ 177.3, 156.5, 152.4, 130.4, 122.1, 114.3, 55.6, 40.3, 25.0, 18.2.

The analytical data are in accordance with reported literature.[5]

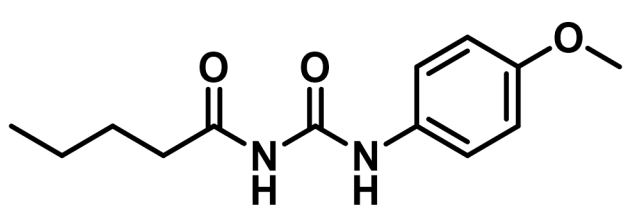

N-((4-methoxyphenyl)carbamoyl)pentanamide **(P33)**

Compound P33 was prepared following the general procedure C (flash chromatography for purification: petroleum ether/ethyl acetate = 15:1−3:1) in 58% yield (43.6 mg) as a white solid, mp: 91.7-93.2 °C.

Reaction time: 6 h

^{1}H NMR (400 MHz, $CDCl_3$) δ 10.56 (s, 1H), 10.11 (s, 1H), 7.43 (d, J = 8.8 Hz, 2H), 6.86 (d, J = 8.7 Hz, 2H), 3.79 (s, 3H), 2.42 (t, J = 7.5 Hz, 2H), 1.78 – 1.61 (m, 2H), 1.49 – 1.34 (m, 2H), 0.94 (t, J = 7.3 Hz, 3H).

^{13}C NMR (101 MHz, $CDCl_3$) δ 176.1, 156.6, 152.6, 130.3, 122.0, 114.3, 55.6, 37.0, 27.2, 22.4, 13.8.

HRMS（ESI, Q-TOF）m/z: $[M+Na]^+$ Calcd for $NaC_{13}H_{18}O_3N_2$, 273.1210; Found: 273.1209.

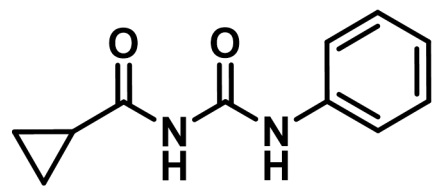

N-(phenylcarbamoyl)cyclopropanecarboxamide **(P34)**

Compound P34 was prepared following the general procedure C (flash chromatography for purification: petroleum ether/ethyl acetate = 15:1−3:1) in 71% yield (43.5 mg) as a white solid, mp: 197.8-199.1 °C.

Reaction time: 6 h

^{1}H NMR (400 MHz, DMSO-d_6) δ 11.03 (s, 1H), 10.60 (s, 1H), 7.49 (d, J = 7.9 Hz, 2H), 7.31 (t, J = 7.9 Hz, 2H), 7.07 (t, J = 7.4 Hz, 1H), 2.00 – 1.79 (m, 1H), 0.92 (d, J = 7.4 Hz, 4H).

^{13}C NMR (101 MHz, DMSO-d_6) δ 176.4, 150.7, 137.7, 129.1, 123.7, 119.7, 14.3, 8.9.

HRMS（ESI, Q-TOF）m/z: $[M+Na]^+$ Calcd for $NaC_{11}H_{12}O_2N_2$, 227.0791; Found: 227.0795.

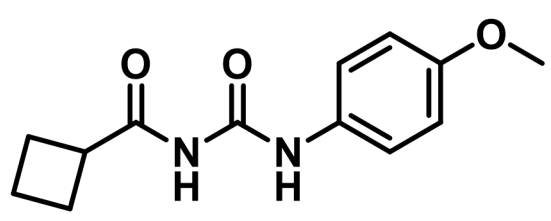

N-((4-methoxyphenyl)carbamoyl)cyclobutanecarboxamide **(P35)**

Compound P35 was prepared following the general procedure C (flash chromatography for purification: petroleum ether/ethyl acetate = 15:1−3:1) in 40% yield (29.8 mg) as a white solid, mp: 112.9-114.2 °C.

Reaction time: 6 h

^{1}H NMR (400 MHz, $CDCl_3$) δ 10.56 (s, 1H), 9.54 (s, 1H), 7.43 (d, J = 9.0 Hz, 2H), 6.88 (d, J = 9.0 Hz, 2H), 3.80 (s, 3H), 3.30 – 3.18 (m, 1H), 2.38 – 2.20 (m, 4H), 2.04 – 1.88 (m, 2H).

^{13}C NMR (101 MHz, $CDCl_3$) δ 177.3, 156.5, 152.4, 130.4, 122.1, 114.3, 55.6, 40.3, 25.0, 18.2.

HRMS（ESI, Q-TOF）m/z: $[M+Na]^+$ Calcd for $NaC_{13}H_{16}O_3N_2$, 271.1053; Found: 271.1062.

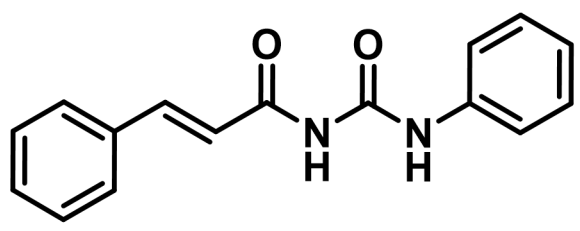

N-(phenylcarbamoyl)cinnamamide **(P36)**

Compound P36 was prepared following the general procedure C (flash chromatography for purification: petroleum ether/ethyl acetate = 15:1−3:1) in 56% yield (44.7 mg) as a white solid,

mp: 201.3-203.0 ℃.

Reaction time: 6 h

^{1}H NMR (400 MHz, DMSO-d_6) δ 10.90 (s, 1H), 10.79 (s, 1H), 7.76 (d, *J* = 15.8 Hz, 1H), 7.68 – 7.59 (m, 2H), 7.55 (d, *J* = 8.0 Hz, 2H), 7.51 – 7.42 (m, 3H), 7.35 (t, *J* = 7.8 Hz, 2H), 7.10 (t, *J* = 7.3 Hz, 1H), 6.88 (d, *J* = 15.8 Hz, 1H).

^{13}C NMR (101 MHz, DMSO-d_6) δ 167.0, 151.2, 143.9, 137.7, 134.0, 130.8, 129.2, 129.1, 128.3, 123.8, 119.9, 119.7.

HRMS（ESI, Q-TOF）m/z: [M+Na]$^+$ Calcd for $NaC_{16}H_{14}O_2N_2$, 289.0947; Found: 289.0955.

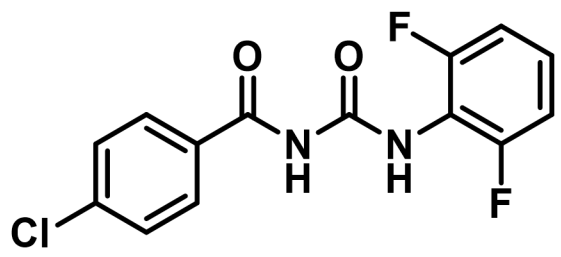

4-chloro-N-((2,6-difluorophenyl)carbamoyl)benzamide **(P37)**

Compound P37 was prepared following the general procedure C (flash chromatography for purification: petroleum ether/ethyl acetate = 15:1−3:1) in 75% yield (69.9 mg) as a white solid, mp: 182.3-184.1 ℃.

Reaction time: 6 h

^{1}H NMR (400 MHz, DMSO-d_6) δ 11.32 (s, 1H), 10.11 (s, 1H), 8.04 (d, *J* = 8.6 Hz, 2H), 7.62 (d, *J* = 8.6 Hz, 2H), 7.46 – 7.33 (m, 1H), 7.20 (t, *J* = 8.2 Hz, 2H).

^{13}C NMR (101 MHz, DMSO-d_6) δ 167.7, 158.0 (dd, *J* = 248.6, 4.8 Hz), 151.5, 138.1, 131.0, 130.4, 128.7, 128.4 (t, *J* = 9.8 Hz), 113.9 (t, *J* = 16.6 Hz), 111.9 (d, *J* = 23.1 Hz).

^{19}F NMR (376 MHz, DMSO-d_6) δ -117.7 (s, 2F).

HRMS（ESI, Q-TOF）m/z: [M+Na]$^+$ Calcd for $NaC_{14}H_9O_2ClF_2N_2$, 333.0213; Found: 333.0215.

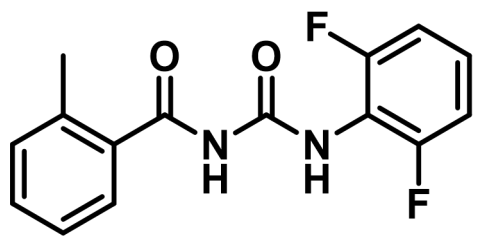

N-((2,6-difluorophenyl)carbamoyl)-2-methylbenzamide **(P38)**

Compound P38 was prepared following the general procedure C (flash chromatography for purification: petroleum ether/ethyl acetate = 15:1−4:1) in 58% yield (50.5 mg) as a white solid, mp: 197.2-199.1 ℃.

Reaction time: 6 h

^{1}H NMR (400 MHz, DMSO-d_6) δ 11.62 (s, 1H), 10.28 (s, 1H), 7.96 (d, *J* = 7.4 Hz, 1H), 7.72 – 7.54 (m, 1H), 7.32 – 7.15 (m, 4H), 7.07 (t, *J* = 7.4 Hz, 1H), 2.29 (s, 3H).

^{13}C NMR (101 MHz, DMSO-d_6) δ 162.7, 158.8 (dd, *J* = 250.4, 6.9 Hz), 150.2, 135.7, 133.4 (t, *J* = 10.0 Hz), 130.4, 128.1, 126.5, 124.3, 121.3, 113.4 (t, *J* = 21.2 Hz), 112.2 (d, *J* = 23.5 Hz), 17.6.

^{19}F NMR (377 MHz, DMSO-d_6) δ -113.2 (s, 2F).

HRMS（ESI, Q-TOF）m/z: [M+Na]$^+$ Calcd for $NaC_{15}H_{12}O_2F_2N_2$, 313.0759; Found: 313.0767.

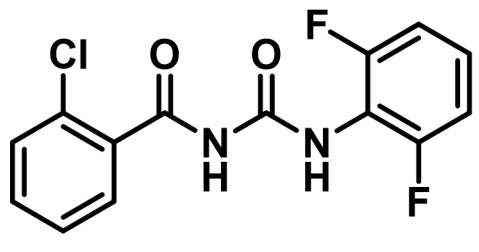

2-chloro-N-((2,6-difluorophenyl)carbamoyl)benzamide **(P39)**

Compound P39 was prepared following the general procedure C (flash chromatography for purification: petroleum ether/ethyl acetate = 15:1−4:1) in 55% yield (51.3 mg) as a white solid, mp: 199.4-201.0 ℃.

Reaction time: 6 h

^{1}H NMR (400 MHz, DMSO-*d$_6$*) δ 11.79 (s, 1H), 10.81 (s, 1H), 8.24 (d, *J* = 8.0 Hz, 1H), 7.72 – 7.58 (m, 1H), 7.56 (d, *J* = 7.9 Hz, 1H), 7.38 (t, *J* = 7.6 Hz, 1H), 7.27 (t, *J* = 8.3 Hz, 2H), 7.17 (t, *J* = 7.7 Hz, 1H).

^{13}C NMR (101 MHz, DMSO-*d$_6$*) δ 162.7, 158.7 (dd, *J* = 250.6, 6.9 Hz), 150.0, 134.3, 133.6 (t, *J* = 9.6 Hz), 129.4, 128.0, 125.2, 122.8, 121.8, 113.1 (t, *J* = 21.1 Hz), 112.3 (d, *J* = 23.7 Hz).

^{19}F NMR (376 MHz, DMSO-*d$_6$*) δ -112.6 (s, 2F).

HRMS（ESI, Q-TOF）m/z: [M+Na]$^+$ Calcd for $NaC_{14}H_9O_2ClF_2N_2$, 333.0213; Found: 333.0221.

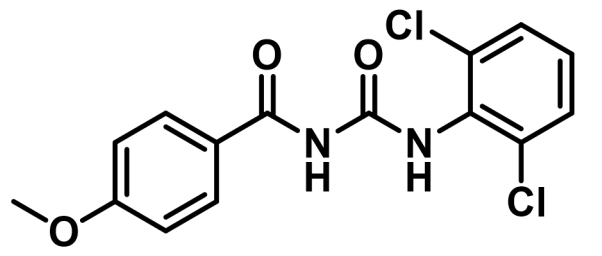

N-((2,6-dichlorophenyl)carbamoyl)-4-methoxybenzamide **(P40)**

Compound P40 was prepared following the general procedure C (flash chromatography for purification: petroleum ether/ethyl acetate = 15:1−4:1) in 49% yield (49.9 mg) as a white solid, mp: 185.3-187.1 ℃.

Reaction time: 8 h

^{1}H NMR (400 MHz, DMSO-*d$_6$*) δ 11.11 (s, 1H), 10.52 (s, 1H), 8.08 (d, *J* = 8.9 Hz, 2H), 7.57 (d, *J* = 8.1 Hz, 2H), 7.42 – 7.33 (m, 1H), 7.07 (d, *J* = 8.9 Hz, 2H), 3.85 (s, 3H).

^{13}C NMR (101 MHz, DMSO-*d$_6$*) δ 167.9, 163.2, 151.6, 133.6, 132.3, 130.7, 129.2, 128.6, 123.9, 114.0, 55.6.

HRMS（ESI, Q-TOF）m/z: [M+Na]$^+$ Calcd for $NaC_{15}H_{12}O_3Cl_2N_2$, 361.0117; Found: 361.0124.

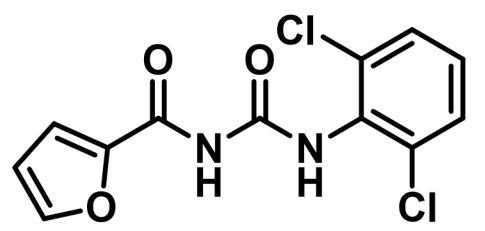

N-((2,6-dichlorophenyl)carbamoyl)furan-2-carboxamide **(P41)**

Compound P41 was prepared following the general procedure C (flash chromatography for purification: petroleum ether/ethyl acetate = 15:1−3:1) in 63% yield (56.5 mg) as a white solid, mp: 181.9-183.8 ℃.

Reaction time: 8 h

^{1}H NMR (400 MHz, DMSO-*d$_6$*) δ 11.16 (s, 1H), 10.17 (s, 1H), 8.06 (d, *J* = 1.2 Hz, 1H), 7.75 (d, *J* = 3.5 Hz, 1H), 7.56 (d, *J* = 8.1 Hz, 2H), 7.42 – 7.33 (m, 1H), 6.79 – 6.72 (m, 1H).

^{13}C NMR (101 MHz, DMSO-*d$_6$*) δ 158.6, 151.1, 148.3, 145.1, 133.7, 132.2, 129.4, 128.5, 118.0, 112.6.

HRMS（ESI, Q-TOF）m/z: [M+Na]$^+$ Calcd for $NaC_{12}H_8O_3Cl_2N_2$, 320.9804; Found: 320.9812.

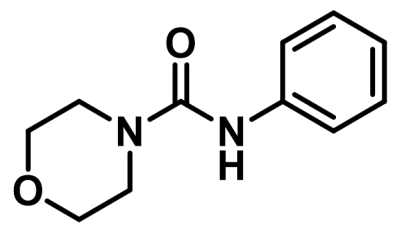

***N*-phenylmorpholine-4-carboxamide** (**P42**)

Compound P42 was prepared following the general procedure D_1 (flash chromatography for purification: petroleum ether/ethyl acetate = 5:1−1:1) in 81% yield (50.1 mg) as a white solid, mp: 148.7-150.4 °C.

Reaction time: 6 h

^{1}H NMR (400 MHz, $CDCl_3$) δ 7.30 (d, *J* = 7.7 Hz, 2H), 7.27 – 7.19 (m, 2H), 7.01 (t, *J* = 7.2 Hz, 1H), 6.91 (s, 1H), 3.71 – 3.55 (m, 4H), 3.48 – 3.34 (m, 4H).

^{13}C NMR (101 MHz, $CDCl_3$) δ 155.5, 138.9, 128.9, 123.4, 120.6, 66.5, 44.3.

The analytical data are in accordance with reported literature.[6]

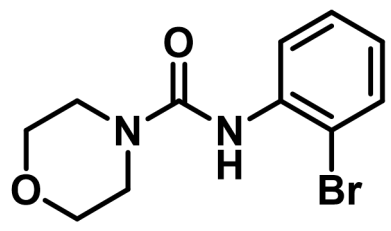

***N*-(2-bromophenyl)morpholine-4-carboxamide** (**P43**)

Compound P43 was prepared following the general procedure D_1 (flash chromatography for purification: petroleum ether/ethyl acetate = 5:1−1:1) in 54% yield (46.2 mg) as a white solid, mp: 111.5-113.3 °C.

Reaction time: 6 h

^{1}H NMR (400 MHz, $CDCl_3$) δ 8.18 (d, *J* = 8.1 Hz, 1H), 7.50 (d, *J* = 8.0 Hz, 1H), 7.29 (t, *J* = 8.0 Hz, 1H), 7.01 (s, 1H), 6.90 (t, *J* = 7.5 Hz, 1H), 3.82 – 3.71 (m, 4H), 3.58 – 3.43 (m, 4H).

^{13}C NMR (101 MHz, $CDCl_3$) δ 154.4, 136.7, 132.1, 128.6, 124.0, 121.3, 113.3, 66.6, 44.3.

The analytical data are in accordance with reported literature.[6]

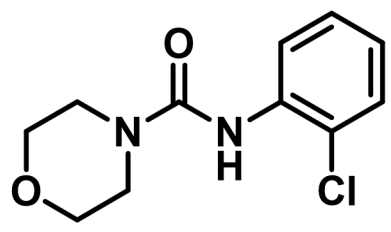

***N*-(2-chlorophenyl)morpholine-4-carboxamide** (**P44**)

Compound P44 was prepared following the general procedure D_1 (flash chromatography for purification: petroleum ether/ethyl acetate = 5:1−1:1) in 54% yield (39.0 mg) as a white solid, mp: 123.6-125.6 °C.

Reaction time: 6 h

^{1}H NMR (400 MHz, $CDCl_3$) δ 8.15 (d, *J* = 8.2 Hz, 1H), 7.31 (d, *J* = 8.0 Hz, 1H), 7.23 (t, *J* = 8.2 Hz, 1H), 7.02 – 6.91 (m, 2H), 3.85 – 3.68 (m, 4H), 3.60 – 3.40 (m, 4H).

^{13}C NMR (101 MHz, $CDCl_3$) δ 154.3, 135.6, 128.9, 127.8, 123.4, 122.5, 121.1, 66.5, 44.2.

The analytical data are in accordance with reported literature.[7]

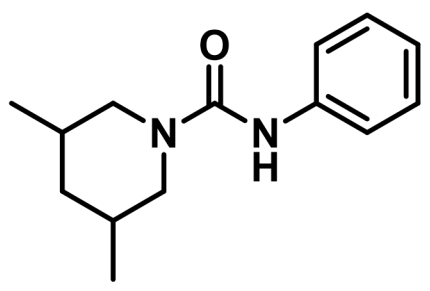

3,5-dimethyl-N-phenylpiperidine-1-carboxamide (**P45**)

Compound P45 was prepared following the general procedure D_1 (flash chromatography for purification: petroleum ether/ethyl acetate = 15:1−3:1) in 55% yield (38.3 mg) as a white solid, mp: 175.8-177.6 °C.

Reaction time: 6 h

^{1}H NMR (400 MHz, $CDCl_3$) δ 7.38 – 7.31 (m, 2H), 7.25 (t, *J* = 7.9 Hz, 2H), 7.03 – 6.96 (m, 1H), 6.48 (s, 1H), 4.05 – 3.92 (m, 2H), 2.37 – 2.24 (m, 2H), 1.88 – 1.78 (m, 1H), 1.73 – 1.55 (m, 2H), 0.95 – 0.81 (m, 6H), 0.80 – 0.67 (m, 1H).

^{13}C NMR (101 MHz, $CDCl_3$) δ 154.8, 139.4, 128.9, 122.9, 119.9, 51.6, 42.4, 31.3, 19.2.

HRMS（ESI, Q-TOF）m/z: $[M+H]^+$ Calcd for $C_{14}H_{21}ON_2$, 233.1648; Found: 233.1654.

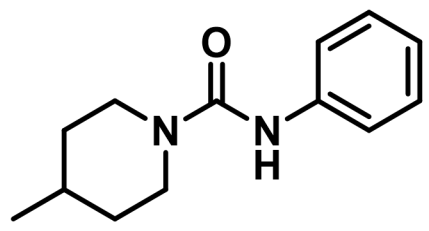

4-methyl-N-phenylpiperidine-1-carboxamide **(P46)**

Compound P46 was prepared following the general procedure D_1 (flash chromatography for purification: petroleum ether/ethyl acetate = 15:1−3:1) in 45% yield (29.5 mg) as a white solid, mp: 104.4-106.5 °C.

Reaction time: 6 h

^{1}H NMR (400 MHz, $CDCl_3$) δ 7.34 (d, *J* = 7.9 Hz, 2H), 7.25 (t, *J* = 7.7 Hz, 2H), 6.99 (t, *J* = 7.3 Hz, 1H), 6.51 (s, 1H), 4.02 (d, *J* = 13.1 Hz, 2H), 2.82 (t, *J* = 12.7 Hz, 2H), 1.66 (d, *J* = 13.1 Hz, 2H), 1.61 – 1.49 (m, 1H), 1.21 – 1.08 (m, 2H), 0.95 (d, *J* = 6.5 Hz, 3H).

^{13}C NMR (101 MHz, $CDCl_3$) δ 155.2, 139.5, 128.9, 122.9, 120.0, 44.8, 34.0, 31.1, 21.9.

HRMS（ESI, Q-TOF）m/z: $[M+H]^+$ Calcd for $C_{13}H_{19}ON_2$, 219.1492; Found: 219.1498.

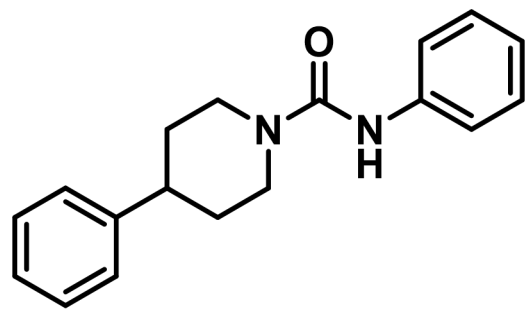

N,4-diphenylpiperidine-1-carboxamide **(P47)**

Compound P47 was prepared following the general procedure D_1 (flash chromatography for purification: petroleum ether/ethyl acetate = 15:1−3:1) in 36% yield (30.3 mg) as a white solid, mp: 146.6-148.4 °C.

Reaction time: 6 h

^{1}H NMR (400 MHz, $CDCl_3$) δ 7.44 – 7.36 (m, 2H), 7.37 – 7.27 (m, 4H), 7.23 (t, *J* = 9.4 Hz, 3H), 7.04 (t, *J* = 7.3 Hz, 1H), 6.60 (s, 1H), 4.23 (d, *J* = 13.2 Hz, 2H), 2.98 (t, *J* = 12.6 Hz, 2H), 2.72 (t, *J* = 12.1 Hz, 1H), 1.91 (d, *J* = 12.5 Hz, 2H), 1.79 – 1.69 (m, 2H).

^{13}C NMR (101 MHz, $CDCl_3$) δ 155.1, 145.4, 139.3, 129.0, 128.7, 126.9, 126.6, 123.1, 120.1, 45.1, 42.8, 33.2.

HRMS（ESI, Q-TOF）m/z: $[M+H]^+$ Calcd for $C_{18}H_{21}ON_2$, 281.1648; Found: 281.1656.

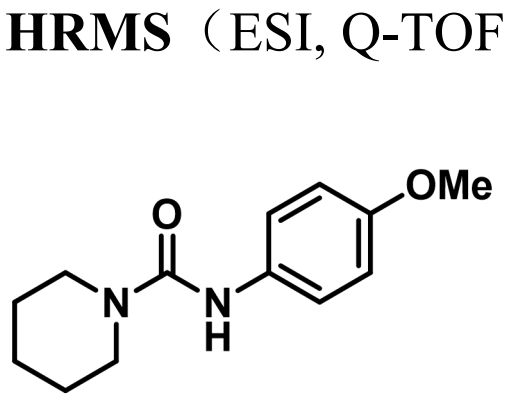

N-(4-methoxyphenyl)piperidine-1-carboxamide **(P48)**

Compound P48 was prepared following the general procedure D_1 (flash chromatography for purification: petroleum ether/ethyl acetate = 10:1−3:1) in 23% yield (16.2 mg) as a white solid, mp: 118.6-120.3 °C.

Reaction time: 6 h

^{1}H NMR (400 MHz, $CDCl_3$) δ 7.23 (d, J = 9.0 Hz, 2H), 6.81 (d, J = 9.0 Hz, 2H), 6.38 (s, 1H), 3.76 (s, 3H), 3.45 – 3.36 (m, 4H), 1.63 – 1.54 (m, 6H).

^{13}C NMR (101 MHz, $CDCl_3$) δ 155.8, 155.7, 132.5, 122.4, 114.1, 55.6, 45.3, 25.8, 24.5.

The analytical data are in accordance with reported literature.[8]

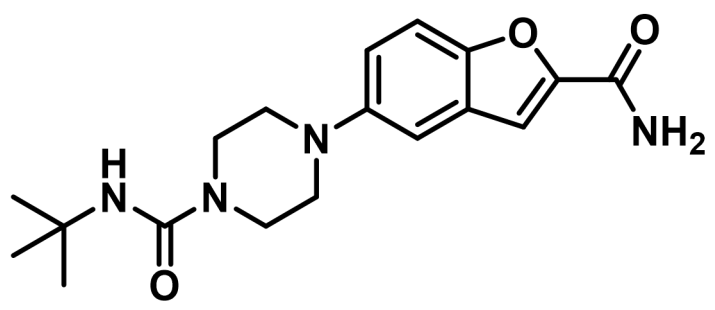

N-(tert-butyl)-4-(2-carbamoylbenzofuran-5-yl)piperazine-1-carboxamide **(P49)**

Compound P49 was prepared following the general procedure D_2 (flash chromatography for purification: petroleum ether/ethyl acetate = 1:1−1:5) in 62% yield (64.1 mg) as a white solid, mp: 191.5-193.8 ℃.

Reaction time: 6 h

^{1}H NMR (400 MHz, DMSO-d_6) δ 8.06 (s, 1H), 7.64 (s, 1H), 7.54 – 7.44 (m, 1H), 7.42 (s, 1H), 7.25 – 7.15 (m, 2H), 3.49 – 3.40 (m, 4H), 3.38 (s, 1H), 3.11 – 3.00 (m, 4H), 1.26 (s, 9H).

^{13}C NMR (101 MHz, DMSO-d_6) δ 159.9, 157.1, 149.6, 149.3, 148.3, 127.8, 118.6, 111.9, 109.7, 108.1, 50.0, 49.9, 43.8, 29.2.

HRMS（ESI, Q-TOF）m/z: $[M+H]^+$ Calcd for $C_{18}H_{25}O_3N_4$, 345.1921; Found: 345.1927.

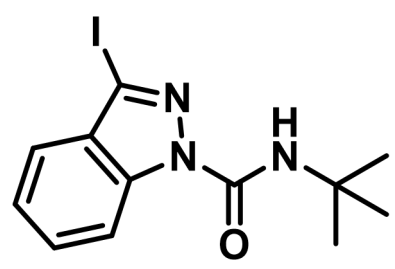

N-(tert-butyl)-3-iodo-1H-indazole-1-carboxamide **(P50)**

Compound P50 was prepared following the general procedure D_2 (flash chromatography for purification: petroleum ether/ethyl acetate = 30:1−10:1) in 62% yield (63.8 mg) as a white solid, mp: 118.1-119.8 ℃.

Reaction time: 6 h

^{1}H NMR (400 MHz, $CDCl_3$) δ 8.35 (d, J = 8.5 Hz, 1H), 7.63 – 7.50 (m, 1H), 7.45 (d, J = 8.1 Hz, 1H), 7.31 (t, J = 7.4 Hz, 1H), 7.02 (s, 1H), 1.51 (s, 9H).

^{13}C NMR (101 MHz, $CDCl_3$) δ 149.6, 139.2, 130.0, 129.9, 123.7, 121.6, 114.9, 99.0, 51.7, 29.1.

HRMS（ESI, Q-TOF）m/z: $[M+H]^+$ Calcd for $C_{12}H_{15}OIN_3$, 344.0254; Found: 344.0254.

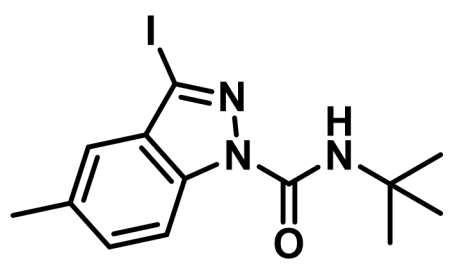

N-(tert-butyl)-3-iodo-5-methyl-1H-indazole-1-carboxamide **(P51)**

Compound P51 was prepared following the general procedure D_2 (flash chromatography for purification: petroleum ether/ethyl acetate = 20:1−10:1) in 65% yield, mp: 116.6-118.4 ℃.

Reaction time: 6 h

^{1}H NMR (400 MHz, $CDCl_3$) δ 8.20 (d, *J* = 8.6 Hz, 1H), 7.36 (d, *J* = 8.6 Hz, 1H), 7.19 (s, 1H), 6.99 (s, 1H), 2.47 (s, 3H), 1.51 (s, 9H).

^{13}C NMR (101 MHz, $CDCl_3$) δ 149.6, 137.7, 133.6, 131.9, 130.1, 120.7, 114.5, 98.6, 51.6, 29.2, 29.1, 21.4.

HRMS（ESI, Q-TOF）m/z: $[M+H]^+$ Calcd for $C_{13}H_{16}OIN_3$, 380.0230; Found: 380.0234.

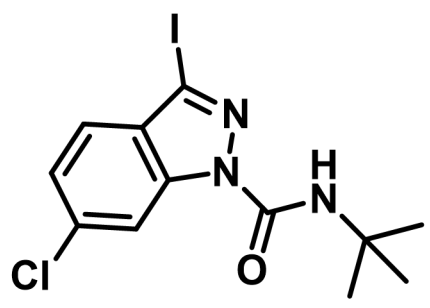

N-(tert-butyl)-6-chloro-3-iodo-1H-indazole-1-carboxamide **(P52)**

Compound P52 was prepared following the general procedure D_2 (flash chromatography for purification: petroleum ether/ethyl acetate = 20:1−10:1) in 67% yield (75.9 mg) as a white solid, mp: 180.8-182.7 ℃.

Reaction time: 6 h

^{1}H NMR (400 MHz, $CDCl_3$) δ 8.41 (s, 1H), 7.34 (d, *J* = 8.6 Hz, 1H), 7.26 (d, *J* = 8.6 Hz, 1H), 6.97 (s, 1H), 1.51 (s, 9H).

^{13}C NMR (101 MHz, $CDCl_3$) δ 149.2, 139.4, 136.8, 128.5, 124.8, 122.5, 114.9, 98.5, 51.8, 29.0.

HRMS（ESI, Q-TOF）m/z: $[M+H]^+$ Calcd for $C_{12}H_{14}ClOIN_3$, 377.9865; Found: 377.9866.

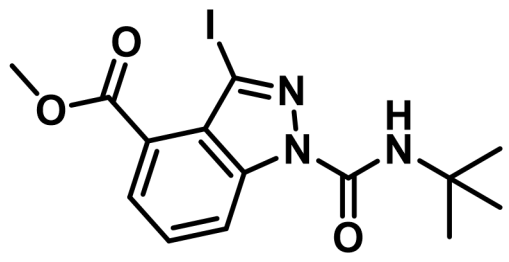

methyl 1-(tert-butylcarbamoyl)-3-iodo-1H-indazole-4-carboxylate **(P53)**

Compound P53 was prepared following the general procedure D_2 (flash chromatography for purification: petroleum ether/ethyl acetate = 20:1−10:1) in 50% yield (60.2 mg) as a white solid, mp: 143.2-145.2 ℃.

Reaction time: 6 h

^{1}H NMR (400 MHz, $CDCl_3$) δ 8.64 (d, *J* = 8.5 Hz, 1H), 7.64 (d, *J* = 7.3 Hz, 1H), 7.58 – 7.51 (m, 1H), 7.06 (s, 1H), 4.02 (s, 3H), 1.51 (s, 9H).

^{13}C NMR (101 MHz, $CDCl_3$) δ 166.6, 149.2, 139.8, 128.8, 126.1, 125.4, 125.3, 118.3, 94.8, 52.4, 51.9, 29.0.

HRMS（ESI, Q-TOF）m/z: $[M+H]^+$ Calcd for $C_{14}H_{17}O_3IN_3$, 402.0309; Found: 402.0312.

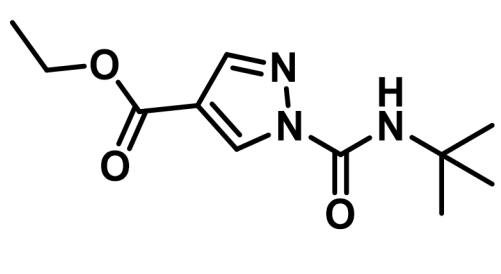

ethyl 1-(tert-butylcarbamoyl)-1H-pyrazole-4-carboxylate **(P54)**

Compound P54 was prepared following the general procedure D_2 (flash chromatography for purification: petroleum ether/ethyl acetate = 15:1−3:1) in 87% yield (62.5 mg) as a white solid, mp: 41.8-42.3 ℃.

Reaction time: 6 h

^{1}H NMR (400 MHz, $CDCl_3$) δ 8.61 (s, 1H), 7.90 (s, 1H), 7.09 (s, 1H), 4.36 – 4.21 (m, 2H), 1.44 (s, 9H), 1.32 (t, *J* = 6.5 Hz, 3H).
^{13}C NMR (101 MHz, $CDCl_3$) δ 162.4, 147.2, 142.2, 131.4, 117.4, 60.7, 51.9, 28.7, 14.4.
HRMS（ESI, Q-TOF）m/z: $[M+Na]^+$ Calcd for $NaC_{11}H_{17}O_3N_3$, 262.1162; Found: 262.1169.

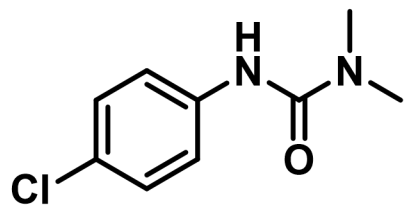

3-(4-chlorophenyl)-1,1-dimethylurea **(P55, Monuron)**
Compound P55 was prepared following the general procedure E (flash chromatography for purification: petroleum ether/ethyl acetate = 5:1−1:1) in 41% yield (24.5 mg) as a white solid, mp: 162.9-164.0 ℃.
Reaction time: 10 h
^{1}H NMR (400 MHz, $CDCl_3$) δ 7.33 (d, *J* = 8.7 Hz, 2H), 7.23 (d, *J* = 8.8 Hz, 2H), 6.32 (s, 1H), 3.02 (s, 6H).
^{13}C NMR (101 MHz, $CDCl_3$) δ 155.6, 137.9, 128.9, 128.0, 121.2, 36.6.
The analytical data are in accordance with reported literature.[9]

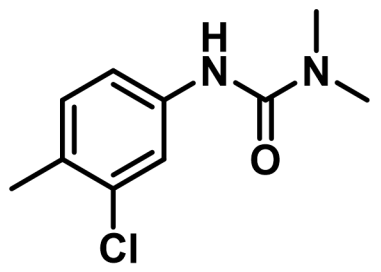

3-(3-chloro-4-methylphenyl)-1,1-dimethylurea **(P56, Chlortoluron)**
Compound P56 was prepared following the general procedure E (flash chromatography for purification: petroleum ether/ethyl acetate = 5:1−1:1) in 38% yield (24.3 mg) as a white solid, mp: 136.2-138.5 ℃.
Reaction time: 10 h
^{1}H NMR (400 MHz, $CDCl_3$) δ 7.48 – 7.44 (m, 1H), 7.20 – 7.13 (m, 1H), 7.13 – 7.07 (m, 1H), 6.27 (s, 1H), 3.02 (s, 6H), 2.30 (s, 3H).
^{13}C NMR (101 MHz, $CDCl_3$) δ 155.6, 138.1, 134.4, 131.0, 130.4, 120.5, 118.3, 36.6, 19.5.
The analytical data are in accordance with reported literature.[9]

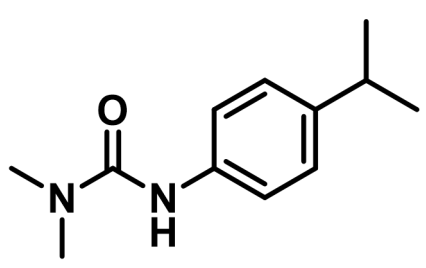

3-(4-isopropylphenyl)-1,1-dimethylurea **(P57, Isoproturon)**
Compound P57 was prepared following the general procedure E (flash chromatography for purification: petroleum ether/ethyl acetate = 5:1−1:1) in 79% yield (48.9 mg) as a white solid, mp: 148.9-150.5 ℃.
Reaction time: 10 h
^{1}H NMR (400 MHz, $CDCl_3$) δ 7.28 (d, *J* = 8.4 Hz, 2H), 7.13 (d, *J* = 8.4 Hz, 2H), 6.29 (s, 1H), 3.01 (s, 6H), 2.92 – 2.79 (m, 1H), 1.22 (d, *J* = 6.9 Hz, 6H).
^{13}C NMR (101 MHz, $CDCl_3$) δ 156.0, 143.7, 136.9, 126.8, 120.3, 36.6, 33.6, 24.2.

The analytical data are in accordance with reported literature.[10]

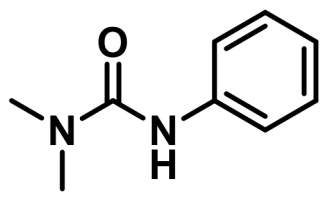

1,1-dimethyl-3-phenylurea **(P58, Fenuron)**

Compound P58 was prepared following the general procedure E (flash chromatography for purification: petroleum ether/ethyl acetate = 5:1−1:1) in 82% yield (40.4 mg) as a white solid, mp: 120.1-121.7 ℃.

Reaction time: 10 h

^{1}H NMR (400 MHz, $CDCl_3$) δ 7.38 (d, J = 7.8 Hz, 2H), 7.30 – 7.26 (m, 2H), 7.02 (t, J = 7.3 Hz, 1H), 6.35 (s, 1H), 3.03 (s, 6H).

^{13}C NMR (101 MHz, $CDCl_3$) δ 155.8, 139.3, 129.0, 123.1, 119.9, 36.6.

The analytical data are in accordance with reported literature.[9]

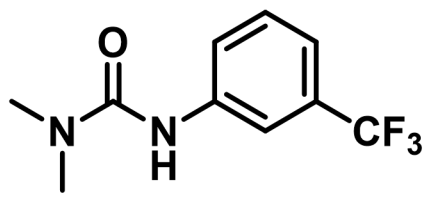

1,1-dimethyl-3-(3-(trifluoromethyl)phenyl)urea **(P59, Fluometuron)**

Compound P59 was prepared following the general procedure E (flash chromatography for purification: petroleum ether/ethyl acetate = 5:1−1:1) in 17% yield (11.9 mg) as a white solid, mp: 146.5-148.3 ℃.

Reaction time: 10 h

^{1}H NMR (400 MHz, $CDCl_3$) δ 7.67 (s, 1H), 7.59 (d, J = 8.2 Hz, 1H), 7.38 (t, J = 8.0 Hz, 1H), 7.28 (s, 1H), 6.44 (s, 1H), 3.05 (s, 6H).

^{13}C NMR (101 MHz, $CDCl_3$) δ 155.4, 139.9, 129.5, 125.5, 122.84, 122.77, 119.6 (d, J = 3.8 Hz), 116.5 (d, J = 3.9 Hz), 36.6.

^{19}F NMR (377 MHz, $CDCl_3$) δ -62.7 (s, 3F).

The analytical data are in accordance with reported literature.[11]

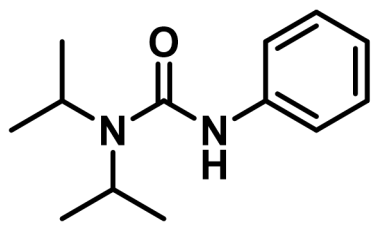

1,1-diisopropyl-3-phenylurea **(P60)**

Compound P60 was prepared following the general procedure D_1 (flash chromatography for purification: petroleum ether/ethyl acetate = 15:1−3:1) in 21% yield (13.9 mg) as a white solid, mp: 109.0-110.2 ℃.

Reaction time: 6 h

^{1}H NMR (400 MHz, $CDCl_3$) δ 7.36 (d, J = 8.1 Hz, 2H), 7.26 (t, J = 7.9 Hz, 2H), 6.99 (t, J = 7.3 Hz, 1H), 6.22 (s, 1H), 4.06 – 3.89 (m, 2H), 1.42 – 1.21 (m, 12H).

^{13}C NMR (101 MHz, $CDCl_3$) δ 154.7, 139.5, 128.9, 122.7, 119.8, 45.6, 21.6.

The analytical data are in accordance with reported literature.[12]

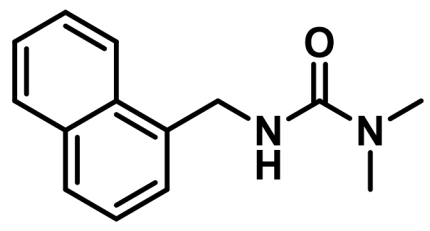

***1,1-dimethyl-3-(naphthalen-1-ylmethyl)urea* (P61)**

Compound P61 (amine substrate: Dimethylamine hydrochloride) was prepared following the general procedure F (flash chromatography for purification: petroleum ether/ethyl acetate = 5:1−1:1) in 42% yield (28.8 mg) as a yellow oil.

Reaction time: 12 h

^{1}H NMR (400 MHz, $CDCl_3$) δ 8.09 (d, *J* = 8.1 Hz, 1H), 7.87 (d, *J* = 7.8 Hz, 1H), 7.80 (d, *J* = 7.8 Hz, 1H), 7.59 – 7.48 (m, 2H), 7.48 – 7.38 (m, 2H), 4.87 (d, *J* = 4.8 Hz, 2H), 4.63 (s, 1H), 2.89 (s, 6H).

^{13}C NMR (101 MHz, $CDCl_3$) δ 158.2, 134.8, 134.0, 131.7, 128.8, 128.5, 126.6, 126.0, 125.5, 123.9, 43.4, 36.4.

HRMS（ESI, Q-TOF）m/z: $[M+H]^+$ Calcd for $C_{14}H_{17}ON_2$, 229.1335; Found: 229.1339.

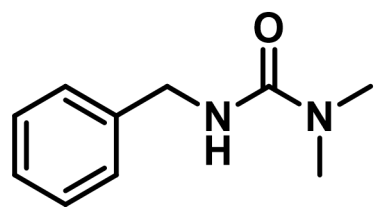

***3-benzyl-1,1-dimethylurea* (P62)**

Compound P62 (amine substrate: Dimethylamine hydrochloride) was prepared following the general procedure F (flash chromatography for purification: petroleum ether/ethyl acetate = 5:1−1:1) in 50% yield (26.7 mg) as a colorless oil.

Reaction time: 12 h

^{1}H NMR (400 MHz, $CDCl_3$) δ 7.36 – 7.24 (m, 5H), 4.65 (s, 1H), 4.42 (d, *J* = 5.4 Hz, 2H), 2.91 (s, 6H).

^{13}C NMR (101 MHz, $CDCl_3$) δ 158.4, 139.8, 128.7, 127.9, 127.4, 45.2, 36.4.

HRMS（ESI, Q-TOF）m/z: $[M+H]^+$ Calcd for $C_{10}H_{15}ON_2$, 179.1179; Found: 179.1185.

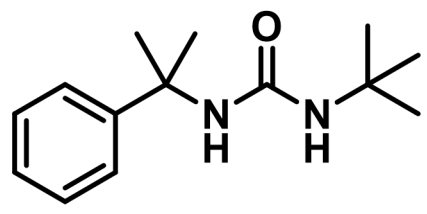

***1-(tert-butyl)-3-(2-phenylpropan-2-yl)urea* (P63)**

Compound P63 was prepared following the general procedure D_1 (flash chromatography for purification: petroleum ether/ethyl acetate = 10:1−2:1) in 70% yield (49.2 mg) as a white solid, mp: 161.1-162.3 ℃.

Reaction time: 6 h

^{1}H NMR (400 MHz, $CDCl_3$) δ 7.47 (d, *J* = 7.5 Hz, 2H), 7.35 (t, *J* = 7.6 Hz, 2H), 7.26 (t, *J* = 7.3 Hz, 1H), 4.78 (s, 1H), 4.00 (s, 1H), 1.59 (s, 6H), 1.09 (s, 9H).

^{13}C NMR (101 MHz, $CDCl_3$) δ 157.1, 146.6, 128.8, 127.2, 125.6, 54.6, 50.3, 30.5, 29.3.

HRMS（ESI, Q-TOF）m/z: $[M+H]^+$ Calcd for $C_{14}H_{23}ON_2$, 235.1805; Found: 235.1808.

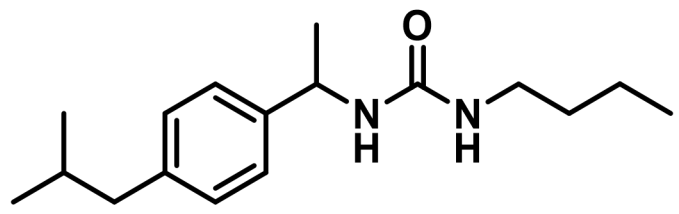

1-butyl-3-(1-(4-isobutylphenyl)ethyl)urea **(P64)**

Compound P64 was prepared following the general procedure D_1 (flash chromatography for purification: petroleum ether/ethyl acetate = 10:1−2:1) in 72% yield (59.7 mg) as a colorless oil.

Reaction time: 6 h

^{1}H NMR (400 MHz, $CDCl_3$) δ 7.21 (d, J = 8.0 Hz, 2H), 7.08 (d, J = 8.0 Hz, 2H), 4.97 (d, J = 6.6 Hz, 1H), 4.78 – 4.68 (m, 1H), 4.58 (s, 1H), 3.19 – 2.97 (m, 2H), 2.43 (d, J = 7.2 Hz, 2H), 1.87 – 1.75 (m, 1H), 1.41 (d, J = 6.8 Hz, 3H), 1.37 – 1.29 (m, 2H), 1.25 – 1.15 (m, 2H), 0.88 (d, J = 6.6 Hz, 6H), 0.83 (t, J = 7.3 Hz, 3H).

^{13}C NMR (101 MHz, $CDCl_3$) δ 158.1, 141.6, 140.8, 129.5, 125.8, 50.1, 45.2, 40.2, 32.3, 30.3, 23.6, 22.5, 20.0, 13.9.

HRMS（ESI, Q-TOF）m/z: $[M+H]^+$ Calcd for $C_{17}H_{29}ON_2$, 277.2274; Found: 277.2277.

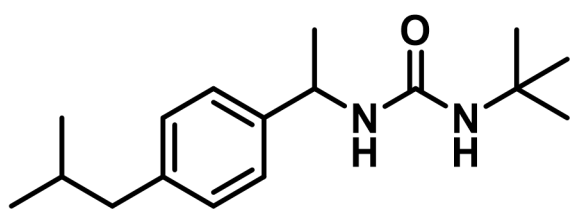

1-(tert-butyl)-3-(1-(4-isobutylphenyl)ethyl)urea **(P65)**

Compound P65 was prepared following the general procedure D_1 (flash chromatography for purification: petroleum ether/ethyl acetate = 10:1−2:1) in 60% yield (49.8 mg) as a white solid, mp: 170.6-171.9 °C.

Reaction time: 6 h

^{1}H NMR (400 MHz, $CDCl_3$) δ 7.20 (d, J = 8.0 Hz, 2H), 7.08 (d, J = 8.1 Hz, 2H), 4.81 (d, J = 6.6 Hz, 1H), 4.73 – 4.62 (m, 1H), 4.43 (s, 1H), 2.44 (d, J = 7.2 Hz, 2H), 1.88 – 1.76 (m, 1H), 1.39 (d, J = 6.8 Hz, 3H), 1.22 (s, 9H), 0.88 (d, J = 6.6 Hz, 6H).

^{13}C NMR (101 MHz, $CDCl_3$) δ 157.3, 141.8, 140.7, 129.5, 125.8, 50.4, 50.1, 45.1, 30.3, 29.5, 23.7, 22.5.

HRMS（ESI, Q-TOF）m/z: $[M+H]^+$ Calcd for $C_{17}H_{29}ON_2$, 277.2274; Found: 277.2280.

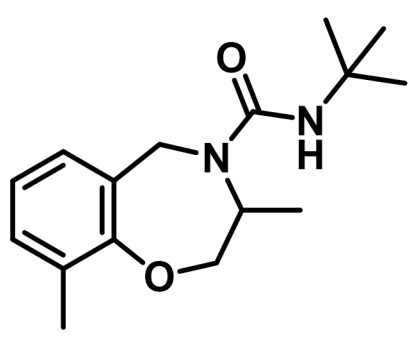

N-(tert-butyl)-3,9-dimethyl-2,3-dihydrobenzo[f][1,4]oxazepine-4(5H)-carboxamide **(P66)**

Compound P66 (amine substrate: Mexiletine hydrochloride) was prepared following the general procedure D_2 (flash chromatography for purification: petroleum ether/ethyl acetate = 10:1−2:1) in 35% yield (29.1 mg) as a yellow oil.

Reaction time: 6 h

^{1}H NMR (400 MHz, $CDCl_3$) δ 7.06 (d, J = 7.3 Hz, 1H), 6.94 (d, J = 6.5 Hz, 1H), 6.86 (t, J = 7.4 Hz, 1H), 4.70 – 4.62 (m, 1H), 4.58 (d, J = 17.0 Hz, 1H), 4.21 (s, 1H), 4.15 (d, J = 17.0 Hz, 1H), 4.11 – 3.99 (m, 2H), 2.20 (s, 3H), 1.27 (d, J = 6.8 Hz, 3H), 1.24 (s, 9H).

^{13}C NMR (101 MHz, $CDCl_3$) δ 157.33, 157.32, 130.4, 129.7, 127.6, 126.3, 122.2, 74.7, 50.9, 50.7, 43.9, 29.5, 16.4, 14.8.

HRMS（ESI, Q-TOF）m/z: $[M+H]^+$ Calcd for $C_{16}H_{25}O_2N_2$, 277.1911; Found: 277.1915.

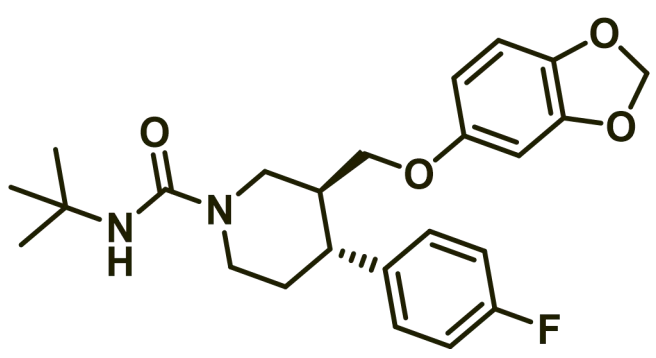

(3S,4R)-3-((benzo[d][1,3]dioxol-5-yloxy)methyl)-N-(tert-butyl)-4-(4-fluorophenyl)piperidine-1-carboxamide **(P67)**

Compound P67 was prepared following the general procedure D_2 (flash chromatography for purification: petroleum ether/ethyl acetate = 10:1−2:1) in 71% yield (91.3 mg) as a colorless oil.

Reaction time: 6 h

^{1}H NMR (400 MHz, $CDCl_3$) δ 7.17 – 7.08 (m, 2H), 6.97 (t, *J* = 8.6 Hz, 2H), 6.61 (d, *J* = 8.5 Hz, 1H), 6.34 (d, *J* = 2.4 Hz, 1H), 6.16 – 6.10 (m, 1H), 5.86 (s, 2H), 4.43 (s, 1H), 4.26 – 3.99 (m, 2H), 3.64 – 3.39 (m, 2H), 2.90 – 2.74 (m, 2H), 2.71 – 2.56 (m, 1H), 2.10 – 2.03 (m, 1H), 1.87 – 1.66 (m, 2H), 1.37 (s, 9H).

^{13}C NMR (101 MHz, $CDCl_3$) δ 161.7 (d, *J* = 244.7 Hz), 157.0, 154.2, 148.3, 141.8, 139.1 (d, *J* = 3.1 Hz), 128.8 (d, *J* = 7.9 Hz), 115.6 (d, *J* = 21.1 Hz), 107.9, 105.6, 101.2, 98.0, 68.9, 50.9, 47.8, 44.7, 44.2, 41.8, 33.9, 29.6.

^{19}F NMR (377 MHz, $CDCl_3$) δ -116.1 (s, 1F).

The analytical data are in accordance with reported literature.[13]

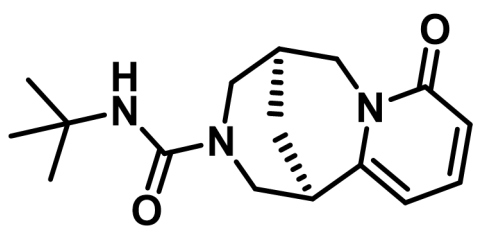

(1R,5R)-N-(tert-butyl)-8-oxo-1,5,6,8-tetrahydro-2H-1,5-methanopyrido[1,2-a][1,5]diazocine-3(4H)-carboxamide **(P68)**

Compound P68 was prepared following the general procedure D_2 (flash chromatography for purification: petroleum ether/ethyl acetate = 1:1−1:5) in 55% yield (47.8 mg) as a white solid, mp: 194.1-195.0 °C.

Reaction time: 6 h

^{1}H NMR (400 MHz, $CDCl_3$) δ 7.25 – 7.19 (m, 1H), 6.40 (d, *J* = 10.1 Hz, 1H), 6.03 (d, *J* = 6.8 Hz, 1H), 4.18 (s, 1H), 4.11 (d, *J* = 15.6 Hz, 1H), 4.01 – 3.88 (m, 2H), 3.87 – 3.75 (m, 1H), 3.06 – 2.96 (m, 3H), 2.43 (s, 1H), 2.01 – 1.84 (m, 2H), 1.15 (s, 9H).

^{13}C NMR (101 MHz, $CDCl_3$) δ 163.5, 156.9, 149.3, 138.8, 117.3, 105.3, 51.5, 50.8, 49.2, 34.7, 29.2, 27.6, 26.0.

HRMS（ESI, Q-TOF）m/z: $[M+H]^+$ Calcd for $C_{16}H_{24}O_2N_3$, 290.1863; Found: 290.1870.

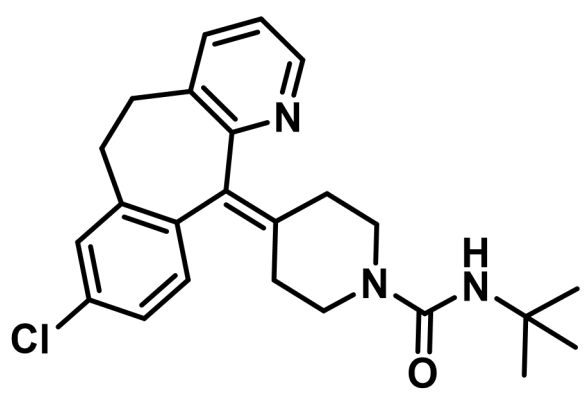

N-(tert-butyl)-4-(8-chloro-5,6-dihydro-11H-benzo[5,6]cyclohepta[1,2-b]pyridin-11-ylidene)piperidine-1-carboxamide **(P69)**

Compound P69 was prepared following the general procedure D_2 (flash chromatography for

purification: petroleum ether/ethyl acetate = 1:1−1:5) in 80% yield (98.4 mg) as a white solid, mp: 169.8-171.1 °C.
Reaction time: 6 h
^{1}H NMR (400 MHz, $CDCl_3$) δ 8.33 (d, *J* = 4.7 Hz, 1H), 7.40 – 7.35 (m, 1H), 7.12 – 7.01 (m, 4H), 4.33 (s, 1H), 3.64 – 3.52 (m, 2H), 3.38 – 3.22 (m, 2H), 3.07 – 2.94 (m, 2H), 2.85 – 2.68 (m, 2H), 2.54 – 2.44 (m, 1H), 2.41 – 2.22 (m, 3H), 1.28 (s, 9H).
^{13}C NMR (101 MHz, $CDCl_3$) δ 157.1, 156.9, 146.7, 139.6, 137.7, 137.59, 137.55, 134.0, 133.4, 132.9, 130.6, 129.0, 126.2, 122.3, 50.8, 44.8, 44.7, 31.7, 31.5, 30.5, 29.5, 27.6.
The analytical data are in accordance with reported literature.[13]

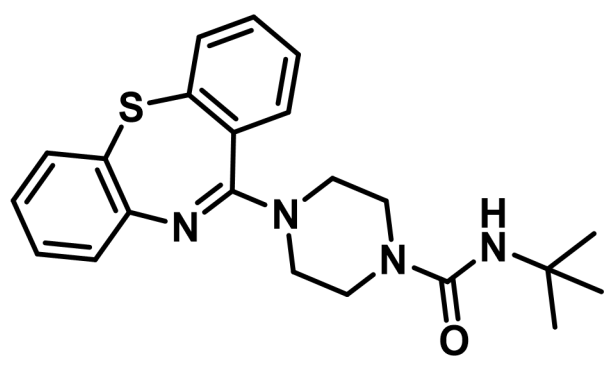

N-(tert-butyl)-4-(dibenzo[b,f][1,4]thiazepin-11-yl)piperazine-1-carboxamide **(P70)**
Compound P70 was prepared following the general procedure D_2 (flash chromatography for purification: petroleum ether/ethyl acetate = 10:1−2:1) in 87% yield (103.0 mg) as a white solid, mp: 196.4-197.9 °C.
Reaction time: 6 h
^{1}H NMR (400 MHz, $CDCl_3$) δ 7.50 (d, *J* = 7.3 Hz, 1H), 7.38 (d, *J* = 7.7 Hz, 1H), 7.36 – 7.27 (m, 3H), 7.17 (t, *J* = 8.3 Hz, 1H), 7.07 (d, *J* = 8.0 Hz, 1H), 6.88 (t, *J* = 7.5 Hz, 1H), 4.38 (s, 1H), 3.73 – 3.20 (m, 8H), 1.35 (s, 9H).
^{13}C NMR (101 MHz, $CDCl_3$) δ 160.8, 157.1, 148.7, 140.0, 134.0, 132.3, 131.1, 129.2, 128.9, 128.5, 128.0, 125.3, 123.1, 50.9, 46.6, 43.5, 29.5.
The analytical data are in accordance with reported literature.[13]

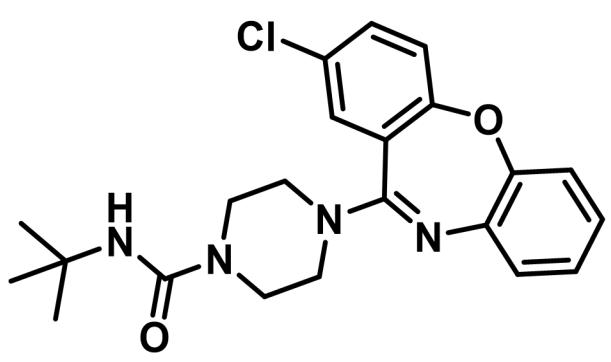

N-(tert-butyl)-4-(2-chlorodibenzo[b,f][1,4]oxazepin-11-yl)piperazine-1-carboxamide **(P71)**
Compound P71 was prepared following the general procedure D_2 (flash chromatography for purification: petroleum ether/ethyl acetate = 1:1−1:5) in 85% yield (105.3 mg) as a white solid, mp: 195.4-196.9 °C.
Reaction time: 6 h
^{1}H NMR (400 MHz, $CDCl_3$) δ 7.39 – 7.33 (m, 1H), 7.28 (d, *J* = 2.5 Hz, 1H), 7.18 – 7.10 (m, 2H), 7.10 – 7.03 (m, 2H), 7.00 – 6.94 (m, 1H), 4.43 (s, 1H), 3.64 – 3.27 (m, 8H), 1.35 (s, 9H).
^{13}C NMR (101 MHz, $CDCl_3$) δ 159.3, 158.8, 157.1, 151.7, 139.9, 132.7, 130.3, 128.9, 127.1, 125.8, 124.83, 124.79, 122.8, 120.2, 50.9, 47.1, 43.5, 29.4.
HRMS（ESI, Q-TOF）m/z: $[M+H]^+$ Calcd for $C_{22}H_{26}O_2ClN_4$, 413.1739; Found: 413.1745.

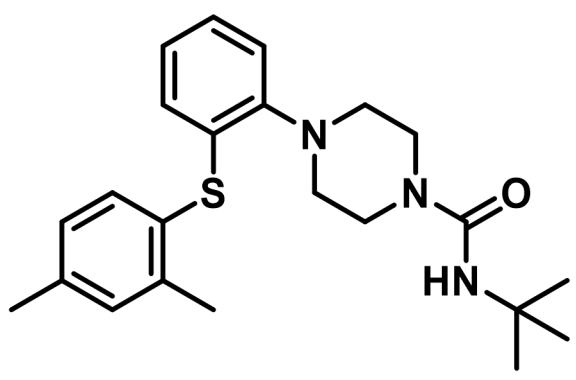

N-(tert-butyl)-4-(2-((2,4-dimethylphenyl)thio)phenyl)piperazine-1-carboxamide **(P72)**

Compound P72 was prepared following the general procedure D_2 (flash chromatography for purification: petroleum ether/ethyl acetate = 1:1−1:5) in 83% yield (99.0 mg) as a white solid, mp: 139.6-141.6 ℃.

Reaction time: 6 h

^{1}H NMR (400 MHz, $CDCl_3$) δ 7.36 (d, J = 7.8 Hz, 1H), 7.14 (s, 1H), 7.09 – 6.99 (m, 3H), 6.89 – 6.83 (m, 1H), 6.55 – 6.50 (m, 1H), 4.47 (s, 1H), 3.57 – 3.45 (m, 4H), 3.12 – 2.97 (m, 4H), 2.34 (s, 3H), 2.31 (s, 3H), 1.38 (s, 9H).

^{13}C NMR (101 MHz, $CDCl_3$) δ 157.2, 148.9, 142.4, 139.3, 136.2, 134.6, 131.8, 127.9, 127.8, 126.3, 125.6, 124.7, 119.9, 51.5, 50.8, 44.3, 29.6, 21.3, 20.7.

The analytical data are in accordance with reported literature.[13]

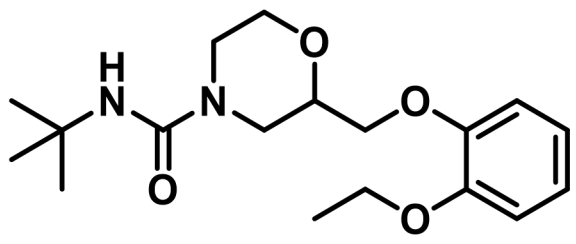

N-(tert-butyl)-2-((2-ethoxyphenoxy)methyl)morpholine-4-carboxamide **(P73)**

Compound P73 was prepared following the general procedure D_2 (flash chromatography for purification: petroleum ether/ethyl acetate = 1:1−1:5) in 84% yield (84.8 mg) as a colorless oil.

Reaction time: 6 h

^{1}H NMR (400 MHz, $CDCl_3$) δ 6.95 – 6.80 (m, 4H), 4.44 (s, 1H), 4.10 – 3.98 (m, 3H), 3.98 – 3.87 (m, 2H), 3.85 – 3.76 (m, 2H), 3.76 – 3.67 (m, 1H), 3.61 – 3.52 (m, 1H), 2.97 – 2.78 (m, 2H), 1.40 (t, J = 7.0 Hz, 3H), 1.31 (s, 9H).

^{13}C NMR (101 MHz, $CDCl_3$) δ 156.9, 149.2, 148.2, 122.2, 121.0, 115.3, 113.7, 73.5, 70.1, 66.4, 64.4, 50.8, 46.5, 43.3, 29.4, 15.0.

HRMS（ESI, Q-TOF）m/z: $[M+H]^+$ Calcd for $C_{18}H_{29}O_4N_2$, 337.2122; Found: 337.2125.

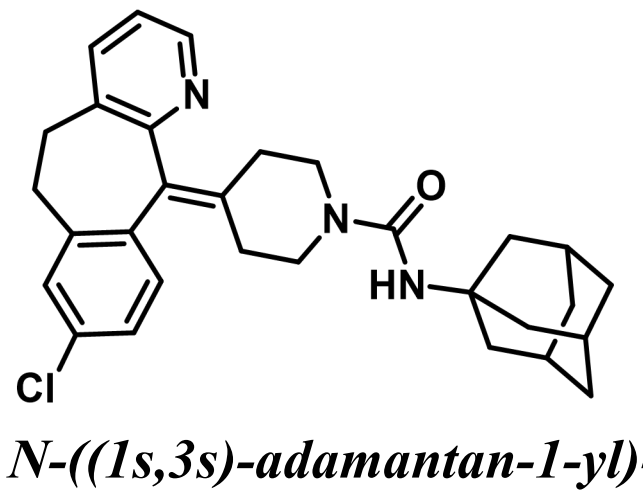

N-((1s,3s)-adamantan-1-yl)-4-(8-chloro-5,6-dihydro-11H-benzo[5,6]cyclohepta[1,2-b]pyridin-11-ylidene)piperidine-1-carboxamide **(P74)**

Compound P74 was prepared following the general procedure D_2 (flash chromatography for purification: petroleum ether/ethyl acetate = 1:1−1:5) in 69% yield (101.1 mg) as a white solid, mp: 224.5-225.5 ℃.

Reaction time: 6 h

^{1}H NMR (400 MHz, $CDCl_3$) δ 8.36 (d, J = 6.3 Hz, 1H), 7.40 (d, J = 9.1 Hz, 1H), 7.13 (s, 1H), 7.08 (d, J = 8.8 Hz, 2H), 7.08 – 7.02 (m, 1H), 4.19 (s, 1H), 3.65 – 3.55 (m, 2H), 3.41 – 3.26 (m,

2H), 3.09 – 2.98 (m, 2H), 2.86 – 2.69 (m, 2H), 2.58 – 2.46 (m, 1H), 2.41 – 2.25 (m, 3H), 2.02 (d, *J* = 4.3 Hz, 3H), 1.94 (d, *J* = 2.5 Hz, 6H), 1.62 (s, 6H).

^{13}C NMR (101 MHz, $CDCl_3$) δ 157.1, 156.5, 146.7, 139.6, 137.73, 137.67, 137.5, 134.0, 133.4, 132.9, 130.6, 129.0, 126.2, 122.3, 51.3, 44.8, 44.7, 42.4, 36.5, 31.7, 31.5, 30.5, 30.3, 29.6.

HRMS（ESI, Q-TOF）m/z: $[M+H]^+$ Calcd for $C_{30}H_{35}OClN_3$, 488.2463; Found: 488.2470.

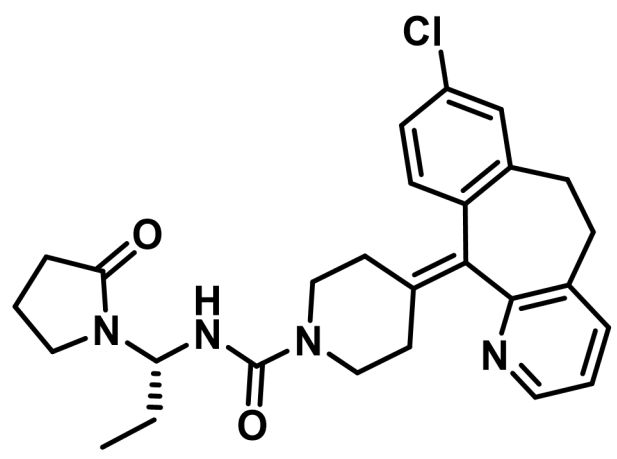

(S)-4-(8-chloro-5,6-dihydro-11H-benzo[5,6]cyclohepta[1,2-b]pyridin-11-ylidene)-N-(1-(2-oxopyrrolidin-1-yl)propyl)piperidine-1-carboxamide **(P75)**

Compound P75 was prepared following the general procedure D_2 (flash chromatography for purification: petroleum ether/ethyl acetate = 1:1−1:5) in 78% yield (112.1 mg) as a white solid, mp: 86.6-88.5 °C.

Reaction time: 6 h

^{1}H NMR (400 MHz, $CDCl_3$) δ 8.39 (d, *J* = 3.6 Hz, 1H), 7.43 (d, *J* = 7.5 Hz, 1H), 7.18 – 7.05 (m, 4H), 6.10 (d, *J* = 9.1 Hz, 1H), 5.06 – 4.92 (m, 1H), 3.75 – 3.58 (m, 3H), 3.48 – 3.27 (m, 3H), 3.17 – 3.06 (m, 2H), 2.89 – 2.72 (m, 2H), 2.58 – 2.49 (m, 1H), 2.42 – 2.26 (m, 5H), 2.04 – 1.97 (m, 2H), 1.92 – 1.82 (m, 2H), 0.92 (t, *J* = 7.4 Hz, 3H).

^{13}C NMR (101 MHz, $CDCl_3$) δ 176.1, 157.1, 156.9, 146.8, 139.7, 137.72, 137.67, 137.3, 134.4, 133.4, 133.1, 130.7, 129.1, 126.3, 122.4, 64.9, 49.1, 49.0, 44.7, 32.3, 31.8, 31.6, 30.5, 30.4, 26.1, 18.8, 10.6.

HRMS（ESI, Q-TOF）m/z: $[M+Na]^+$ Calcd for $NaC_{27}H_{31}O_2ClN_4$, 501.2028; Found: 501.2033.

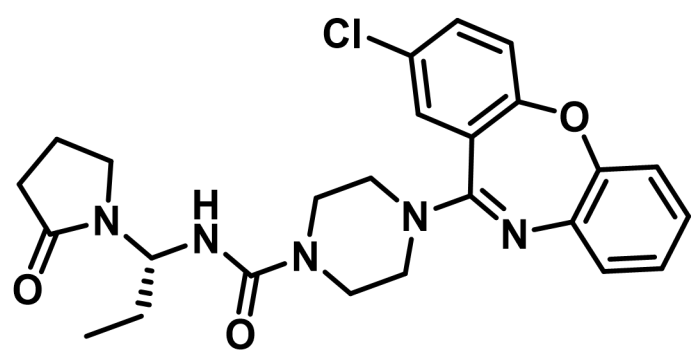

(S)-4-(2-chlorodibenzo[b,f][1,4]oxazepin-11-yl)-N-(1-(2-oxopyrrolidin-1-yl)propyl)piperazine-1-carboxamide **(P76)**

Compound P76 was prepared following the general procedure D_2 (flash chromatography for purification: petroleum ether/ethyl acetate = 1:1−1:5) in 81% yield (117.12 mg) as a white solid, mp: 129.8-131.2 °C.

Reaction time: 10 h

^{1}H NMR (400 MHz, $CDCl_3$) δ 7.40 – 7.34 (m, 1H), 7.28 (d, *J* = 2.6 Hz, 1H), 7.19 – 7.04 (m, 4H), 7.01 – 6.95 (m, 1H), 6.23 (d, *J* = 9.0 Hz, 1H), 5.02 (dd, *J* = 16.4, 7.7 Hz, 1H), 3.68 – 3.54 (m, 2H), 3.54 – 3.38 (m, 8H), 2.35 – 2.29 (m, 2H), 2.05 – 1.81 (m, 4H), 0.93 (t, *J* = 7.4 Hz, 3H).

^{13}C NMR (101 MHz, $CDCl_3$) δ 176.1, 159.4, 158.8, 157.0, 151.8, 139.9, 132.8, 130.4, 128.9, 127.2, 125.9, 124.89, 124.85, 122.8, 120.2, 64.7, 48.8, 47.2, 43.5, 32.2, 26.0, 18.7, 10.5.

HRMS（ESI, Q-TOF）m/z: $[M+H]^+$ Calcd for $C_{25}H_{29}O_3ClN_5$, 482.1953; Found: 482.1960.

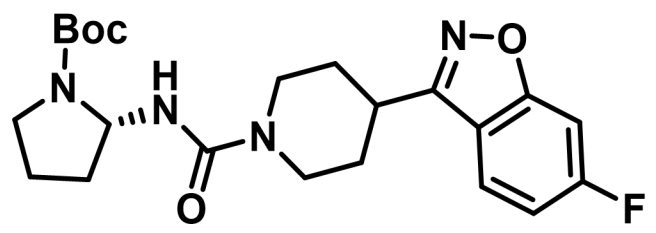

***tert-butyl (S)-2-(4-(6-fluorobenzo[d]isoxazol-3-yl)piperidine-1-carboxamido)pyrrolidine-1-carboxylate* (P77)**

Compound P77 was prepared following the general procedure D_2 (flash chromatography for purification: petroleum ether/ethyl acetate = 1:1−1:5) in 91% yield (118.1 mg) as a white solid, mp: 109.8-111.3 °C.

Reaction time: 6 h

^{1}H NMR (400 MHz, $CDCl_3$) δ 7.60 (s, 1H), 7.17 (d, *J* = 8.4 Hz, 1H), 7.05 – 6.94 (m, 1H), 5.46 (d, *J* = 36.0 Hz, 1H), 4.95 (d, *J* = 47.1 Hz, 1H), 4.08 – 3.95 (m, 2H), 3.45 – 3.34 (m, 1H), 3.29 – 3.13 (m, 2H), 2.94 (t, *J* = 12.1 Hz, 2H), 2.11 – 1.75 (m, 8H), 1.38 (s, 9H).

^{13}C NMR (101 MHz, $CDCl_3$) δ 164.1 (d, *J* = 250.8 Hz), 163.9, 163.7, 160.4, 154.2 (d, *J* = 3.3 Hz), 122.3, 117.1, 112.5 (d, *J* = 25.3 Hz), 97.4 (d, *J* = 26.7 Hz), 79.9, 77.4, 60.4, 43.8, 34.3, 30.0, 28.4, 21.0, 14.2.

^{19}F NMR (377 MHz, $CDCl_3$) δ -109.2 (s, 1F).

HRMS（ESI, Q-TOF）m/z: $[M+Na]^+$ Calcd for $NaC_{22}H_{29}O_4FN_4$, 455.2065; Found: 455.2073.

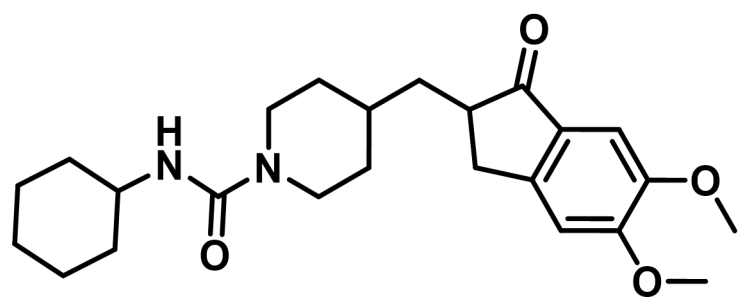

***N-cyclohexyl-4-((5,6-dimethoxy-1-oxo-2,3-dihydro-1H-inden-2-yl)methyl)piperidine-1-carboxamide* (P78)**

Compound P78 was prepared following the general procedure D_2 (flash chromatography for purification: petroleum ether/ethyl acetate = 10:1−2:1) in 78% yield (97.0 mg) as a white solid, mp: 138.4-139.7 °C.

Reaction time: 6 h

^{1}H NMR (400 MHz, $CDCl_3$) δ 7.14 (s, 1H), 6.84 (s, 1H), 4.32 (d, *J* = 7.5 Hz, 1H), 3.94 (s, 3H), 3.88 (s, 3H), 3.66 – 3.56 (m, 1H), 3.31 – 3.17 (m, 1H), 2.81 – 2.64 (m, 4H), 1.94 – 1.84 (m, 3H), 1.77 – 1.52 (m, 7H), 1.38 – 1.28 (m, 3H), 1.25 – 0.98 (m, 6H).

^{13}C NMR (101 MHz, $CDCl_3$) δ 207.7, 157.1, 155.6, 149.5, 148.8, 129.3, 107.4, 104.4, 56.3, 56.2, 49.5, 45.2, 44.3, 38.7, 34.6, 34.1, 33.4, 32.6, 31.6, 25.8, 25.2.

HRMS（ESI, Q-TOF）m/z: $[M+H]^+$ Calcd for $C_{24}H_{35}O_4N_2$, 415.2591; Found: 415.2597.

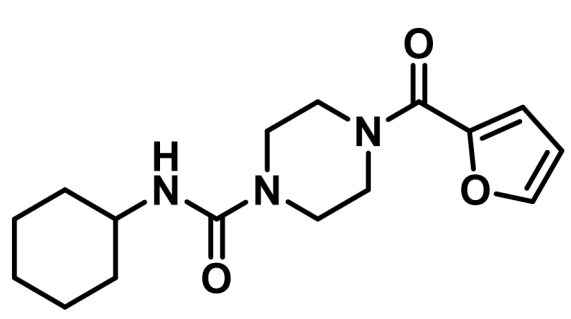

***N-cyclohexyl-4-(furan-2-carbonyl)piperazine-1-carboxamide* (P79)**

Compound P79 was prepared following the general procedure D_2 (flash chromatography for purification: petroleum ether/ethyl acetate = 10:1−2:1) in 79% yield, mp: 145.5-146.9 °C.

Reaction time: 6 h

^{1}H NMR (400 MHz, $CDCl_3$) δ 7.44 (d, *J* = 0.9 Hz, 1H), 6.96 (d, *J* = 3.5 Hz, 1H), 6.46 – 6.41 (m, 1H), 4.60 (d, *J* = 7.2 Hz, 1H), 3.75 (s, 4H), 3.65 – 3.52 (m, 1H), 3.48 – 3.36 (m, 4H), 1.95 – 1.82 (m, 2H), 1.71 – 1.60 (m, 2H), 1.59 – 1.49 (m, 1H), 1.35 – 1.22 (m, 2H), 1.13 – 0.99 (m, 3H).
^{13}C NMR (101 MHz, $CDCl_3$) δ 159.2, 156.9, 147.6, 144.0, 116.8, 111.4, 49.6, 43.6, 33.8, 25.6, 25.1.
HRMS（ESI, Q-TOF）m/z: $[M+Na]^+$ Calcd for $NaC_{16}H_{23}O_3N_3$, 328.1632; Found: 328.1636.

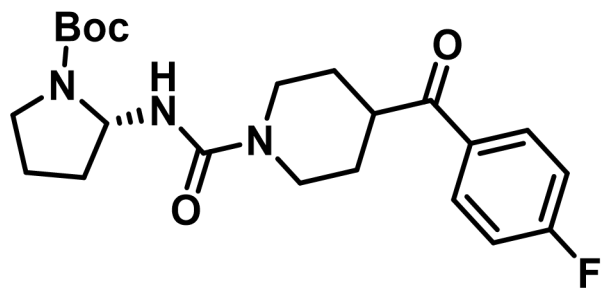

tert-butyl (S)-2-(4-(4-fluorobenzoyl)piperidine-1-carboxamido)pyrrolidine-1-carboxylate **(P80)**
Compound P80 was prepared following the general procedure D_2 (flash chromatography for purification: petroleum ether/ethyl acetate = 1:1−1:5) in 92% yield (115.8 mg) as a white solid, mp: 165.5-167.3 ℃.
Reaction time: 6 h
^{1}H NMR (400 MHz, $CDCl_3$) δ 7.94 – 7.82 (m, 2H), 7.05 (t, *J* = 8.5 Hz, 2H), 5.41 (d, *J* = 52.1 Hz, 1H), 4.98 (d, *J* = 5.0 Hz, 1H), 3.93 (s, 2H), 3.43 – 3.26 (m, 2H), 3.22 – 3.13 (m, 1H), 2.86 (t, *J* = 11.7 Hz, 2H), 2.03 – 1.72 (m, 6H), 1.67 – 1.53 (m, 2H), 1.36 (s, 9H).
^{13}C NMR (101 MHz, $CDCl_3$) δ 200.3, 165.6 (d, *J* = 255.1 Hz), 156.3, 154.2, 132.0 (d, *J* = 2.9 Hz), 130.8 (d, *J* = 9.3 Hz), 115.8 (d, *J* = 21.8 Hz), 79.8, 65.9, 46.0, 43.3, 43.2, 34.2, 28.4, 28.2, 22.2.
^{19}F NMR (377 MHz, $CDCl_3$) δ -104.9 (s, 1F).
HRMS（ESI, Q-TOF）m/z: $[M+Na]^+$ Calcd for $NaC_{22}H_{30}O_4FN_3$, 442.2113; Found: 442.2121.

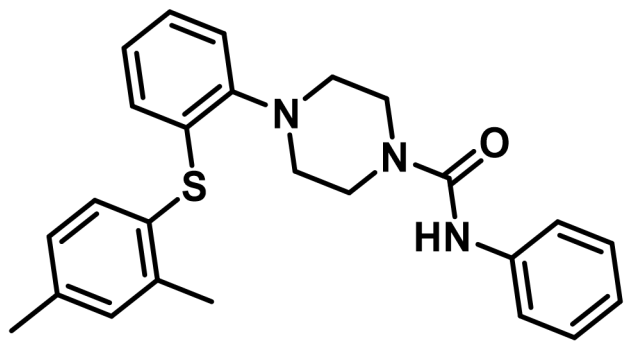

4-(2-((2,4-dimethylphenyl)thio)phenyl)-N-phenylpiperazine-1-carboxamide **(P81)**
Compound P81 was prepared following the general procedure D_2 (flash chromatography for purification: petroleum ether/ethyl acetate = 1:1−1:5) in 37% yield (46.4 mg) as a white solid, mp: 186.2-188.0 ℃.
Reaction time: 7 h
^{1}H NMR (400 MHz, $CDCl_3$) δ 7.42 – 7.35 (m, 3H), 7.30 (t, *J* = 7.9 Hz, 2H), 7.16 (s, 1H), 7.12 – 7.01 (m, 4H), 6.94 – 6.87 (m, 1H), 6.57 – 6.53 (m, 1H), 6.51 (s, 1H), 3.76 – 3.62 (m, 4H), 3.18 – 3.06 (m, 4H), 2.37 (s, 3H), 2.33 (s, 3H).
^{13}C NMR (101 MHz, $CDCl_3$) δ 155.2, 148.7, 142.5, 139.5, 139.1, 136.2, 134.7, 131.9, 129.0, 128.0, 127.8, 126.5, 125.7, 124.9, 123.3, 120.1, 120.0, 51.6, 44.7, 21.3, 20.7.
HRMS（ESI, Q-TOF）m/z: $[M+H]^+$ Calcd for $C_{25}H_{28}ON_3S$, 418.1948; Found: 418.1950.

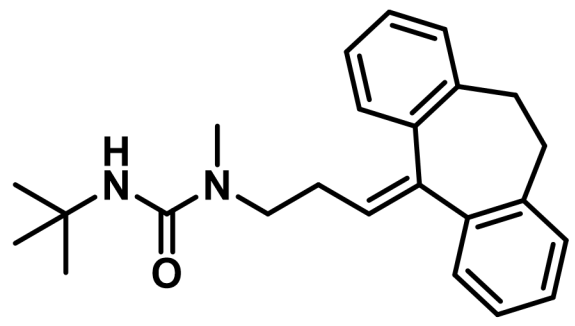

***3-(tert-butyl)-1-(3-(10,11-dihydro-5H-dibenzo[a,d][7]annulen-5-ylidene)propyl)-1-methylurea* (P82)**

Compound P82 (amine substrate: Nortriptyline hydrochloride) was prepared following the general procedure D_2 (flash chromatography for purification: petroleum ether/ethyl acetate = 10:1−2:1) in 76% yield (82.7 mg) as a colourless oil.

Reaction time: 6 h

^{1}H NMR (400 MHz, $CDCl_3$) δ 7.32 – 7.27 (m, 1H), 7.23 – 7.15 (m, 3H), 7.15 – 7.10 (m, 3H), 7.06 – 7.01 (m, 1H), 5.86 (t, *J* = 7.6 Hz, 1H), 4.15 (s, 1H), 3.45 – 3.20 (m, 4H), 3.04 – 2.89 (m, 1H), 2.85 – 2.76 (m, 1H), 2.74 (s, 3H), 2.35 (q, *J* = 7.3 Hz, 2H), 1.30 (s, 9H).

^{13}C NMR (101 MHz, $CDCl_3$) δ 157.2, 144.6, 141.0, 139.9, 139.4, 137.0, 130.1, 128.7, 128.2, 128.1, 127.6, 127.2, 126.1, 125.9, 50.7, 48.6, 34.4, 33.8, 32.1, 29.5, 28.4.

The analytical data are in accordance with reported literature.[13]

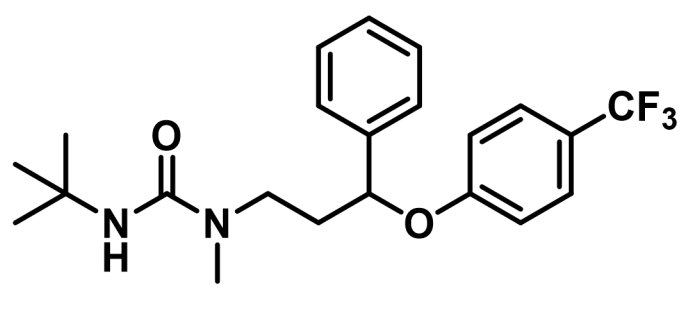

***3-(tert-butyl)-1-methyl-1-(3-phenyl-3-(4-(trifluoromethyl)phenoxy)propyl)urea* (P83)**

Compound P83 was prepared following the general procedure D_2 (flash chromatography for purification: petroleum ether/ethyl acetate = 10:1−2:1) in 45% yield (55.2 mg) as a colorless oil.

Reaction time: 6 h

^{1}H NMR (400 MHz, $CDCl_3$) δ 7.34 (d, *J* = 8.6 Hz, 2H), 7.24 (d, *J* = 4.3 Hz, 4H), 7.18 (t, *J* = 4.3 Hz, 1H), 6.81 (d, *J* = 8.6 Hz, 2H), 5.13 (dd, *J* = 8.6, 4.0 Hz, 1H), 4.16 (s, 1H), 3.47 – 3.26 (m, 2H), 2.76 (s, 3H), 2.19 – 1.98 (m, 2H), 1.16 (s, 9H).

^{13}C NMR (101 MHz, $CDCl_3$) δ 160.3, 157.4, 140.7, 129.0, 128.0, 126.9 (q, *J* = 3.7 Hz), 125.8, 124.4 (q, *J* = 271.1 Hz), 123.1 (q, *J* = 32.9 Hz), 115.9, 77.8, 50.7, 45.7, 37.2, 34.5, 29.5.

^{19}F NMR (377 MHz, $CDCl_3$) δ -61.6 (s, 3F).

The analytical data are in accordance with reported literature.[13]

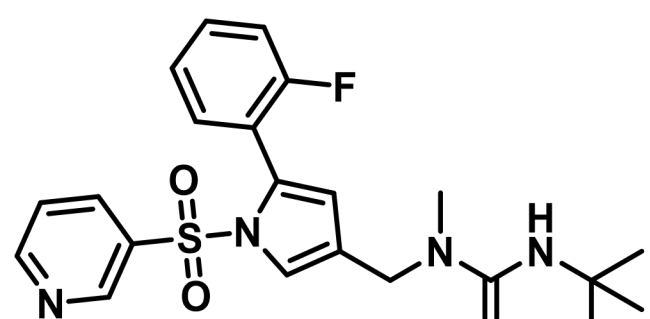

***3-(tert-butyl)-1-((5-(2-fluorophenyl)-1-(pyridin-3-ylsulfonyl)-1H-pyrrol-3-yl)methyl)-1-methylurea* (P84)**

Compound P84 was prepared following the general procedure D_2 (flash chromatography for purification: petroleum ether/ethyl acetate = 10:1−2:1) in 55% yield (73.4 mg) as a yellow oil.

Reaction time: 6 h

^{1}H NMR (400 MHz, $CDCl_3$) δ 8.74 (d, *J* = 6.3 Hz, 1H), 8.57 (s, 1H), 7.70 – 7.64 (m, 1H), 7.43 – 7.35 (m, 1H), 7.31 (t, *J* = 6.4 Hz, 2H), 7.15 – 7.09 (m, 2H), 7.02 (t, *J* = 8.9 Hz, 1H), 6.20 (s, 1H), 4.28 (s, 1H), 4.28 (s, 2H), 2.80 (s, 3H), 1.31 (s, 9H).
^{13}C NMR (101 MHz, $CDCl_3$) δ 160.8 (d, *J* = 249.3 Hz), 157.4, 154.3, 147.9, 135.2, 134.7, 133.2, 131.3 (d, *J* = 8.1 Hz), 129.4, 126.0, 123.6, 123.5 (d, *J* = 3.6 Hz), 121.3, 119.0 (d, *J* = 15.4 Hz), 117.8, 115.4 (d, *J* = 21.9 Hz), 50.9, 44.8, 34.4, 29.5.
^{19}F NMR (377 MHz, $CDCl_3$) δ -111.2 (s, 1F).
The analytical data are in accordance with reported literature.[13]

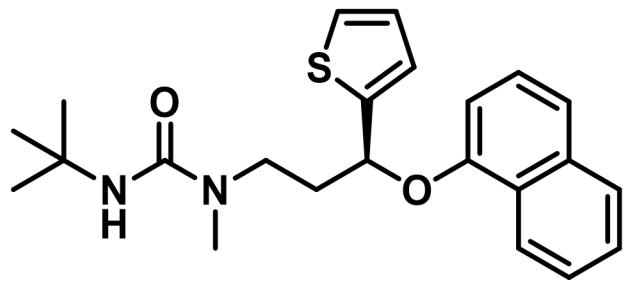

(S)-3-(tert-butyl)-1-methyl-1-(3-(naphthalen-1-yloxy)-3-(thiophen-2-yl)propyl)urea **(P85)**
Compound P85 (amine substrate: Duloxetine hydrochloride) was prepared following the general procedure D_2 (flash chromatography for purification: petroleum ether/ethyl acetate = 10:1−2:1) in 45% yield (53.5 mg) as a yellow oil.
Reaction time: 6 h
^{1}H NMR (400 MHz, $CDCl_3$) δ 8.38 – 8.32 (m, 1H), 7.81 – 7.75 (m, 1H), 7.53 – 7.46 (m, 2H), 7.40 (d, *J* = 8.2 Hz, 1H), 7.27 (s, 1H), 7.19 (d, *J* = 5.0 Hz, 1H), 7.07 (d, *J* = 3.4 Hz, 1H), 6.95 – 6.90 (m, 1H), 6.84 (d, *J* = 7.7 Hz, 1H), 5.76 – 5.69 (m, 1H), 4.24 (s, 1H), 3.58 – 3.42 (m, 2H), 2.86 (s, 3H), 2.51 – 2.26 (m, 2H), 1.15 (s, 9H).
^{13}C NMR (101 MHz, $CDCl_3$) δ 157.5, 152.9, 144.7, 134.7, 127.7, 126.8, 126.5, 126.2, 125.8, 125.4, 125.04, 124.96, 122.0, 121.0, 107.3, 73.6, 50.7, 45.8, 37.5, 34.5, 29.3.
The analytical data are in accordance with reported literature.[13]

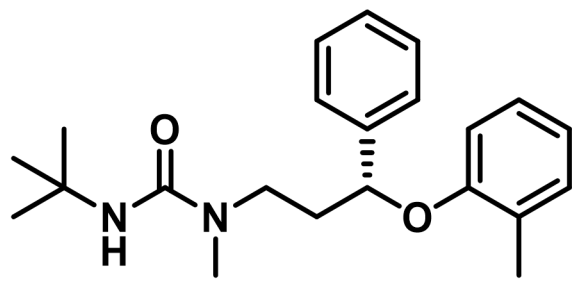

N-(tert-butyl)-3,9-dimethyl-2,3-dihydrobenzo[f][1,4]oxazepine-4(5H)-carboxamide **(P86)**
Compound P86 (amine substrate: Atomoxetine hydrochloride) was prepared following the general procedure D_2 (flash chromatography for purification: petroleum ether/ethyl acetate = 10:1−2:1) in 42% yield (44.7 mg) as a white solid, mp: 93.0-94.6 °C.
Reaction time: 6 h
^{1}H NMR (400 MHz, $CDCl_3$) δ 7.30 (t, *J* = 6.3 Hz, 4H), 7.26 – 7.20 (m, 1H), 7.11 (d, *J* = 7.3 Hz, 1H), 6.95 (t, *J* = 7.3 Hz, 1H), 6.77 (t, *J* = 7.3 Hz, 1H), 6.59 (d, *J* = 8.2 Hz, 1H), 5.25 – 5.16 (m, 1H), 4.27 (s, 1H), 3.57 – 3.29 (m, 2H), 2.85 (s, 3H), 2.34 (s, 3H), 2.18 – 2.07 (m, 2H), 1.20 (s, 9H).
^{13}C NMR (101 MHz, $CDCl_3$) δ 157.6, 155.4, 141.6, 130.8, 128.8, 127.7, 126.9, 126.8, 125.8, 120.6, 113.0, 76.6, 50.6, 46.0, 37.3, 34.3, 29.4, 16.7.
The analytical data are in accordance with reported literature.[13]

4. Gram-scale reaction of P72

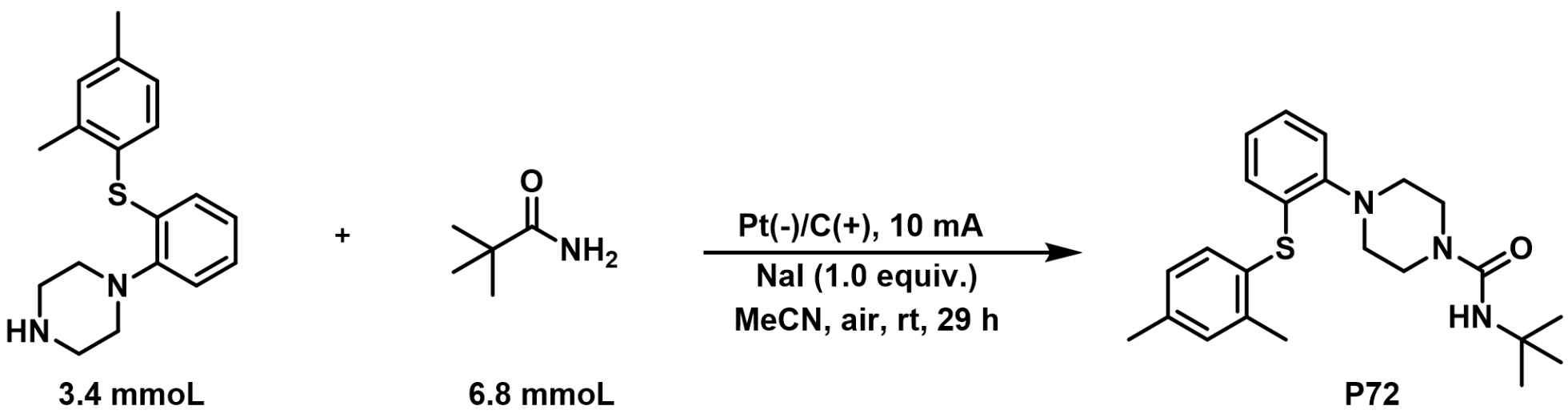

Setup shown in Figure S2: To a 100 mL three-necked flask equipped with a stir bar was added Vortioxetine (1.02 g, 3.4 mmol, 1.0 equiv.), 2,2-dimethylpropanamide (687.8 mg, 6.8 mmol, 2.0 equiv.), NaI (509.6 mg, 3.4 mmol, 1.0 equiv.), and MeCN (40 mL). No need to change of air, a graphite rod was used as the anode, and a platinum plate was used as the cathode, the mixture was stirred at a constant current of 10 mA at room temperature until complete consumption of the Vortioxetine (monitored by TLC). The reaction lasted 30 hours, the vacuum concentrated reaction solution was extracted with saturated $NaHSO_3$ solution and EtOAc. The aqueous layer was separated and extracted with EtOAc. The combined organic layer was washed with brine, dried over Na_2SO_4, filtered, and concentrated *in vacuo*. The resulting residue was purified by chromatography on silica gel to give the desired product **P72** (987 mg, 73% yield).

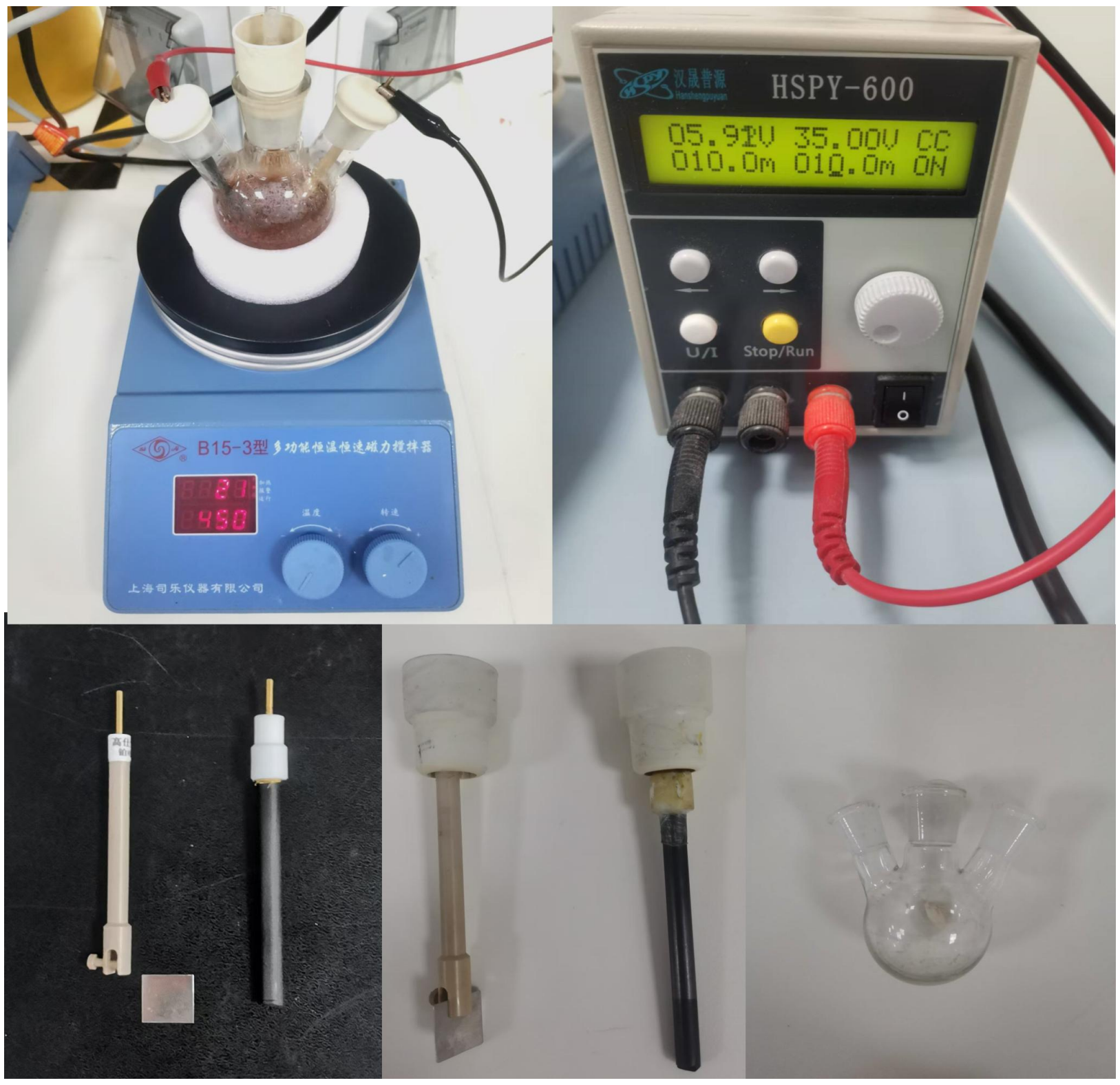

Figure S2. Details of the gram-scale reaction equipment.

5. Electrochemical continuous-flow experiment

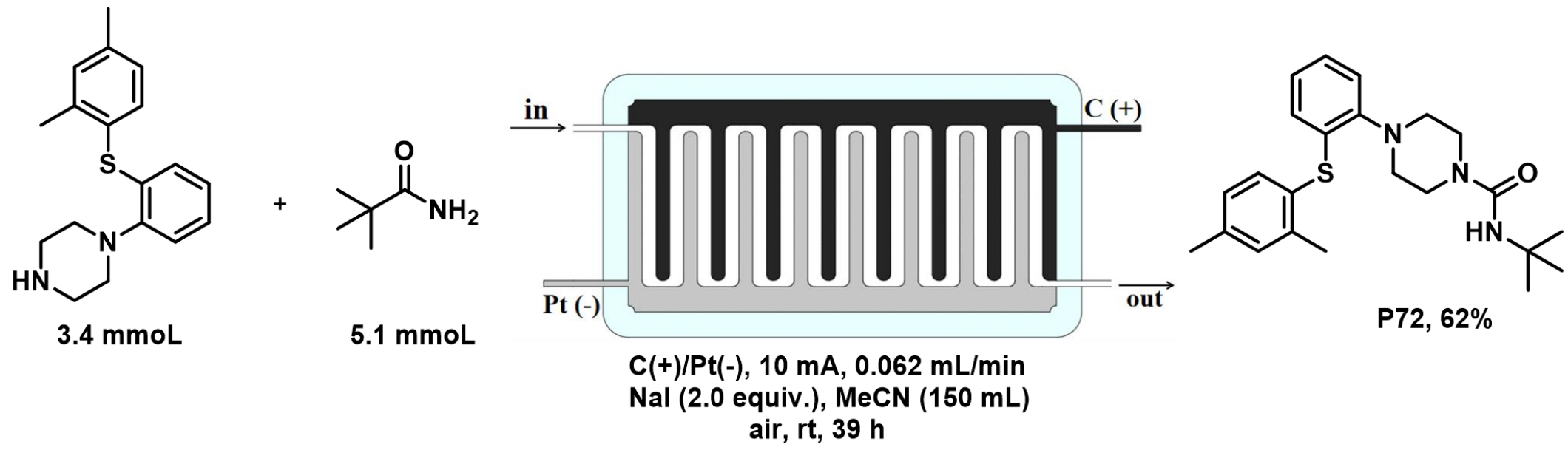

First, assembled and established the flow electrochemistry device (**Figure S3**). Then, to a 200 mL bottle was added Vortioxetine (1.02 g, 3.4 mmol, 1.0 equiv.), 2,2-dimethylpropanamide (515.9 mg, 5.1 mmol, 1.5 equiv.), NaI (1.02 g, 6.8 mmol, 2.0 equiv.), and MeCN (150 mL), the mixture was swirled until homogeneous. The reaction mixture was delivered into the flow cell via a peristaltic pump and electrolyzed at a constant current of 10 mA at room temperature. The flow rate was 0.065 mL/min until the complete electrolysis of the mixture is completed. The vacuum concentrated reaction solution was extracted with saturated $NaHSO_3$ solution and EtOAc. The aqueous layer was separated and extracted with EtOAc. The combined organic layer was washed with brine, dried over Na_2SO_4, filtered, and concentrated *in vacuo*. The resulting residue was purified by chromatography on silica gel to give the desired product **P72** (838 mg, 62% yield).

The continuous-flow equipment was purchased from the EZONE (model: PER-10). The reaction mixture is added via a peristaltic pump (from the LEADFLUID BT101L). The solvent of the continuous-flow reactor is 3 mL, and the spacing between carbon and Pt electrode materials is 1 mm, the current is controlled by the HSPY-600 (**Figure S3**). Connect the line and pipeline, connect the positive electrode to carbon material, the negative electrode to Pt material, and connect the pipeline through a connector (**Figure S4**).

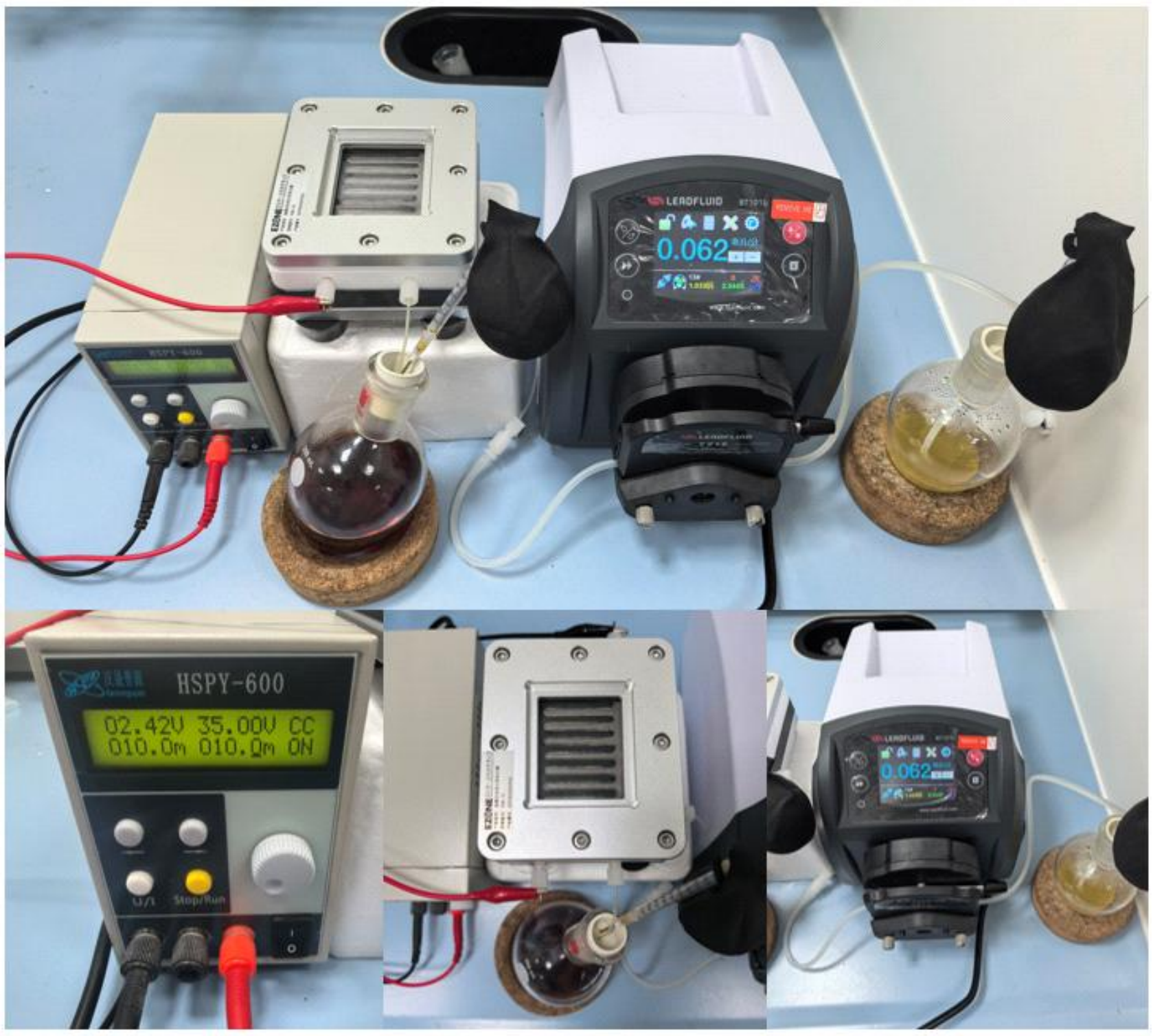

Figure S3. Details of the continuous-flow equipment.

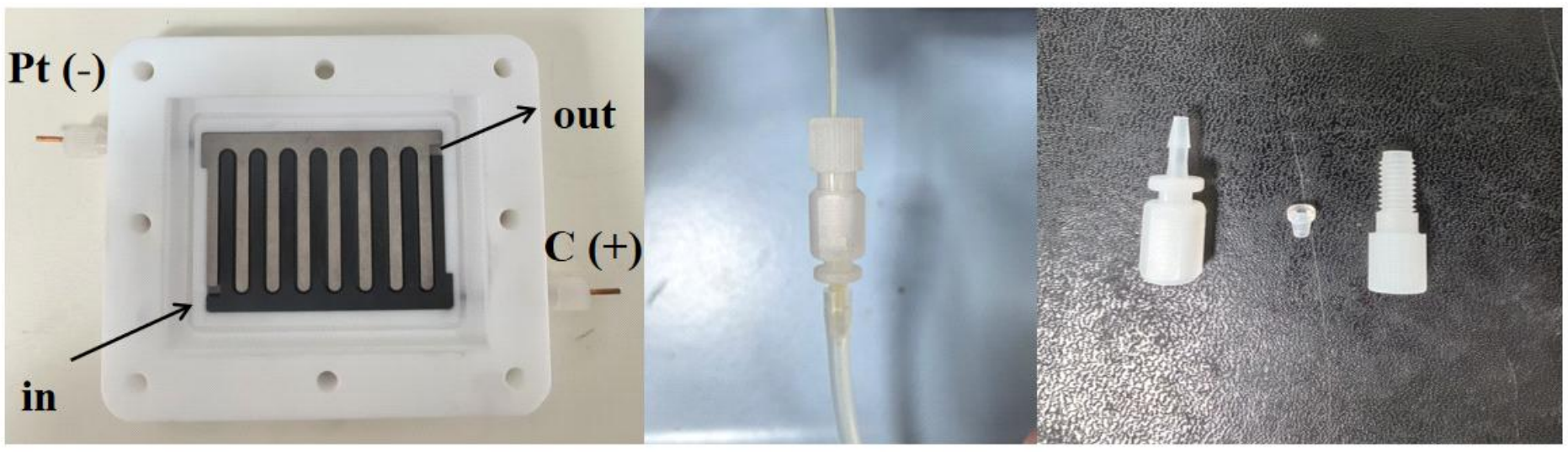

Figure S4. Details of the electrodes and pipelines.

6. Synthetic applications

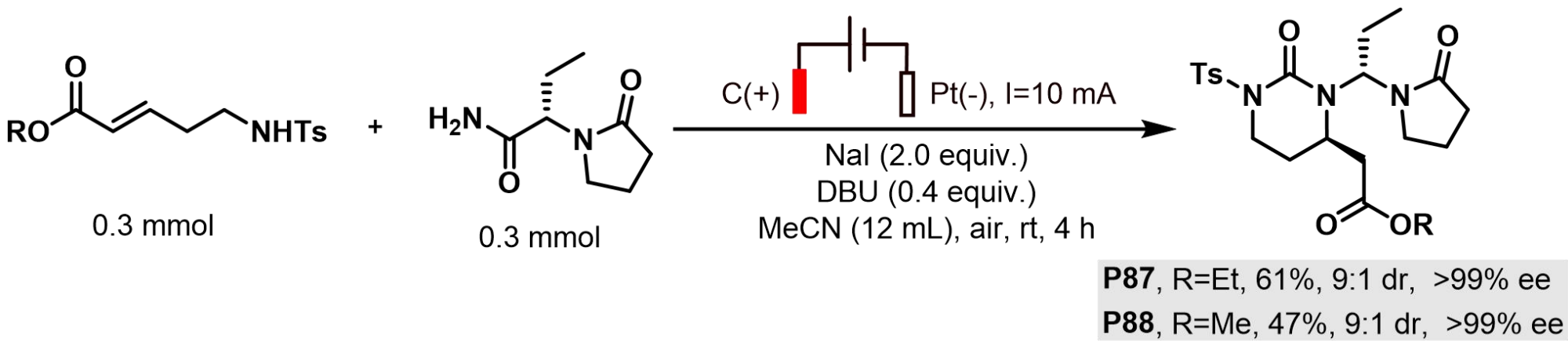

Allylamine (0.3 mmol, 1.0 equiv.), amides (0.3 mmol, 1.0 equiv.), sodium iodide (0.6 mmol, 2.0 equiv.), DBU (0.12 mmol, 0.4 equiv.), and MeCN (12 mL) were added to 25 mL three-neck flask with a teflon-coated stir bar. A graphite rod was used as the anode, and a platinum plate was used

as the cathode. The resulting mixture was stirred and electrolyzed with constant current 10 mA under air at room temperature until complete consumption of the amide (monitored by TLC). Then, transferred to a round-bottomed flask, concentrated *in vacuo*. The solutions were extracted with ethyl acetate and saturated sodium bisulfite, and the aqueous phase was extracted twice with ethyl acetate. The organic solution was dried over anhydrous sodium sulfate, filtered, and concentrated *in vacuo*, the residue was purified by flash chromatography on silica gel to obtain the products **P87** (85.2 mg, 61% yield as a yellow oil) and **P88** (63.7 mg, 47% yield as a yellow oil).

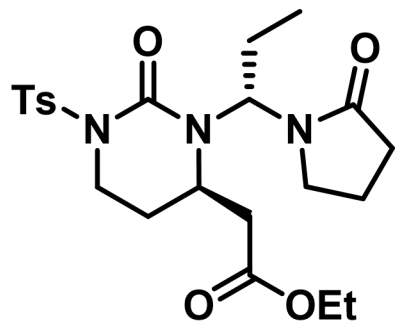

***ethyl 2-((R)-2-oxo-3-((R)-1-(2-oxopyrrolidin-1-yl)propyl)-1-tosylhexahydropyrimidin-4-yl)acetate* (P87)**

^{1}H NMR (400 MHz, $CDCl_3$) δ 7.81 (d, *J* = 7.9 Hz, 2H), 7.25 (d, *J* = 8.4 Hz, 2H), 4.97 – 4.81 (m, 1H), 4.33 – 4.21 (m, 1H), 4.17 – 4.02 (m, 2H), 4.02 – 3.88 (m, 1H), 3.82 – 3.65 (m, 1H), 3.53 – 3.28 (m, 2H), 2.68 – 2.42 (m, 2H), 2.38 (s, 3H), 2.28 – 2.03 (m, 4H), 1.98 – 1.68 (m, 4H), 1.23 (t, *J* = 7.0 Hz, 3H), 0.71 (t, *J* = 7.1 Hz, 3H).

^{13}C NMR (101 MHz, $CDCl_3$) δ 176.5, 170.0, 151.1, 144.1, 136.9, 129.2, 128.1, 70.5, 61.0, 53.2, 46.0, 41.6, 37.6, 31.2, 26.1, 24.0 21.6, 18.7, 14.1, 10.8.

HRMS（ESI, Q-TOF）m/z: $[M+Na]^+$ Calcd for $C_{22}H_{31}N_3NaO_6S$, 488.1826; Found: 488.1832.

HPLC (9:1 dr, >99% ee, AD-H, 4.6 mm × 250 mm, particle size 5 μm, hexane/isopropanol = 75/25, flow 1.0 mL/min, detection at 210 nm) retention time = 20.18 min (major) and 41.31 min (minor).

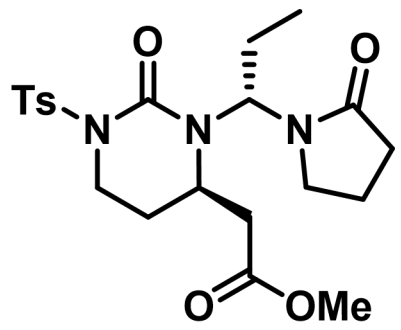

***methyl 2-((R)-2-oxo-3-((R)-1-(2-oxopyrrolidin-1-yl)propyl)-1-tosylhexahydropyrimidin-4-yl)acetate* (P88)**

^{1}H NMR (400 MHz, $CDCl_3$) δ 7.83 (d, *J* = 7.9 Hz, 2H), 7.27 (d, *J* = 8.3 Hz, 2H), 4.91 (t, *J* = 7.4 Hz, 1H), 4.30 (s, 1H), 4.05 – 3.91 (m, 1H), 3.80 – 3.70 (m, 1H), 3.68 (s, 3H), 3.53 – 3.32 (m, 2H), 2.70 – 2.55 (m, 1H), 2.56 – 2.46 (m, 1H), 2.40 (s, 3H), 2.33 – 2.20 (m, 2H), 2.11 – 1.74 (m, 6H), 0.73 (t, *J* = 7.2 Hz, 3H).

^{13}C NMR (101 MHz, $CDCl_3$) δ 176.6, 170.5, 151.2, 144.2, 137.0, 129.3, 128.2, 70.6, 53.2, 52.1, 46.2, 41.6, 37.4, 31.3, 26.2, 24.1, 21.7, 18.8, 10.9.

HRMS（ESI, Q-TOF）m/z: $[M+Na]^+$ Calcd for $C_{21}H_{29}N_3NaO_6S$, 474.1669; Found: 474.1675.

HPLC (9:1 dr, >99% ee, AD-H, 4.6 mm × 250 mm, particle size 5 μm, hexane/isopropanol = 75/25, flow 1.0 mL/min, detection at 210 nm) retention time = 18.14 min (major) and 30.82 min (minor).

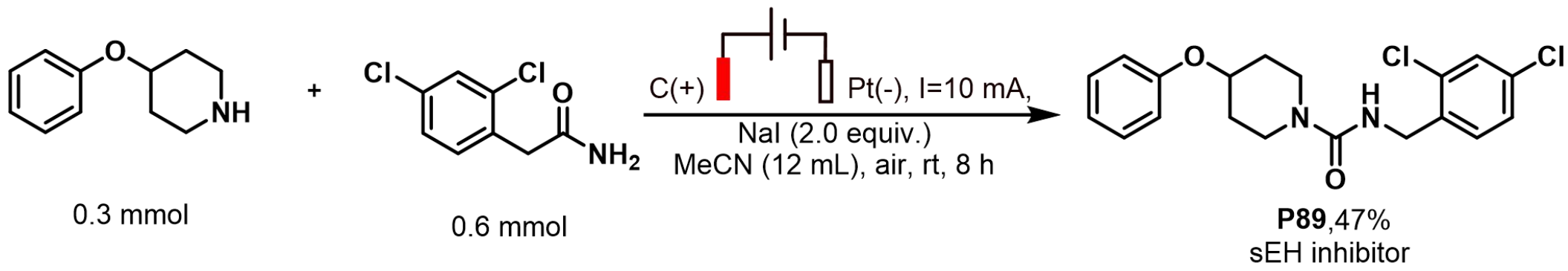

4-phenoxypiperidine (0.3 mmol, 1.0 equiv.), amides (0.6 mmol, 2.0 equiv.), sodium iodide (0.6 mmol, 2.0 equiv.), and MeCN (12 mL) were added to 25 mL three-neck flask with a teflon-coated stir bar. A graphite rod was used as the anode, and a platinum plate was used as the cathode. The resulting mixture was stirred and electrolyzed with constant current 10 mA under air at room temperature until complete consumption of the amide (monitored by TLC). Then, transferred to a round-bottomed flask, concentrated *in vacuo*. The solutions were extracted with ethyl acetate and saturated sodium bisulfite, and the aqueous phase was extracted twice with ethyl acetate. The organic solution was dried over anhydrous sodium sulfate, filtered, and concentrated *in vacuo*, the residue was purified by flash chromatography on silica gel to obtain the products **P89** (53.5 mg, 47% yield as a white solid).

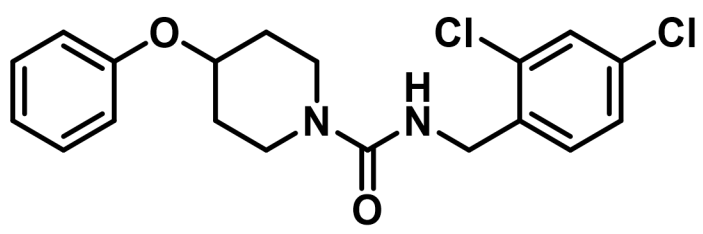

N-(2,4-dichlorobenzyl)-4-phenoxypiperidine-1-carboxamide **(P89)**

^{1}H NMR (400 MHz, $CDCl_3$) δ 7.32 (s, 2H), 7.26 – 7.20 (m, 2H), 7.17 (d, *J* = 6.6 Hz, 1H), 6.91 (t, *J* = 7.3 Hz, 1H), 6.86 (d, *J* = 7.7 Hz, 2H), 5.07 (s, 1H), 4.51 – 4.31 (m, 3H), 3.68 – 3.45 (m, 2H), 3.38 – 3.20 (m, 2H), 1.96 – 1.66 (m, 5H).

^{13}C NMR (101 MHz, $CDCl_3$) δ 157.4, 157.2, 135.7, 134.2, 133.8, 131.2, 129.7, 129.3, 127.4, 121.3, 116.2, 71.7, 42.5, 41.0, 30.4.

The analytical data are in accordance with reported literature.[13]

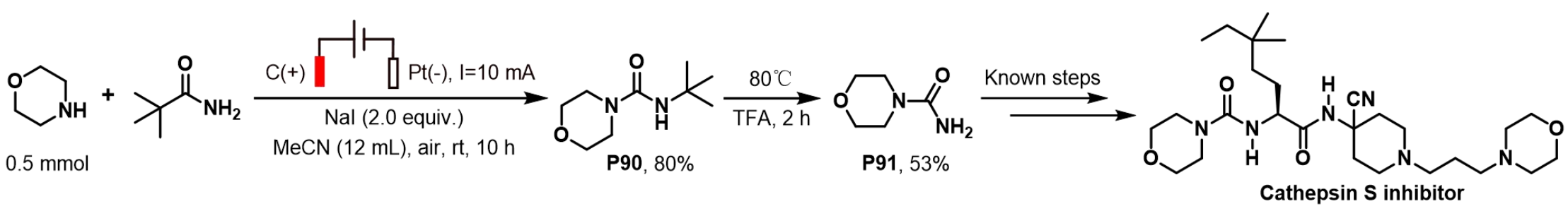

Morpholine (0.5 mmol, 1.0 equiv.), 2,2-dimethylpropanamide (1.0 mmol, 2.0 equiv.), sodium iodide (1.0 mmol, 2.0 equiv.), and MeCN (12 mL) were added to 25 mL three-neck flask with a teflon-coated stir bar. A graphite rod was used as the anode, and a platinum plate was used as the cathode. The resulting mixture was stirred and electrolyzed with constant current 10 mA under air at room temperature until complete consumption of the amide (monitored by TLC). Then, transferred to a round-bottomed flask, concentrated *in vacuo*. The solutions were extracted with

ethyl acetate and saturated sodium bisulfite, and the aqueous phase was extracted twice with ethyl acetate. The organic solution was dried over anhydrous sodium sulfate, filtered, and concentrated *in vacuo*, the residue was purified by flash chromatography on silica gel to obtain the products **P90** (74.5 mg, 80% yield as a white solid).

To an 8 mL flask equipped with a stir bar was added **P90** (0.2 mmol, 1.0 equiv.), and TFA (1 mL).[14] The mixture was stirred for 2 hours at 80℃ in air (monitored by TLC), the reaction mixture was neutralized with saturated $NaHCO_3$ to PH > 7, extracted with EtOAc, washed with brine, dried over Na_2SO_4, filtered, concentrated, the resulting residue was purified by chromatography on silica gel to give product **P91** (13.8 mg, 53% yield as a white solid).

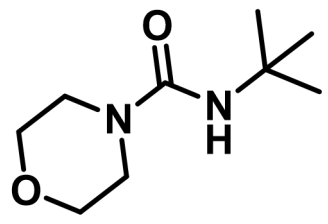

N-(tert-butyl)morpholine-4-carboxamide **(P90)**
^{1}H NMR (400 MHz, $CDCl_3$) δ 4.33 (s, 1H), 3.61 (s, 4H), 3.23 (s, 4H), 1.29 (s, 9H).
^{13}C NMR (101 MHz, $CDCl_3$) δ 157.1, 66.5, 50.8, 44.1, 29.4.
The analytical data are in accordance with reported literature.[13]

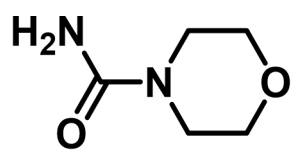

morpholine-4-carboxamide **(P91)**
^{1}H NMR (400 MHz, CD_3OD) δ 3.72 – 3.56 (m, 4H), 3.45 – 3.33 (m, 4H).
^{13}C NMR (101 MHz, CD_3OD) δ 161.1, 67.4, 45.1.
The analytical data are in accordance with reported literature.[13]

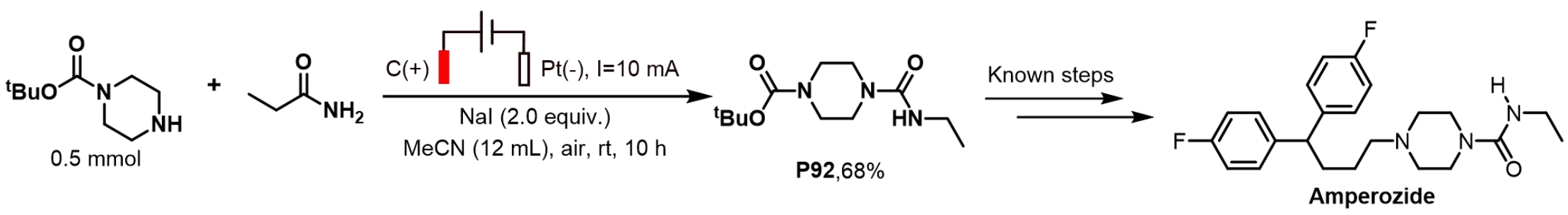

Tert-butyl piperazine-1-carboxylate (0.5 mmol, 1.0 equiv.), propionamide (1.0 mmol, 2.0 equiv.), sodium iodide (1.0 mmol, 2.0 equiv.), and MeCN (12 mL) were added to 25 mL three-neck flask with a teflon-coated stir bar. A graphite rod was used as the anode, and a platinum plate was used as the cathode. The resulting mixture was stirred and electrolyzed with constant current 10 mA under air at room temperature until complete consumption of the amide (monitored by TLC). Then, transferred to a round-bottomed flask, concentrated *in vacuo*. The solutions were extracted with ethyl acetate and saturated sodium bisulfite, and the aqueous phase was extracted twice with ethyl acetate. The organic solution was dried over anhydrous sodium sulfate, filtered,

and concentrated *in vacuo*, the residue was purified by flash chromatography on silica gel to obtain the products **P92** (87.5 mg, 68% yield as a white solid).

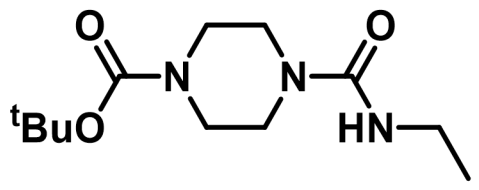

tert-butyl 4-(ethylcarbamoyl)piperazine-1-carboxylate **(P92)**

^{1}H NMR (400 MHz, $CDCl_3$) δ 4.94 (s, 1H), 3.46 – 3.22 (m, 8H), 3.21 – 3.06 (m, 2H), 1.38 (s, 9H), 1.05 (t, *J* = 7.1 Hz, 3H).

^{13}C NMR (101 MHz, $CDCl_3$) δ 157.7, 154.6, 80.1, 43.4, 35.7, 28.3, 15.5.

The analytical data are in accordance with reported literature.[13]

7. Mechanism studies

7.1 Cyclic voltammetry

Cyclic voltammetry was performed in a 50 mL glass vial fitted with a platinum column (1 mm×10 mm) working electrode, a platinum plate (10 mm×10 mm×0.3 mm) counter electrode at room temperature. The reference was an Ag/AgCl electrode submerged in saturated aqueous KCl solution. 25 mL MeCN containing 0.3 mmol ^{n}Bu$_4$NBF$_4$ were poured into the electrochemical cell in all experiments. The scan rate is 0.1 V/s, ranging from 0 V to 2.5 V.

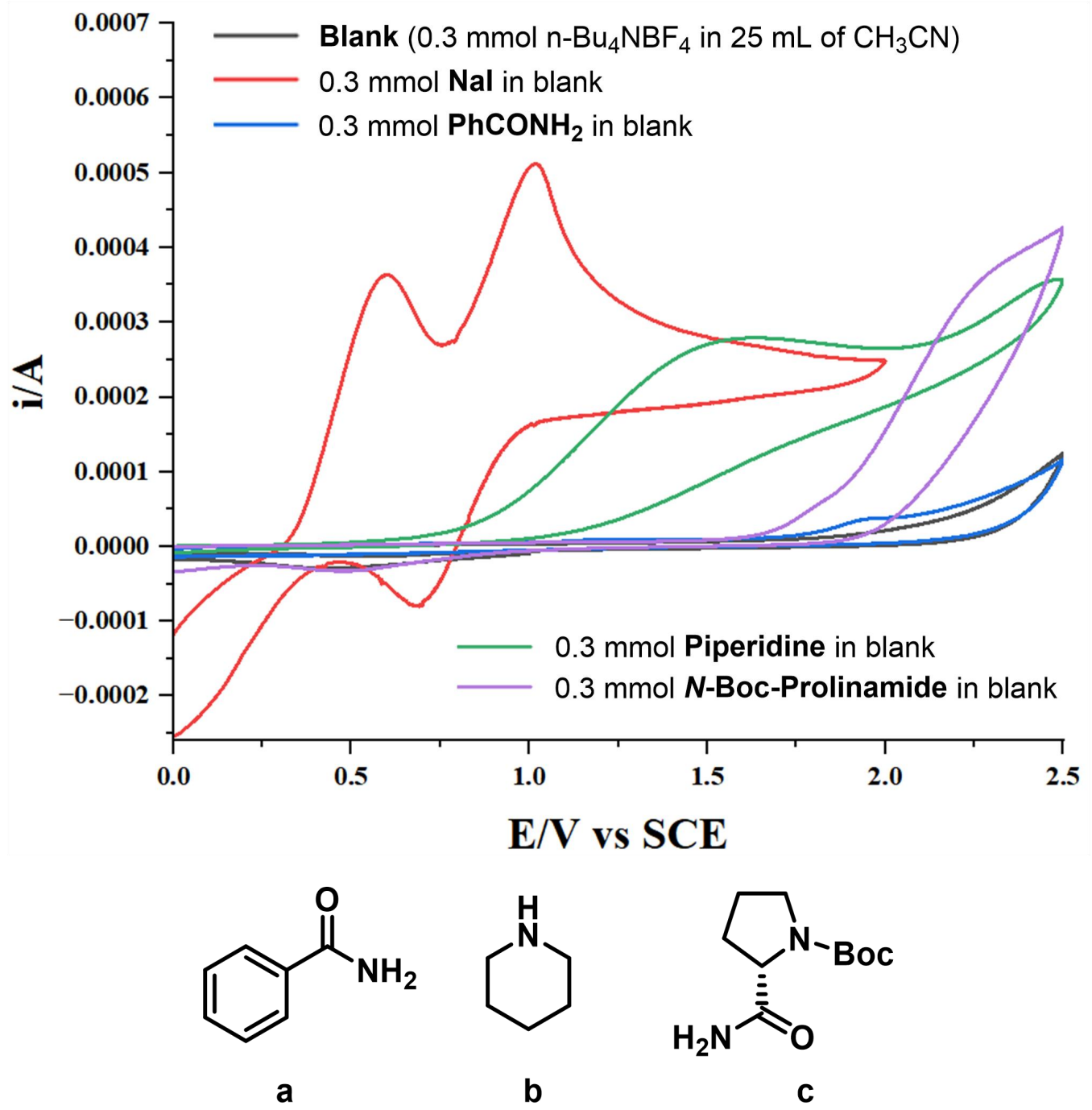

Figure S5. Cyclic voltammetry diagram.

(1). Black: Blank group: ^{n}Bu$_4$NBF$_4$ (0.3 mmol) and MeCN (25 mL) were poured into the electrochemical cell in cyclic voltammetry. The scan rate was 0.1 V/s, ranging from 0 V to 2.5 V.

(2). Red: NaI (0.3 mmol), $^{n}Bu_4NBF_4$ (0.3 mmol) and MeCN (25 mL) were poured into the electrochemical cell in cyclic voltammetry. The scan rate was 0.1 V/s, ranging from 0 V to 2.0 V.

(3). Blue: **a** (0.3 mmol), $^{n}Bu_4NBF_4$ (0.3 mmol) and MeCN (25 mL) were poured into the electrochemical cell in cyclic voltammetry. The scan rate was 0.1 V/s, ranging from 0 V to 2.5 V.

(4). Green: **b** (0.3 mmol), $^{n}Bu_4NBF_4$ (0.3 mmol) and MeCN (25 mL) were poured into the electrochemical cell in cyclic voltammetry. The scan rate was 0.1 V/s, ranging from 0 V to 2.5 V.

(5). Purple: **c** (0.3 mmol), $^{n}Bu_4NBF_4$ (0.3 mmol) and MeCN (25 mL) were poured into the electrochemical cell in cyclic voltammetry. The scan rate was 0.1 V/s, ranging from 0 V to 2.5 V.

7.2 Controlled experiments

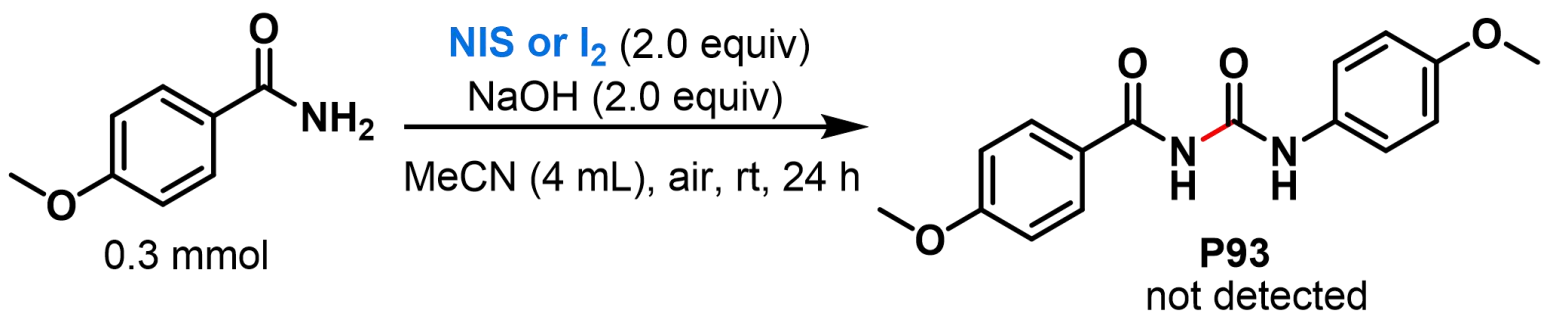

To an 8 mL flask equipped with a stir bar was added 4-methoxybenzamide (45.3 mg, 0.3 mmol, 1.0 equiv.), NaOH (24 mg, 0.6 mmol, 2.0 equiv.), additive (0.6 mmol, 2.0 equiv., NIS (135.0 mg) or I_2 (152.3 mg)), and MeCN (4 mL). The mixture was stirred for 24 hours at room temperature in air (monitored by TLC and NMR), no product was obtained (**P93**).

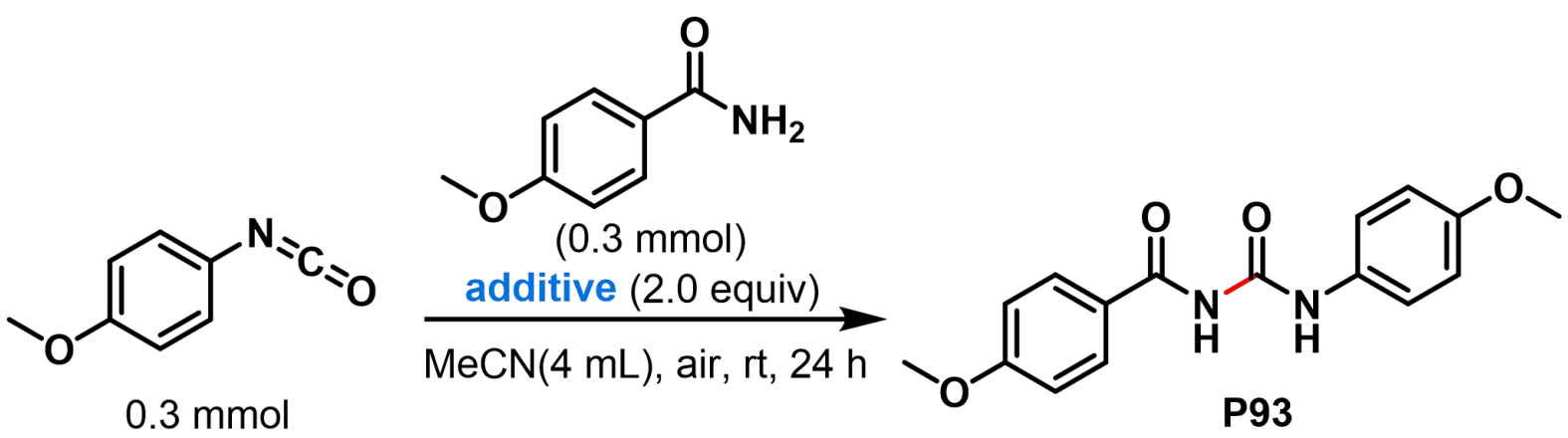

without additive	not detected
additive = NaI, I_2, HI,	not detected
= NaOH,	29%

To an 8 mL flask equipped with a stir bar was added 1-isocyanato-4-methoxybenzene (44.7 mg, 0.3 mmol, 1.0 equiv.), 4-methoxybenzamide (45.3 mg, 0.3 mmol, 1.0 equiv.), additive (0.6 mmol, 2.0 equiv.), and MeCN (4 mL). The mixture was stirred for 24 hours at room temperature in air (monitored by TLC and NMR), no product **P93** was obtained when the additive was NaI (89.9 mg), I_2 (152.3 mg), HCl (50 μL, 12.0 M solution in water), HI (139.5 mg, 55%w/w aq. soln.) or unused. However, when NaOH (24.0 mg) is added, the resulting residue was purified by chromatography on silica gel to give product **P93** (26.1 mg, 29% yield as a white solid, mp:

217.6-219.0 °C).

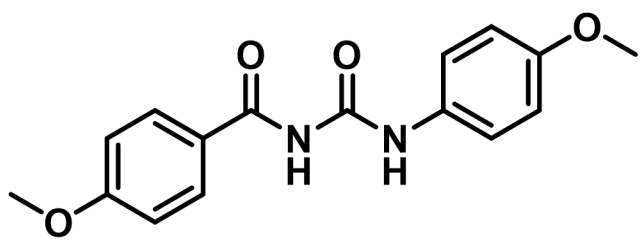

4-methoxy-N-((4-methoxyphenyl)carbamoyl)benzamide **(P93)**

^{1}H NMR (400 MHz, DMSO-d_6) δ 10.81 (s, 1H), 10.76 (s, 1H), 8.04 (d, J = 8.9 Hz, 2H), 7.49 (d, J = 9.0 Hz, 2H), 7.06 (d, J = 8.9 Hz, 2H), 6.93 (d, J = 9.0 Hz, 2H), 3.85 (s, 3H), 3.74 (s, 3H).

^{13}C NMR (101 MHz, DMSO-d_6) δ 167.9, 163.1, 155.7, 151.4, 130.7, 130.5, 124.2, 121.6, 114.1, 113.9, 55.6, 55.2.

HRMS（ESI, Q-TOF）m/z: [M+Na]$^+$ Calcd for $NaC_{16}H_{16}O_4N_2$, 323.1002; Found: 323.1005.

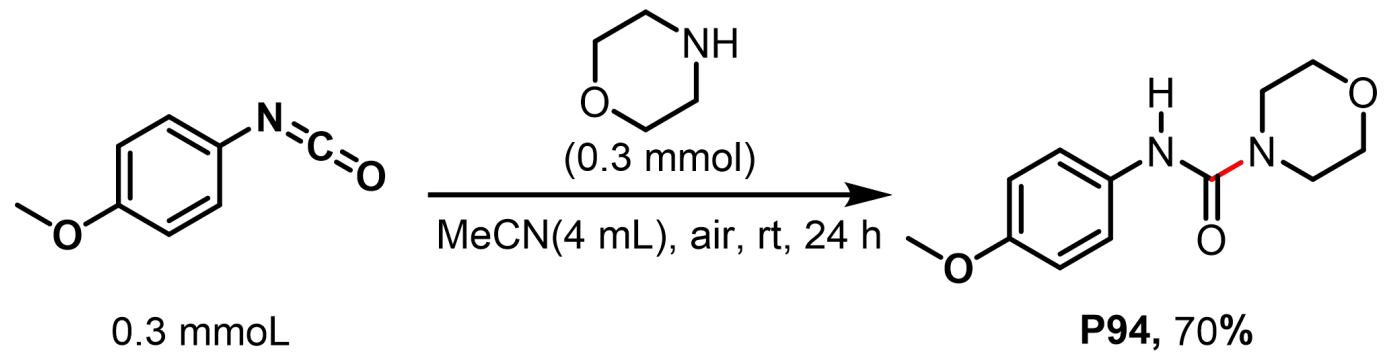

To an 8 mL flask equipped with a stir bar was added 1-isocyanato-4-methoxybenzene (44.7 mg, 0.3 mmol, 1.0 equiv.), morpholine (52.3 mg, 0.6 mmol, 2.0 equiv.), and MeCN (4 mL). The mixture was stirred for 24 hours at room temperature in air, concentrated the reaction mixture under reduced pressure. The resulting residue was purified by chromatography on silica gel to give the desired product **P94** (flash chromatography for purification: petroleum ether/ethyl acetate = 10:1−1:1, 49.6 mg, 70% yield as a white solid, mp: 118.4-119.9 °C).

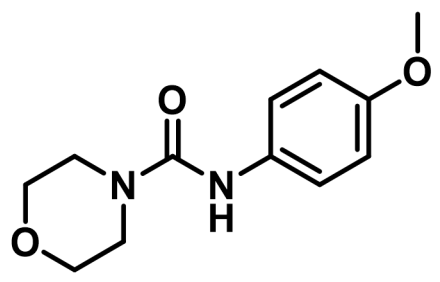

N-(4-methoxyphenyl)morpholine-4-carboxamide **(P94)**

^{1}H NMR (400 MHz, $CDCl_3$) δ 7.18 (d, J = 8.9 Hz, 2H), 6.81 – 6.73 (m, 3H), 3.74 (s, 3H), 3.64 – 3.57 (m, 4H), 3.39 – 3.32 (m, 4H).

^{13}C NMR (101 MHz, $CDCl_3$) δ 156.04, 156.02, 131.9, 123.0, 114.0, 66.5, 55.5, 44.2.

The analytical data are in accordance with reported literature.[8]

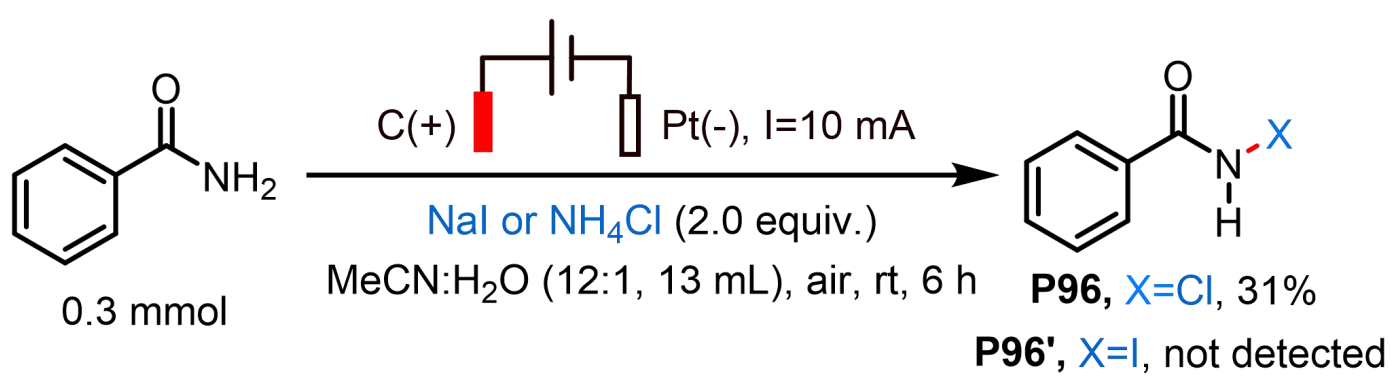

Benzamide (0.3 mmol, 1.0 equiv.), NH_4Cl (0.6 mmol, 2.0 equiv.), and MeCN:H_2O (12:1, 13 mL) were added to 25 mL three-neck flask with a teflon-coated stir bar. A graphite rod was used as the anode, and a platinum plate was used as the cathode. The resulting mixture was stirred and

electrolyzed with constant current 10 mA under air at room temperature until complete consumption of the amide (monitored by TLC). Then, transferred to a round-bottomed flask, concentrated *in vacuo*. The residue was purified by flash chromatography on silica gel to obtain the products **P96** (14.5 mg, 31% yield as a white solid), when NaI (0.6 mmol, 2.0 equiv.) instead of NH_4Cl (0.6 mmol, 2.0 equiv.), no product was obtained (**P96'**).

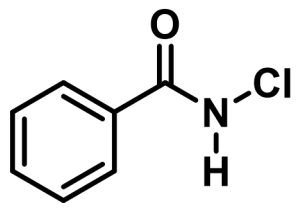

***N-chlorobenzamidee* (P96)**

^{1}H NMR (400 MHz, $CDCl_3$) δ 7.78 (d, J = 7.8 Hz, 2H), 7.74 (s, 1H), 7.53 (t, J = 6.8 Hz, 1H), 7.42 (t, J = 7.0 Hz, 2H).

^{13}C NMR (101 MHz, $CDCl_3$) δ 167.4, 132.7, 131.9, 129.0, 127.7.

The analytical data are in accordance with reported literature.[15]

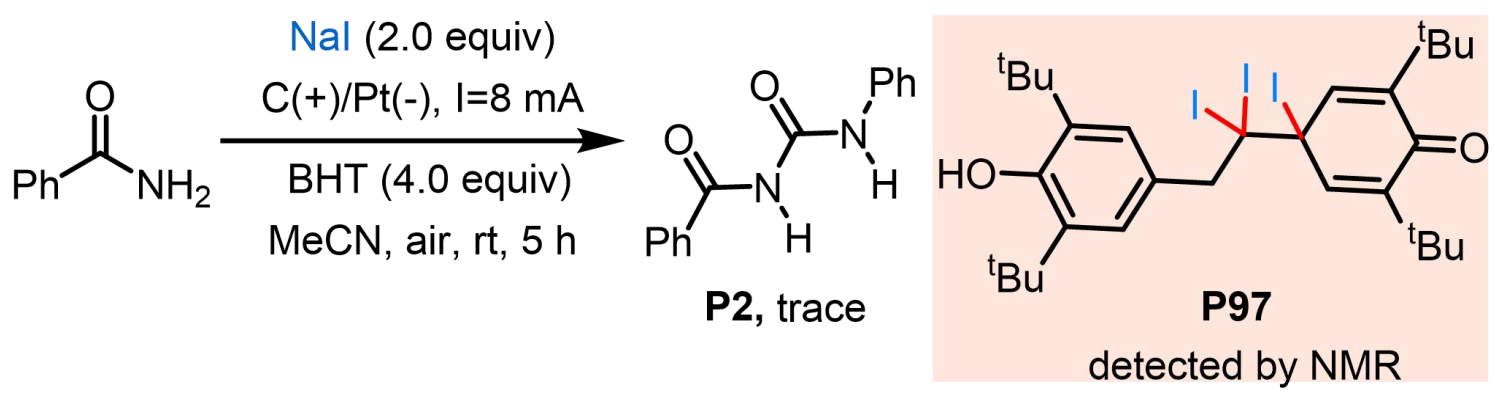

Benzamide (0.3 mmol, 1.0 equiv.), NaI (0.6 mmol, 2.0 equiv.), BHT (1.2 mmol, 4.0 equiv.), and MeCN (12 mL) were added to 25 mL three-neck flask with a teflon-coated stir bar. A graphite rod was used as the anode, and a platinum plate was used as the cathode. The resulting mixture was stirred and electrolyzed with constant current 8 mA for 5 hours under air at room temperature. Then, transferred to a round-bottomed flask, concentrated *in vacuo*. The residue was purified by flash chromatography on silica gel to obtain the products **P97** (88.2 mg, 18% yield as a yellow oil).

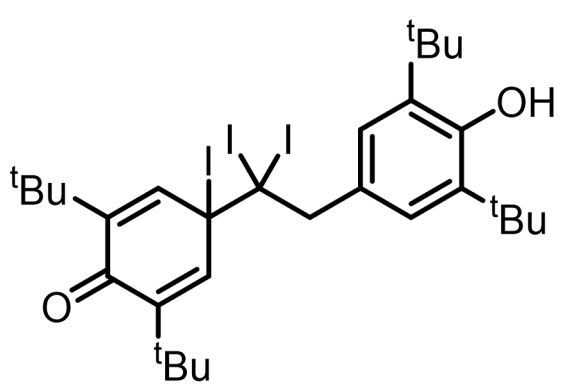

***2,6-di-tert-butyl-4-(2-(3,5-di-tert-butyl-4-hydroxyphenyl)-1,1-diiodoethyl)-4-iodocyclohexa-2,5-dien-1-one* (P97)**

^{1}H NMR (400 MHz, $CDCl_3$) δ 6.81 (s, 2H), 6.51 (s, 2H), 5.05 (s, 1H), 2.75 (s, 2H), 1.41 (s, 18H), 1.18 (s, 18H).

^{13}C NMR (101 MHz, $CDCl_3$) δ 186.6, 152.6, 146.8, 145.9, 135.2, 127.8, 126.6, 48.2, 41.1, 34.7, 34.3, 30.4, 29.6, 26.4.

HRMS（ESI, Q-TOF）m/z: [M] Calcd for $C_{30}H_{43}I_3O_2$-3I, 435.3263; Found:435.3253.

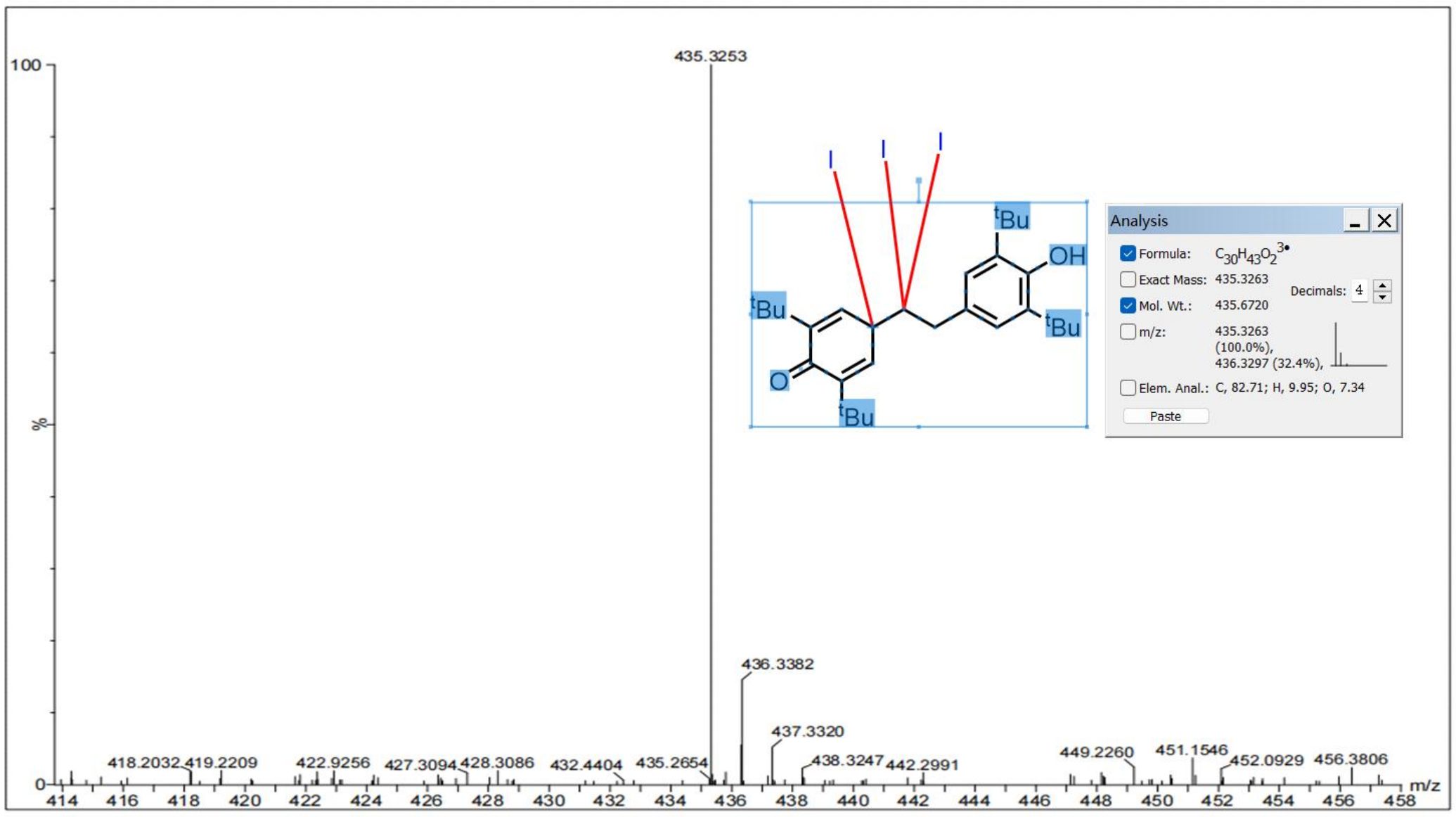

Figure S6. HRMS data of $C_{30}H_{43}I_3O_2$-3I.

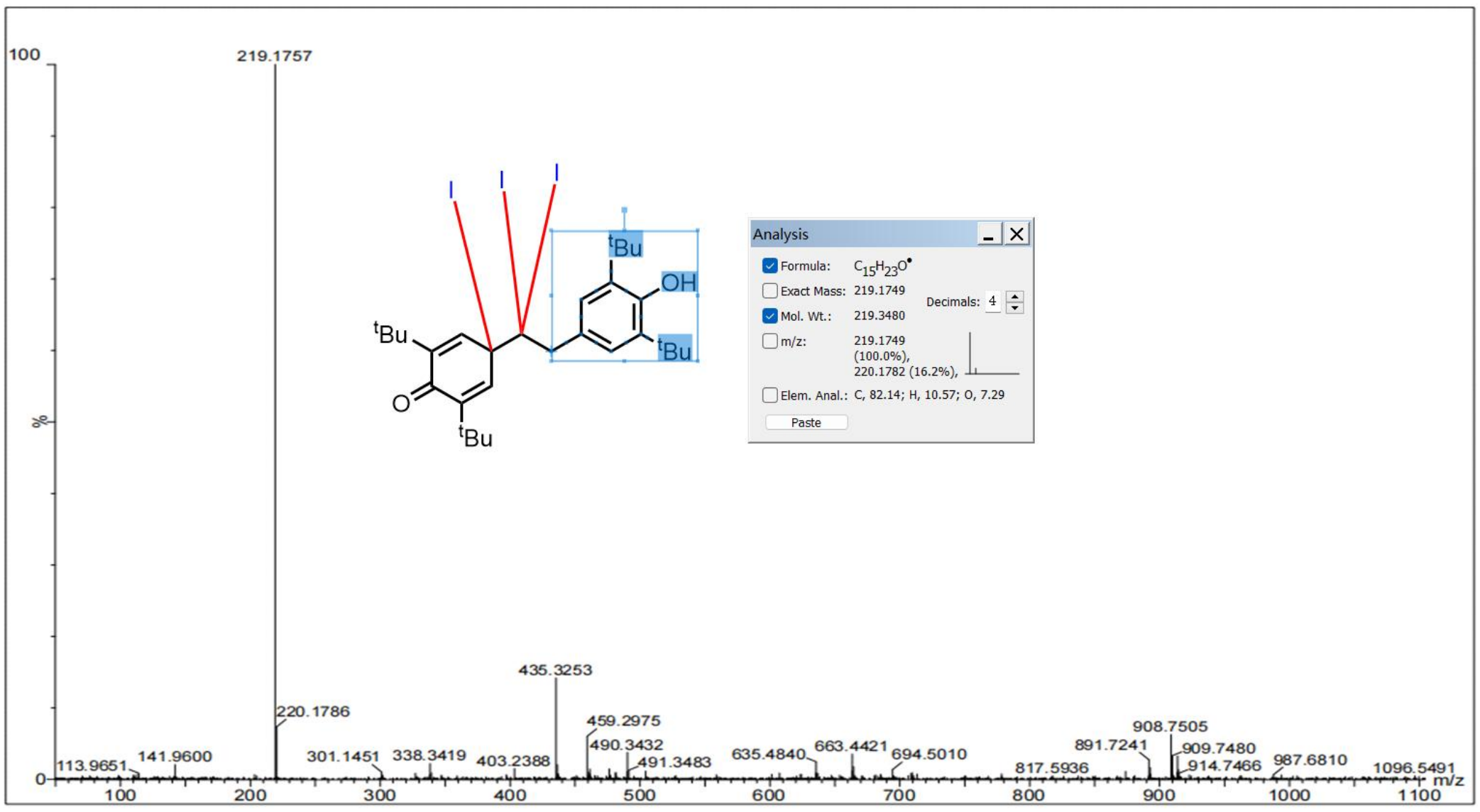

Figure S7. HRMS data of **P97** fragment.

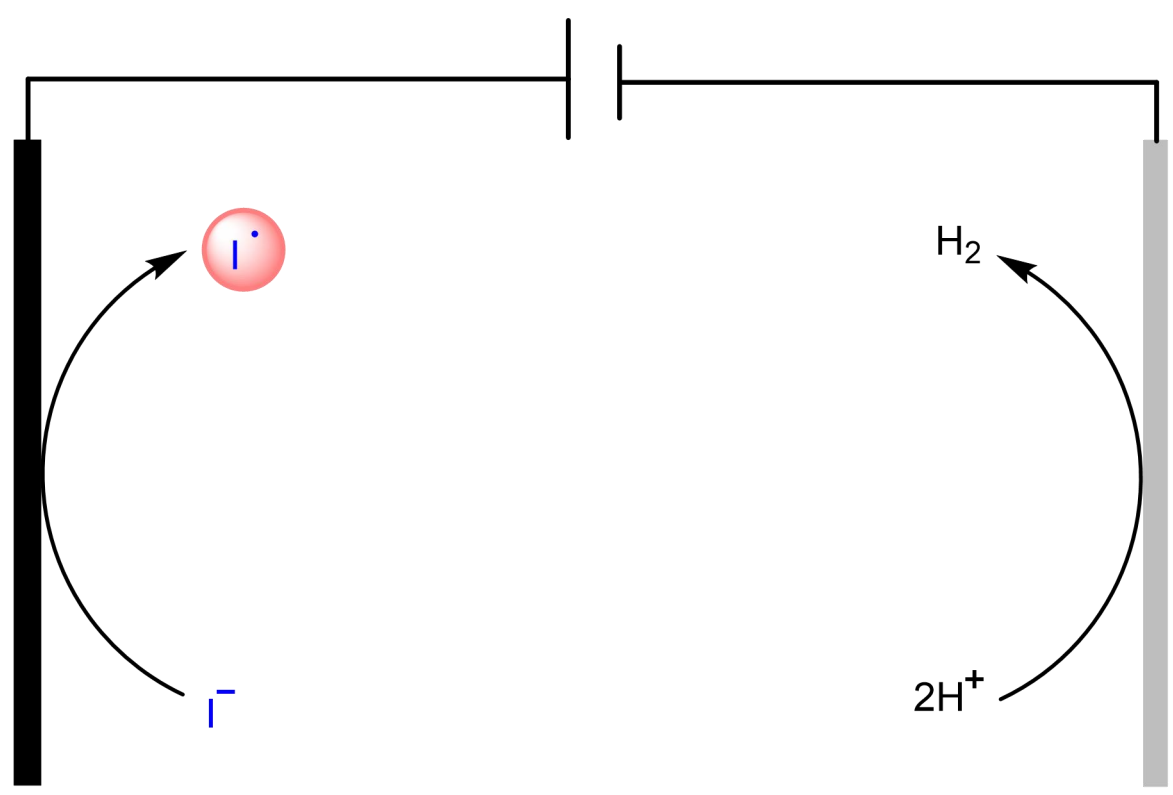

Figure S8. Electrocatalytic generation of iodine radicals.

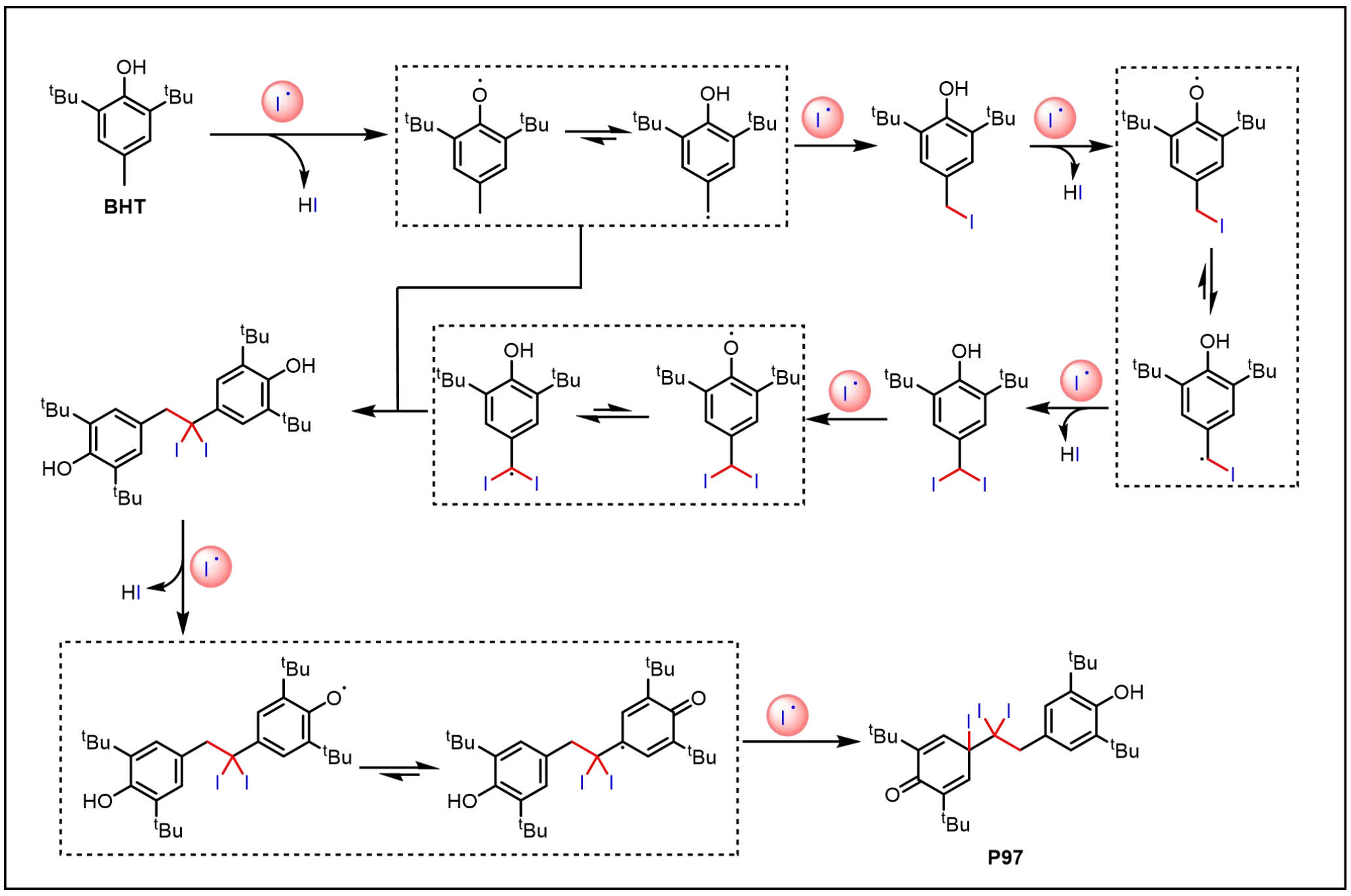

Figure S9. Proposed mechanism study of **P97**.

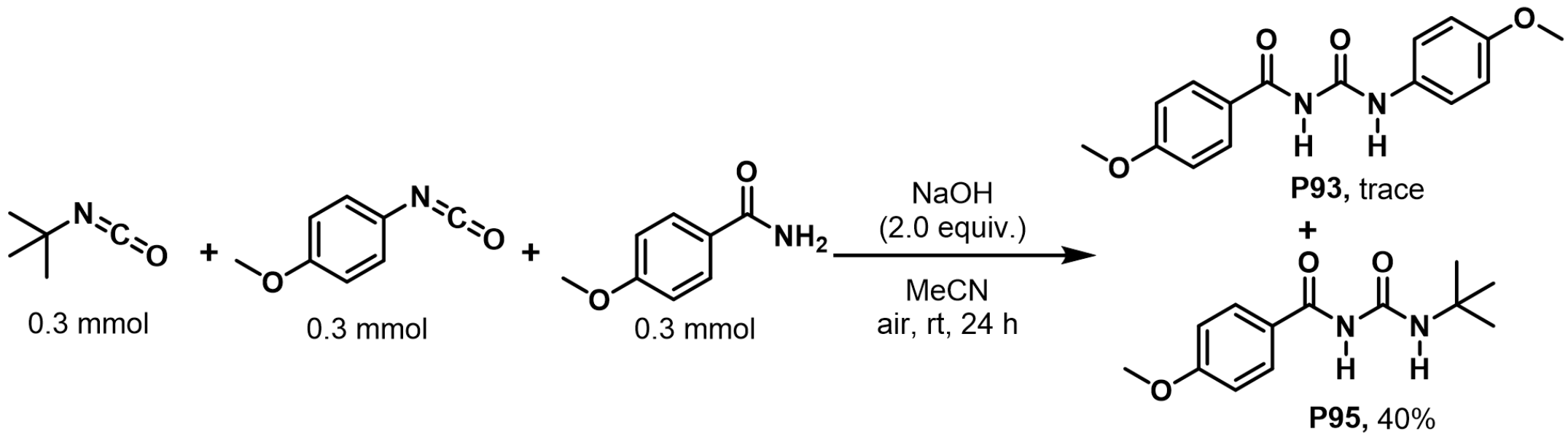

To an 8 mL flask equipped with a stir bar was added 1-isocyanato-4-methoxybenzene (44.7 mg, 0.3 mmol, 1.0 equiv.), tert-Butylisocyanate (29.7 mg, 0.3 mmol, 1.0 equiv.), 4-methoxybenzamide (45.3 mg, 0.3 mmol, 1.0 equiv.), NaOH (24 mg, 0.6 mmol, 2.0 equiv.), and MeCN (4 mL). The mixture was stirred for 24 hours at room temperature in air, concentrated the reaction mixture under reduced pressure. The resulting residue was purified by chromatography on silica gel to give the desired product **P95** (flash chromatography for purification: petroleum ether/ethyl acetate = 10:1−1:1, 30.0 mg, 40% yield as a white solid, mp: 174.1-176.9 °C).

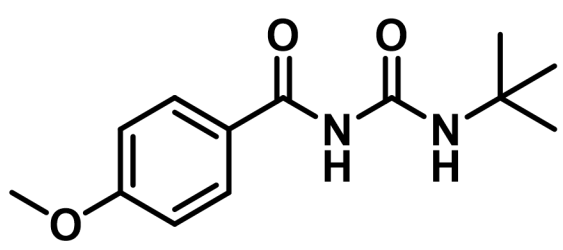

N-(tert-butylcarbamoyl)-4-methoxybenzamide **(P95)**

^{1}H NMR (400 MHz, $CDCl_3$) δ 9.97 (s, 1H), 8.94 (s, 1H), 8.05 (d, *J* = 8.6 Hz, 2H), 6.93 (d, *J* = 8.6 Hz, 2H), 3.86 (s, 3H), 1.44 (s, 9H).

^{13}C NMR (101 MHz, $CDCl_3$) δ 168.1, 163.4, 153.7, 130.2, 125.0, 114.0, 55.6, 51.0, 29.1.
HRMS（ESI, Q-TOF）m/z: $[M+Na]^+$ Calcd for $C_{13}H_{18}N_2NaO_3$, 273.1210; Found: 273.1216.

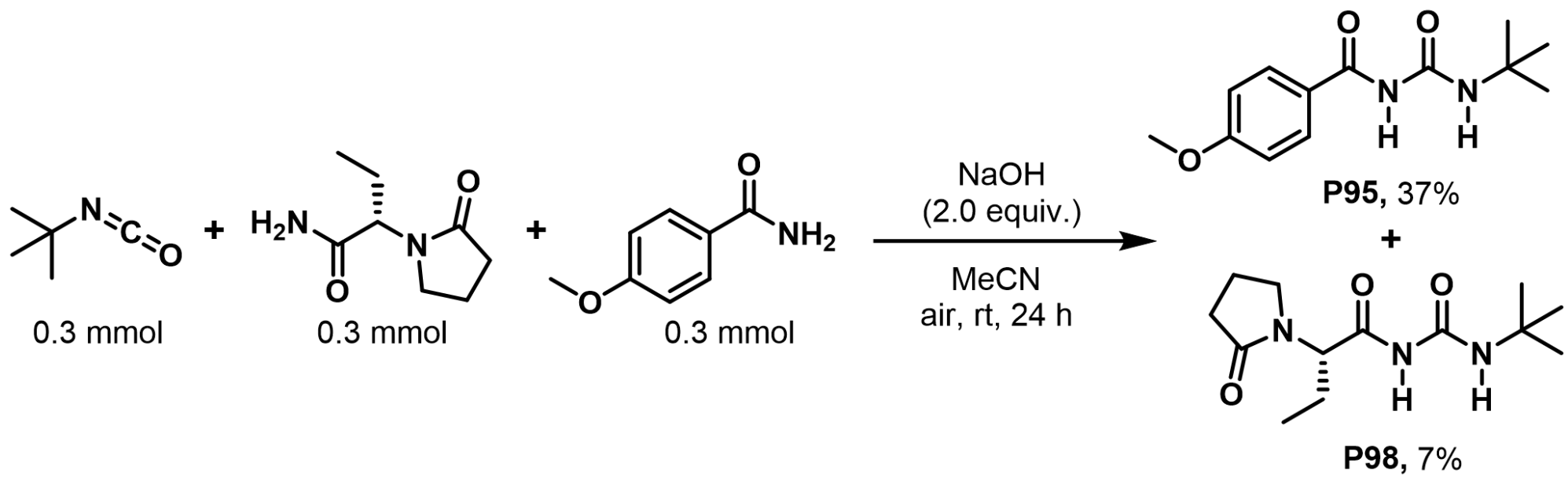

To an 8 mL flask equipped with a stir bar was added 1-isocyanato-4-methoxybenzene (44.7 mg, 0.3 mmol, 1.0 equiv.), tert-Butylisocyanate (29.7 mg, 0.3 mmol, 1.0 equiv.), 4-methoxybenzamide (45.3 mg, 0.3 mmol, 1.0 equiv.), NaOH (24 mg, 0.6 mmol, 2.0 equiv.), and MeCN (4 mL). The mixture was stirred for 24 hours at room temperature in air, concentrated the reaction mixture under reduced pressure. The resulting residue was purified by chromatography on silica gel to give the desired product **P95** (27.8 mg, 37% yield) or **P98** (flash chromatography for purification: petroleum ether/ethyl acetate = 10:1−1:1, 5.7 mg, 7% yield as a white solid, mp: 127.7-128.9 °C).

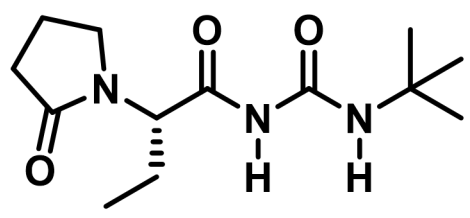

(S)-N-(tert-butylcarbamoyl)-2-(2-oxopyrrolidin-1-yl)butanamide **(P98)**
^{1}H NMR (400 MHz, $CDCl_3$) δ 9.87 (s, 1H), 8.24 (s, 1H), 4.65 – 4.48 (m, 1H), 3.66 – 3.27 (m, 2H), 2.37 (t, J = 7.3 Hz, 2H), 2.09 – 1.89 (m, 3H), 1.76 – 1.58 (m, 1H), 1.30 (s, 9H), 0.86 (t, J = 7.0 Hz, 3H).
^{13}C NMR (101 MHz, $CDCl_3$) δ 176.1, 173.0, 152.6, 56.7, 50.8, 43.9, 30.8, 28.8, 22.0, 18.3, 10.8.
HRMS（ESI, Q-TOF）m/z: $[M+Na]^+$ Calcd for $C_{13}H_{23}N_3NaO_3$, 292.1632; Found: 292.1636.

Table S3. Amide and isocyanate of controlled experiments.[a]

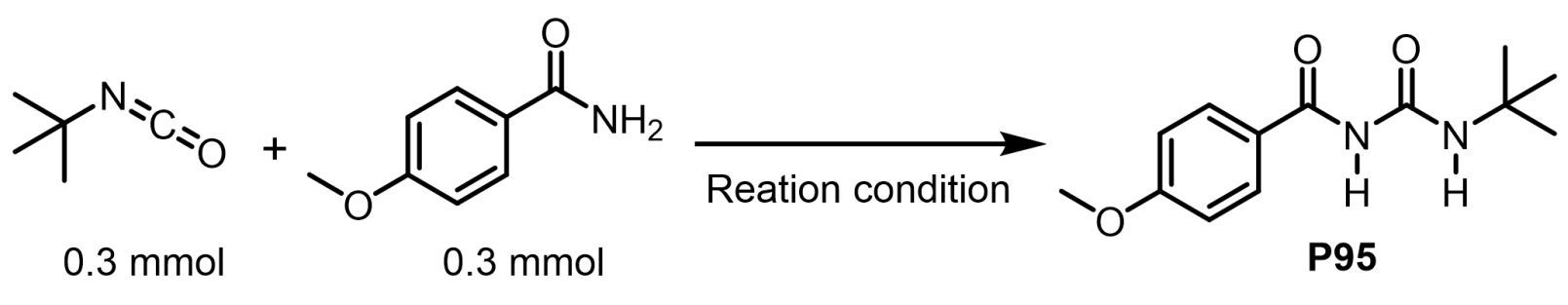

Entry	Reaction conditions	**P95** of Yield (%)[b]
1	MeCN, air, rt, 24 h	n.d.
2	NaOH (2.0 equiv.), MeCN, air, rt, 24 h	48
3	NaI (2.0 equiv.), MeCN, air, rt, 24 h	n.d.
4	I_2 (2.0 equiv.), MeCN, air, rt, 24 h	n.d.
5	HI (2.0 equiv.), MeCN, air, rt, 24 h	n.d.
6	Pt (-) / C (+), NaI (2.0 equiv.), MeCN, air, rt, 8 mA, 6 h	46

7	Pt (-) / C (+), $^{n}Bu_4NBF_4$ (2.0 equiv.), MeCN, air, rt, 8 mA, 6 h	30

[a] Without further notification, the reactions were performed with tert-Butylisocyanate (29.7 mg, 0.3 mmol, 1.0 equiv.), 4-methoxybenzamide (45.3 mg, 0.3 mmol, 1.0 equiv.); no electricity: MeCN (4 mL); electricity: MeCN (12 mL); [b] Isolated yields; n.d.: not detected.

8. X-ray crystallographic data

Preparation of the crystal: Single crystals for X-ray studies of compound **P27** and **P34** were obtained by slow evaporation of a solution of the compound in the mixture of trichloromethane and *n*-hexane at room temperature.

Crystal measurement: Single crystals of compound **P27** and **P34** were collected on a Rigaku Oxford Diffraction Supernova Dual Source, Cu at Zero, AtlasS2 diffractometer using Cu Kα radiation. The data were collected and processed using CrysAlisPro. Crystallographic data for the structure reported in this paper have been deposited at the Cambridge Crystallographic Data Center and allocated with the deposition numbers: CCDC 2443330 of compound **P27**; CCDC 2443332 of compound **P34**.

The ellipsoids are shown at 30% probability levels

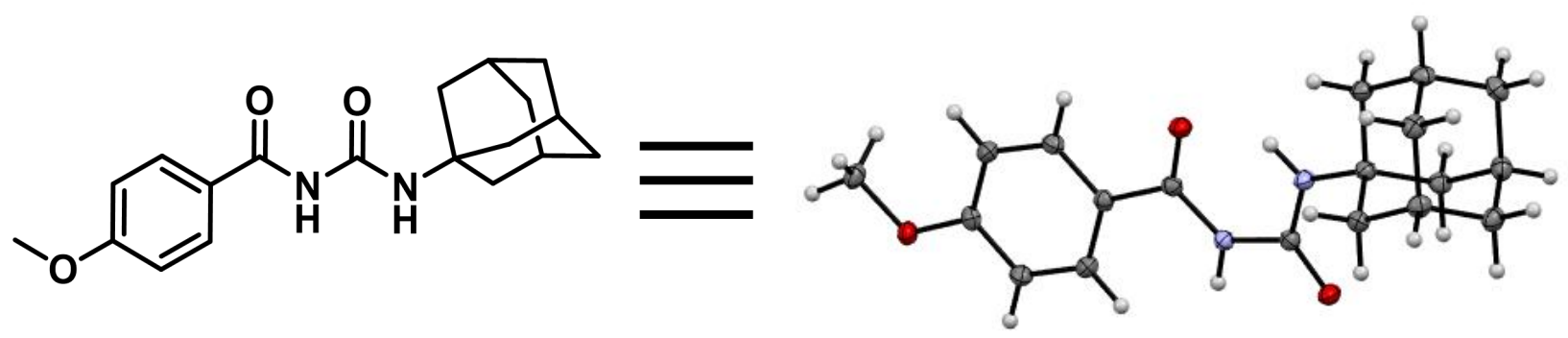

Crystal data and structure refinement for **CCDC 2443330**.

Identification code	**P27**
Empirical formula	$C_{19}H_{24}N_2O_3$
Formula weight	328.40
Temperature/K	100.00(10)
Crystal system	triclinic
Space group	P-1
a/Å	7.31858(10)
b/Å	10.27929(15)
c/Å	11.21498(14)
α/°	102.2736(12)
β/°	100.8324(11)
γ/°	90.8120(11)
Volume/Å^3	808.44(2)
Z	2

ρ_{calc}g/cm^3	1.349
μ/mm^{-1}	0.738
F(000)	352.0
Crystal size/mm^3	0.15 × 0.13 × 0.11
Radiation	Cu Kα (λ = 1.54184)
2Θ range for data collection/°	8.226 to 146.83
Index ranges	-9 ≤ h ≤ 7, -12 ≤ k ≤ 12, -13 ≤ l ≤ 13
Reflections collected	9970
Independent reflections	3116 [R_{int} = 0.0270, R_{sigma} = 0.0251]
Data/restraints/parameters	3116/0/226
Goodness-of-fit on F^2	1.062
Final R indexes [I>=2σ (I)]	R_1 = 0.0373, wR_2 = 0.0979
Final R indexes [all data]	R_1 = 0.0401, wR_2 = 0.1002
Largest diff. peak/hole / e Å^{-3}	0.18/-0.33

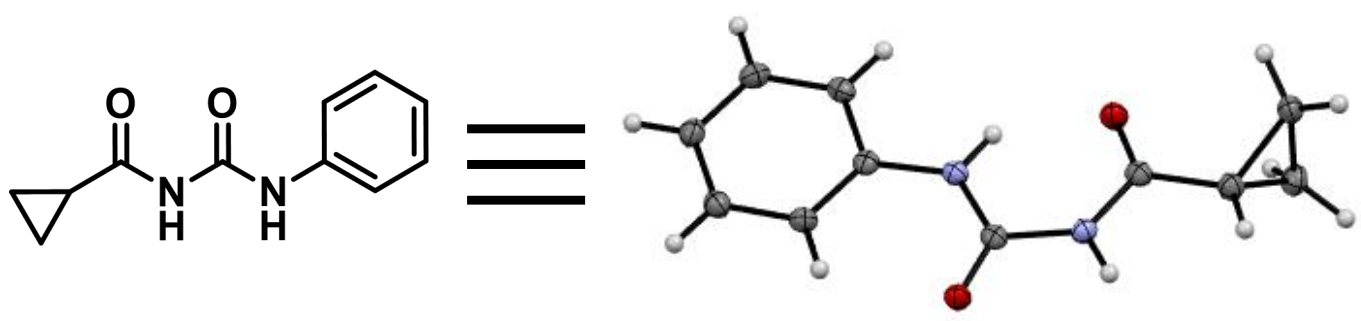

Crystal data and structure refinement for **CCDC 2443332**.

Identification code	**P34**
Empirical formula	$C_{11}H_{12}N_2O_2$
Formula weight	204.23
Temperature/K	99.99(10)
Crystal system	monoclinic
Space group	$P2_1/n$
a/Å	12.8342(2)
b/Å	5.48290(10)
c/Å	14.0333(2)
α/°	90
β/°	97.8800(10)
γ/°	90
Volume/Å^3	978.18(3)
Z	4
ρ_{calc}g/cm^3	1.387
μ/mm^{-1}	0.798
F(000)	432.0
Crystal size/mm^3	0.15 × 0.13 × 0.12
Radiation	Cu Kα (λ = 1.54184)
2Θ range for data collection/°	8.758 to 145.476
Index ranges	-15 ≤ h ≤ 12, -2 ≤ k ≤ 6, -17 ≤ l ≤ 17

Reflections collected	6256
Independent reflections	1893 [R_{int} = 0.0244, R_{sigma} = 0.0239]
Data/restraints/parameters	1893/0/145
Goodness-of-fit on F^2	1.070
Final R indexes [I>=2σ (I)]	R_1 = 0.0341, wR_2 = 0.0904
Final R indexes [all data]	R_1 = 0.0372, wR_2 = 0.0931
Largest diff. peak/hole / e $Å^{-3}$	0.44/-0.17

9. Referances

[1] S. Aich, M. Saha, D. Ghosh, S. A. Molla, A. K. Sarkar, D. Bag, R. Rahaman, S. Khamarui and D. K. Maiti, *Org. Lett.,* **2024,** *26,* 10970-10975.

[2] T. Librowski, M. Kubacka, M. Meusel, S. Scolari, C. E. Müller and M. Gütschow, *Eur. J. Pharmacol.,* **2007,** *559,* 138-149.

[3] M. V. Il'in, L. A. Lesnikova, D. S. Bolotin, A. S. Novikov, V. V. Suslonov and V. Y. Kukushkin, *New J. Chem.,* **2020,** *44,* 1253-1262.

[4] M. S. Yadav, S. K. Singh, A. K. Agrahari, A. S. Singh and V. K. Tiwari, *Synth.,* **2021,** *53,* 2494-2502.

[5] A. K. Singh, R. Chawla and L. D. S. Yadav, *Tetrahedron Lett.,* **2013,** *54,* 5099-5102.

[6] Z. Gan, G. Li, Q. Yan, W. Deng, Y.-Y. Jiang and D. Yang, *Green Chem.,* **2020,** *22,* 2956-2962.

[7] P. Dubé, N. F. F. Nathel, M. Vetelino, M. Couturier, C. L. Aboussafy, S. Pichette, M. L. Jorgensen and M. Hardink, *Org. Lett.,* **2009,** *11,* 5622-5625.

[8] K. E. Houghtling, A. M. Canfield and S. M. Paradine, *Org. Lett.,* **2022,** *24,* 5787-5790.

[9] W. Rauf, A. L. Thompson and J. M. Brown, *Chem. Commun.,* **2009,** 3874-3876.

[10] A. D. Karche, P. Kamalakannan, R. Powar, G. G. Shenoy and K. J. Padiya, *Org. Process Res. Dev.,* **2022,** *26,* 3141-3152.

[11] C. E. Houlden, M. Hutchby, C. D. Bailey, J. G. Ford, S. N. G. Tyler, M. R. Gagné, G. C. Lloyd-Jones and K. I. Booker-Milburn, *Angew. Chem. Int. Ed.,* **2009,** *48,* 1830-1833.

[12] C. E. Houlden, C. D. Bailey, J. G. Ford, M. R. Gagné, G. C. Lloyd-Jones and K. I. Booker-Milburn, *J. Am. Chem. Soc.,* **2008,** *130,* 10066-10067.

[13] J. Wang, S. Wang, Z. Wei, P. Wang, Y. Cao, Y. Huang, L. He and A. Lei, *Science* **2024,** *386,* 776-782.

[14] J. Li, X. Wang, Z. Wang and Y. Shi, *Org. Lett.,* **2021,** *23,* 8958−8962

[15] L. Song, Y. Meng, T. Zhao, L. Liu, X. Pan, B. Huang, H. Yao, R. Lin and R. Tong, *Green Chem.,* **2024,** *26,* 428-438.

10. HPLC data

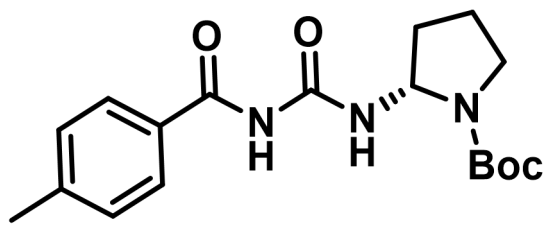

tert-butyl (S)-2-(3-(4-methylbenzoyl)ureido)pyrrolidine-1-carboxylate **(P18)**

Chiral HPLC analysis: Diacel Chiralpak AD-H, hexane/isopropanol = 85/15, flow rate 1.0 mL/min, detection at 210 nm, $t_{(minor)}$ = 14.34 min, $t_{(major)}$ = 18.64 min.

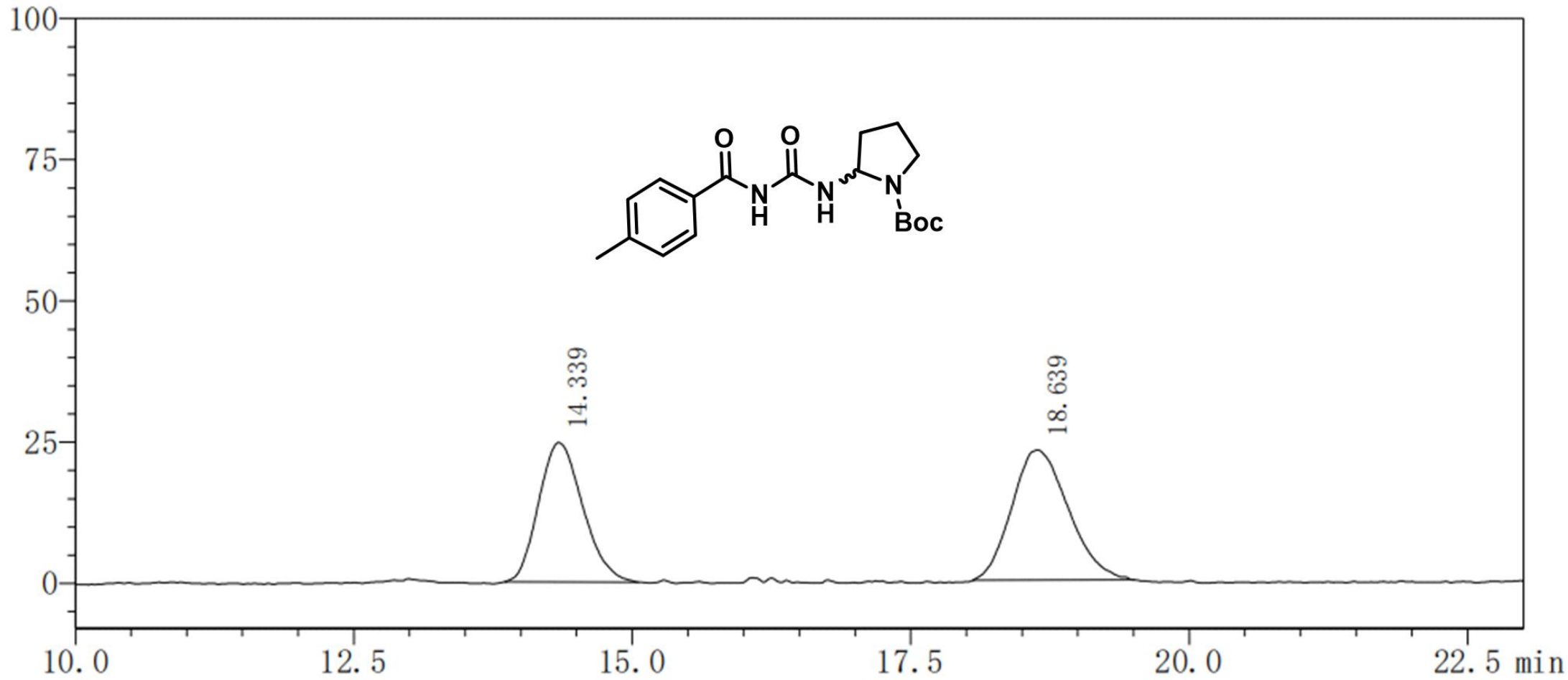

PDA 210 nm

ID#	Rt. Time	Area	Height	Area %	Resolution
1	14.339	679437	24656	45.511	
2	18.639	813473	22979	54.489	5.164

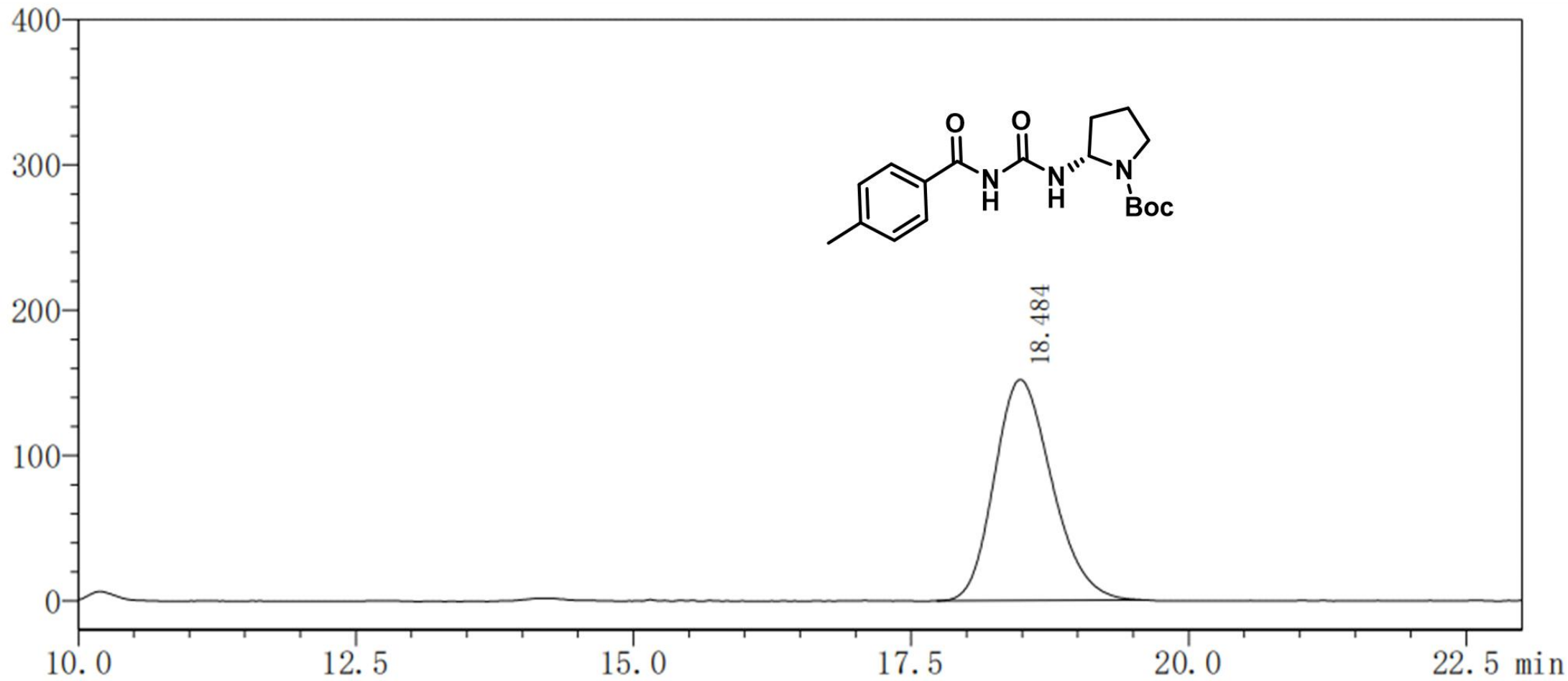

PDA 210 nm

ID#	Rt. Time	Area	Height	Area %	Resolution
1	18.484	5338582	150303	100.000	
2	--	--	--	--	--
				＞99% ee	

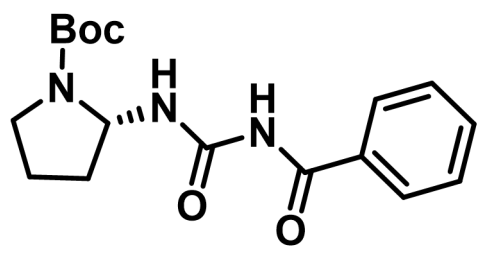

tert-butyl (S)-2-(3-benzoylureido)pyrrolidine-1-carboxylate **(P19)**

Chiral HPLC analysis: Diacel Chiralpak AD-H, hexane/isopropanol = 85/15, flow rate 1.0 mL/min, detection at 210 nm, $t_{(minor)}$ = 11.48 min, $t_{(major)}$ = 14.97 min.

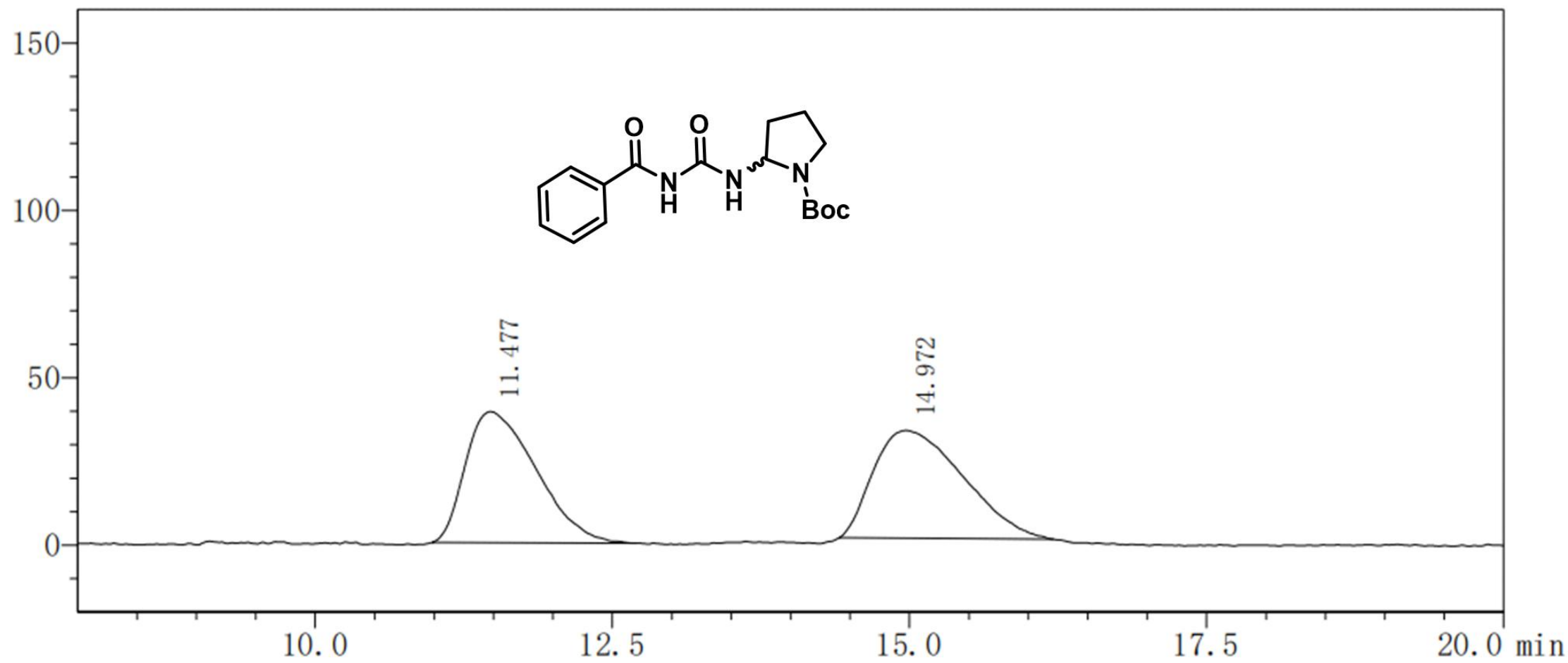

PDA 210 nm

ID#	Rt. Time	Area	Height	Area %	Resolution
1	11.477	1610590	39086	48.857	
2	14.972	1685959	32158	51.143	2.776

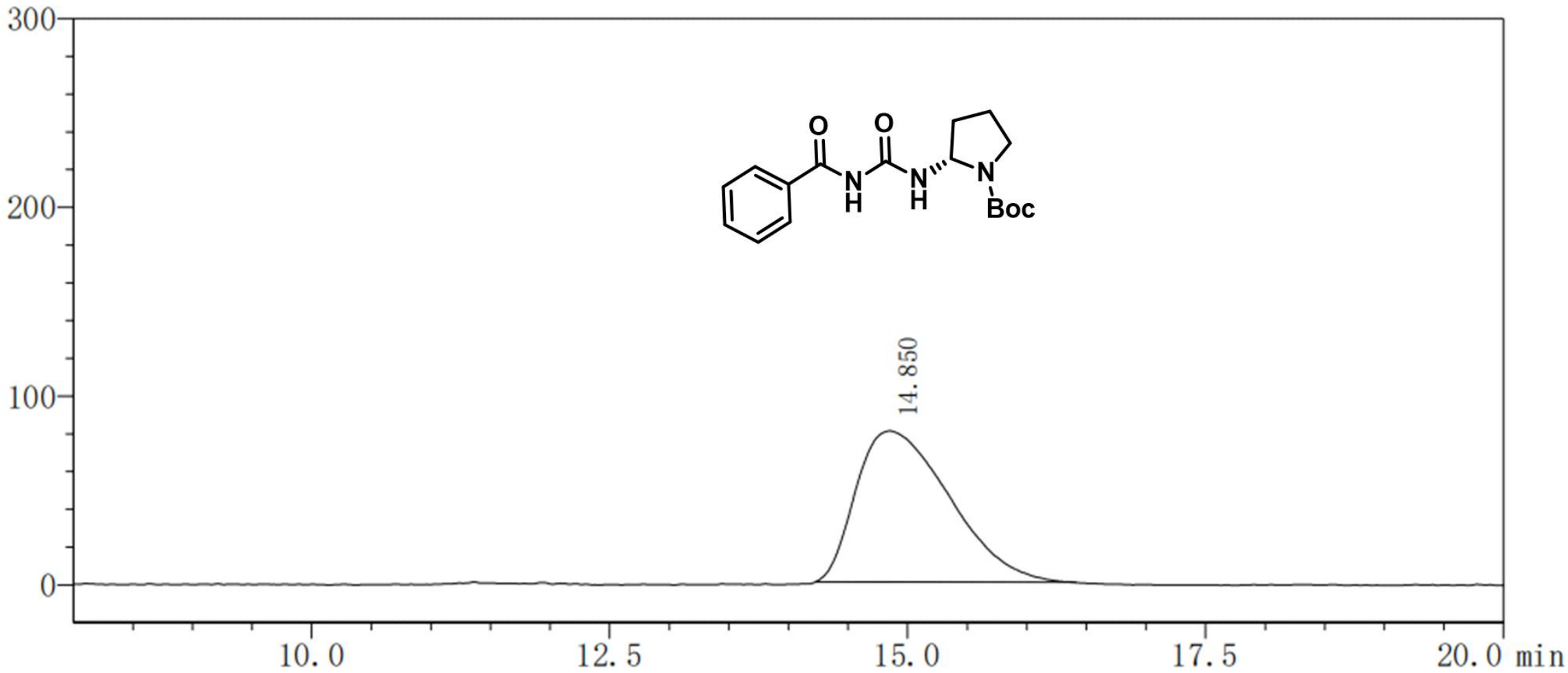

PDA 210 nm

ID#	Rt. Time	Area	Height	Area %	Resolution
1	14.850	4377886	80131	100.000	
2	--	--	--	--	--
				＞99% ee	

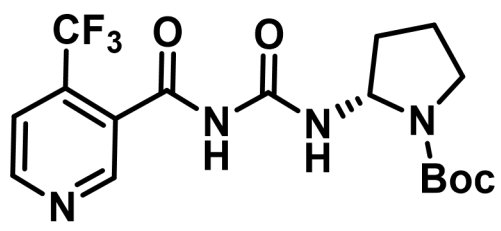

***tert*-butyl (S)-2-(3-(4-(trifluoromethyl)nicotinoyl)ureido)pyrrolidine-1-carboxylate** (**P20**)

Chiral HPLC analysis: Diacel Chiralpak AD-H, hexane/isopropanol = 85/15, flow rate 1.0 mL/min, detection at 210 nm, $t_{(minor)}$ = 11.09 min, $t_{(major)}$ = 16.65 min.

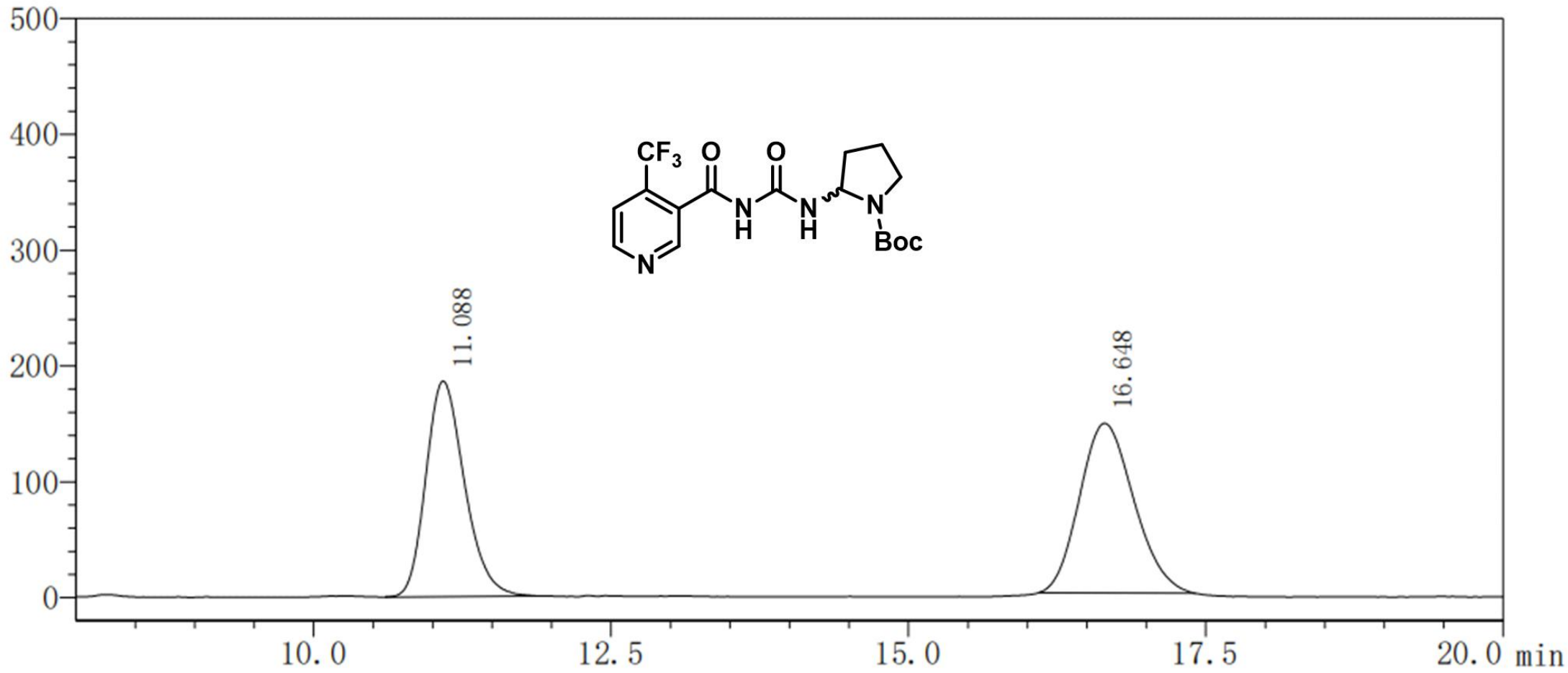

PDA 210 nm

ID#	Rt. Time	Area	Height	Area %	Resolution
1	11.088	4215974	186088	47.497	
2	16.648	4660385	146449	52.503	7.692

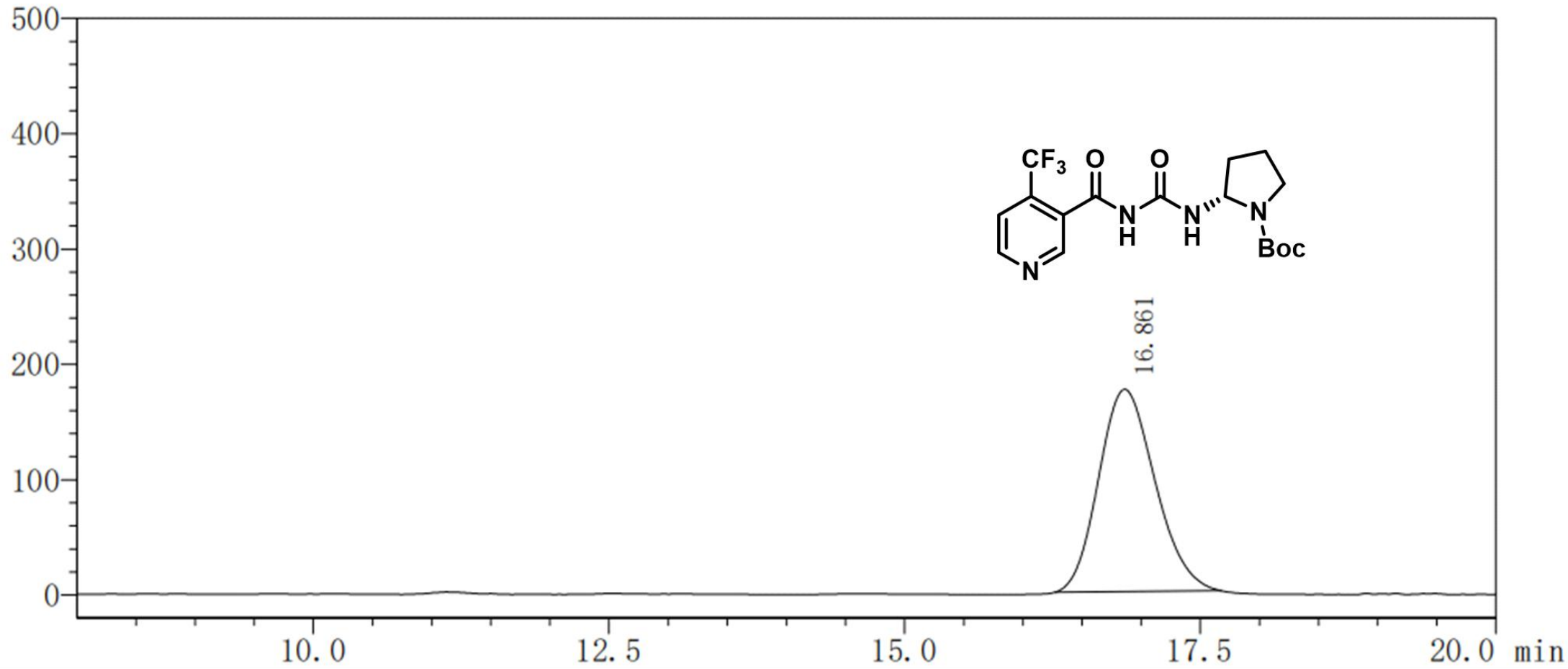

PDA 210 nm

ID#	Rt. Time	Area	Height	Area %	Resolution
1	16.861	5807149	175245	100.000	
2	--	--	--	--	--
				＞99% ee	

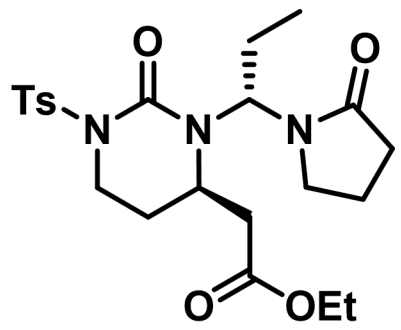

ethyl 2-((R)-2-oxo-3-((R)-1-(2-oxopyrrolidin-1-yl)propyl)-1-tosylhexahydropyrimidin-4-yl)acetate **(P87)**

Chiral HPLC analysis: Diacel Chiralpak AD-H, hexane/isopropanol = 75/25, flow rate 1.0 mL/min, detection at 210 nm, $t_{(major)}$ = 20.18 min, $t_{(minor)}$ = 41.31 min.

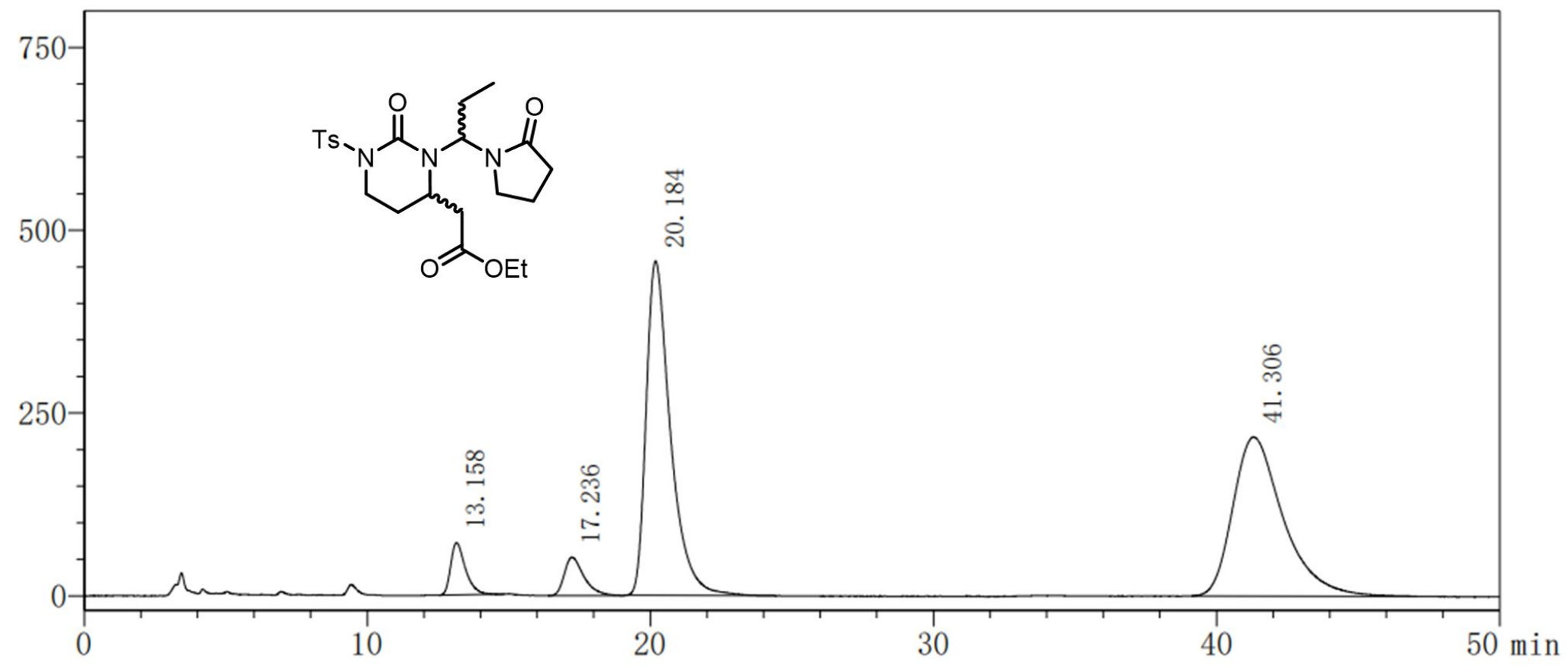

PDA 210 nm

ID#	Rt. Time	Area	Height	Area %	Resolution
1	13.158	2612054	71718	4.407	
2	17.236	2522078	52556	4.256	3.763
3	20.184	27279986	457623	46.031	2.161
4	41.306	26850694	217479	45.306	9.089

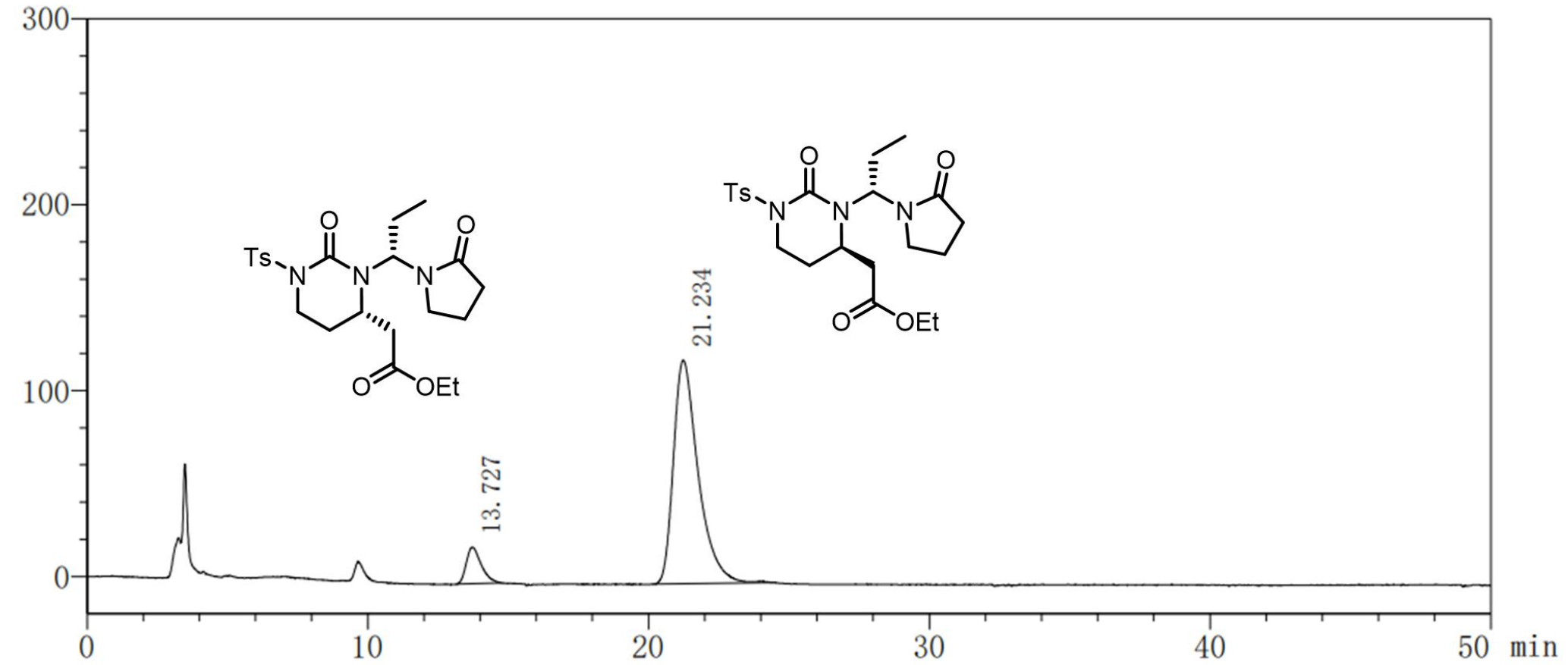

PDA 210 nm

ID#	Rt. Time	Area	Height	Area %	Resolution
1	13.727	716345	19490	8.833	
2	--	--	--	--	--
3	21.234	7393249	120319	91.167	5.938
4	--	--	--	--	--
				＞99% ee	

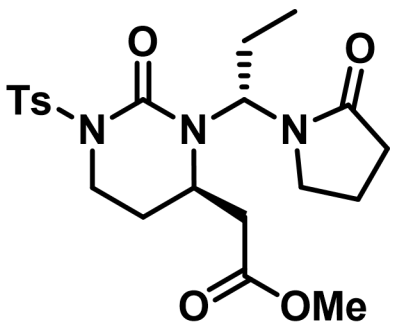

methyl 2-((R)-2-oxo-3-((R)-1-(2-oxopyrrolidin-1-yl)propyl)-1-tosylhexahydropyrimidin-4-yl)acetate **(P88)**

Chiral HPLC analysis: Diacel Chiralpak AD-H, hexane/isopropanol = 75/25, flow rate 1.0 mL/min, detection at 210 nm, $t_{(major)}$ = 18.14 min, $t_{(minor)}$ = 30.82 min.

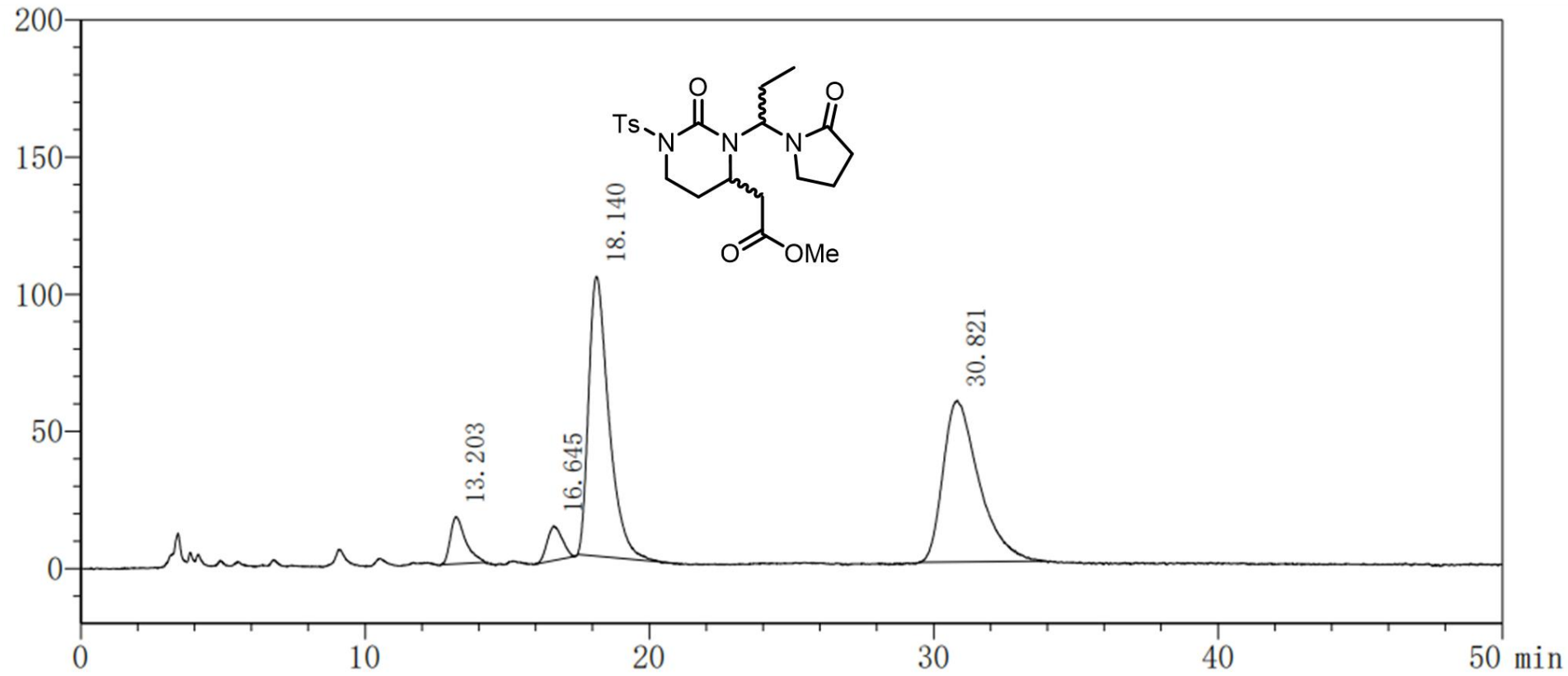

PDA 210 nm

ID#	Rt. Time	Area	Height	Area %	Resolution
1	13.203	634411	17091	5.596	
2	16.645	464022	12495	4.093	3.512
3	18.140	5057202	102026	44.606	1.295
4	30.821	5181869	58778	45.706	7.150

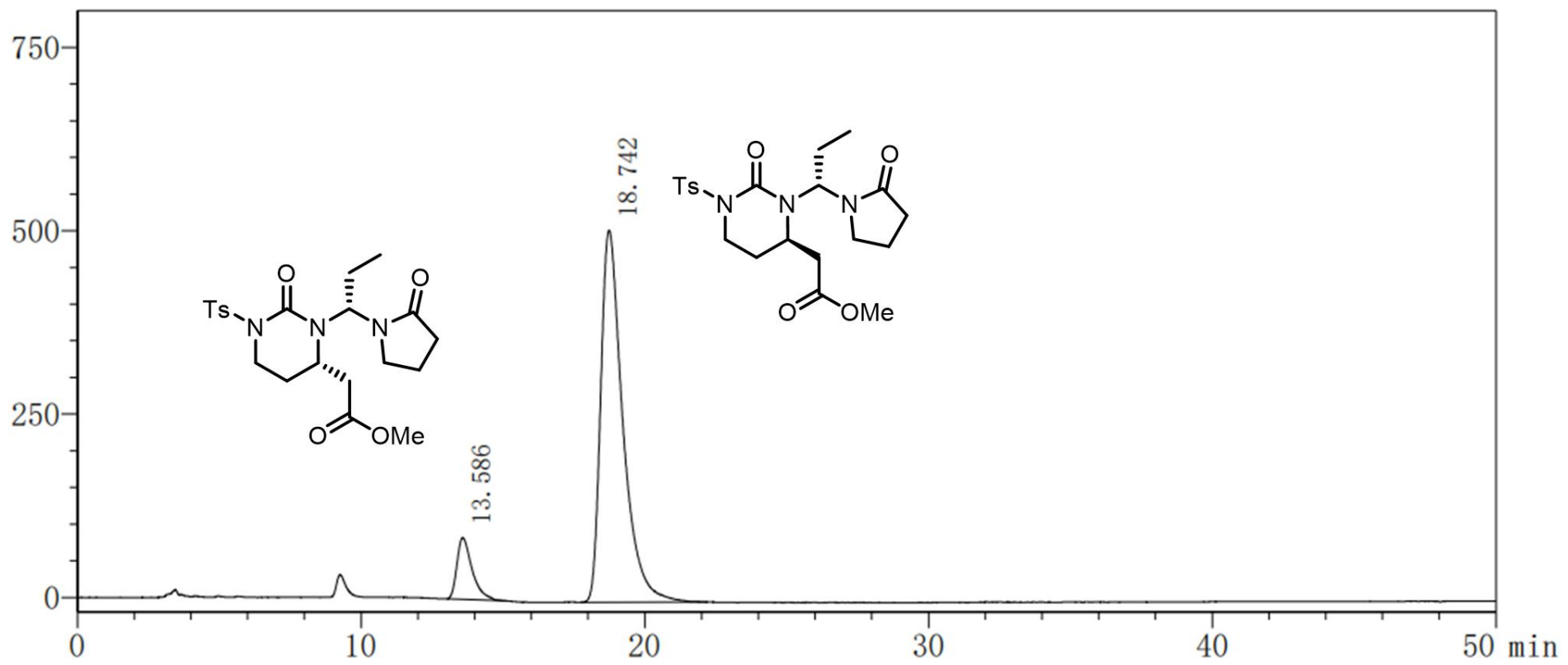

PDA 210 nm

ID#	Rt. Time	Area	Height	Area %	Resolution
1	13.586	3118783	84076	10.179	
2	--	--	--	--	--
3	18.742	27521689	507351	89.821	4.501
4	--	--	--	--	--
				＞99% ee	

11. NMR spectra

^{1}H NMR of P1
DMSO-d_6, 400 MHz

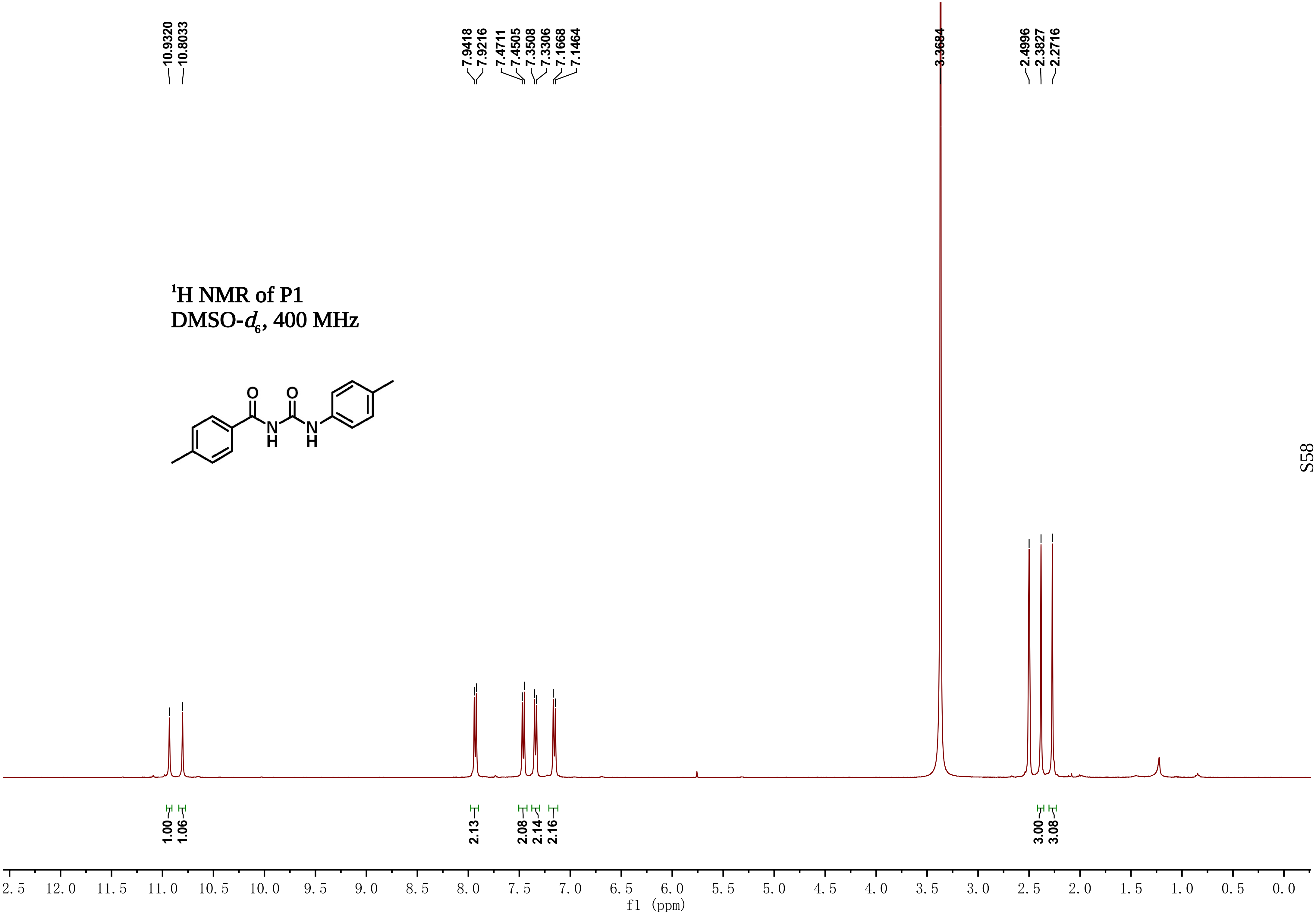

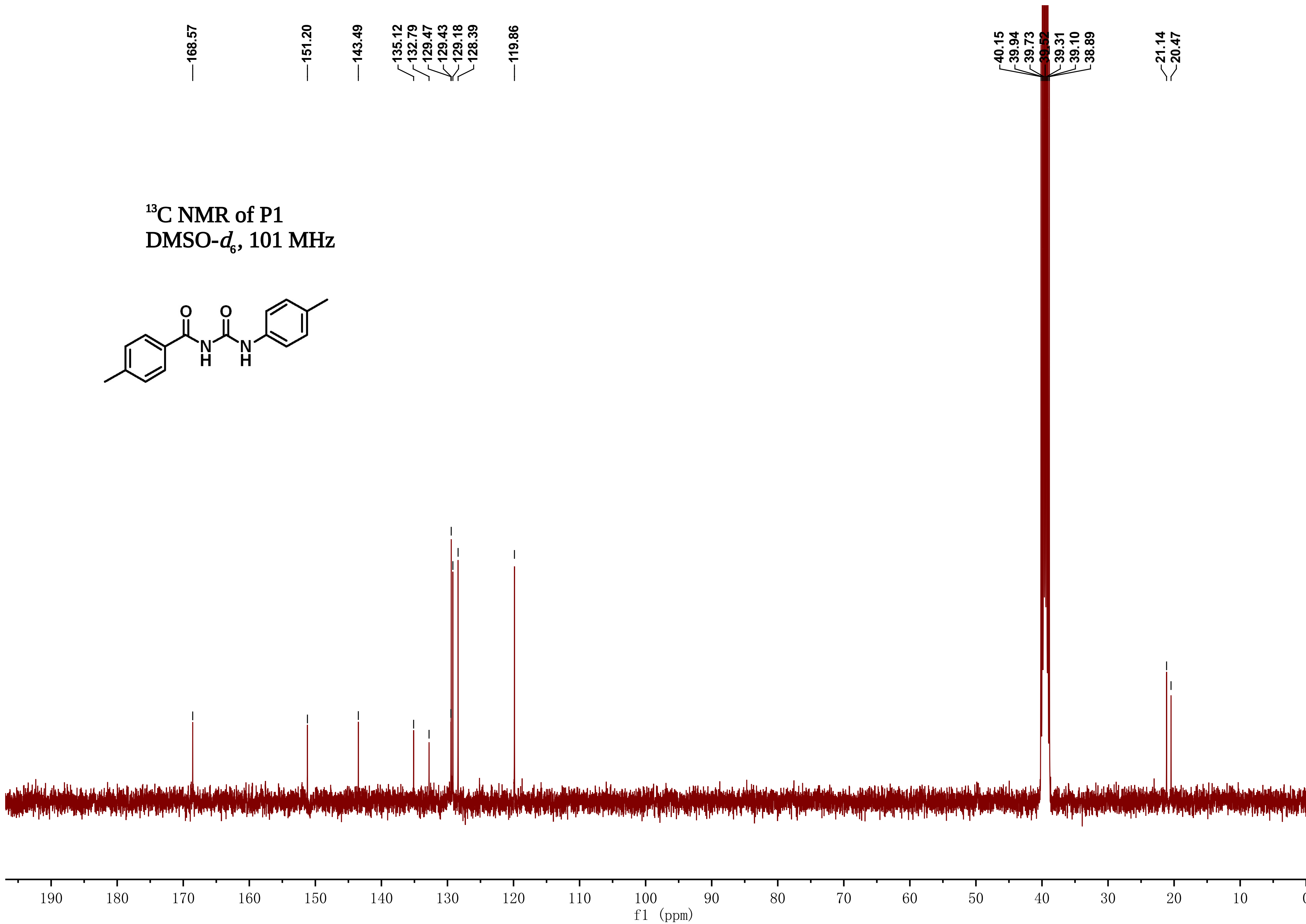

168.57
151.20
143.49
135.12
132.79
129.47
129.43
129.18
128.39
119.86
40.15
39.94
39.73
39.52
39.31
39.10
38.89
21.14
20.47
13C NMR of P1
DMSO-d6, 101 MHz
O
O
N
H
N
H
190
180
170
160
150
140
130
120
110
100
90
80
70
60
50
40
30
20
10
0
f1 (ppm)

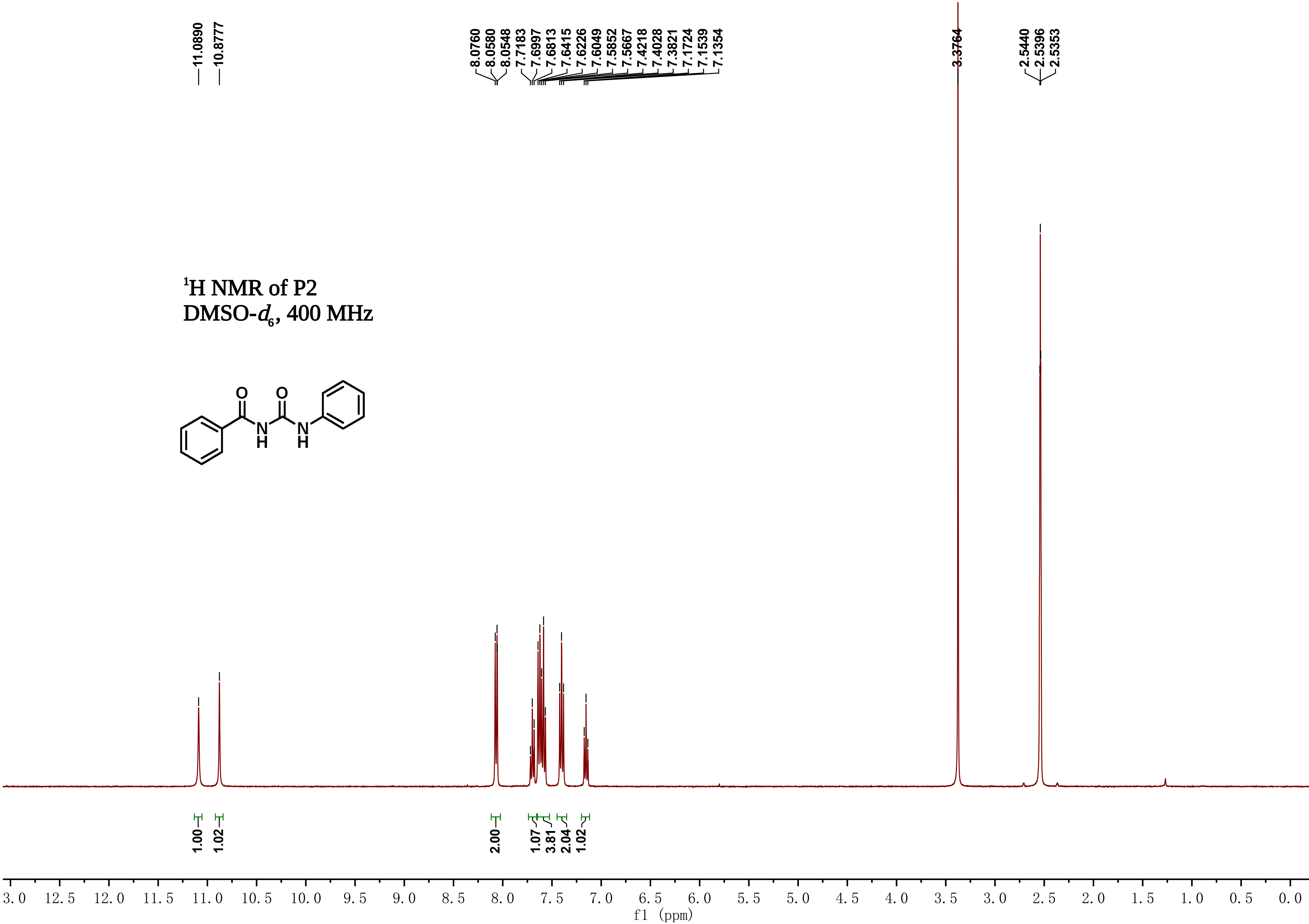

11.0890
10.8777
8.0760
8.0580
8.0548
7.7183
7.6997
7.6813
7.6415
7.6226
7.6049
7.5852
7.5667
7.4218
7.4028
7.3821
7.1724
7.1539
7.1354
3.3764
2.5440
2.5396
2.5353
1H NMR of P2
DMSO-d6, 400 MHz
O
O
N
H
N
H
1.00
1.02
2.00
1.07
3.81
2.04
1.02
f1 (ppm)

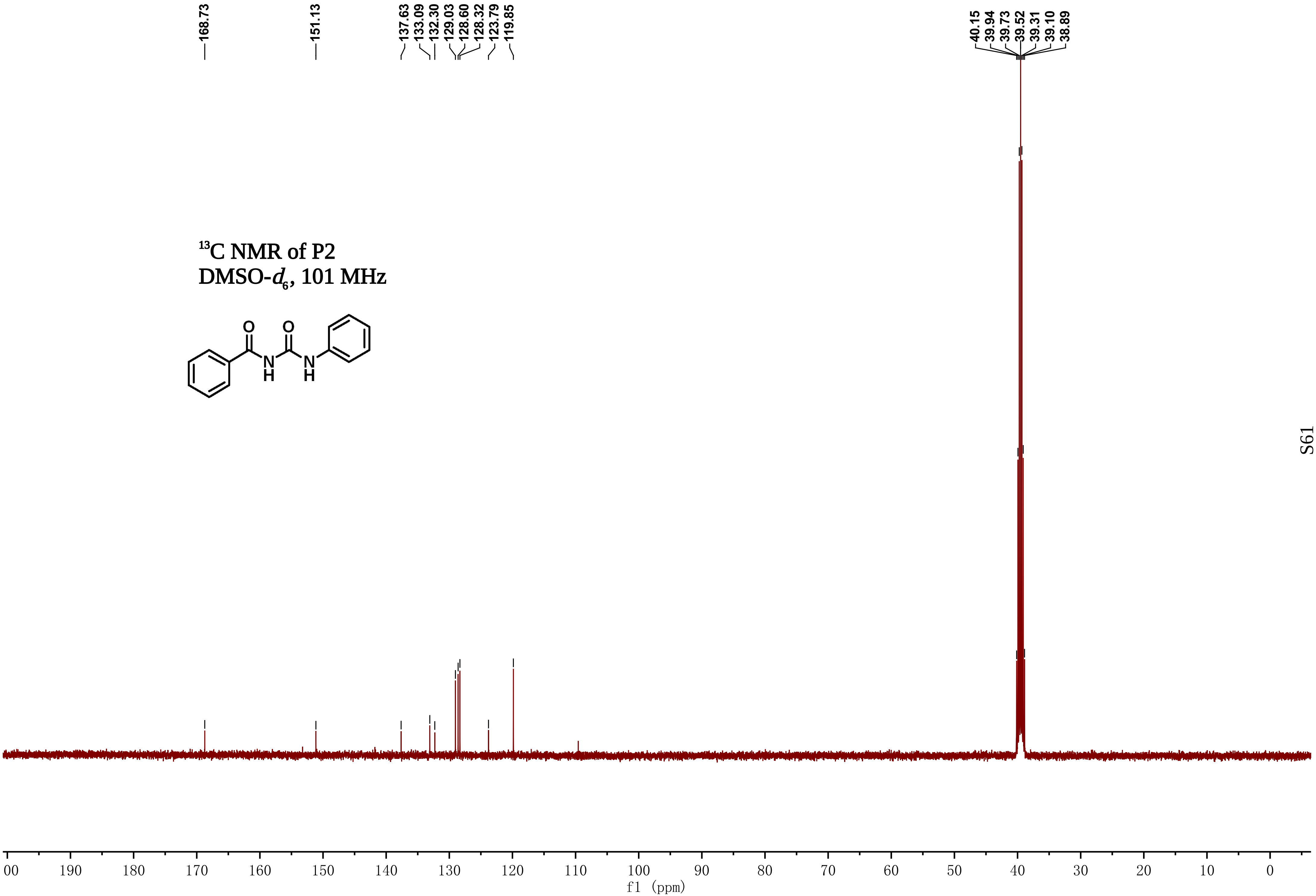
168.73
151.13
137.63
133.09
132.30
129.03
128.60
128.32
123.79
119.85
40.15
39.94
39.73
39.52
39.31
39.10
38.89
^{13}C NMR of P2
DMSO-d_6, 101 MHz
O
O
N
H
N
H
00
190
180
170
160
150
140
130
120
110
100
90
80
70
60
50
40
30
20
10
0
f1 (ppm)

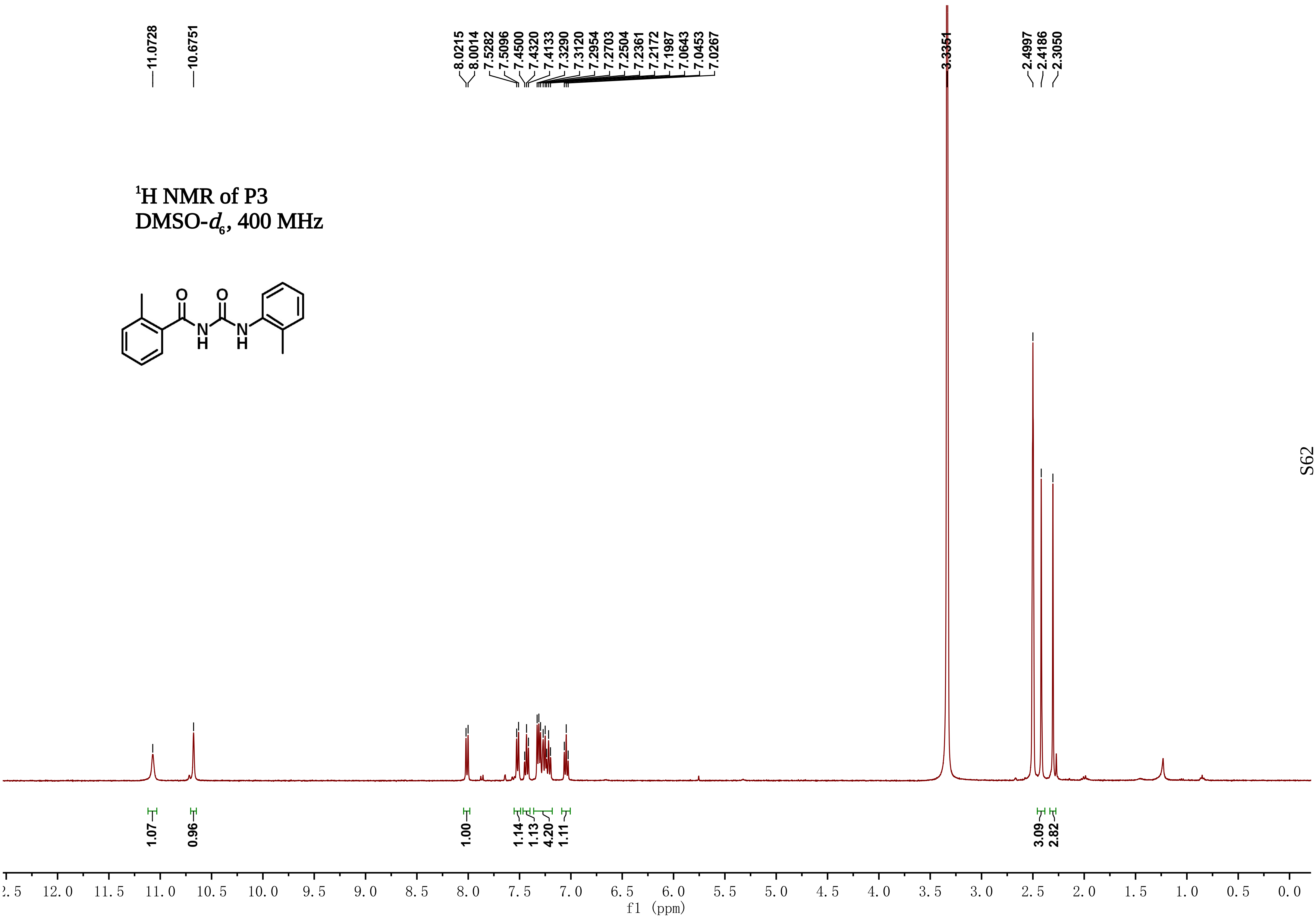

1H NMR of P3
DMSO-d_6, 400 MHz
11.0728
10.6751
8.0215
8.0014
7.5282
7.5096
7.4500
7.4320
7.4133
7.3290
7.3120
7.2954
7.2703
7.2504
7.2361
7.2172
7.1987
7.0643
7.0453
7.0267
3.3351
2.4997
2.4186
2.3050
1.07
0.96
1.00
1.14
1.13
4.20
1.11
3.09
2.82
12.5 12.0 11.5 11.0 10.5 10.0 9.5 9.0 8.5 8.0 7.5 7.0 6.5 6.0 5.5 5.0 4.5 4.0 3.5 3.0 2.5 2.0 1.5 1.0 0.5 0.0
f1 (ppm)

^{13}C NMR of P3
DMSO-d_6, 101 MHz

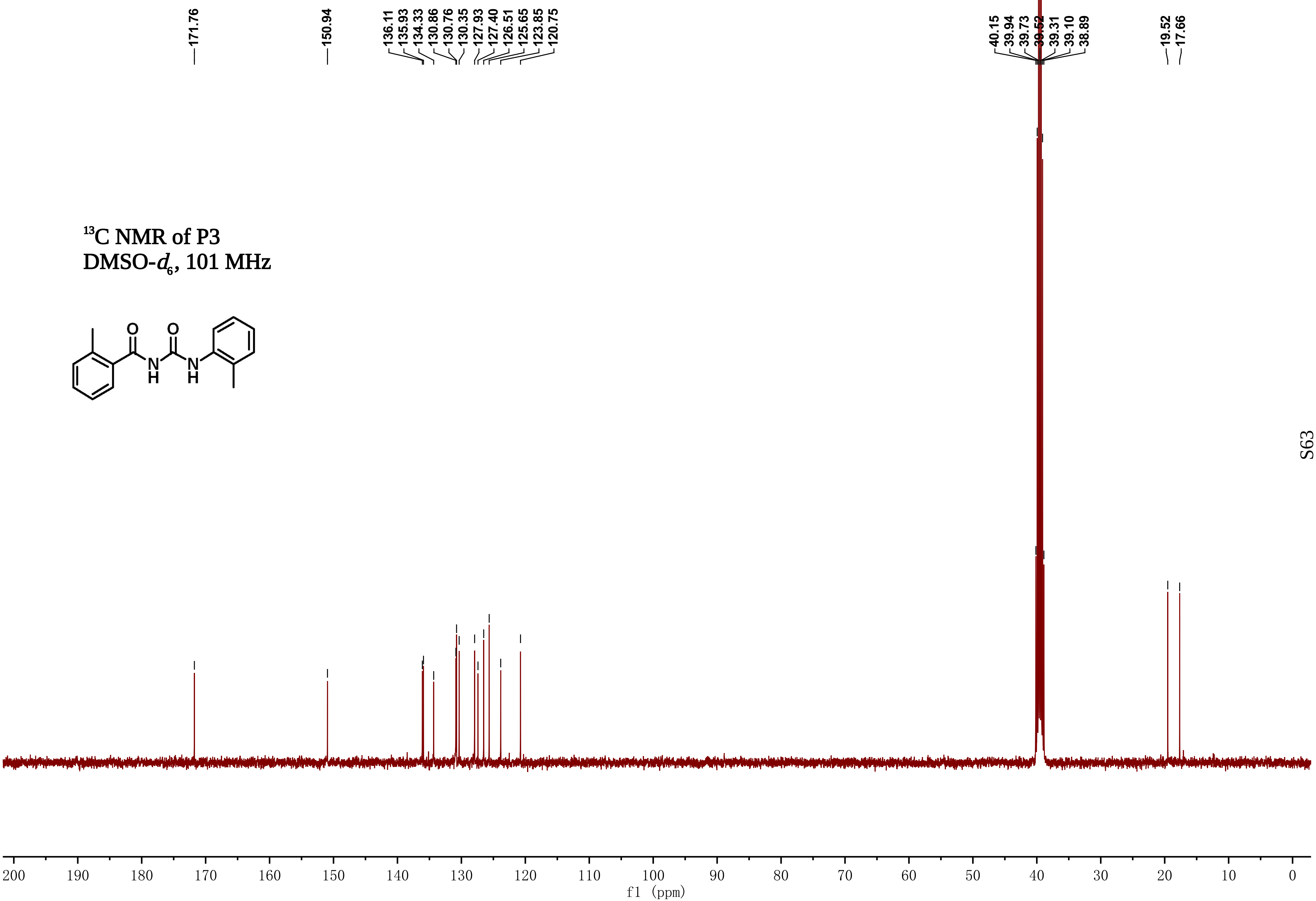

^{1}H NMR of P4
DMSO-d_6, 400 MHz

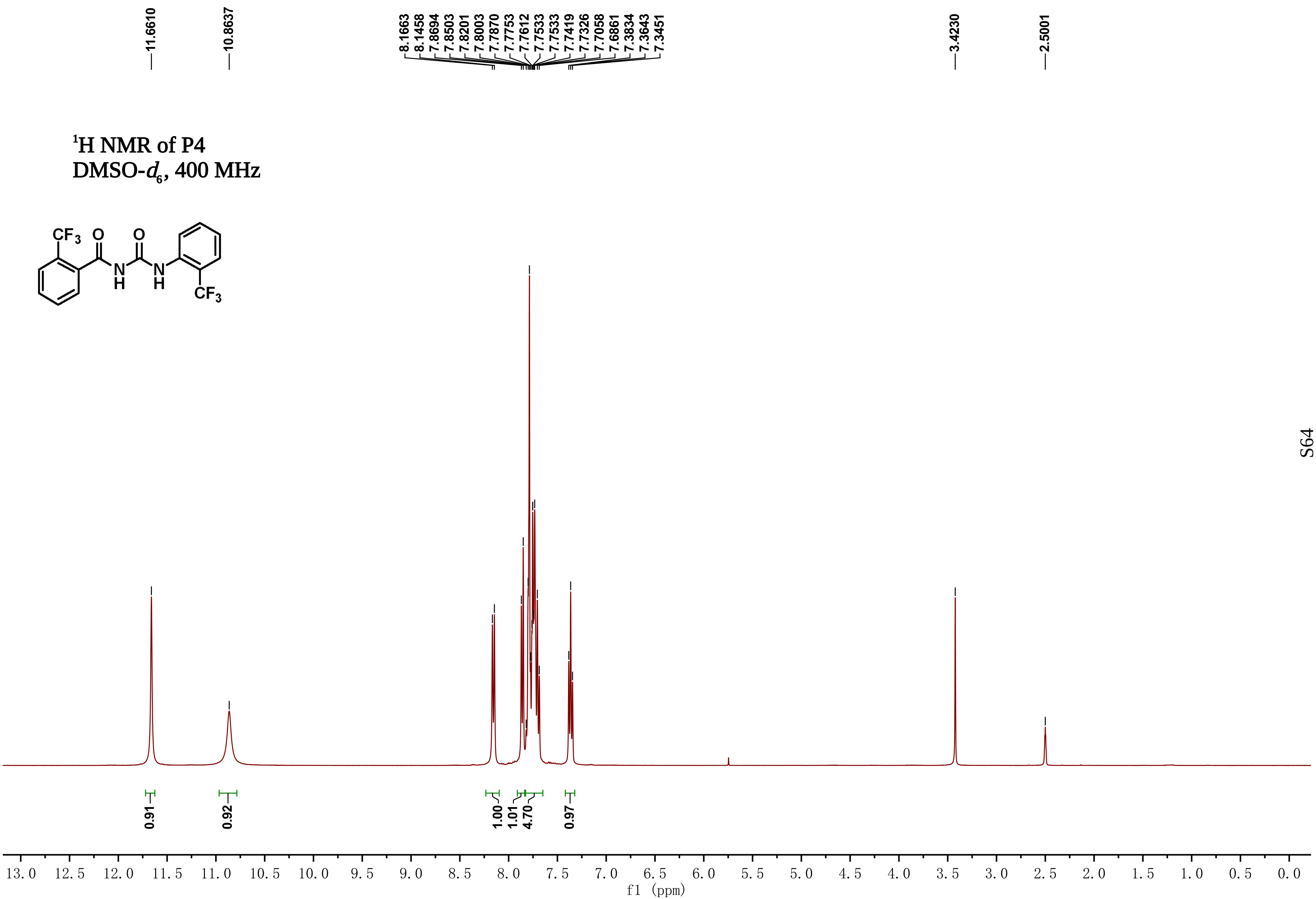

^{13}C NMR of P4
DMSO-d_6, 101 MHz

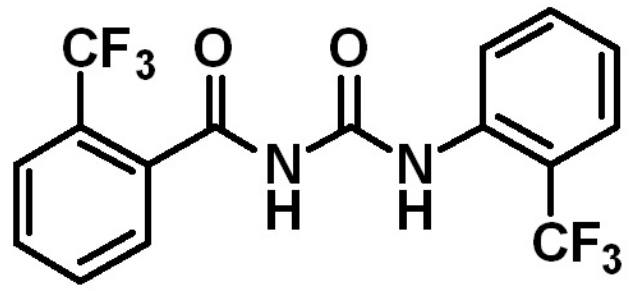

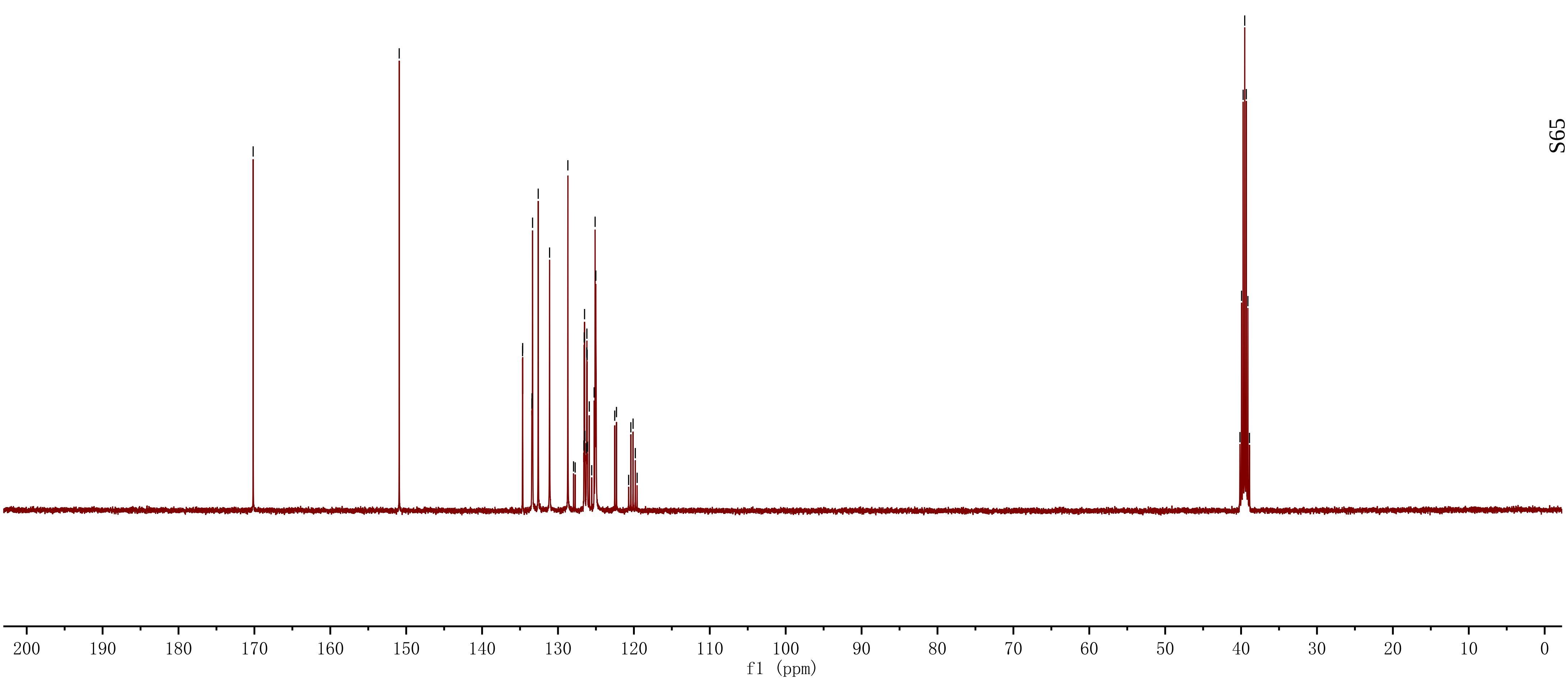

^{19}F NMR of P4
DMSO-d_6, 376 MHz

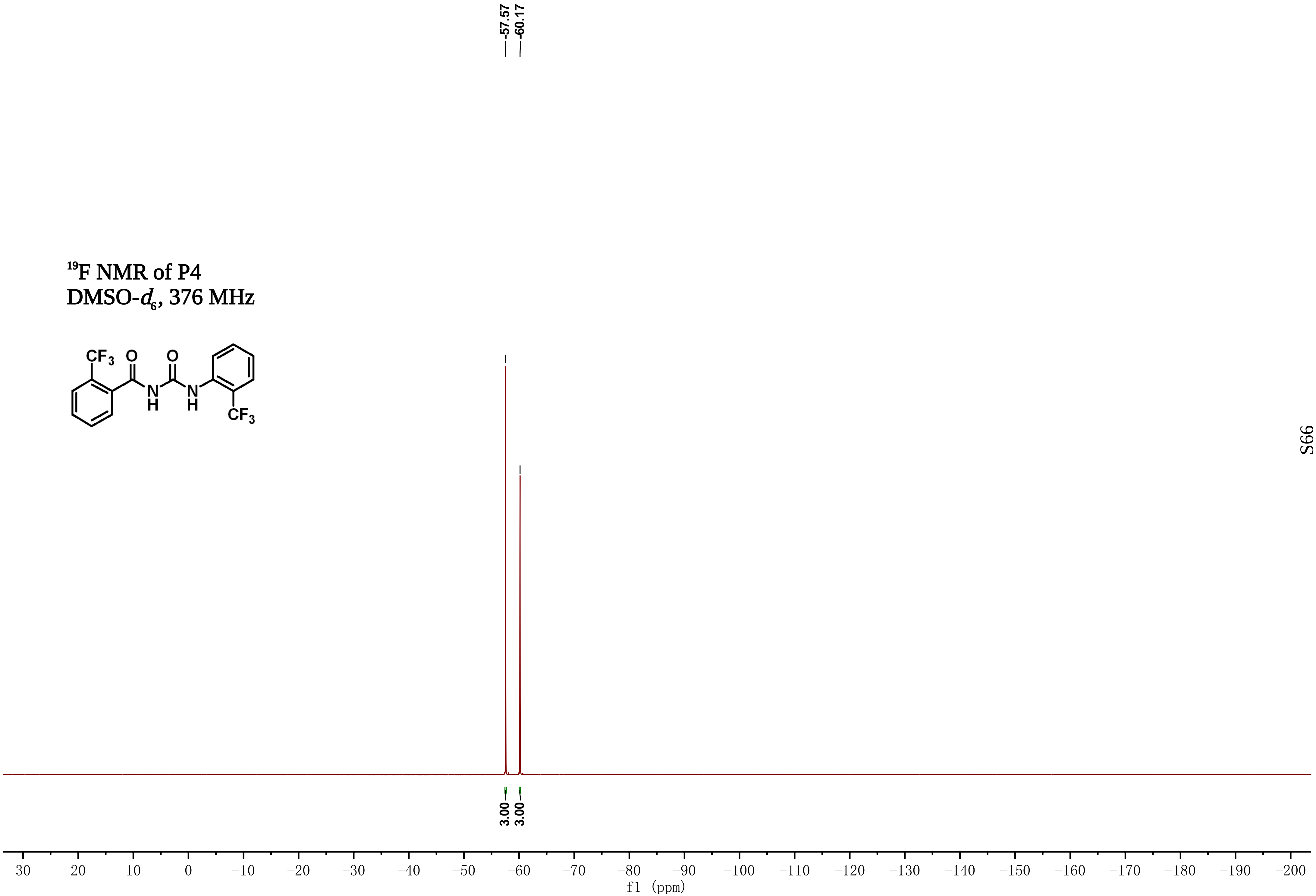

^{1}H NMR of P5
$CDCl_3$, 400 MHz

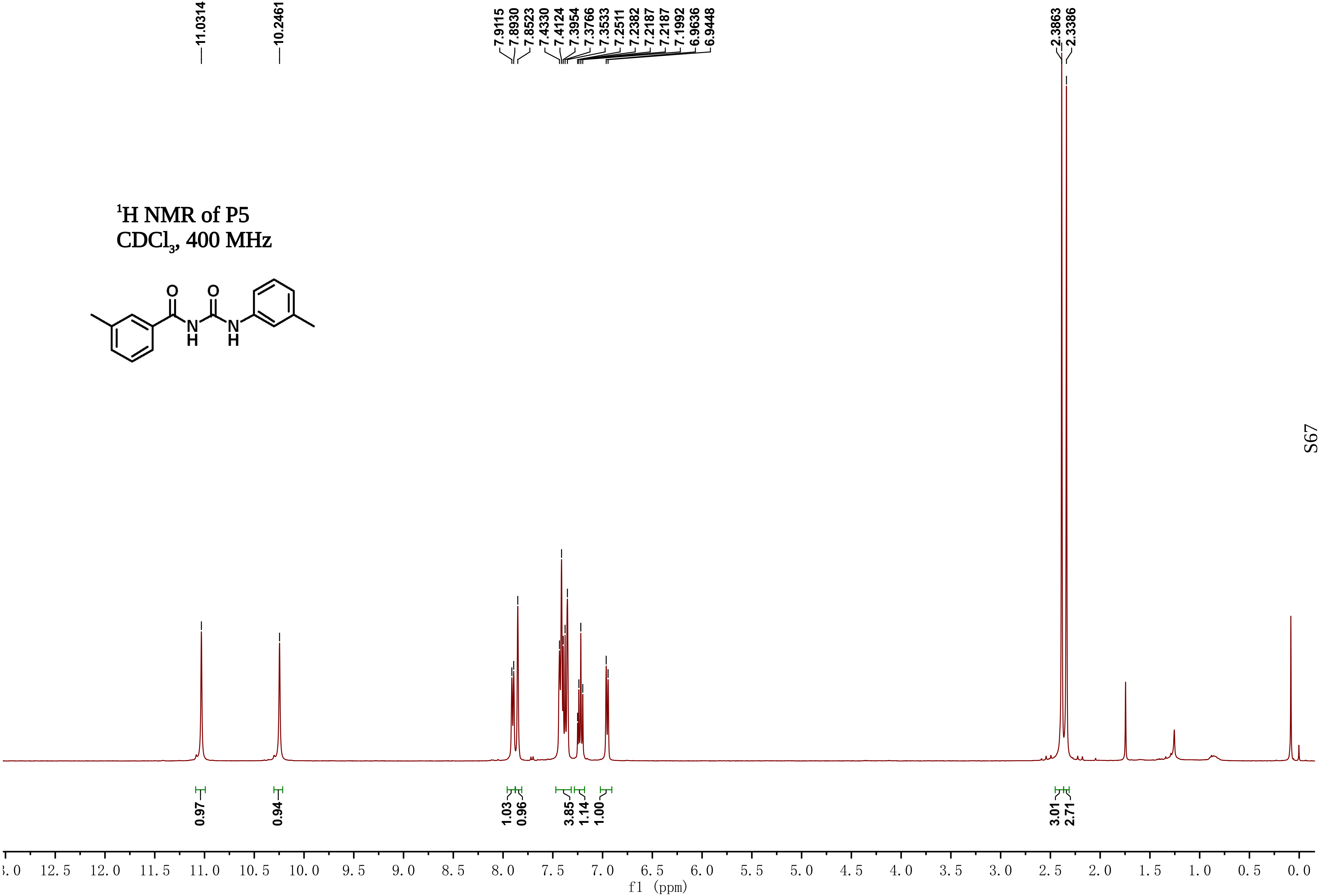

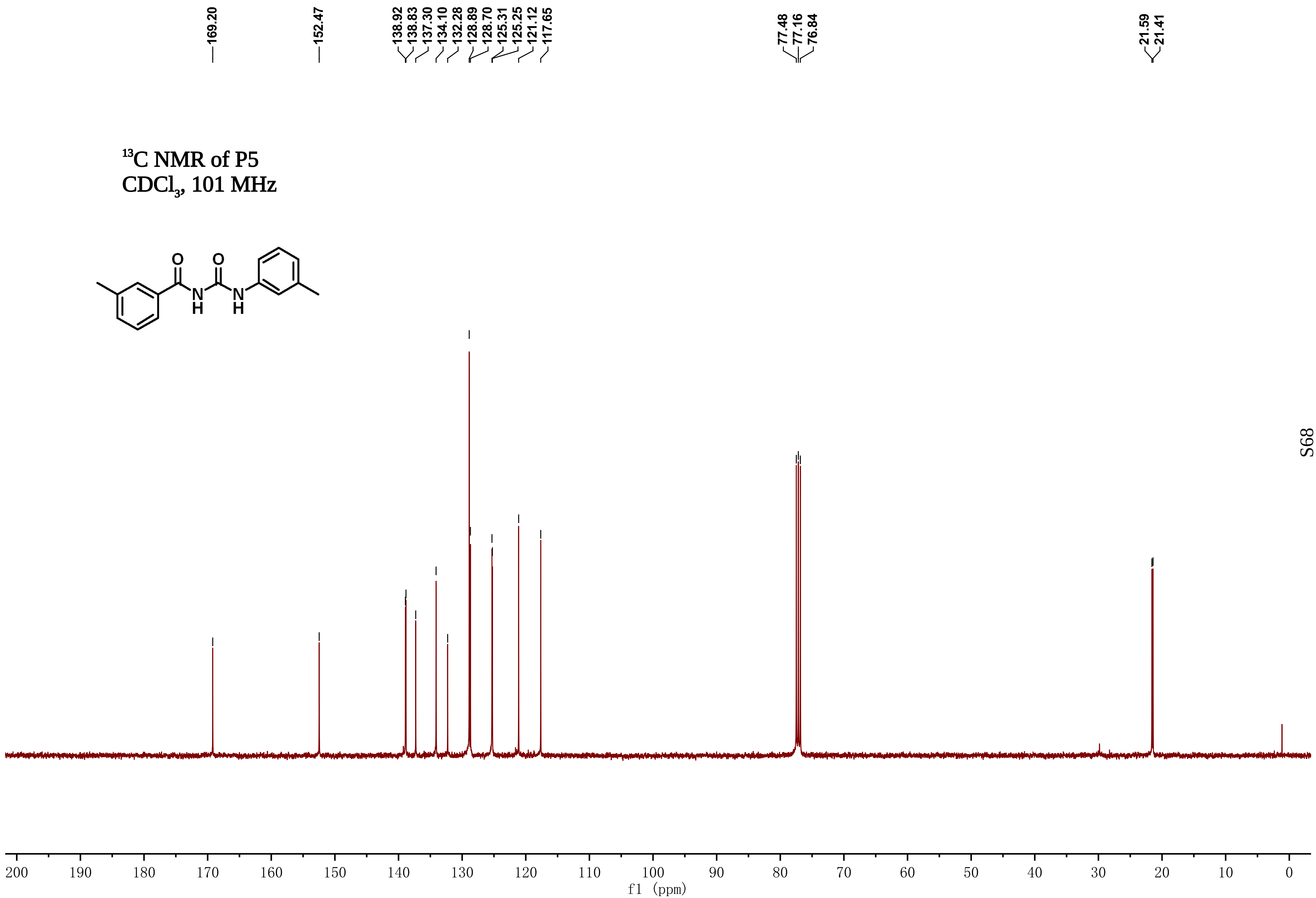
169.20
152.47
138.92
138.83
137.30
134.10
132.28
128.89
128.70
125.31
125.25
121.12
117.65
77.48
77.16
76.84
21.59
21.41
13C NMR of P5
CDCl3, 101 MHz
O
O
N
H
N
H
200 190 180 170 160 150 140 130 120 110 100 90 80 70 60 50 40 30 20 10 0
f1 (ppm)

^{1}H NMR of P6
DMSO-d_6, 400 MHz

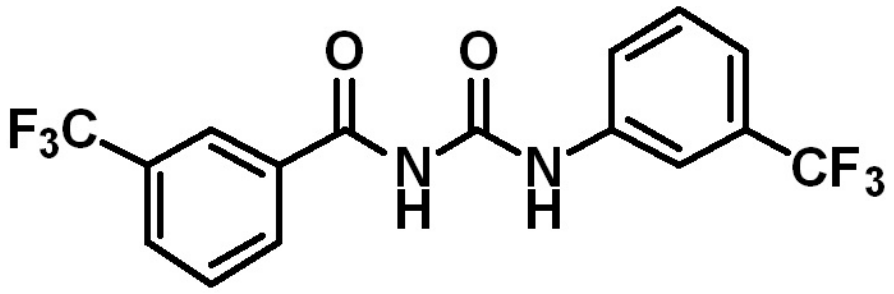

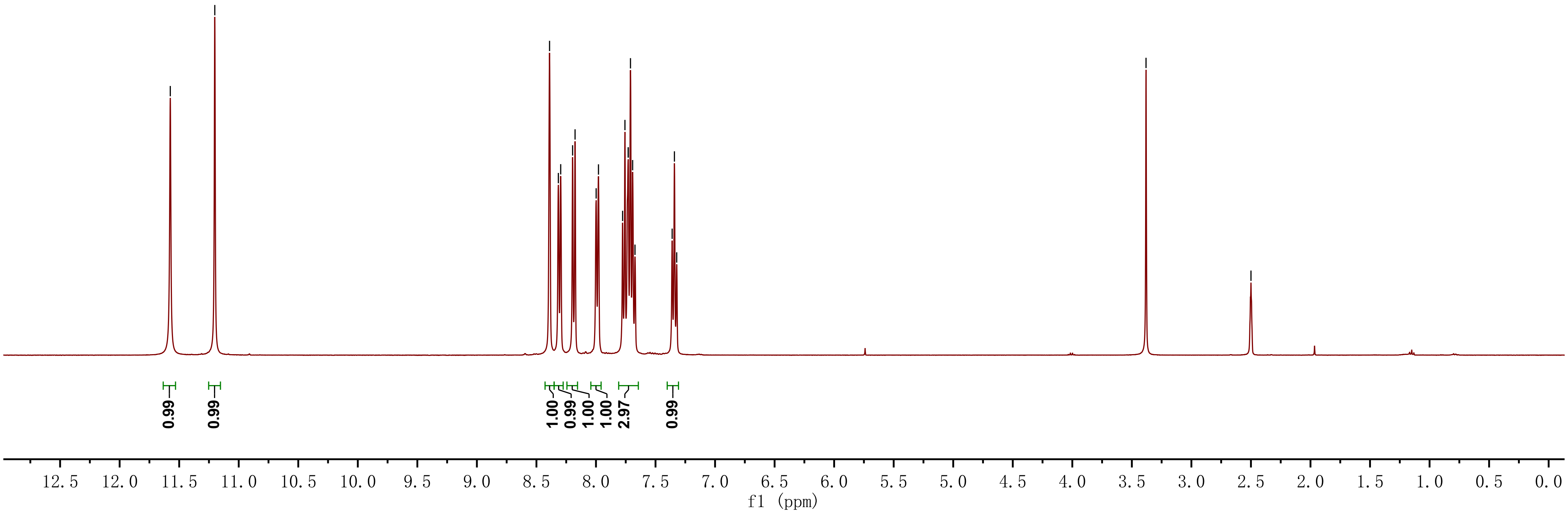

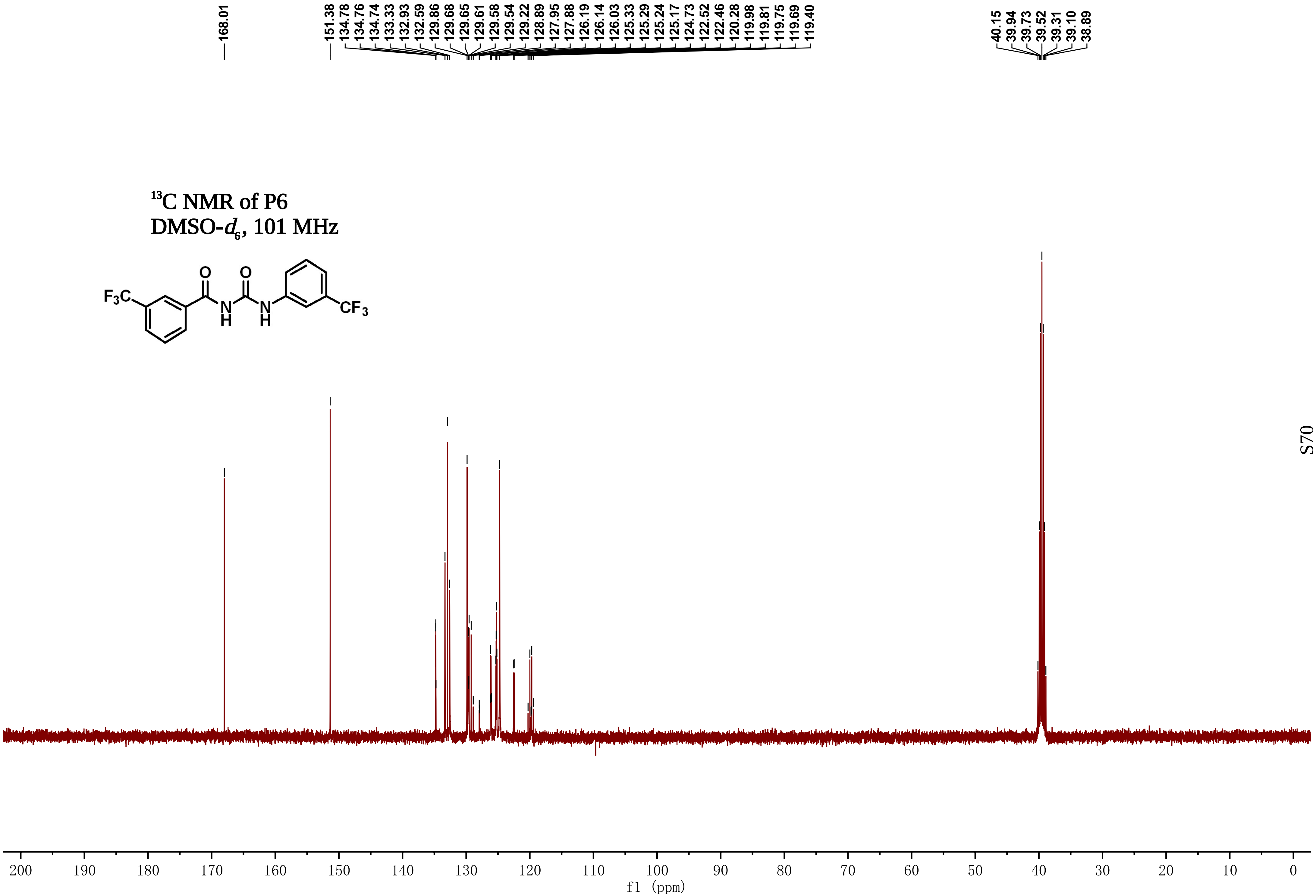
168.01
151.38
134.78
134.76
134.74
133.33
132.93
132.59
129.86
129.68
129.65
129.61
129.58
129.54
129.22
128.89
127.95
127.88
126.19
126.14
126.03
125.33
125.29
125.24
125.17
124.73
122.52
122.46
120.28
119.98
119.81
119.75
119.69
119.40
40.15
39.94
39.73
39.52
39.31
39.10
38.89
13C NMR of P6
DMSO-d6, 101 MHz
F3C
O
O
N
H
N
H
CF3
200 190 180 170 160 150 140 130 120 110 100 90 80 70 60 50 40 30 20 10 0
f1 (ppm)

^{19}F NMR of P6
DMSO-d_6, 376 MHz

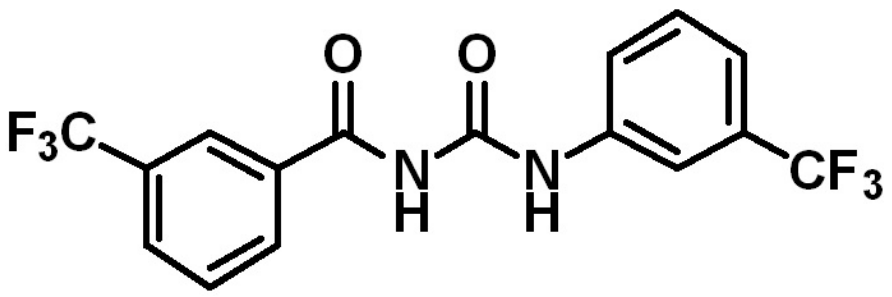

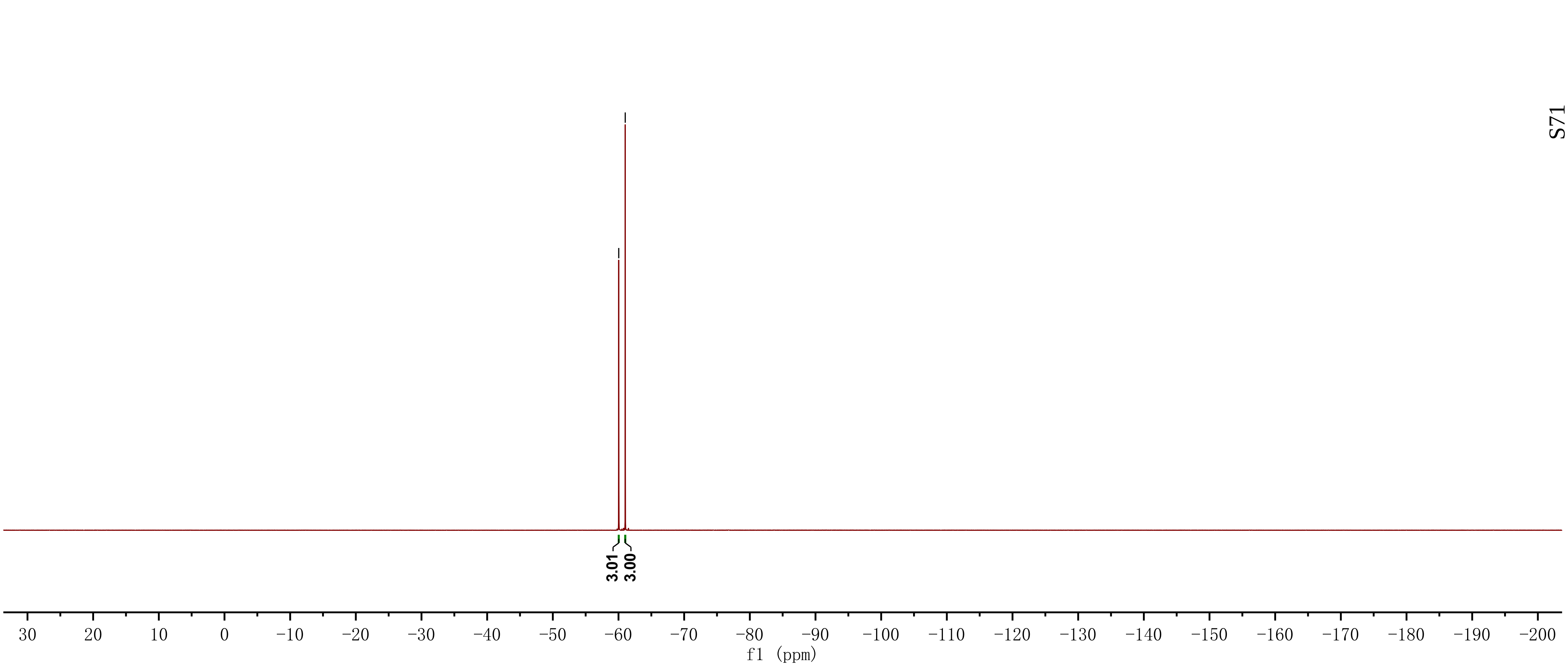

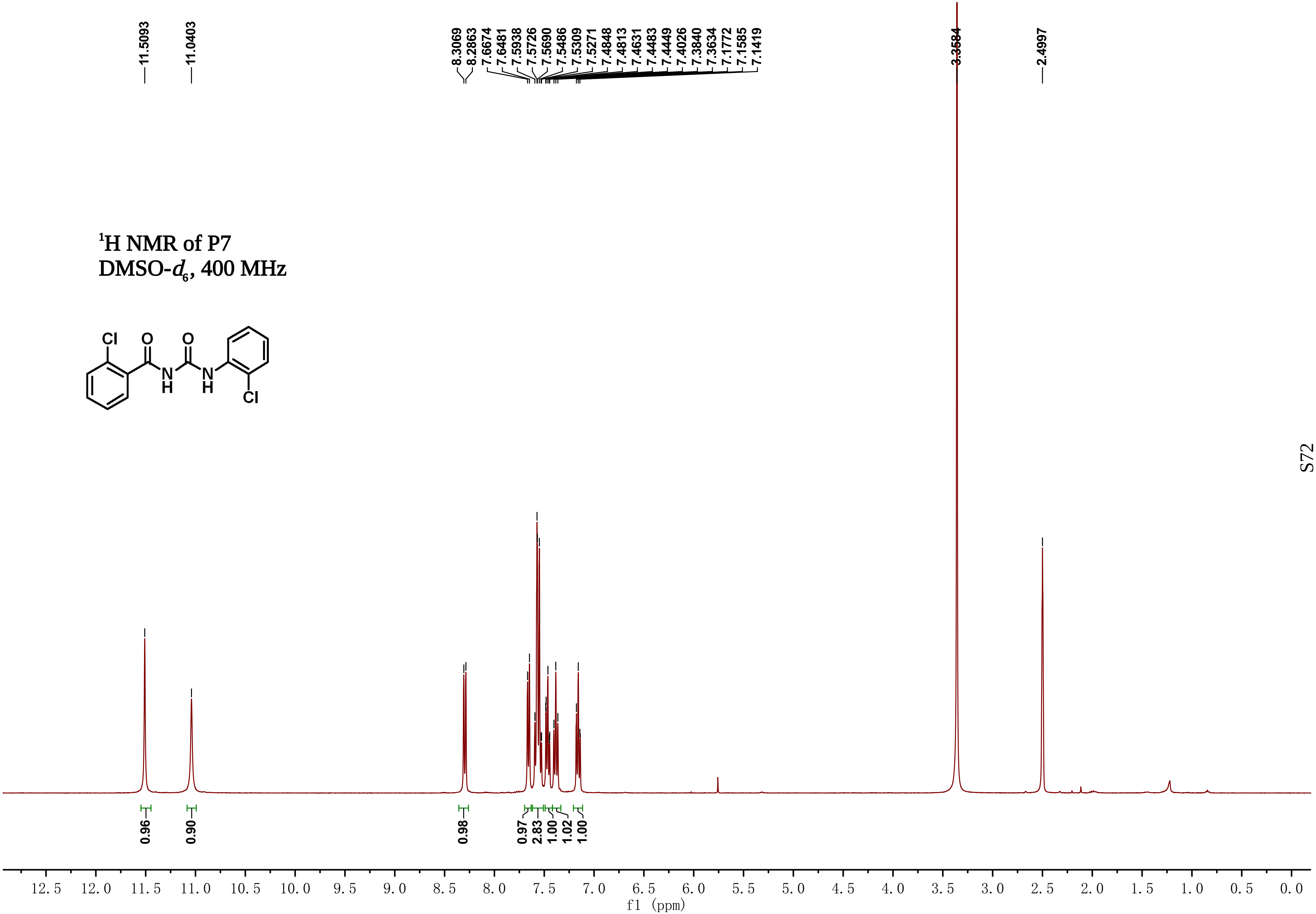

1H NMR of P7
DMSO-d6, 400 MHz
Cl
O
O
N
H
N
H
Cl
11.5093
11.0403
8.3069
8.2863
7.6674
7.6481
7.5938
7.5726
7.5690
7.5486
7.5309
7.5271
7.4848
7.4813
7.4631
7.4483
7.4449
7.4026
7.3840
7.3634
7.1772
7.1585
7.1419
3.3584
2.4997
0.96
0.90
0.98
0.97
2.83
1.00
1.02
1.00
12.5 12.0 11.5 11.0 10.5 10.0 9.5 9.0 8.5 8.0 7.5 7.0 6.5 6.0 5.5 5.0 4.5 4.0 3.5 3.0 2.5 2.0 1.5 1.0 0.5 0.0
f1 (ppm)

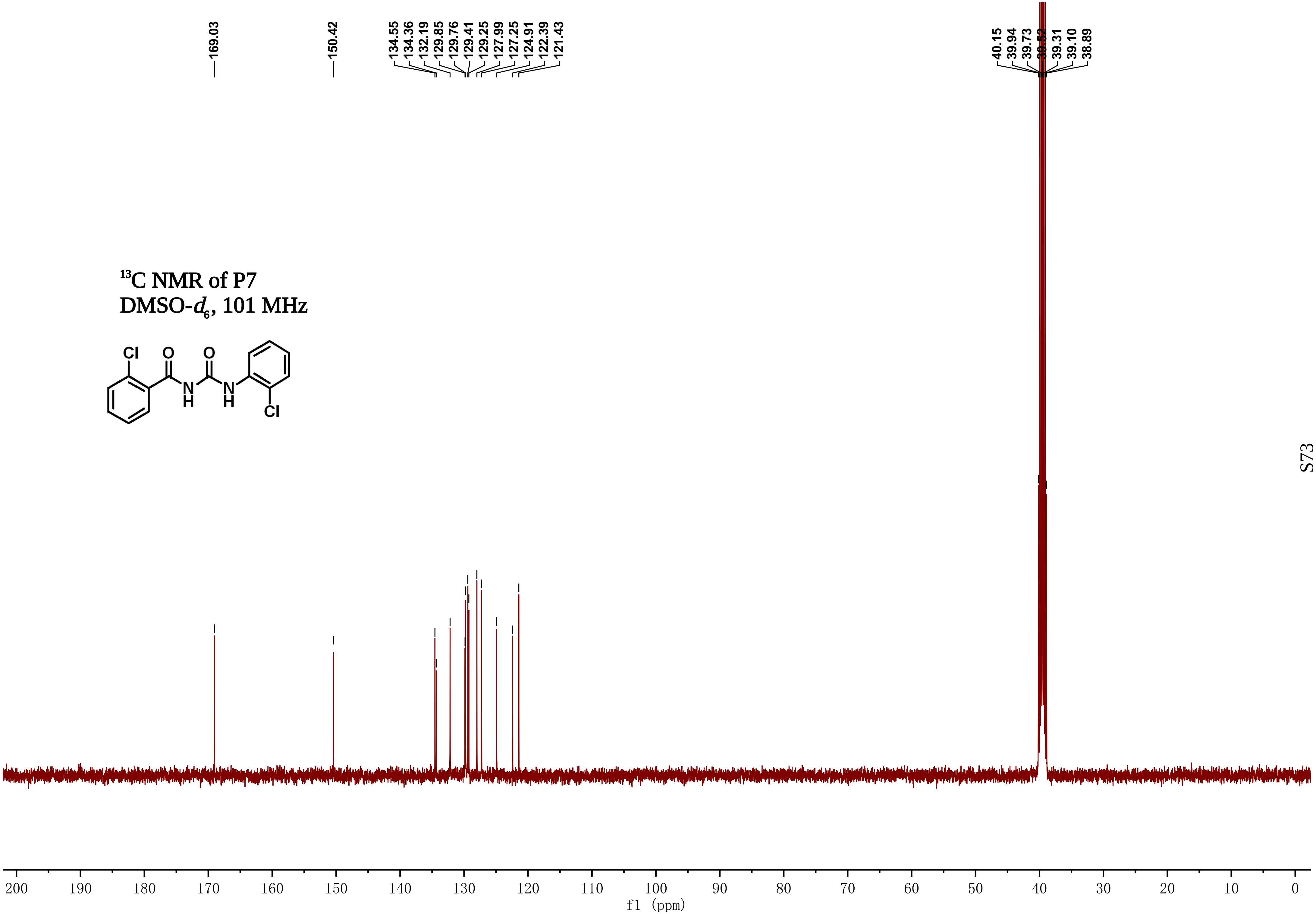
169.03
150.42
134.55
134.36
132.19
129.85
129.76
129.41
129.25
127.99
127.25
124.91
122.39
121.43
40.15
39.94
39.73
39.52
39.31
39.10
38.89
13C NMR of P7
DMSO-d6, 101 MHz
Cl
O
O
N
H
N
H
Cl
200
190
180
170
160
150
140
130
120
110
100
90
80
70
60
50
40
30
20
10
0
f1 (ppm)

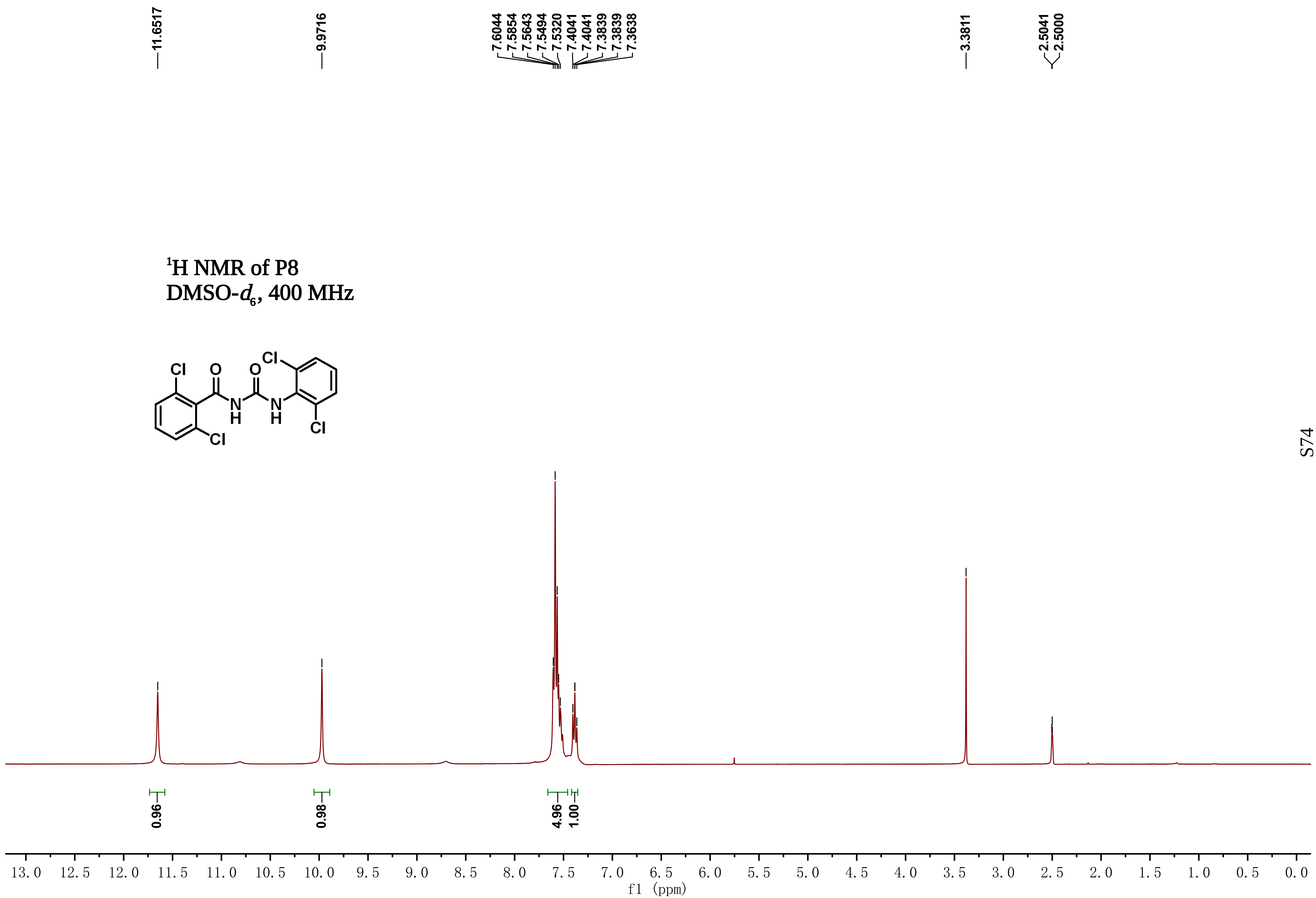

11.6517
9.9716
7.6044
7.5854
7.5643
7.5494
7.5320
7.4041
7.4041
7.3839
7.3839
7.3638
3.3811
2.5041
2.5000
^{1}H NMR of P8
DMSO-d_6, 400 MHz
Cl
O
O
Cl
N
H
N
H
Cl
Cl
0.96
0.98
4.96
1.00
13.0 12.5 12.0 11.5 11.0 10.5 10.0 9.5 9.0 8.5 8.0 7.5 7.0 6.5 6.0 5.5 5.0 4.5 4.0 3.5 3.0 2.5 2.0 1.5 1.0 0.5 0.0
f1 (ppm)

^{13}C NMR of P8
DMSO-d_6, 101 MHz

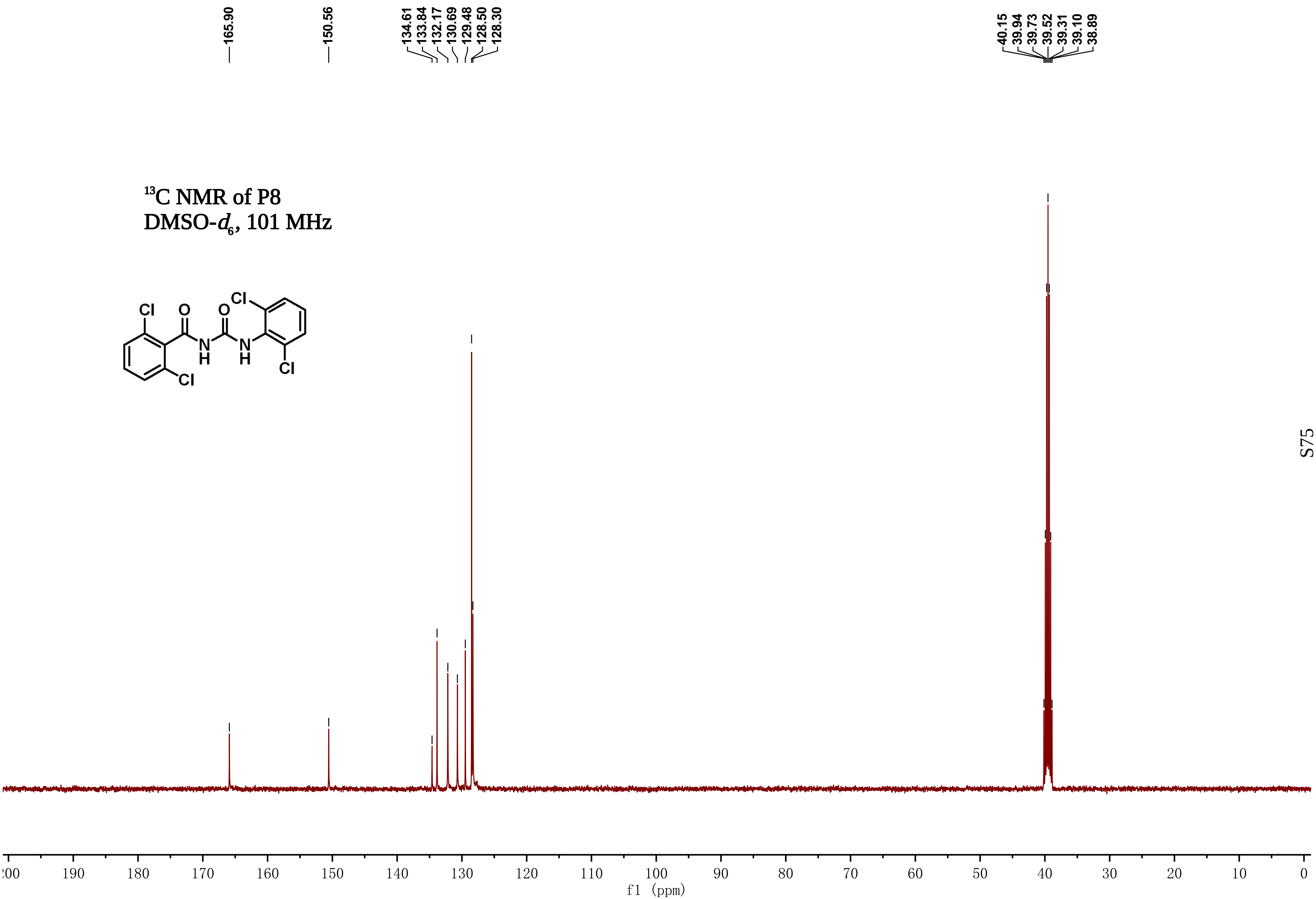

^{1}H NMR of P9
DMSO-d_6, 400 MHz

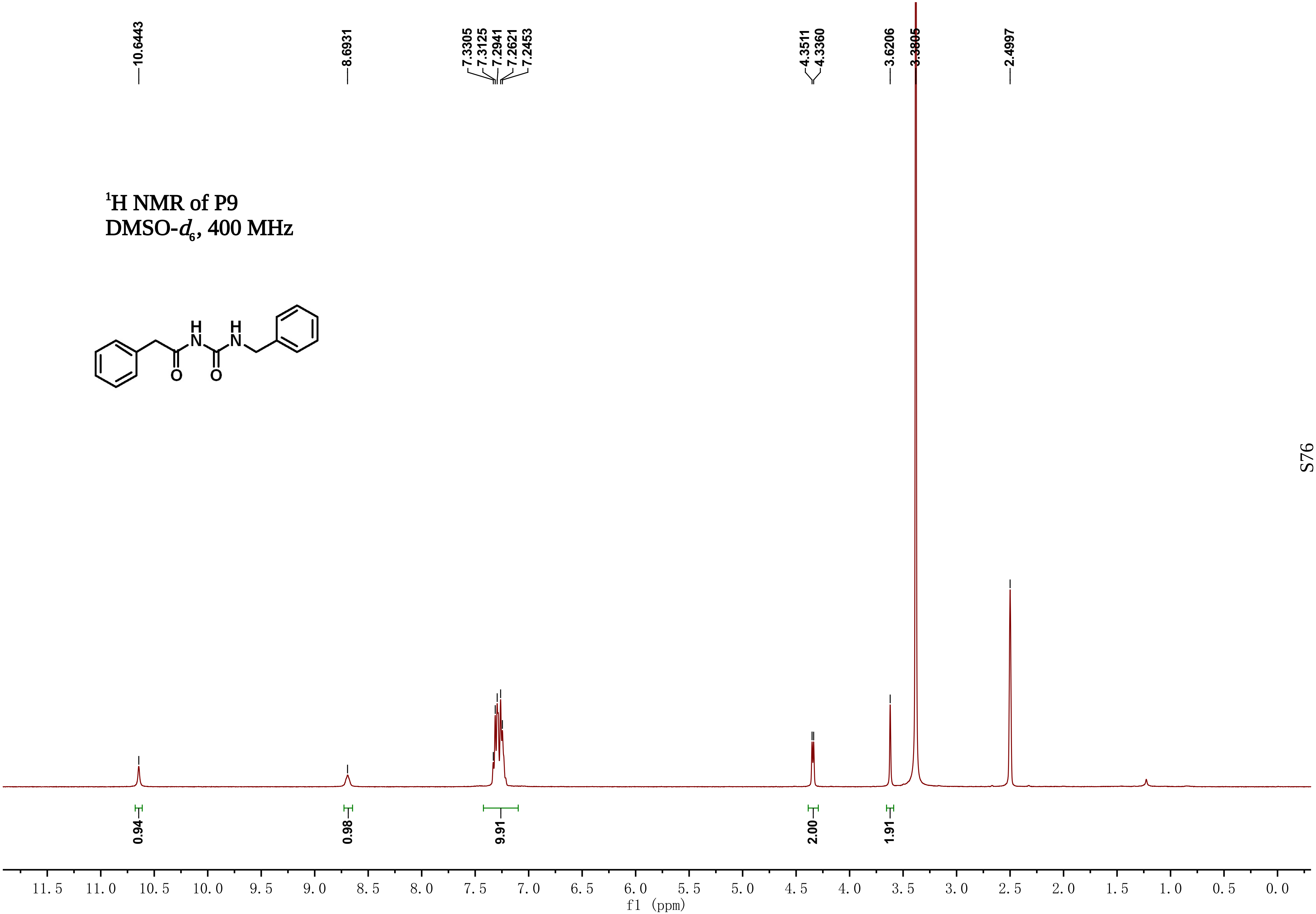

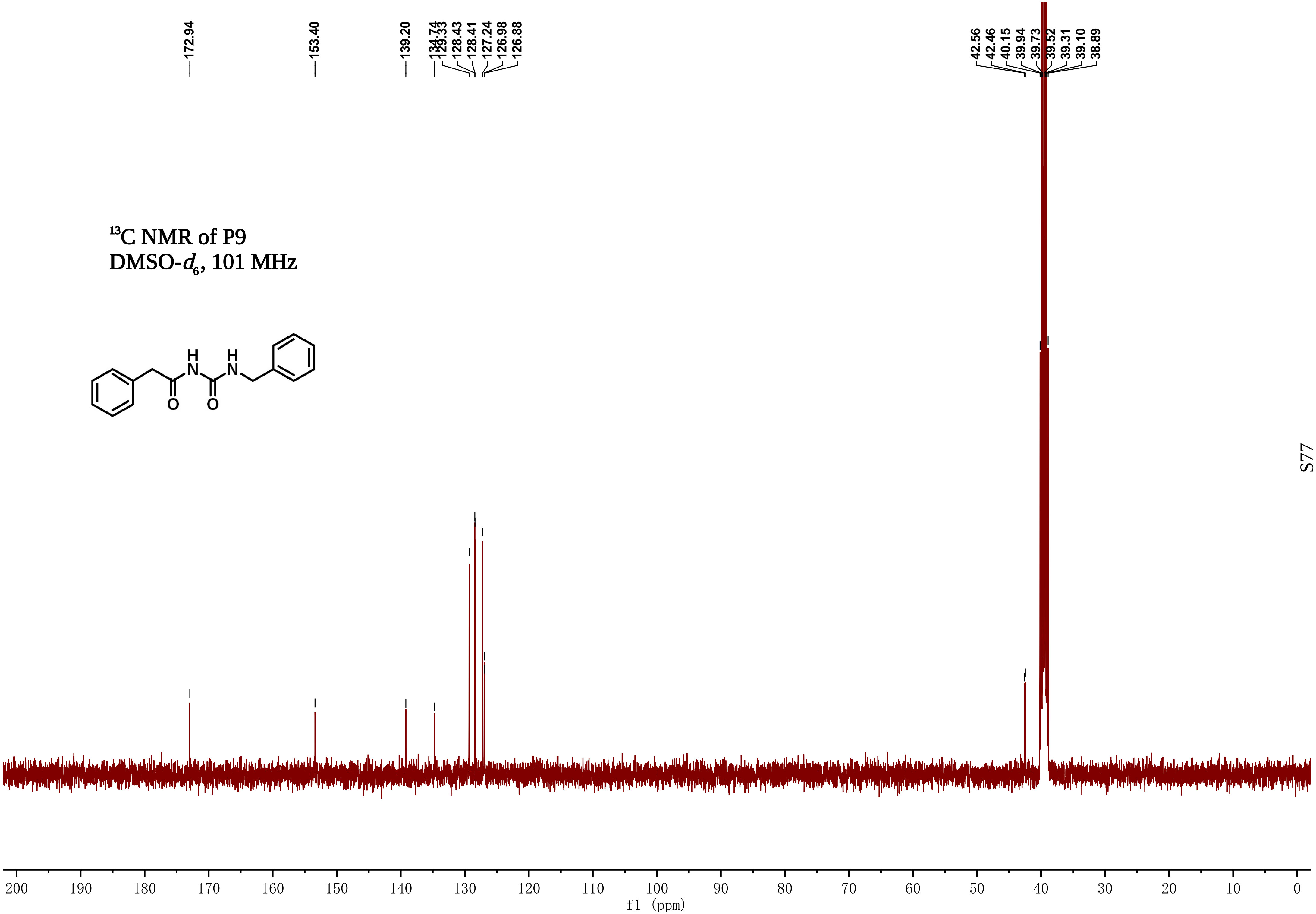
172.94
153.40
139.20
134.74
129.33
128.43
128.41
127.24
126.98
126.88
42.56
42.46
40.15
39.94
39.73
39.52
39.31
39.10
38.89
13C NMR of P9
DMSO-d6, 101 MHz
H
N
H
N
O
O
200
190
180
170
160
150
140
130
120
110
100
90
80
70
60
50
40
30
20
10
0
f1 (ppm)

^{1}H NMR of P10
$CDCl_3$, 400 MHz

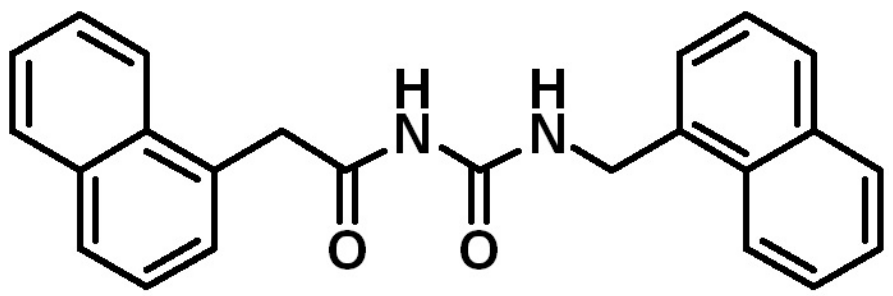

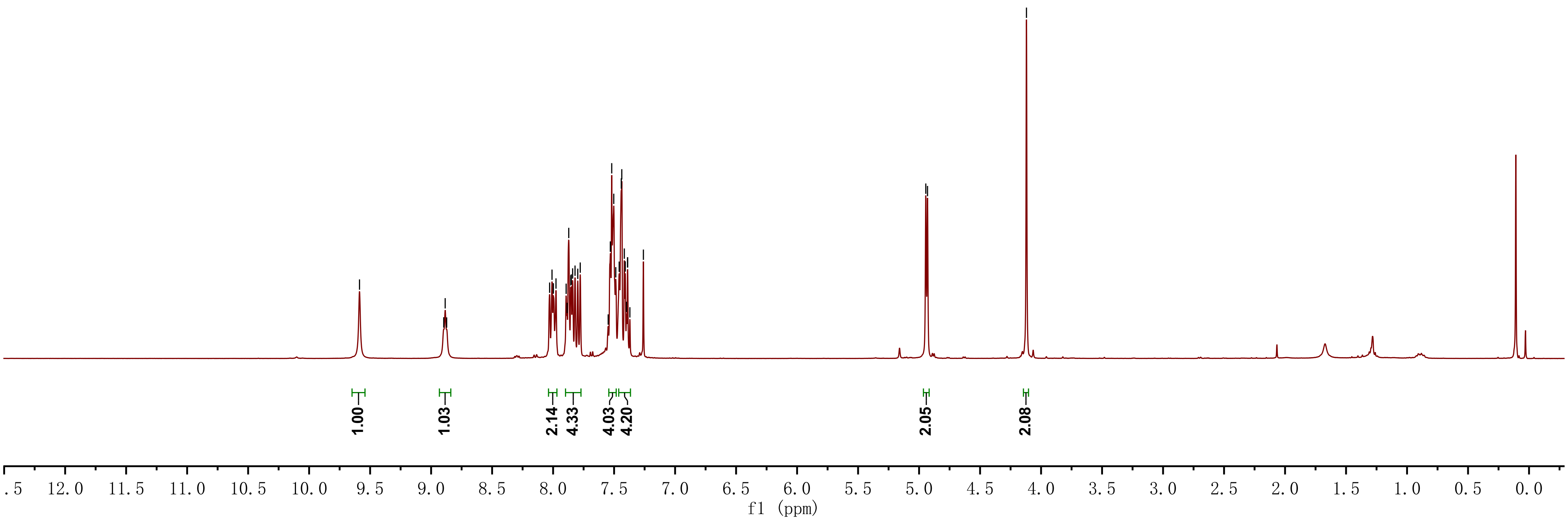

^{13}C NMR of P10
$CDCl_3$, 101 MHz

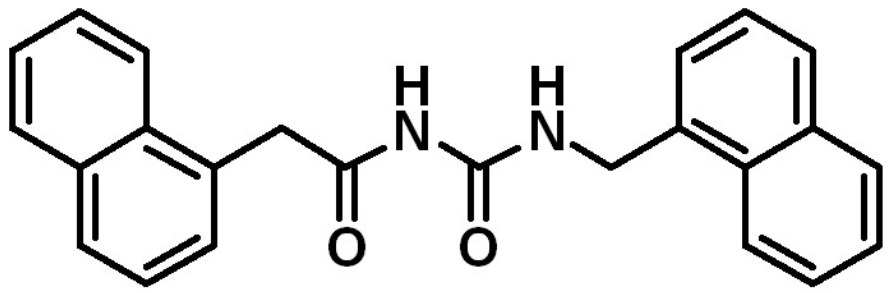

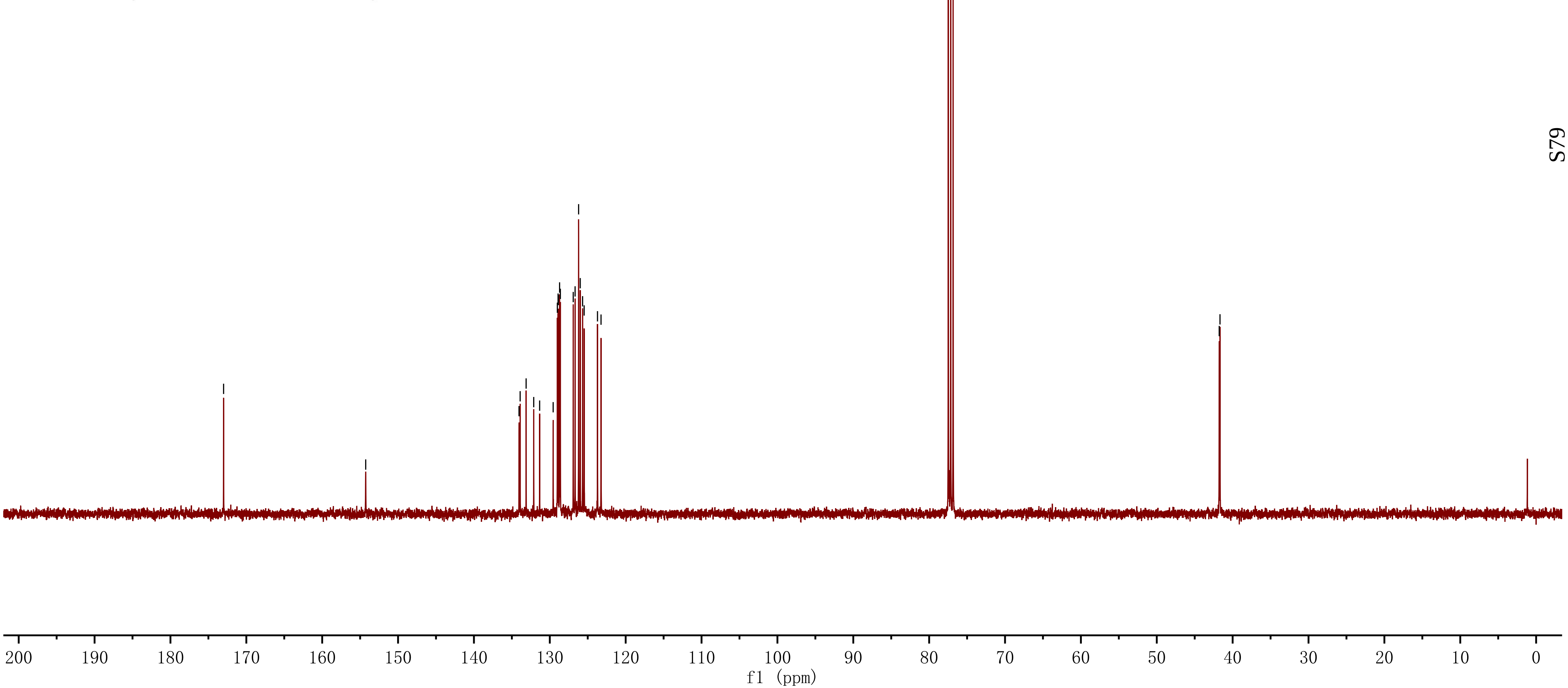

^{1}H NMR of P11
$CDCl_3$, 400 MHz

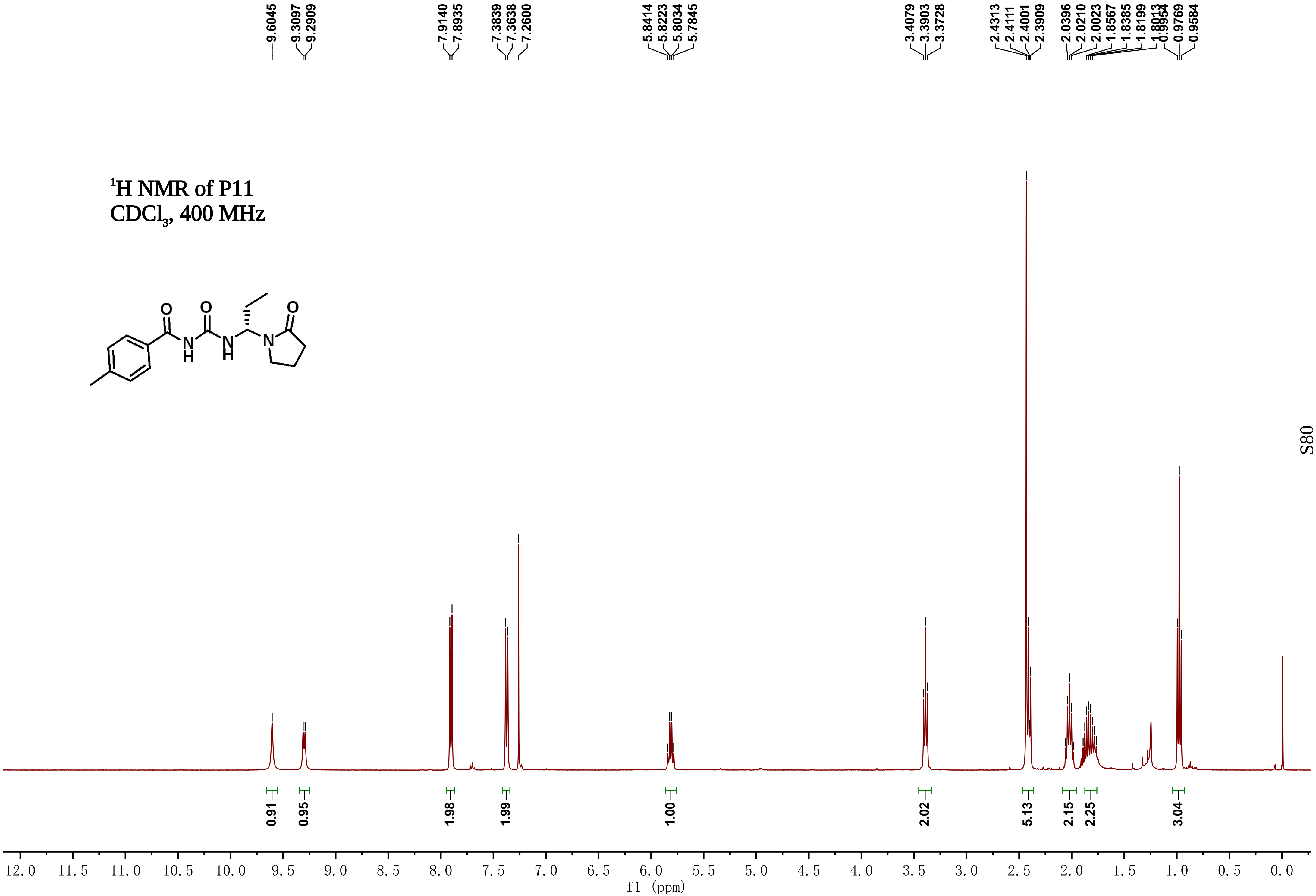

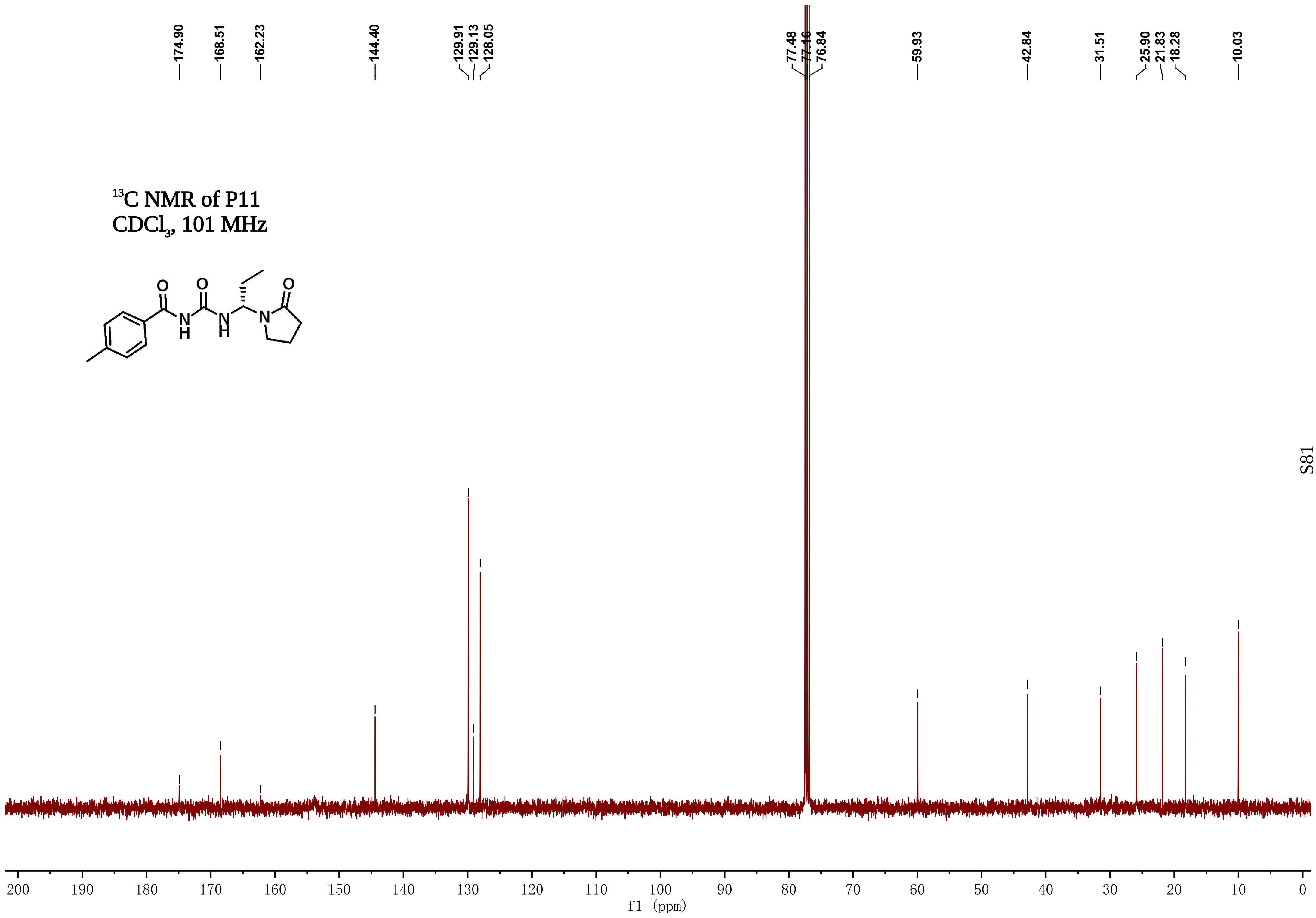

174.90
168.51
162.23
144.40
129.91
129.13
128.05
77.48
77.16
76.84
59.93
42.84
31.51
25.90
21.83
18.28
10.03
^{13}C NMR of P11
$CDCl_3$, 101 MHz
O
O
O
N
H
N
H
N
200
190
180
170
160
150
140
130
120
110
100
90
80
70
60
50
40
30
20
10
0
f1 (ppm)

^{1}H NMR of P12
$CDCl_3$, 400 MHz

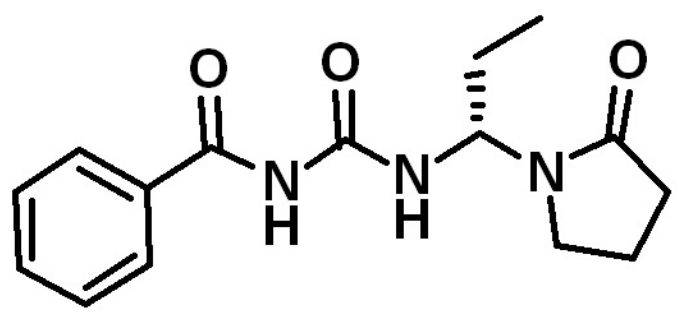

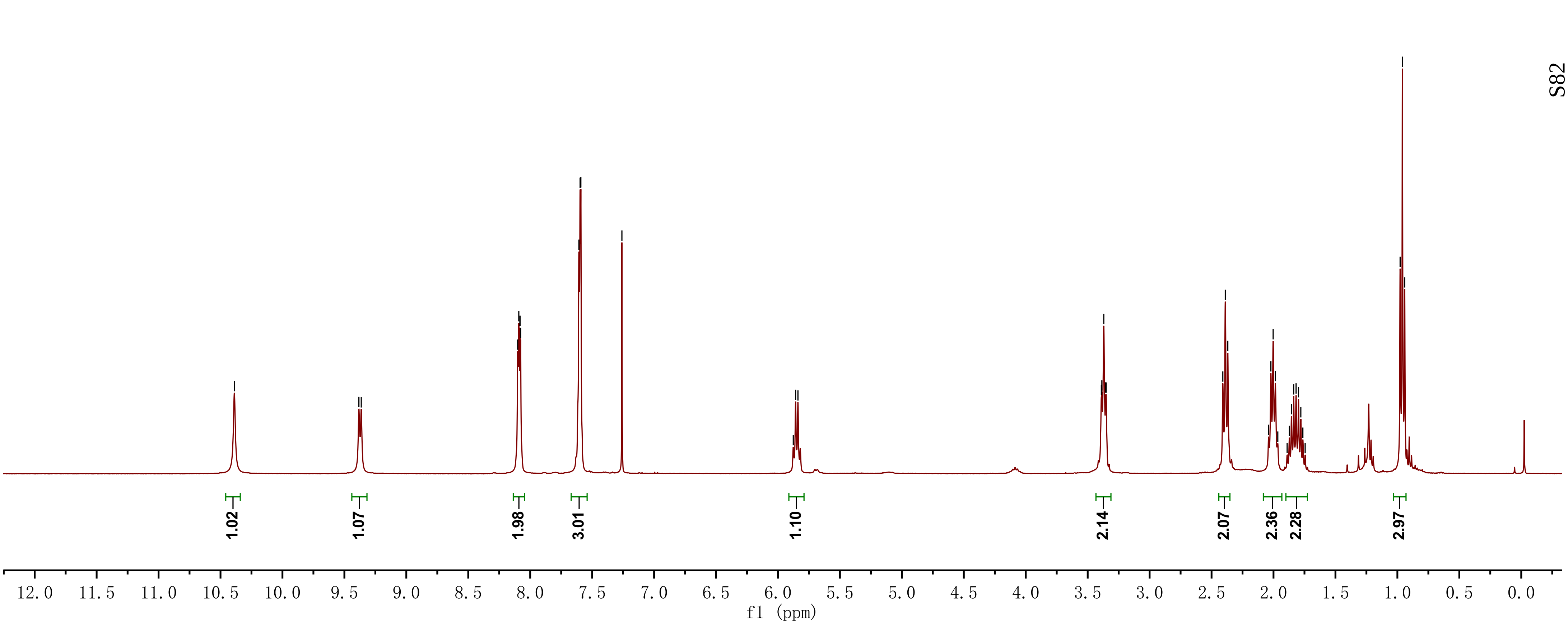

^{13}C NMR of P12
$CDCl_3$, 101 MHz

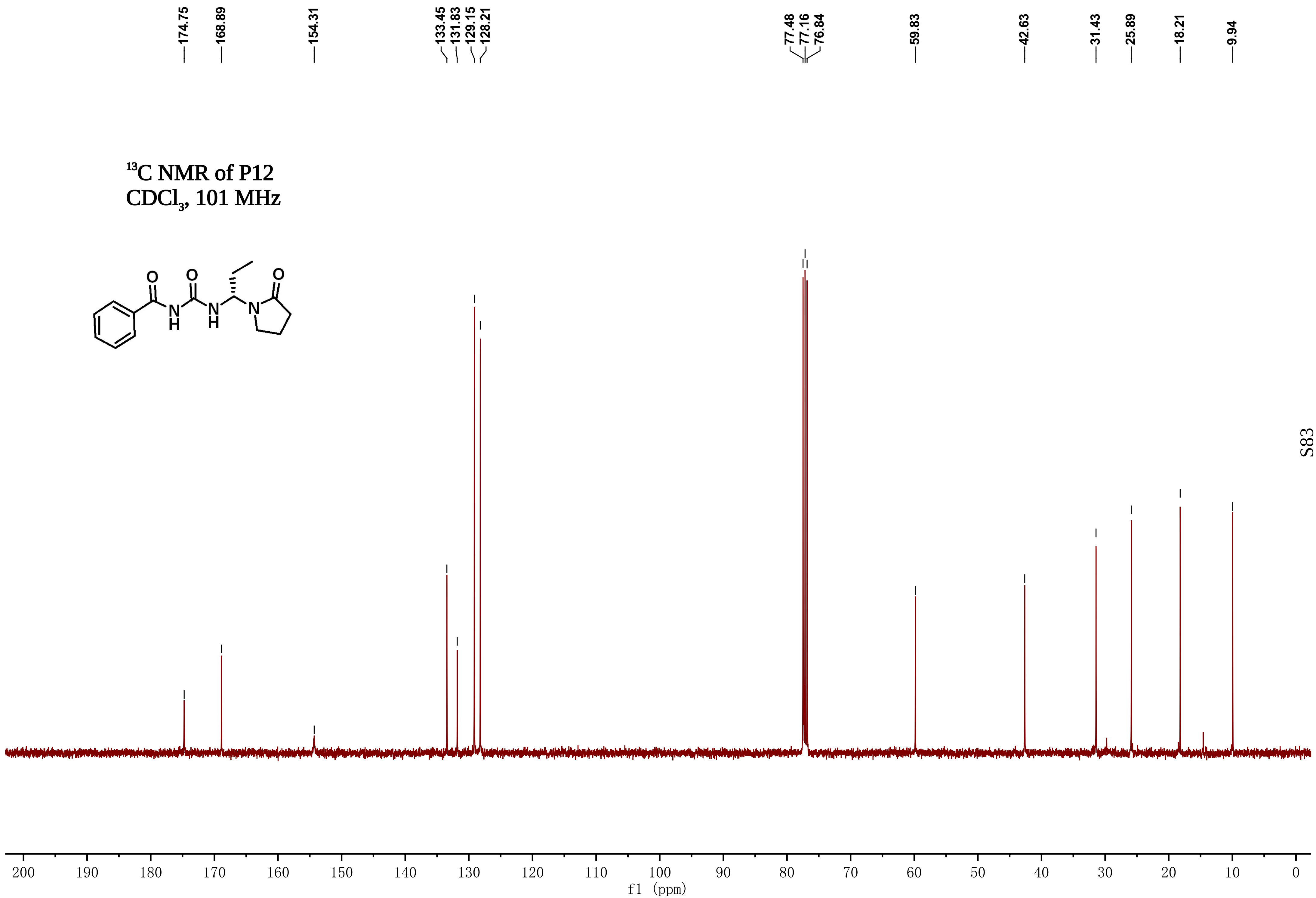

^{1}H NMR of P13
$CDCl_3$, 400 MHz

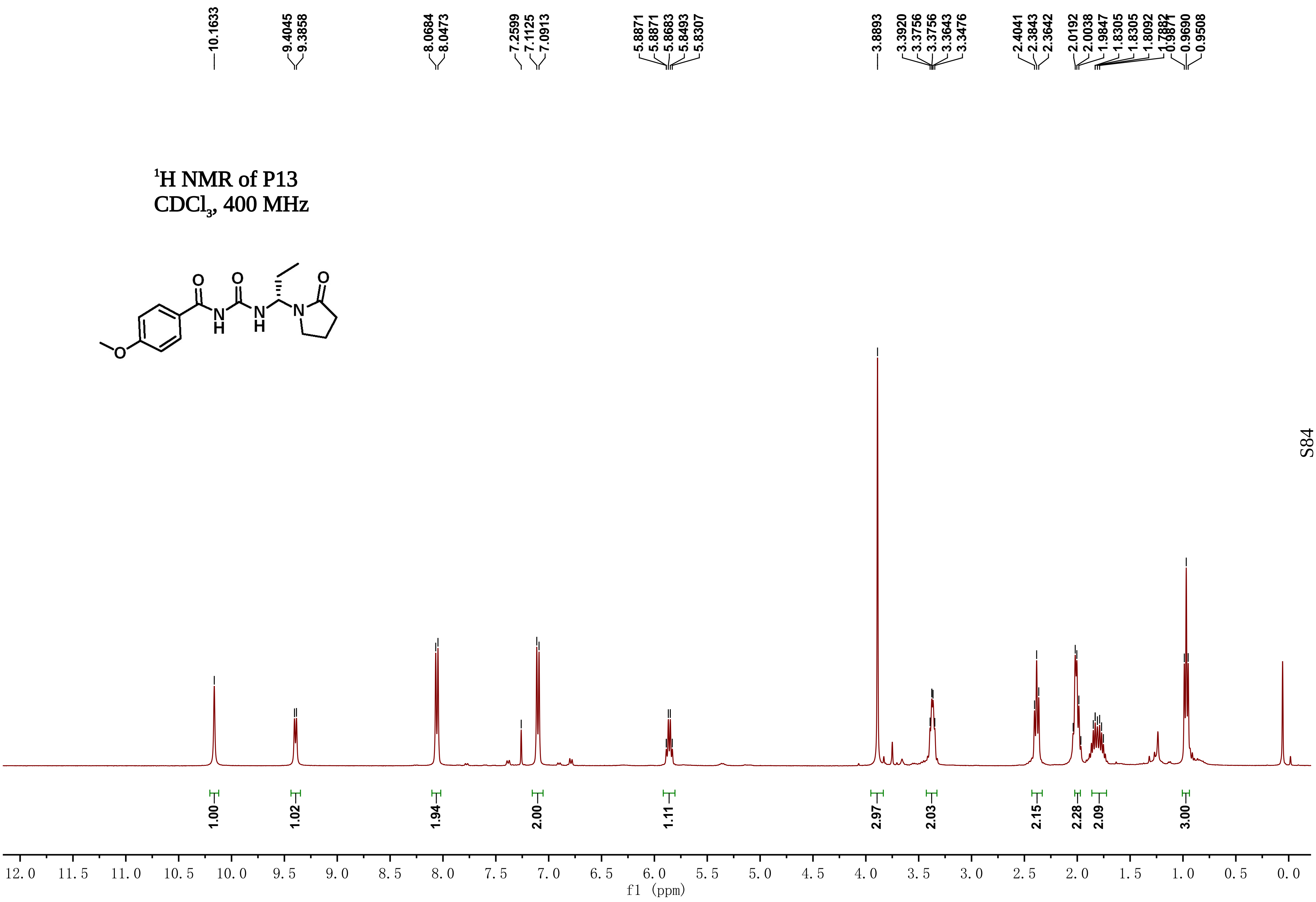

^{13}C NMR of P13
$CDCl_3$, 101 MHz

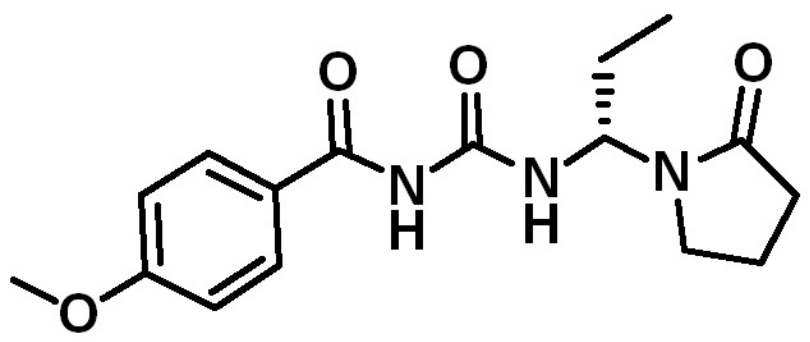

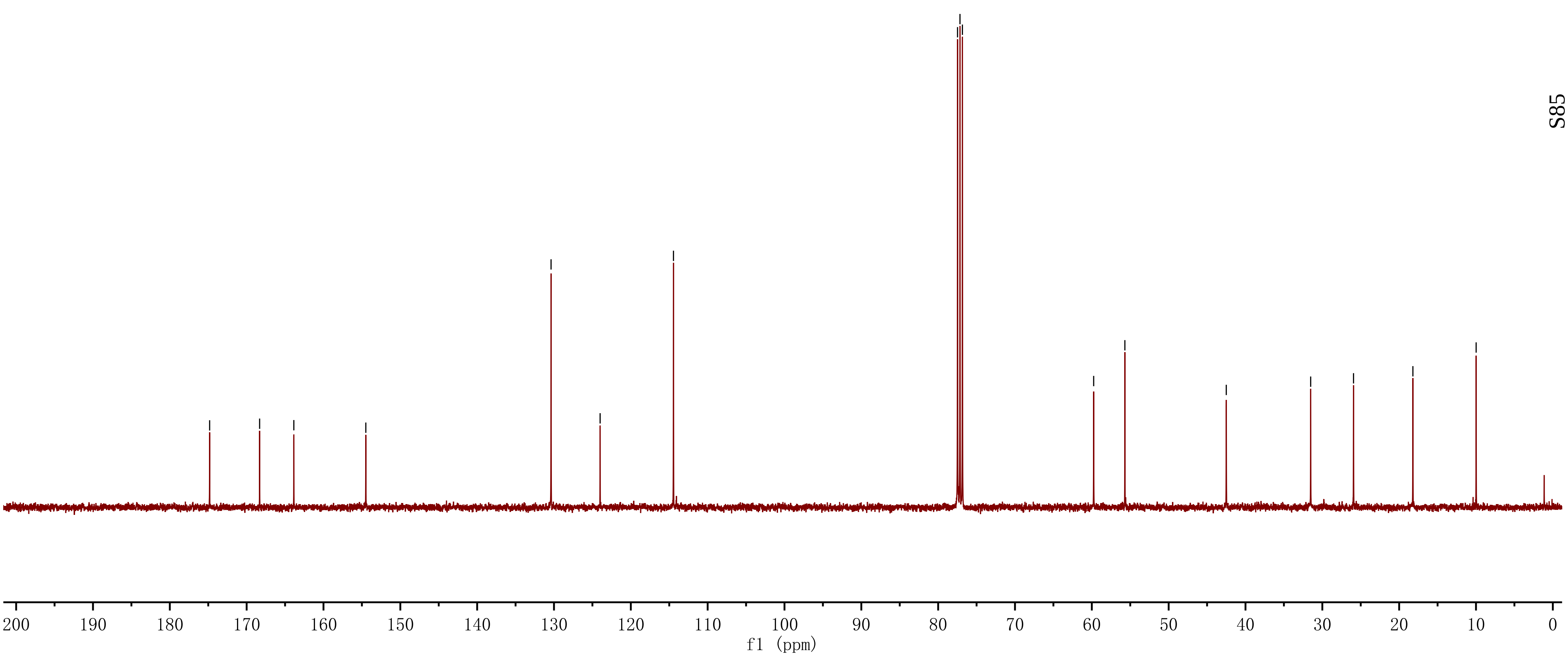

^{1}H NMR of P14
$CDCl_3$, 400 MHz

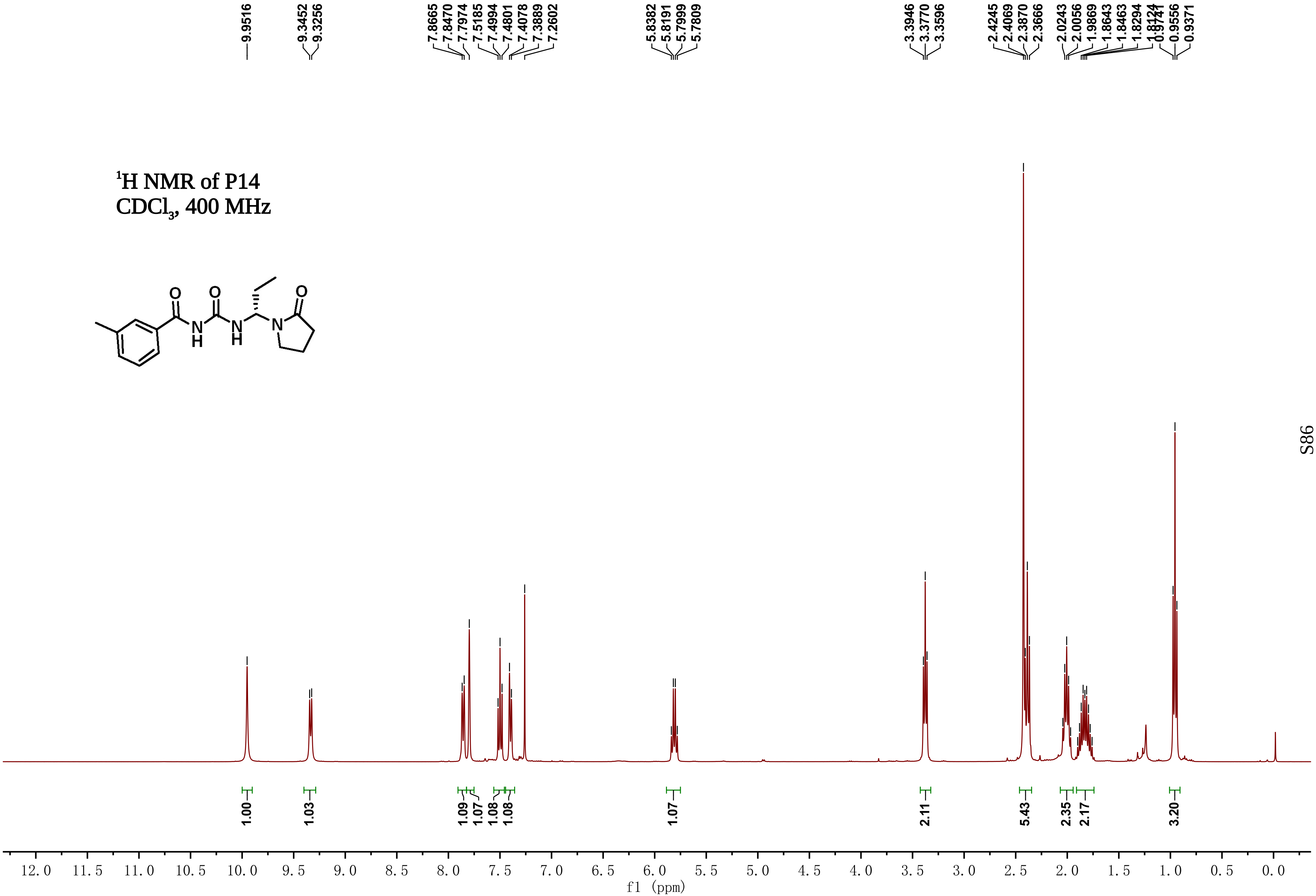

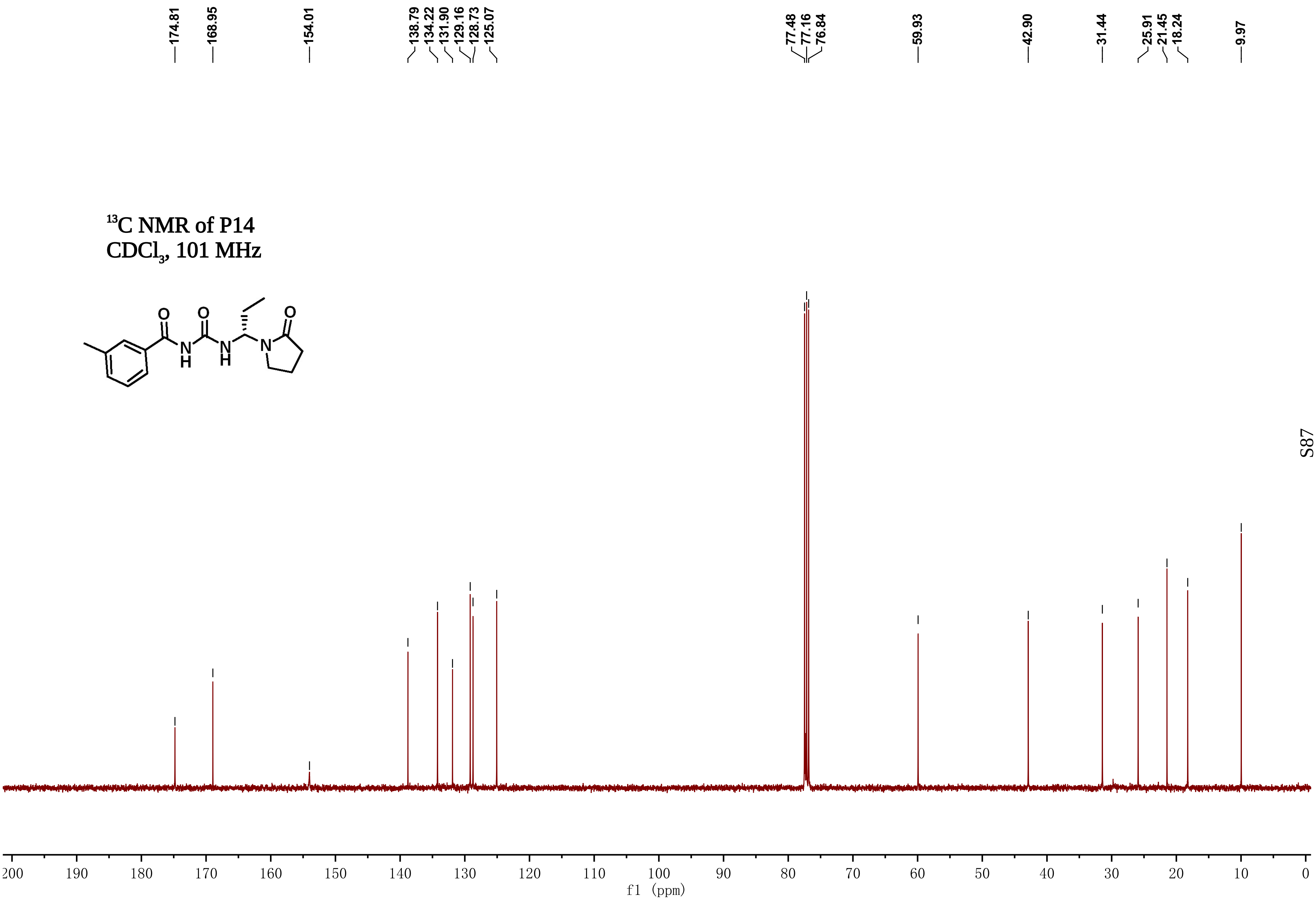

13C NMR of P14
CDCl3, 101 MHz
174.81
168.95
154.01
138.79
134.22
131.90
129.16
128.73
125.07
77.48
77.16
76.84
59.93
42.90
31.44
25.91
21.45
18.24
9.97
O
O
O
N
H
N
H
N
200 190 180 170 160 150 140 130 120 110 100 90 80 70 60 50 40 30 20 10 0
f1 (ppm)

^{1}H NMR of P15
$CDCl_3$, 400 MHz

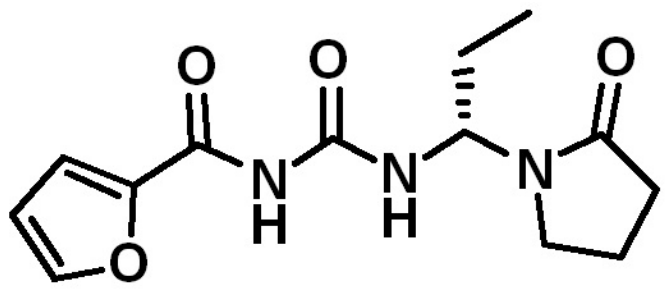

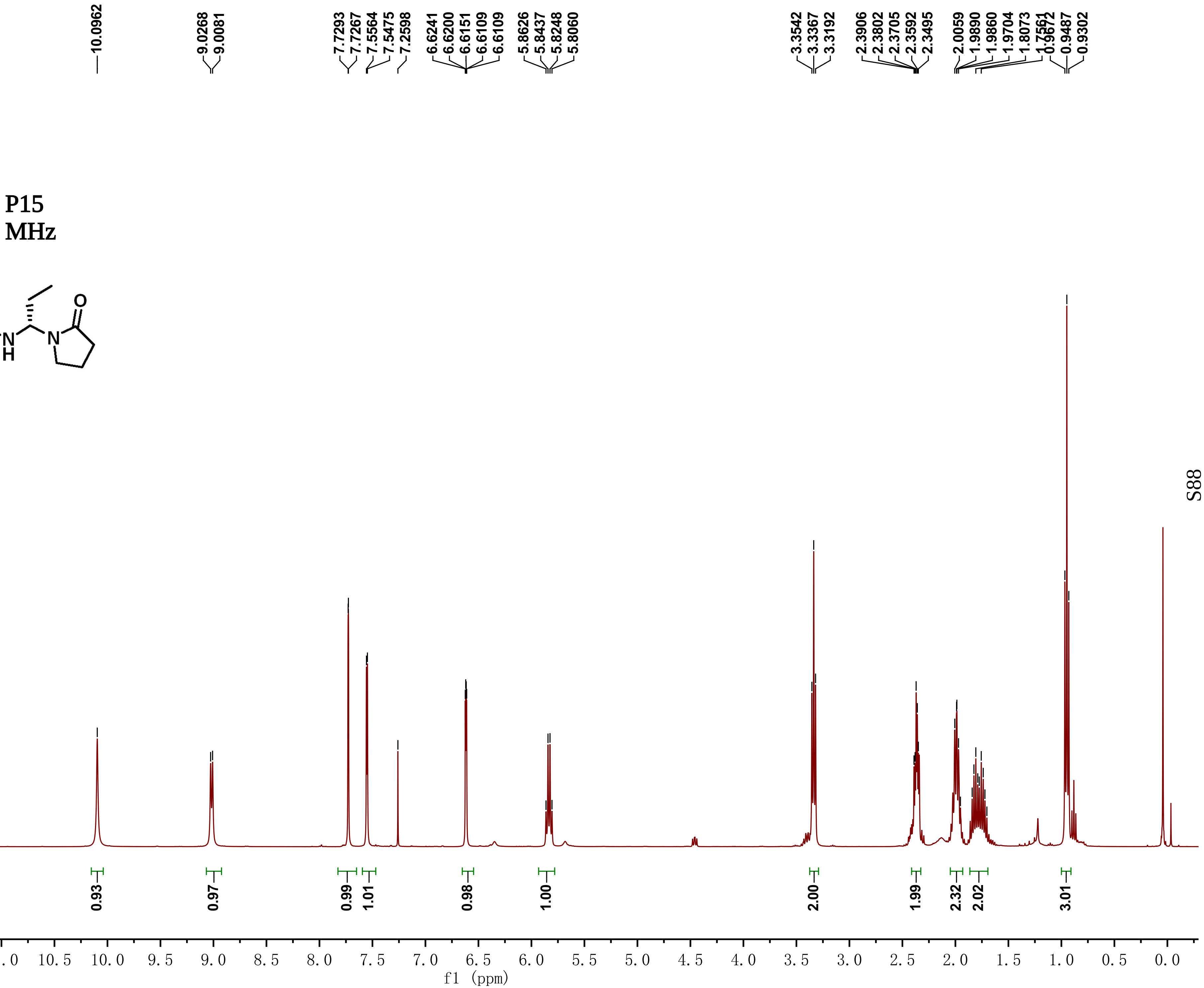

^{13}C NMR of P15
$CDCl_3$, 101 MHz

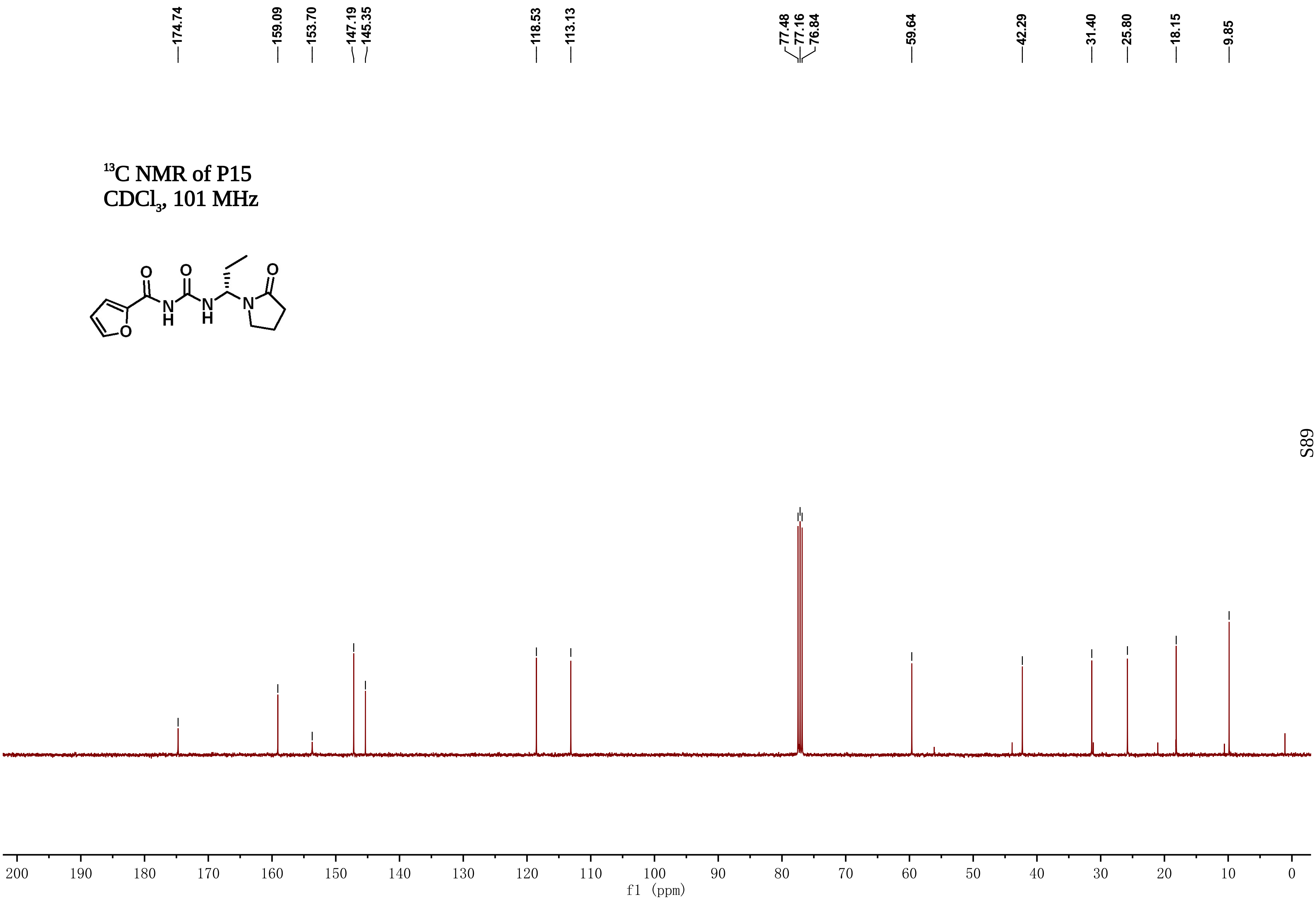

^{1}H NMR of P16
$CDCl_3$, 400 MHz

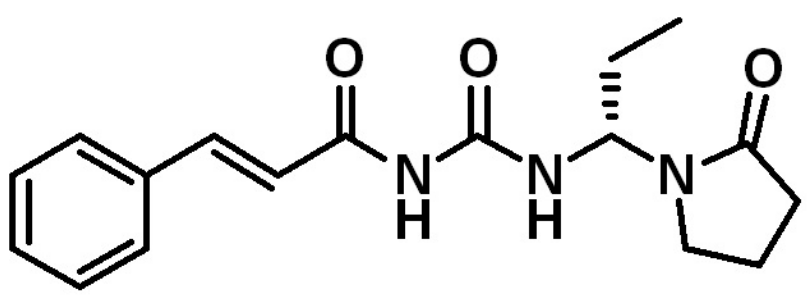

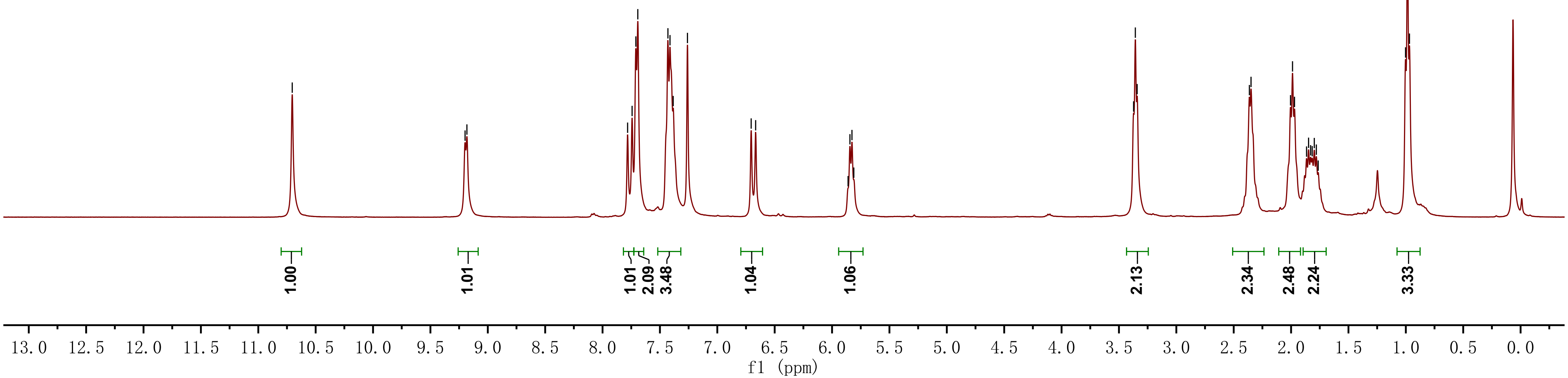

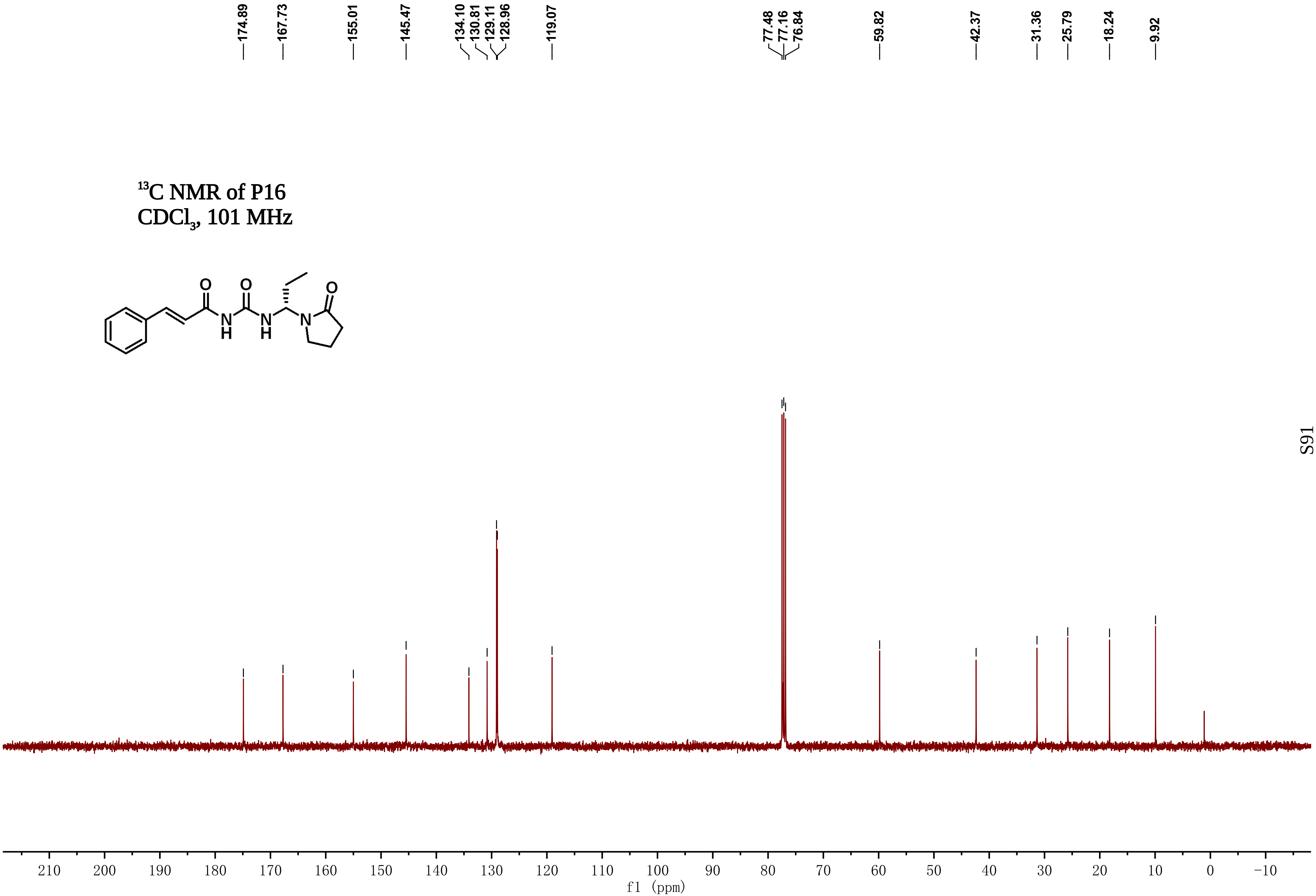

13C NMR of P16
CDCl3, 101 MHz
174.89
167.73
155.01
145.47
134.10
130.81
129.11
128.96
119.07
77.48
77.16
76.84
59.82
42.37
31.36
25.79
18.24
9.92
O
O
O
N
H
N
H
N
210 200 190 180 170 160 150 140 130 120 110 100 90 80 70 60 50 40 30 20 10 0 −10
f1 (ppm)

^{1}H NMR of P17
$CDCl_3$, 400 MHz

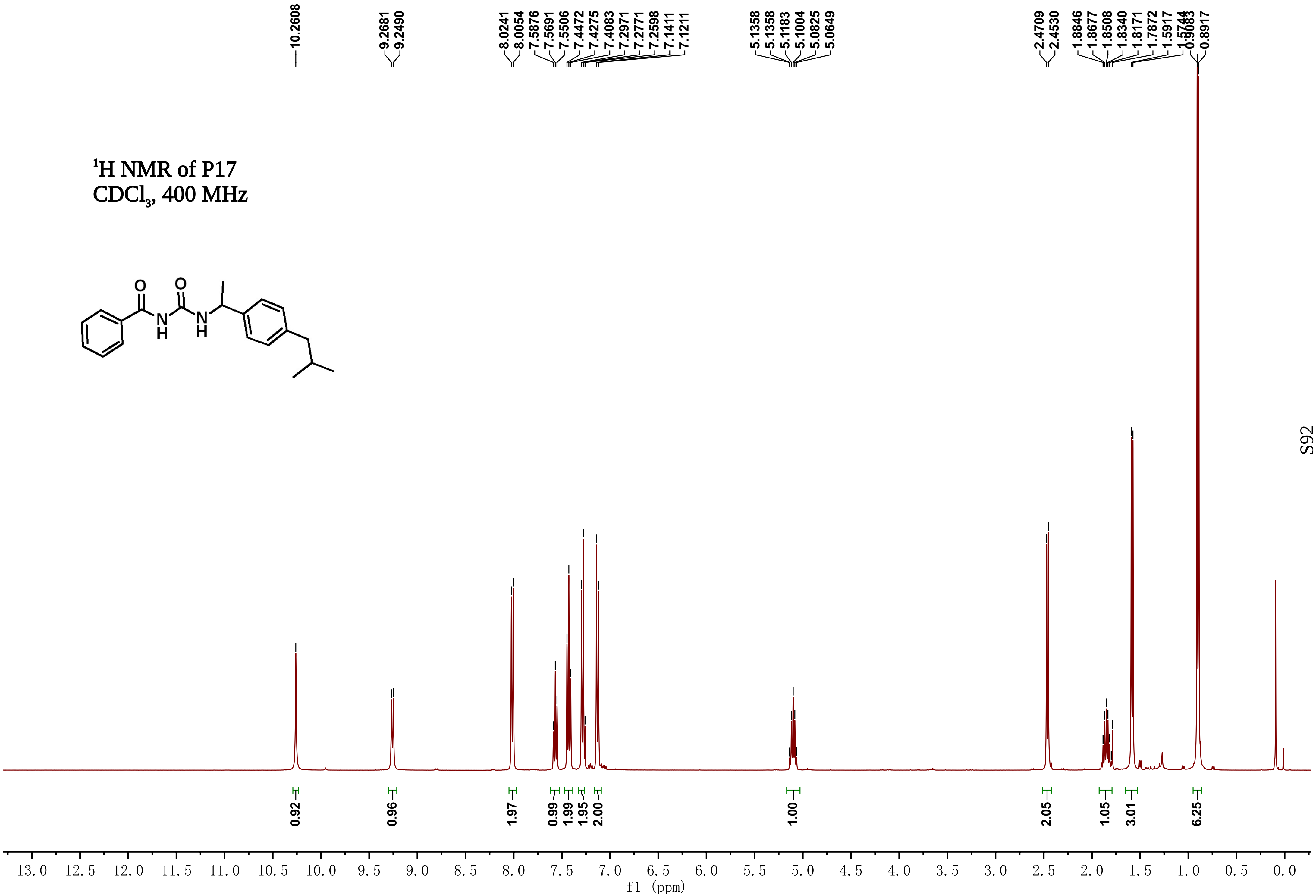

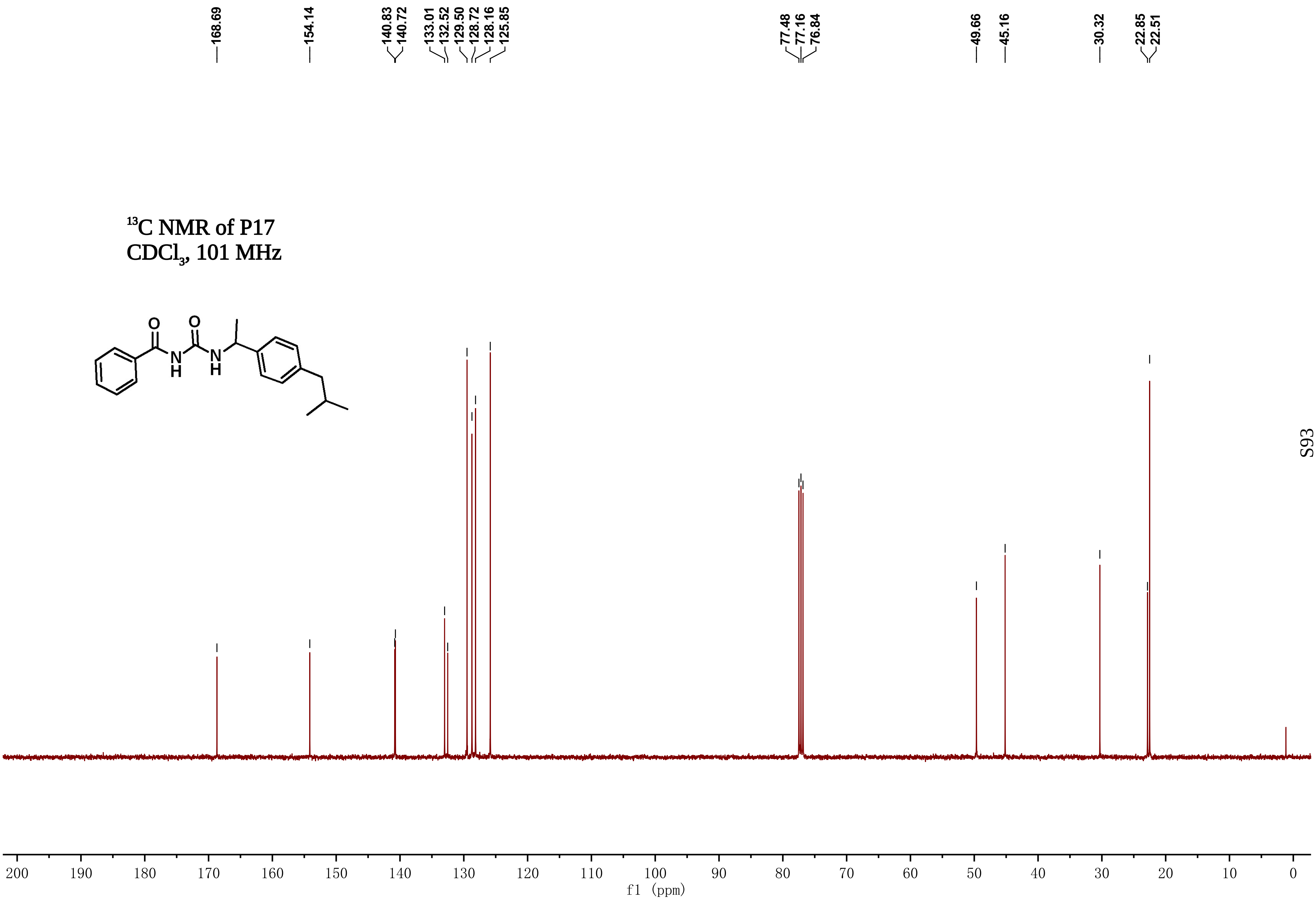

168.69
154.14
140.83
140.72
133.01
132.52
129.50
128.72
128.16
125.85
77.48
77.16
76.84
49.66
45.16
30.32
22.85
22.51
13C NMR of P17
CDCl3, 101 MHz
O
O
N
H
N
H
200 190 180 170 160 150 140 130 120 110 100 90 80 70 60 50 40 30 20 10 0
f1 (ppm)

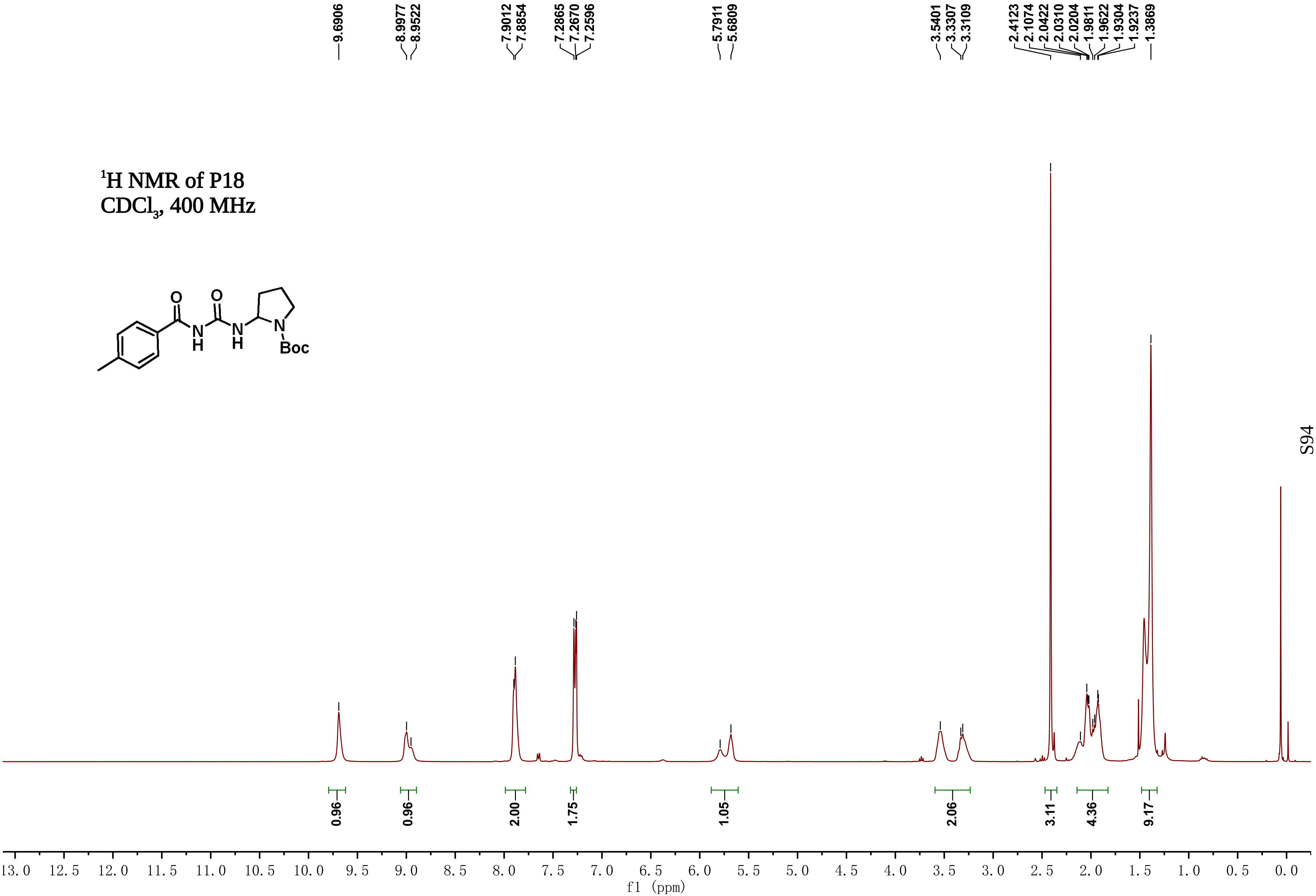

1H NMR of P18
CDCl3, 400 MHz
9.6906
8.9977
8.9522
7.9012
7.8854
7.2865
7.2670
7.2596
5.7911
5.6809
3.5401
3.3307
3.3109
2.4123
2.1074
2.0422
2.0310
2.0204
1.9811
1.9622
1.9304
1.9237
1.3869
0.96
0.96
2.00
1.75
1.05
2.06
3.11
4.36
9.17
O
O
N
H
N
H
N
Boc
f1 (ppm)

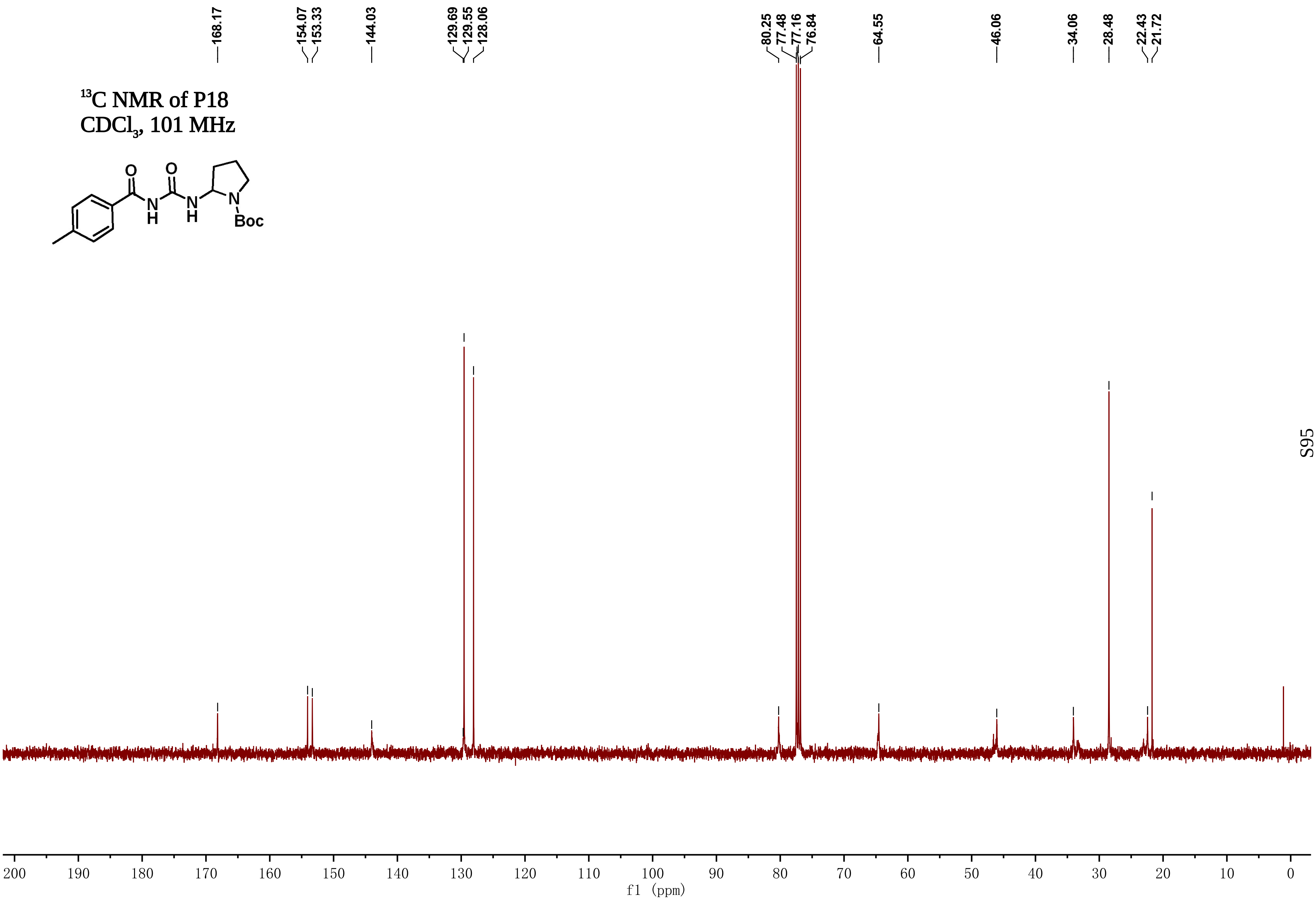

^{13}C NMR of P18
$CDCl_3$, 101 MHz
O
O
N
H
N
H
N
Boc
168.17
154.07
153.33
144.03
129.69
129.55
128.06
80.25
77.48
77.16
76.84
64.55
46.06
34.06
28.48
22.43
21.72
200 190 180 170 160 150 140 130 120 110 100 90 80 70 60 50 40 30 20 10 0
f1 (ppm)

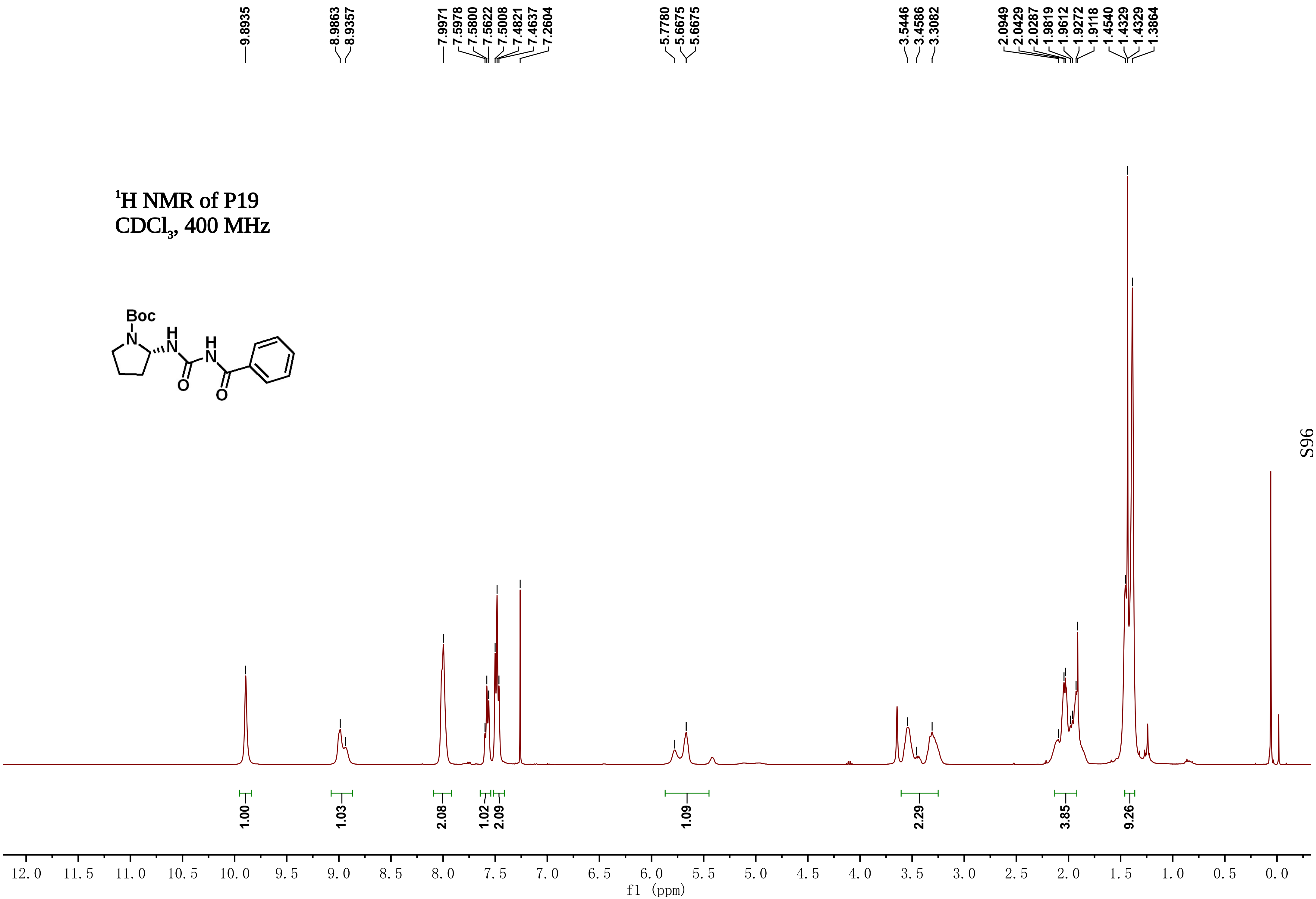

9.8935
8.9863
8.9357
7.9971
7.5978
7.5800
7.5622
7.5008
7.4821
7.4637
7.2604
5.7780
5.6675
5.6675
3.5446
3.4586
3.3082
2.0949
2.0429
2.0287
1.9819
1.9612
1.9272
1.9118
1.4540
1.4329
1.4329
1.3864
1H NMR of P19
CDCl3, 400 MHz
Boc
N
H
N
H
N
O
O
1.00
1.03
2.08
1.02
2.09
1.09
2.29
3.85
9.26
12.0 11.5 11.0 10.5 10.0 9.5 9.0 8.5 8.0 7.5 7.0 6.5 6.0 5.5 5.0 4.5 4.0 3.5 3.0 2.5 2.0 1.5 1.0 0.5 0.0
f1 (ppm)

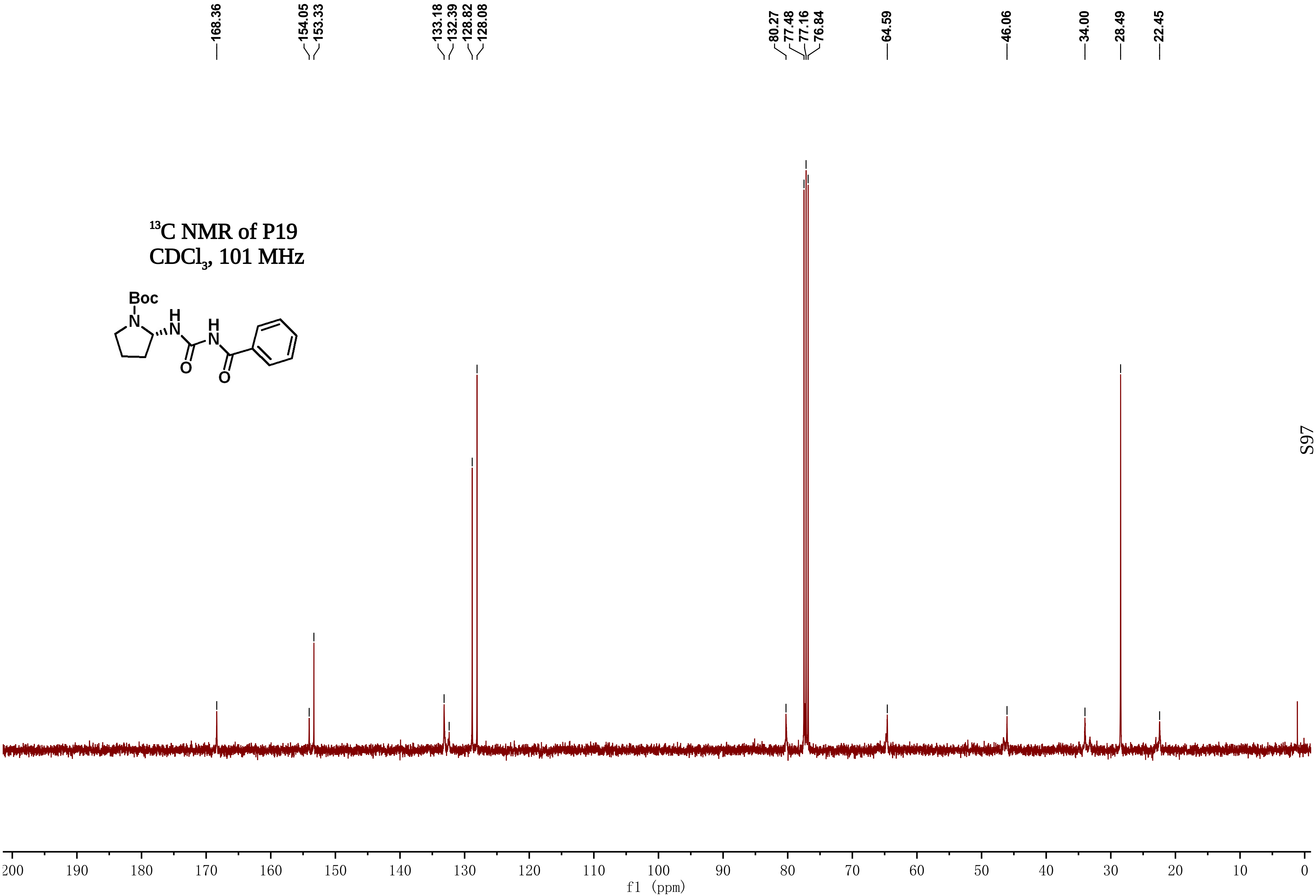
168.36
154.05
153.33
133.18
132.39
128.82
128.08
80.27
77.48
77.16
76.84
64.59
46.06
34.00
28.49
22.45
^{13}C NMR of P19
$CDCl_3$, 101 MHz
Boc
N
H
N
H
N
O
O
200 190 180 170 160 150 140 130 120 110 100 90 80 70 60 50 40 30 20 10 0
f1 (ppm)

^{1}H NMR of P20
$CDCl_3$, 400 MHz

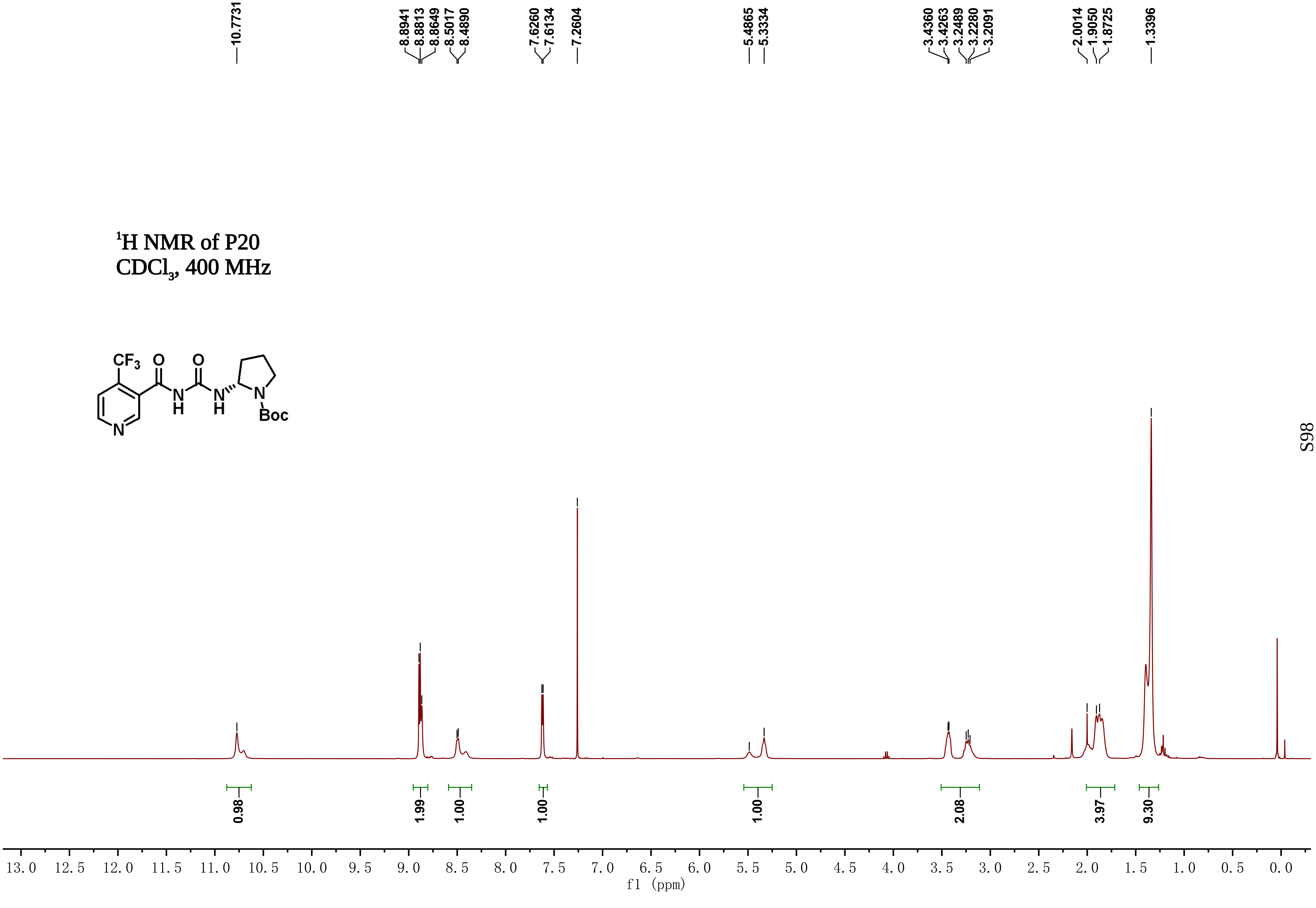

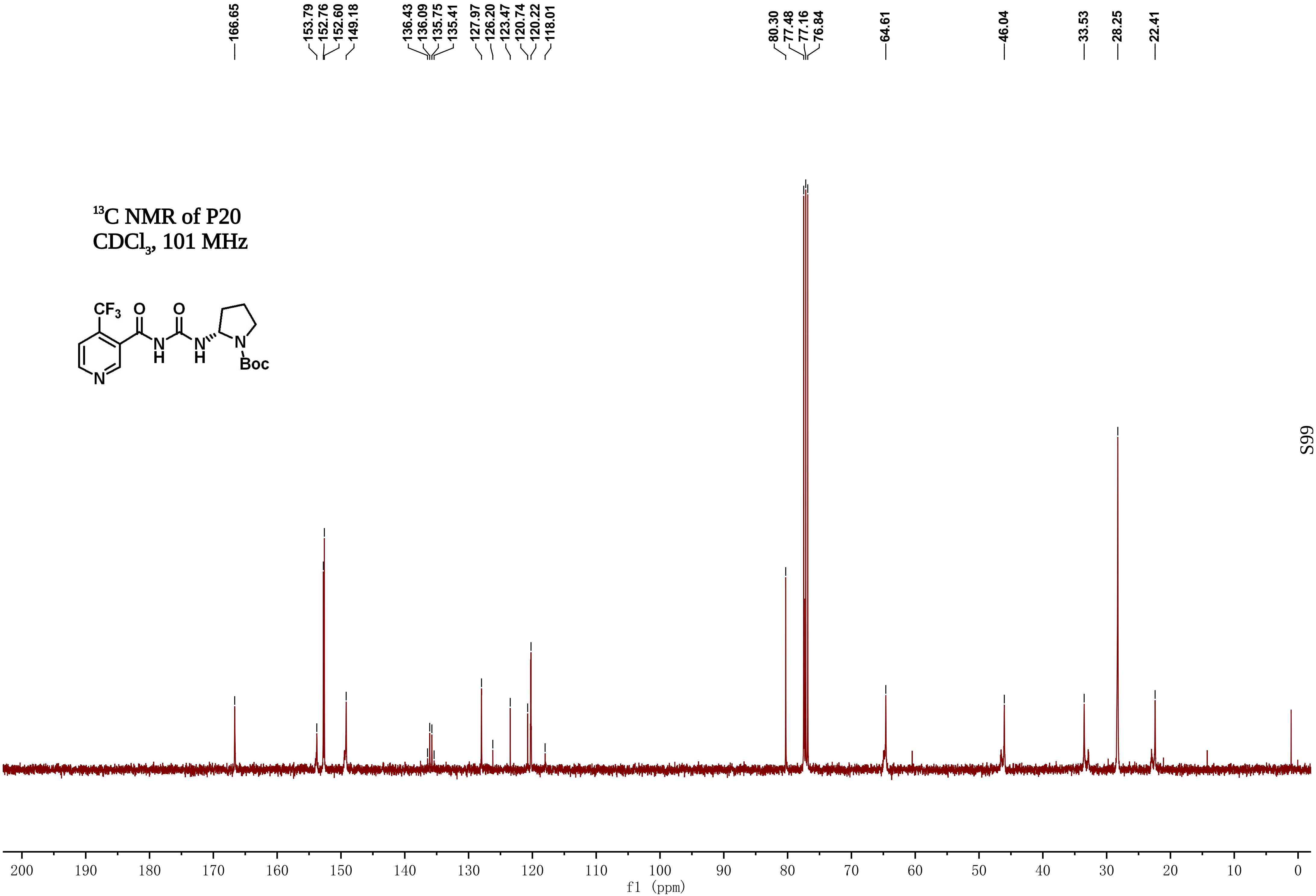

166.65
153.79
152.76
152.60
149.18
136.43
136.09
135.75
135.41
127.97
126.20
123.47
120.74
120.22
118.01
80.30
77.48
77.16
76.84
64.61
46.04
33.53
28.25
22.41
13C NMR of P20
CDCl3, 101 MHz
CF3
O
O
N
H
N
H
N
Boc
N
f1 (ppm)

^{19}F NMR of P20
$CDCl_3$, 376 MHz

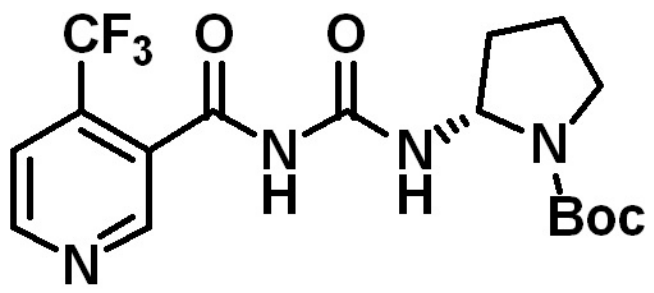

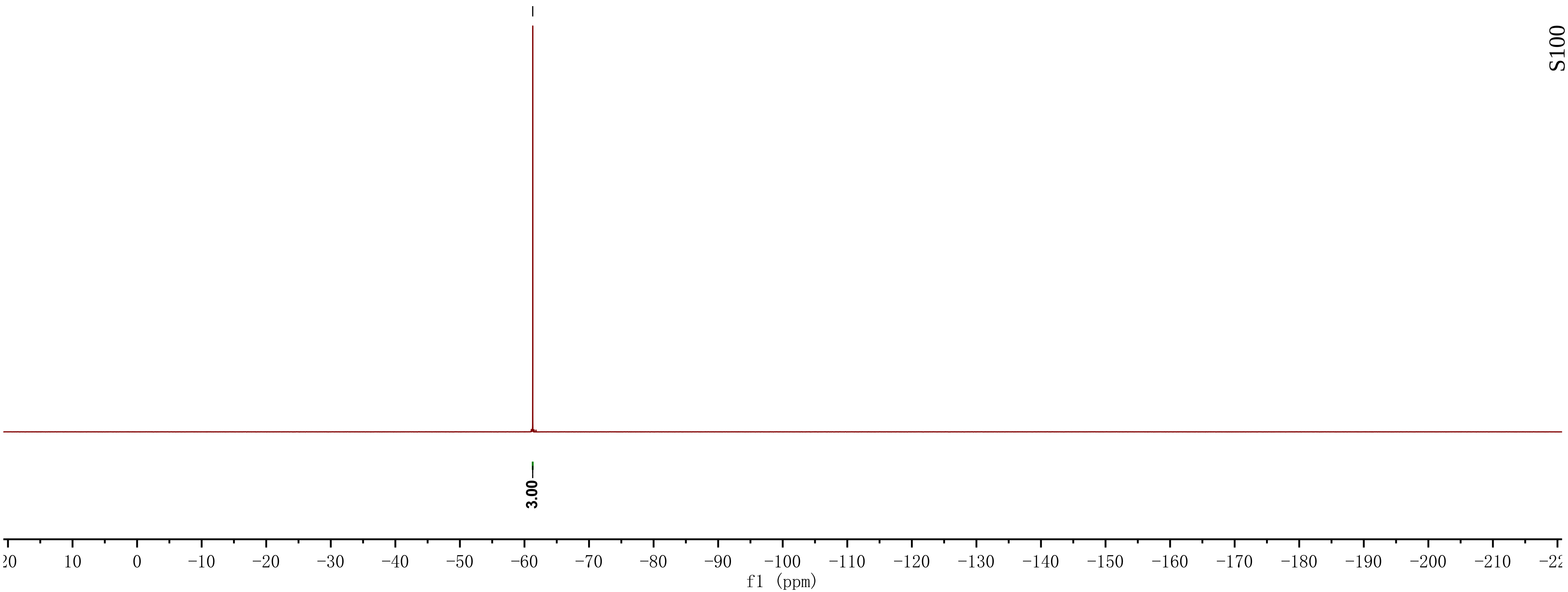

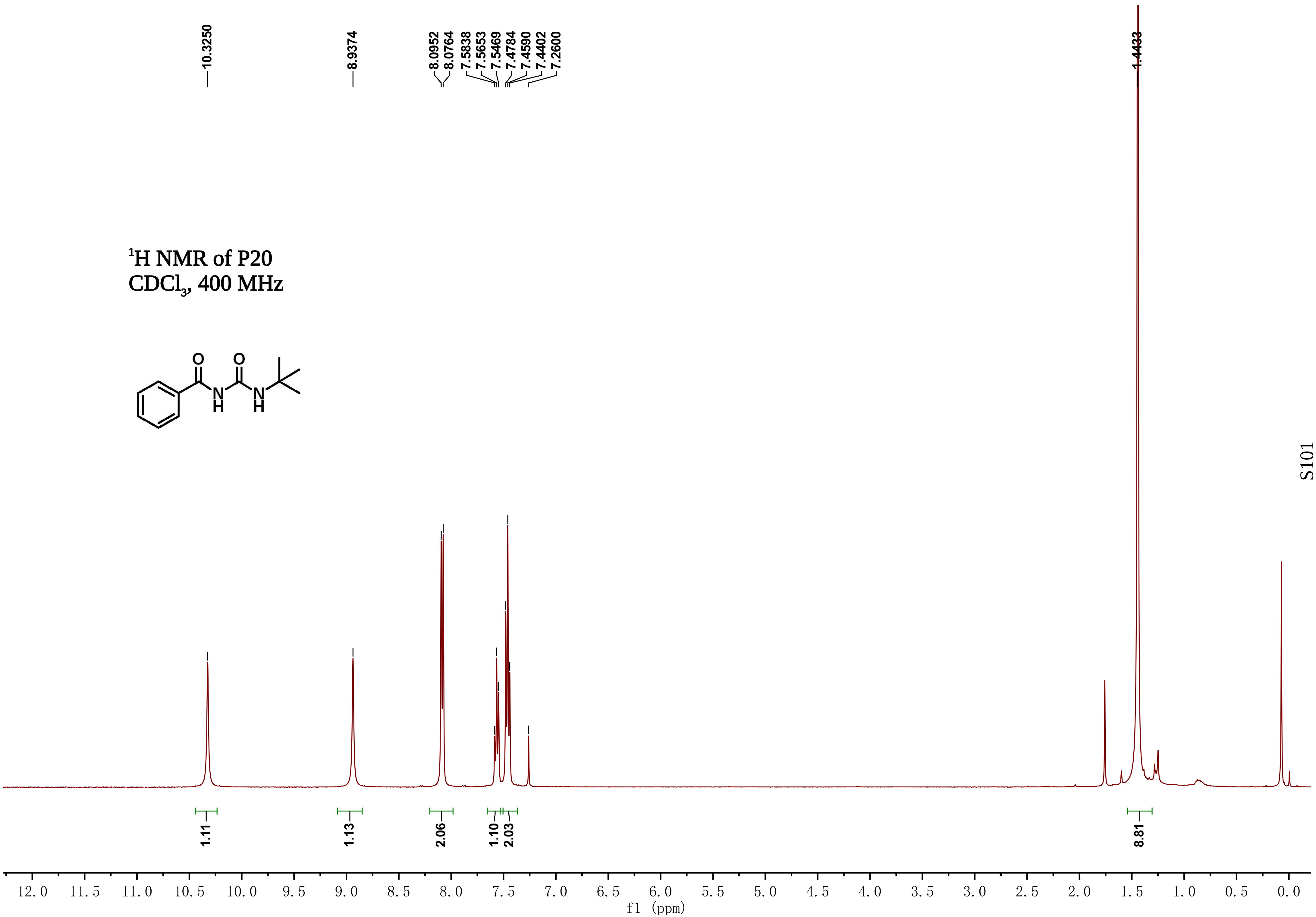

1H NMR of P20
CDCl3, 400 MHz
10.3250
8.9374
8.0952
8.0764
7.5838
7.5653
7.5469
7.4784
7.4590
7.4402
7.2600
1.4433
1.11
1.13
2.06
1.10
2.03
8.81
12.0 11.5 11.0 10.5 10.0 9.5 9.0 8.5 8.0 7.5 7.0 6.5 6.0 5.5 5.0 4.5 4.0 3.5 3.0 2.5 2.0 1.5 1.0 0.5 0.0
f1 (ppm)

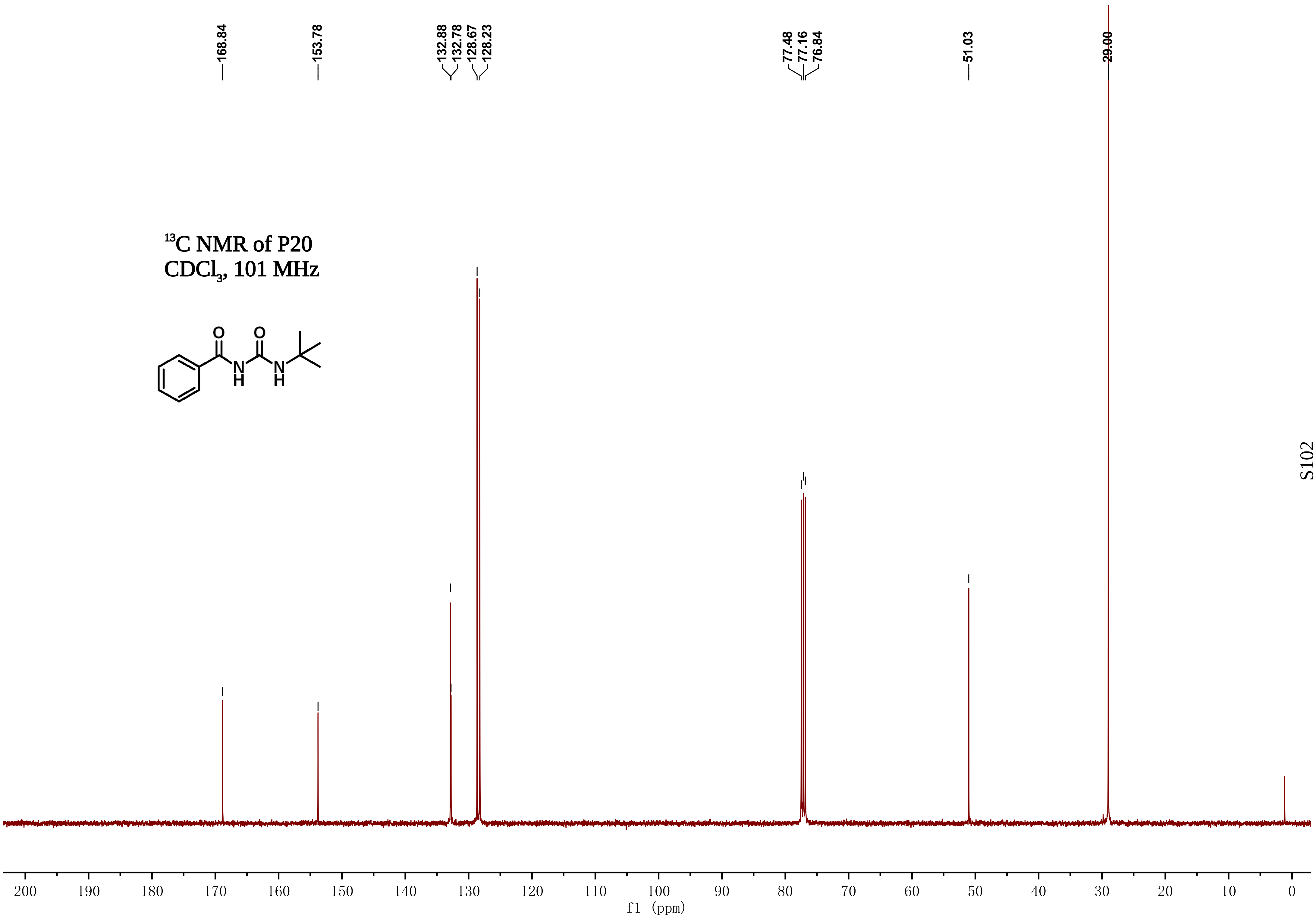
168.84
153.78
132.88
132.78
128.67
128.23
77.48
77.16
76.84
51.03
29.00
13C NMR of P20
CDCl3, 101 MHz
O
O
N
H
N
H
200 190 180 170 160 150 140 130 120 110 100 90 80 70 60 50 40 30 20 10 0
f1 (ppm)

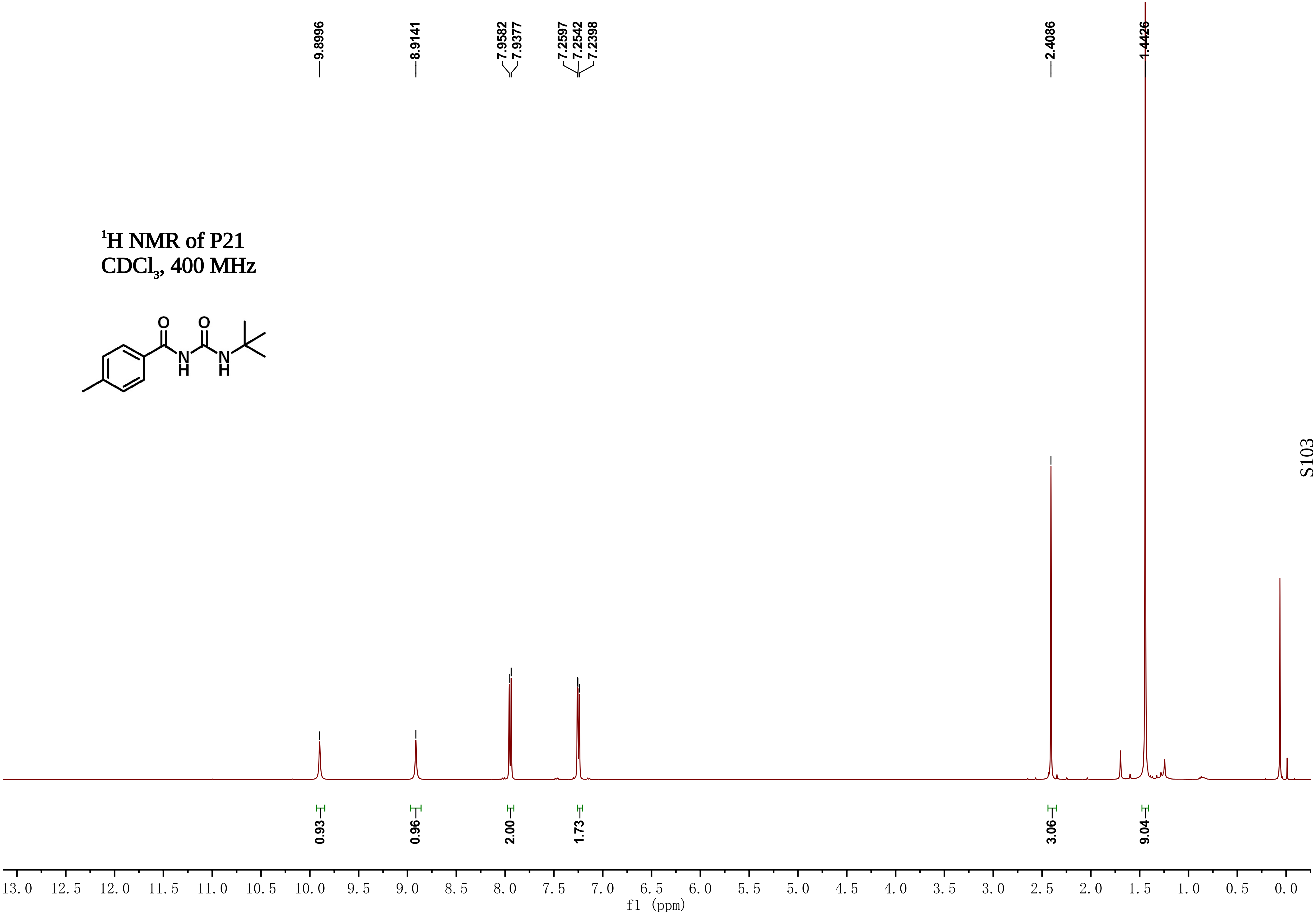

^{1}H NMR of P21
CDCl3, 400 MHz
9.8996
8.9141
7.9582
7.9377
7.2597
7.2542
7.2398
2.4086
1.4426
0.93
0.96
2.00
1.73
3.06
9.04
13.0 12.5 12.0 11.5 11.0 10.5 10.0 9.5 9.0 8.5 8.0 7.5 7.0 6.5 6.0 5.5 5.0 4.5 4.0 3.5 3.0 2.5 2.0 1.5 1.0 0.5 0.0
f1 (ppm)

^{13}C NMR of P21
$CDCl_3$, 101 MHz

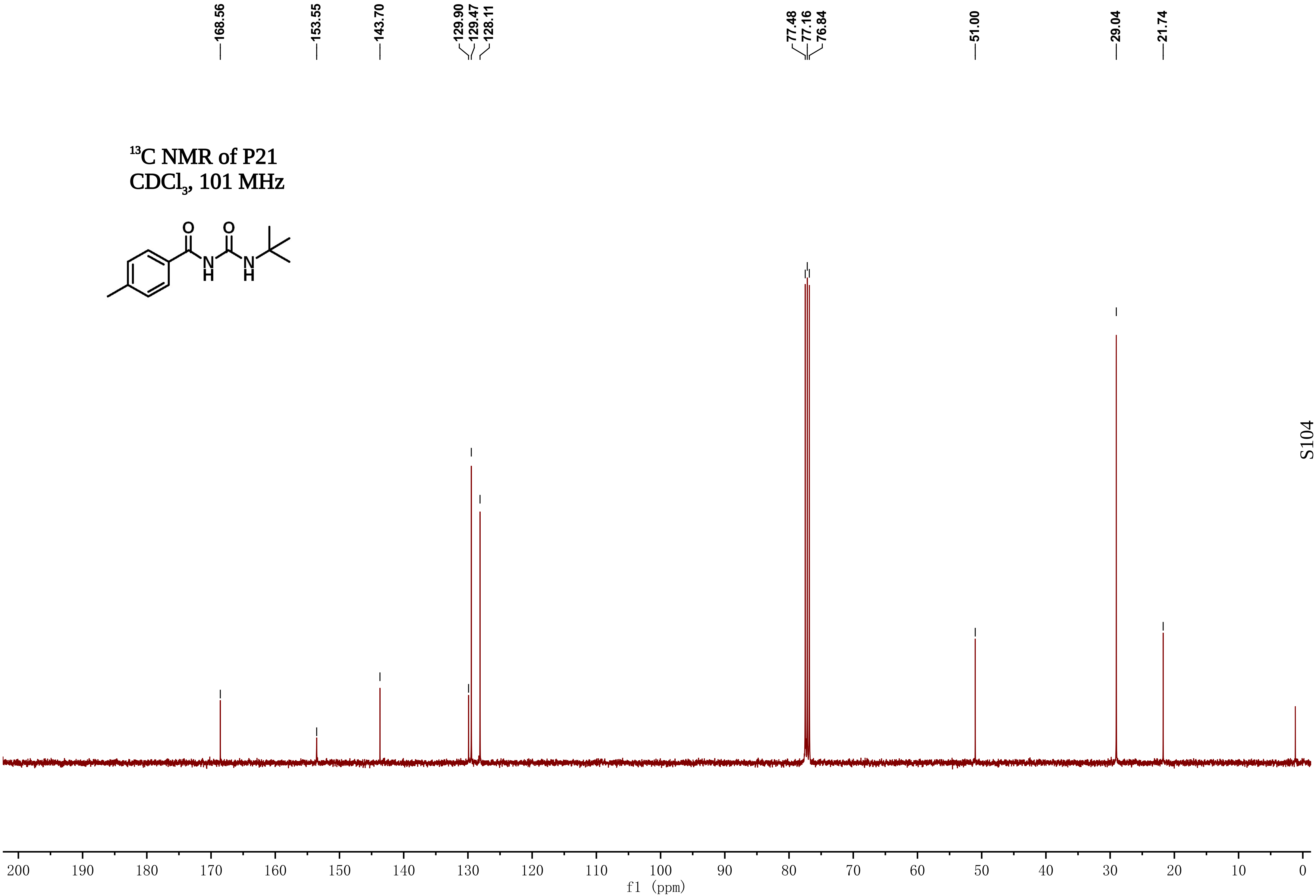

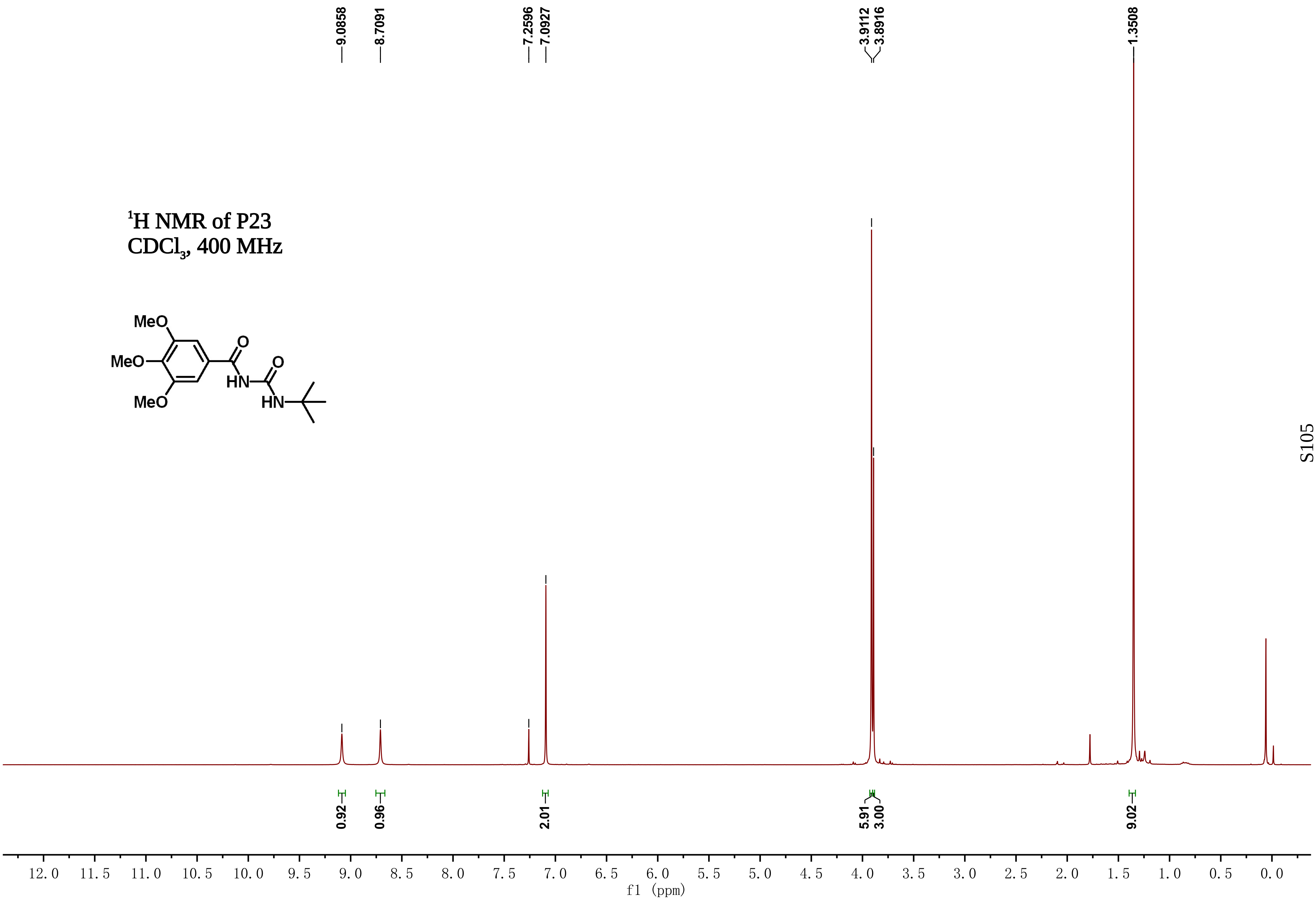

9.0858
8.7091
7.2596
7.0927
3.9112
3.8916
1.3508
^{1}H NMR of P23
$CDCl_3$, 400 MHz
MeO
MeO
MeO
O
O
HN
HN
0.92
0.96
2.01
5.91
3.00
9.02
12.0 11.5 11.0 10.5 10.0 9.5 9.0 8.5 8.0 7.5 7.0 6.5 6.0 5.5 5.0 4.5 4.0 3.5 3.0 2.5 2.0 1.5 1.0 0.5 0.0
f1 (ppm)

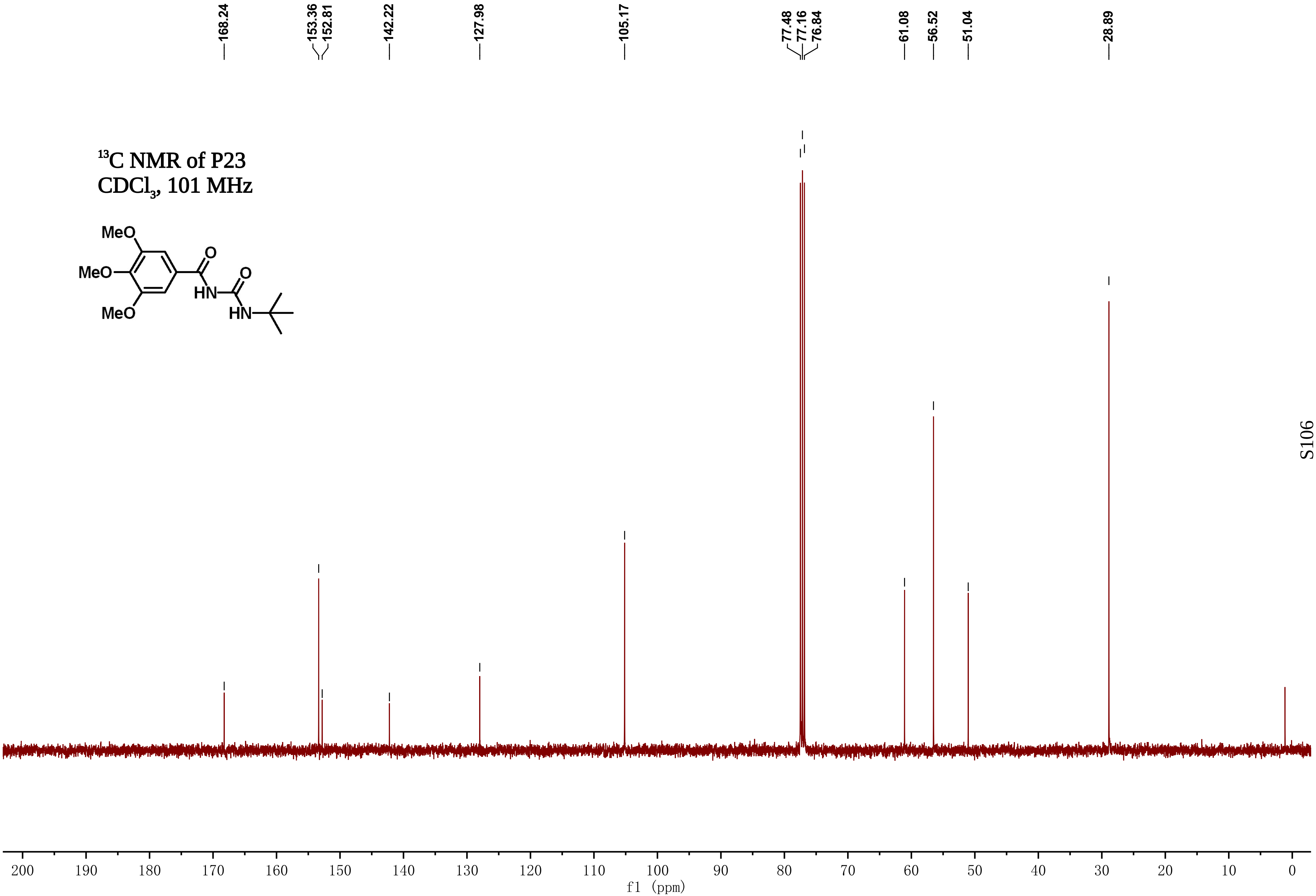

168.24
153.36
152.81
142.22
127.98
105.17
77.48
77.16
76.84
61.08
56.52
51.04
28.89
^{13}C NMR of P23
$CDCl_3$, 101 MHz
MeO
MeO
MeO
O
O
HN
HN
200 190 180 170 160 150 140 130 120 110 100 90 80 70 60 50 40 30 20 10 0
f1 (ppm)

^{1}H NMR of P24
$CDCl_3$, 400 MHz

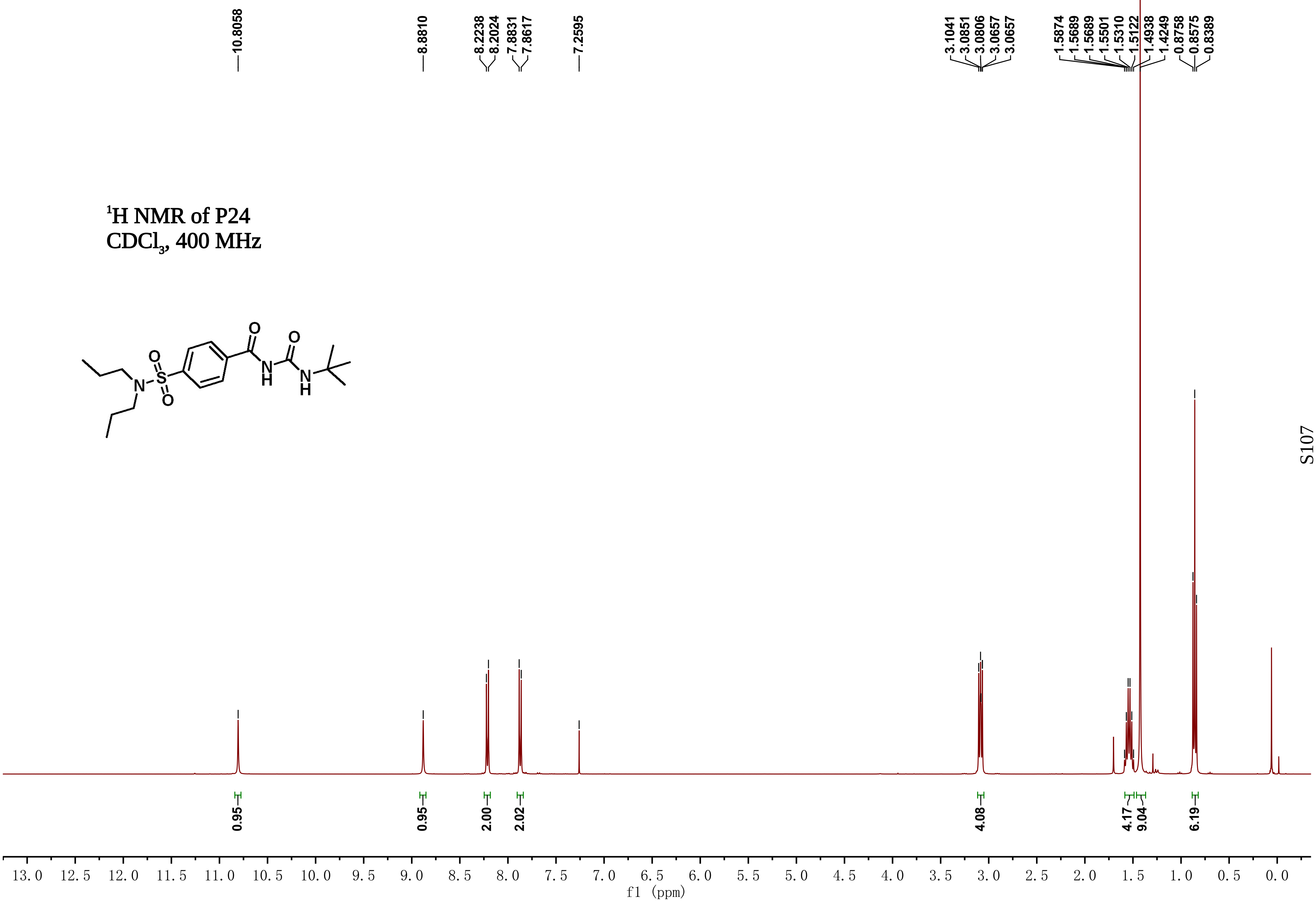

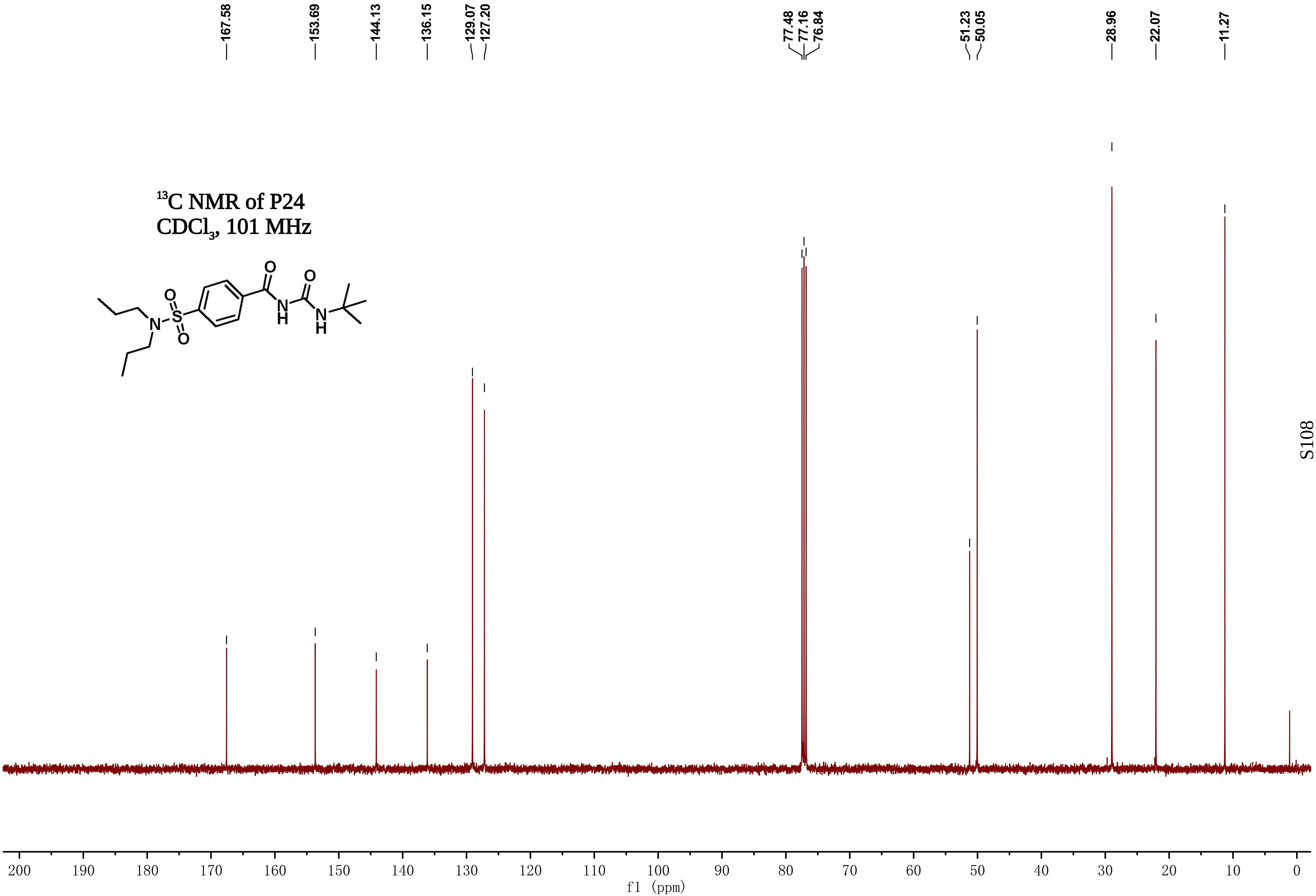

167.58
153.69
144.13
136.15
129.07
127.20
77.48
77.16
76.84
51.23
50.05
28.96
22.07
11.27
^{13}C NMR of P24
$CDCl_3$, 101 MHz
O
O
N
S
O
O
N
H
N
H
200 190 180 170 160 150 140 130 120 110 100 90 80 70 60 50 40 30 20 10 0
f1 (ppm)

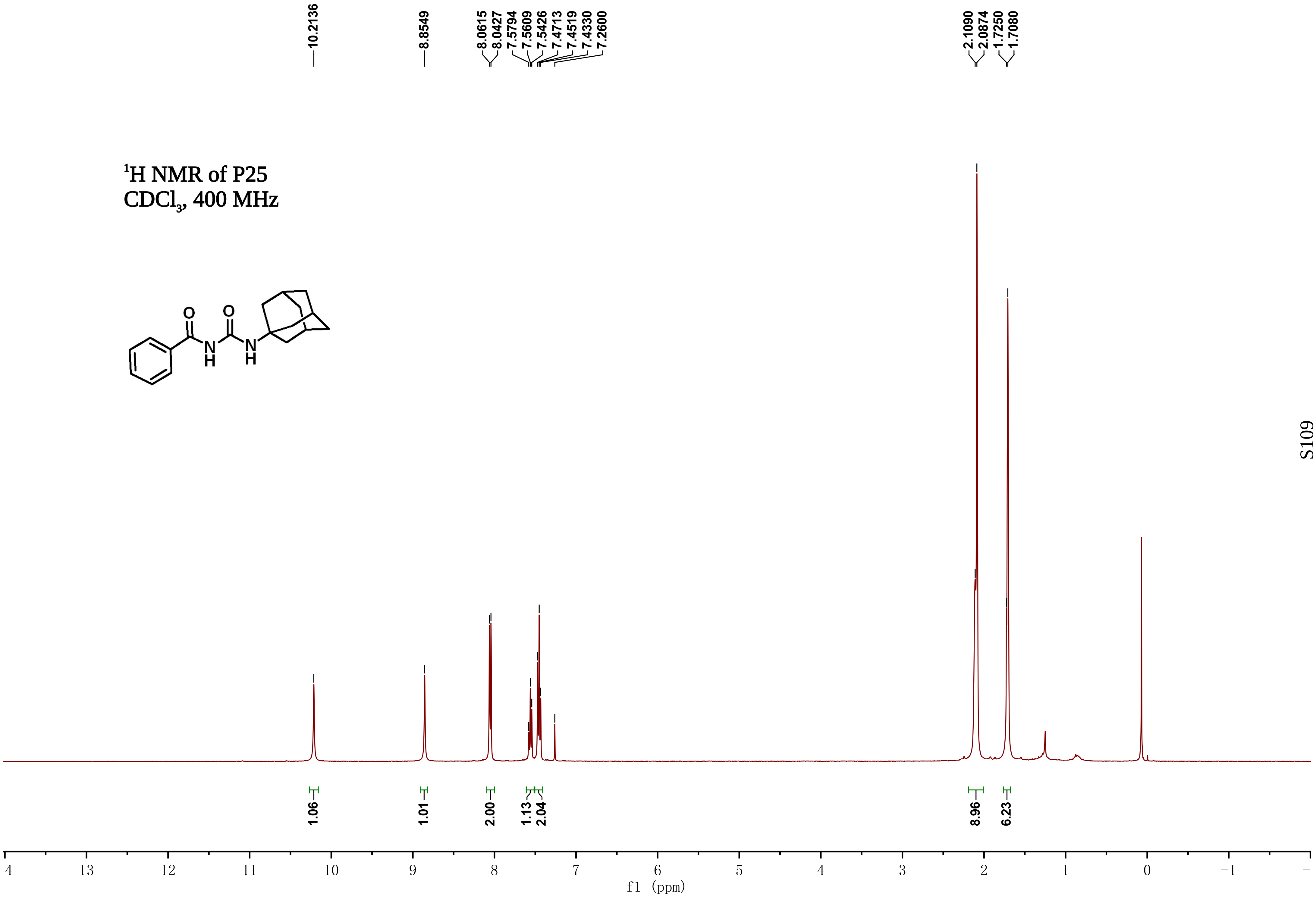

1H NMR of P25
CDCl3, 400 MHz
10.2136
8.8549
8.0615
8.0427
7.5794
7.5609
7.5426
7.4713
7.4519
7.4330
7.2600
2.1090
2.0874
1.7250
1.7080
1.06
1.01
2.00
1.13
2.04
8.96
6.23
f1 (ppm)

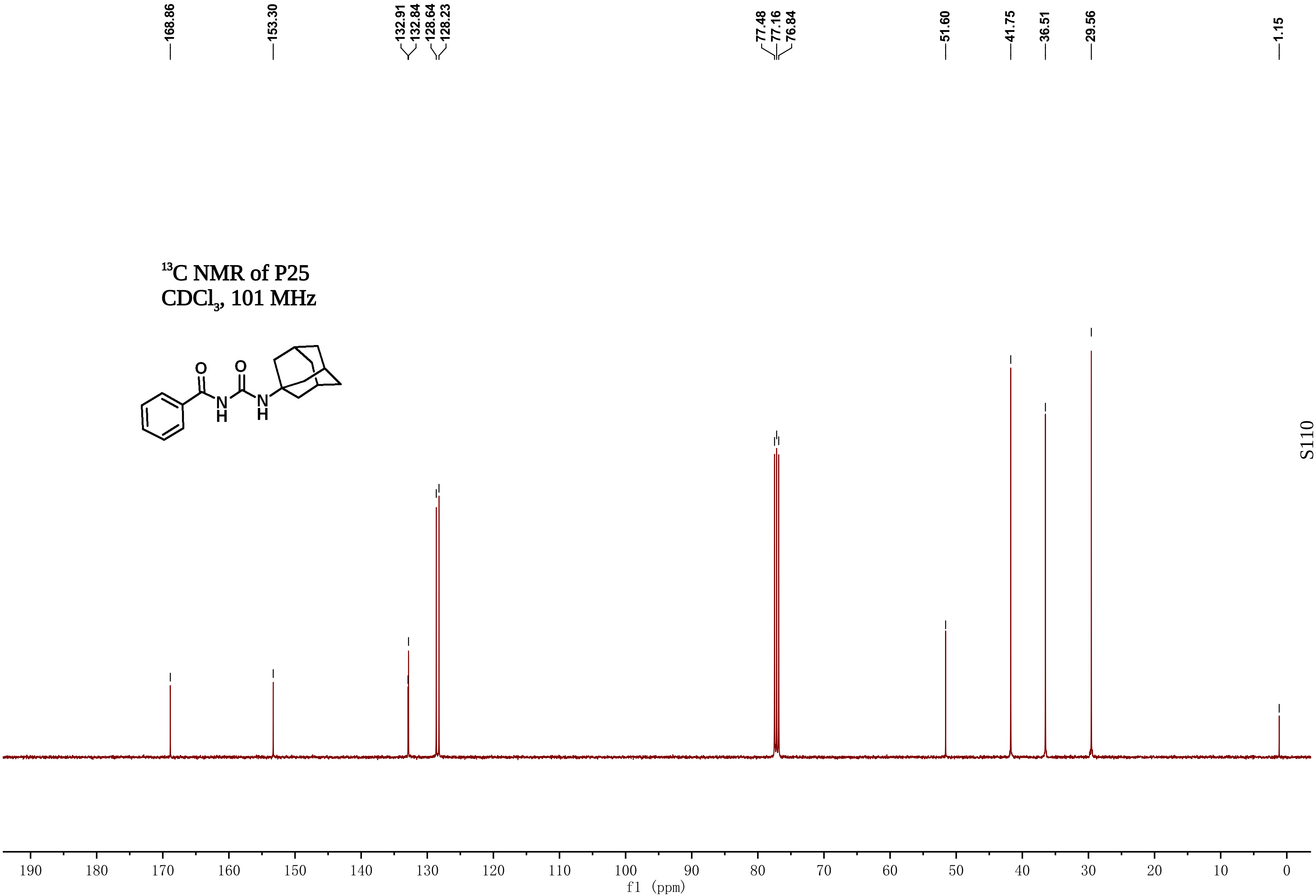
168.86
153.30
132.91
132.84
128.64
128.23
77.48
77.16
76.84
51.60
41.75
36.51
29.56
1.15
13C NMR of P25
CDCl3, 101 MHz
O
O
N
H
N
H
190 180 170 160 150 140 130 120 110 100 90 80 70 60 50 40 30 20 10 0
f1 (ppm)

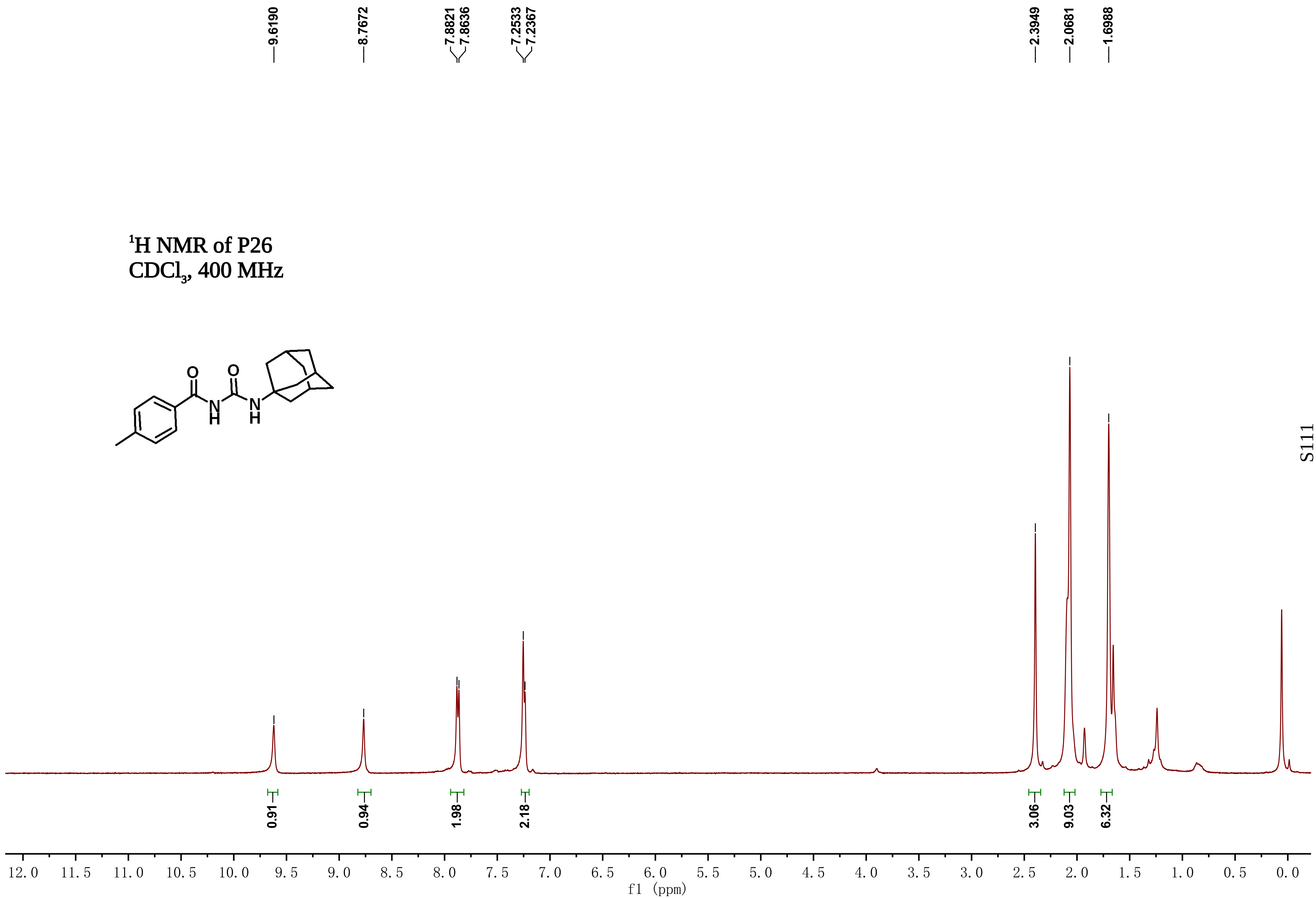

9.6190
8.7672
7.8821
7.8636
7.2533
7.2367
2.3949
2.0681
1.6988
^{1}H NMR of P26
CDCl$_3$, 400 MHz
0.91
0.94
1.98
2.18
3.06
9.03
6.32
12.0 11.5 11.0 10.5 10.0 9.5 9.0 8.5 8.0 7.5 7.0 6.5 6.0 5.5 5.0 4.5 4.0 3.5 3.0 2.5 2.0 1.5 1.0 0.5 0.0
f1 (ppm)

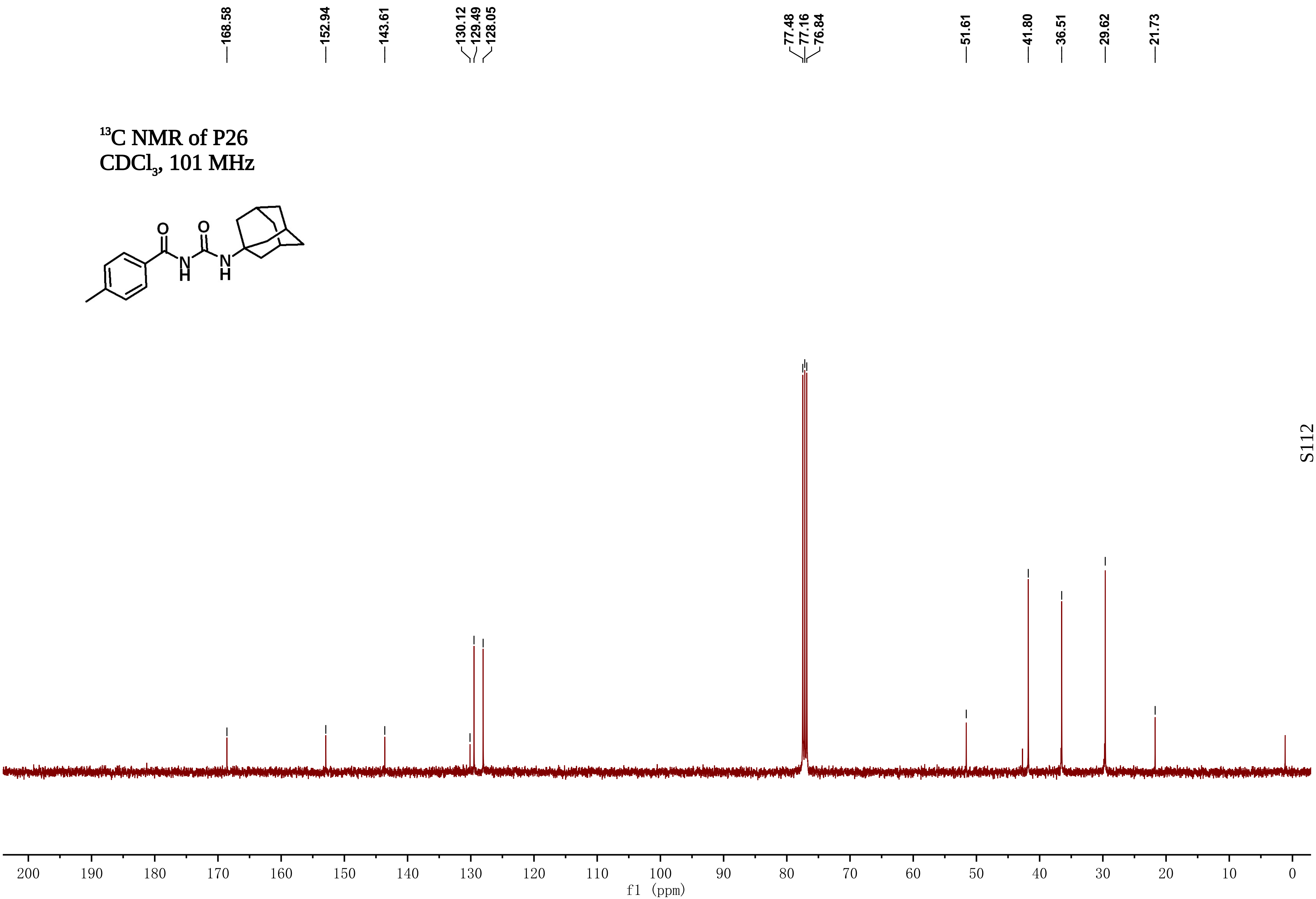

^{13}C NMR of P26
$CDCl_3$, 101 MHz
168.58
152.94
143.61
130.12
129.49
128.05
77.48
77.16
76.84
51.61
41.80
36.51
29.62
21.73
O
O
N
H
N
H
200
190
180
170
160
150
140
130
120
110
100
90
80
70
60
50
40
30
20
10
0
f1 (ppm)

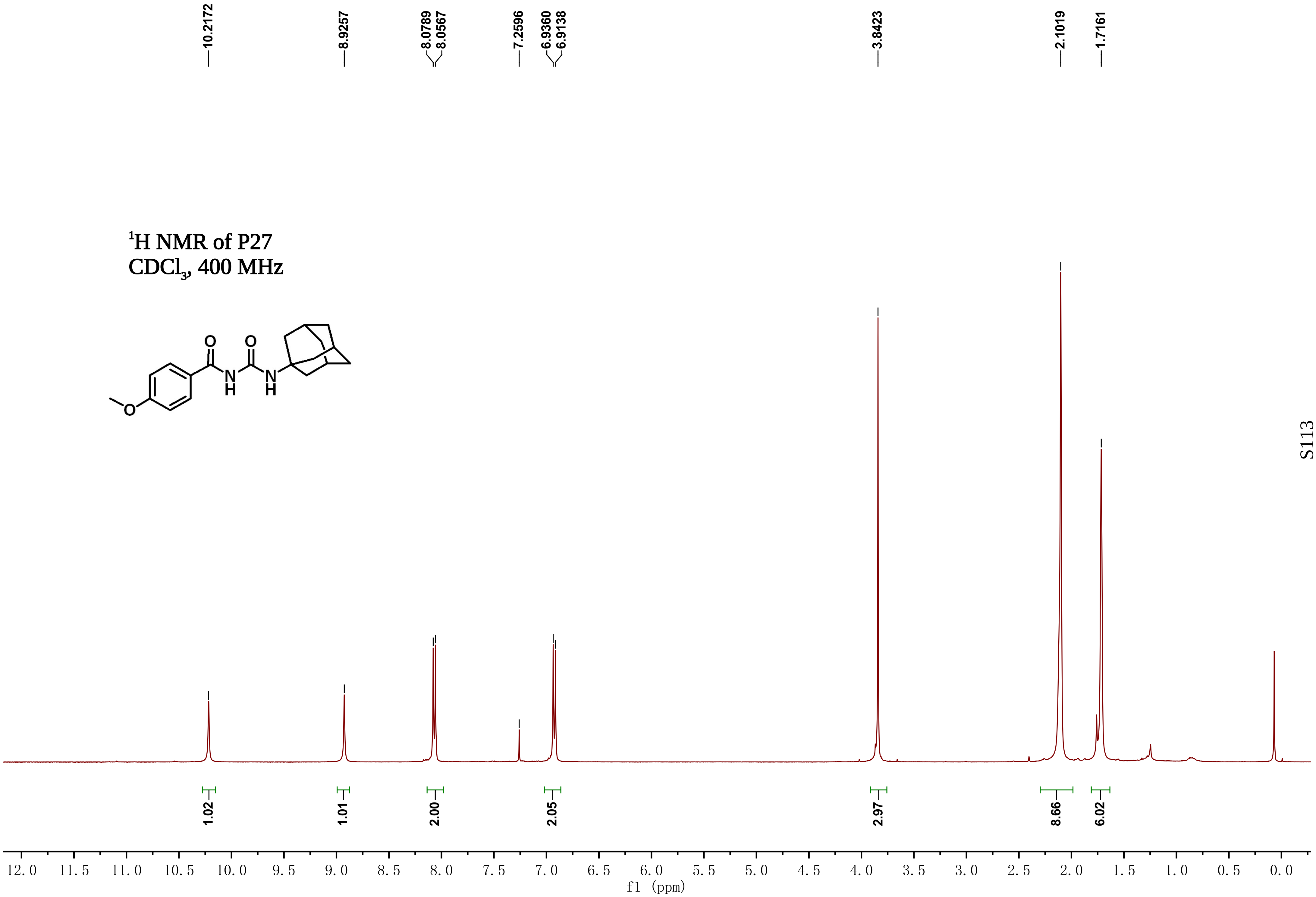

1H NMR of P27
CDCl3, 400 MHz
10.2172
8.9257
8.0789
8.0567
7.2596
6.9360
6.9138
3.8423
2.1019
1.7161
1.02
1.01
2.00
2.05
2.97
8.66
6.02
12.0 11.5 11.0 10.5 10.0 9.5 9.0 8.5 8.0 7.5 7.0 6.5 6.0 5.5 5.0 4.5 4.0 3.5 3.0 2.5 2.0 1.5 1.0 0.5 0.0
f1 (ppm)

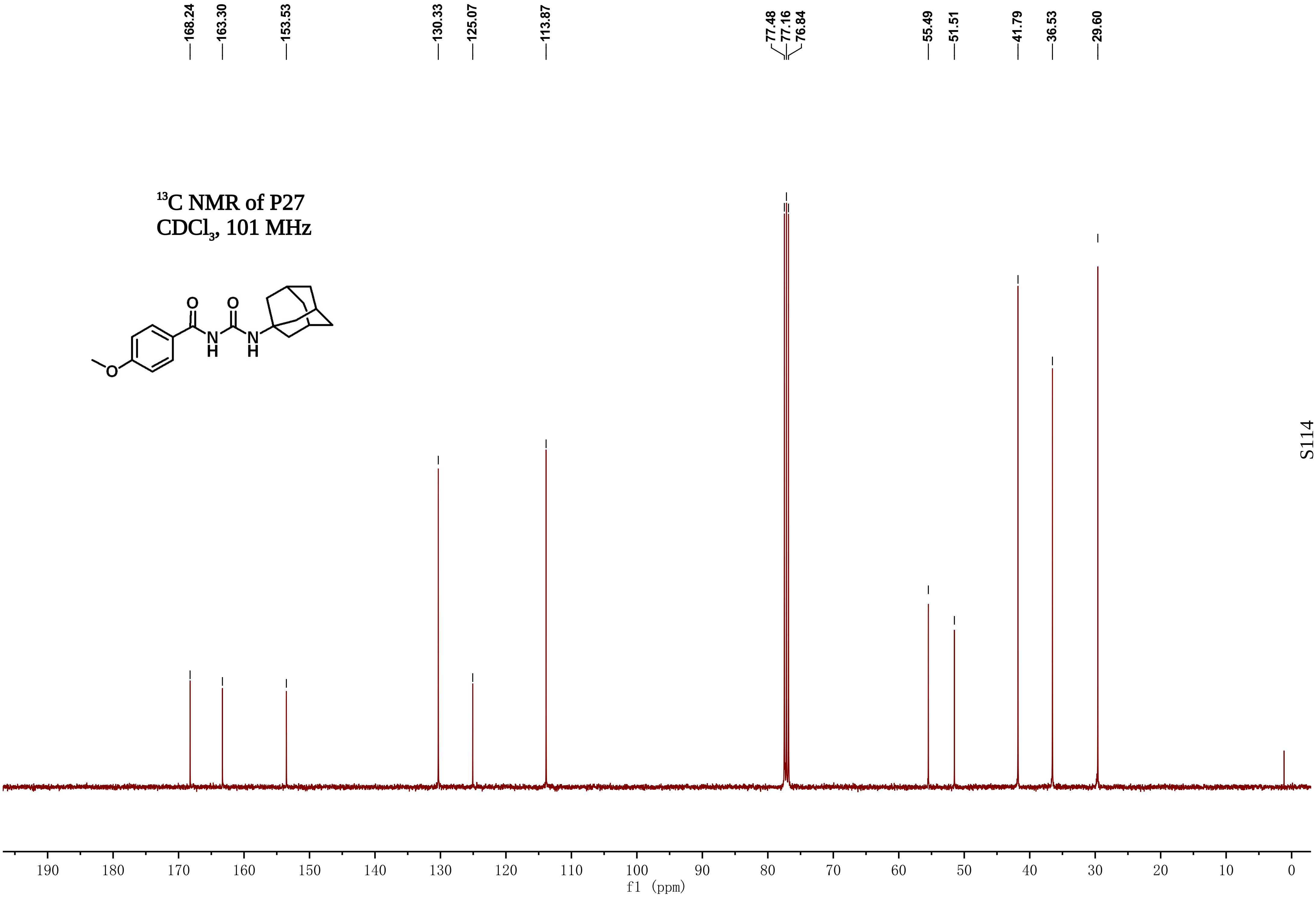

168.24
163.30
153.53
130.33
125.07
113.87
77.48
77.16
76.84
55.49
51.51
41.79
36.53
29.60
^{13}C NMR of P27
$CDCl_3$, 101 MHz
O
O
N
H
N
H
O
190
180
170
160
150
140
130
120
110
100
90
80
70
60
50
40
30
20
10
0
f1 (ppm)

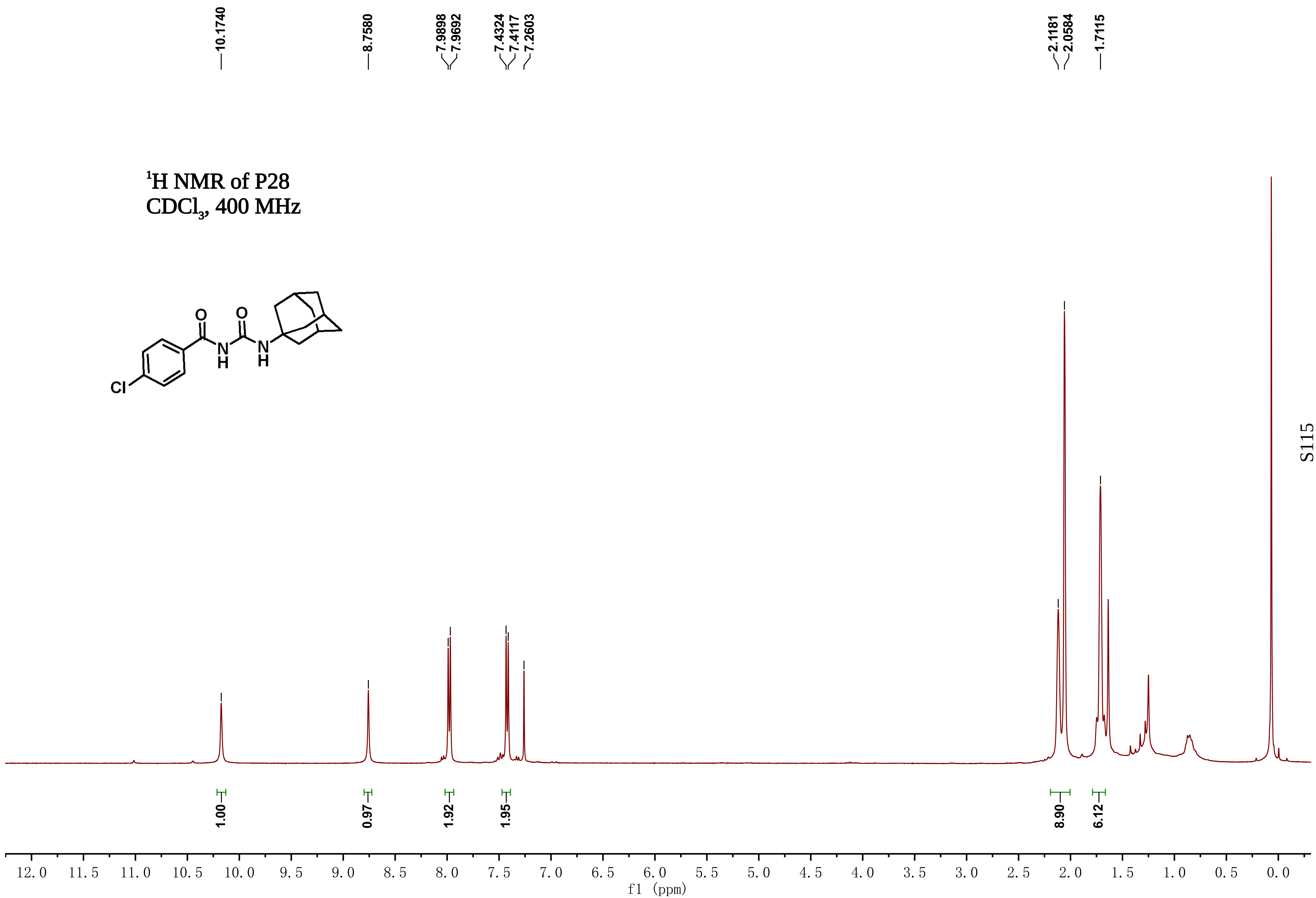
10.1740
8.7580
7.9898
7.9692
7.4324
7.4117
7.2603
2.1181
2.0584
1.7115
1H NMR of P28
CDCl3, 400 MHz
O
O
N
H
N
H
Cl
1.00
0.97
1.92
1.95
8.90
6.12
12.0 11.5 11.0 10.5 10.0 9.5 9.0 8.5 8.0 7.5 7.0 6.5 6.0 5.5 5.0 4.5 4.0 3.5 3.0 2.5 2.0 1.5 1.0 0.5 0.0
f1 (ppm)

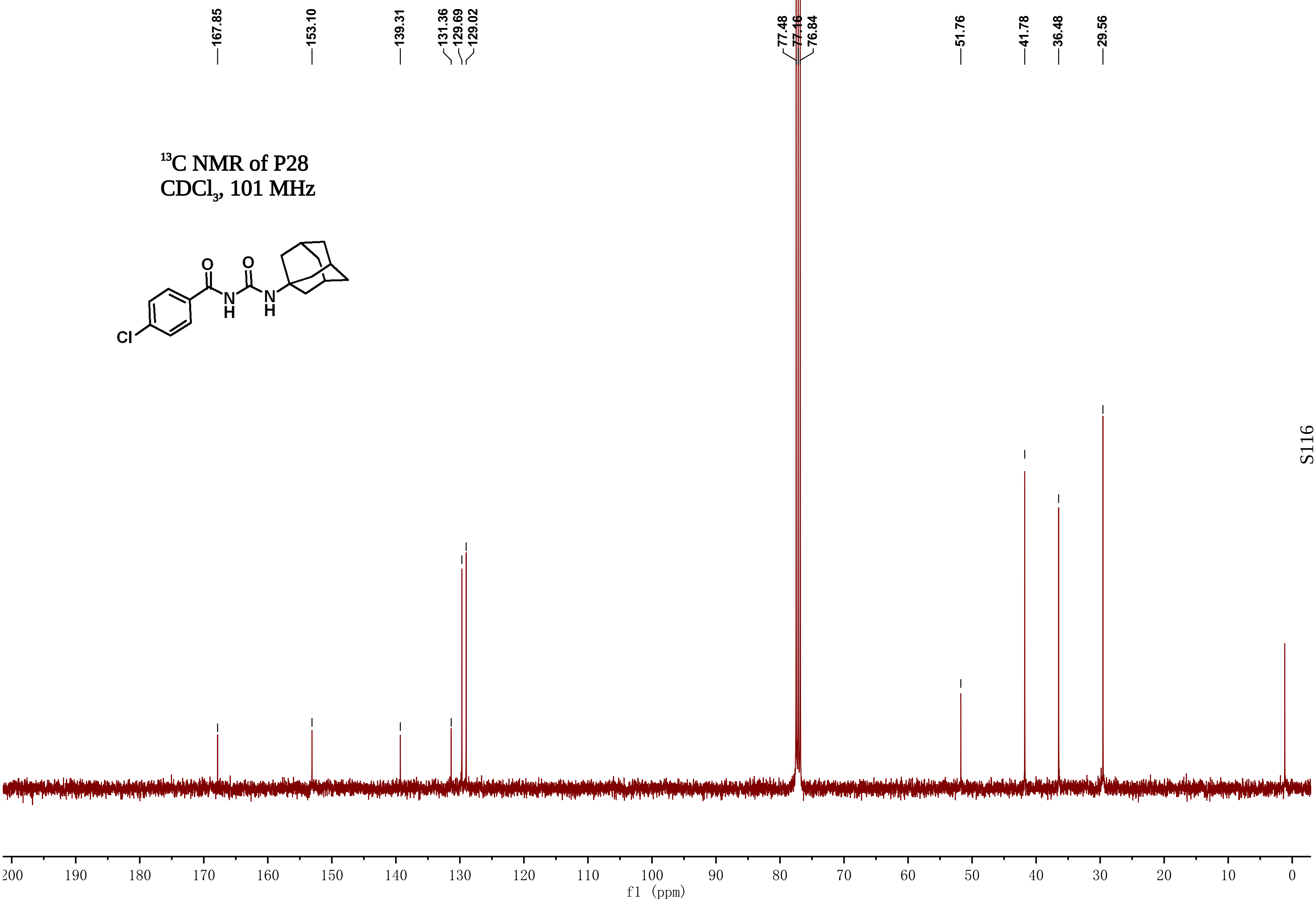
167.85
153.10
139.31
131.36
129.69
129.02
77.48
77.16
76.84
51.76
41.78
36.48
29.56
^{13}C NMR of P28
$CDCl_3$, 101 MHz
O
O
N
H
N
H
Cl
200 190 180 170 160 150 140 130 120 110 100 90 80 70 60 50 40 30 20 10 0
f1 (ppm)

^{1}H NMR of P29
$CDCl_3$, 400 MHz

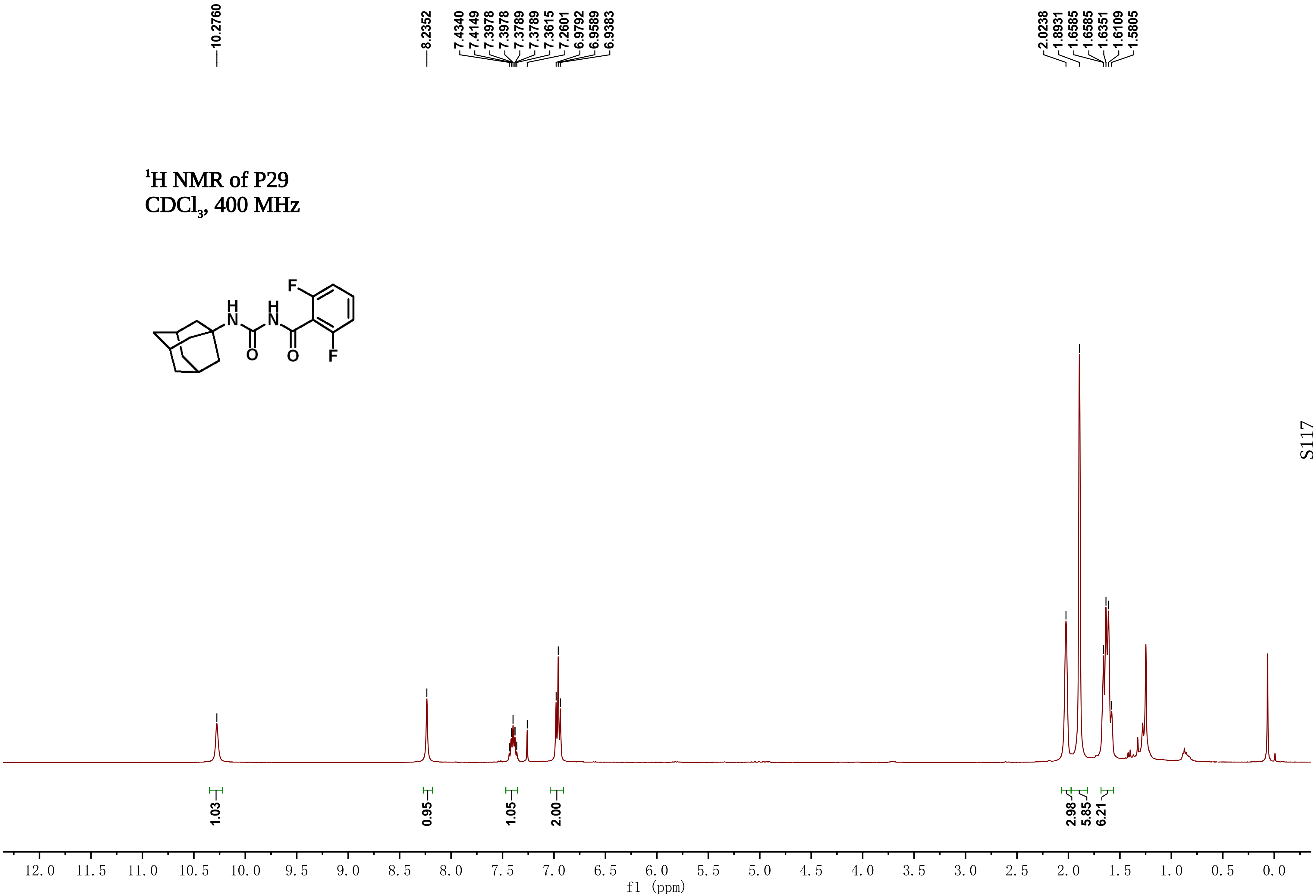

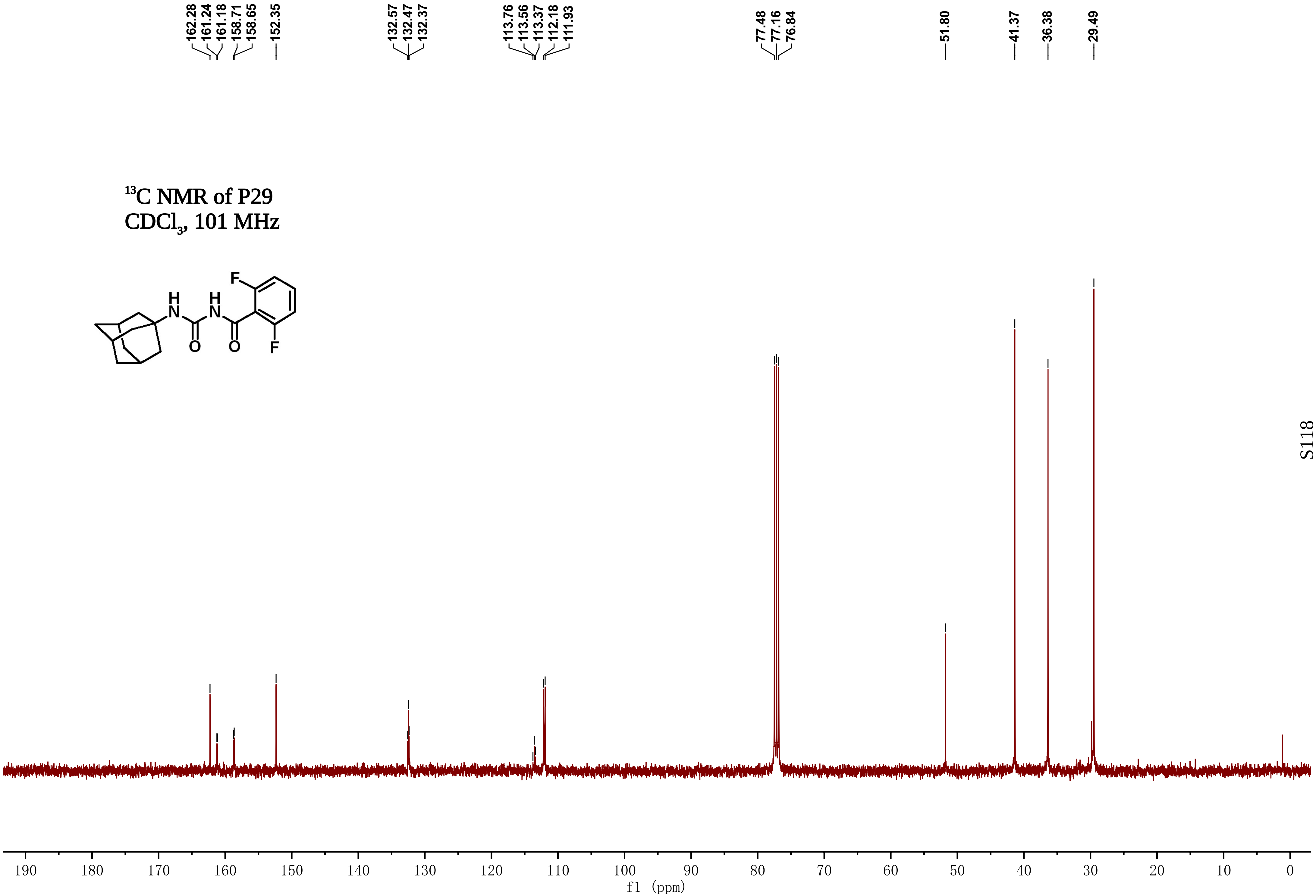

^{13}C NMR of P29
$CDCl_3$, 101 MHz
H
N
H
N
O
O
F
F
162.28
161.24
161.18
158.71
158.65
152.35
132.57
132.47
132.37
113.76
113.56
113.37
112.18
111.93
77.48
77.16
76.84
51.80
41.37
36.38
29.49
190 180 170 160 150 140 130 120 110 100 90 80 70 60 50 40 30 20 10 0
f1 (ppm)

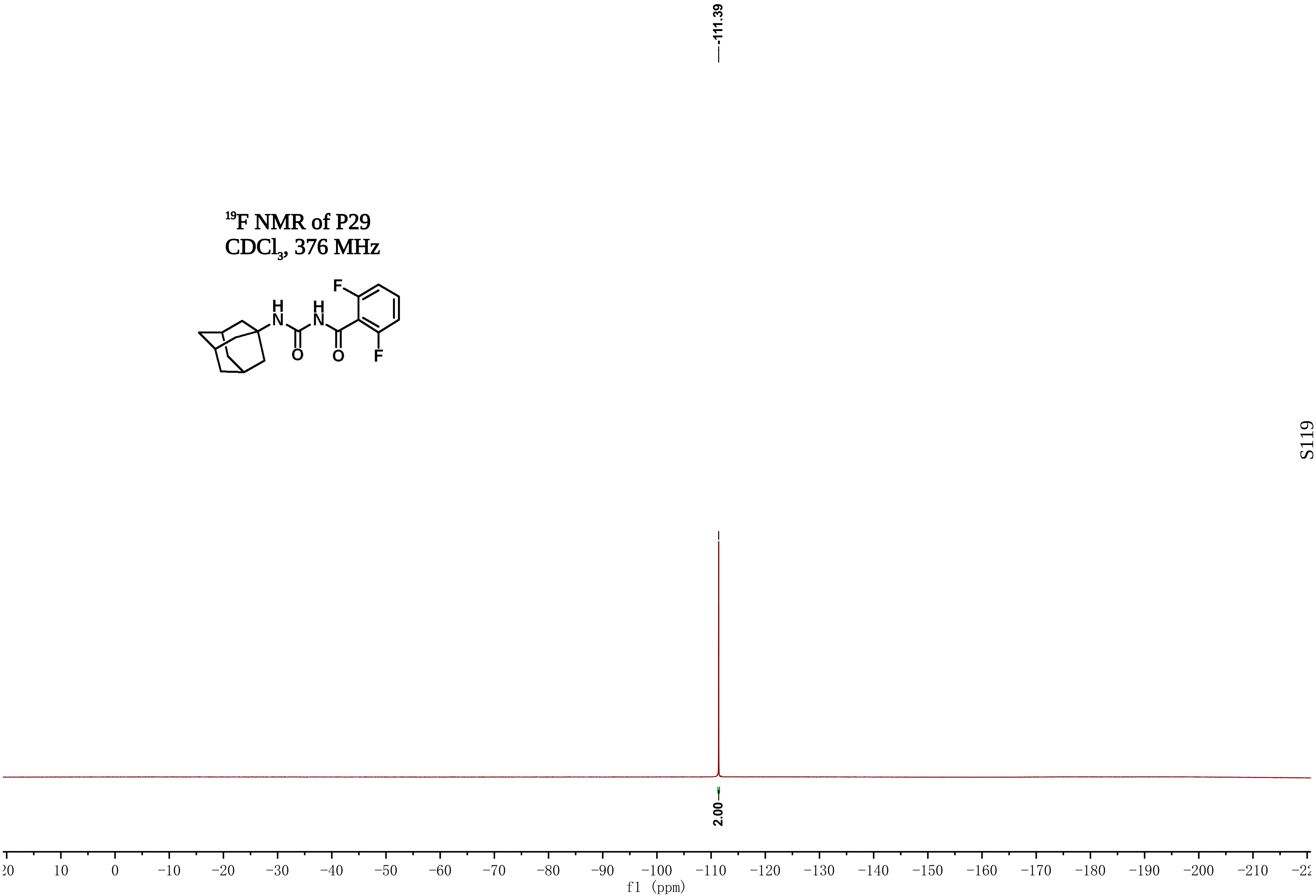
-111.39
19F NMR of P29
CDCl3, 376 MHz
H
N
H
N
O
O
F
F
2.00
20
10
0
−10
−20
−30
−40
−50
−60
−70
−80
−90
−100
−110
−120
−130
−140
−150
−160
−170
−180
−190
−200
−210
−220
f1 (ppm)

^1H NMR of P30
$CDCl_3$, 400 MHz

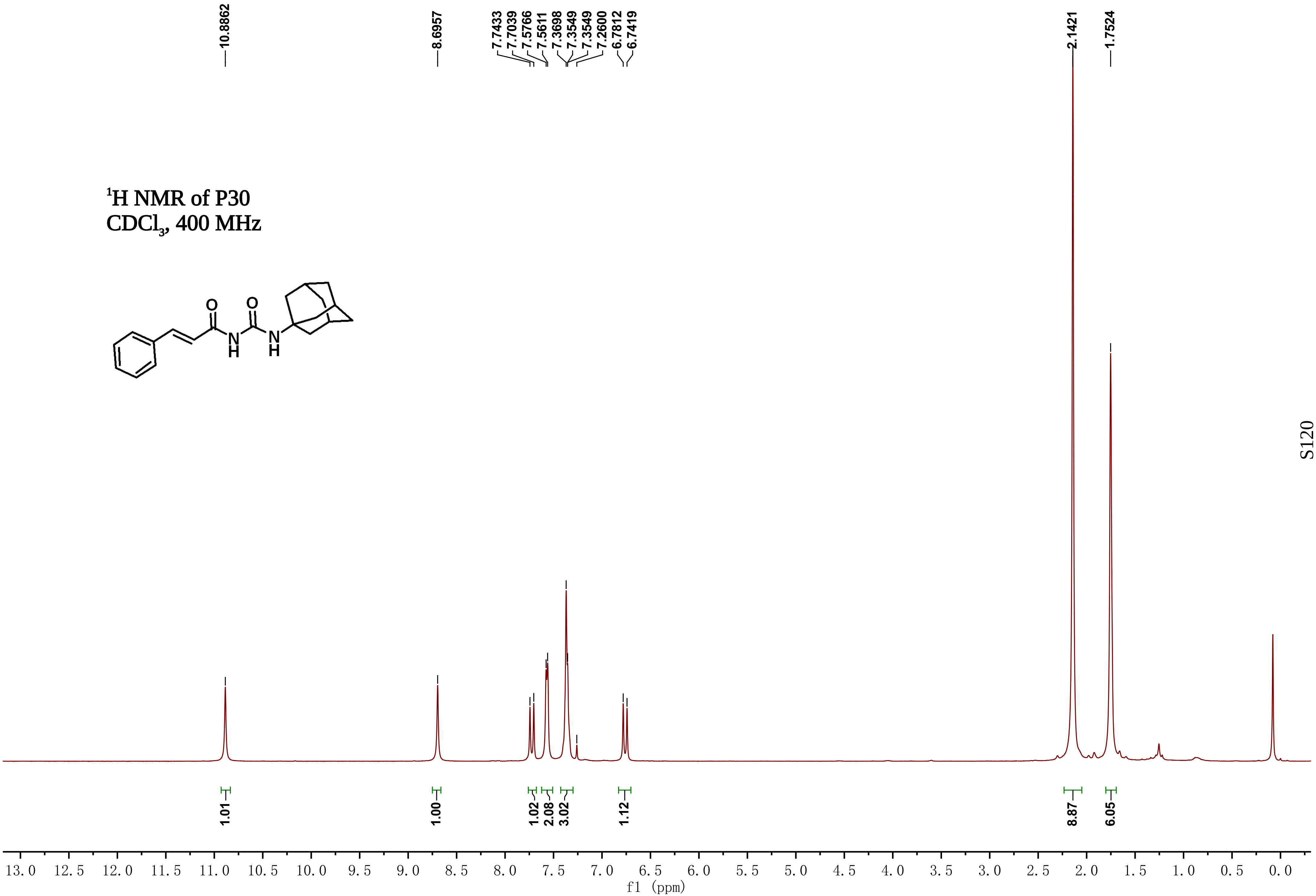

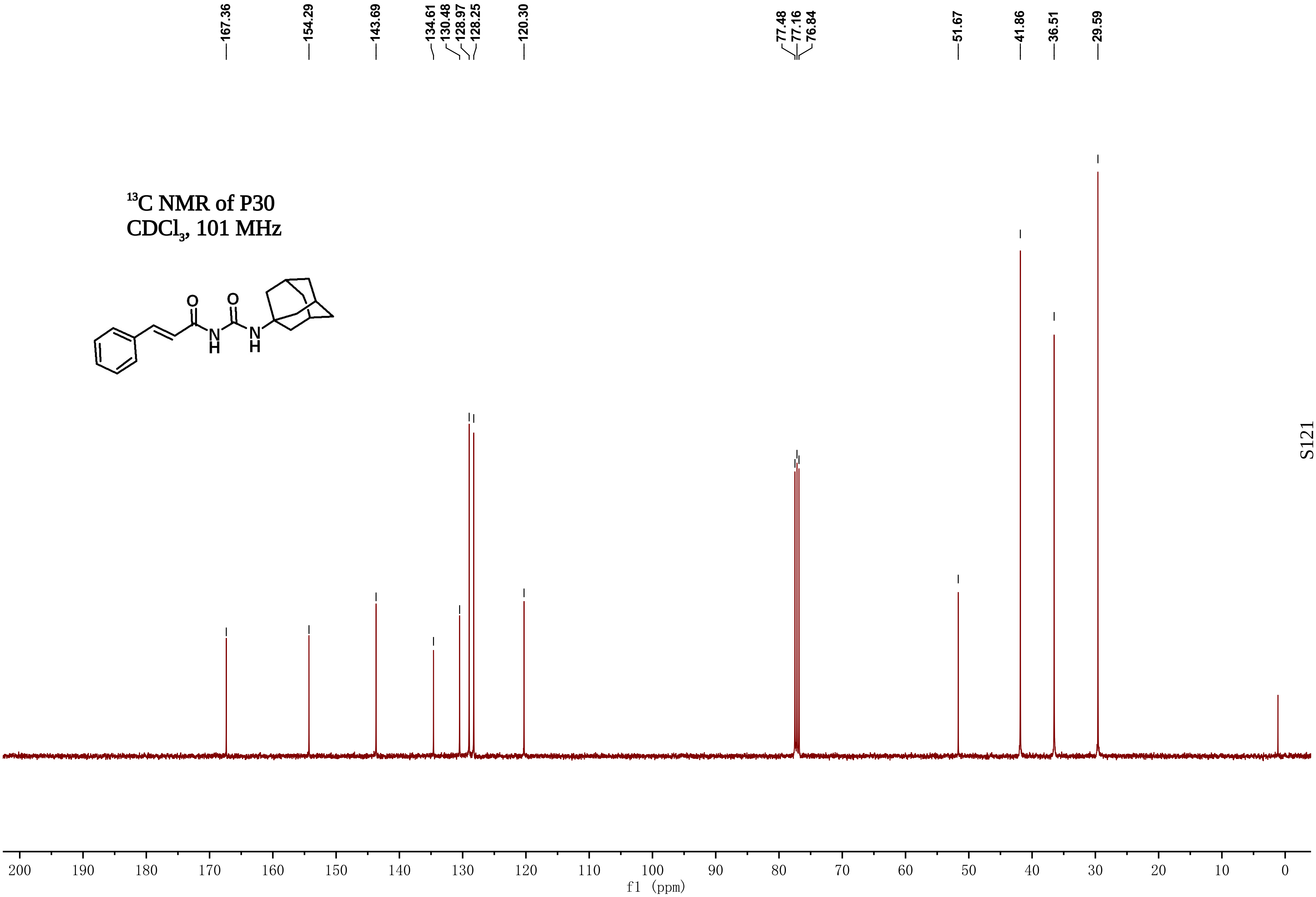

13C NMR of P30
CDCl3, 101 MHz
167.36
154.29
143.69
134.61
130.48
128.97
128.25
120.30
77.48
77.16
76.84
51.67
41.86
36.51
29.59
O
O
N
H
N
H
200 190 180 170 160 150 140 130 120 110 100 90 80 70 60 50 40 30 20 10 0
f1 (ppm)

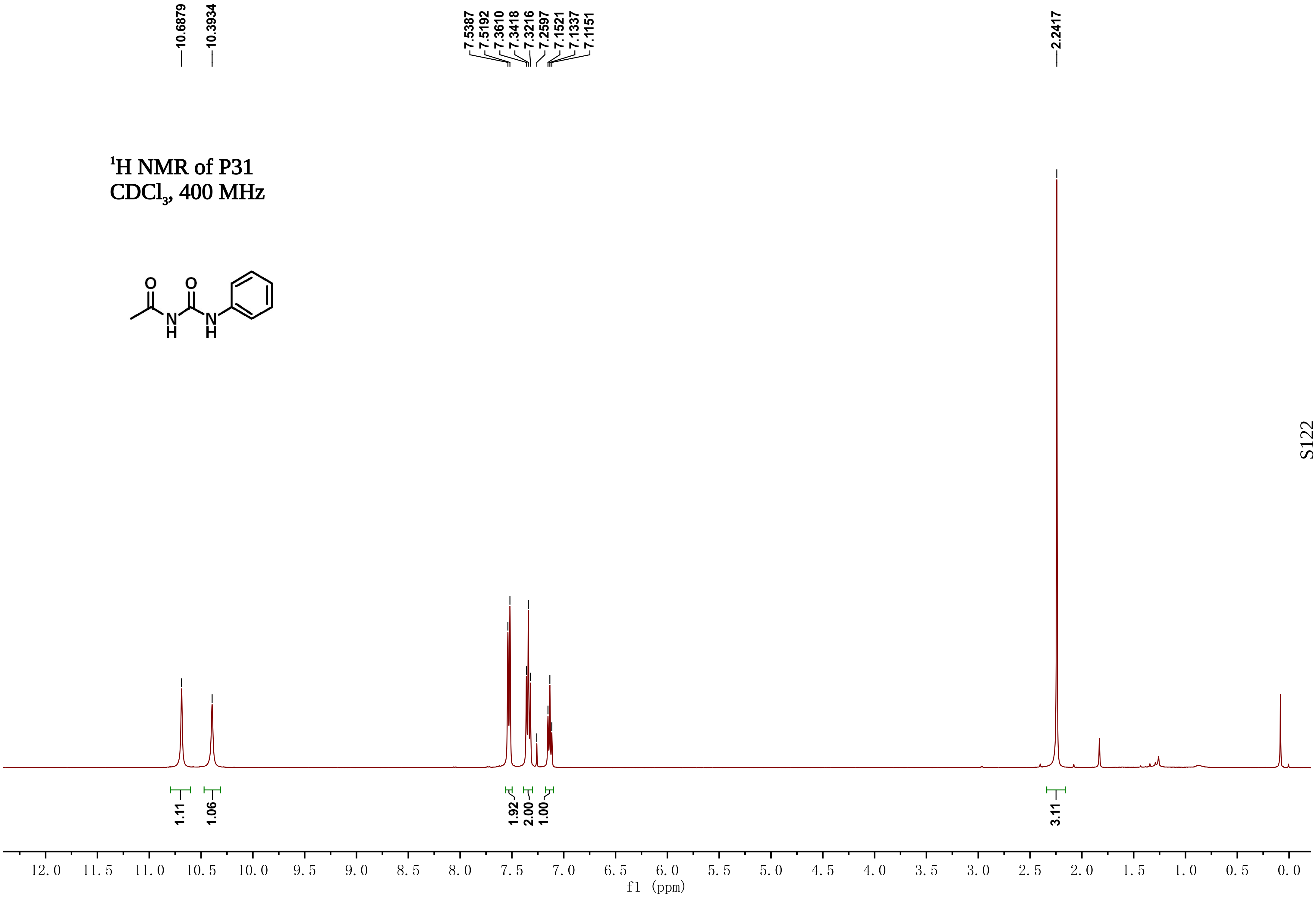

10.6879
10.3934
7.5387
7.5192
7.3610
7.3418
7.3216
7.2597
7.1521
7.1337
7.1151
2.2417
1H NMR of P31
CDCl3, 400 MHz
O
O
N
H
N
H
1.11
1.06
1.92
2.00
1.00
3.11
12.0 11.5 11.0 10.5 10.0 9.5 9.0 8.5 8.0 7.5 7.0 6.5 6.0 5.5 5.0 4.5 4.0 3.5 3.0 2.5 2.0 1.5 1.0 0.5 0.0
f1 (ppm)

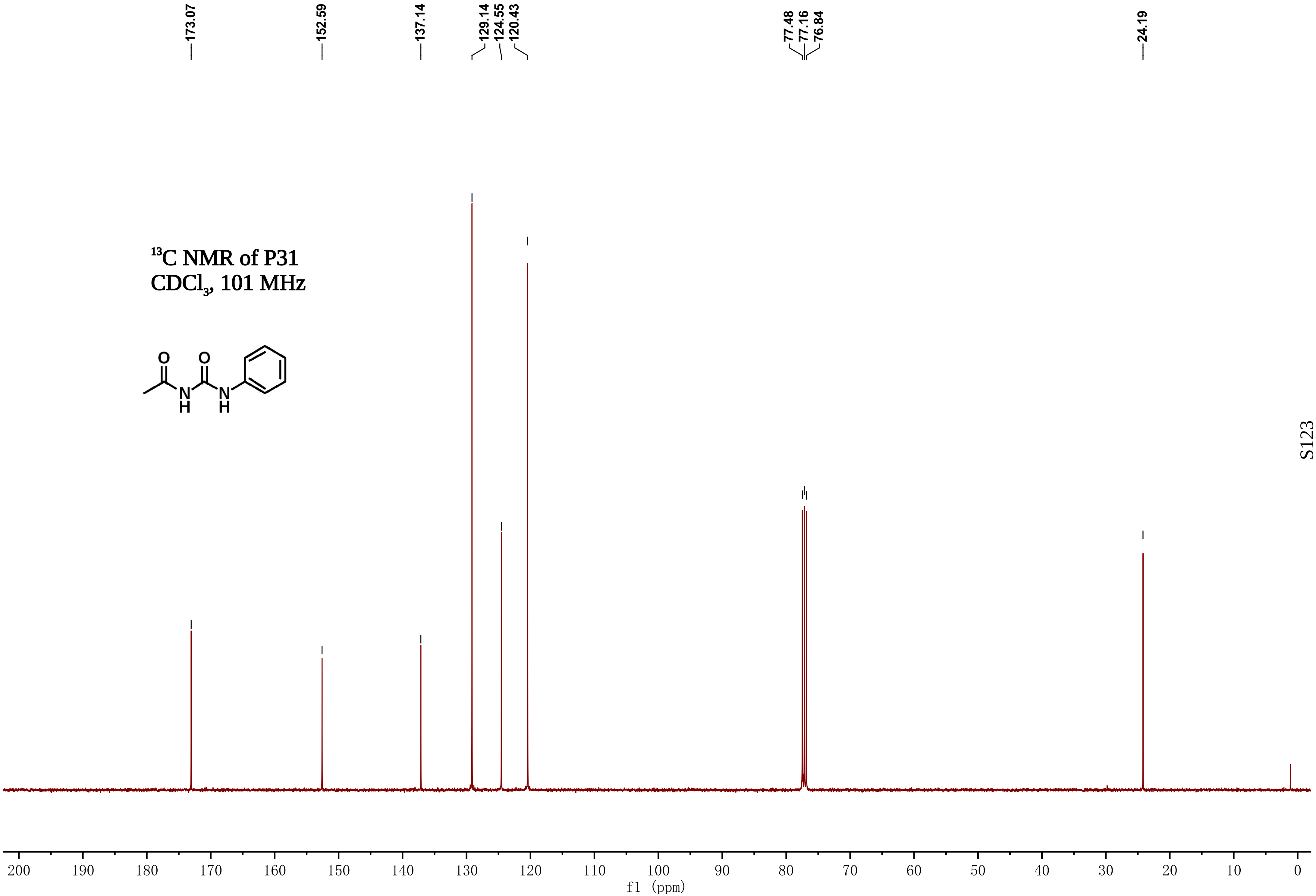
173.07
152.59
137.14
129.14
124.55
120.43
77.48
77.16
76.84
24.19
13C NMR of P31
CDCl3, 101 MHz
O
O
N
H
N
H
200
190
180
170
160
150
140
130
120
110
100
90
80
70
60
50
40
30
20
10
0
f1 (ppm)

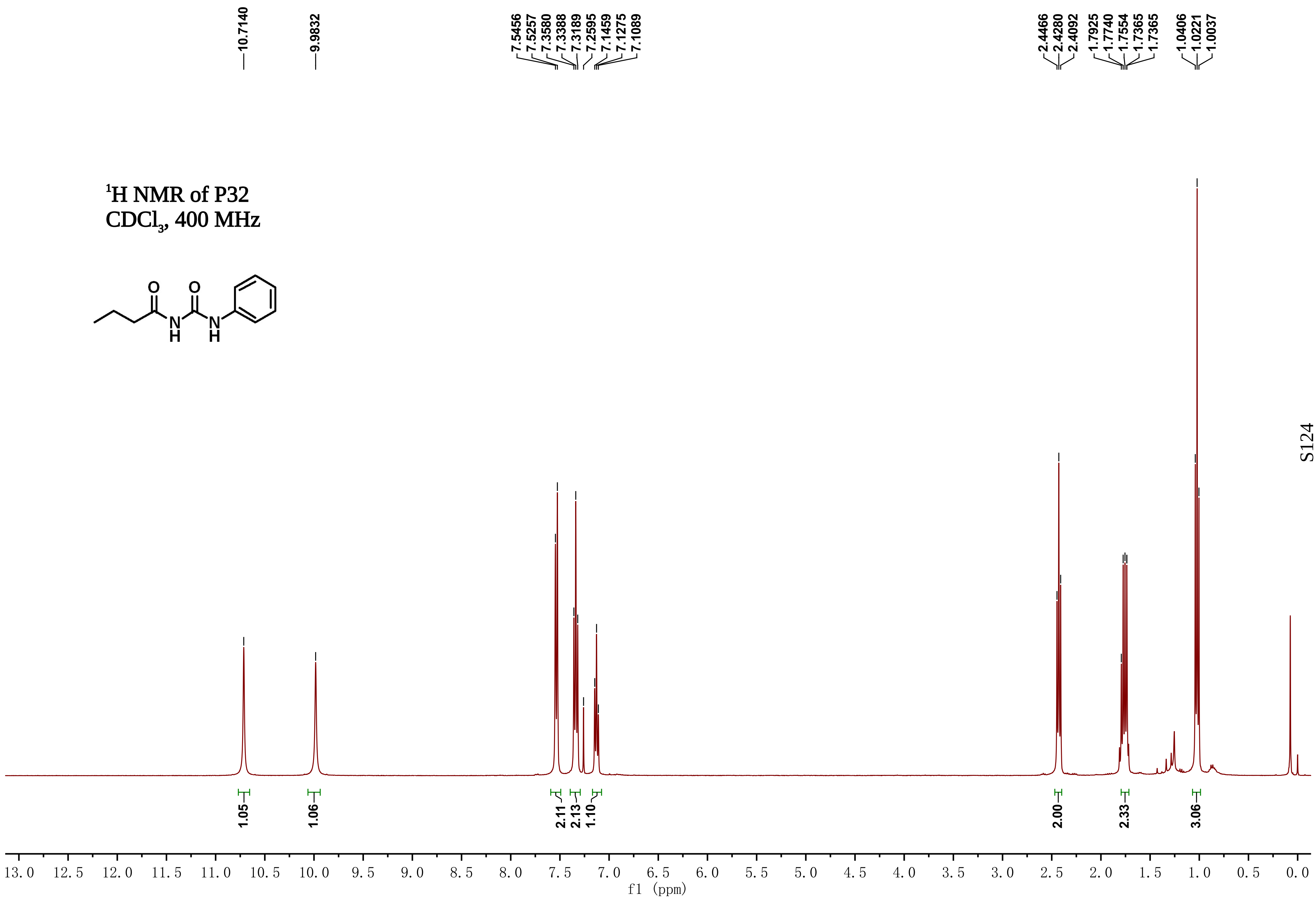

^{1}H NMR of P32
$CDCl_3$, 400 MHz
10.7140
9.9832
7.5456
7.5257
7.3580
7.3388
7.3189
7.2595
7.1459
7.1275
7.1089
2.4466
2.4280
2.4092
1.7925
1.7740
1.7554
1.7365
1.7365
1.0406
1.0221
1.0037
1.05
1.06
2.11
2.13
1.10
2.00
2.33
3.06
13.0 12.5 12.0 11.5 11.0 10.5 10.0 9.5 9.0 8.5 8.0 7.5 7.0 6.5 6.0 5.5 5.0 4.5 4.0 3.5 3.0 2.5 2.0 1.5 1.0 0.5 0.0
f1 (ppm)

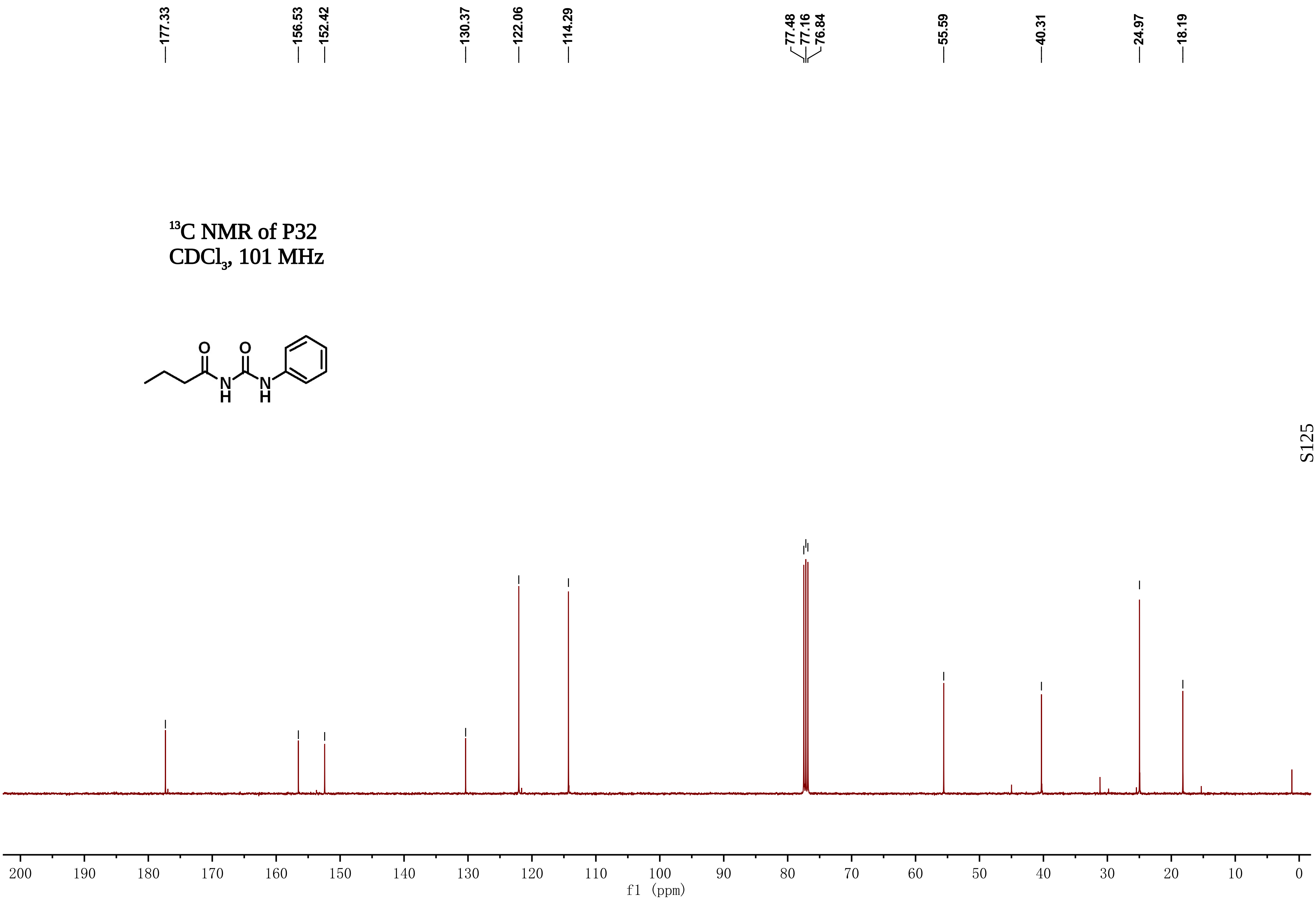
177.33
156.53
152.42
130.37
122.06
114.29
77.48
77.16
76.84
55.59
40.31
24.97
18.19
^{13}C NMR of P32
$CDCl_3$, 101 MHz
O
O
N
H
N
H
200 190 180 170 160 150 140 130 120 110 100 90 80 70 60 50 40 30 20 10 0
f1 (ppm)

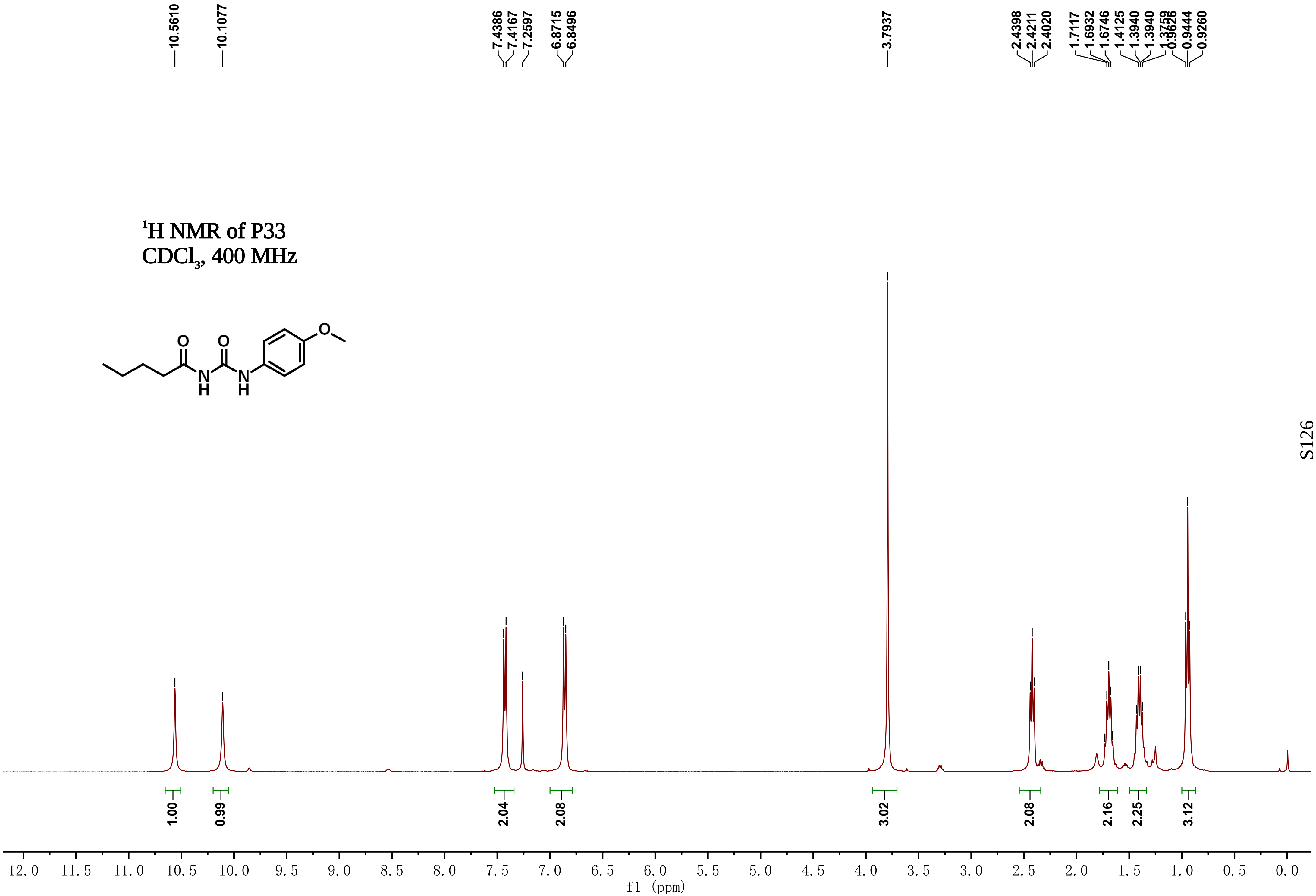
1H NMR of P33
CDCl3, 400 MHz
O
O
N
H
N
H
O
10.5610
10.1077
7.4386
7.4167
7.2597
6.8715
6.8496
3.7937
2.4398
2.4211
2.4020
1.7117
1.6932
1.6746
1.4125
1.3940
1.3940
1.3759
0.9626
0.9444
0.9260
1.00
0.99
2.04
2.08
3.02
2.08
2.16
2.25
3.12
12.0 11.5 11.0 10.5 10.0 9.5 9.0 8.5 8.0 7.5 7.0 6.5 6.0 5.5 5.0 4.5 4.0 3.5 3.0 2.5 2.0 1.5 1.0 0.5 0.0
f1 (ppm)

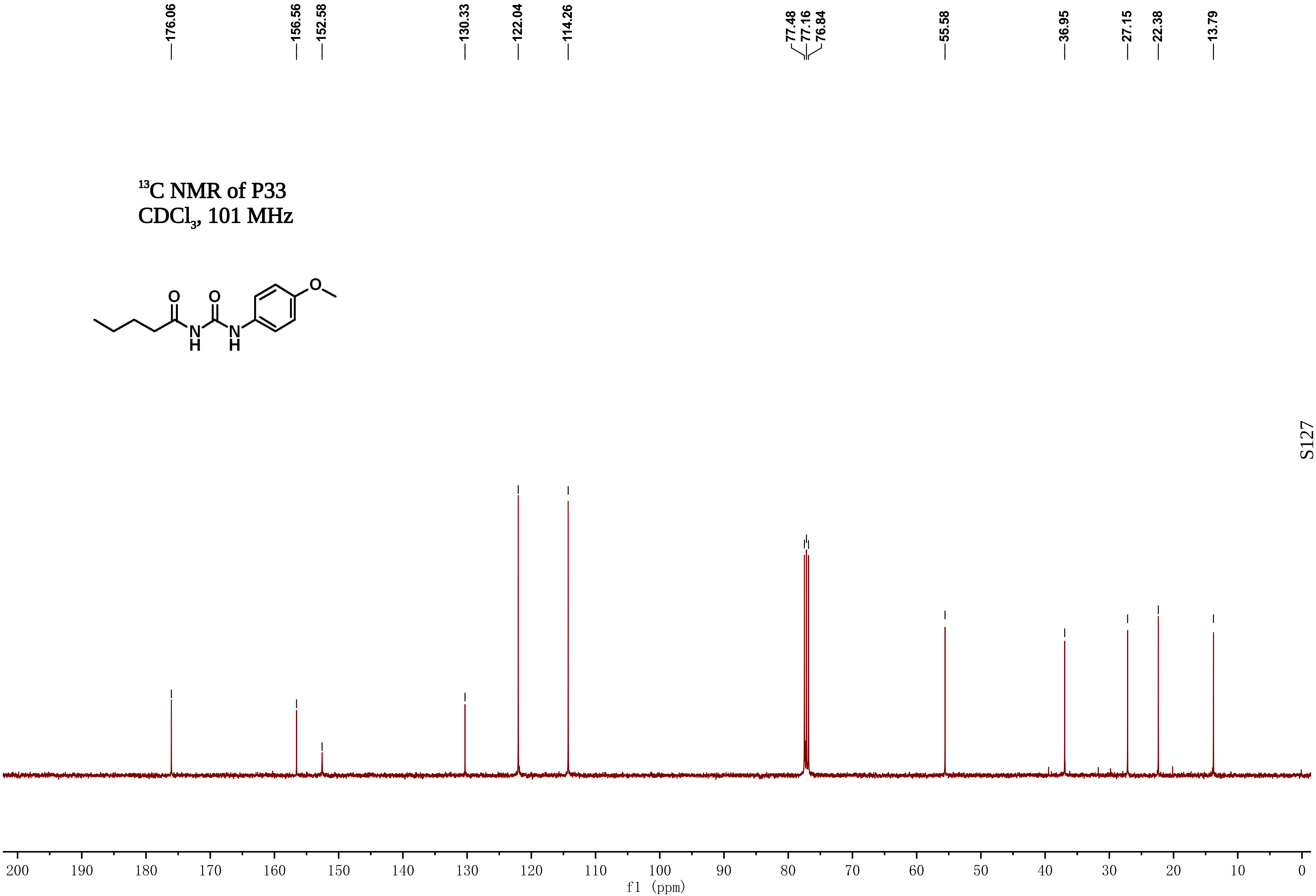
176.06
156.56
152.58
130.33
122.04
114.26
77.48
77.16
76.84
55.58
36.95
27.15
22.38
13.79
^{13}C NMR of P33
$CDCl_3$, 101 MHz
O
O
O
N
H
N
H
200 190 180 170 160 150 140 130 120 110 100 90 80 70 60 50 40 30 20 10 0
f1 (ppm)

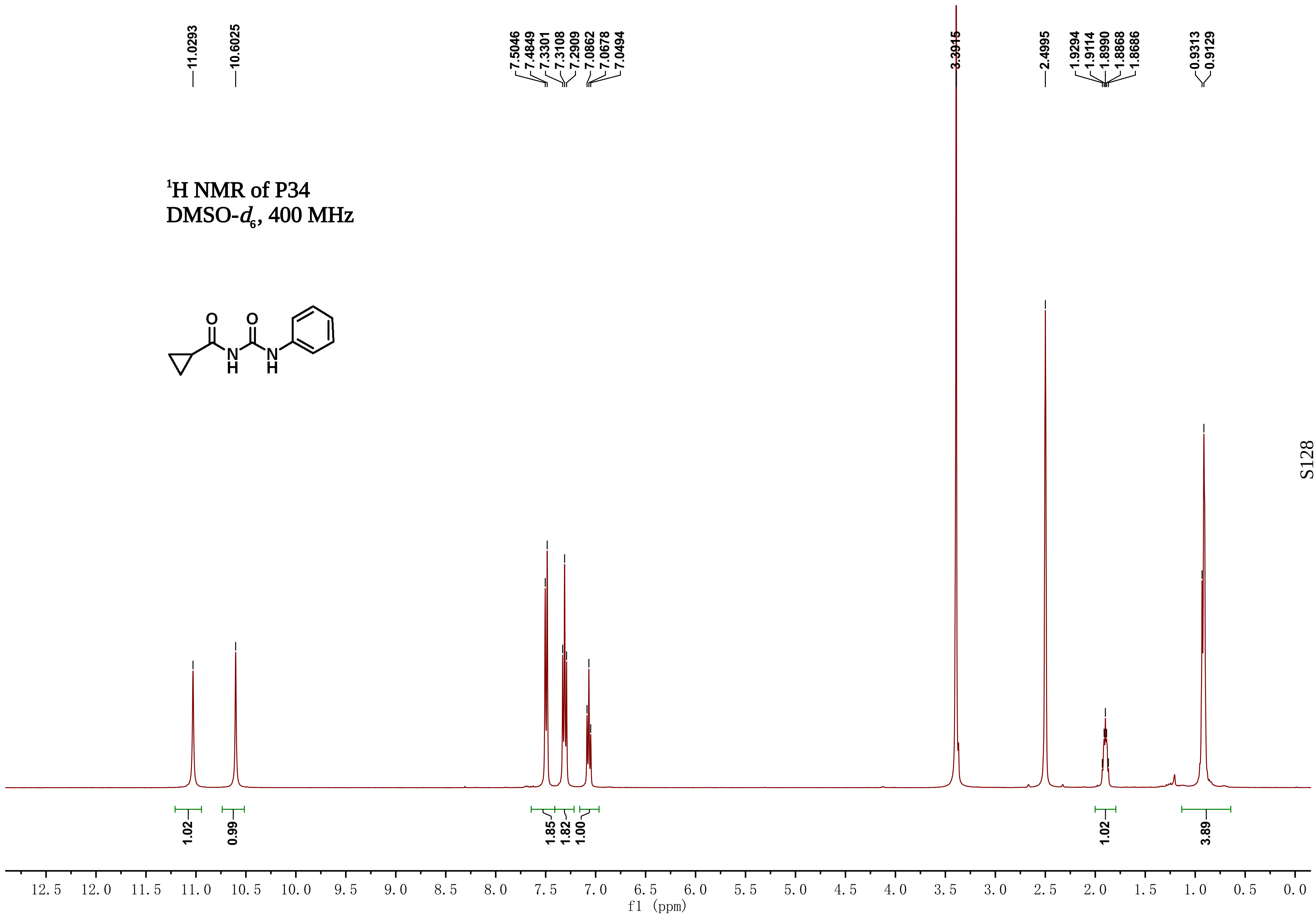

1H NMR of P34
DMSO-d6, 400 MHz
11.0293
10.6025
7.5046
7.4849
7.3301
7.3108
7.2909
7.0862
7.0678
7.0494
3.3915
2.4995
1.9294
1.9114
1.8990
1.8868
1.8686
0.9313
0.9129
1.02
0.99
1.85
1.82
1.00
1.02
3.89
O
O
N
H
N
H
12.5 12.0 11.5 11.0 10.5 10.0 9.5 9.0 8.5 8.0 7.5 7.0 6.5 6.0 5.5 5.0 4.5 4.0 3.5 3.0 2.5 2.0 1.5 1.0 0.5 0.0
f1 (ppm)

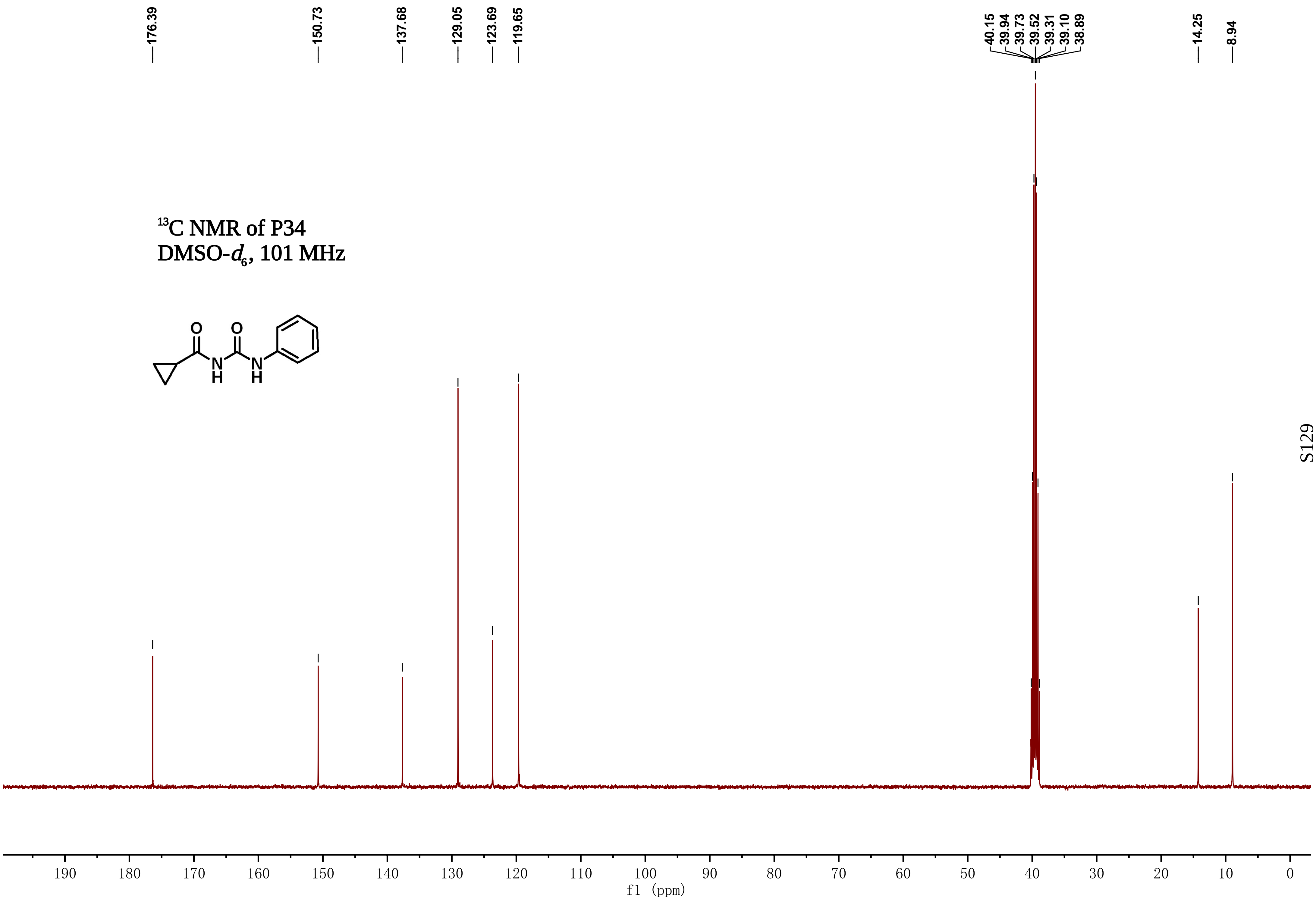

^{13}C NMR of P34
DMSO-d_6, 101 MHz
176.39
150.73
137.68
129.05
123.69
119.65
40.15
39.94
39.73
39.52
39.31
39.10
38.89
14.25
8.94
O
O
N
H
N
H
190 180 170 160 150 140 130 120 110 100 90 80 70 60 50 40 30 20 10 0
f1 (ppm)

^{1}H NMR of P35
$CDCl_3$, 400 MHz

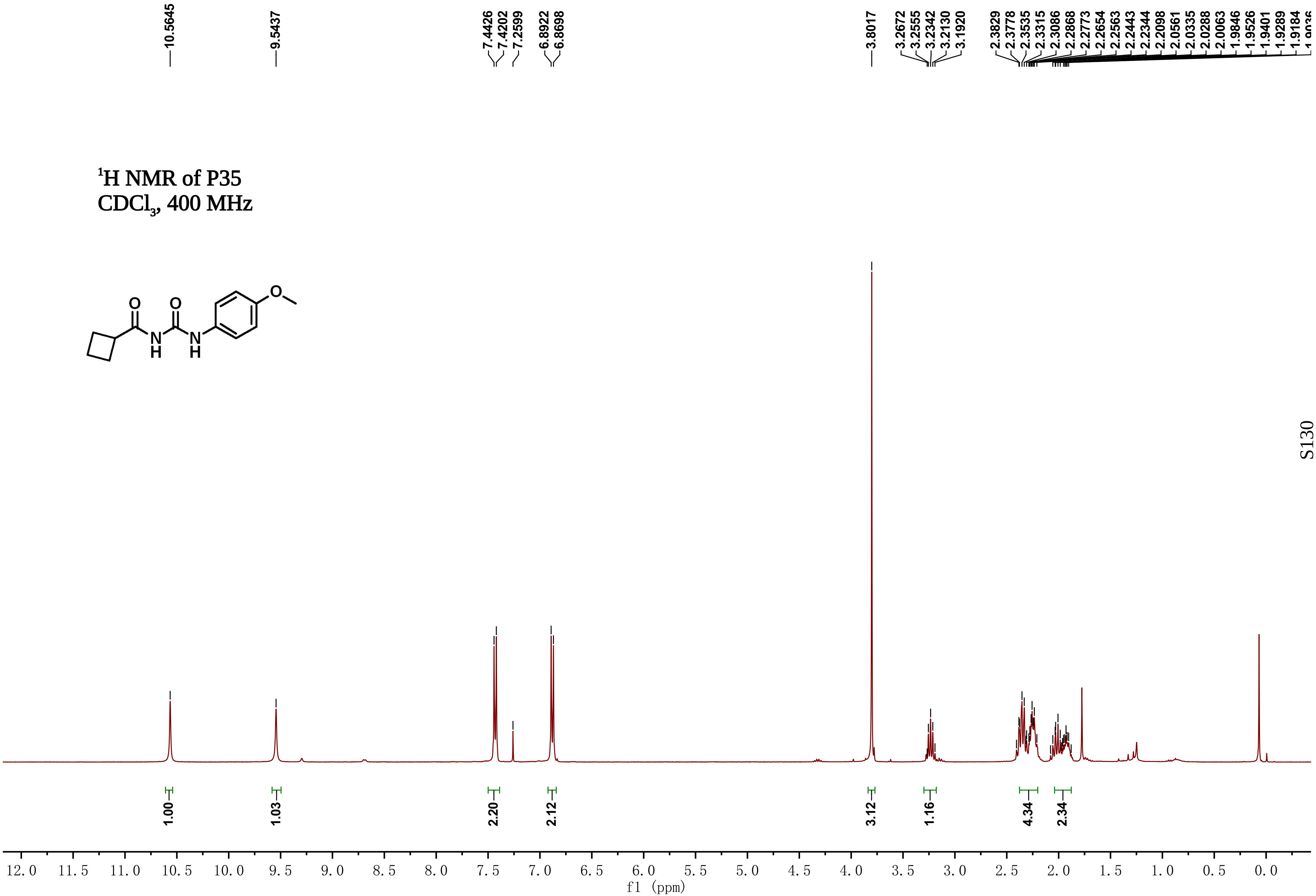

^{13}C NMR of P35
$CDCl_3$, 101 MHz

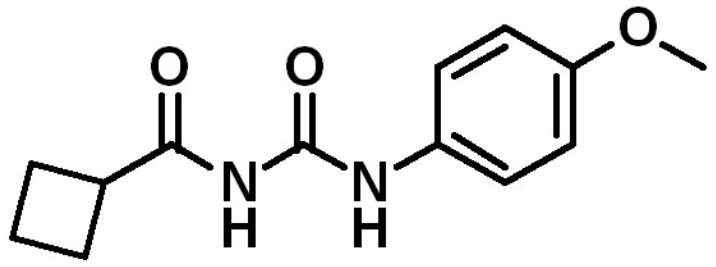

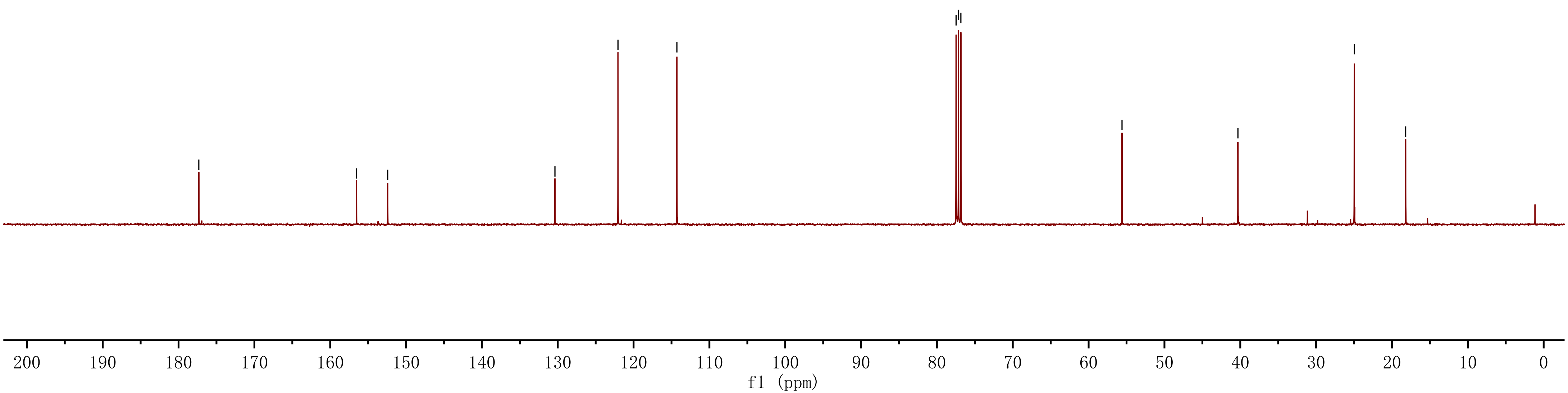

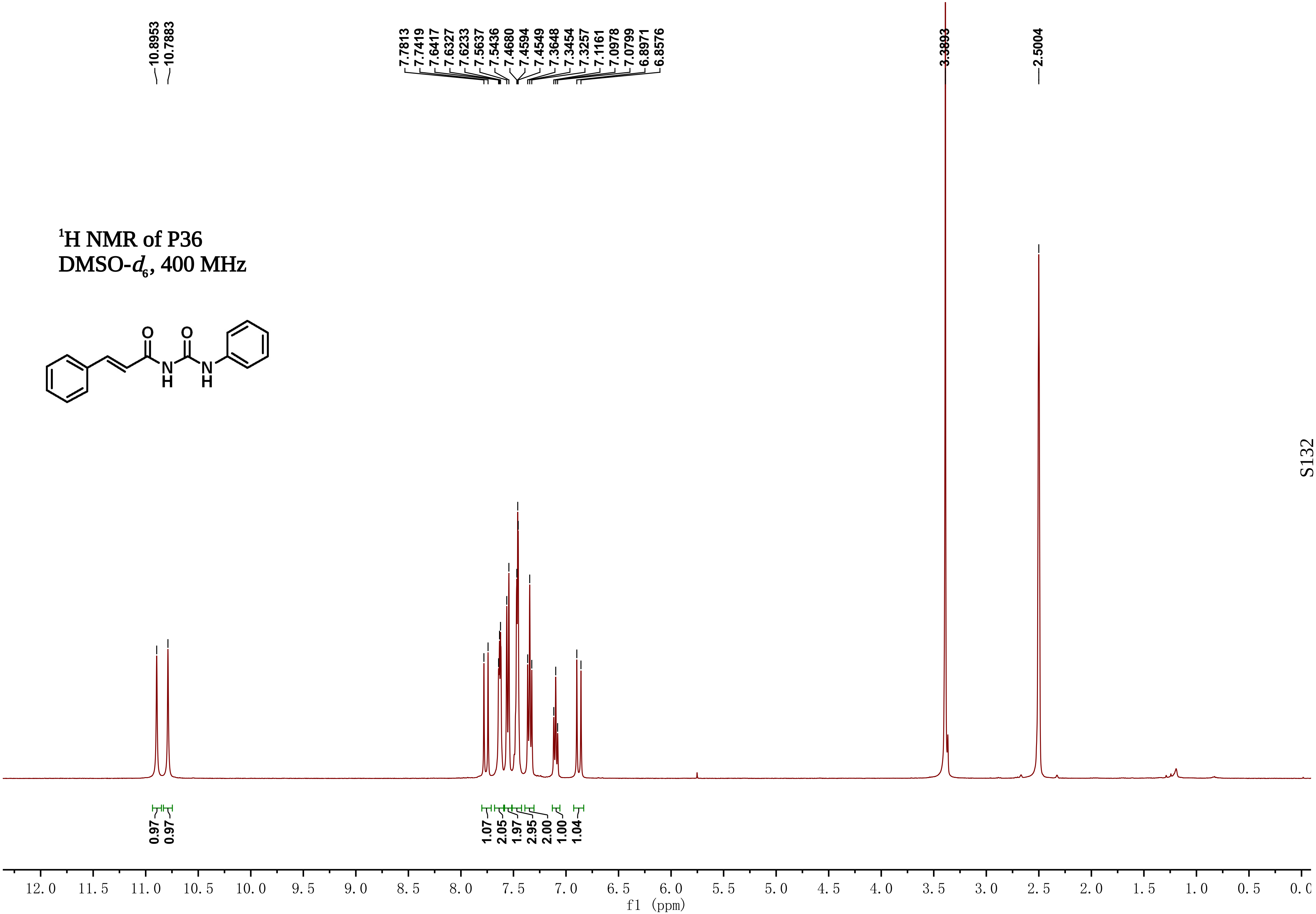

10.8953
10.7883
7.7813
7.7419
7.6417
7.6327
7.6233
7.5637
7.5436
7.4680
7.4594
7.4549
7.3648
7.3454
7.3257
7.1161
7.0978
7.0799
6.8971
6.8576
3.3893
2.5004
¹H NMR of P36
DMSO-d6, 400 MHz
0.97
0.97
1.07
2.05
1.97
2.95
2.00
1.00
1.04
12.0 11.5 11.0 10.5 10.0 9.5 9.0 8.5 8.0 7.5 7.0 6.5 6.0 5.5 5.0 4.5 4.0 3.5 3.0 2.5 2.0 1.5 1.0 0.5 0.0
f1 (ppm)

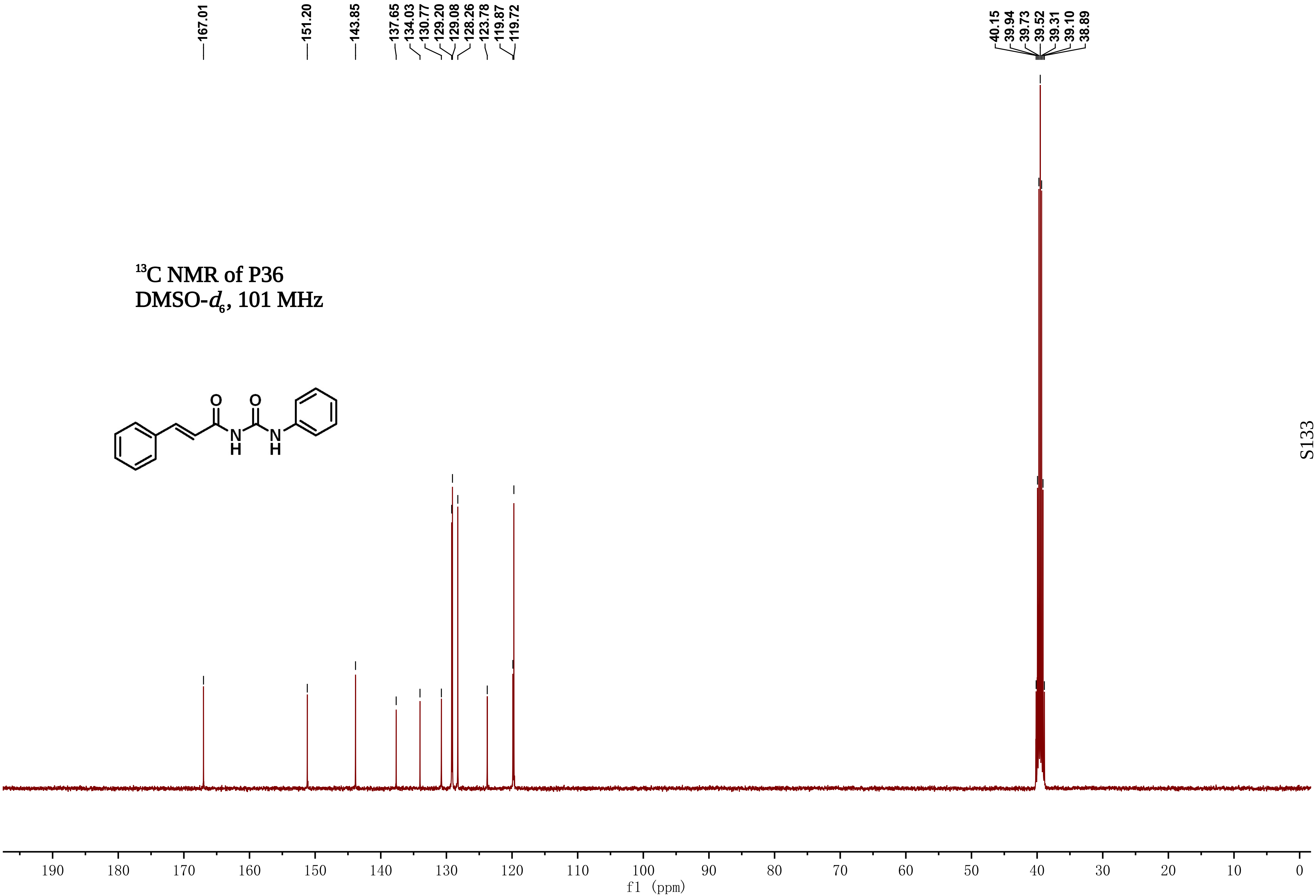
167.01
151.20
143.85
137.65
134.03
130.77
129.20
129.08
128.26
123.78
119.87
119.72
40.15
39.94
39.73
39.52
39.31
39.10
38.89
^{13}C NMR of P36
DMSO-d_6, 101 MHz
O
O
N
H
N
H
190
180
170
160
150
140
130
120
110
100
90
80
70
60
50
40
30
20
10
0
f1 (ppm)

^{1}H NMR of P37
DMSO-d_6, 400 MHz

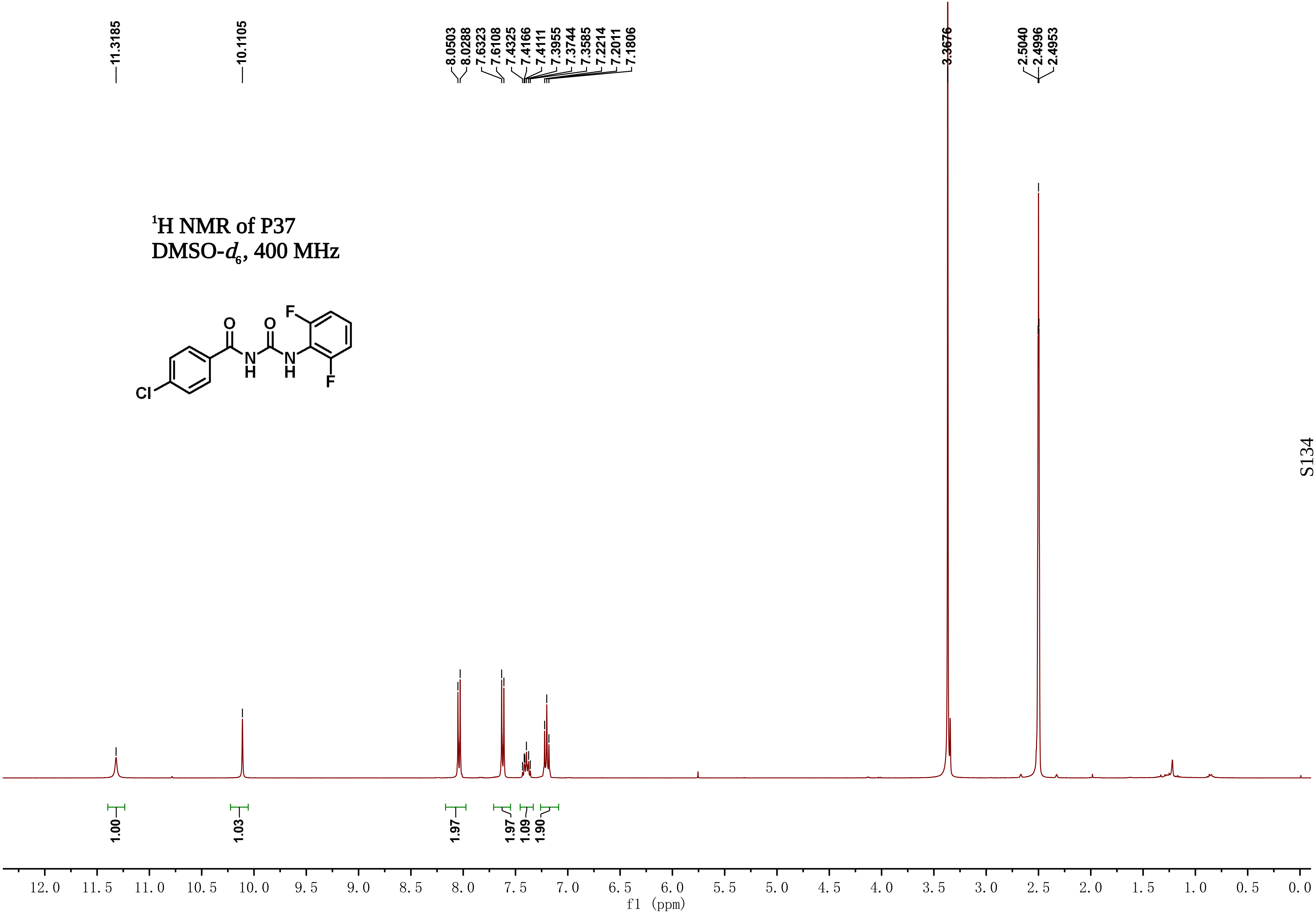

^{13}C NMR of P37
DMSO-d_6, 101 MHz

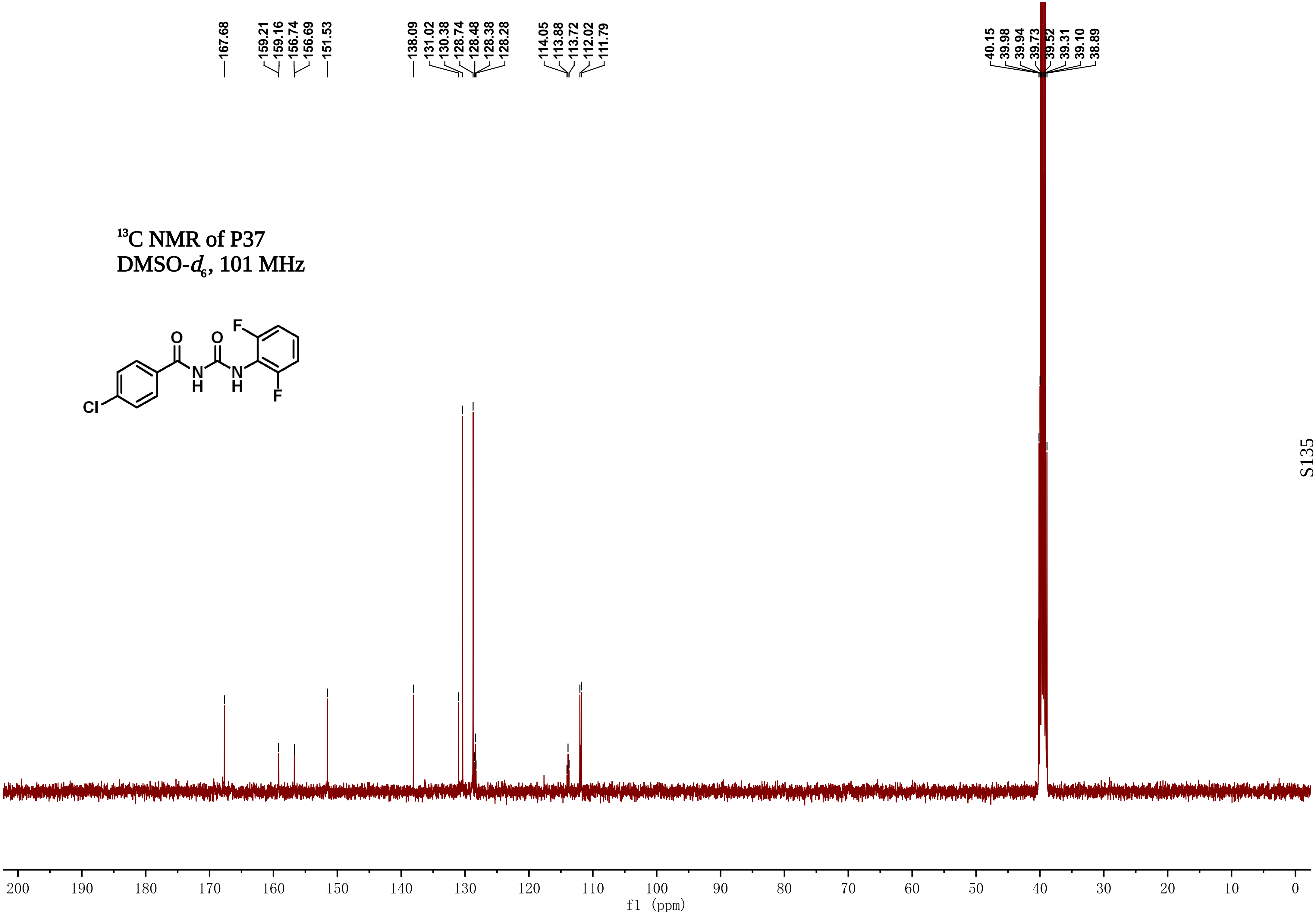

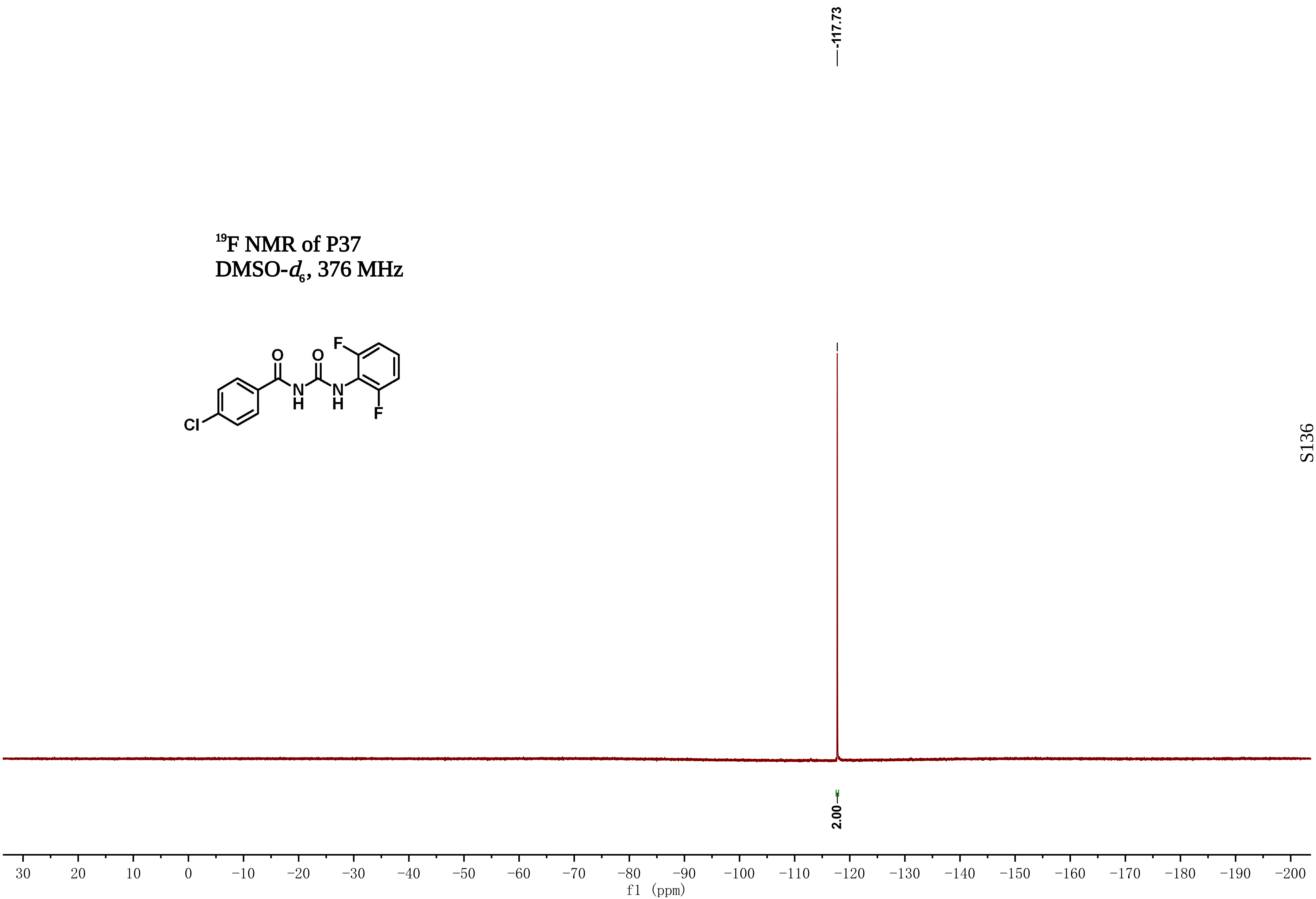

-117.73
^{19}F NMR of P37
DMSO-d_6, 376 MHz
O
O
F
N
H
N
H
F
Cl
2.00
30 20 10 0 −10 −20 −30 −40 −50 −60 −70 −80 −90 −100 −110 −120 −130 −140 −150 −160 −170 −180 −190 −200
f1 (ppm)

^{1}H NMR of P38
DMSO-d_6, 400 MHz

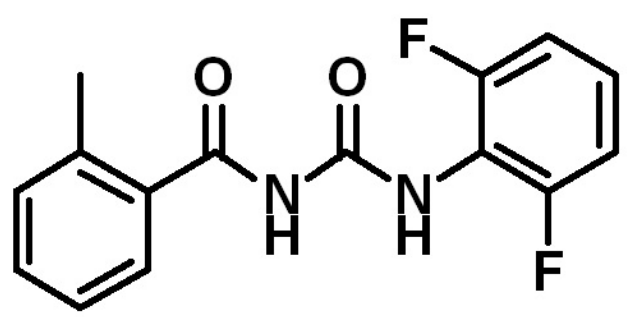

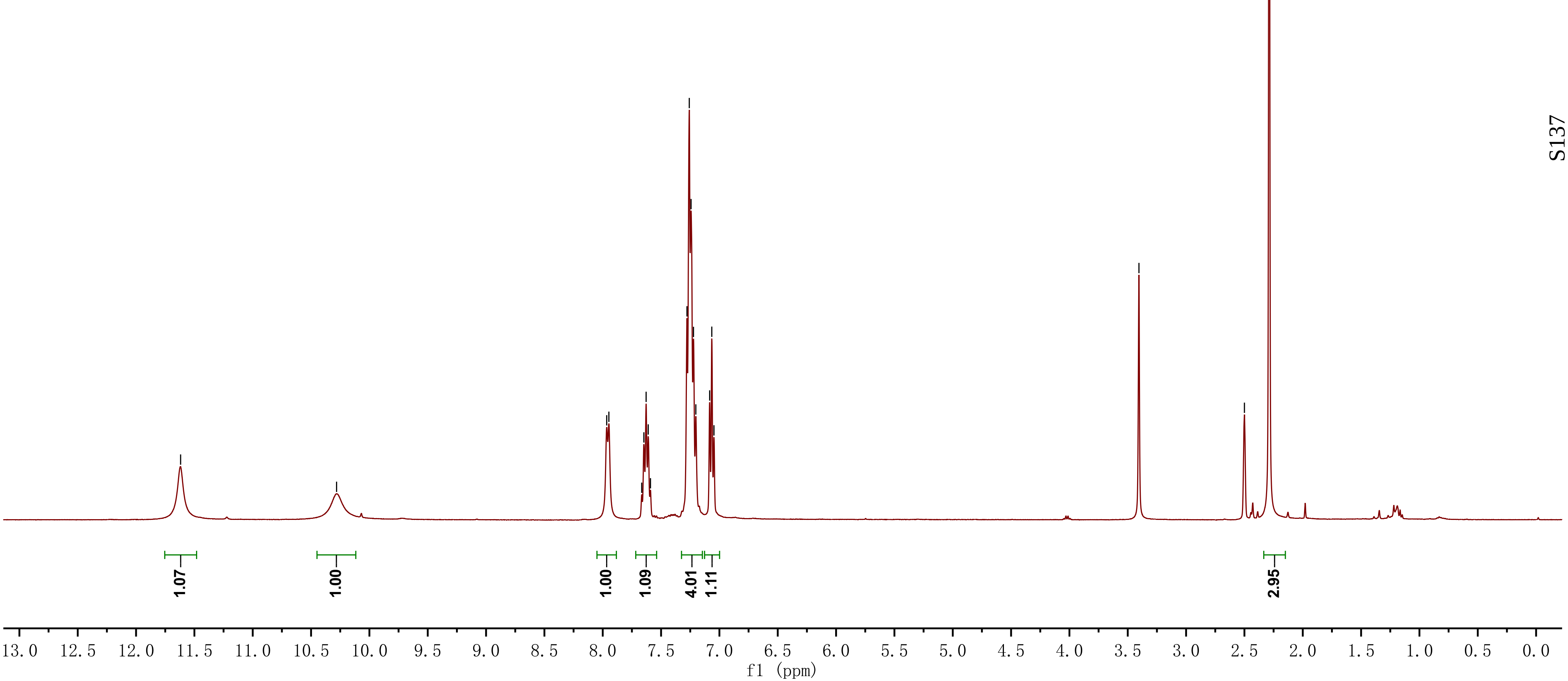

^{13}C NMR of P38

DMSO-d_6, 101 MHz

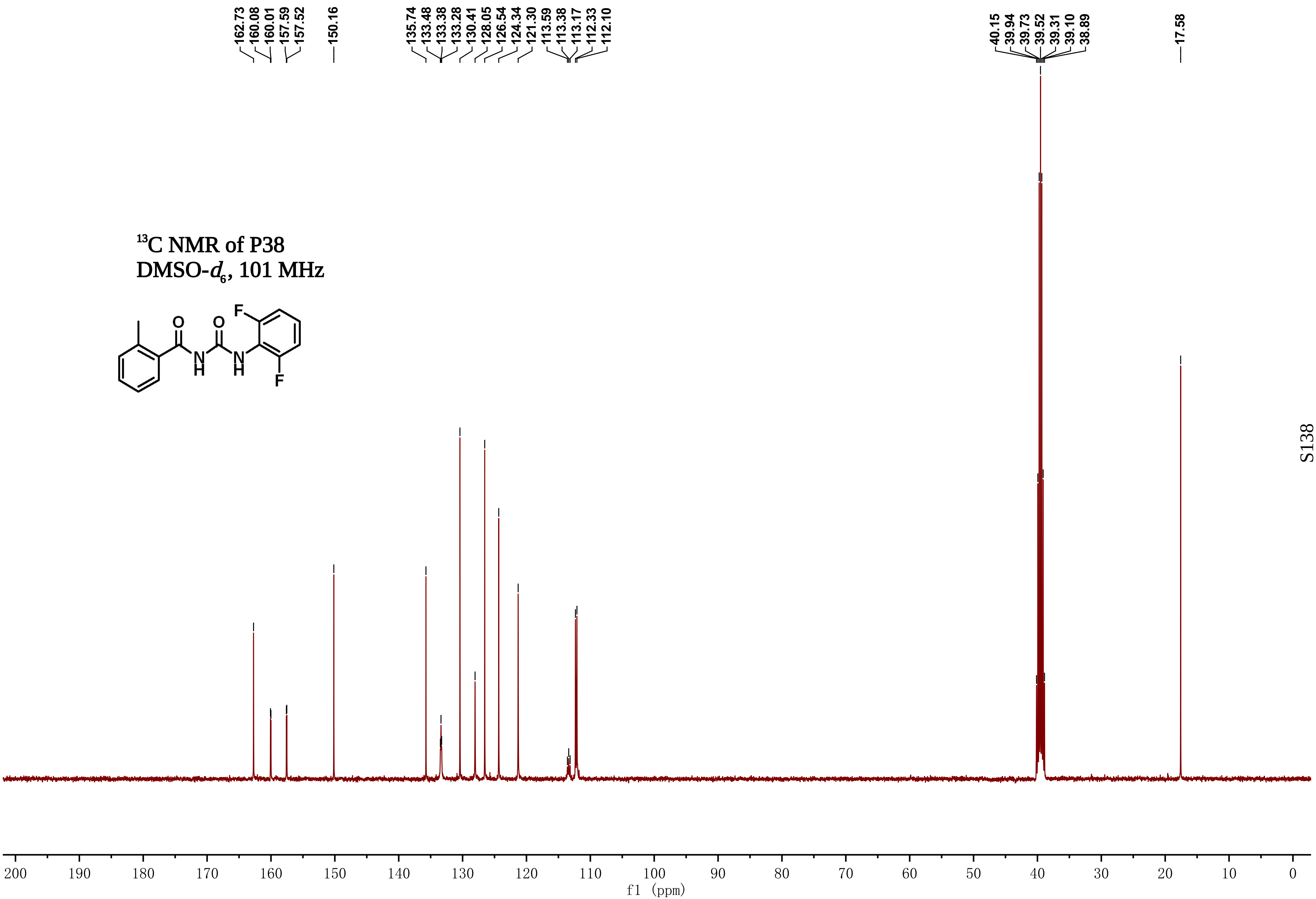

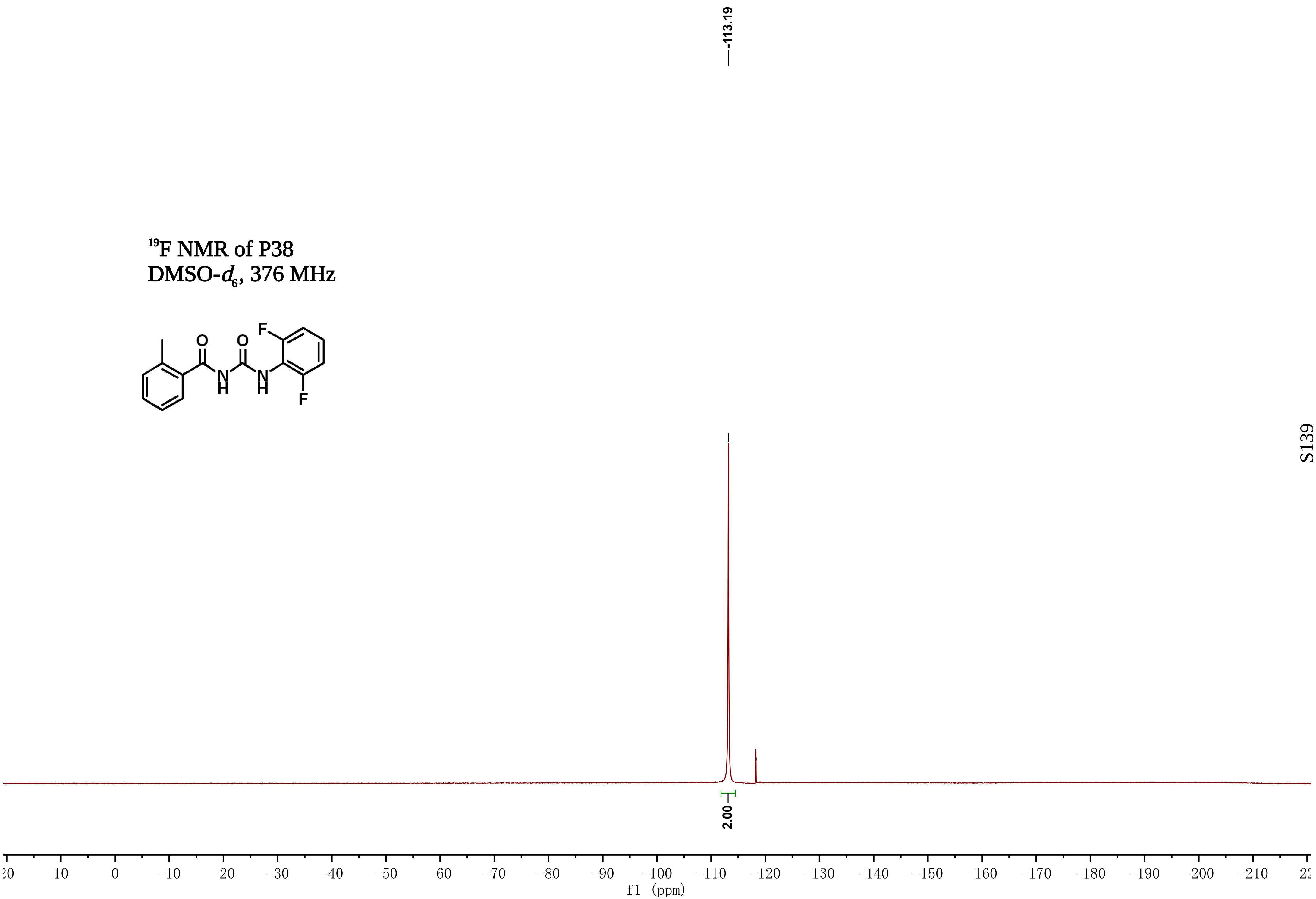

19F NMR of P38
DMSO-d6, 376 MHz
-113.19
2.00
20
10
0
−10
−20
−30
−40
−50
−60
−70
−80
−90
−100
−110
−120
−130
−140
−150
−160
−170
−180
−190
−200
−210
f1 (ppm)

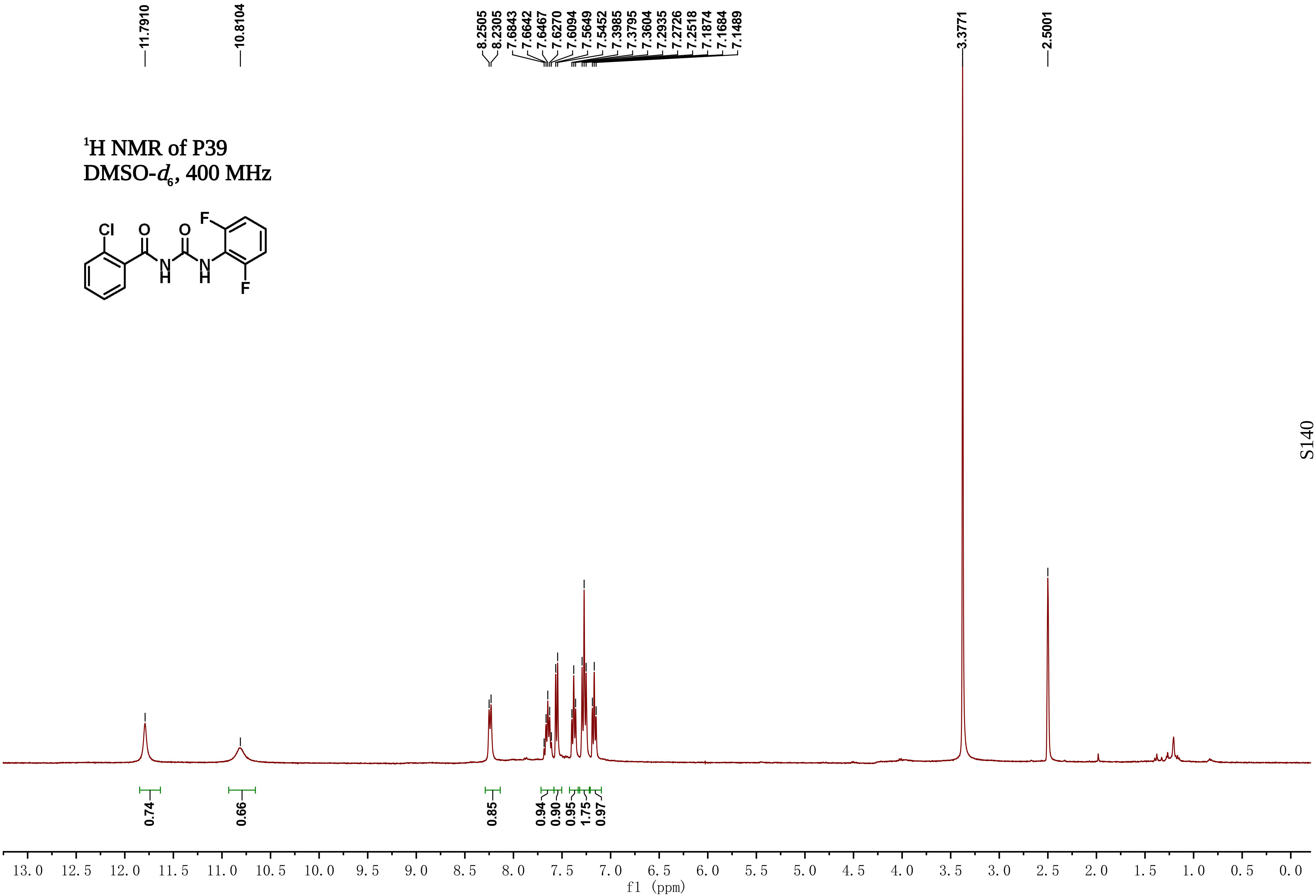

1H NMR of P39
DMSO-d6, 400 MHz
Cl
O
O
F
N
H
N
H
F
11.7910
10.8104
8.2505
8.2305
7.6843
7.6642
7.6467
7.6270
7.6094
7.5649
7.5452
7.3985
7.3795
7.3604
7.2935
7.2726
7.2518
7.1874
7.1684
7.1489
3.3771
2.5001
0.74
0.66
0.85
0.94
0.90
0.95
1.75
0.97
13.0 12.5 12.0 11.5 11.0 10.5 10.0 9.5 9.0 8.5 8.0 7.5 7.0 6.5 6.0 5.5 5.0 4.5 4.0 3.5 3.0 2.5 2.0 1.5 1.0 0.5 0.0
f1 (ppm)

^{13}C NMR of P39
DMSO-d_6, 101 MHz

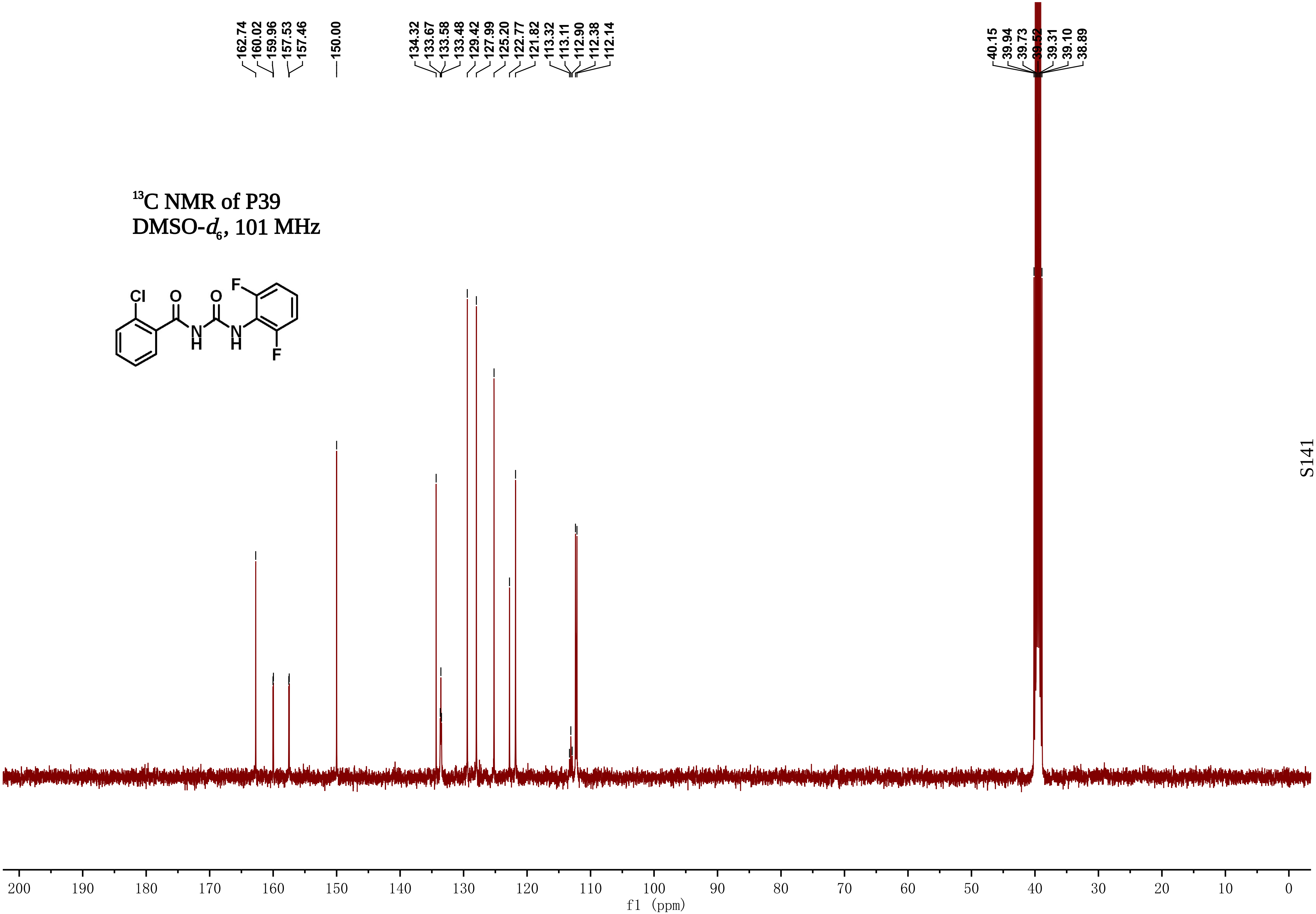

^{19}F NMR of P39
DMSO-d_6, 376 MHz

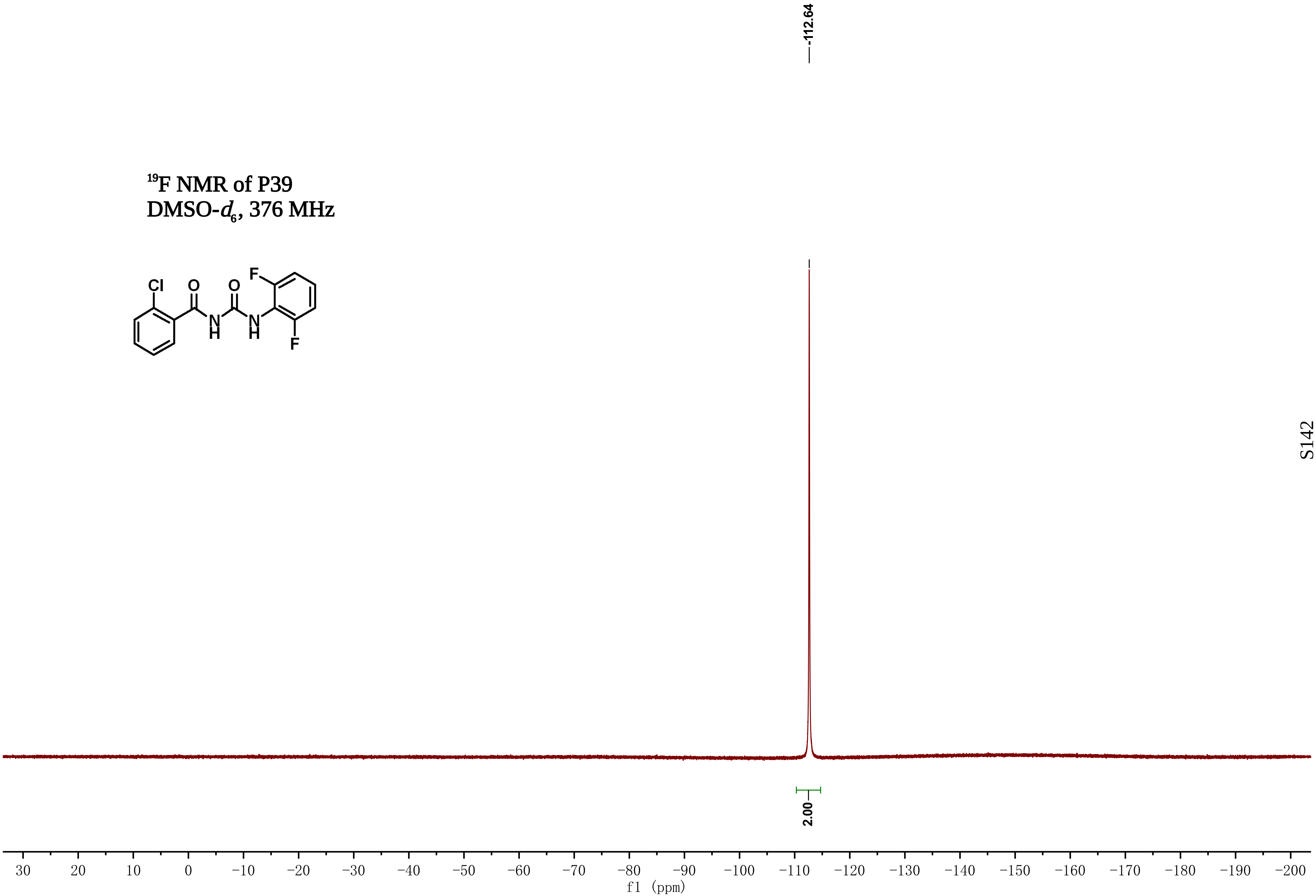

^{1}H NMR of P40
DMSO-d_6, 400 MHz

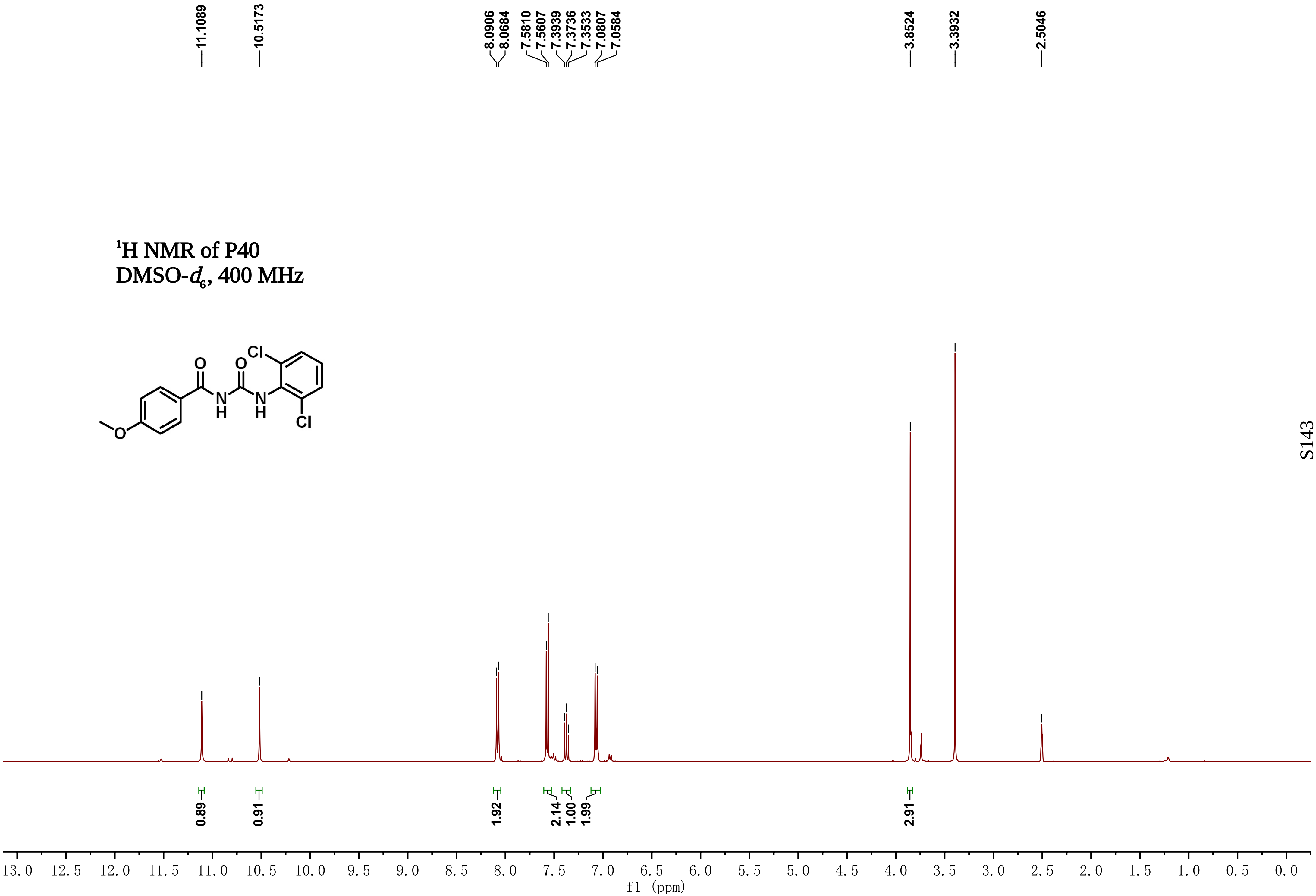

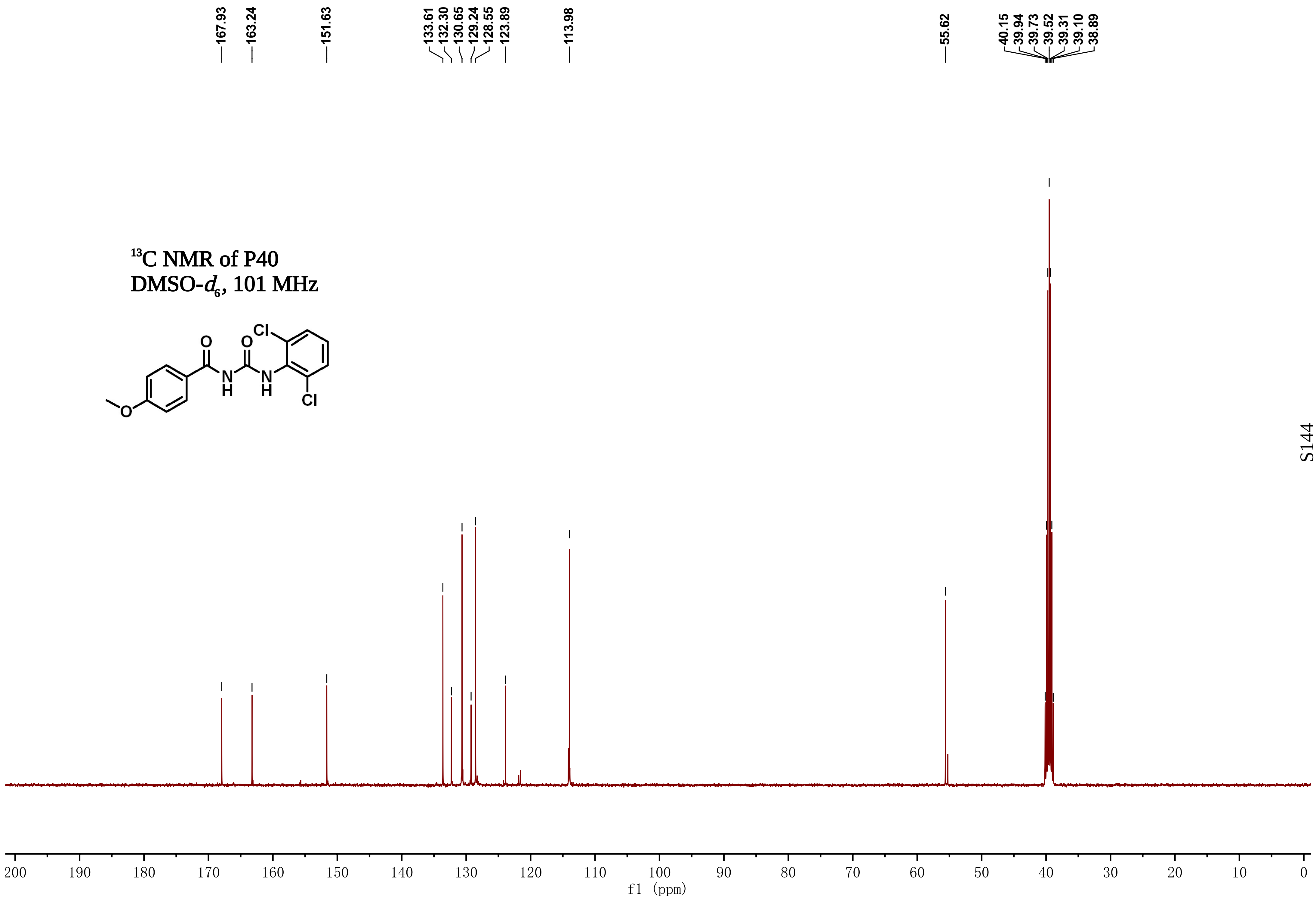
167.93
163.24
151.63
133.61
132.30
130.65
129.24
128.55
123.89
113.98
55.62
40.15
39.94
39.73
39.52
39.31
39.10
38.89
13C NMR of P40
DMSO-d6, 101 MHz
O
O
Cl
N
H
N
H
Cl
O
200 190 180 170 160 150 140 130 120 110 100 90 80 70 60 50 40 30 20 10 0
f1 (ppm)

^{1}H NMR of P41
DMSO-d_6, 400 MHz

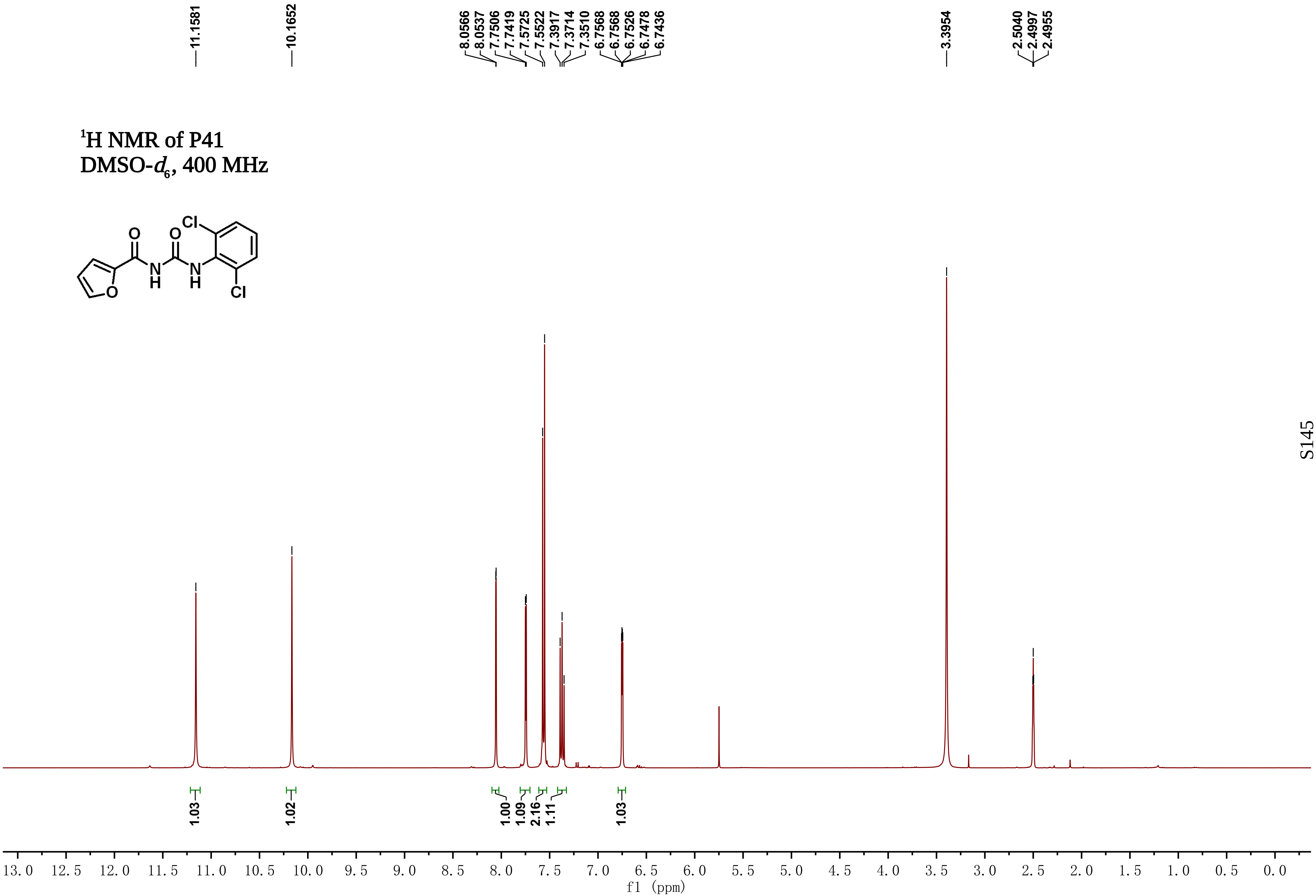

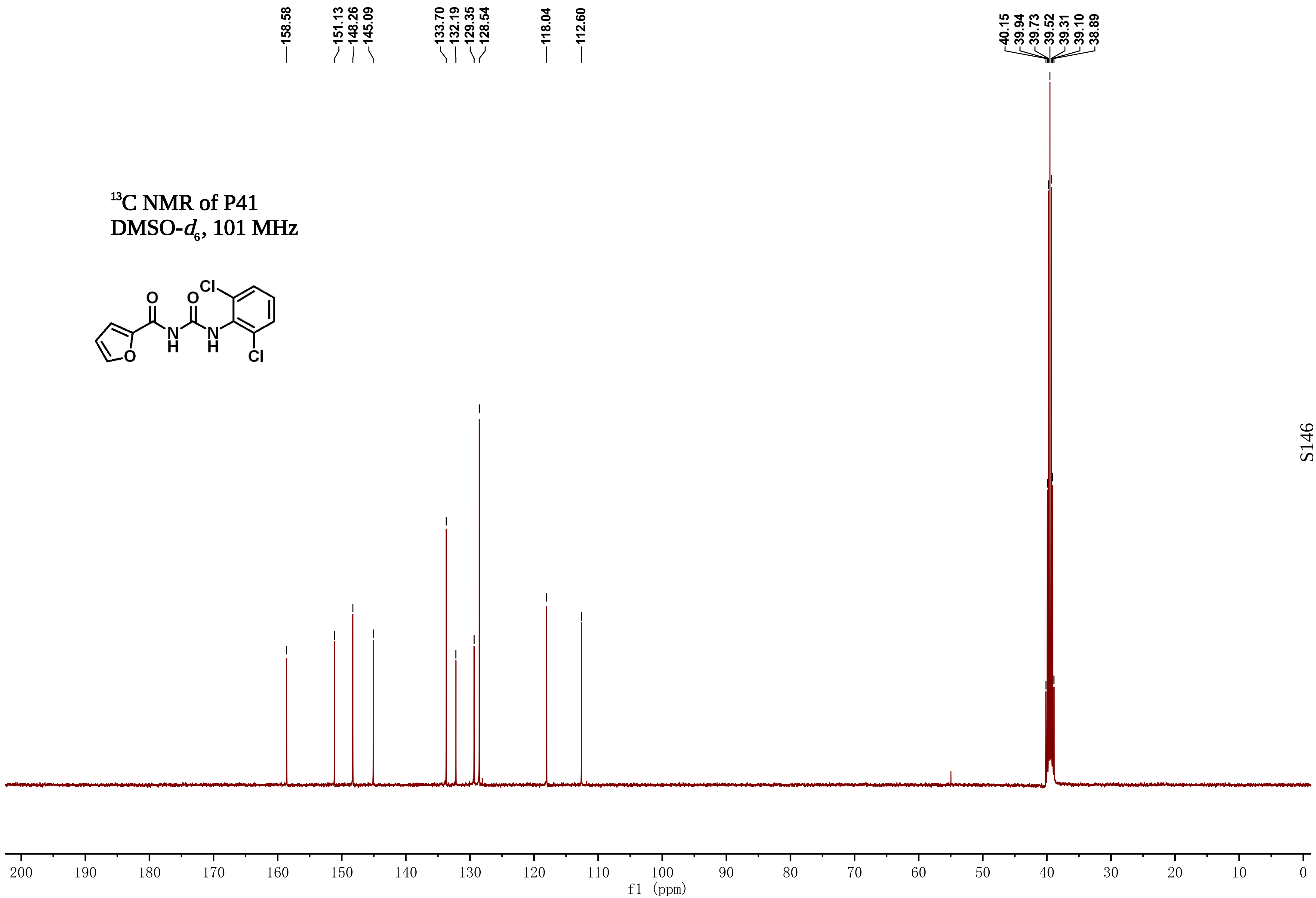

13C NMR of P41
DMSO-d6, 101 MHz
158.58
151.13
148.26
145.09
133.70
132.19
129.35
128.54
118.04
112.60
40.15
39.94
39.73
39.52
39.31
39.10
38.89
O
O
Cl
N
H
N
H
O
Cl
200 190 180 170 160 150 140 130 120 110 100 90 80 70 60 50 40 30 20 10 0
f1 (ppm)

^{1}H NMR of P42
$CDCl_3$, 400 MHz

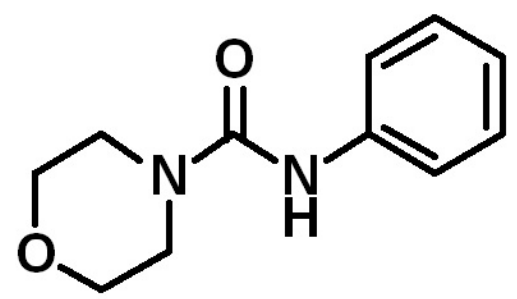

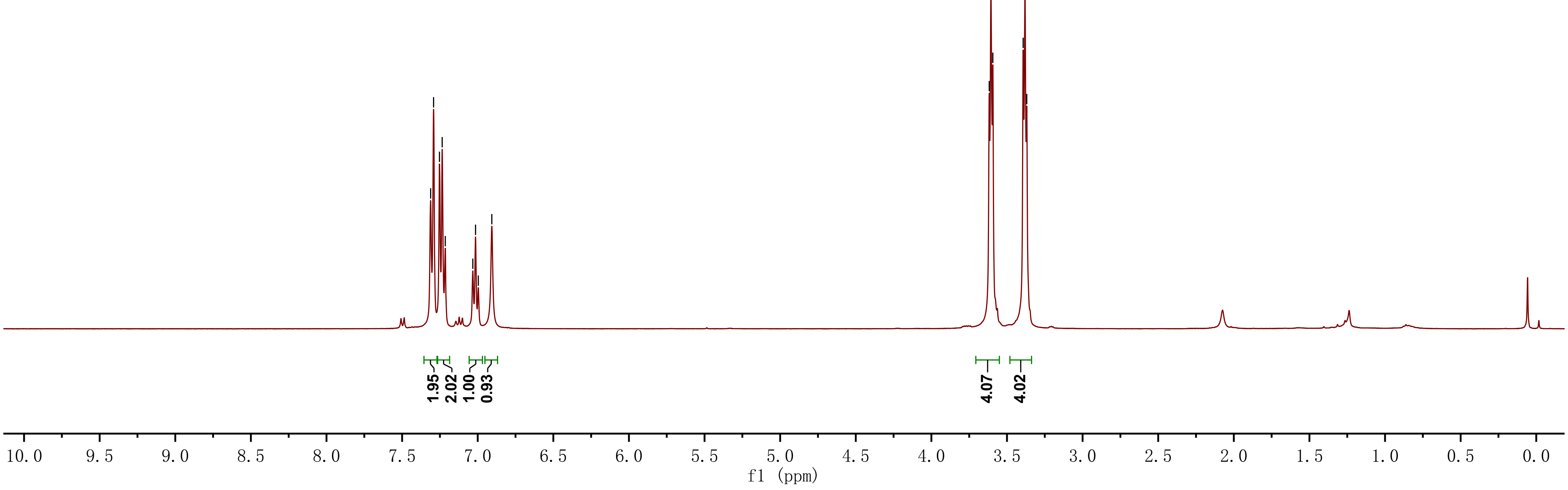

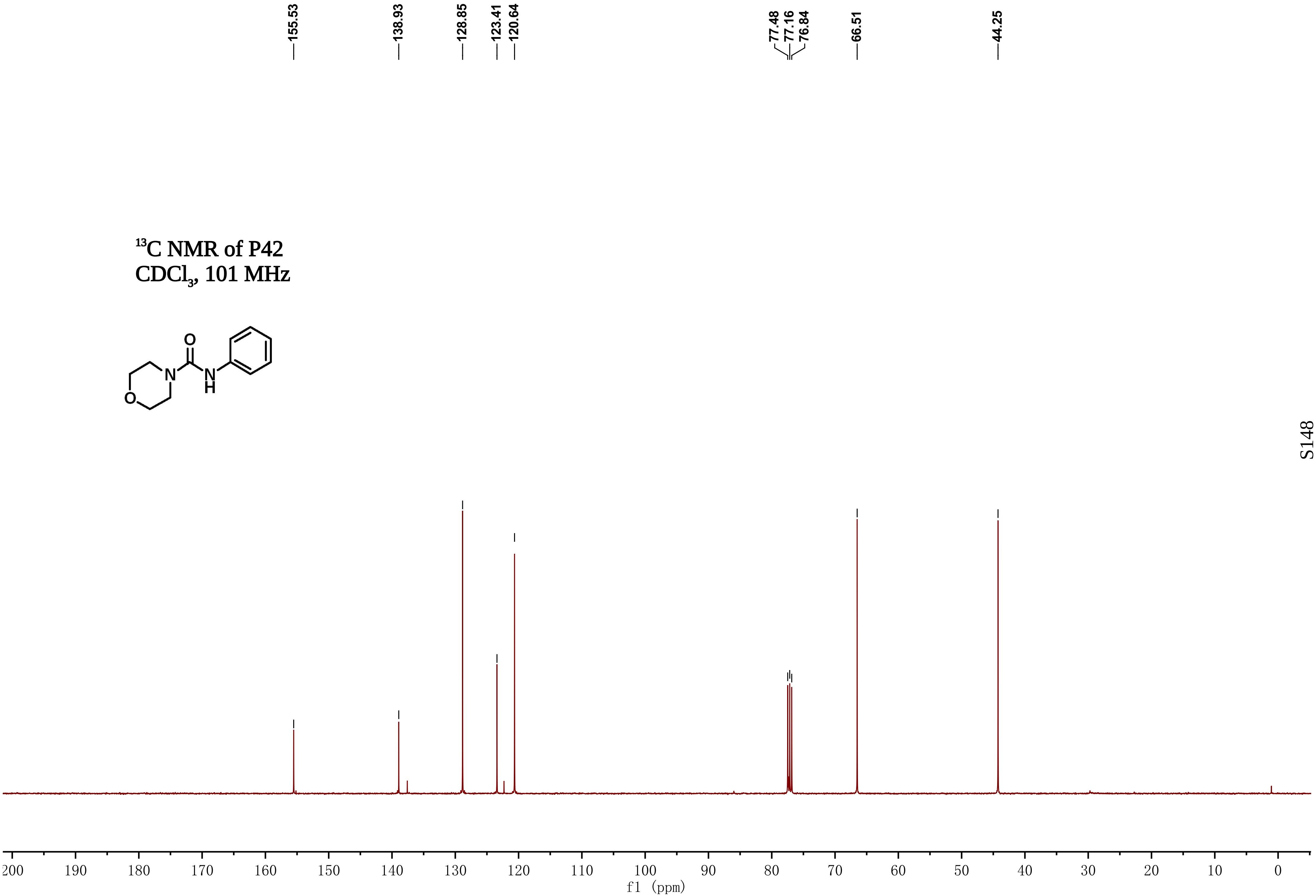

^{13}C NMR of P42
$CDCl_3$, 101 MHz

^{1}H NMR of P43
$CDCl_3$, 400 MHz

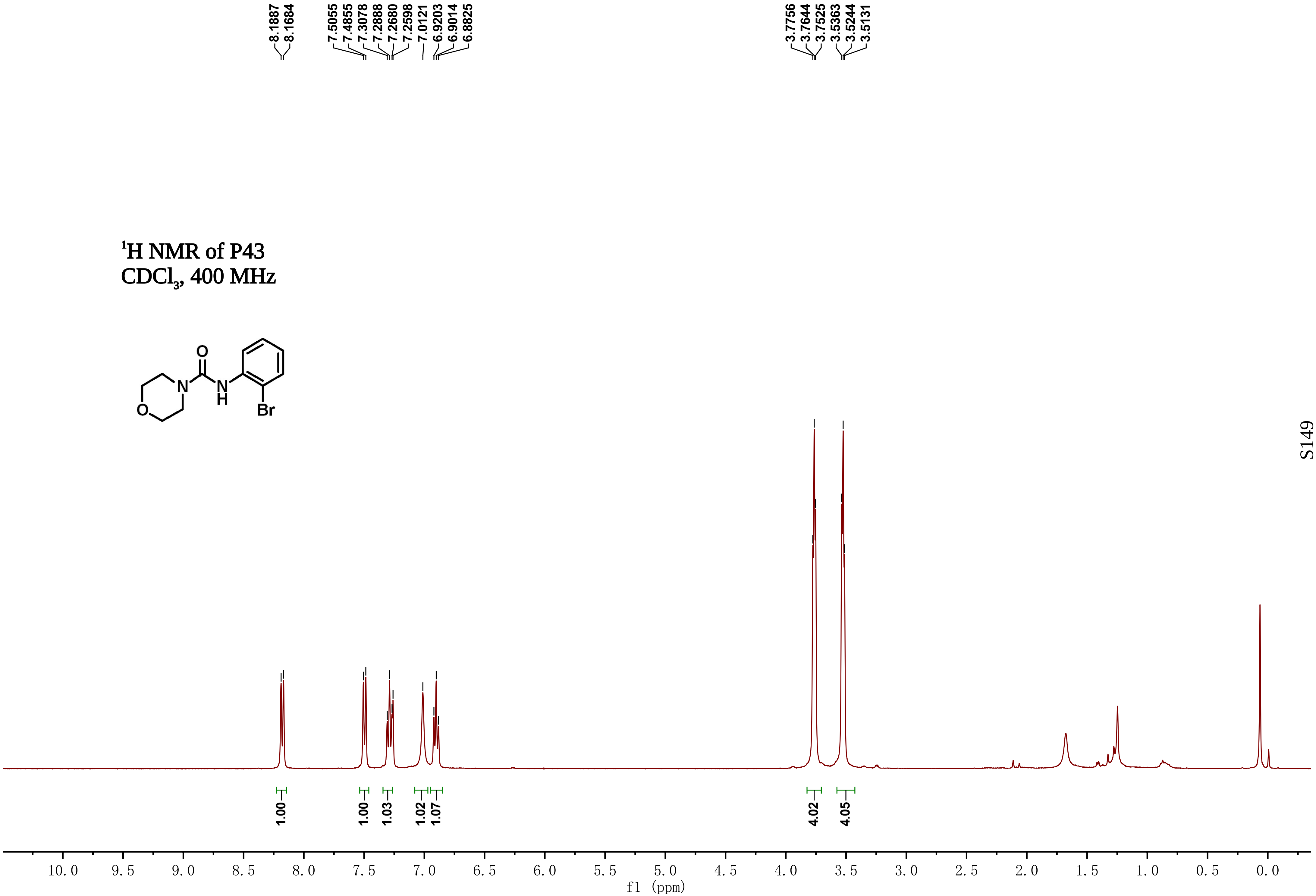

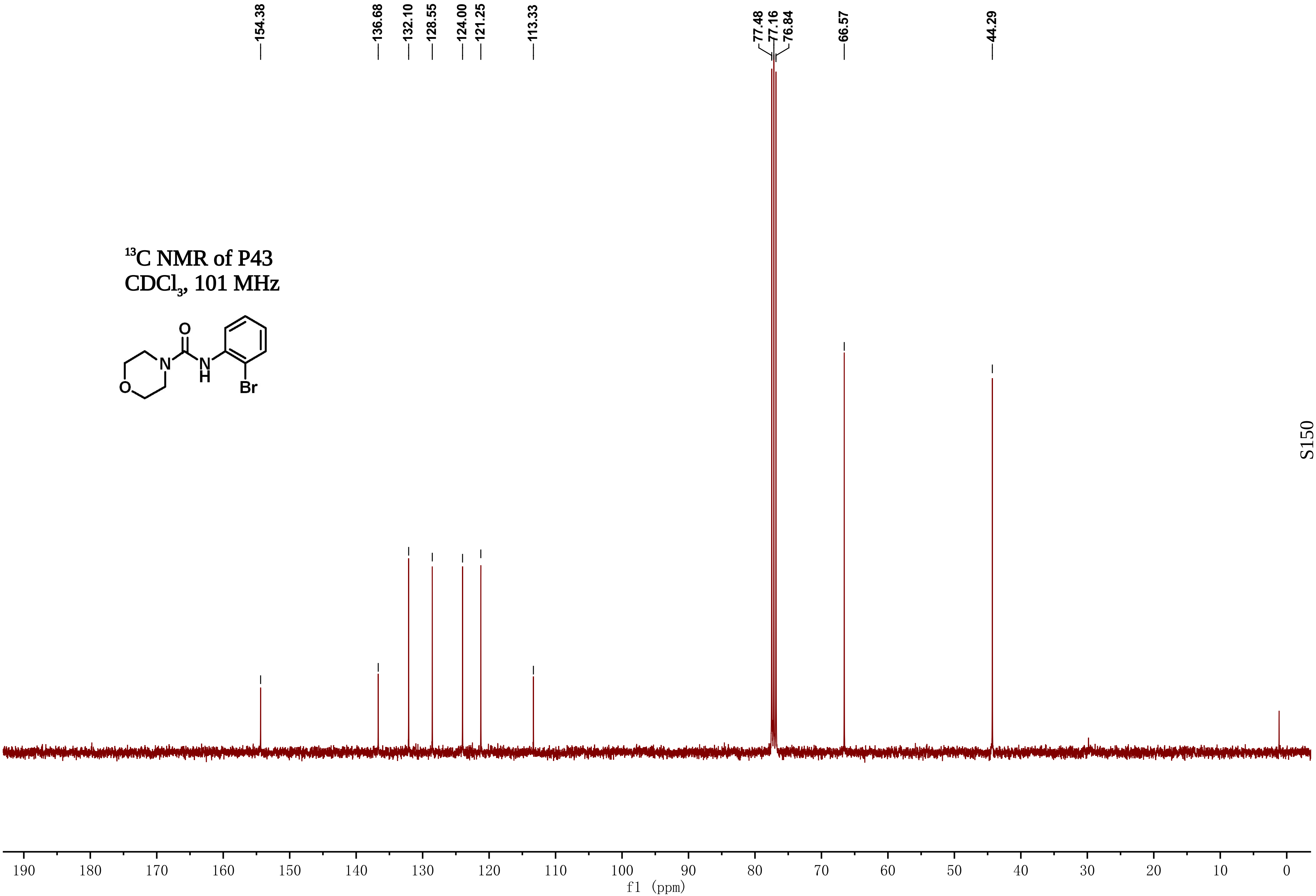
154.38
136.68
132.10
128.55
124.00
121.25
113.33
77.48
77.16
76.84
66.57
44.29
¹³C NMR of P43
CDCl₃, 101 MHz
O
N
N
H
Br
O
190
180
170
160
150
140
130
120
110
100
90
80
70
60
50
40
30
20
10
0
f1 (ppm)

^{1}H NMR of P44
$CDCl_3$, 400 MHz

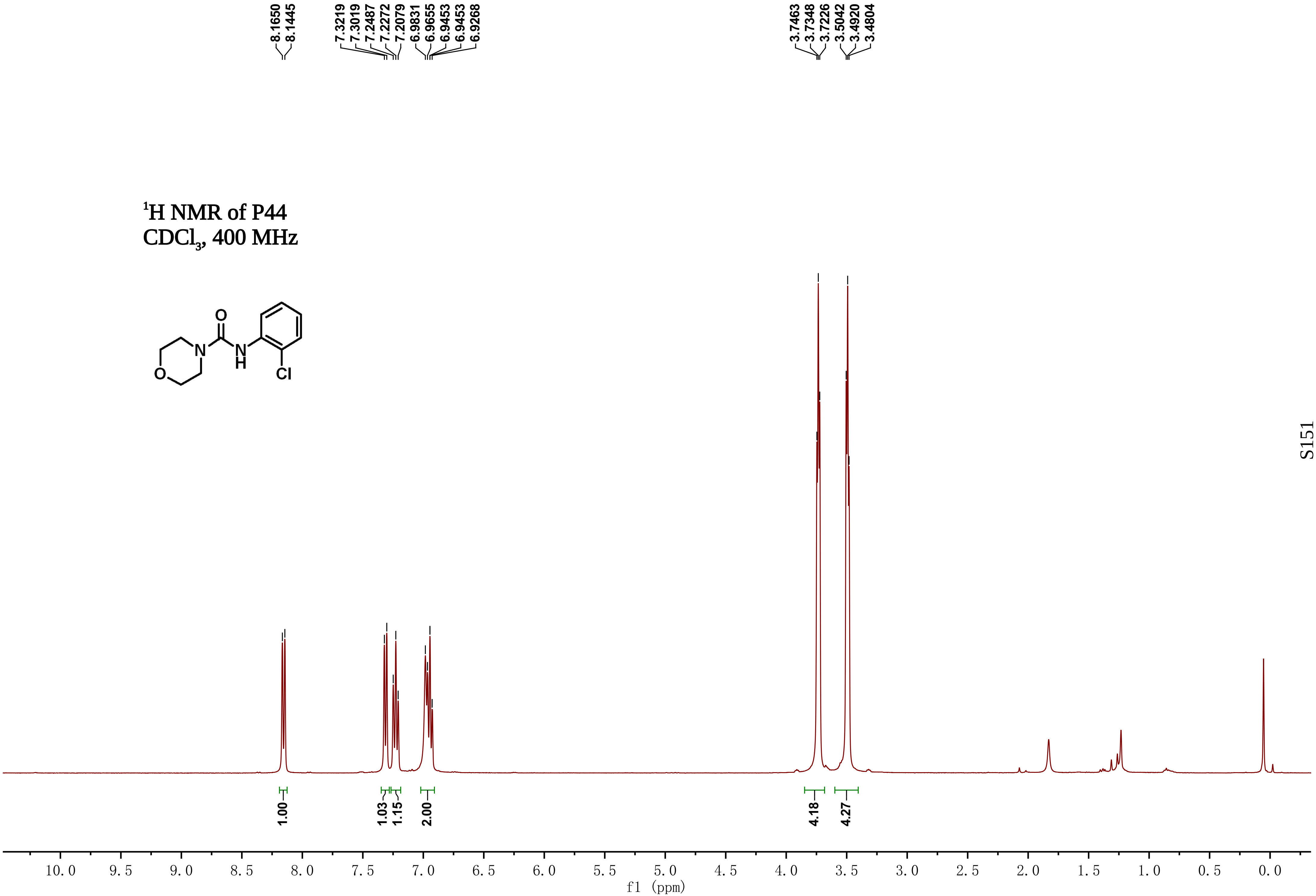

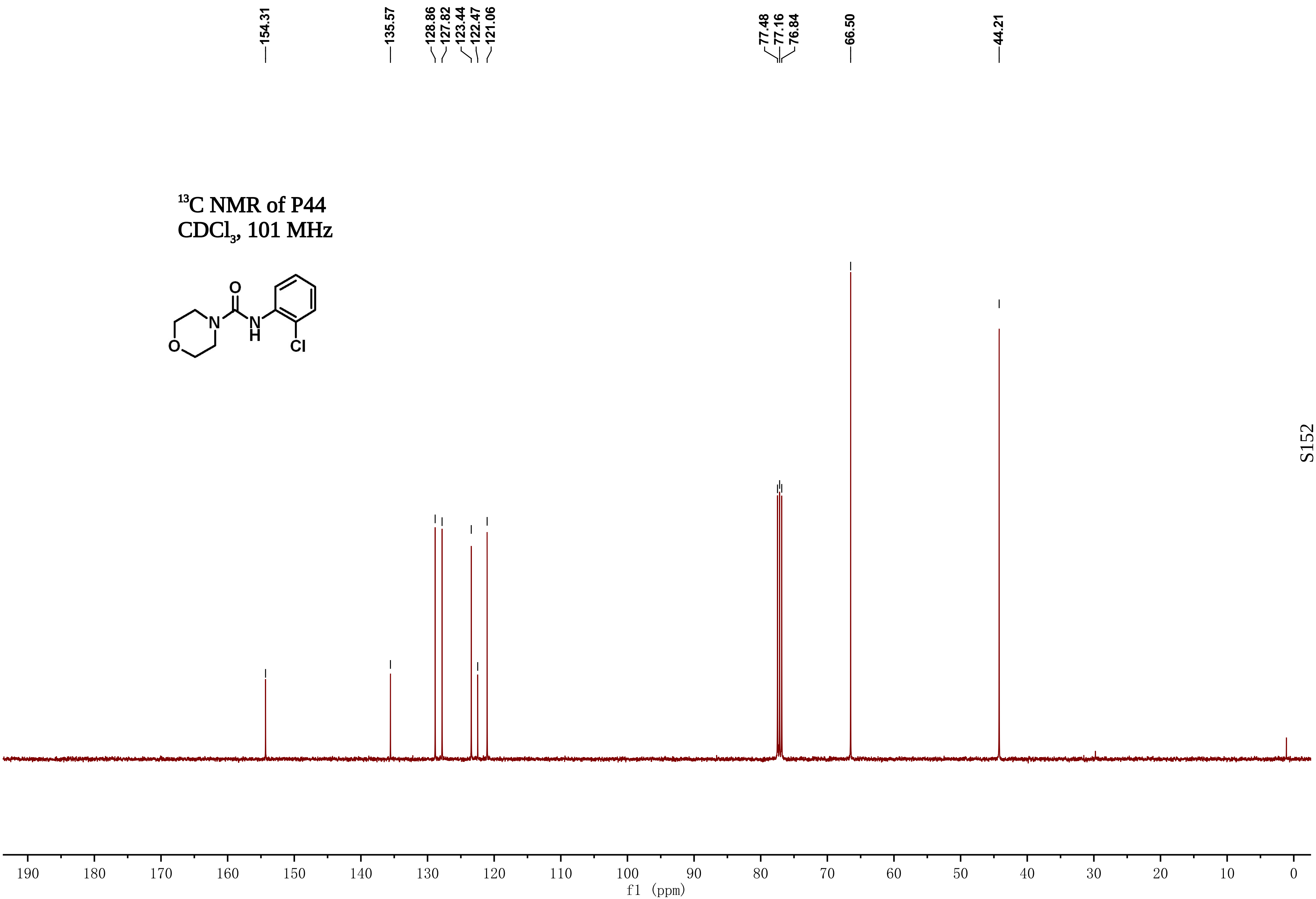
154.31
135.57
128.86
127.82
123.44
122.47
121.06
77.48
77.16
76.84
66.50
44.21
13C NMR of P44
CDCl3, 101 MHz
O
N
N
H
Cl
O
190
180
170
160
150
140
130
120
110
100
90
80
70
60
50
40
30
20
10
0
f1 (ppm)

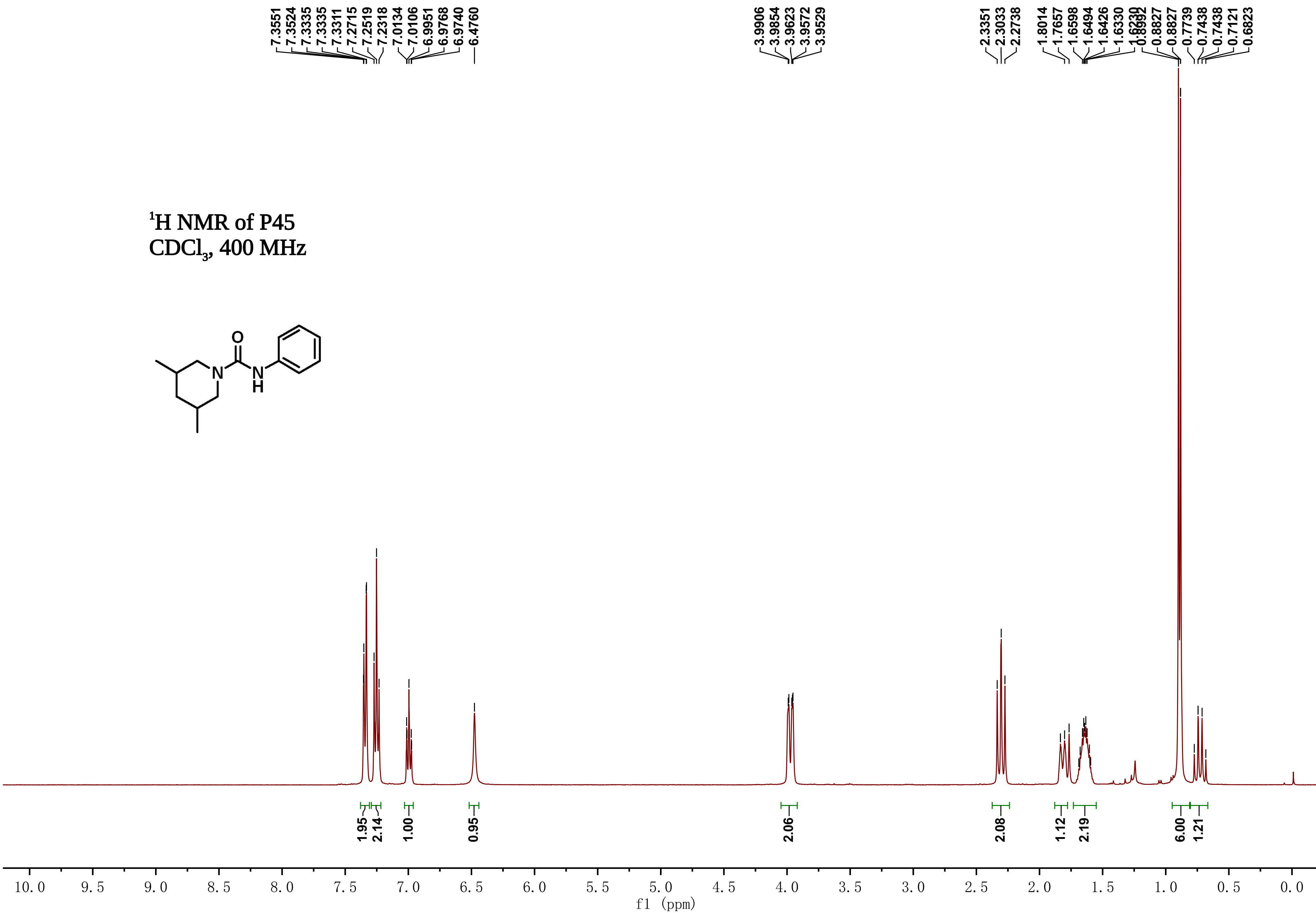

1H NMR of P45
CDCl3, 400 MHz
7.3551
7.3524
7.3335
7.3335
7.3311
7.2715
7.2519
7.2318
7.0134
7.0106
6.9951
6.9768
6.9740
6.4760
3.9906
3.9854
3.9623
3.9572
3.9529
2.3351
2.3033
2.2738
1.8014
1.7657
1.6598
1.6494
1.6426
1.6330
0.8827
0.8827
0.7739
0.7438
0.7438
0.7121
0.6823
1.95
2.14
1.00
0.95
2.06
2.08
1.12
2.19
6.00
1.21
10.0
9.5
9.0
8.5
8.0
7.5
7.0
6.5
6.0
5.5
5.0
4.5
4.0
3.5
3.0
2.5
2.0
1.5
1.0
0.5
0.0
f1 (ppm)

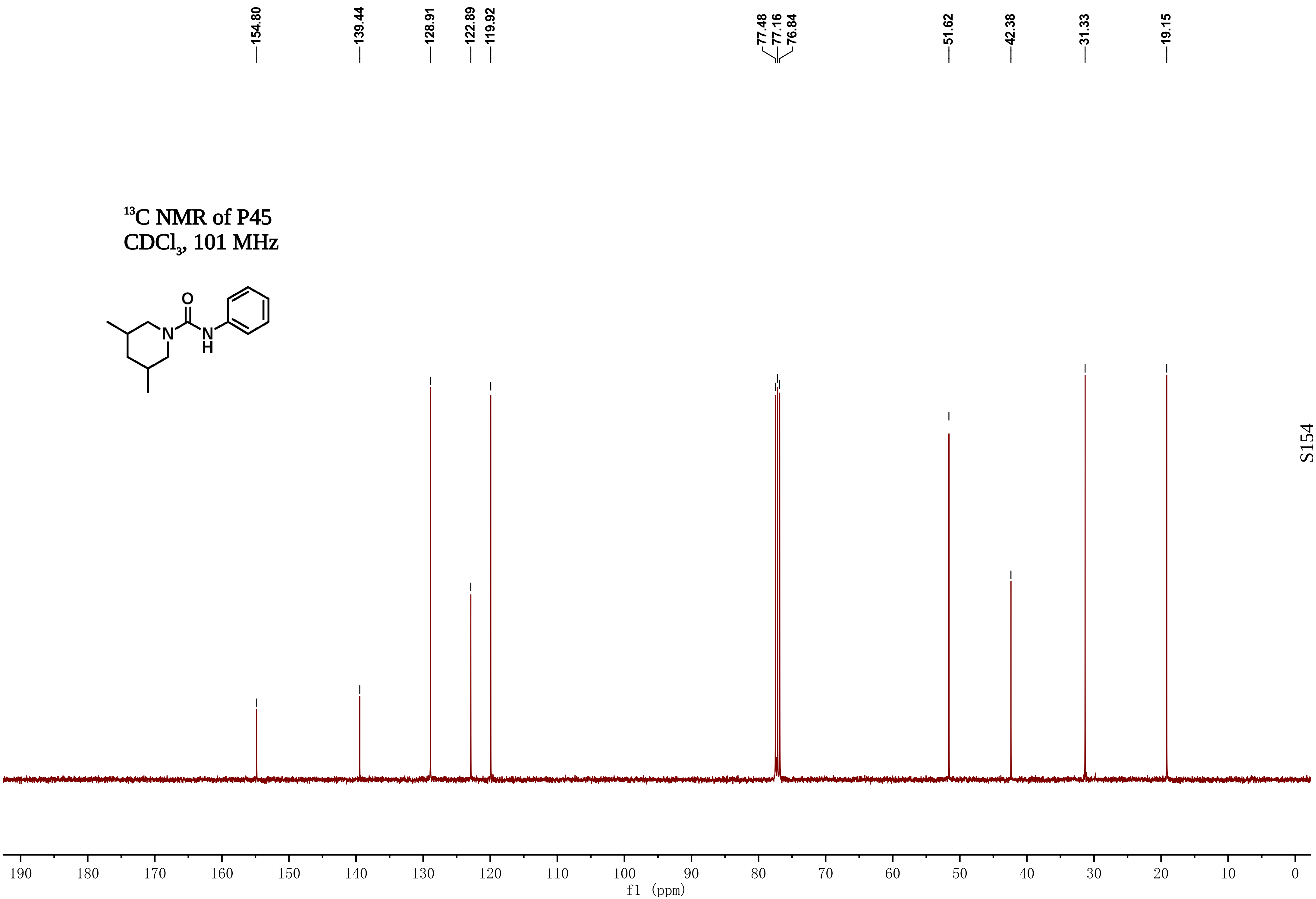
154.80
139.44
128.91
122.89
119.92
77.48
77.16
76.84
51.62
42.38
31.33
19.15
^{13}C NMR of P45
$CDCl_3$, 101 MHz
O
N
N
H
190
180
170
160
150
140
130
120
110
100
90
80
70
60
50
40
30
20
10
0
f1 (ppm)

^{1}H NMR of P46
$CDCl_3$, 400 MHz

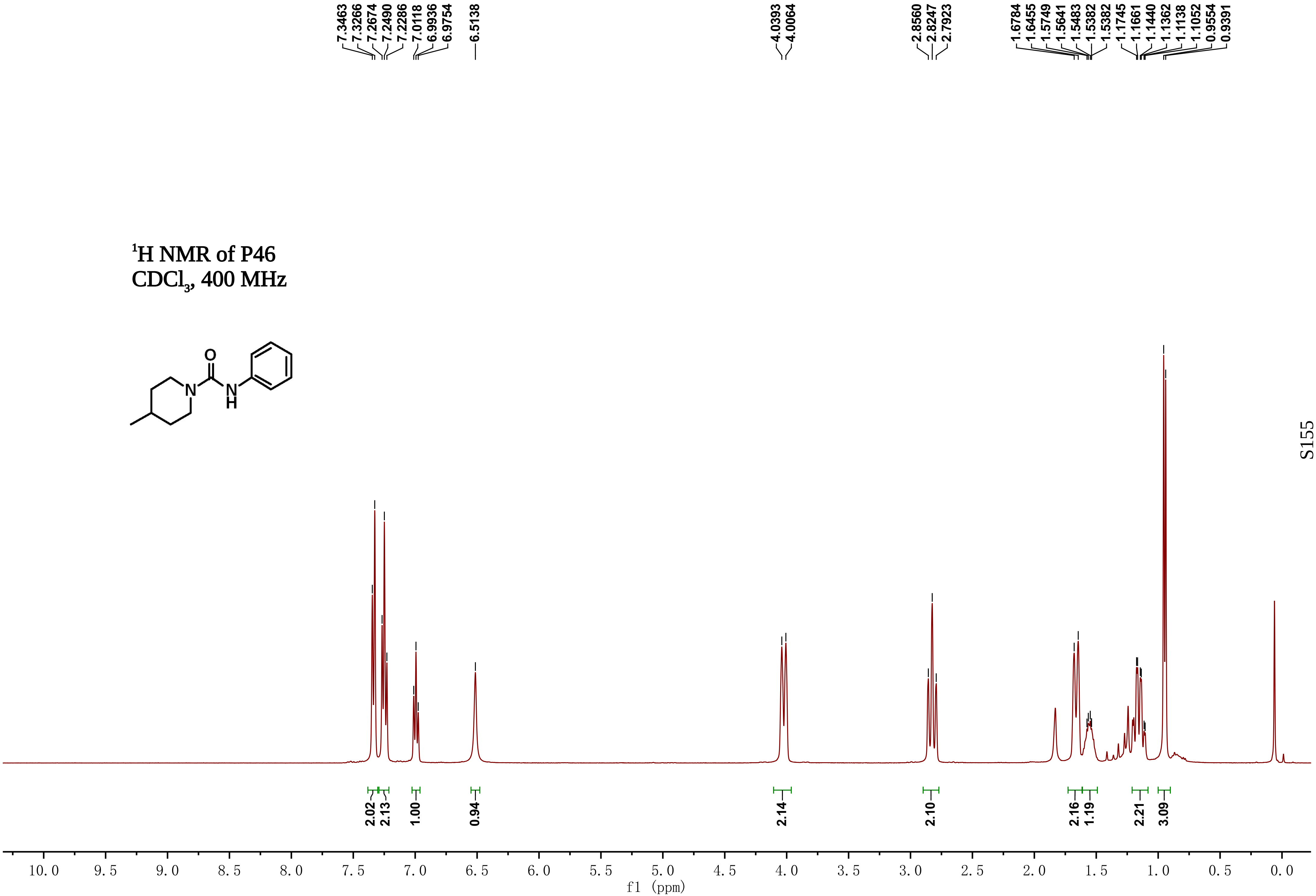

^{13}C NMR of P46
$CDCl_3$, 101 MHz

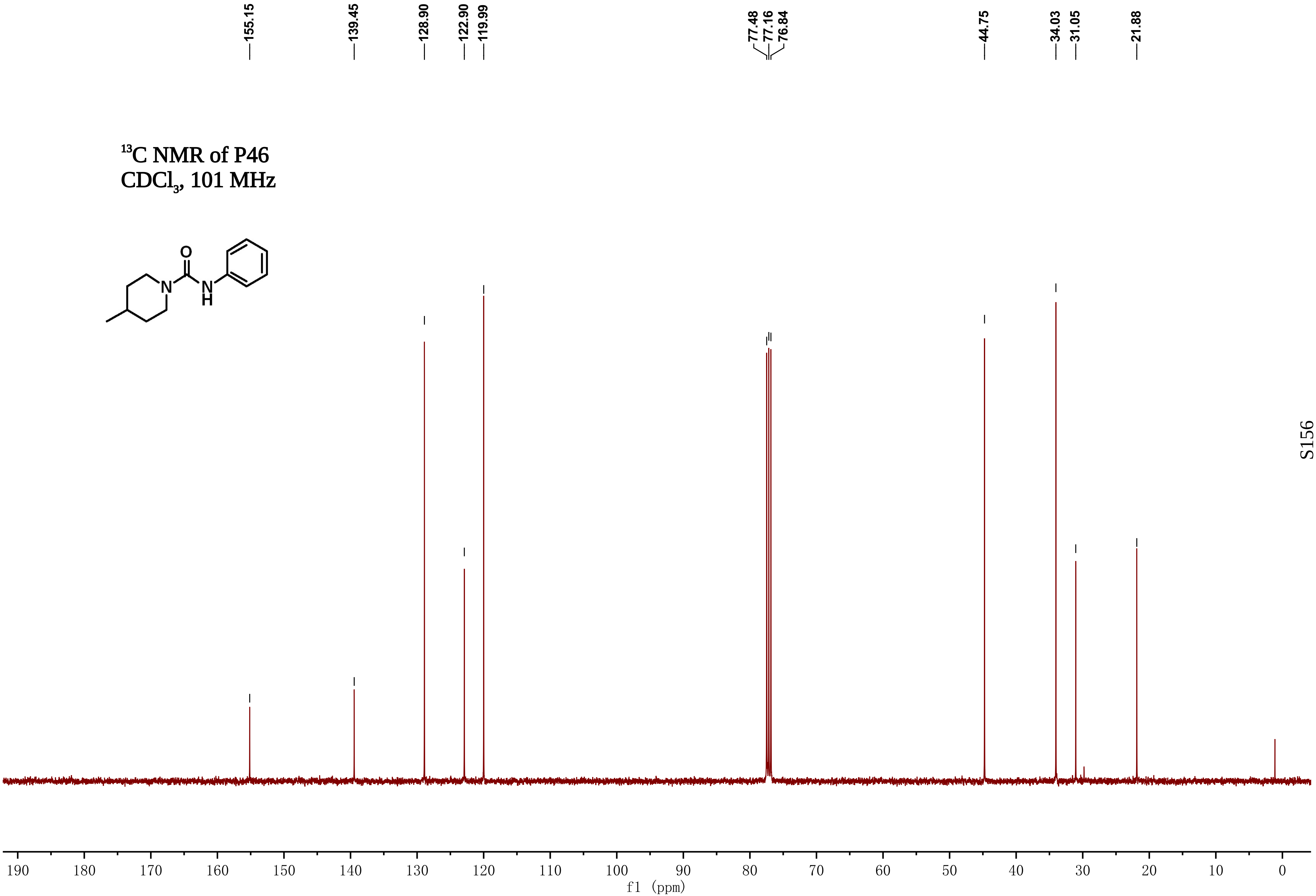

^{1}H NMR of P47
CDCl$_3$, 400 MHz

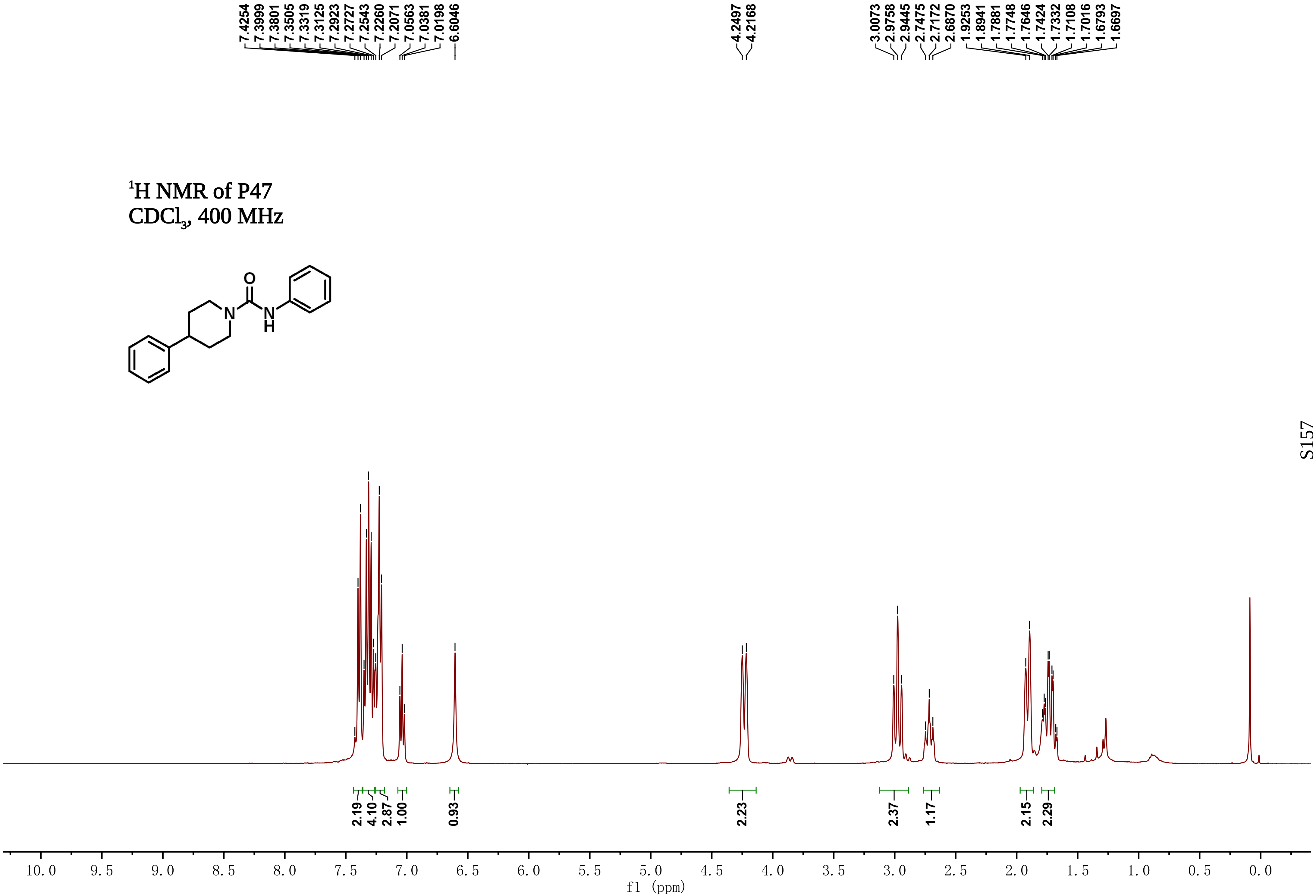

^{13}C NMR of P47
CDCl$_3$, 101 MHz

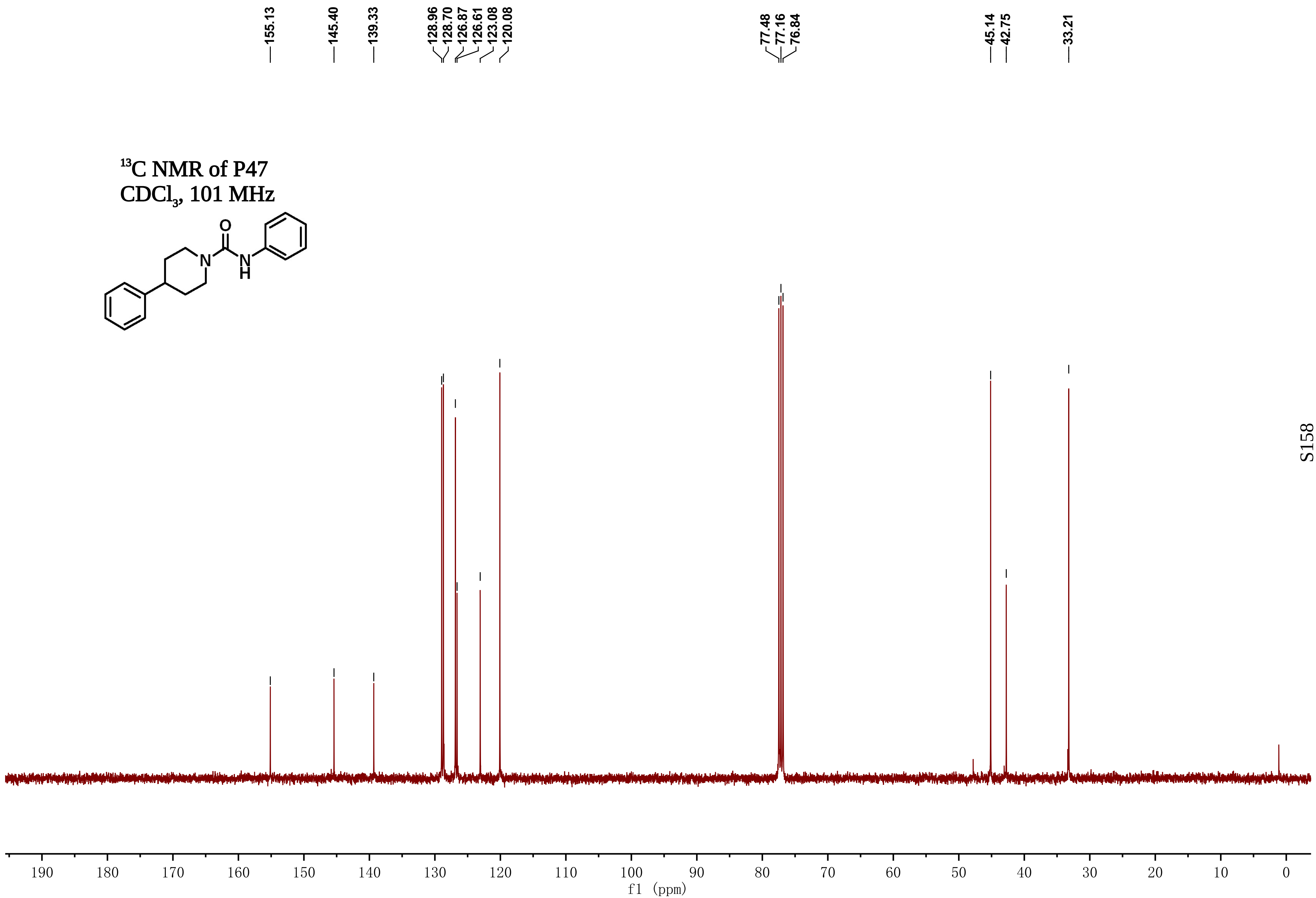

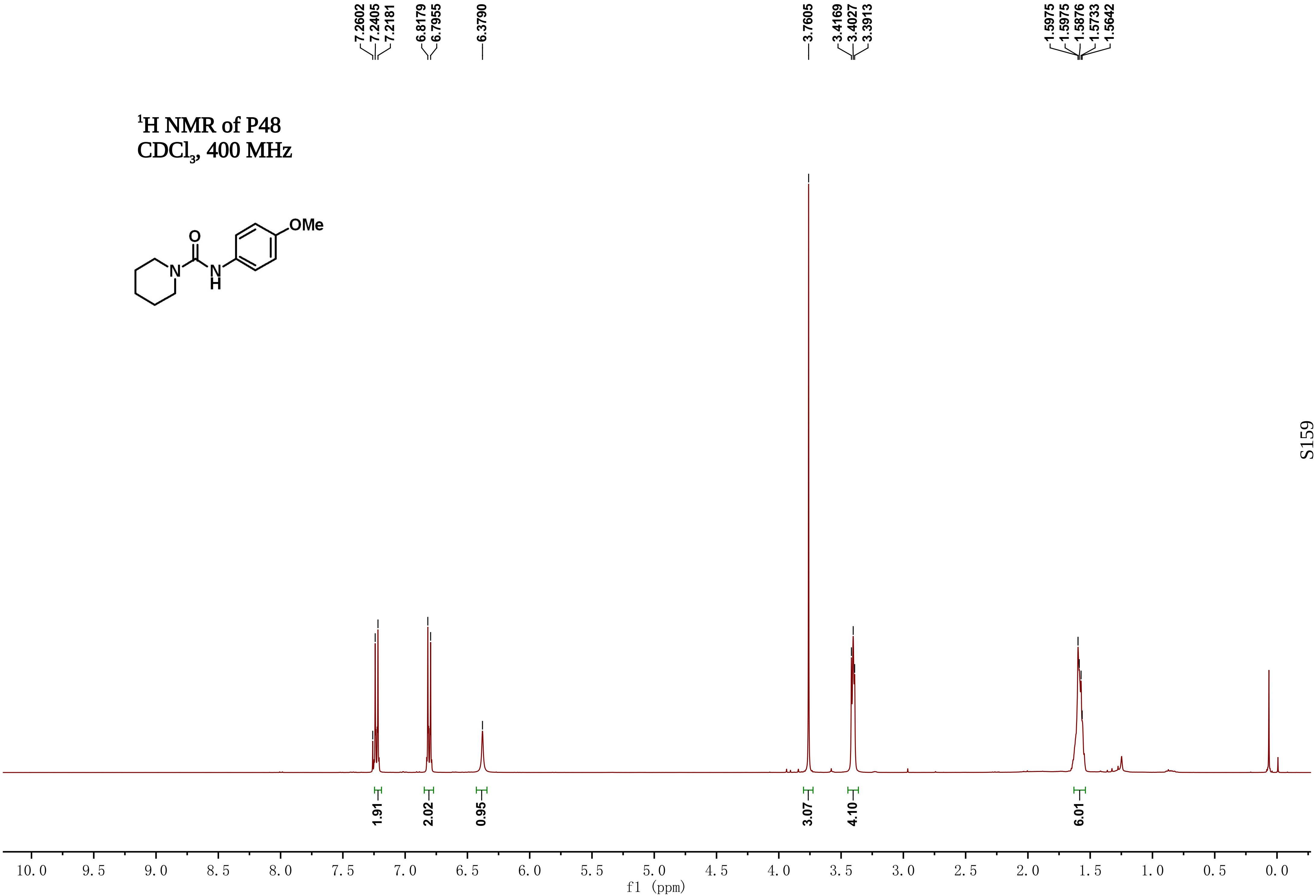
7.2602
7.2405
7.2181
6.8179
6.7955
6.3790
3.7605
3.4169
3.4027
3.3913
1.5975
1.5975
1.5876
1.5733
1.5642
^{1}H NMR of P48
$CDCl_3$, 400 MHz
OMe
O
N
N
H
1.91
2.02
0.95
3.07
4.10
6.01
10.0 9.5 9.0 8.5 8.0 7.5 7.0 6.5 6.0 5.5 5.0 4.5 4.0 3.5 3.0 2.5 2.0 1.5 1.0 0.5 0.0
f1 (ppm)

^{13}C NMR of P48
$CDCl_3$, 101 MHz

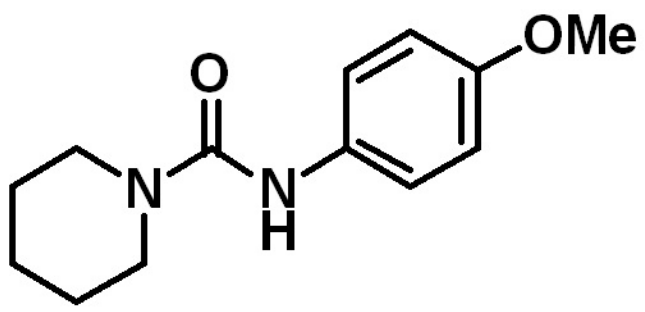

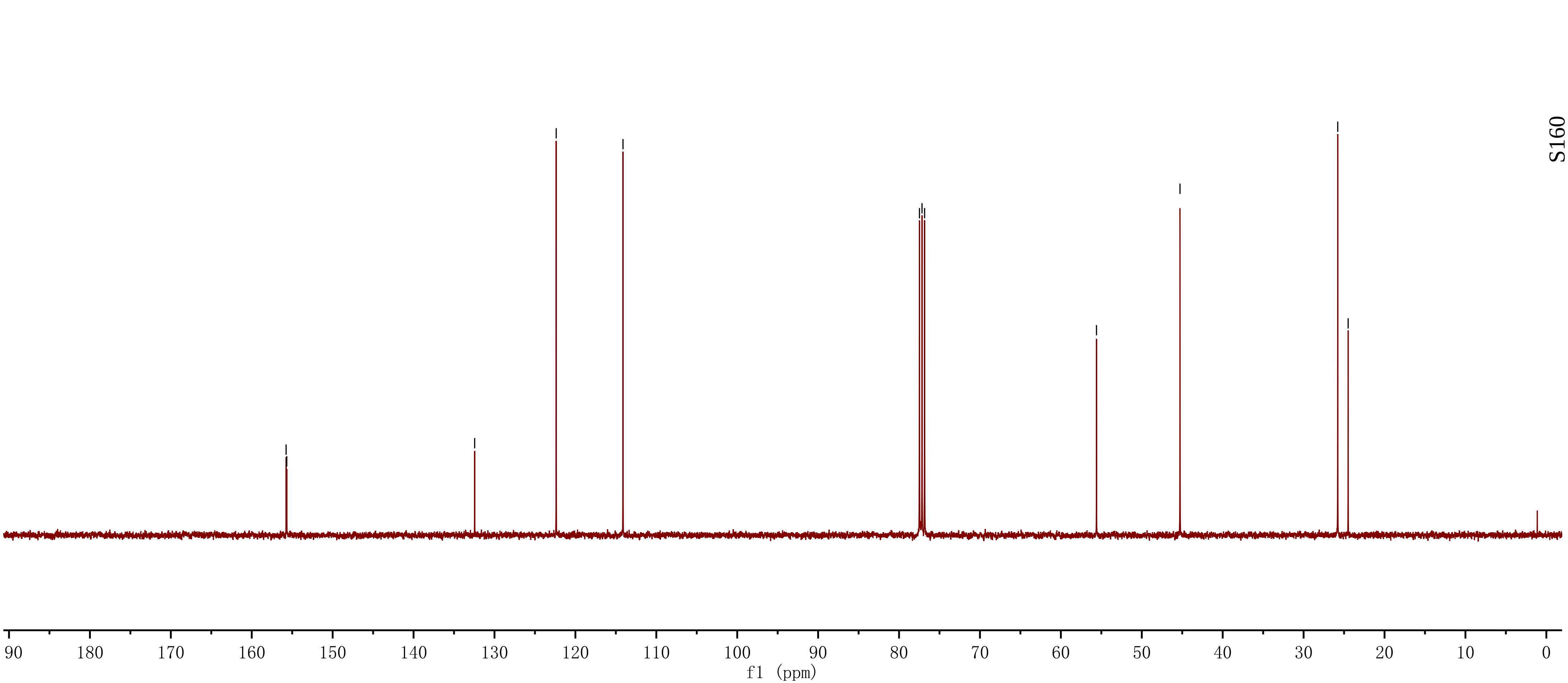

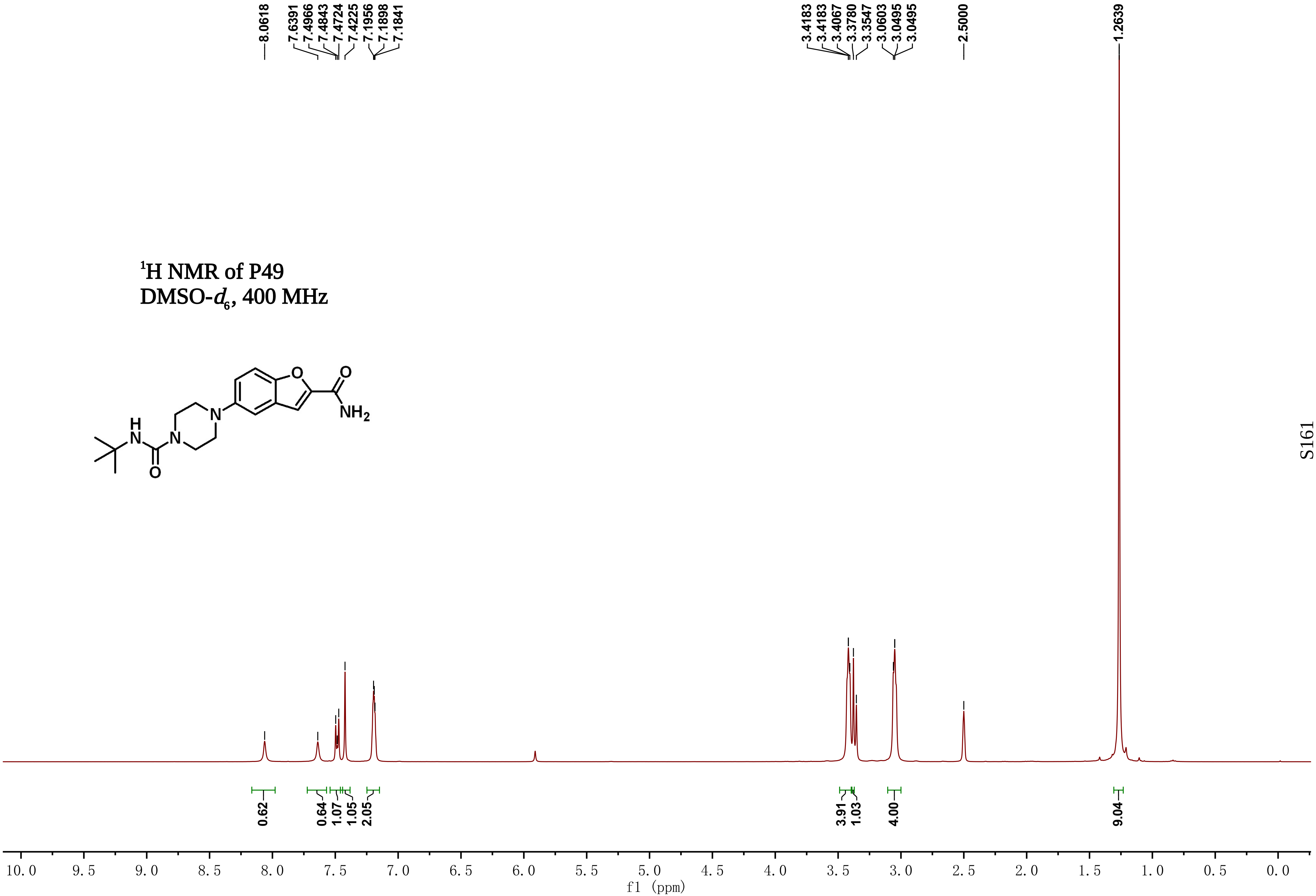
1H NMR of P49
DMSO-d6, 400 MHz
8.0618
7.6391
7.4966
7.4843
7.4724
7.4225
7.1956
7.1898
7.1841
3.4183
3.4183
3.4067
3.3780
3.3547
3.0603
3.0495
3.0495
2.5000
1.2639
0.62
0.64
1.07
1.05
2.05
3.91
1.03
4.00
9.04
10.0
9.5
9.0
8.5
8.0
7.5
7.0
6.5
6.0
5.5
5.0
4.5
4.0
3.5
3.0
2.5
2.0
1.5
1.0
0.5
0.0
f1 (ppm)
O
O
NH2
N
H
N
N
O

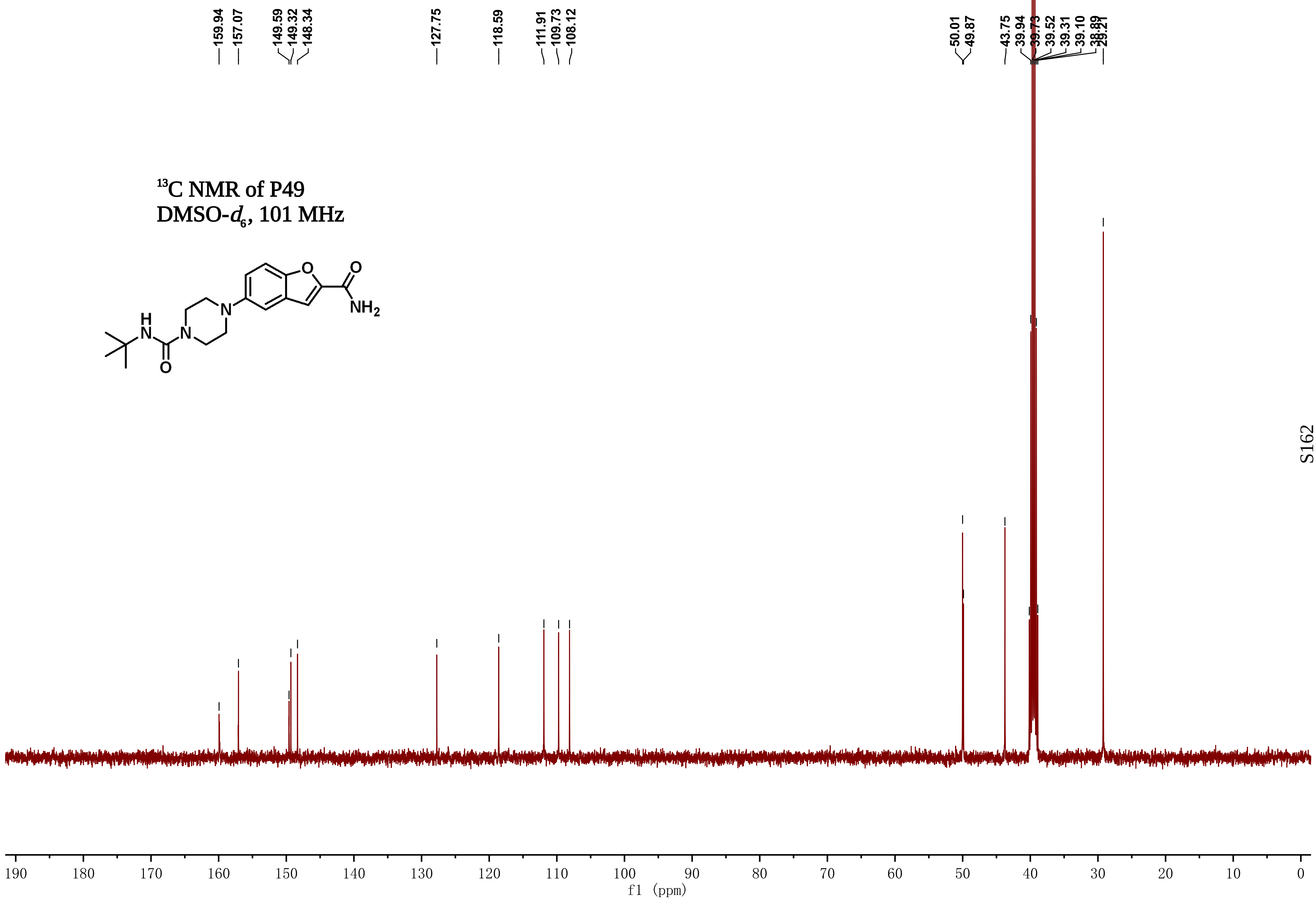

159.94
157.07
149.59
149.32
148.34
127.75
118.59
111.91
109.73
108.12
50.01
49.87
43.75
39.94
39.73
39.52
39.31
39.10
38.89
29.21
^{13}C NMR of P49
DMSO-d_6, 101 MHz
f1 (ppm)

^{1}H NMR of P50
$CDCl_3$, 400 MHz

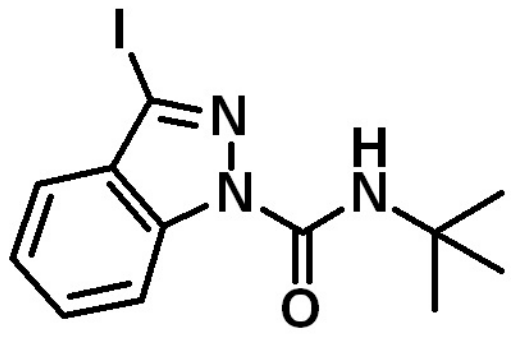

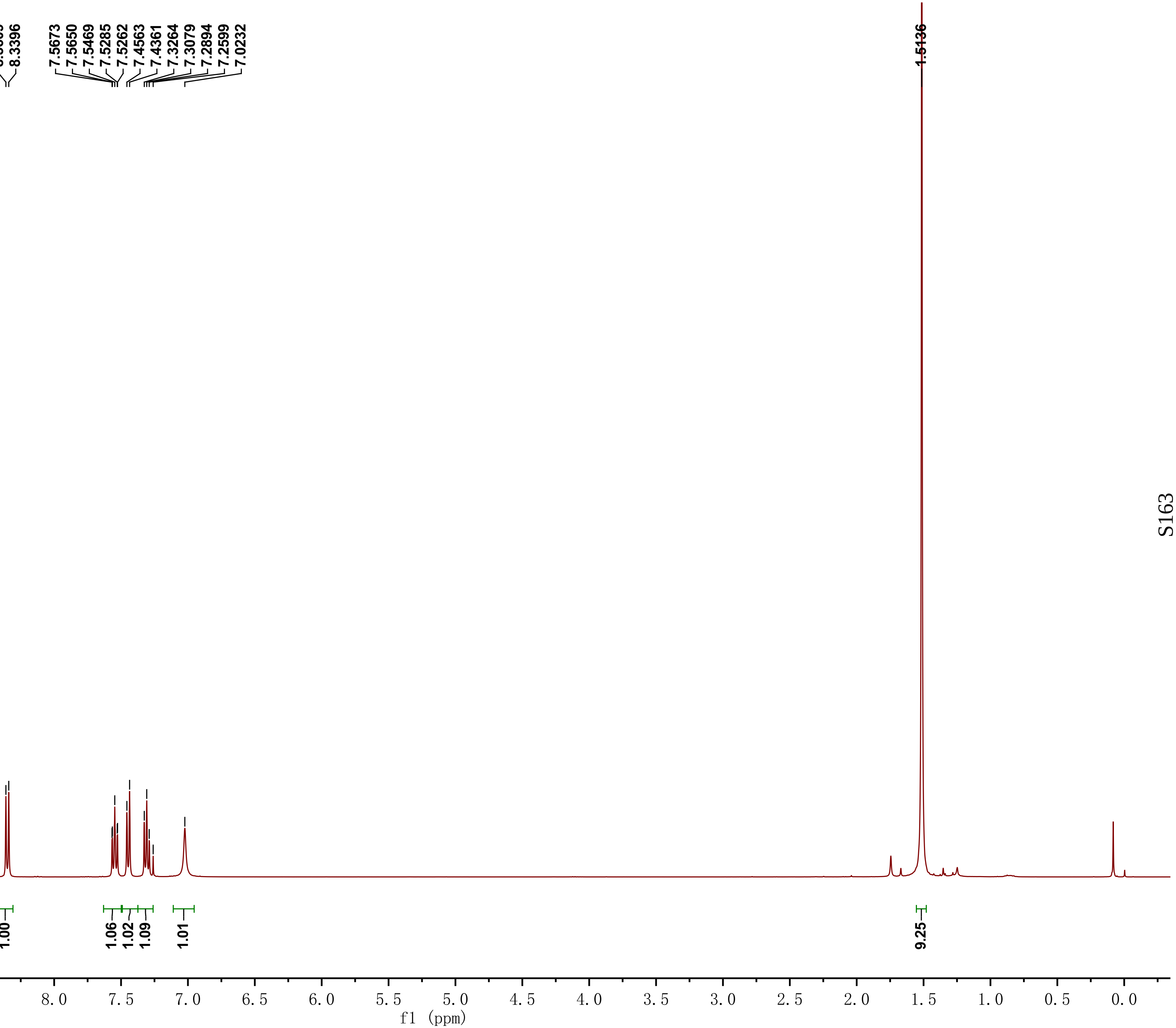

^{13}C NMR of P50
$CDCl_3$, 101 MHz

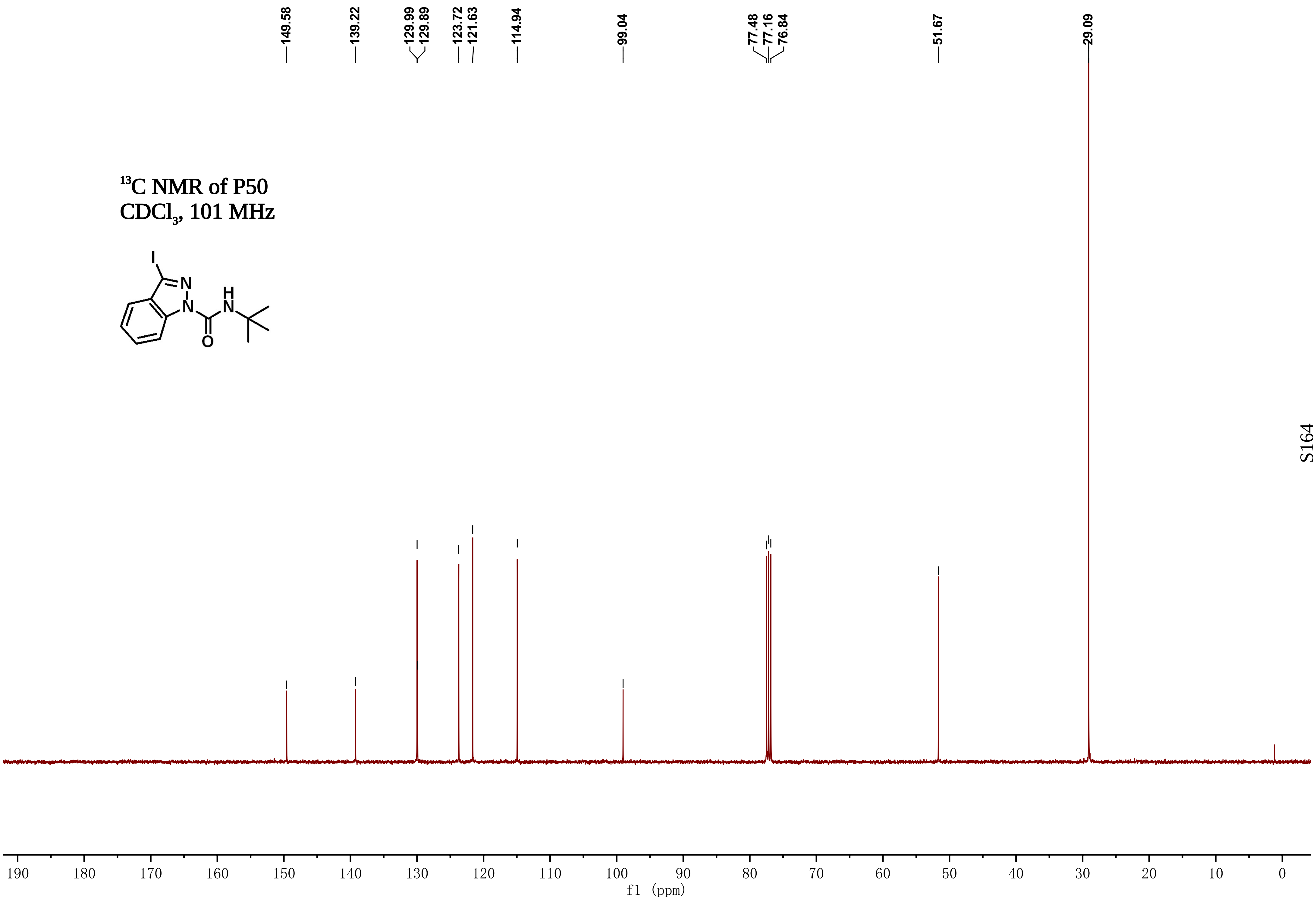

^{1}H NMR of P51
$CDCl_3$, 400 MHz

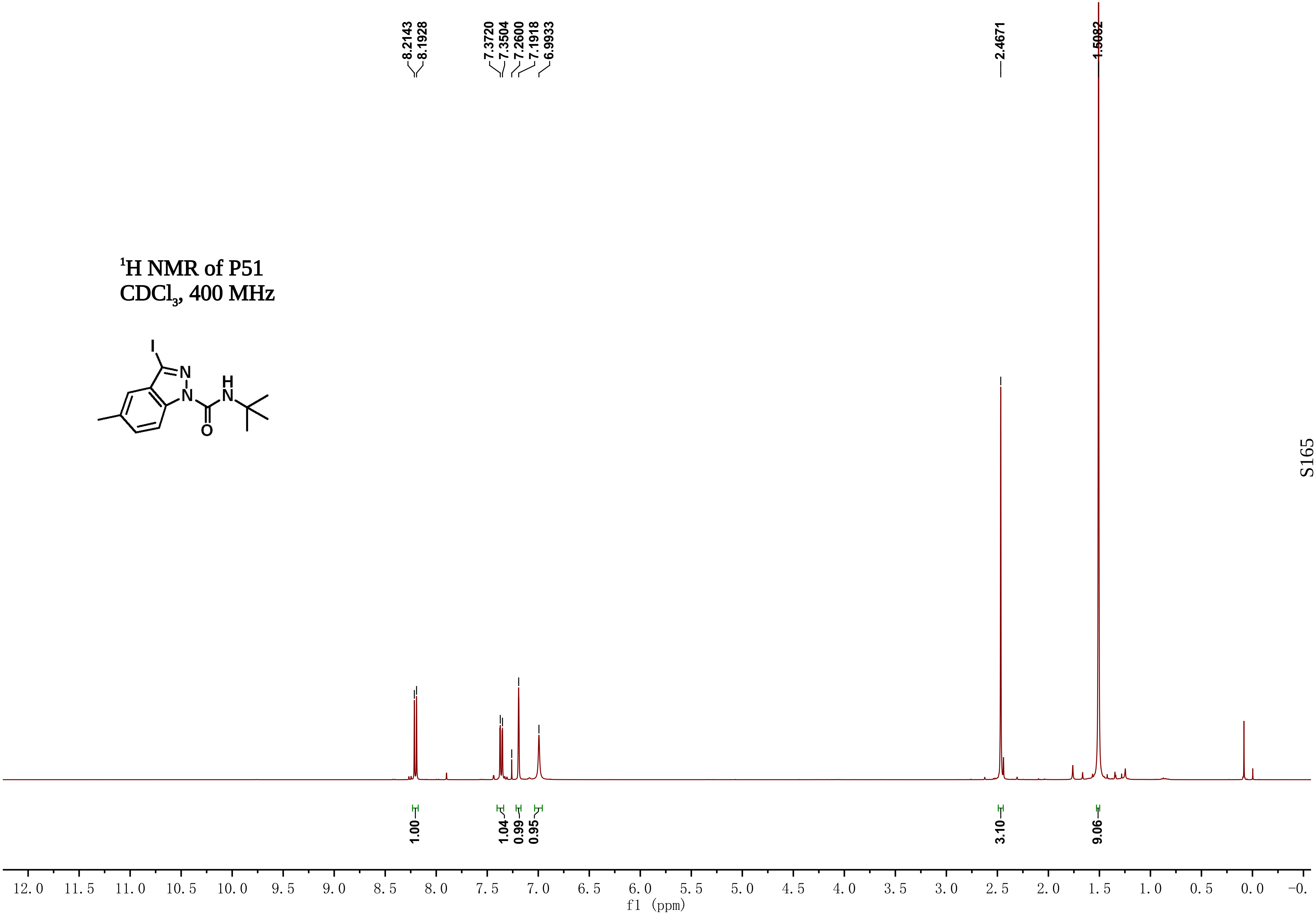

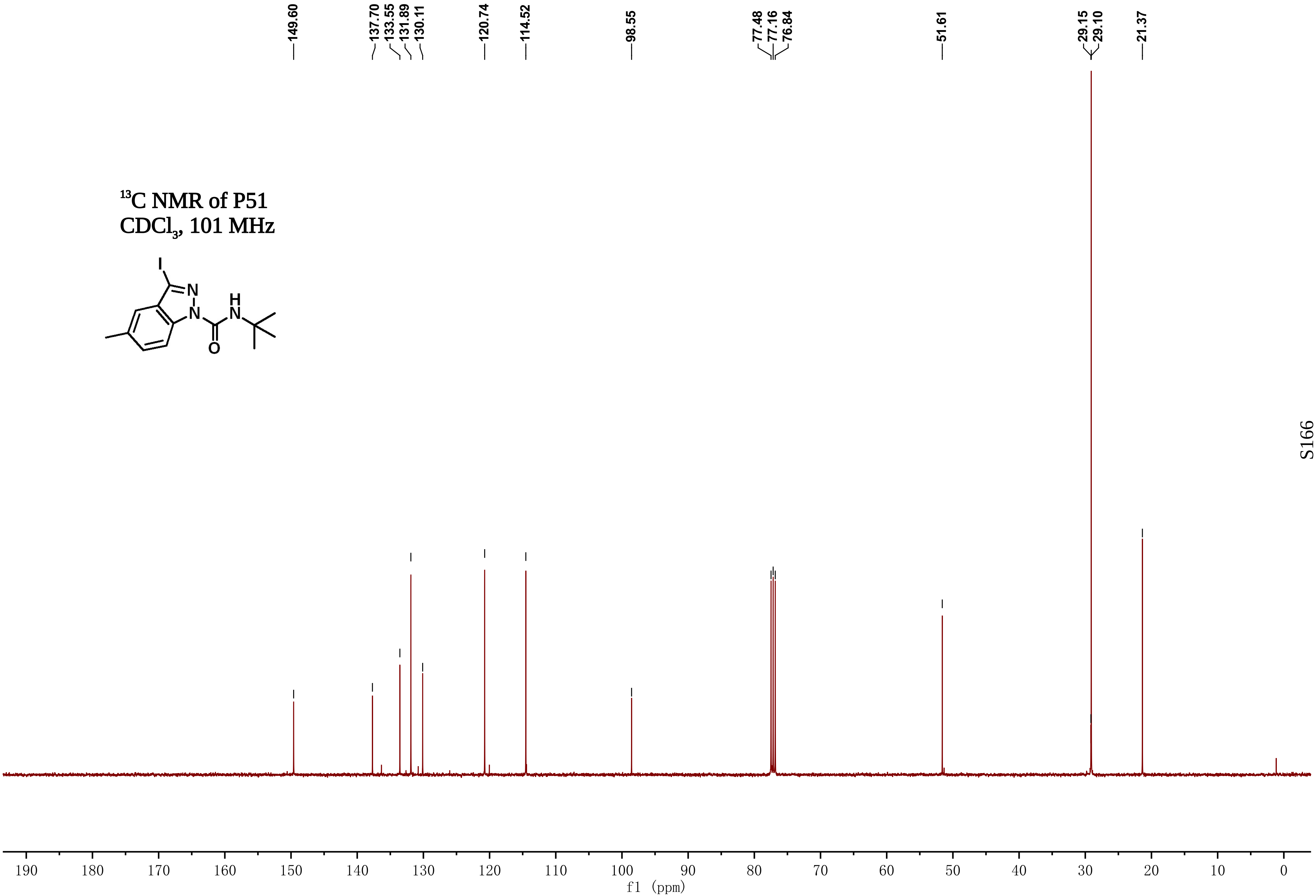

^{13}C NMR of P51
$CDCl_3$, 101 MHz
149.60
137.70
133.55
131.89
130.11
120.74
114.52
98.55
77.48
77.16
76.84
51.61
29.15
29.10
21.37
190 180 170 160 150 140 130 120 110 100 90 80 70 60 50 40 30 20 10 0
f1 (ppm)

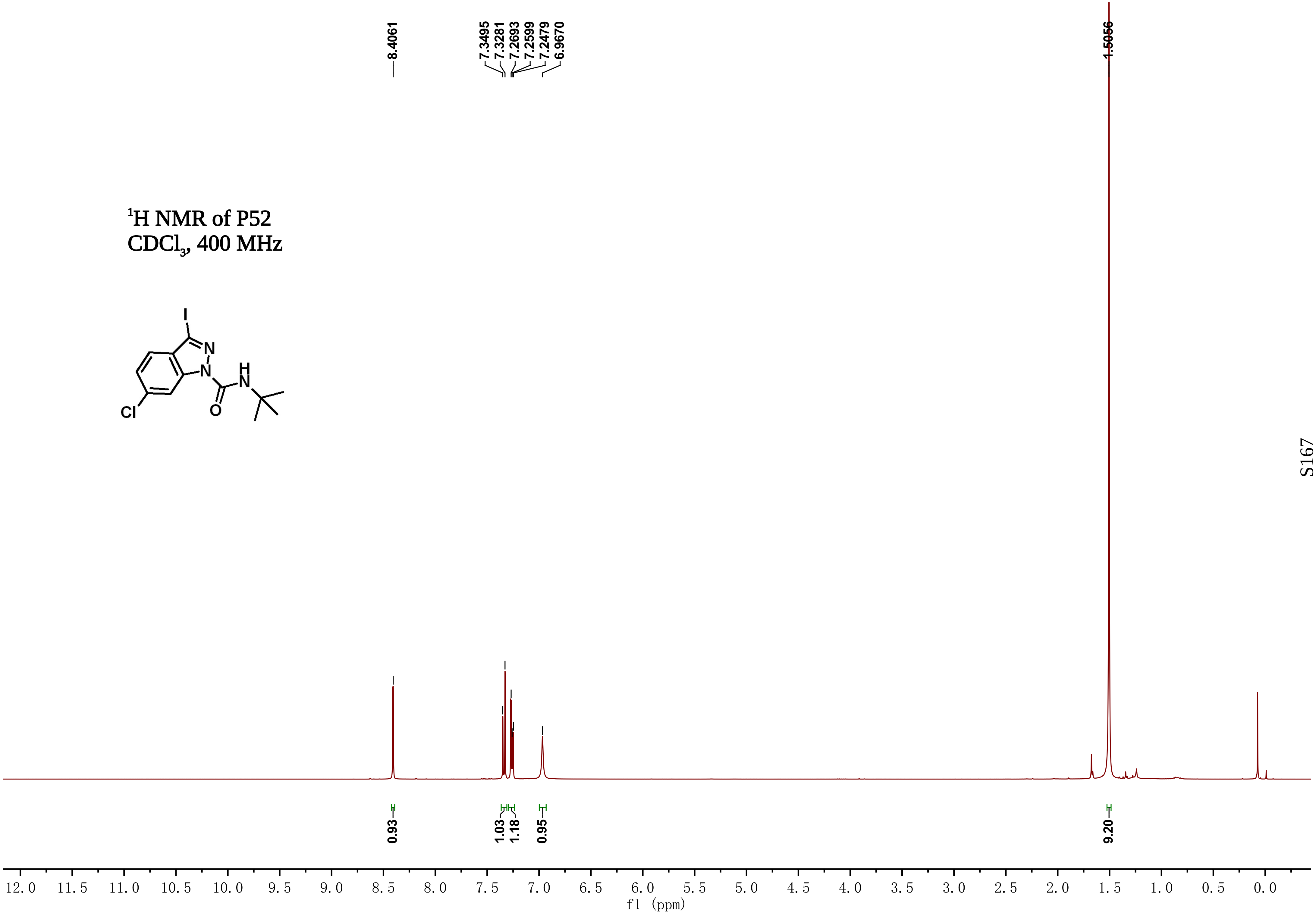

^{1}H NMR of P52
$CDCl_3$, 400 MHz

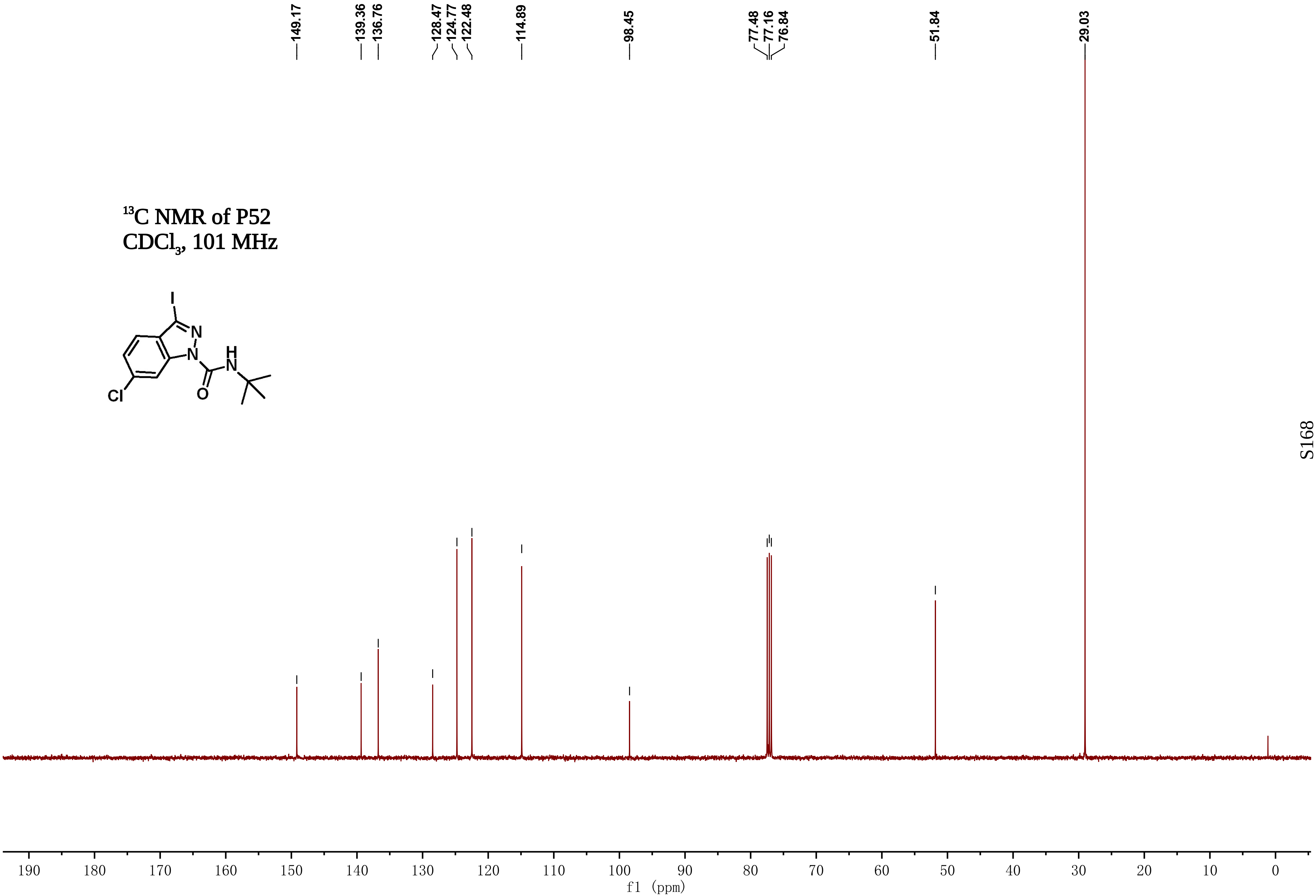

^{13}C NMR of P52
$CDCl_3$, 101 MHz

^{1}H NMR of P53
$CDCl_3$, 400 MHz

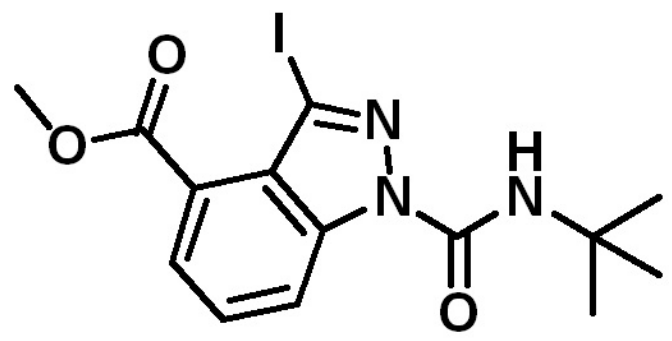

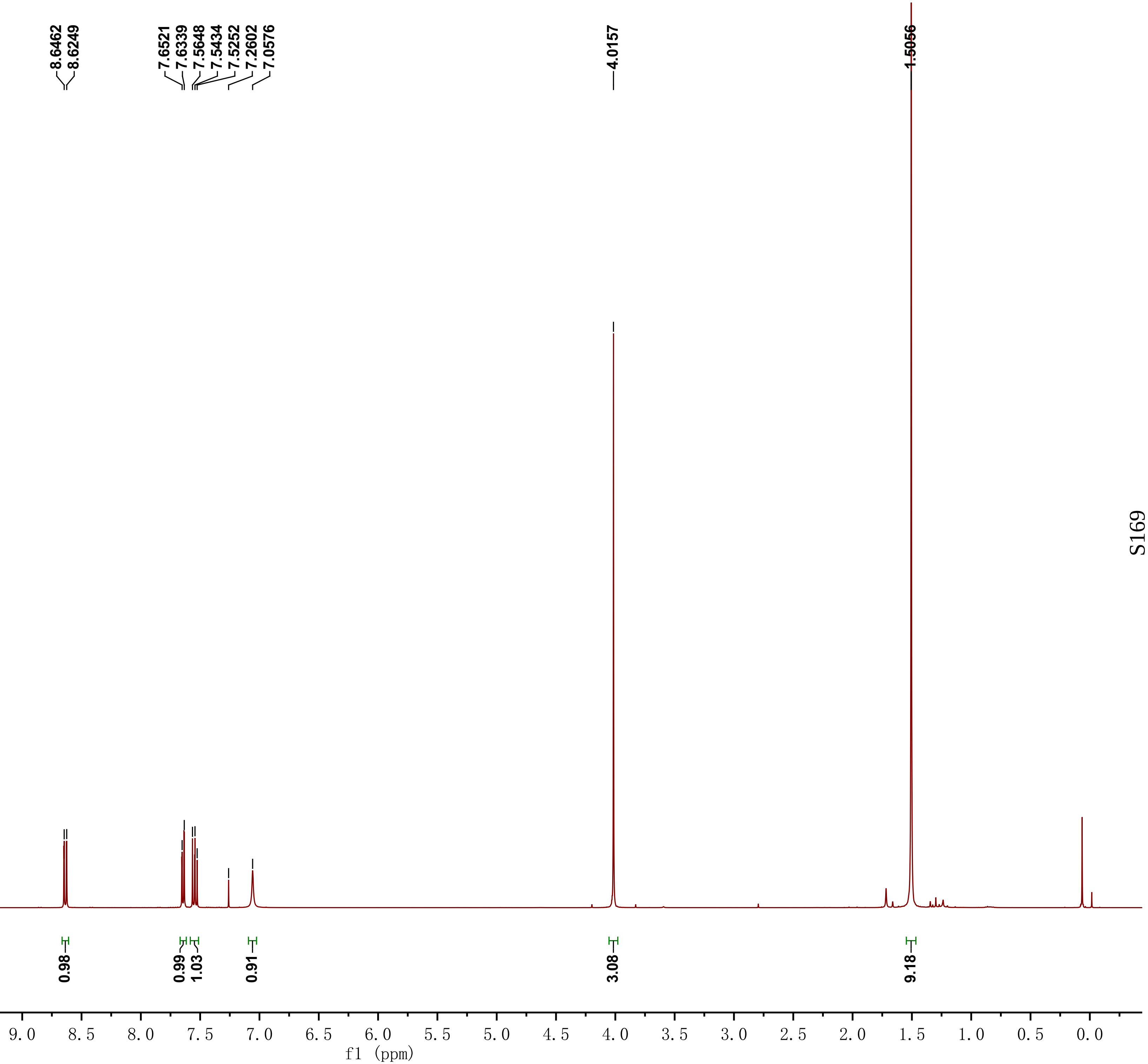

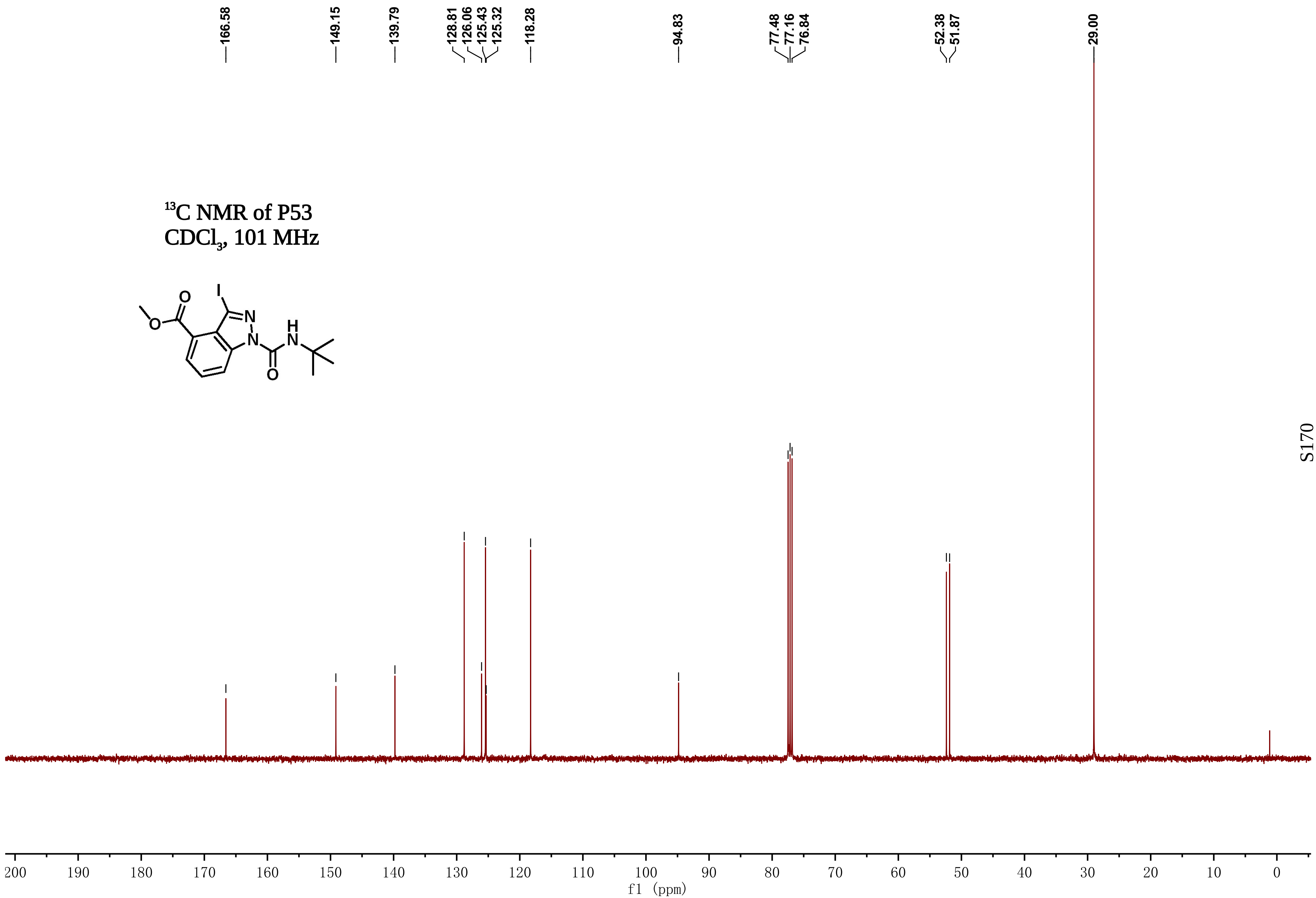
166.58
149.15
139.79
128.81
126.06
125.43
125.32
118.28
94.83
77.48
77.16
76.84
52.38
51.87
29.00
^{13}C NMR of P53
$CDCl_3$, 101 MHz
I
N
N
H
N
O
O
O
200 190 180 170 160 150 140 130 120 110 100 90 80 70 60 50 40 30 20 10 0
f1 (ppm)

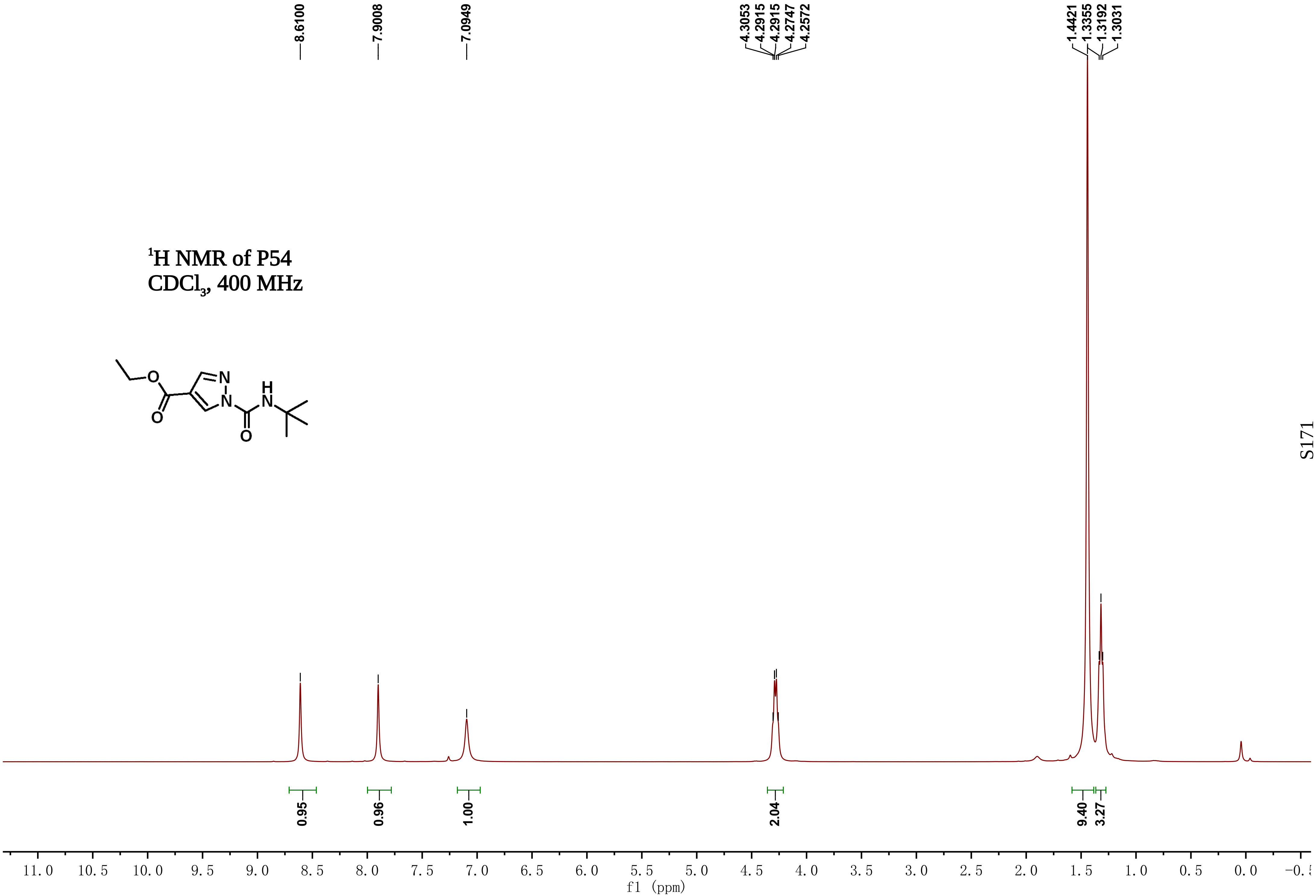

1H NMR of P54
CDCl3, 400 MHz
8.6100
7.9008
7.0949
4.3053
4.2915
4.2915
4.2747
4.2572
1.4421
1.3355
1.3192
1.3031
0.95
0.96
1.00
2.04
9.40
3.27
11.0 10.5 10.0 9.5 9.0 8.5 8.0 7.5 7.0 6.5 6.0 5.5 5.0 4.5 4.0 3.5 3.0 2.5 2.0 1.5 1.0 0.5 0.0
f1 (ppm)

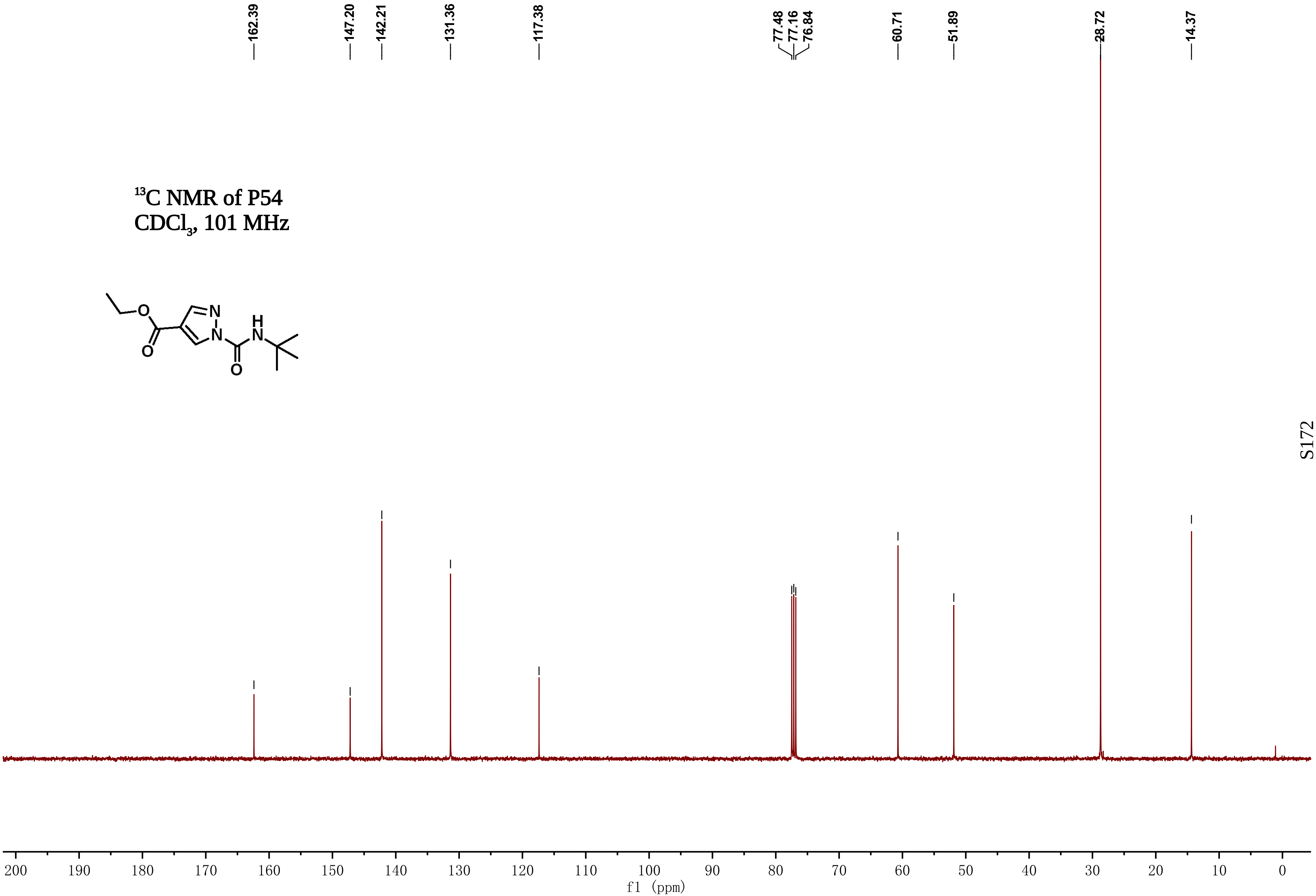
162.39
147.20
142.21
131.36
117.38
77.48
77.16
76.84
60.71
51.89
28.72
14.37
^{13}C NMR of P54
$CDCl_3$, 101 MHz
200 190 180 170 160 150 140 130 120 110 100 90 80 70 60 50 40 30 20 10 0
f1 (ppm)

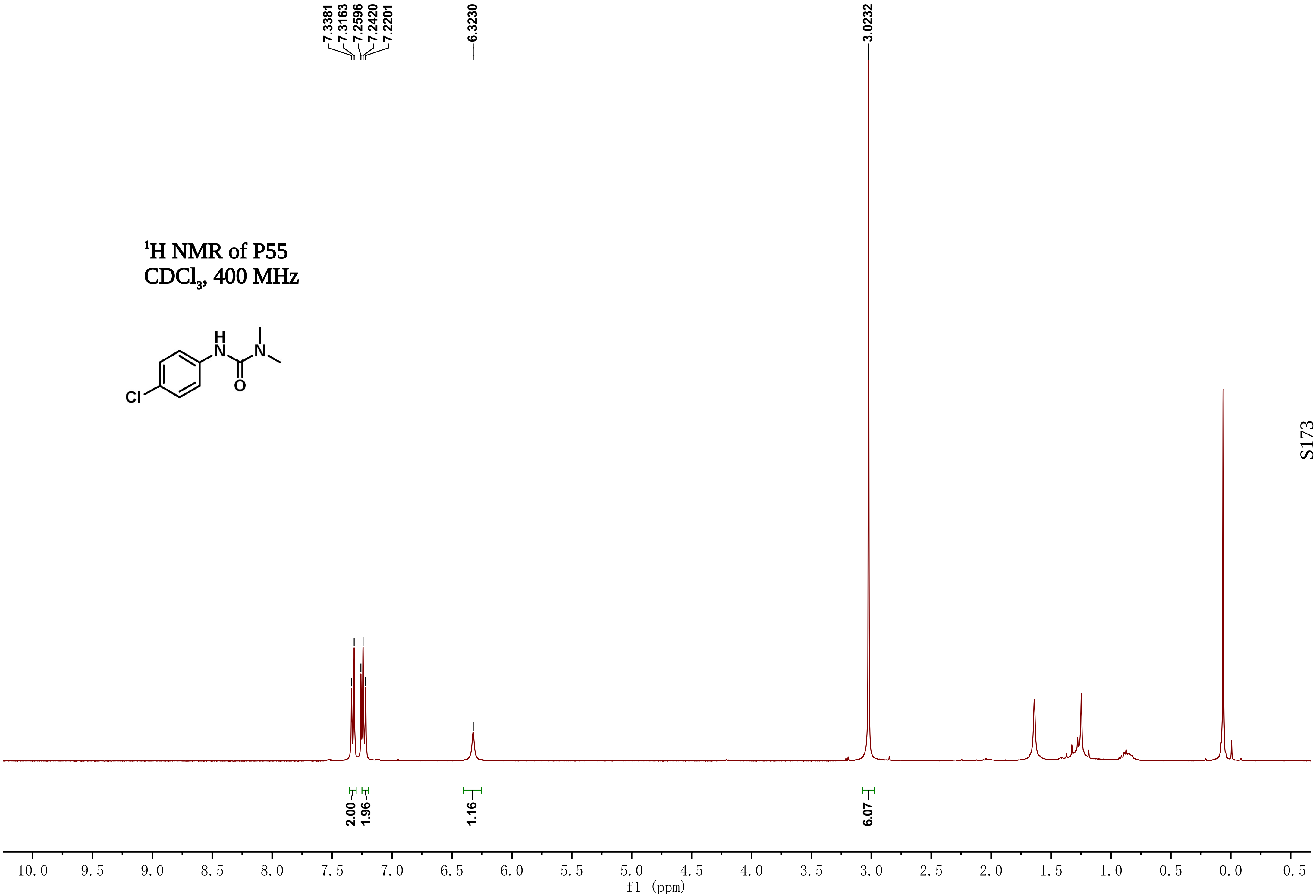

7.3381
7.3163
7.2596
7.2420
7.2201
6.3230
3.0232
^{1}H NMR of P55
$CDCl_3$, 400 MHz
H
N
N
O
Cl
2.00
1.96
1.16
6.07
10.0 9.5 9.0 8.5 8.0 7.5 7.0 6.5 6.0 5.5 5.0 4.5 4.0 3.5 3.0 2.5 2.0 1.5 1.0 0.5 0.0 −0.5
f1 (ppm)

^{13}C NMR of P55
$CDCl_3$, 101 MHz

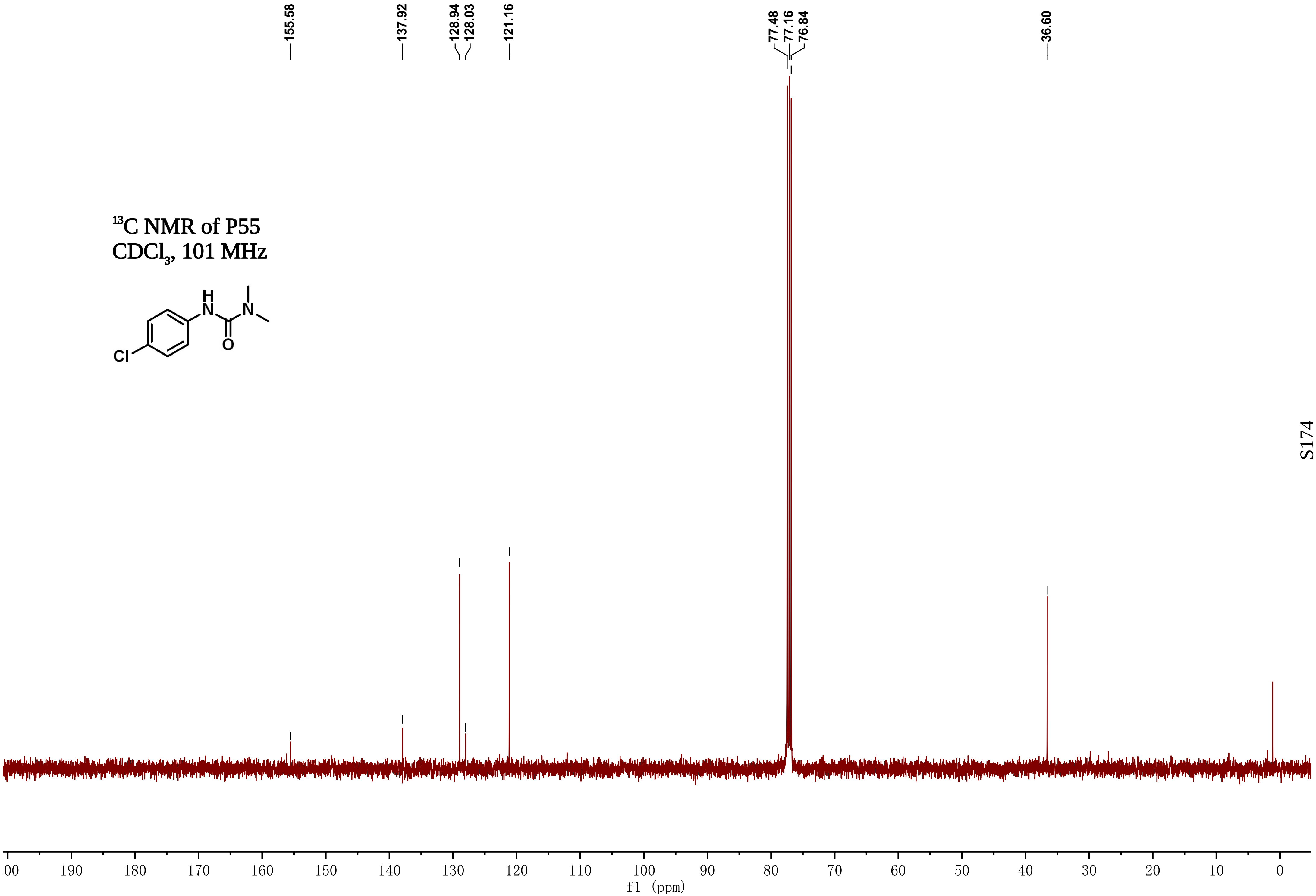

^{1}H NMR of P56
$CDCl_3$, 400 MHz

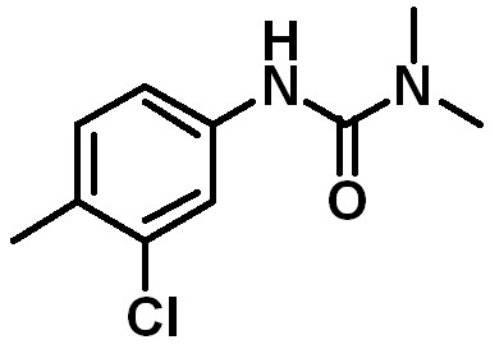

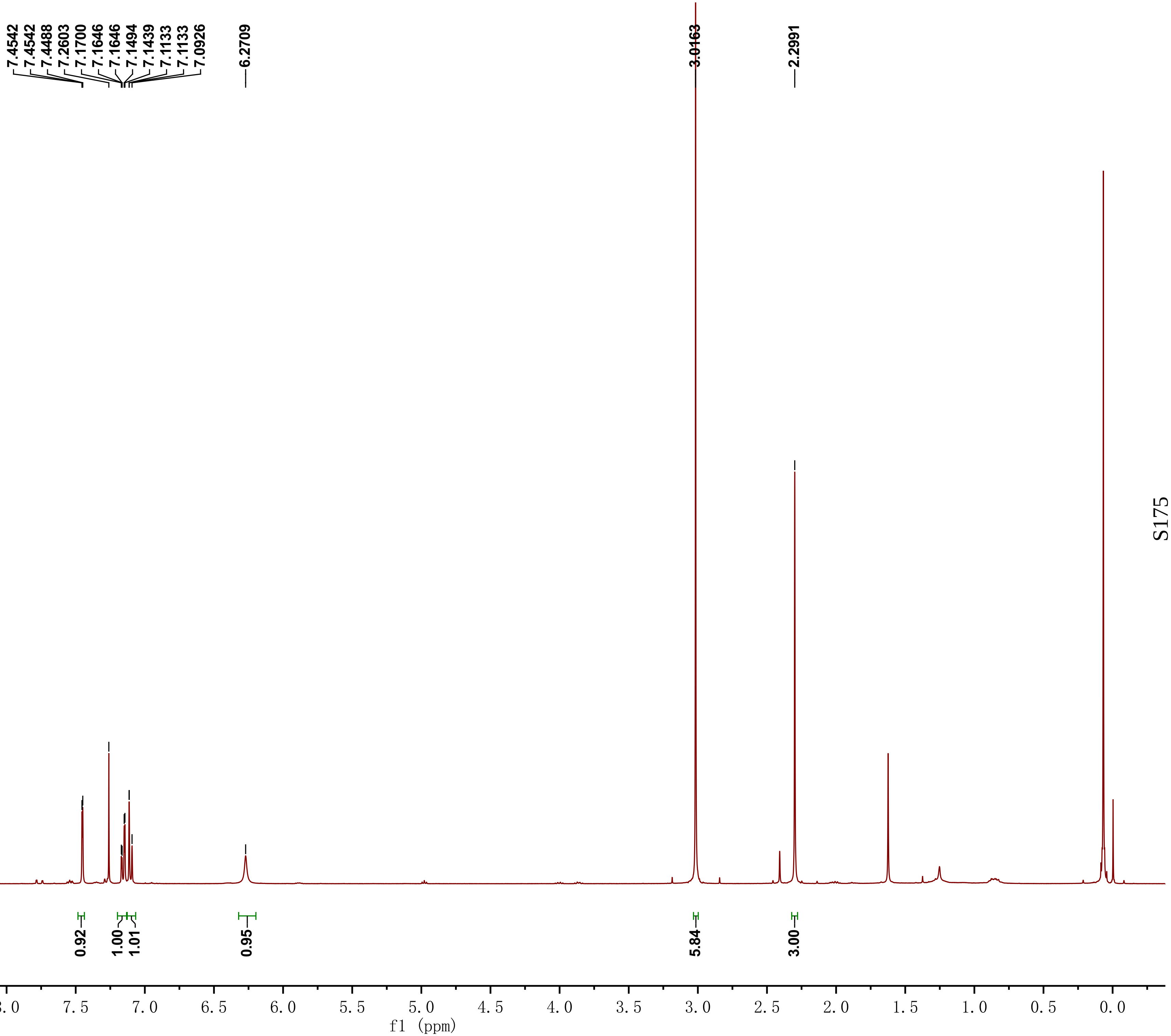

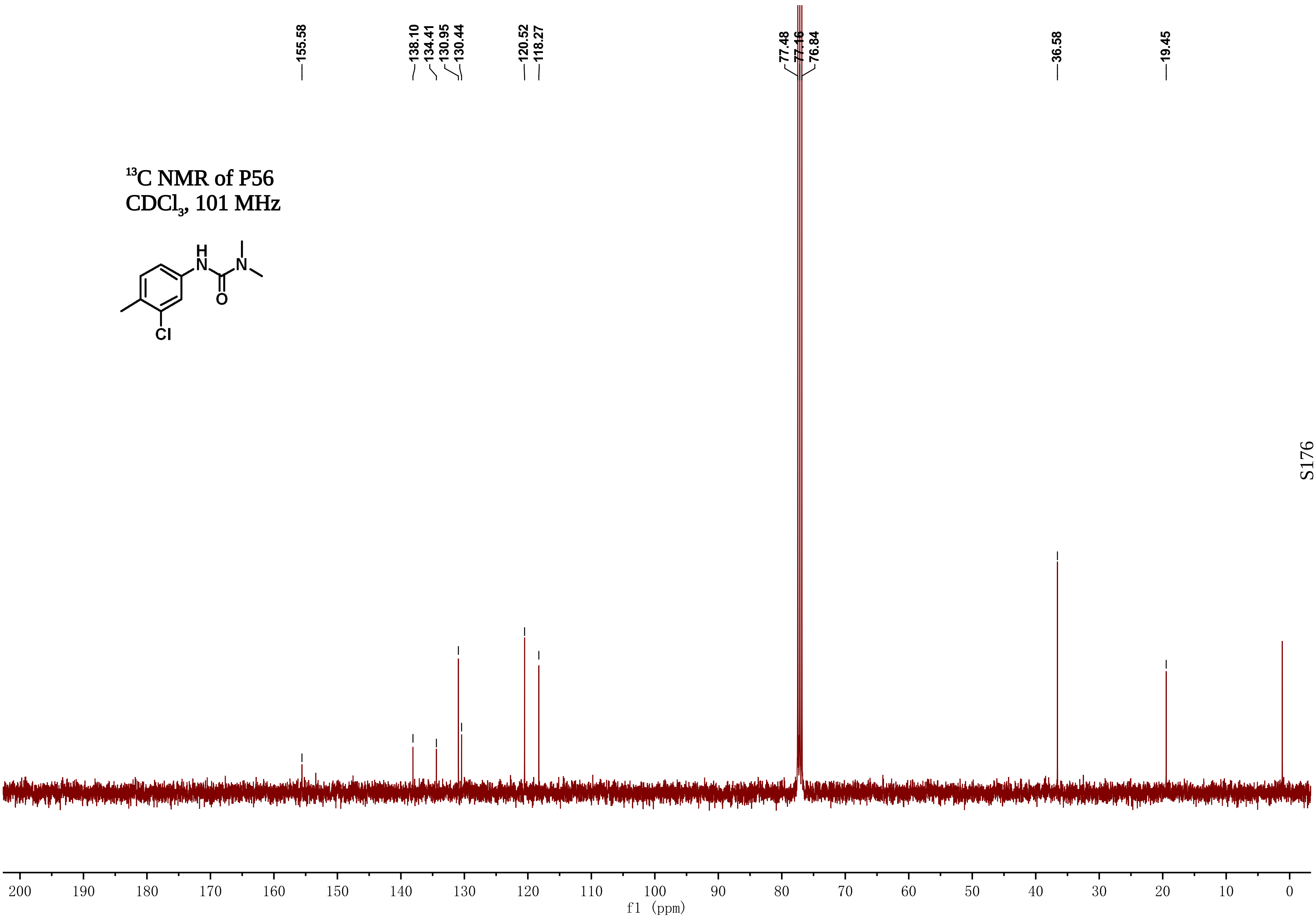

155.58
138.10
134.41
130.95
130.44
120.52
118.27
77.48
77.16
76.84
36.58
19.45
13C NMR of P56
CDCl3, 101 MHz
H
N
N
O
Cl
200 190 180 170 160 150 140 130 120 110 100 90 80 70 60 50 40 30 20 10 0
f1 (ppm)

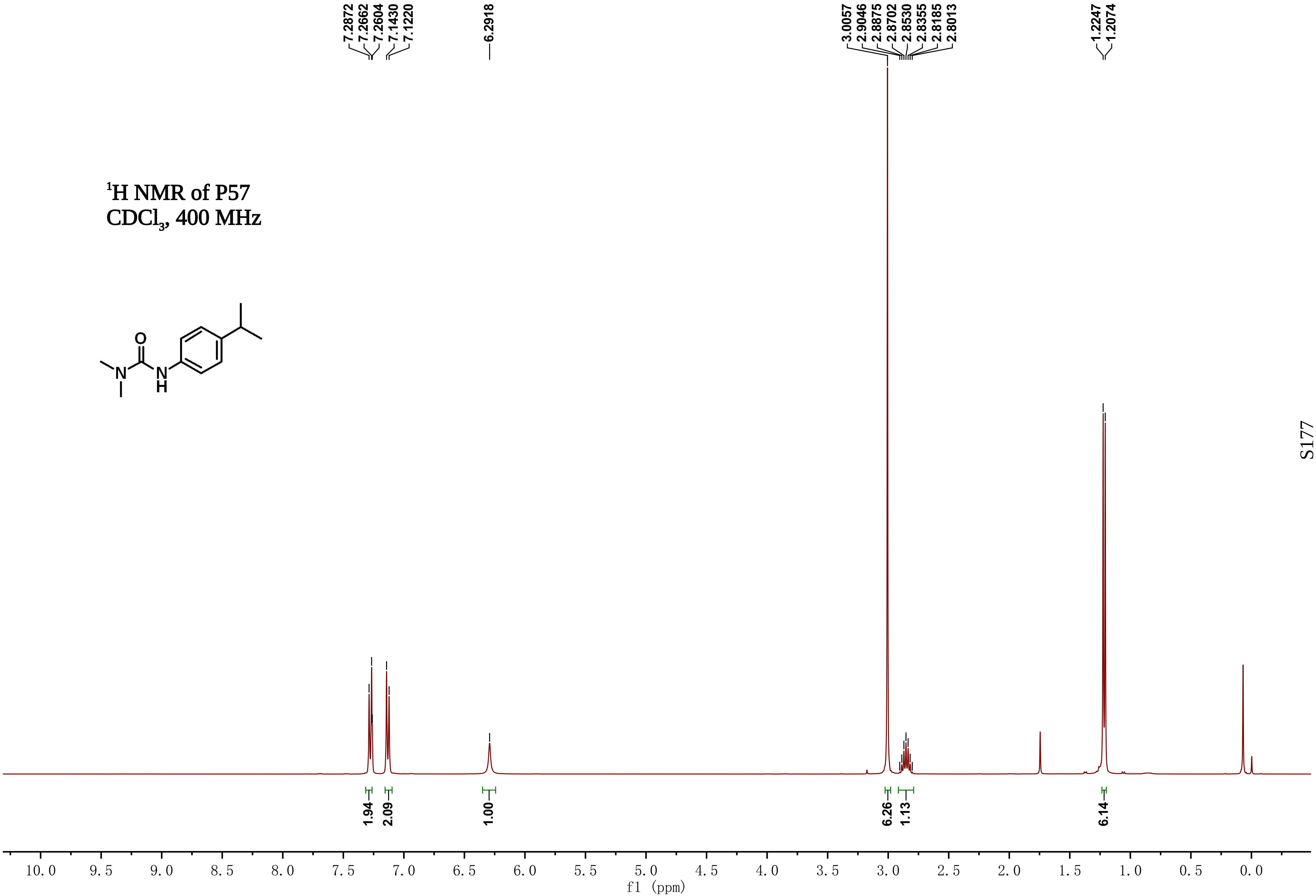

^{1}H NMR of P57
$CDCl_3$, 400 MHz
O
N
N
H
7.2872
7.2662
7.2604
7.1430
7.1220
6.2918
3.0057
2.9046
2.8875
2.8702
2.8530
2.8355
2.8185
2.8013
1.2247
1.2074
1.94
2.09
1.00
6.26
1.13
6.14
10.0 9.5 9.0 8.5 8.0 7.5 7.0 6.5 6.0 5.5 5.0 4.5 4.0 3.5 3.0 2.5 2.0 1.5 1.0 0.5 0.0
f1 (ppm)

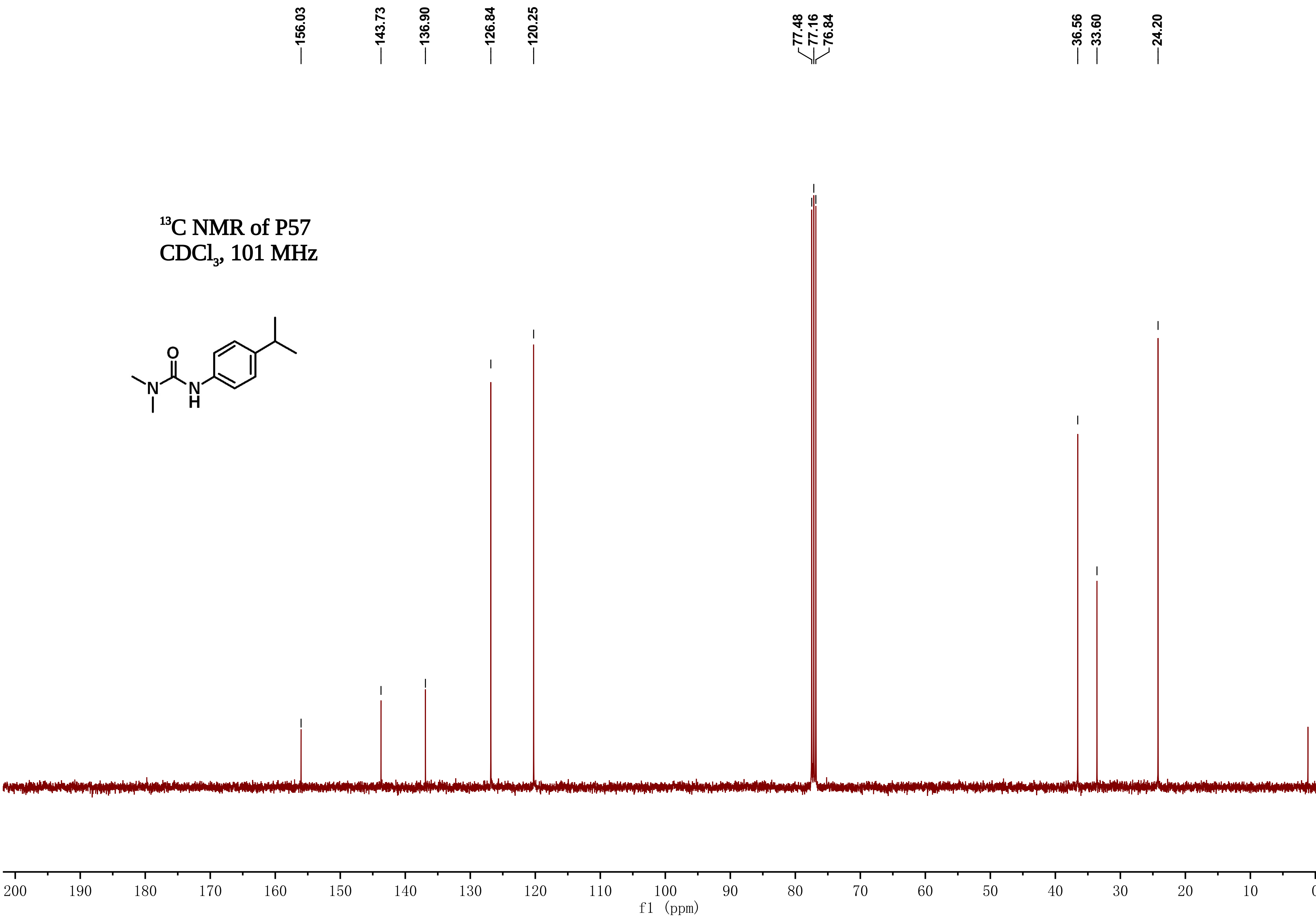
156.03
143.73
136.90
126.84
120.25
77.48
77.16
76.84
36.56
33.60
24.20
13C NMR of P57
CDCl3, 101 MHz
O
N
N
H
200 190 180 170 160 150 140 130 120 110 100 90 80 70 60 50 40 30 20 10 0
f1 (ppm)

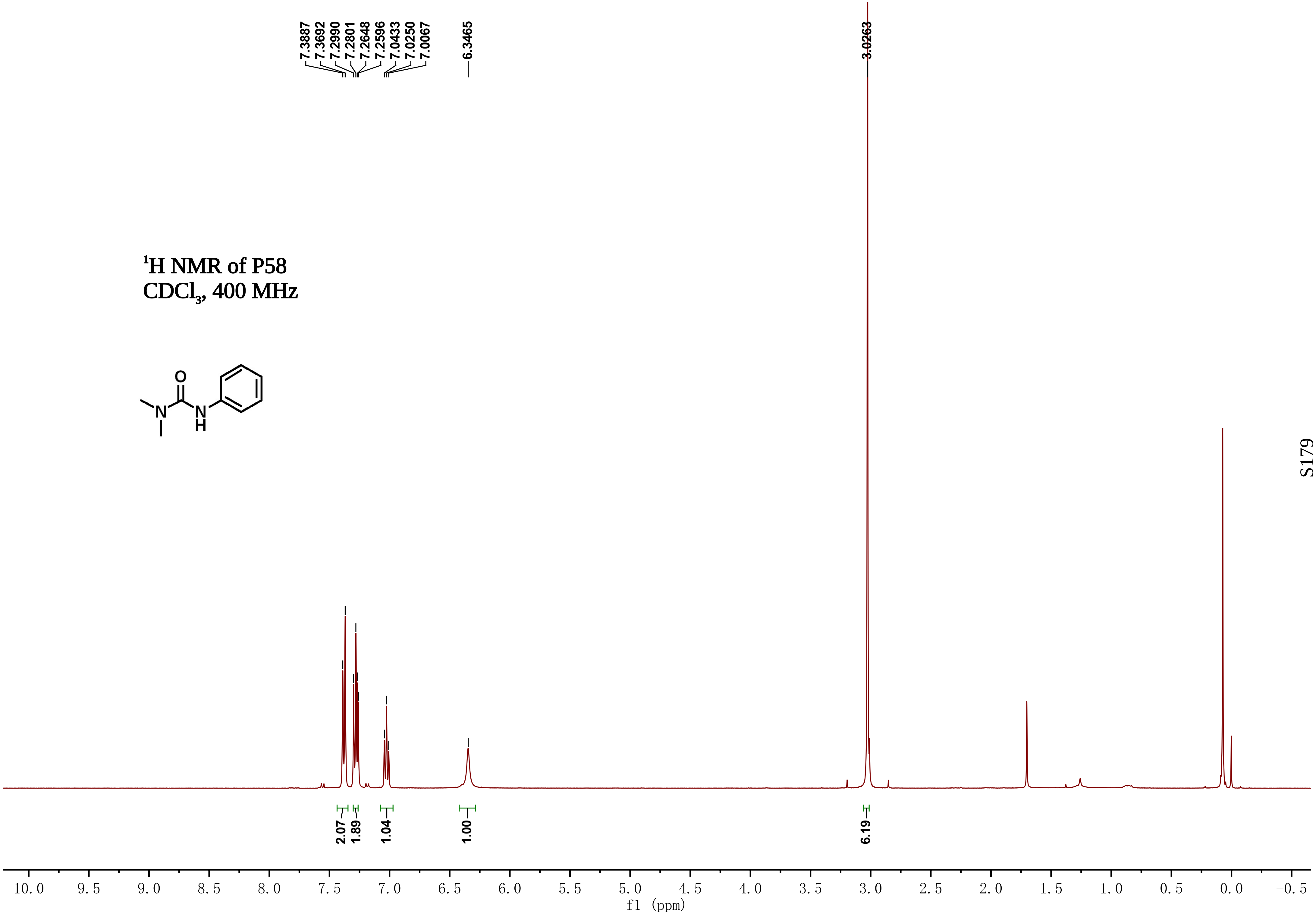

^{1}H NMR of P58

$CDCl_3$, 400 MHz

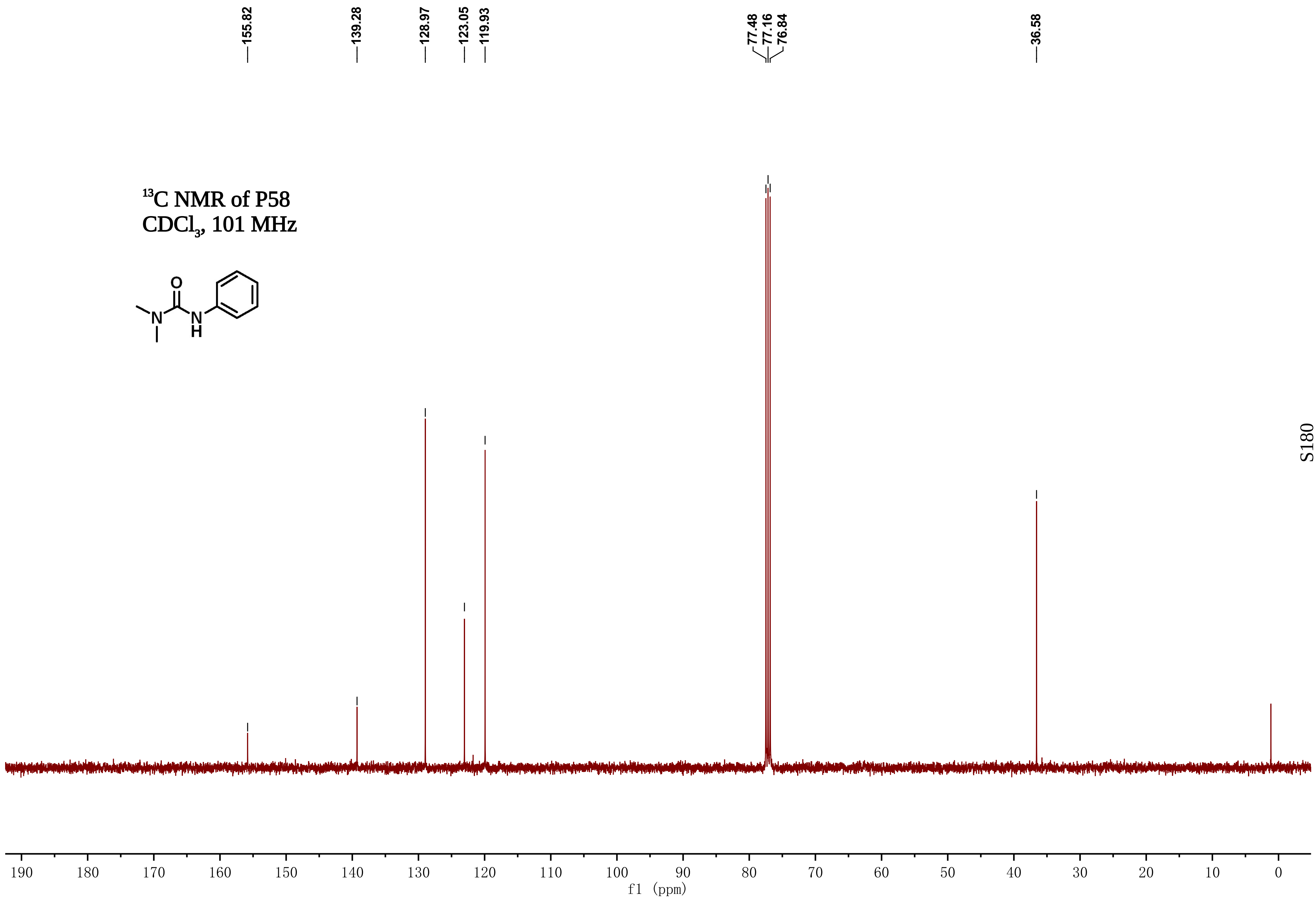
155.82
139.28
128.97
123.05
119.93
77.48
77.16
76.84
36.58
^{13}C NMR of P58
$CDCl_3$, 101 MHz
O
N
N
H
190 180 170 160 150 140 130 120 110 100 90 80 70 60 50 40 30 20 10 0
f1 (ppm)

^{1}H NMR of P59
$CDCl_3$, 400 MHz

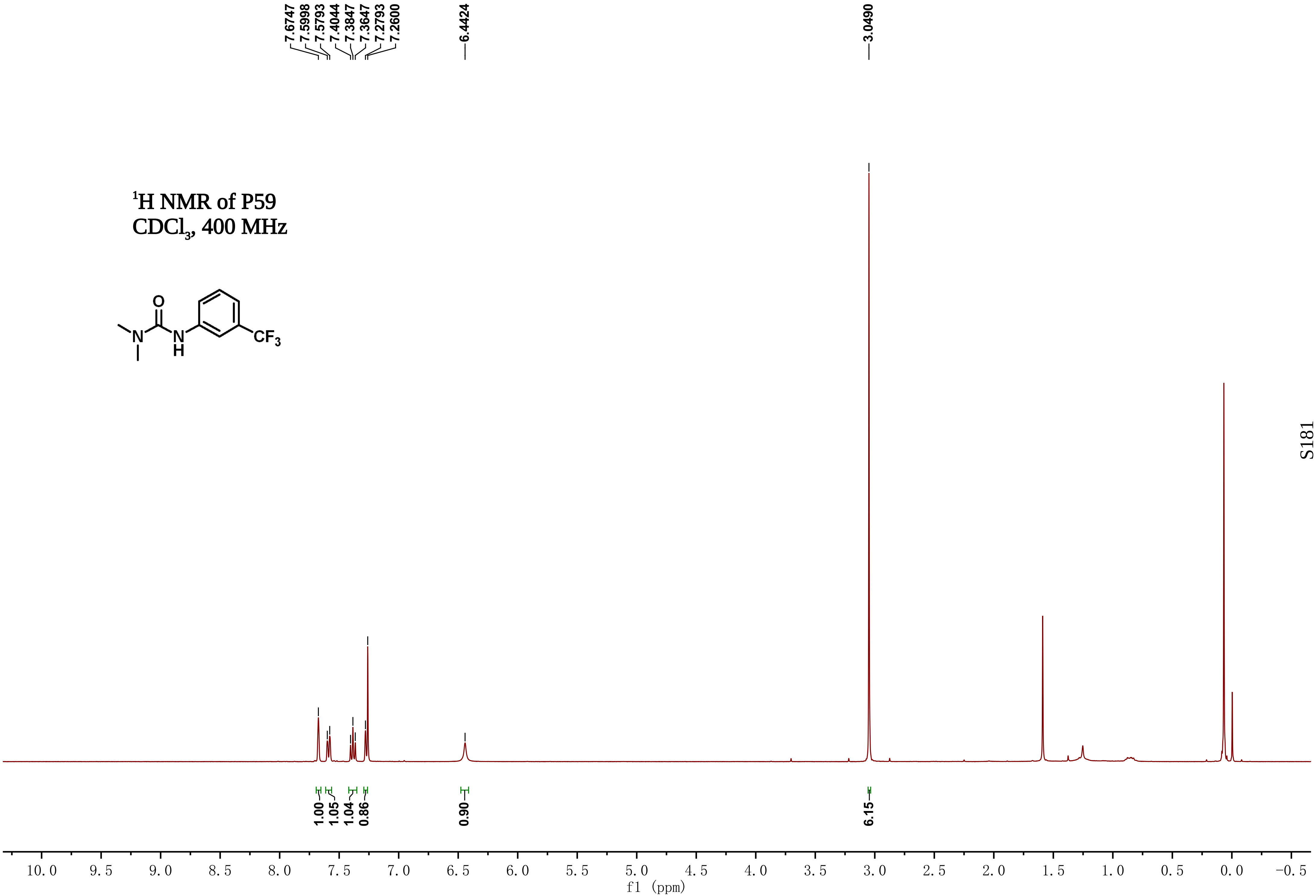

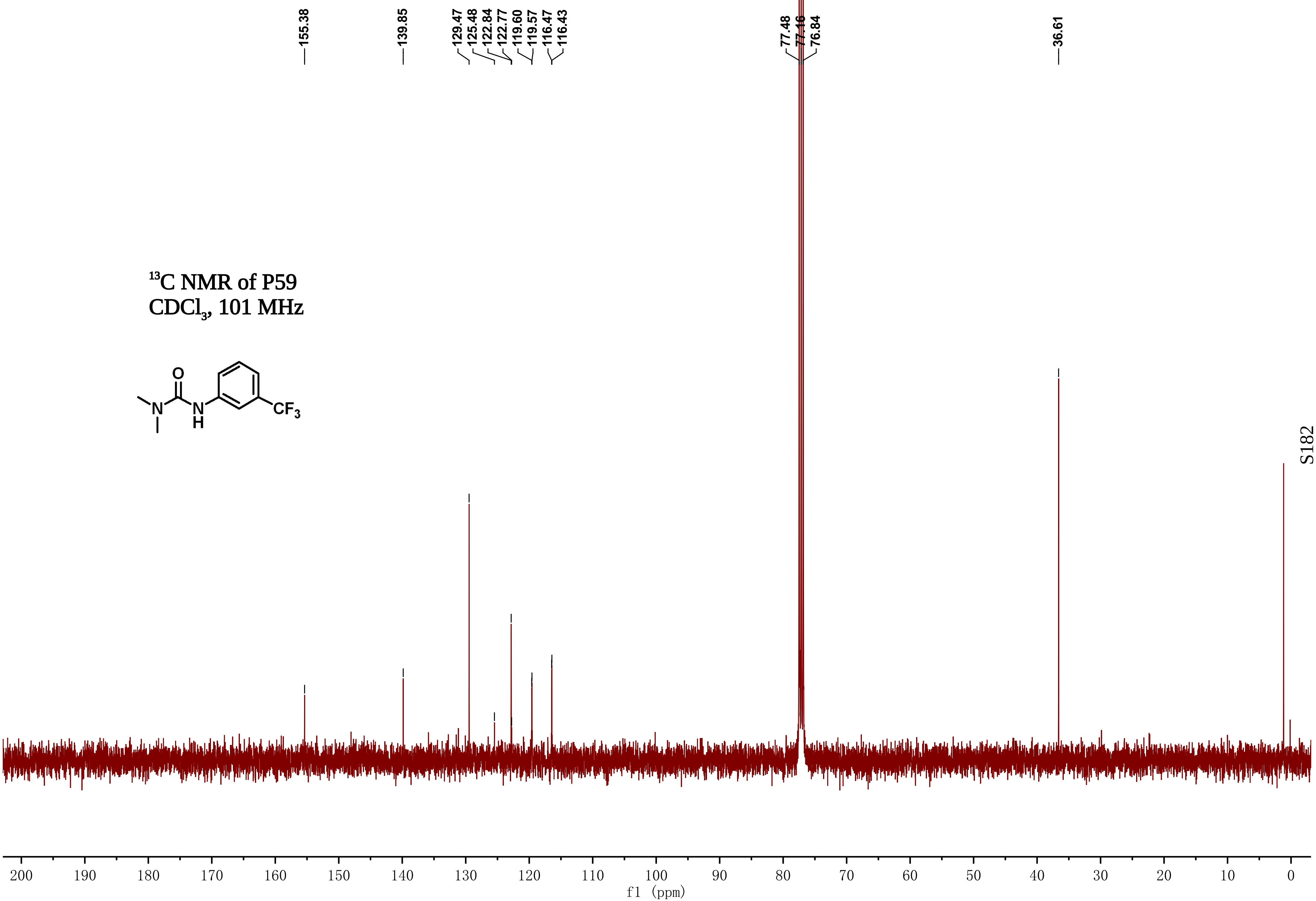
155.38
139.85
129.47
125.48
122.84
122.77
119.60
119.57
116.47
116.43
77.48
77.16
76.84
36.61
^{13}C NMR of P59
$CDCl_3$, 101 MHz
O
N
N
H
CF_3
200 190 180 170 160 150 140 130 120 110 100 90 80 70 60 50 40 30 20 10 0
f1 (ppm)

^{19}F NMR of P59
$CDCl_3$, 376 MHz

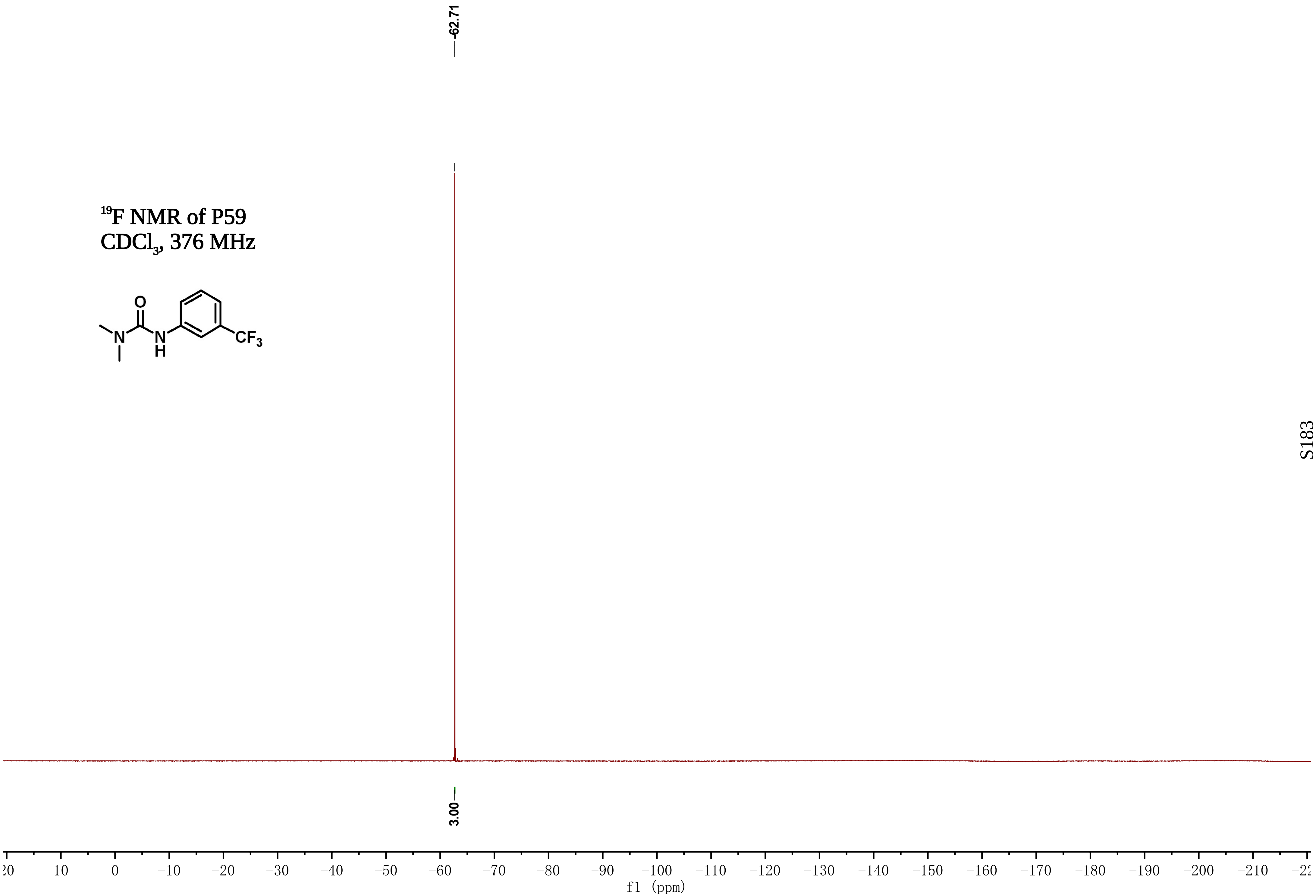

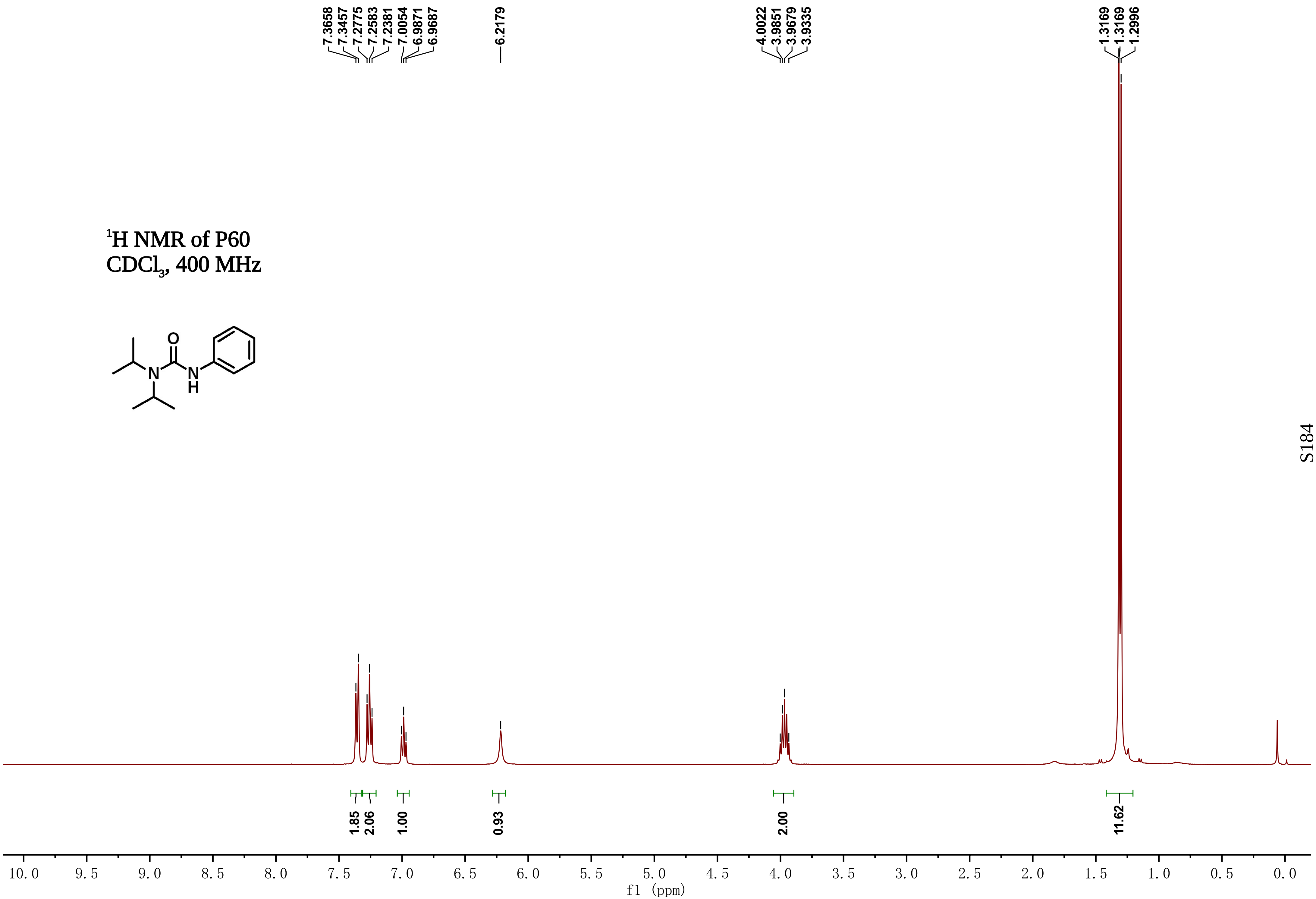

7.3658
7.3457
7.2775
7.2583
7.2381
7.0054
6.9871
6.9687
6.2179
4.0022
3.9851
3.9679
3.9335
1.3169
1.3169
1.2996
¹H NMR of P60
CDCl3, 400 MHz
O
N
N
H
1.85
2.06
1.00
0.93
2.00
11.62
10.0
9.5
9.0
8.5
8.0
7.5
7.0
6.5
6.0
5.5
5.0
4.5
4.0
3.5
3.0
2.5
2.0
1.5
1.0
0.5
0.0
f1 (ppm)

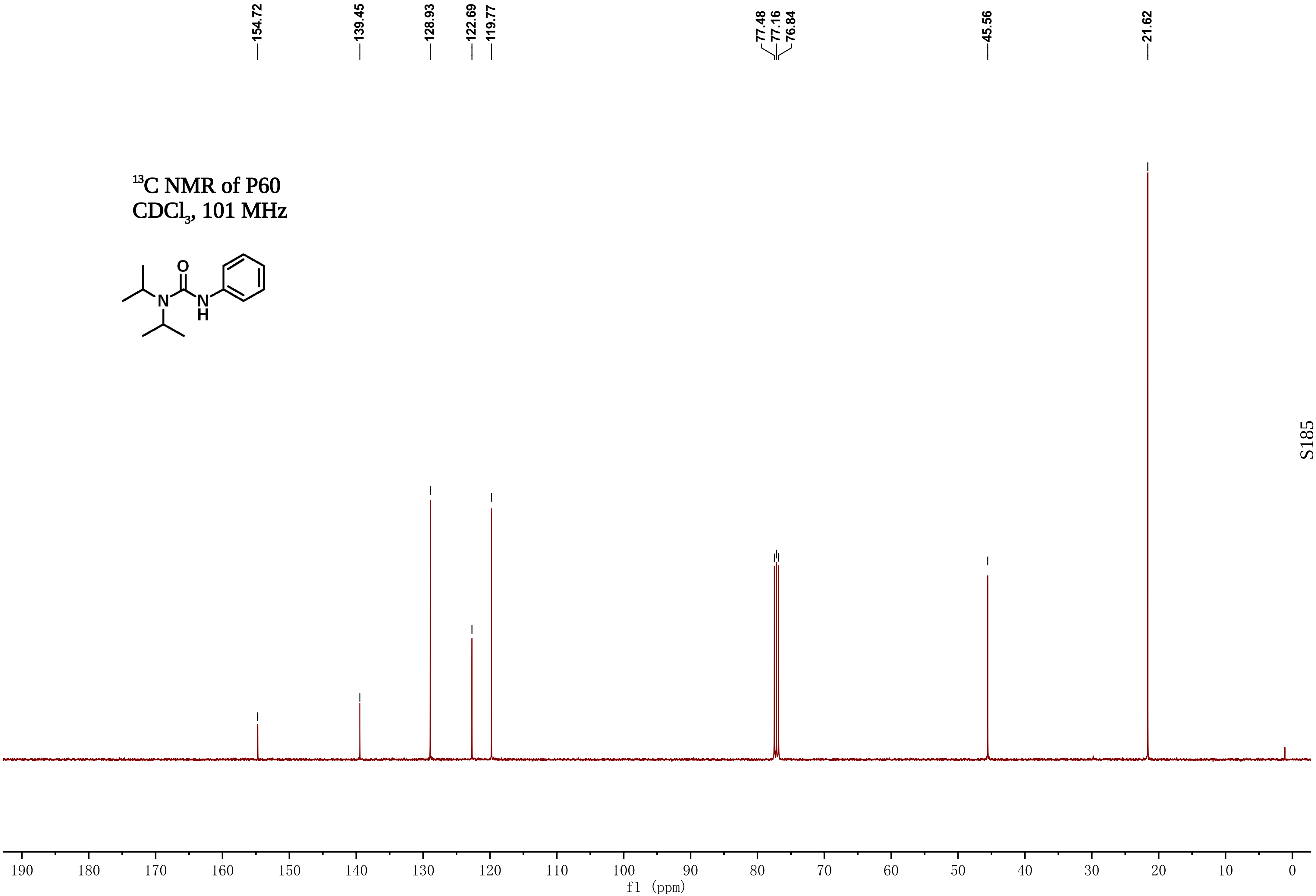
154.72
139.45
128.93
122.69
119.77
77.48
77.16
76.84
45.56
21.62
^{13}C NMR of P60
$CDCl_3$, 101 MHz
O
N
N
H
190 180 170 160 150 140 130 120 110 100 90 80 70 60 50 40 30 20 10 0
f1 (ppm)

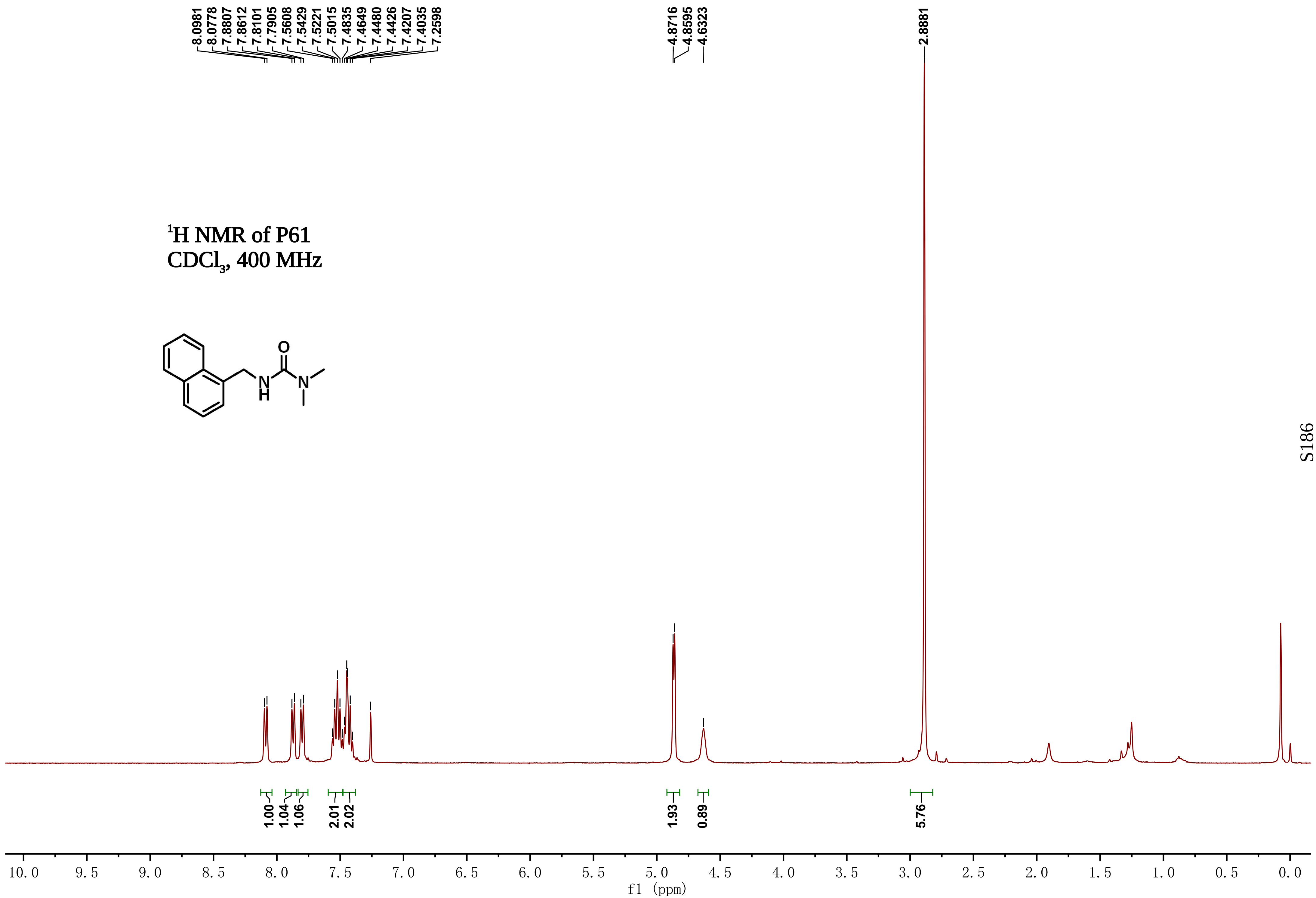

8.0981
8.0778
7.8807
7.8612
7.8101
7.7905
7.5608
7.5429
7.5221
7.5015
7.4835
7.4649
7.4480
7.4426
7.4207
7.4035
7.2598
4.8716
4.8595
4.6323
2.8881
^{1}H NMR of P61
$CDCl_3$, 400 MHz
O
N
H
N
1.00
1.04
1.06
2.01
2.02
1.93
0.89
5.76
10.0
9.5
9.0
8.5
8.0
7.5
7.0
6.5
6.0
5.5
5.0
4.5
4.0
3.5
3.0
2.5
2.0
1.5
1.0
0.5
0.0
f1 (ppm)

^{13}C NMR of P61
$CDCl_3$, 101 MHz

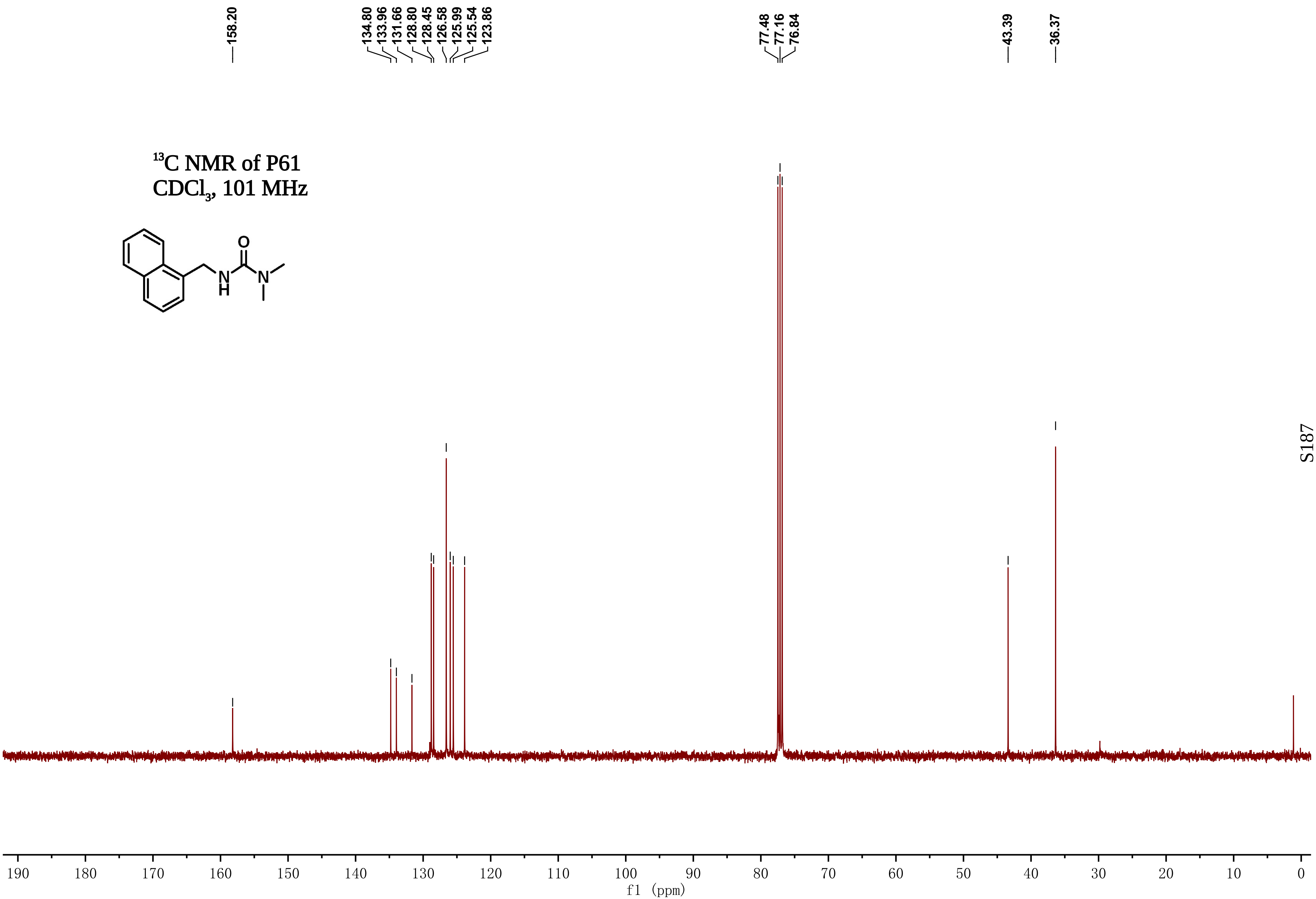

^{1}H NMR of P62
$CDCl_3$, 400 MHz

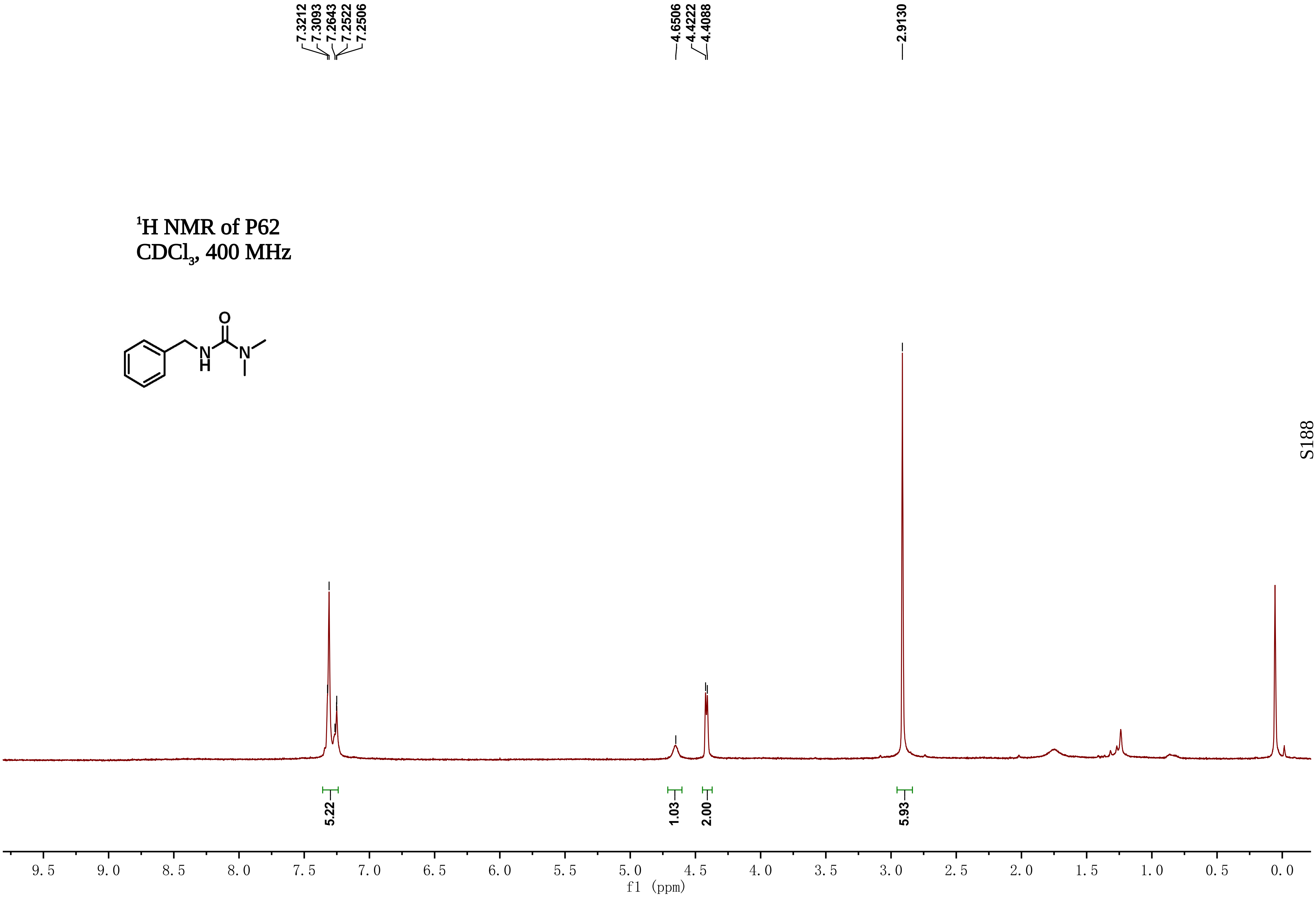

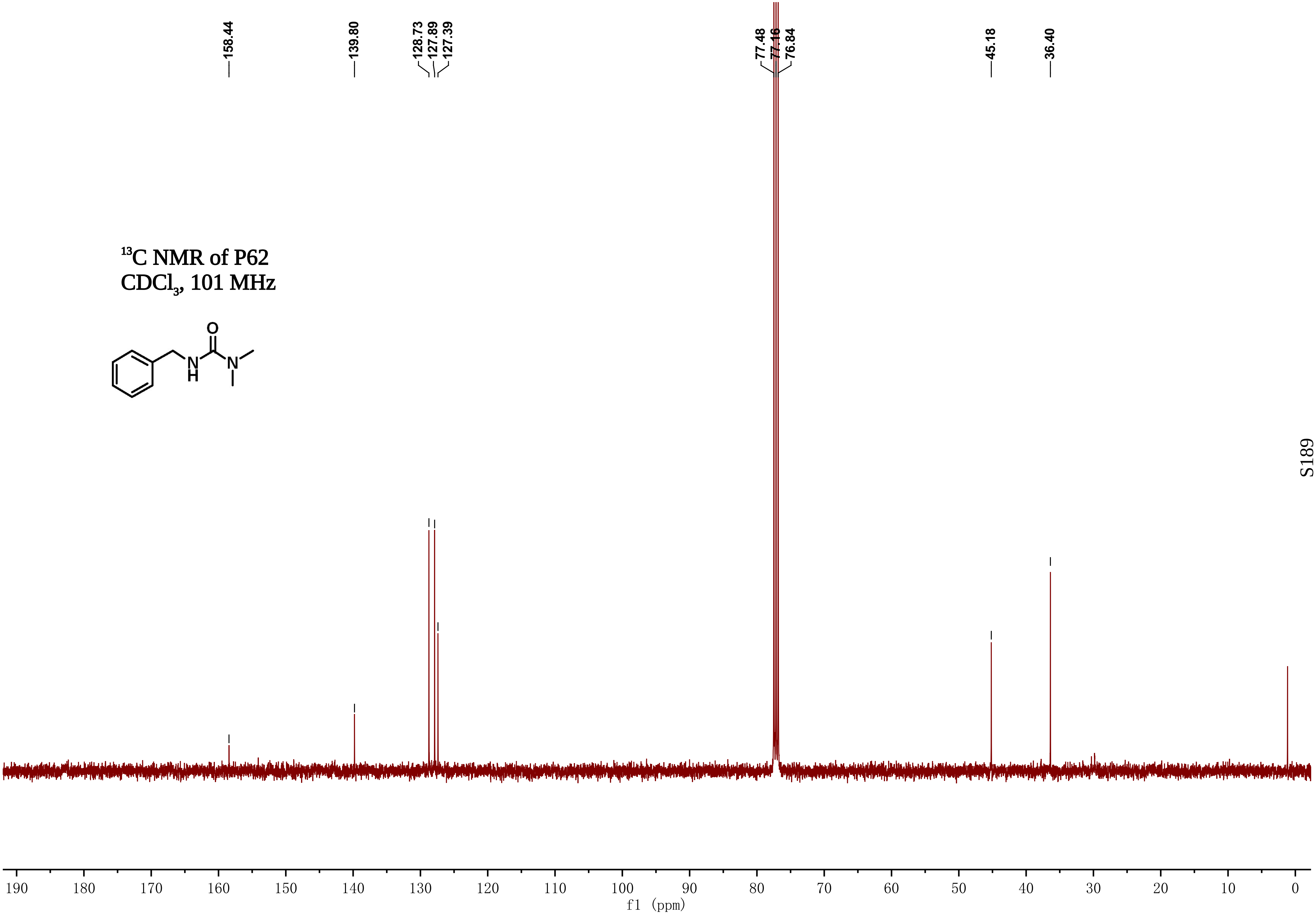

158.44
139.80
128.73
127.89
127.39
77.48
77.16
76.84
45.18
36.40
13C NMR of P62
CDCl3, 101 MHz
O
N
H
N
190 180 170 160 150 140 130 120 110 100 90 80 70 60 50 40 30 20 10 0
f1 (ppm)

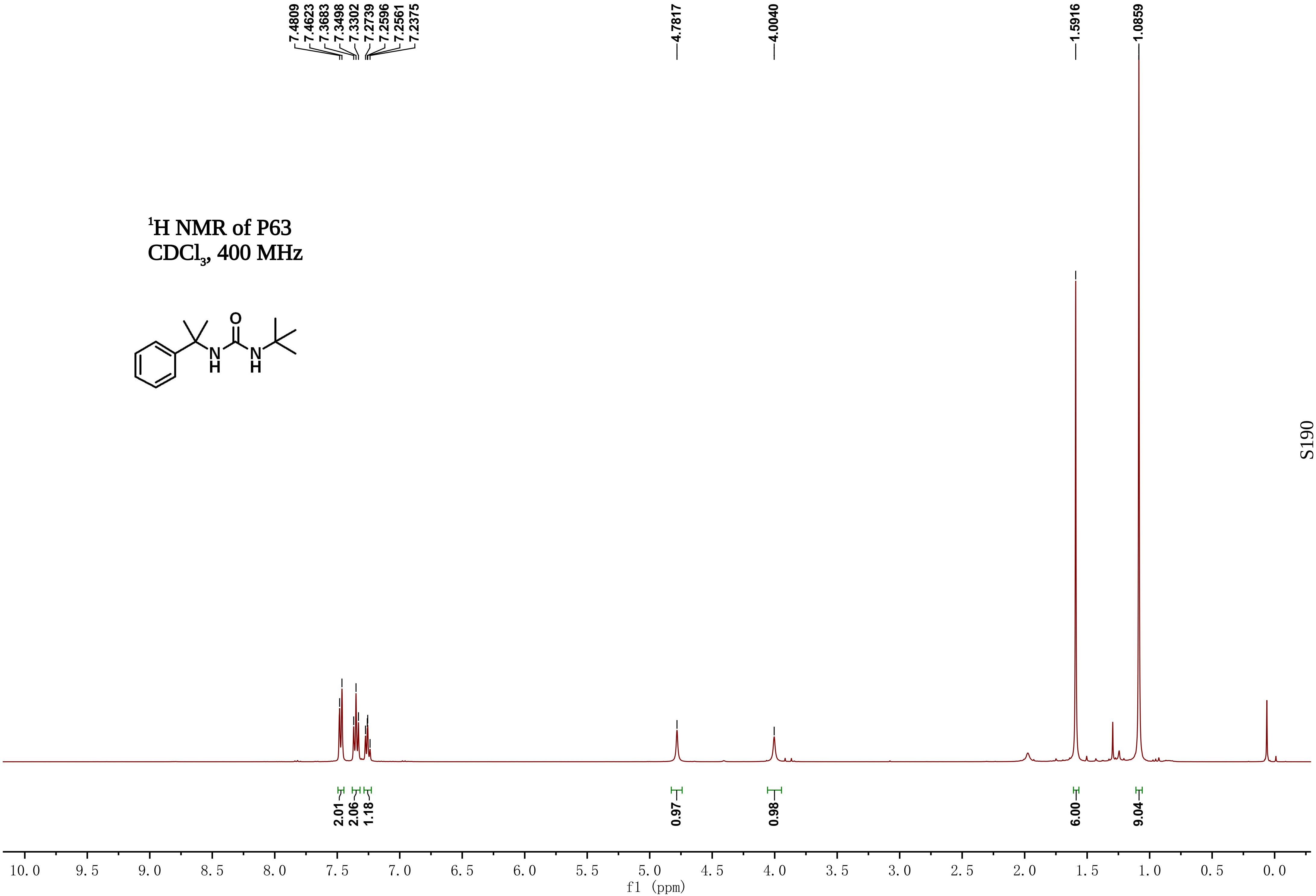
7.4809
7.4623
7.3683
7.3498
7.3302
7.2739
7.2596
7.2561
7.2375
4.7817
4.0040
1.5916
1.0859
^{1}H NMR of P63
$CDCl_3$, 400 MHz
O
N
H
N
H
2.01
2.06
1.18
0.97
0.98
6.00
9.04
10.0
9.5
9.0
8.5
8.0
7.5
7.0
6.5
6.0
5.5
5.0
4.5
4.0
3.5
3.0
2.5
2.0
1.5
1.0
0.5
0.0
f1 (ppm)

^{13}C NMR of P63
$CDCl_3$, 101 MHz

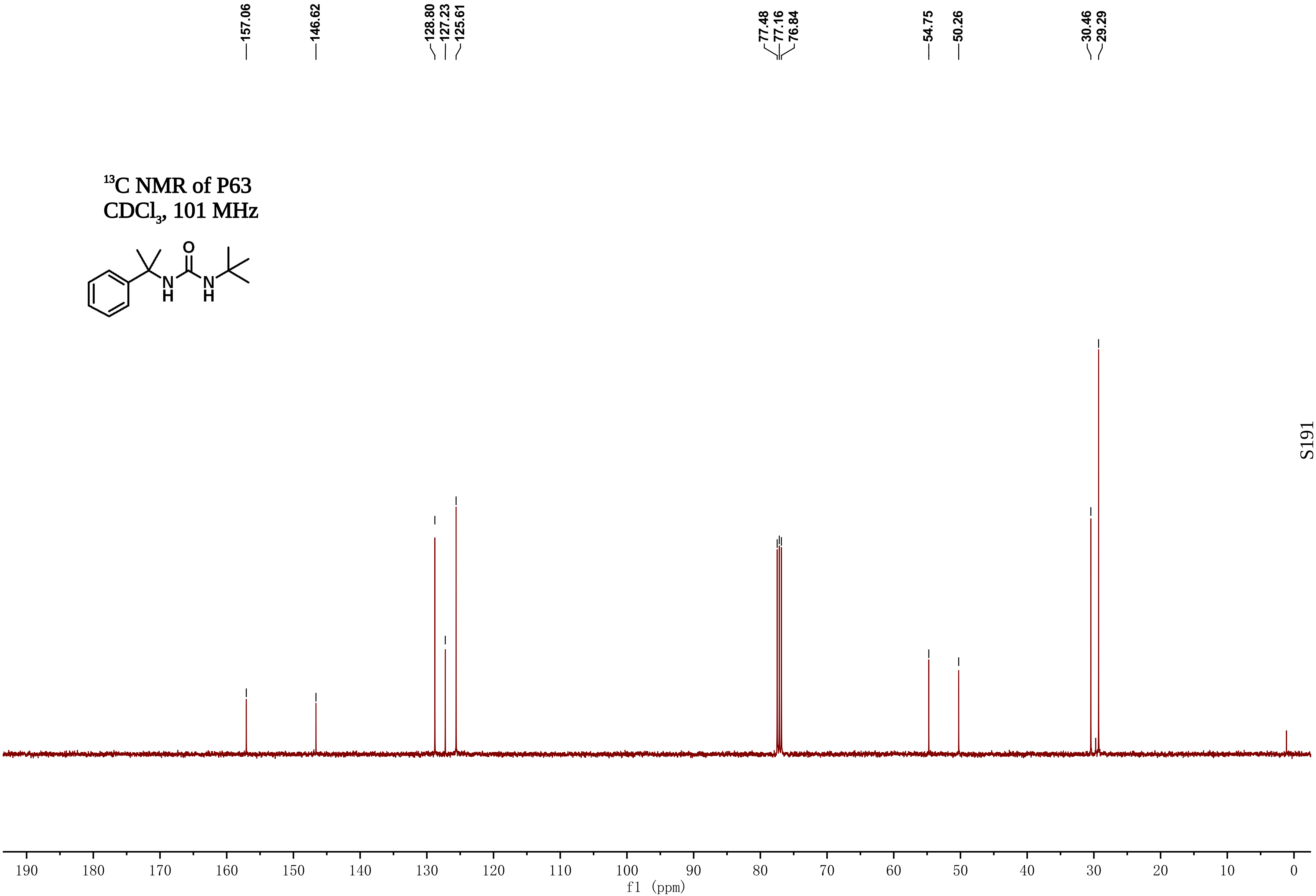

^{1}H NMR of P64
$CDCl_3$, 400 MHz

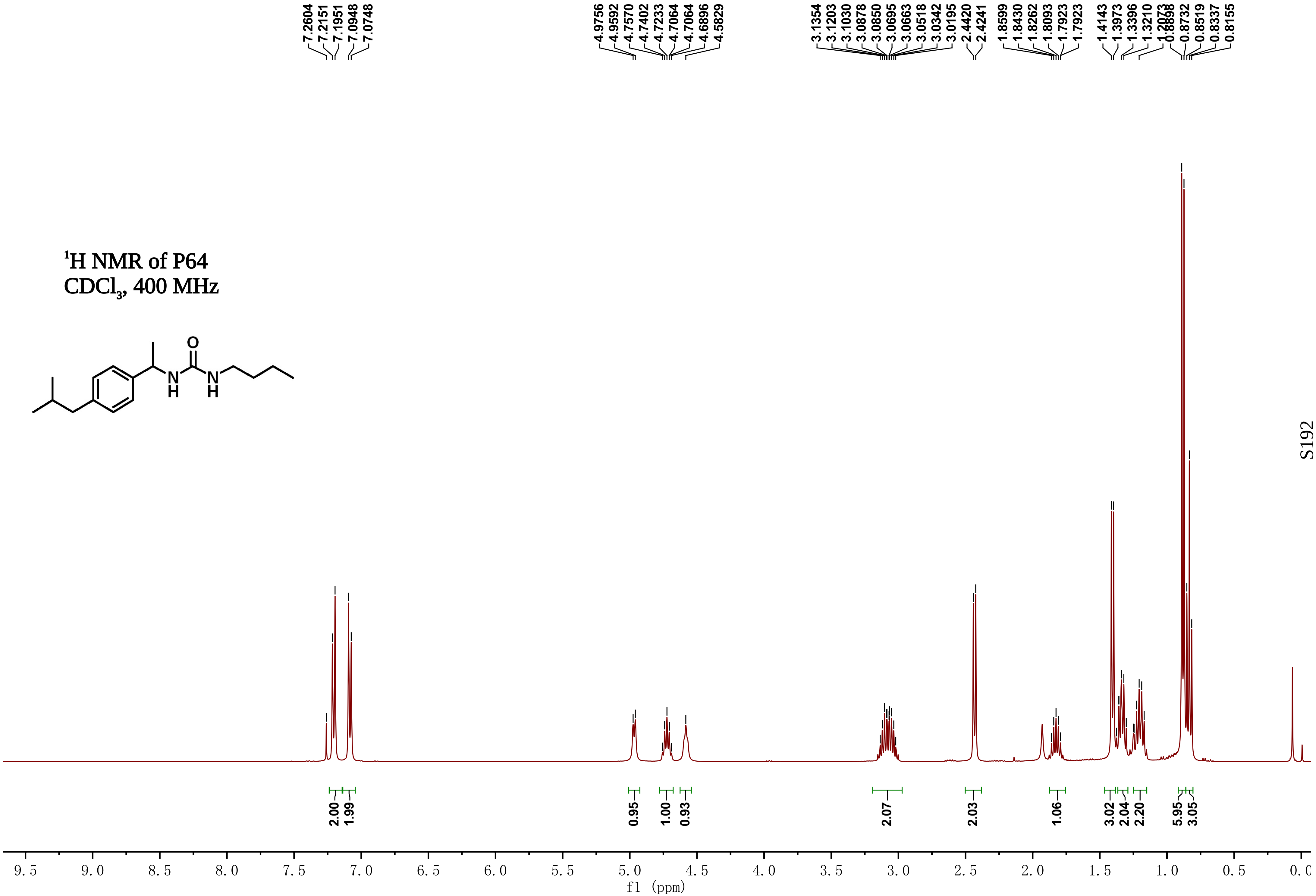

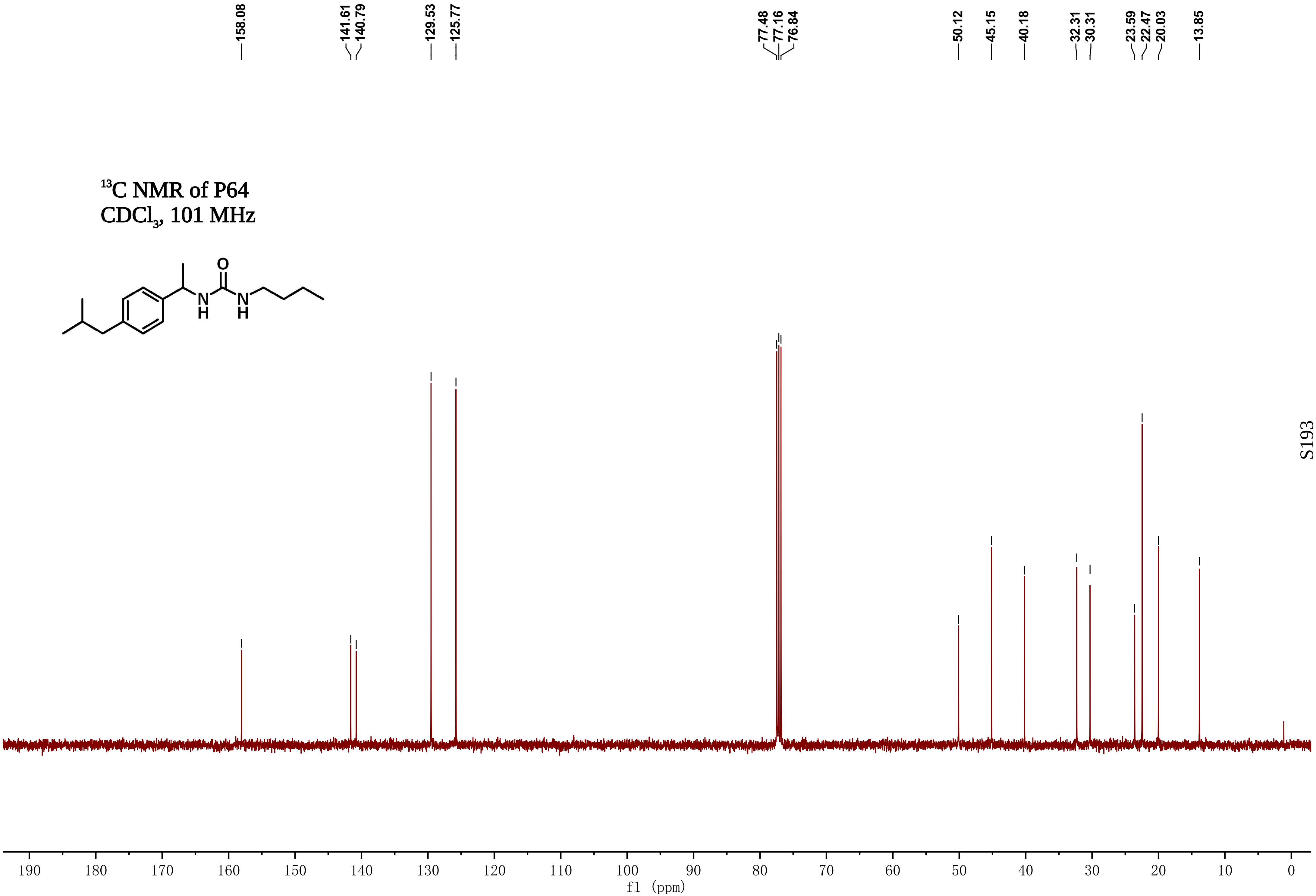

158.08
141.61
140.79
129.53
125.77
77.48
77.16
76.84
50.12
45.15
40.18
32.31
30.31
23.59
22.47
20.03
13.85
^{13}C NMR of P64
$CDCl_3$, 101 MHz
O
N
H
N
H
190 180 170 160 150 140 130 120 110 100 90 80 70 60 50 40 30 20 10 0
f1 (ppm)

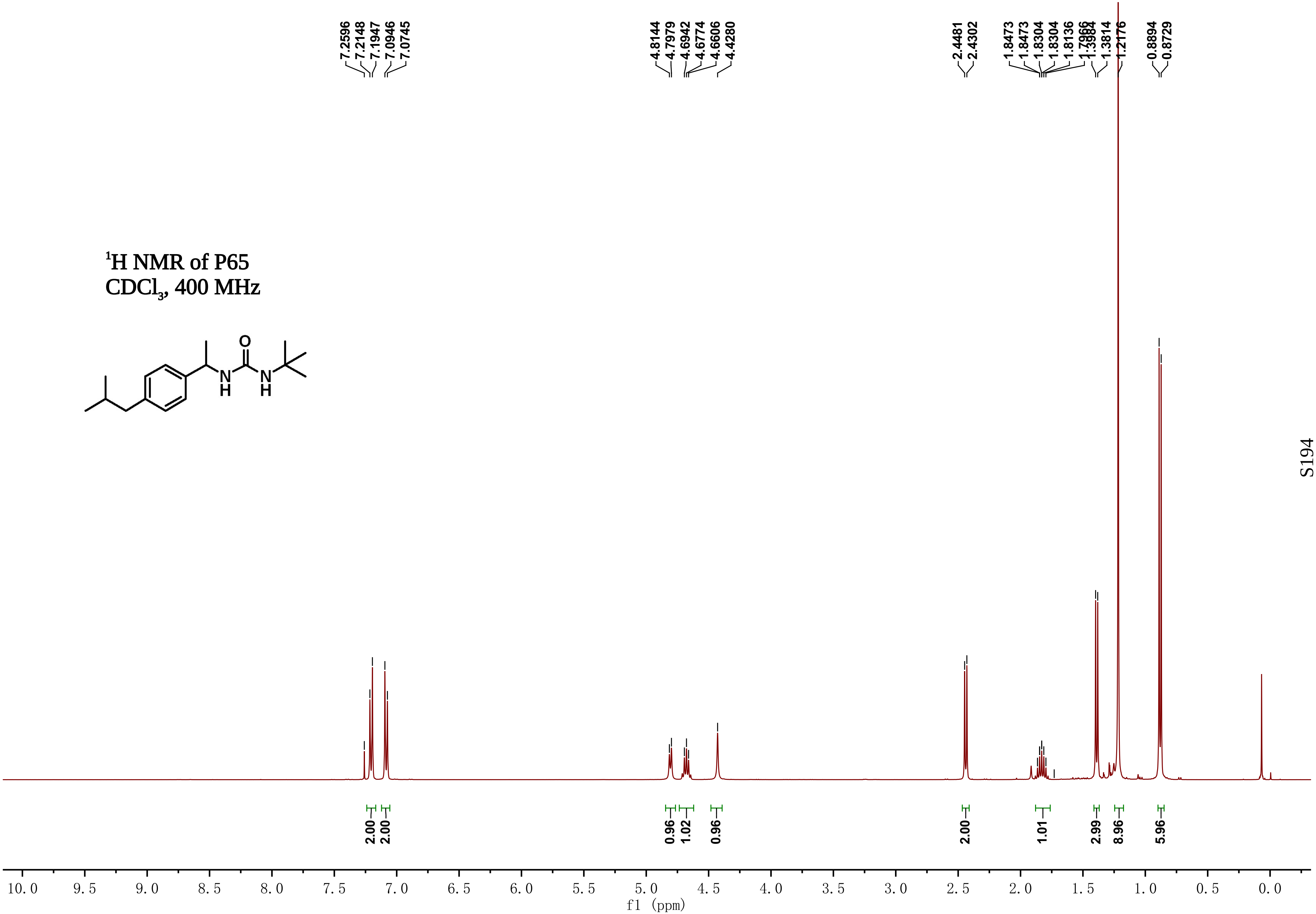

1H NMR of P65
CDCl3, 400 MHz
7.2596
7.2148
7.1947
7.0946
7.0745
4.8144
4.7979
4.6942
4.6774
4.6606
4.4280
2.4481
2.4302
1.8473
1.8473
1.8304
1.8304
1.8136
1.7966
1.3982
1.3814
1.2176
0.8894
0.8729
2.00
2.00
0.96
1.02
0.96
2.00
1.01
2.99
8.96
5.96
O
N
H
N
H
10.0 9.5 9.0 8.5 8.0 7.5 7.0 6.5 6.0 5.5 5.0 4.5 4.0 3.5 3.0 2.5 2.0 1.5 1.0 0.5 0.0
f1 (ppm)

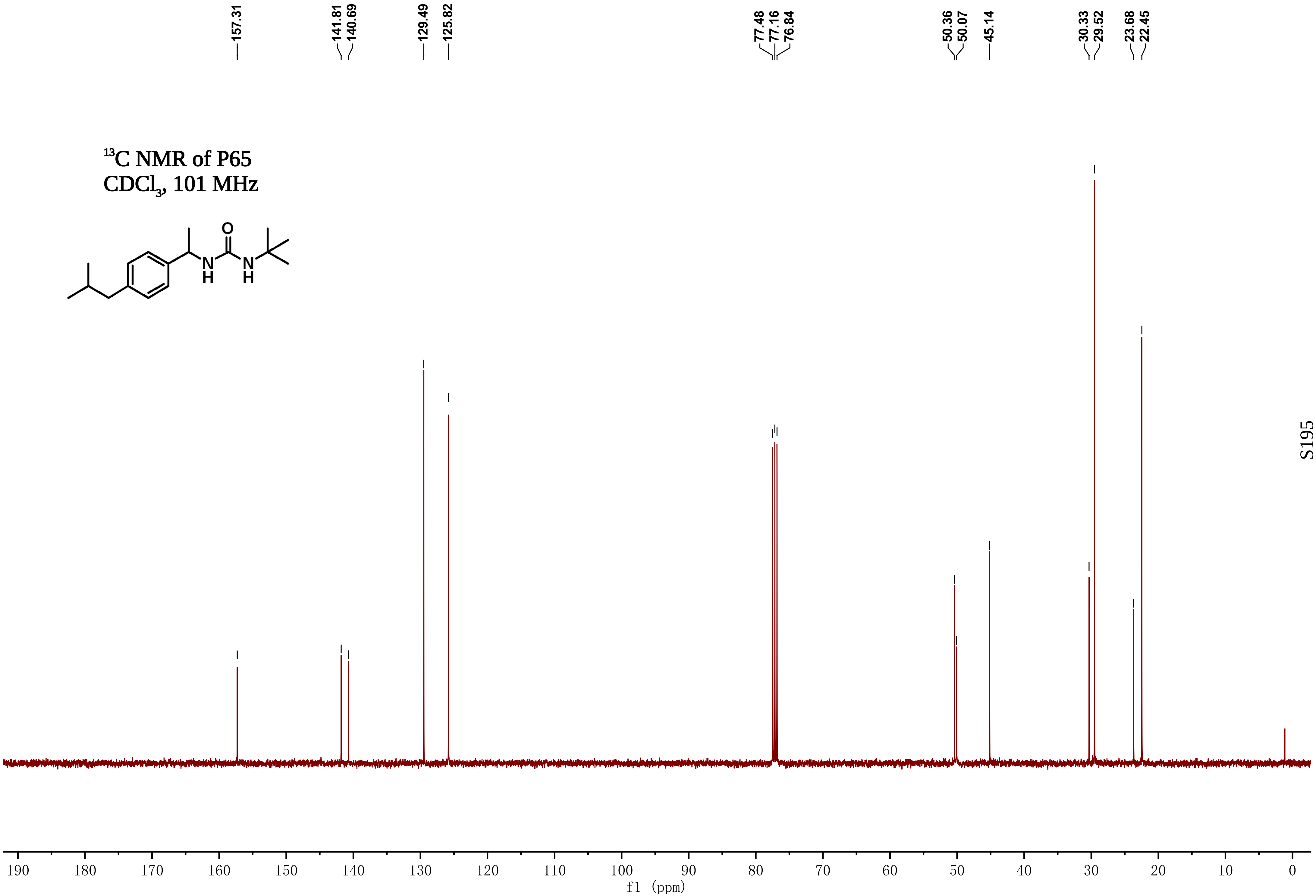
157.31
141.81
140.69
129.49
125.82
77.48
77.16
76.84
50.36
50.07
45.14
30.33
29.52
23.68
22.45
^{13}C NMR of P65
$CDCl_3$, 101 MHz
O
N
H
N
H
190
180
170
160
150
140
130
120
110
100
90
80
70
60
50
40
30
20
10
0
f1 (ppm)

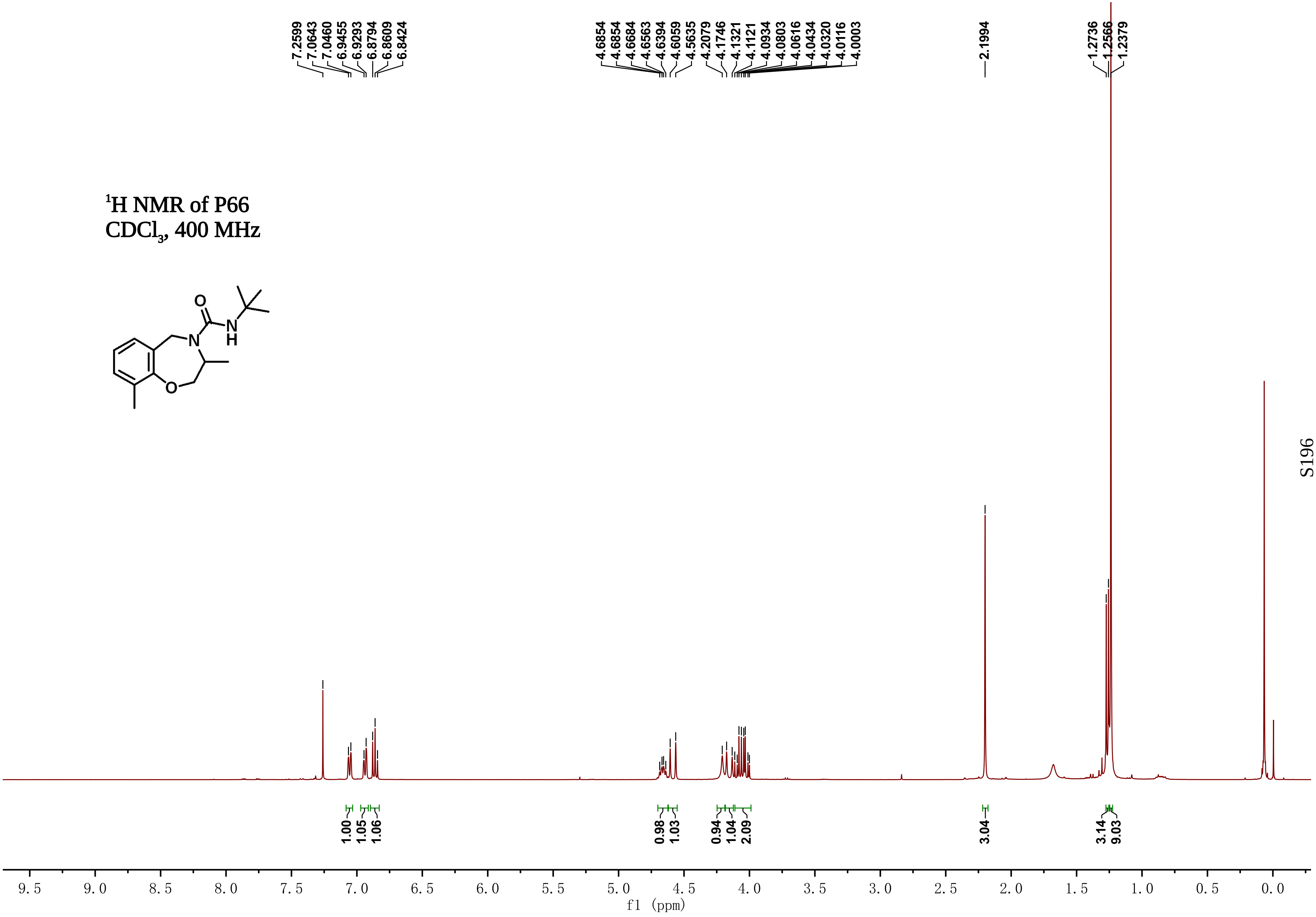

^{1}H NMR of P66
$CDCl_3$, 400 MHz
7.2599
7.0643
7.0460
6.9455
6.9293
6.8794
6.8609
6.8424
4.6854
4.6854
4.6684
4.6563
4.6394
4.6059
4.5635
4.2079
4.1746
4.1321
4.1121
4.0934
4.0803
4.0616
4.0434
4.0320
4.0116
4.0003
2.1994
1.2736
1.2566
1.2379
1.00
1.05
1.06
0.98
1.03
0.94
1.04
2.09
3.04
3.14
9.03
O
N
H
N
O
9.5
9.0
8.5
8.0
7.5
7.0
6.5
6.0
5.5
5.0
4.5
4.0
3.5
3.0
2.5
2.0
1.5
1.0
0.5
0.0
f1 (ppm)

^{13}C NMR of P66
$CDCl_3$, 101 MHz

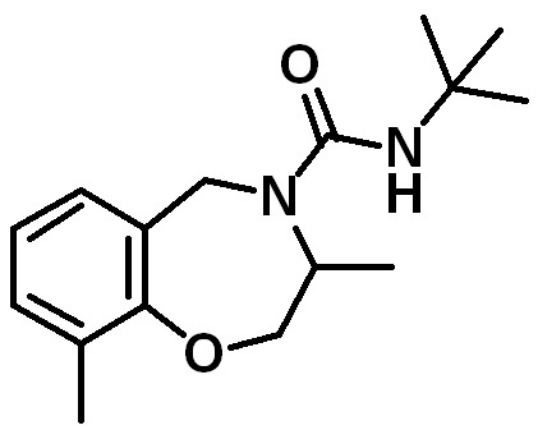

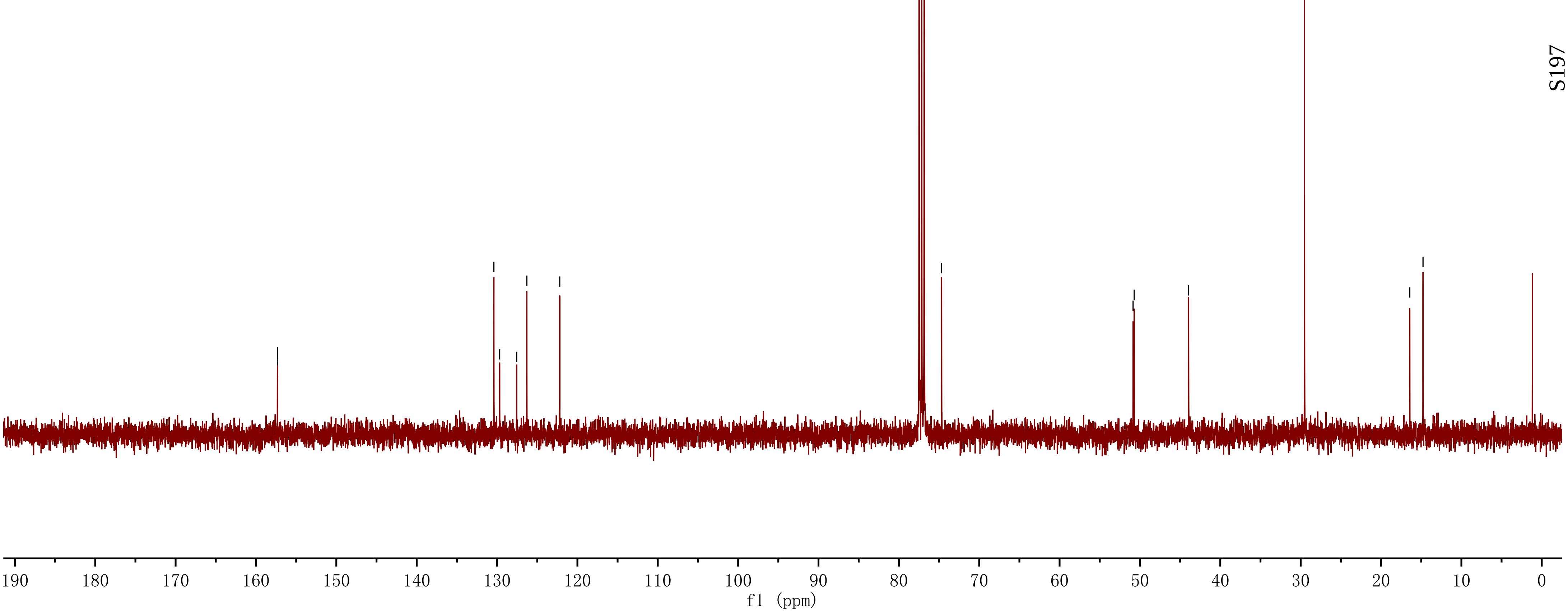

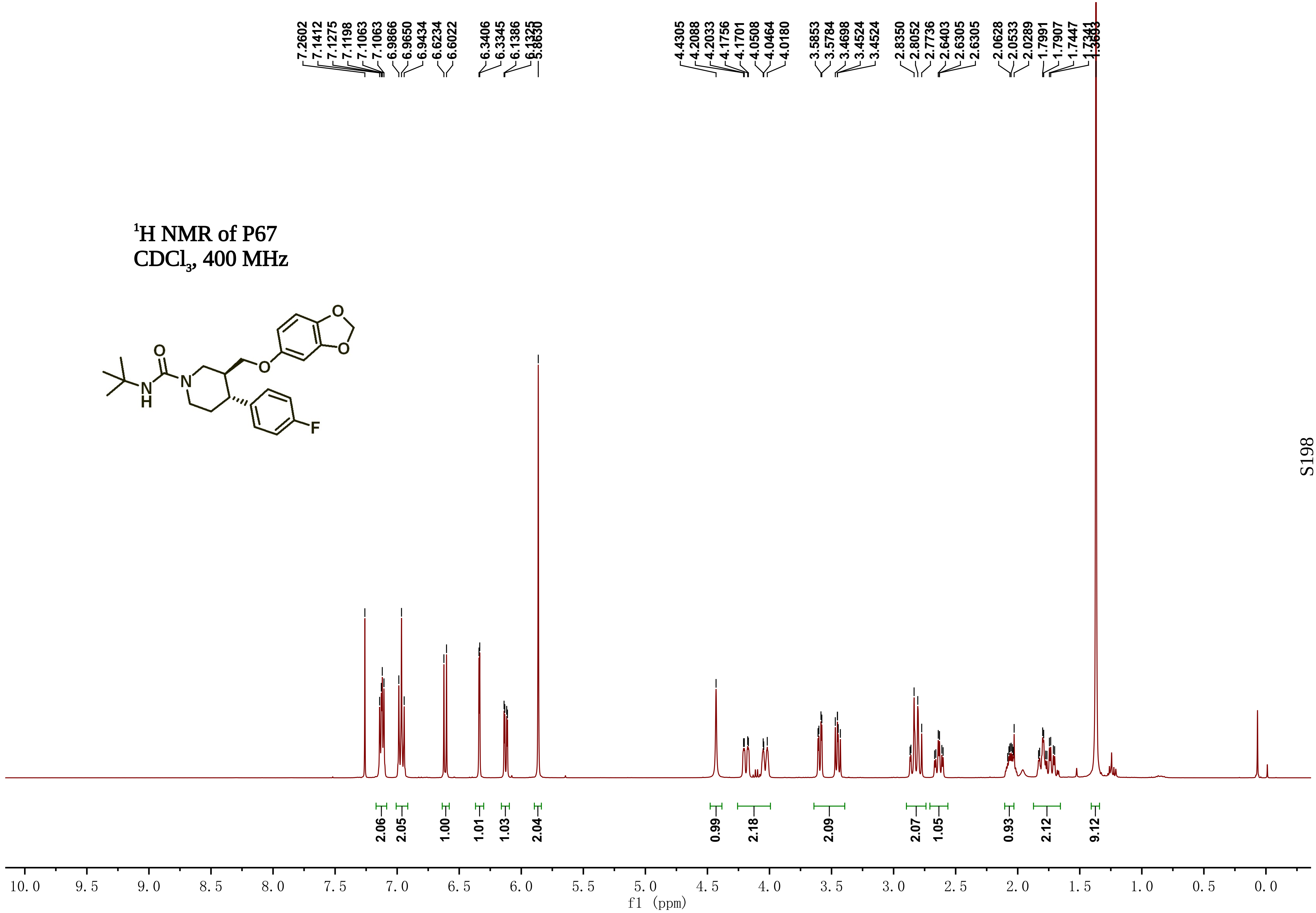

7.2602
7.1412
7.1275
7.1198
7.1063
7.1063
6.9866
6.9650
6.9434
6.6234
6.6022
6.3406
6.3345
6.1386
6.1325
5.8630
4.4305
4.2088
4.2033
4.1756
4.1701
4.0508
4.0464
4.0180
3.5853
3.5784
3.4698
3.4524
3.4524
2.8350
2.8052
2.7736
2.6403
2.6305
2.6305
2.0628
2.0533
2.0289
1.7991
1.7907
1.7447
¹H NMR of P67
CDCl₃, 400 MHz
O
N
H
F
2.06
2.05
1.00
1.01
1.03
2.04
0.99
2.18
2.09
2.07
1.05
0.93
2.12
9.12
10.0 9.5 9.0 8.5 8.0 7.5 7.0 6.5 6.0 5.5 5.0 4.5 4.0 3.5 3.0 2.5 2.0 1.5 1.0 0.5 0.0
f1 (ppm)

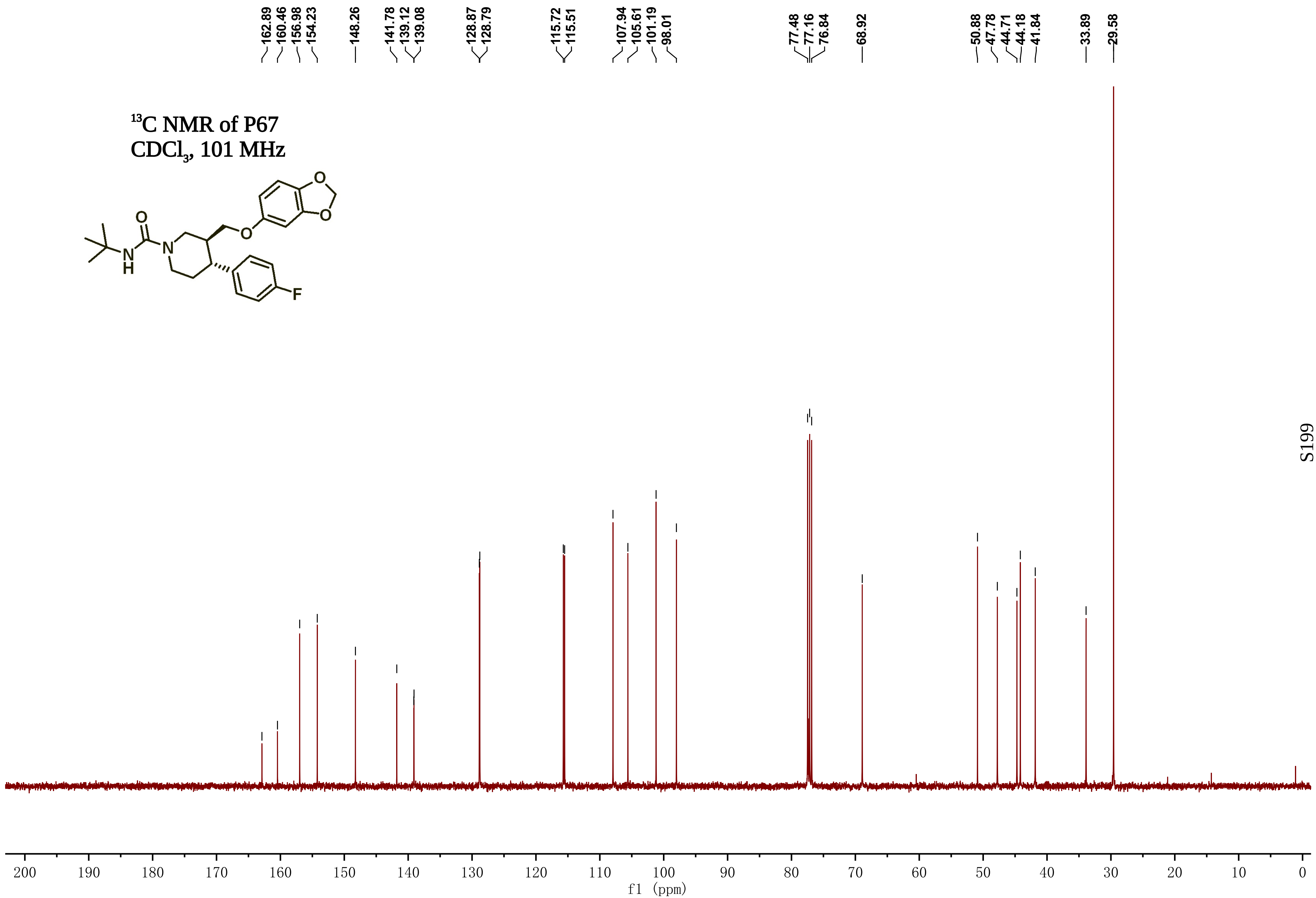
162.89
160.46
156.98
154.23
148.26
141.78
139.12
139.08
128.87
128.79
115.72
115.51
107.94
105.61
101.19
98.01
77.48
77.16
76.84
68.92
50.88
47.78
44.71
44.18
41.84
33.89
29.58
13C NMR of P67
CDCl3, 101 MHz
O
N
H
N
O
O
O
F
200 190 180 170 160 150 140 130 120 110 100 90 80 70 60 50 40 30 20 10 0
f1 (ppm)

^{19}F NMR of P67
$CDCl_3$, 376 MHz

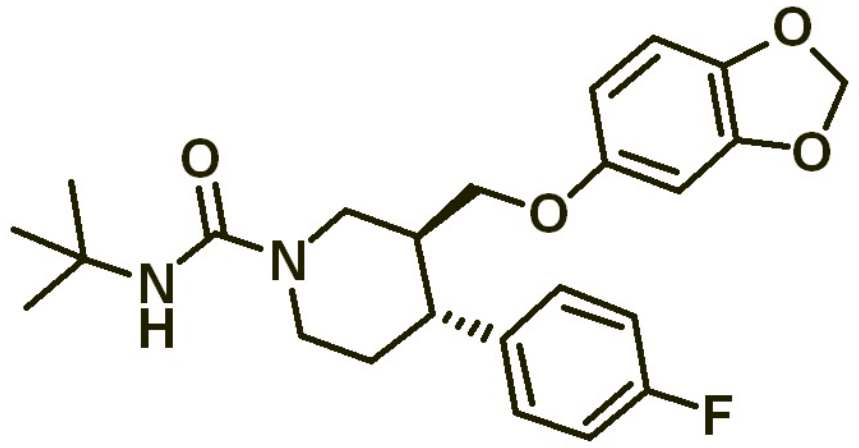

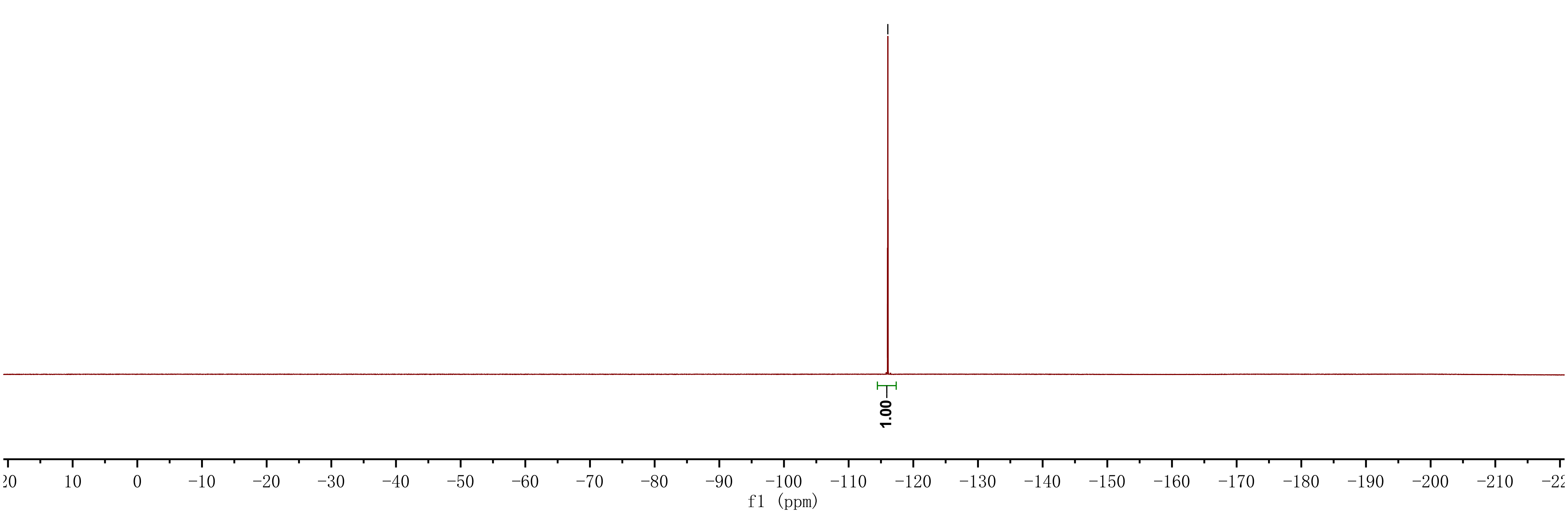

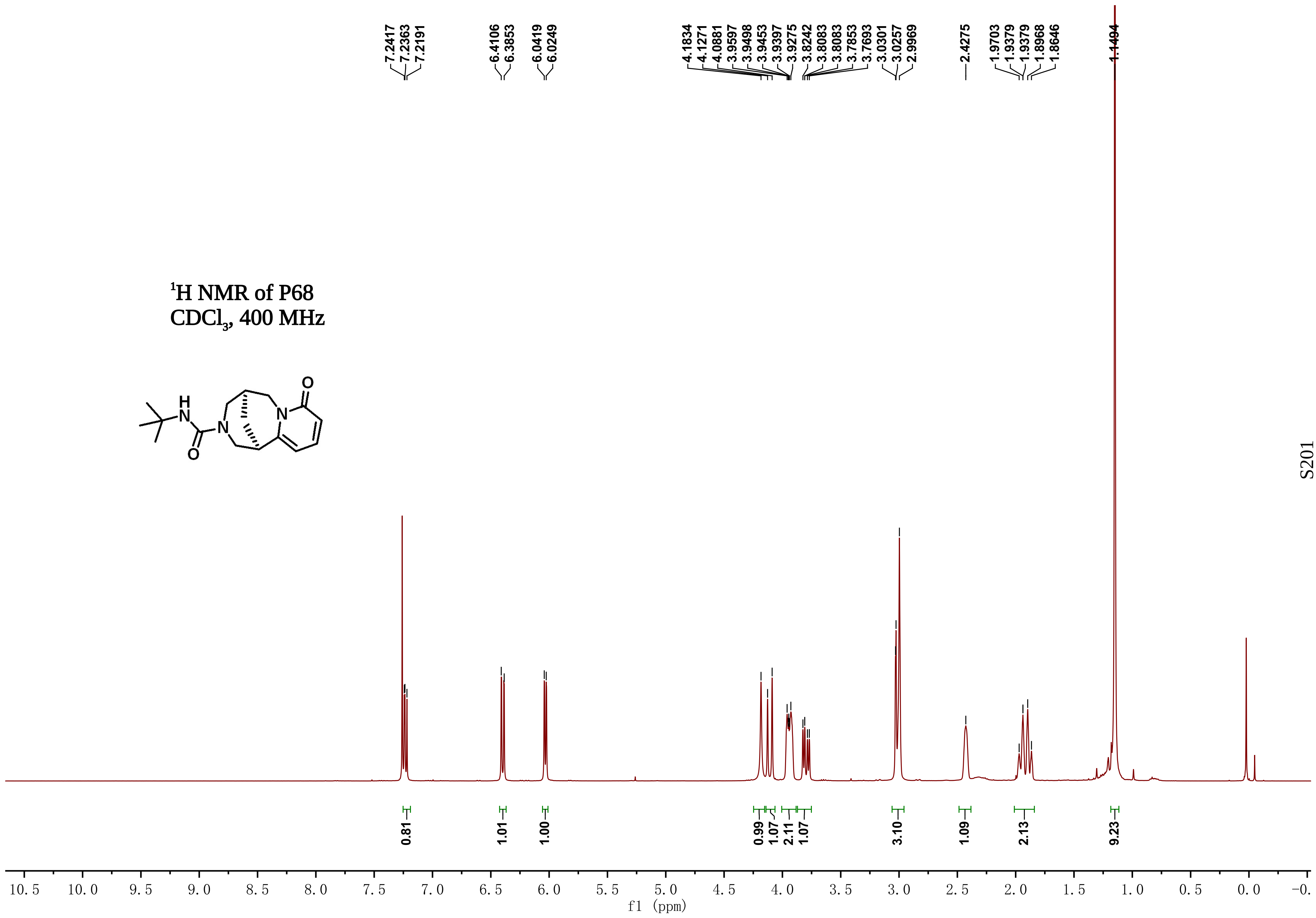

7.2417
7.2363
7.2191
6.4106
6.3853
6.0419
6.0249
4.1834
4.1271
4.0881
3.9597
3.9498
3.9453
3.9397
3.9275
3.8242
3.8083
3.8083
3.7853
3.7693
3.0301
3.0257
2.9969
2.4275
1.9703
1.9379
1.9379
1.8968
1.8646
1.1494
1H NMR of P68
CDCl3, 400 MHz
0.81
1.01
1.00
0.99
1.07
2.11
1.07
3.10
1.09
2.13
9.23
10.5 10.0 9.5 9.0 8.5 8.0 7.5 7.0 6.5 6.0 5.5 5.0 4.5 4.0 3.5 3.0 2.5 2.0 1.5 1.0 0.5 0.0 -0.
f1 (ppm)

^{13}C NMR of P68
$CDCl_3$, 101 MHz

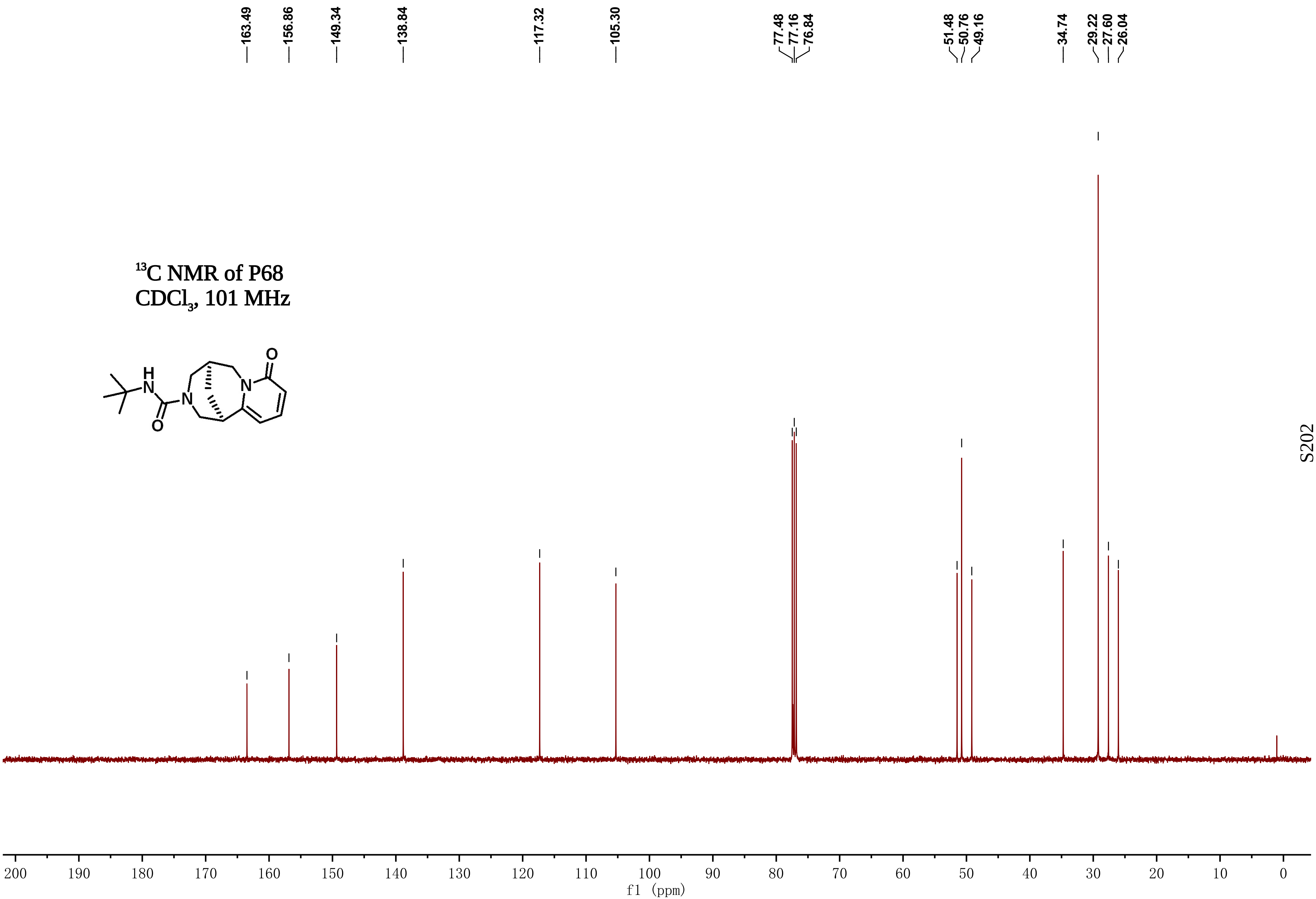

^{1}H NMR of P69
$CDCl_3$, 400 MHz

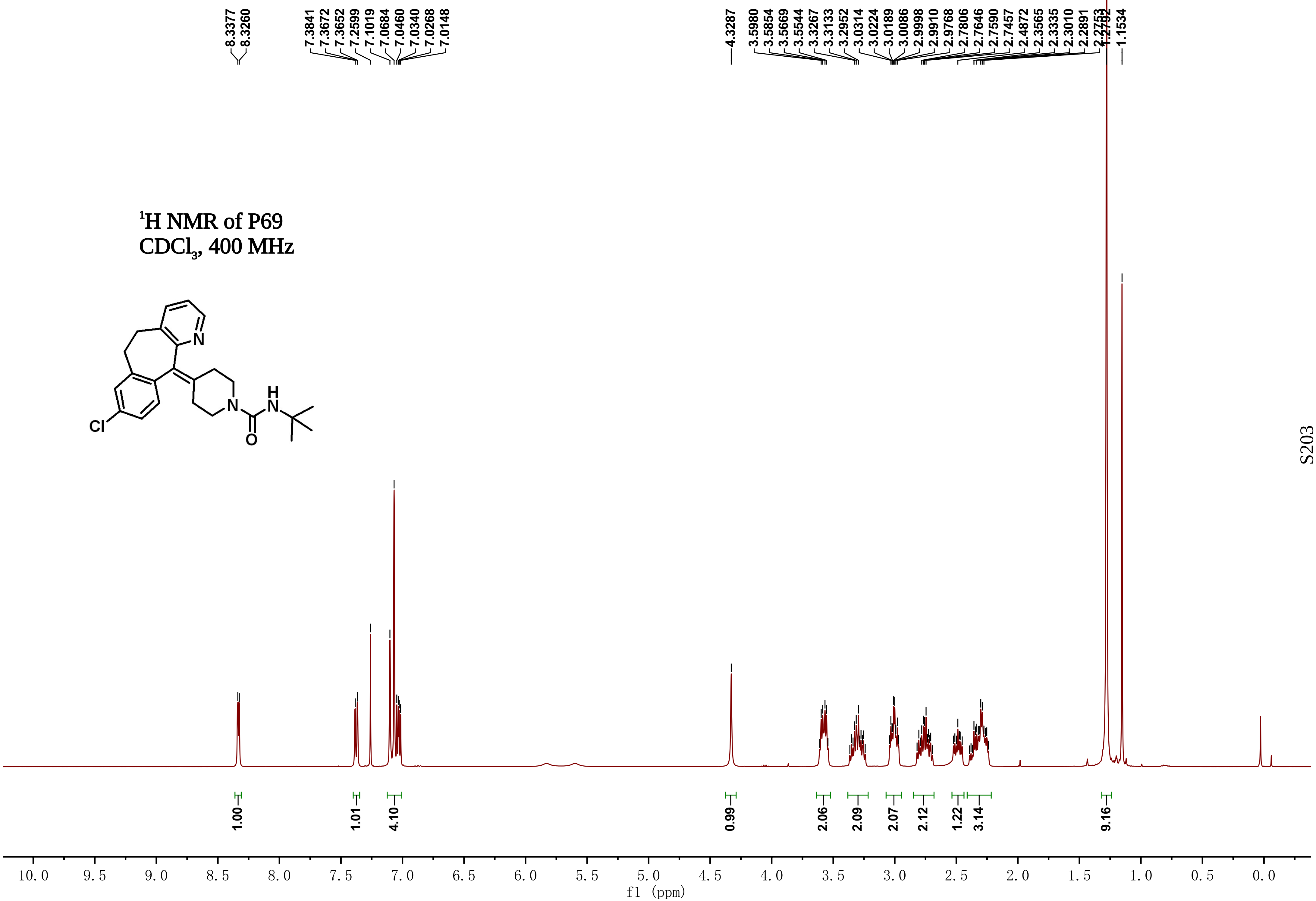

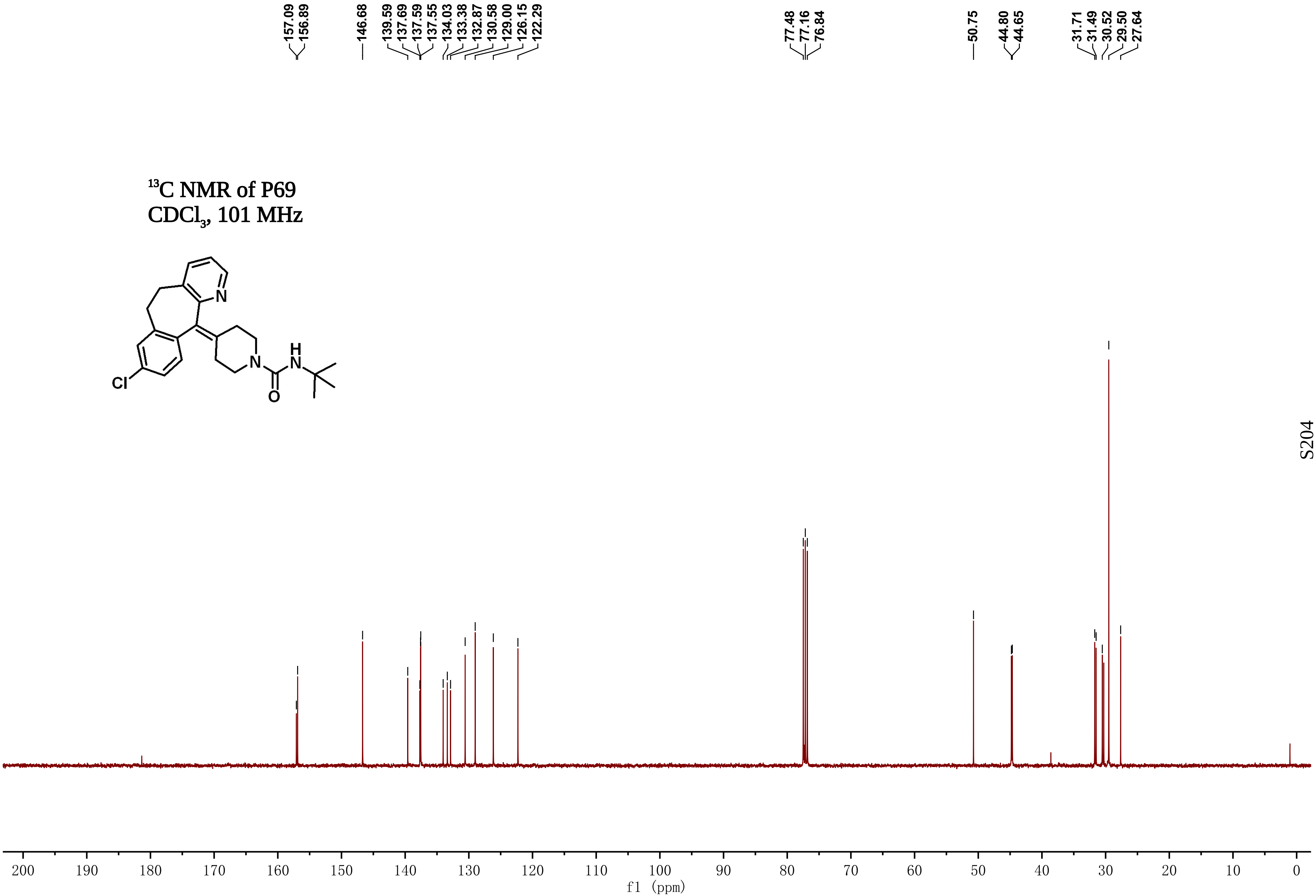
157.09
156.89
146.68
139.59
137.69
137.59
137.55
134.03
133.38
132.87
130.58
129.00
126.15
122.29
77.48
77.16
76.84
50.75
44.80
44.65
31.71
31.49
30.52
29.50
27.64
13C NMR of P69
CDCl3, 101 MHz
N
H
N
N
O
Cl
200 190 180 170 160 150 140 130 120 110 100 90 80 70 60 50 40 30 20 10 0
f1 (ppm)

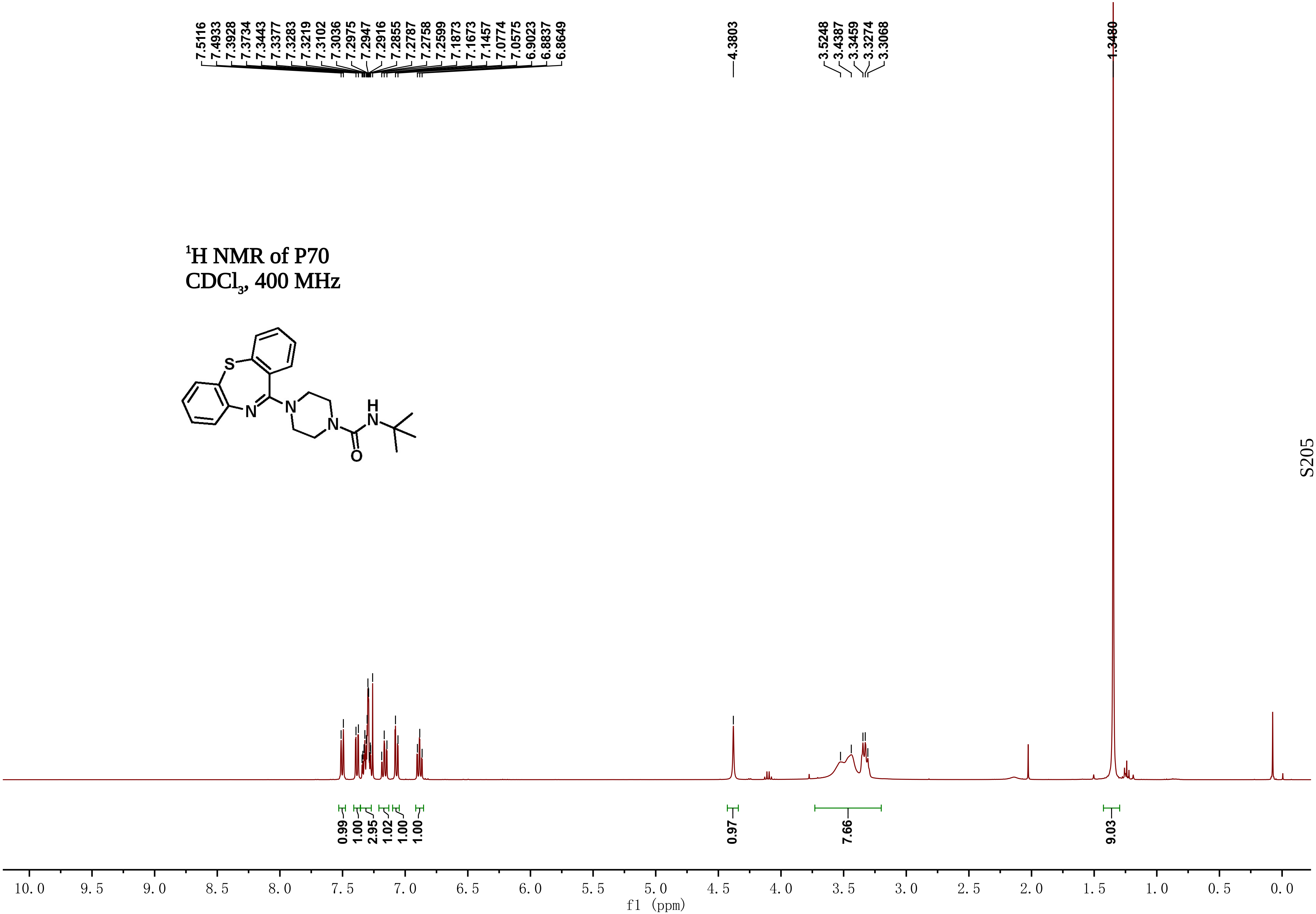

^{1}H NMR of P70
$CDCl_3$, 400 MHz

^{13}C NMR of P70
$CDCl_3$, 101 MHz

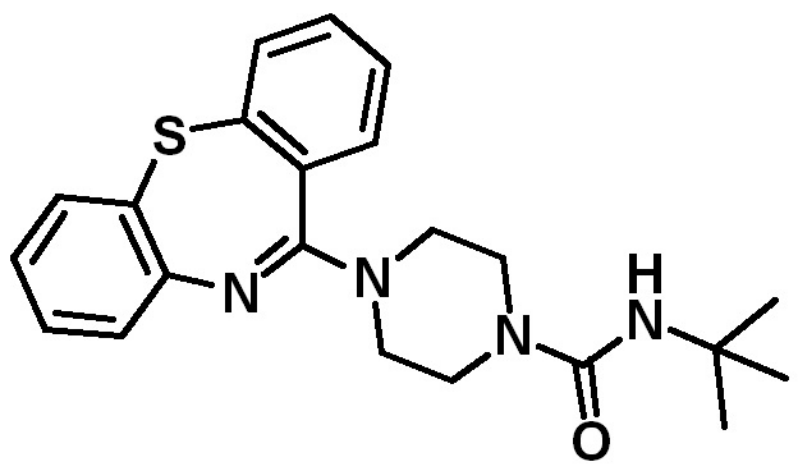

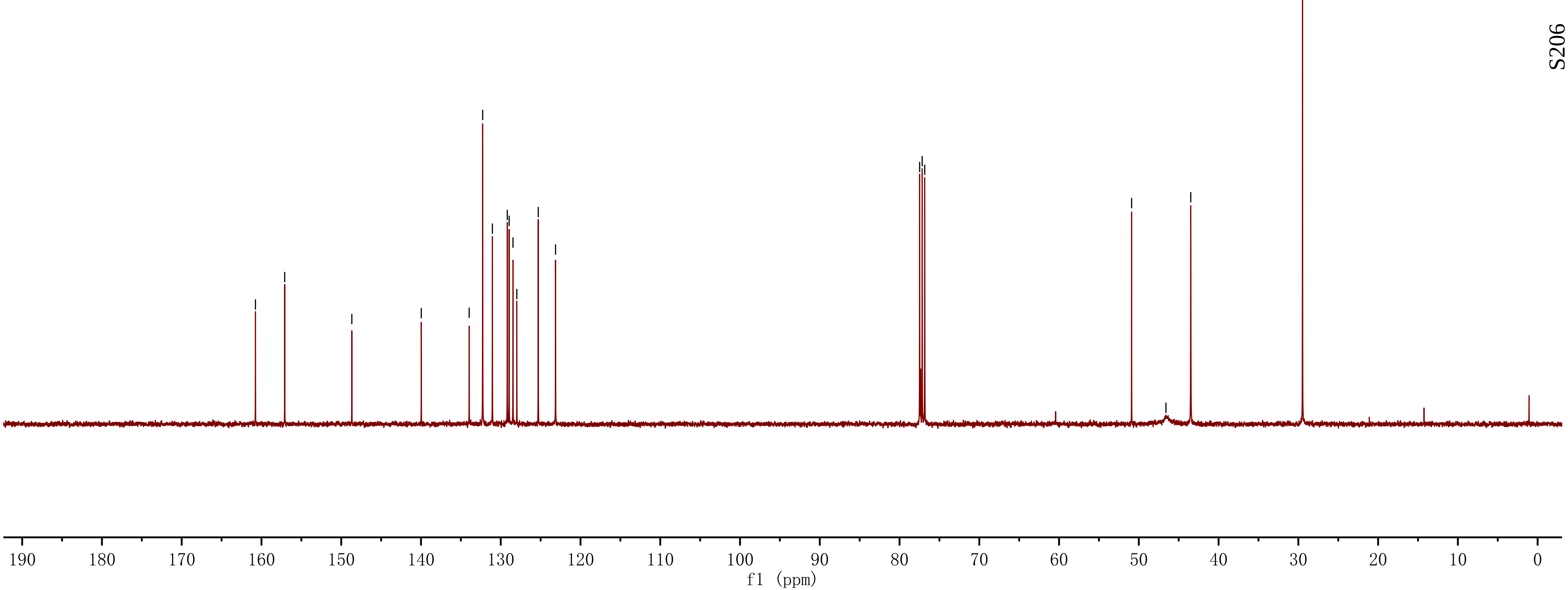

S206

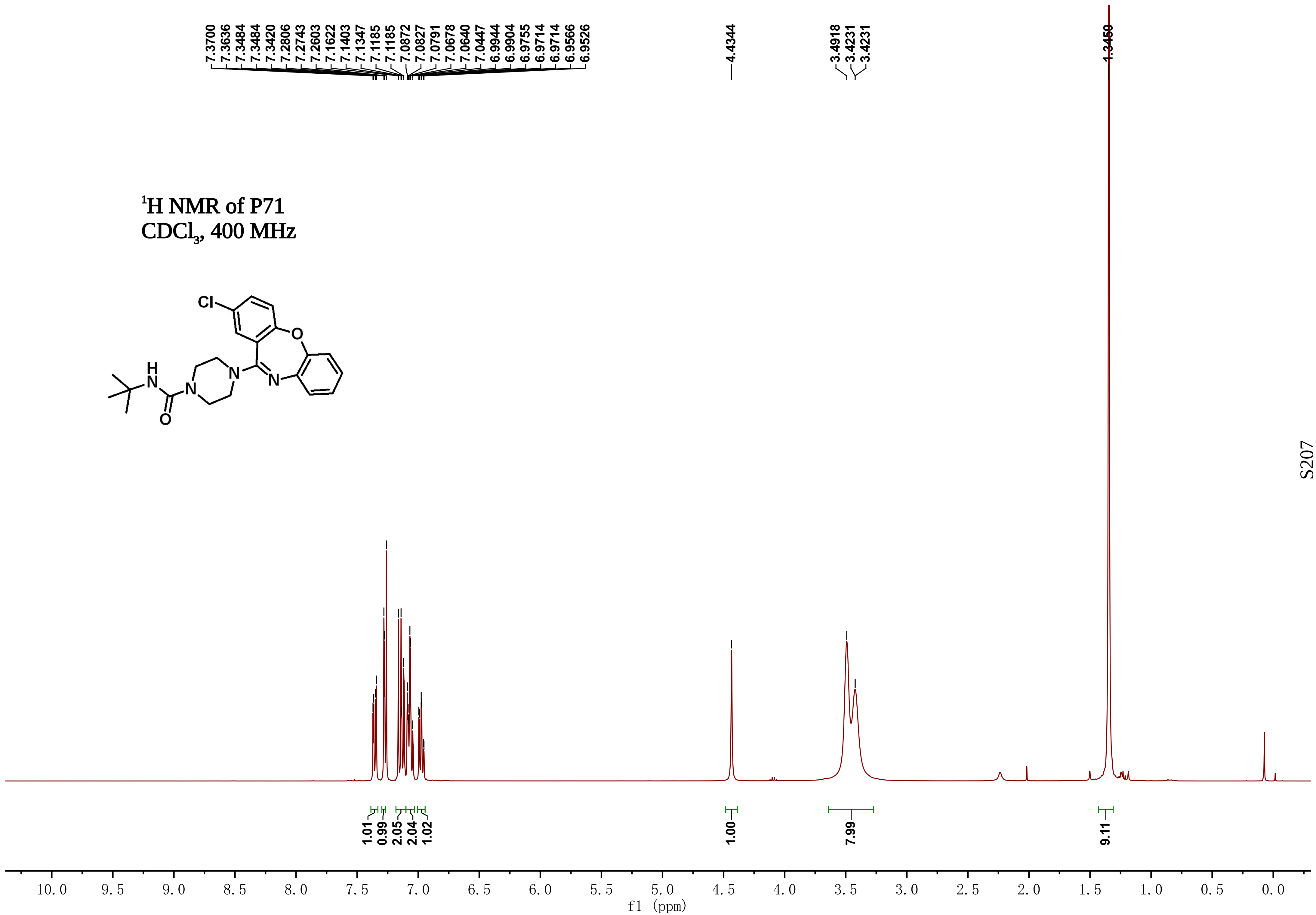

7.3700
7.3636
7.3484
7.3484
7.3420
7.2806
7.2743
7.2603
7.1622
7.1403
7.1347
7.1185
7.1185
7.0872
7.0827
7.0791
7.0678
7.0640
7.0447
6.9944
6.9904
6.9755
6.9714
6.9714
6.9566
6.9526
4.4344
3.4918
3.4231
3.4231
1.3459
¹H NMR of P71
CDCl₃, 400 MHz
Cl
O
N
N
H
N
N
O
1.01
0.99
2.05
2.04
1.02
1.00
7.99
9.11
10.0
9.5
9.0
8.5
8.0
7.5
7.0
6.5
6.0
5.5
5.0
4.5
4.0
3.5
3.0
2.5
2.0
1.5
1.0
0.5
0.0
f1 (ppm)

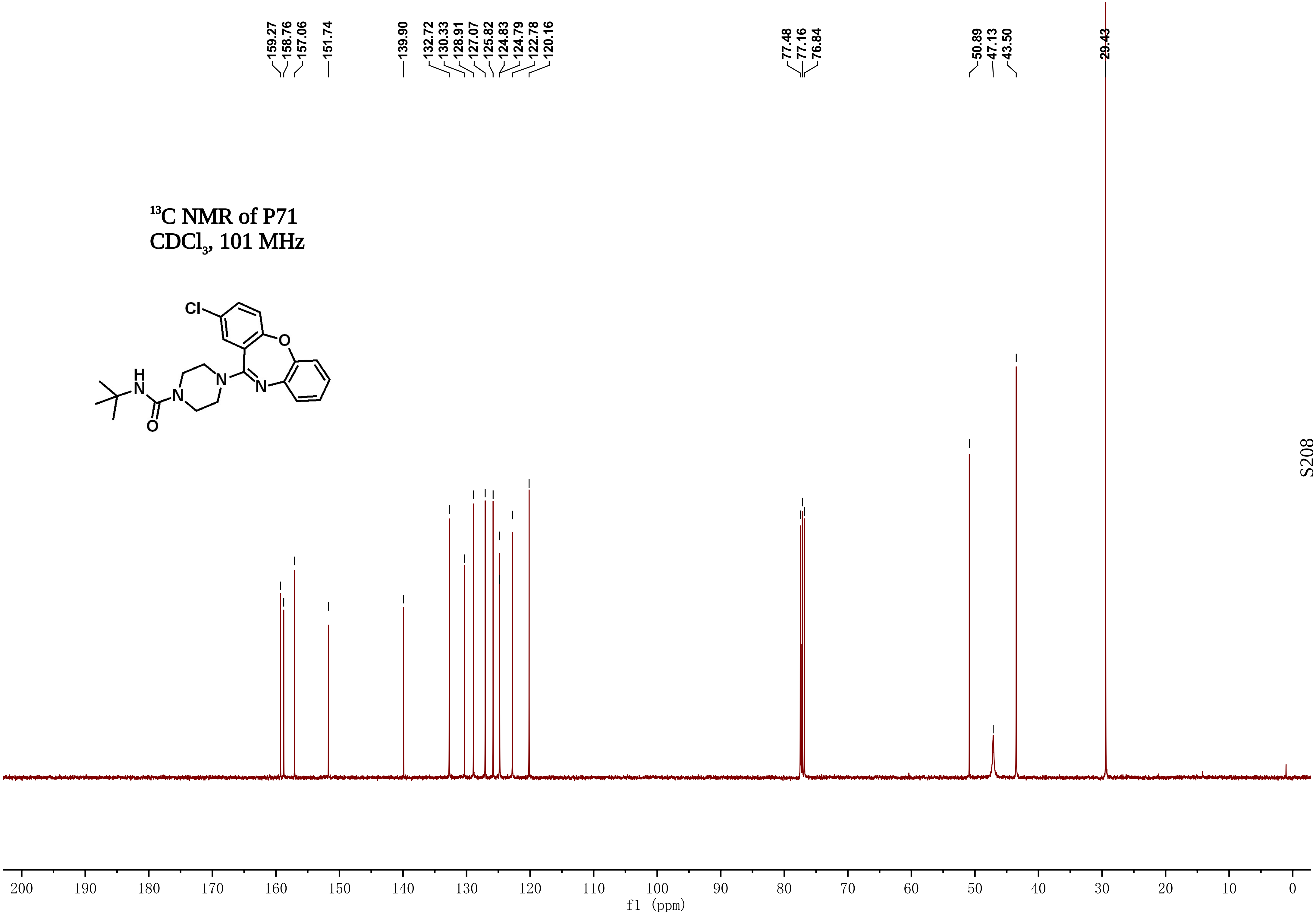
159.27
158.76
157.06
151.74
139.90
132.72
130.33
128.91
127.07
125.82
124.83
124.79
122.78
120.16
77.48
77.16
76.84
50.89
47.13
43.50
29.43
^{13}C NMR of P71
$CDCl_3$, 101 MHz
Cl
O
N
N
H
N
N
O
200 190 180 170 160 150 140 130 120 110 100 90 80 70 60 50 40 30 20 10 0
f1 (ppm)

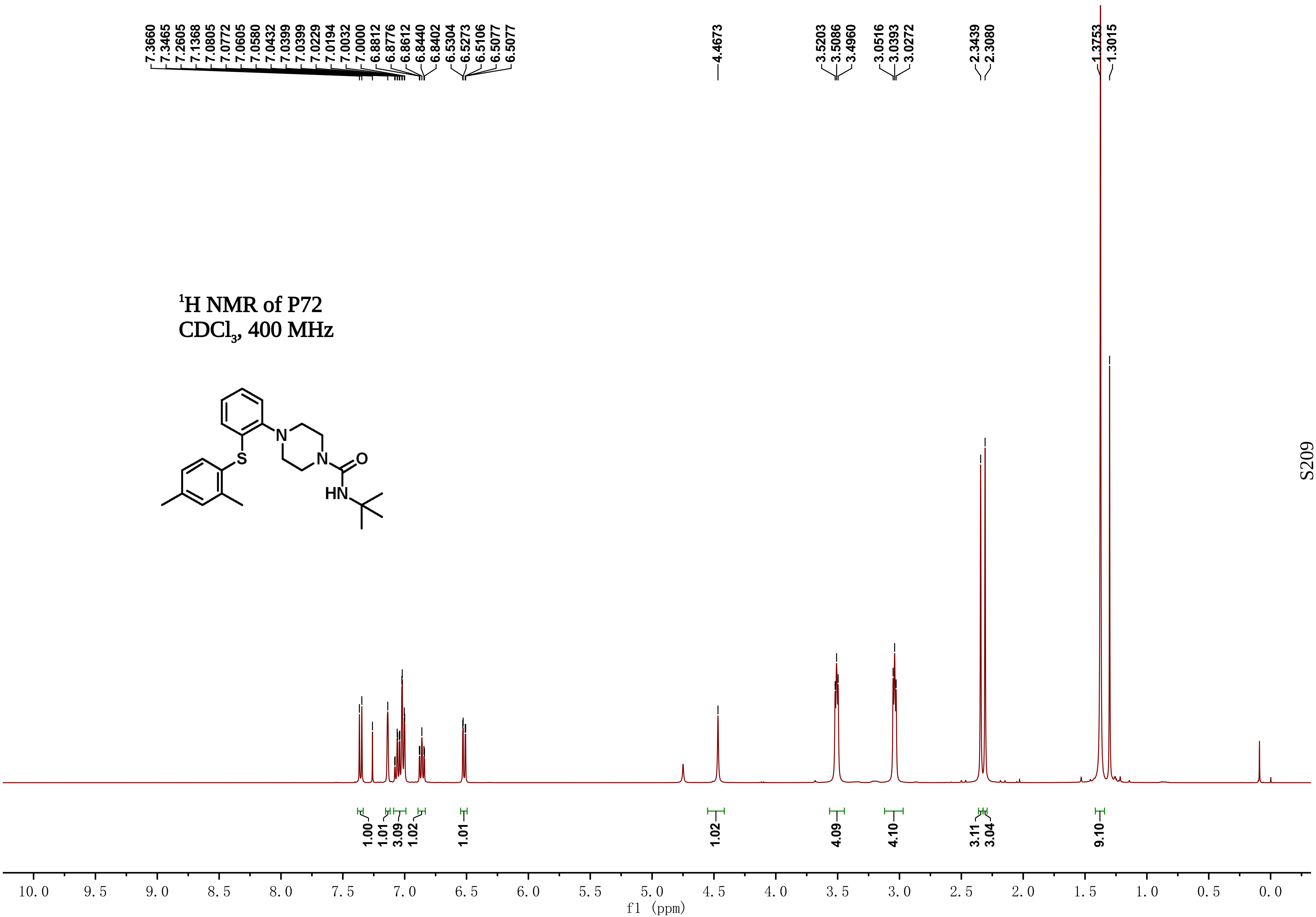

^{1}H NMR of P72
CDCl3, 400 MHz
7.3660
7.3465
7.2605
7.1368
7.0805
7.0772
7.0605
7.0580
7.0432
7.0399
7.0399
7.0229
7.0194
7.0032
7.0000
6.8812
6.8776
6.8612
6.8440
6.8402
6.5304
6.5273
6.5106
6.5077
6.5077
4.4673
3.5203
3.5086
3.4960
3.0516
3.0393
3.0272
2.3439
2.3080
1.3753
1.3015
1.00
1.01
3.09
1.02
1.01
1.02
4.09
4.10
3.11
3.04
9.10
S
N
N
O
HN
10.0
9.5
9.0
8.5
8.0
7.5
7.0
6.5
6.0
5.5
5.0
4.5
4.0
3.5
3.0
2.5
2.0
1.5
1.0
0.5
0.0
f1 (ppm)

^{13}C NMR of P72
$CDCl_3$, 101 MHz

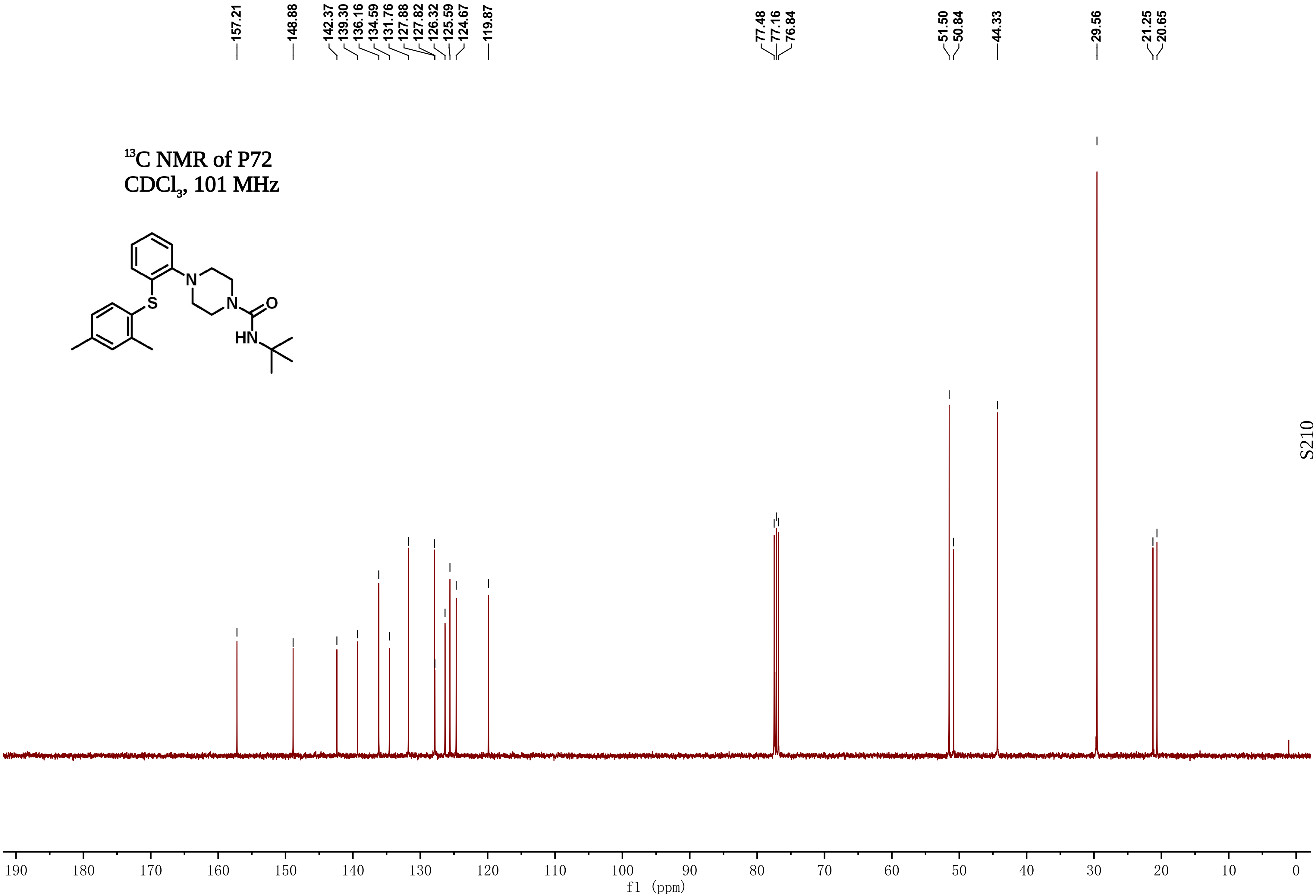

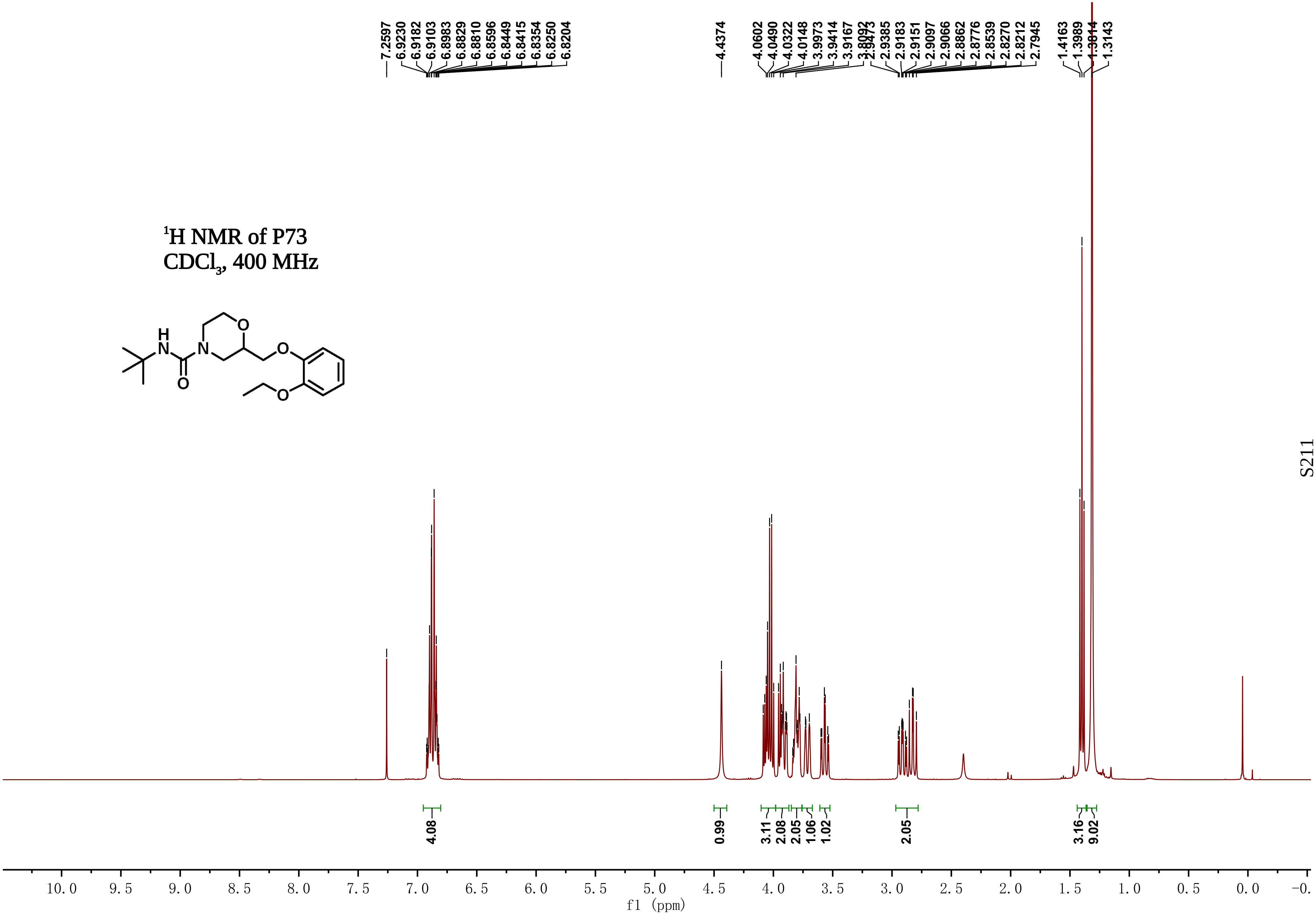

1H NMR of P73
CDCl3, 400 MHz
7.2597
6.9230
6.9182
6.9103
6.8983
6.8829
6.8810
6.8596
6.8449
6.8415
6.8354
6.8250
6.8204
4.4374
4.0602
4.0490
4.0322
4.0148
3.9973
3.9414
3.9167
2.9385
2.9183
2.9151
2.9097
2.9066
2.8862
2.8776
2.8539
2.8270
2.8212
2.7945
1.4163
1.3989
1.3814
1.3143
4.08
0.99
3.11
2.08
2.05
1.06
1.02
2.05
3.16
9.02
f1 (ppm)

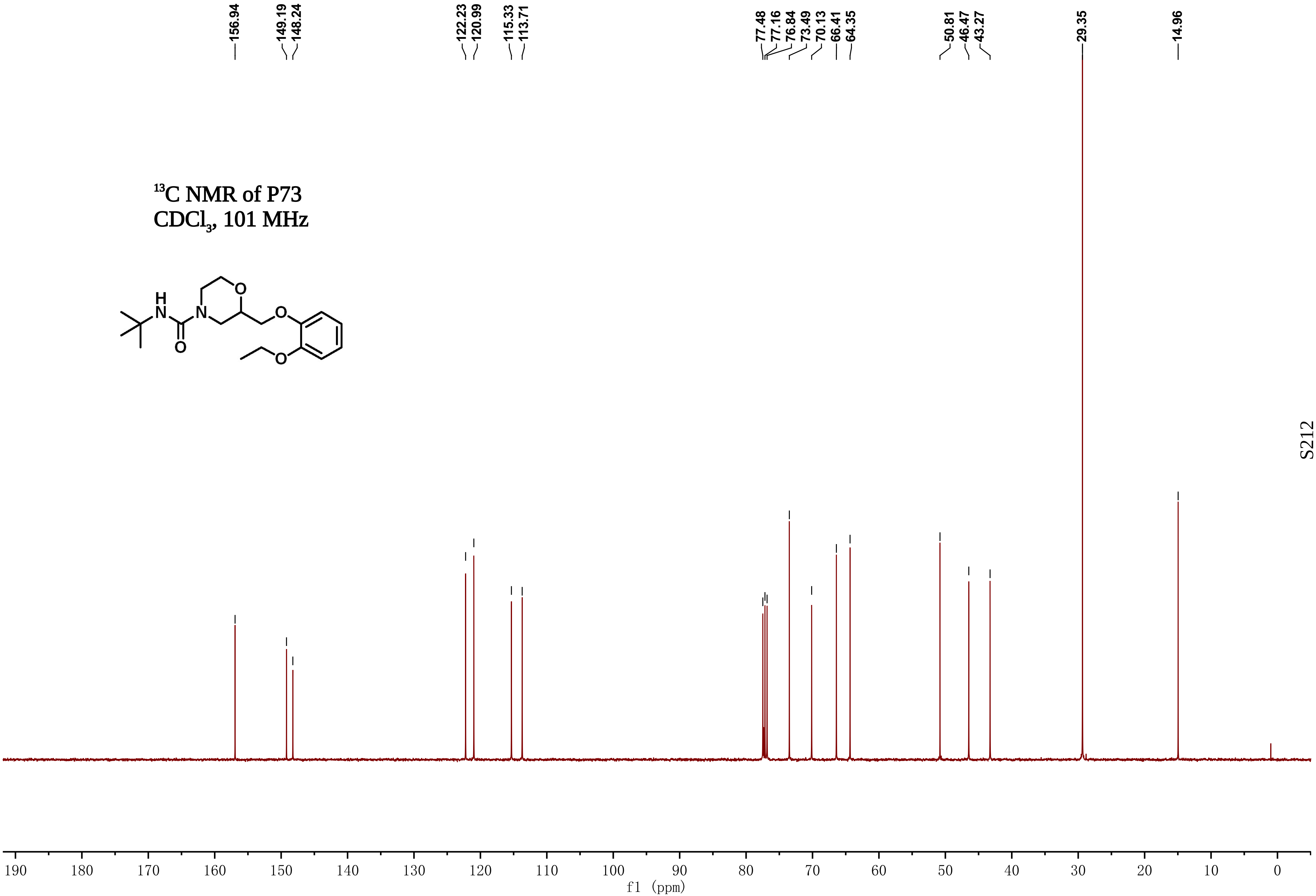

156.94
149.19
148.24
122.23
120.99
115.33
113.71
77.48
77.16
76.84
73.49
70.13
66.41
64.35
50.81
46.47
43.27
29.35
14.96
13C NMR of P73
CDCl3, 101 MHz
190 180 170 160 150 140 130 120 110 100 90 80 70 60 50 40 30 20 10 0
f1 (ppm)

^{1}H NMR of P74
CDCl$_3$, 400 MHz

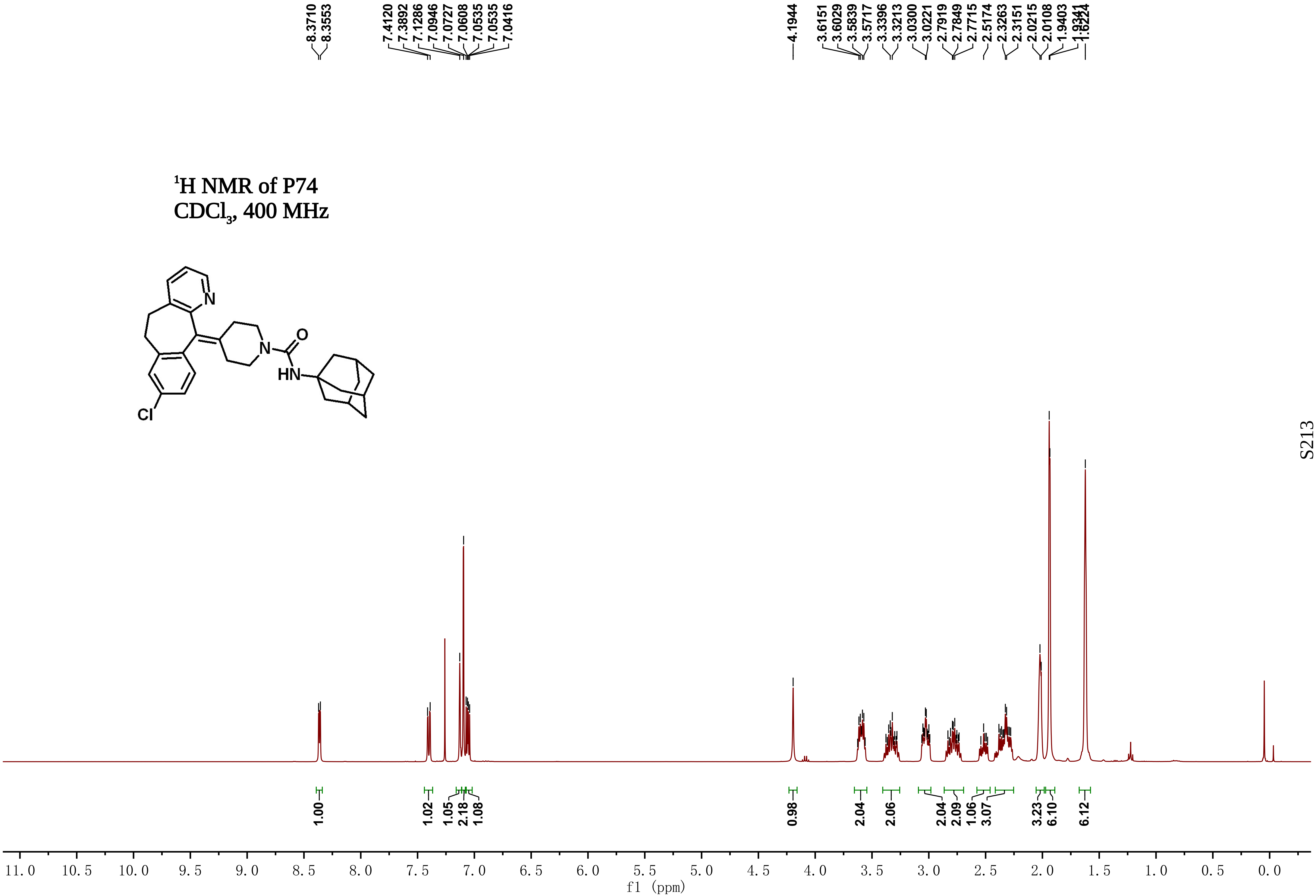

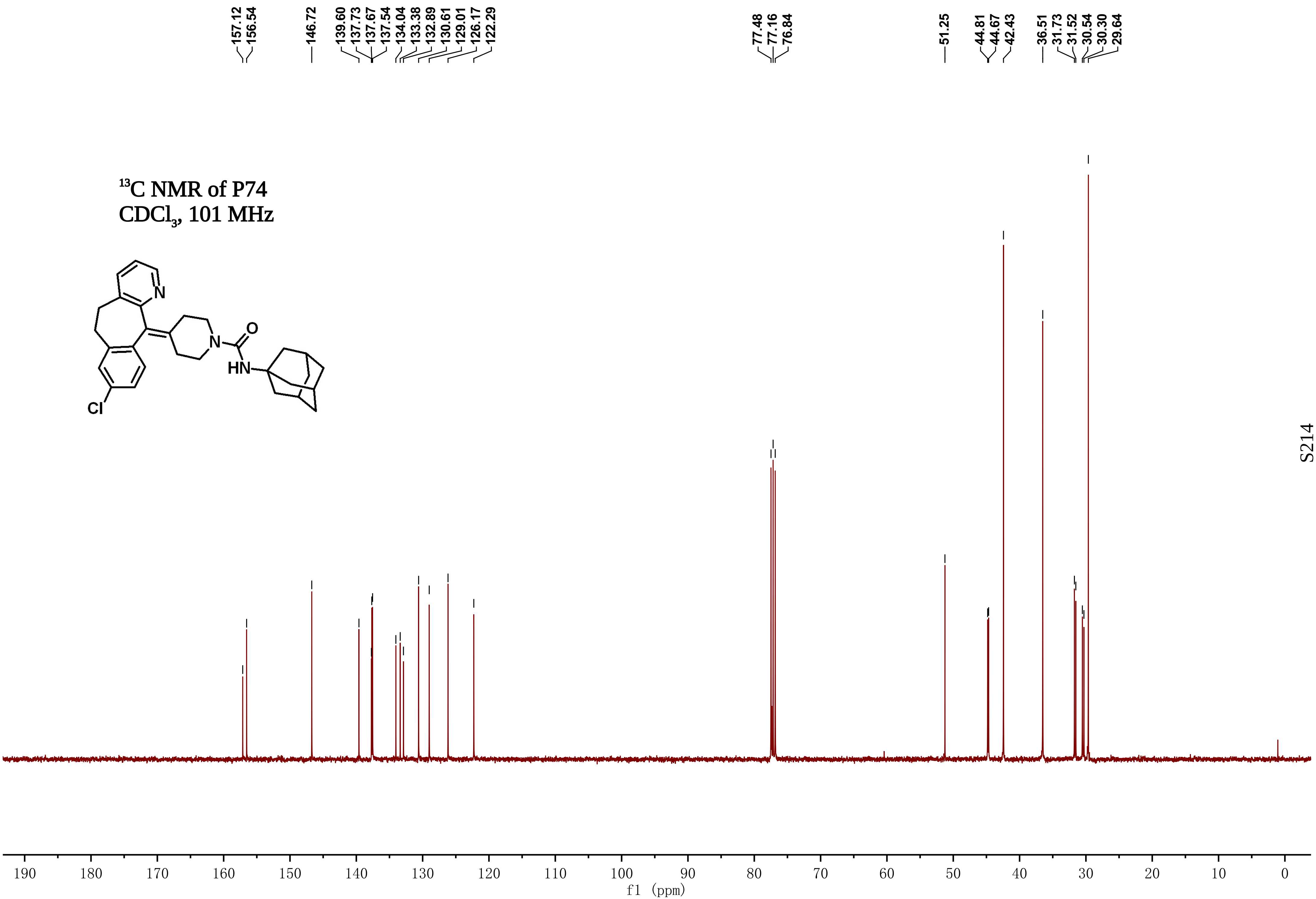
157.12
156.54
146.72
139.60
137.73
137.67
137.54
134.04
133.38
132.89
130.61
129.01
126.17
122.29
77.48
77.16
76.84
51.25
44.81
44.67
42.43
36.51
31.73
31.52
30.54
30.30
29.64
13C NMR of P74
CDCl3, 101 MHz
N
O
N
HN
Cl
190 180 170 160 150 140 130 120 110 100 90 80 70 60 50 40 30 20 10 0
f1 (ppm)

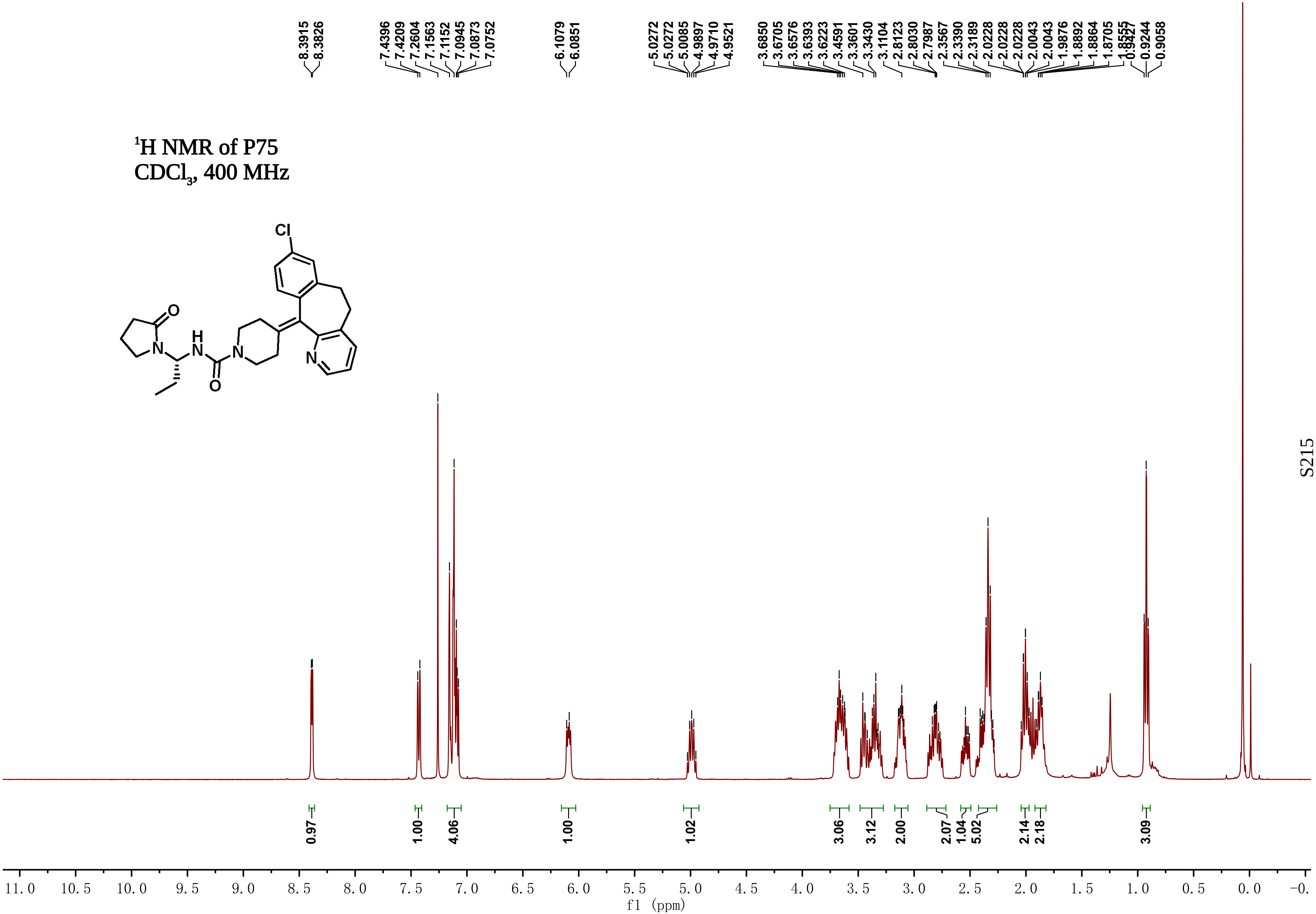

1H NMR of P75
CDCl3, 400 MHz
Cl
O
N
H
N
N
O
N
8.3915
8.3826
7.4396
7.4209
7.2604
7.1563
7.1152
7.0945
7.0873
7.0752
6.1079
6.0851
5.0272
5.0272
5.0085
4.9897
4.9710
4.9521
3.6850
3.6705
3.6576
3.6393
3.6223
3.4591
3.3601
3.3430
3.1104
2.8123
2.8030
2.7987
2.3567
2.3390
2.3189
2.0228
2.0228
2.0228
2.0043
2.0043
1.9876
1.8892
1.8864
1.8705
1.8555
0.9427
0.9244
0.9058
0.97
1.00
4.06
1.00
1.02
3.06
3.12
2.00
2.07
1.04
5.02
2.14
2.18
3.09
11.0 10.5 10.0 9.5 9.0 8.5 8.0 7.5 7.0 6.5 6.0 5.5 5.0 4.5 4.0 3.5 3.0 2.5 2.0 1.5 1.0 0.5 0.0 -0.
f1 (ppm)

^{13}C NMR of P75
$CDCl_3$, 101 MHz

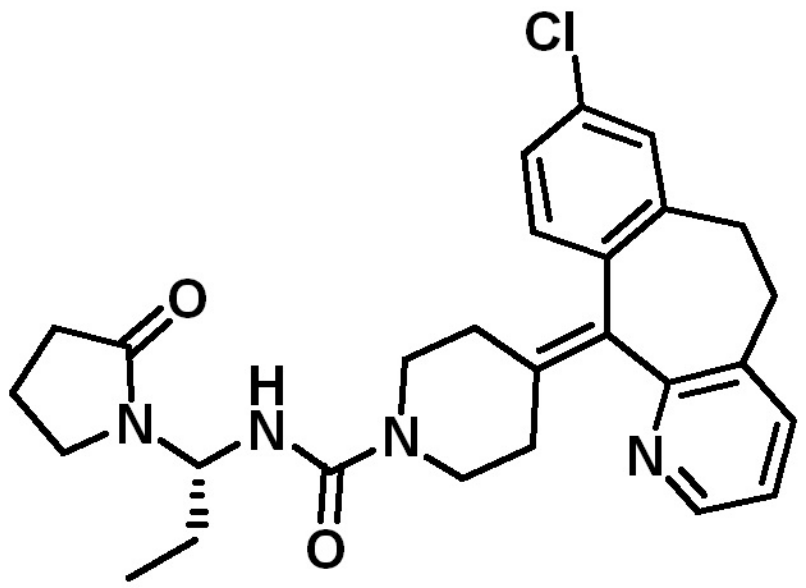

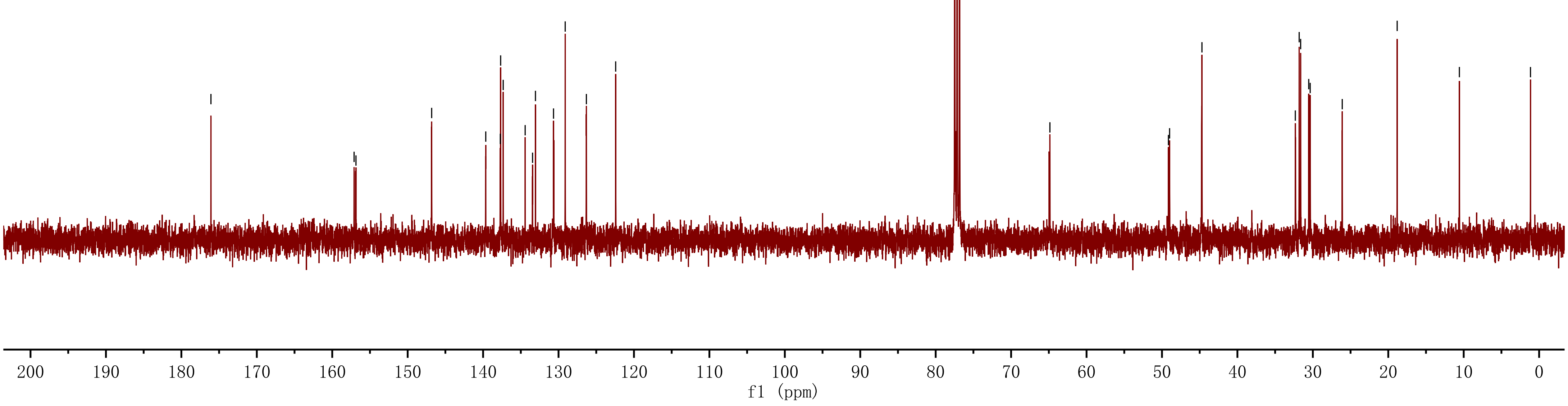

^{1}H NMR of P76
$CDCl_3$, 400 MHz

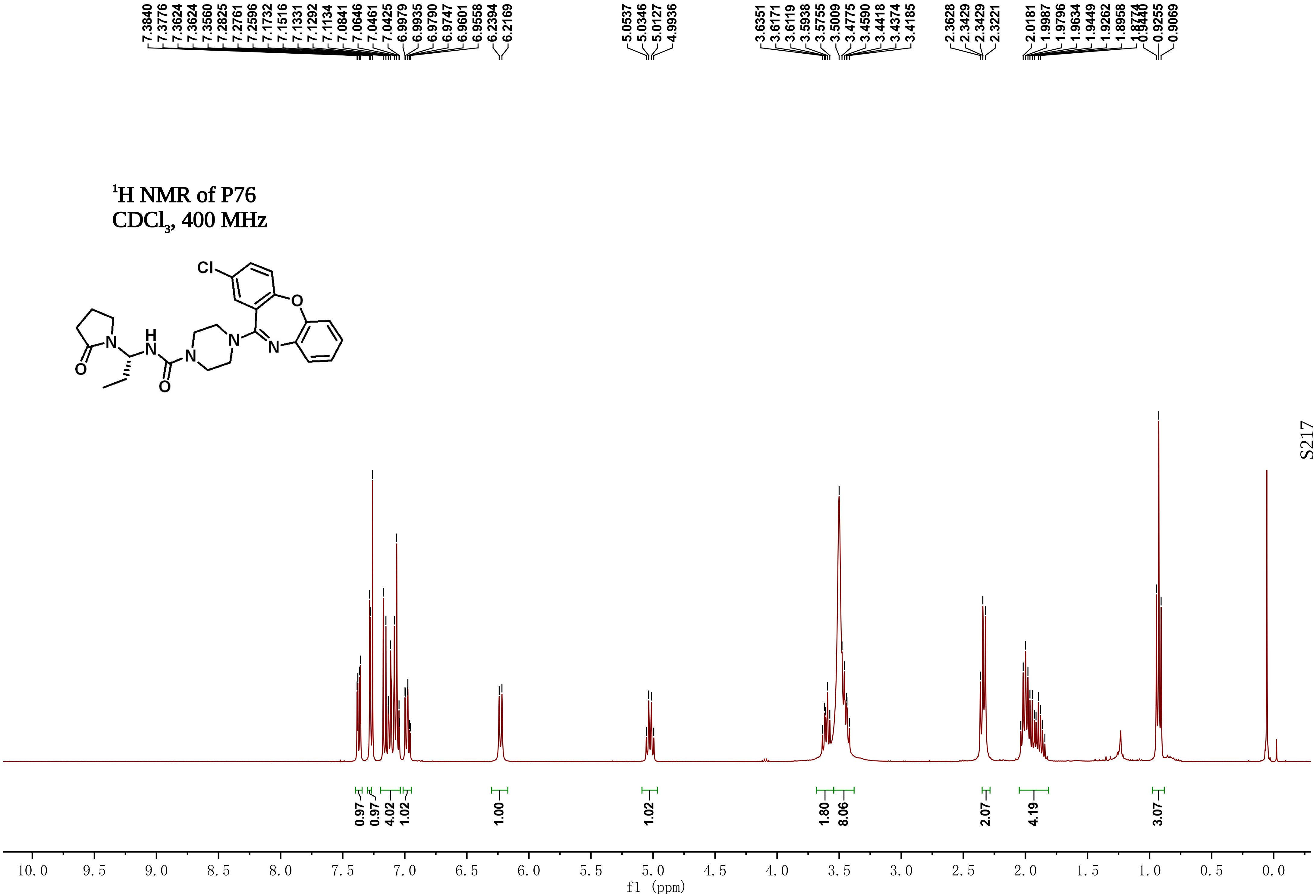

^{13}C NMR of P76
$CDCl_3$, 101 MHz

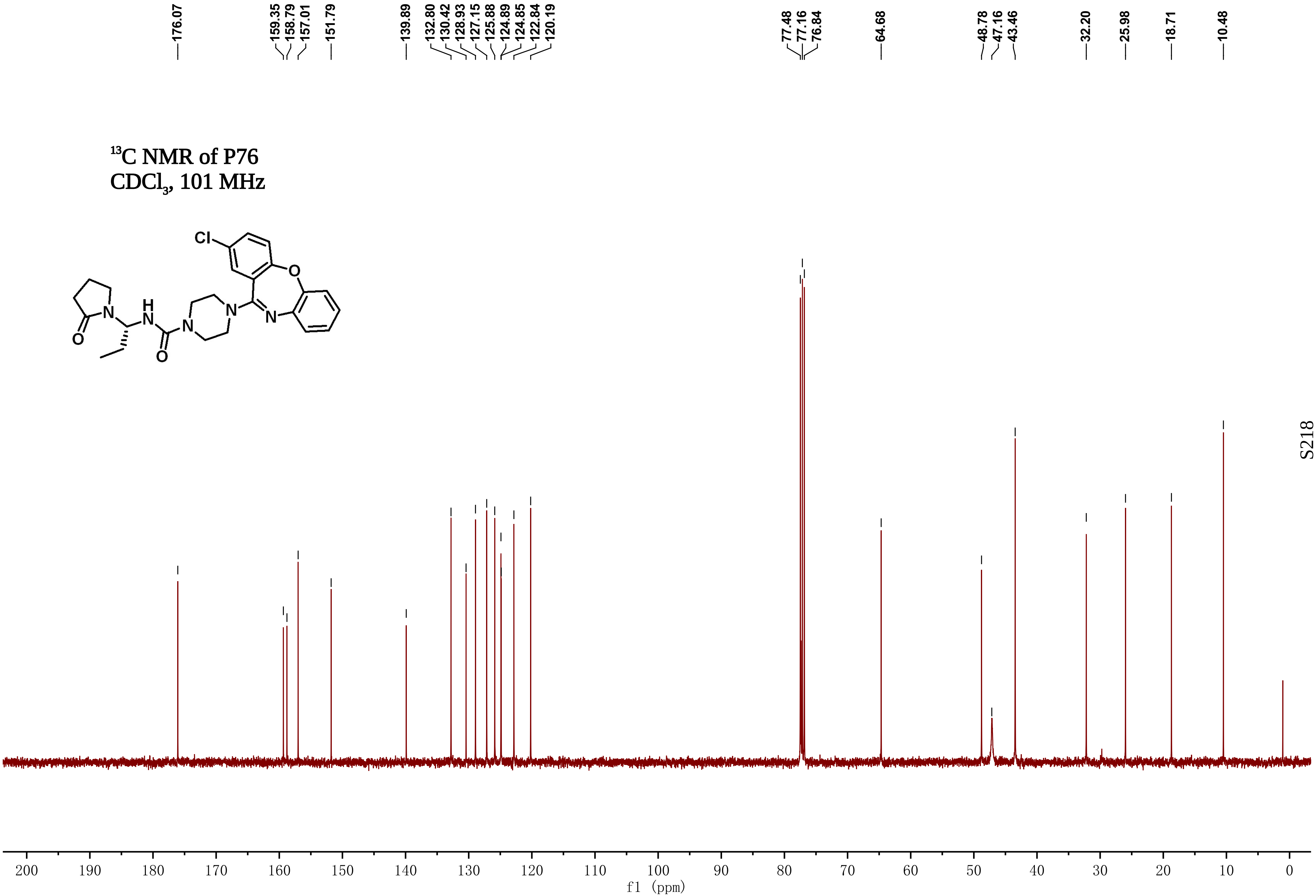

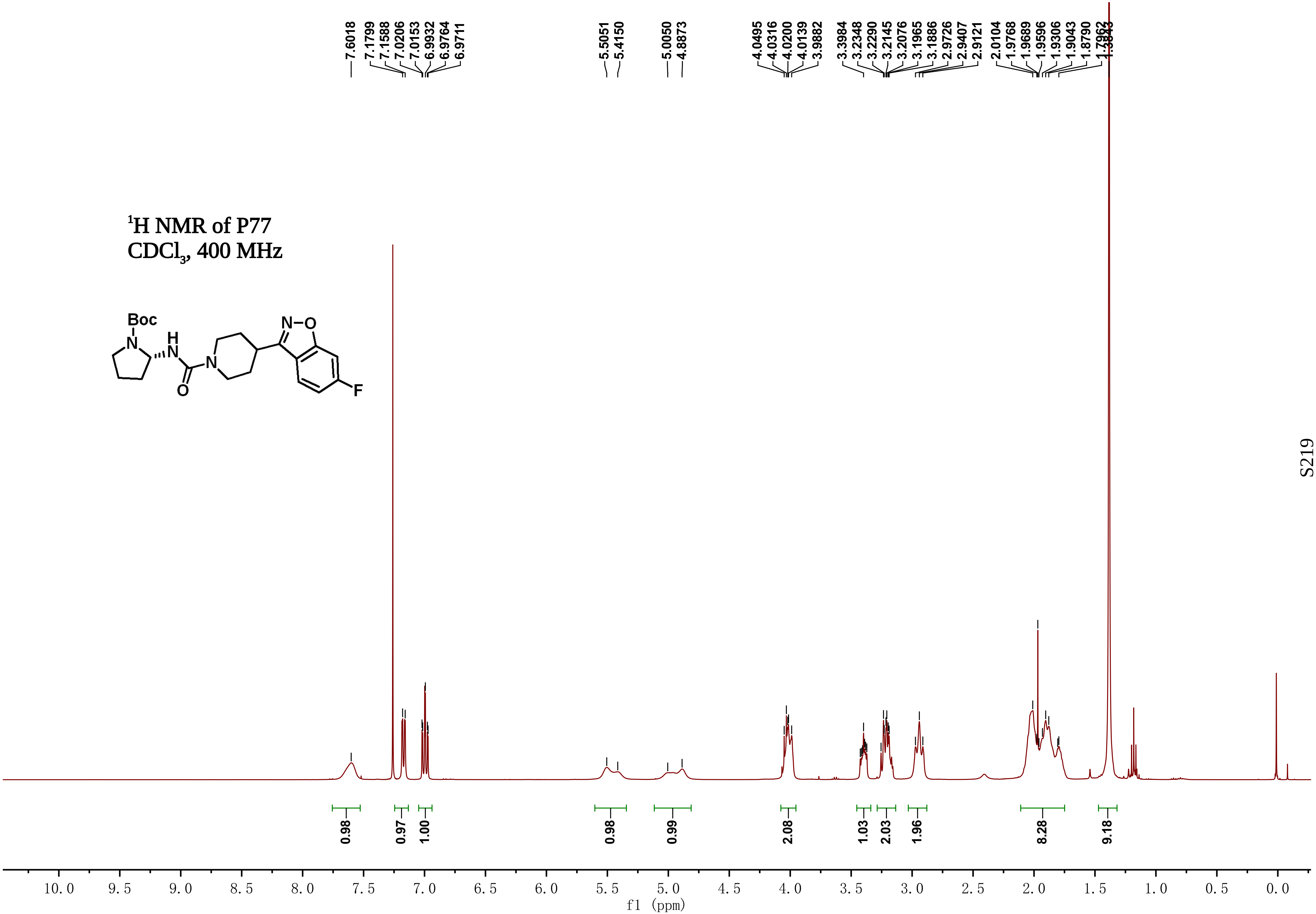

1H NMR of P77
CDCl3, 400 MHz
Boc
N
H
N
N
O
N–O
F
7.6018
7.1799
7.1588
7.0206
7.0153
6.9932
6.9764
6.9711
5.5051
5.4150
5.0050
4.8873
4.0495
4.0316
4.0200
4.0139
3.9882
3.3984
3.2348
3.2290
3.2145
3.2076
3.1965
3.1886
2.9726
2.9407
2.9121
2.0104
1.9768
1.9689
1.9596
1.9306
1.9043
1.8790
1.7962
0.98
0.97
1.00
0.98
0.99
2.08
1.03
2.03
1.96
8.28
9.18
10.0 9.5 9.0 8.5 8.0 7.5 7.0 6.5 6.0 5.5 5.0 4.5 4.0 3.5 3.0 2.5 2.0 1.5 1.0 0.5 0.0
f1 (ppm)

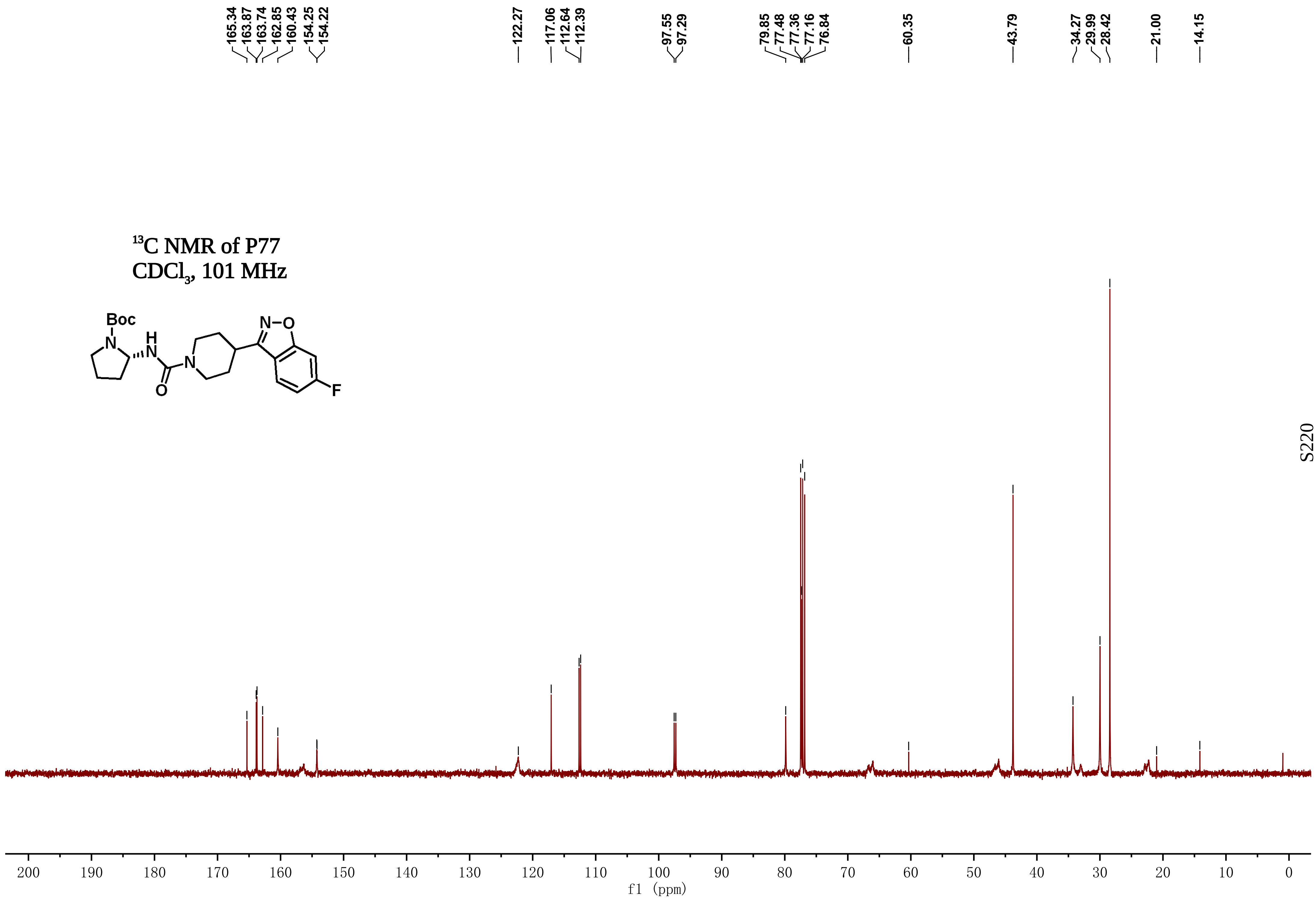

165.34
163.87
163.74
162.85
160.43
154.25
154.22
122.27
117.06
112.64
112.39
97.55
97.29
79.85
77.48
77.36
77.16
76.84
60.35
43.79
34.27
29.99
28.42
21.00
14.15
^{13}C NMR of P77
$CDCl_3$, 101 MHz
Boc
N
H
N
O
N
N–O
F
200 190 180 170 160 150 140 130 120 110 100 90 80 70 60 50 40 30 20 10 0
f1 (ppm)

^{19}F NMR of P77
$CDCl_3$, 376 MHz

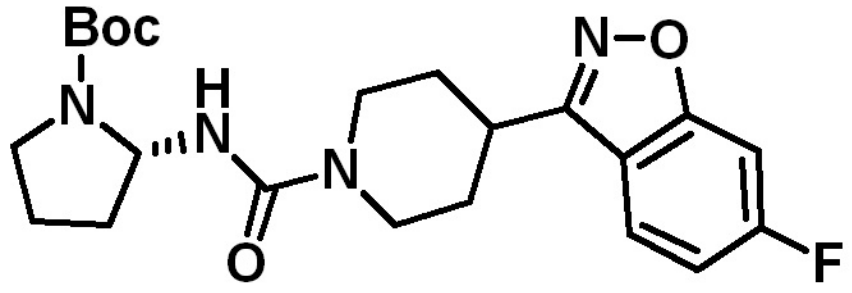

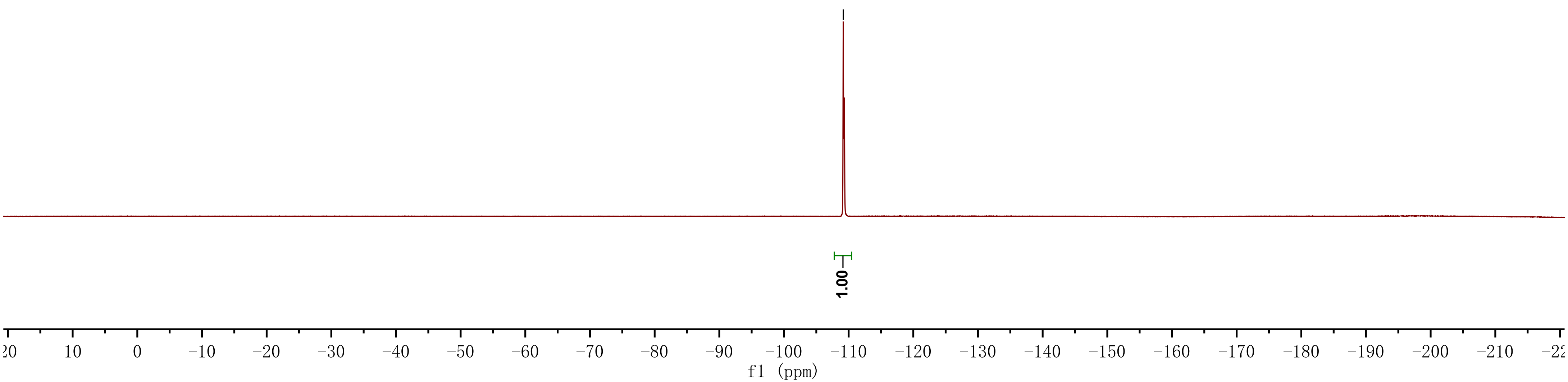

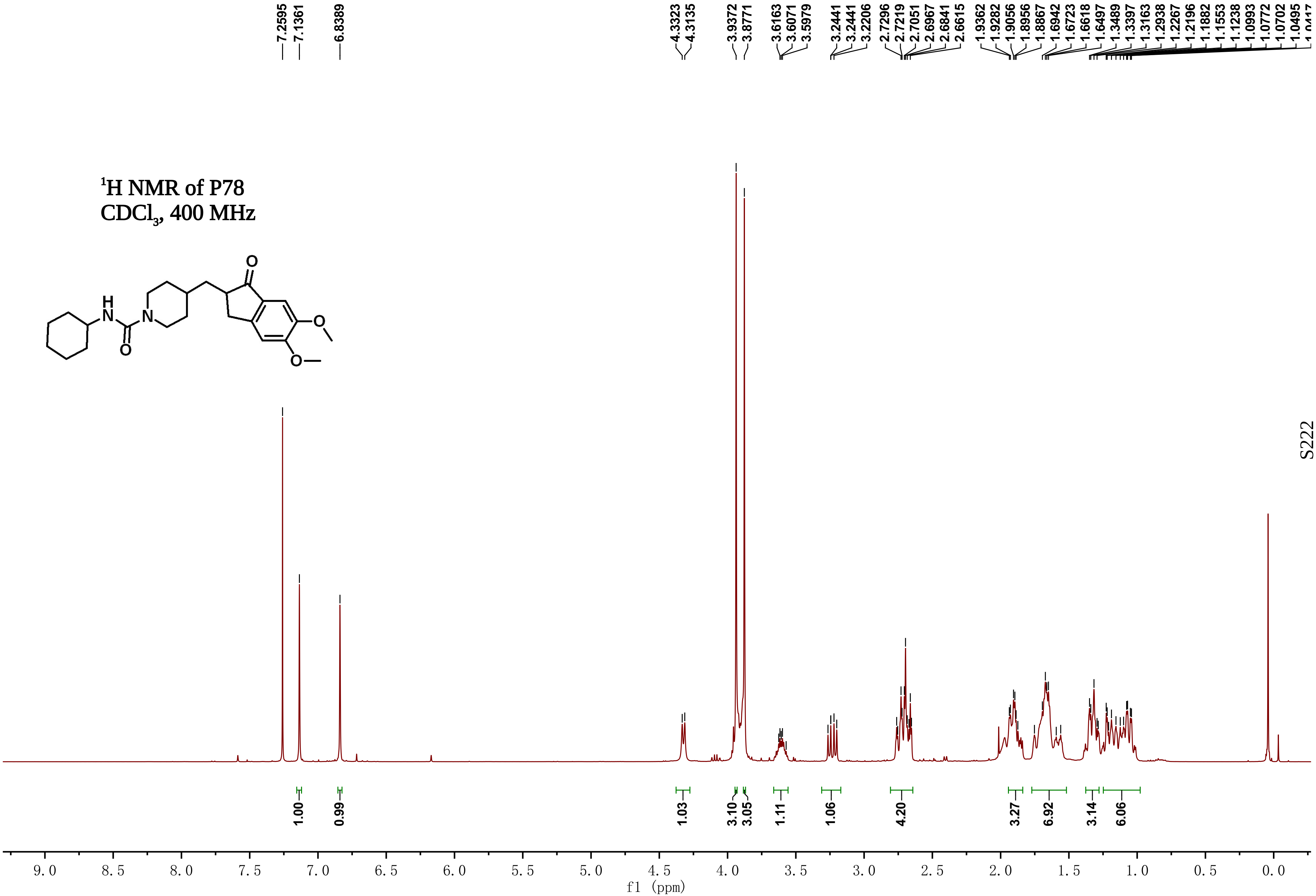

1H NMR of P78
CDCl3, 400 MHz
7.2595
7.1361
6.8389
4.3323
4.3135
3.9372
3.8771
3.6163
3.6071
3.5979
3.2441
3.2441
3.2206
2.7296
2.7219
2.7051
2.6967
2.6841
2.6615
1.9362
1.9282
1.9056
1.8956
1.8867
1.6942
1.6723
1.6618
1.6497
1.3489
1.3397
1.3163
1.2938
1.2267
1.2196
1.1882
1.1553
1.1238
1.0993
1.0772
1.0702
1.0495
1.00
0.99
1.03
3.10
3.05
1.11
1.06
4.20
3.27
6.92
3.14
6.06
9.0 8.5 8.0 7.5 7.0 6.5 6.0 5.5 5.0 4.5 4.0 3.5 3.0 2.5 2.0 1.5 1.0 0.5 0.0
f1 (ppm)

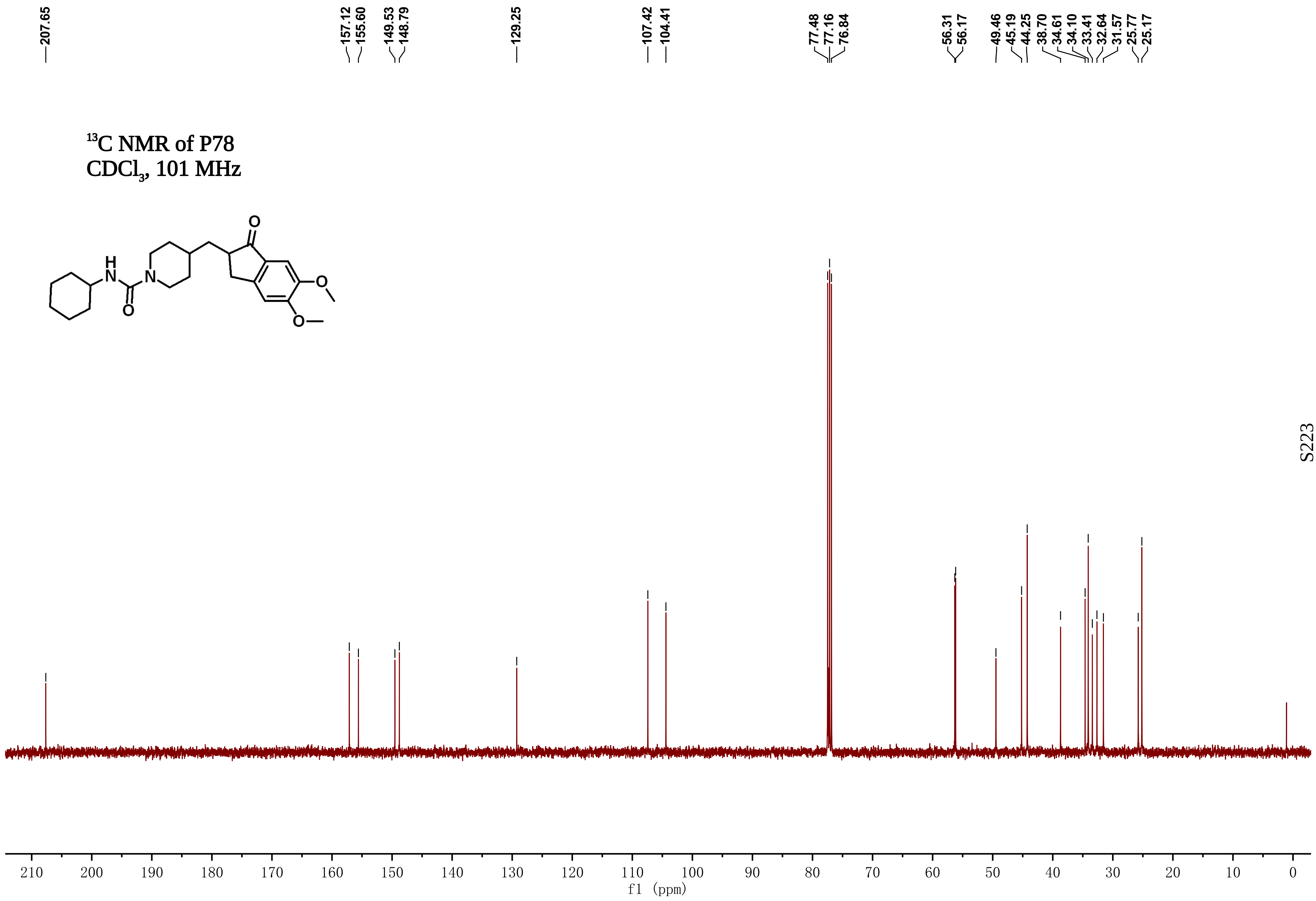
207.65
157.12
155.60
149.53
148.79
129.25
107.42
104.41
77.48
77.16
76.84
56.31
56.17
49.46
45.19
44.25
38.70
34.61
34.10
33.41
32.64
31.57
25.77
25.17
¹³C NMR of P78
CDCl₃, 101 MHz
O
H
N
N
O
O
O
210 200 190 180 170 160 150 140 130 120 110 100 90 80 70 60 50 40 30 20 10 0
f1 (ppm)

^{1}H NMR of P79
$CDCl_3$, 400 MHz

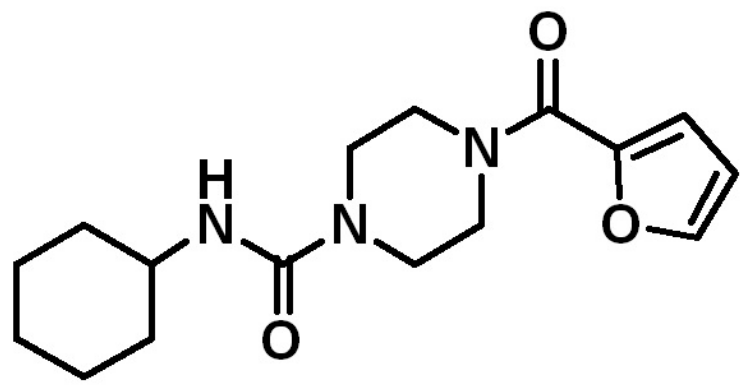

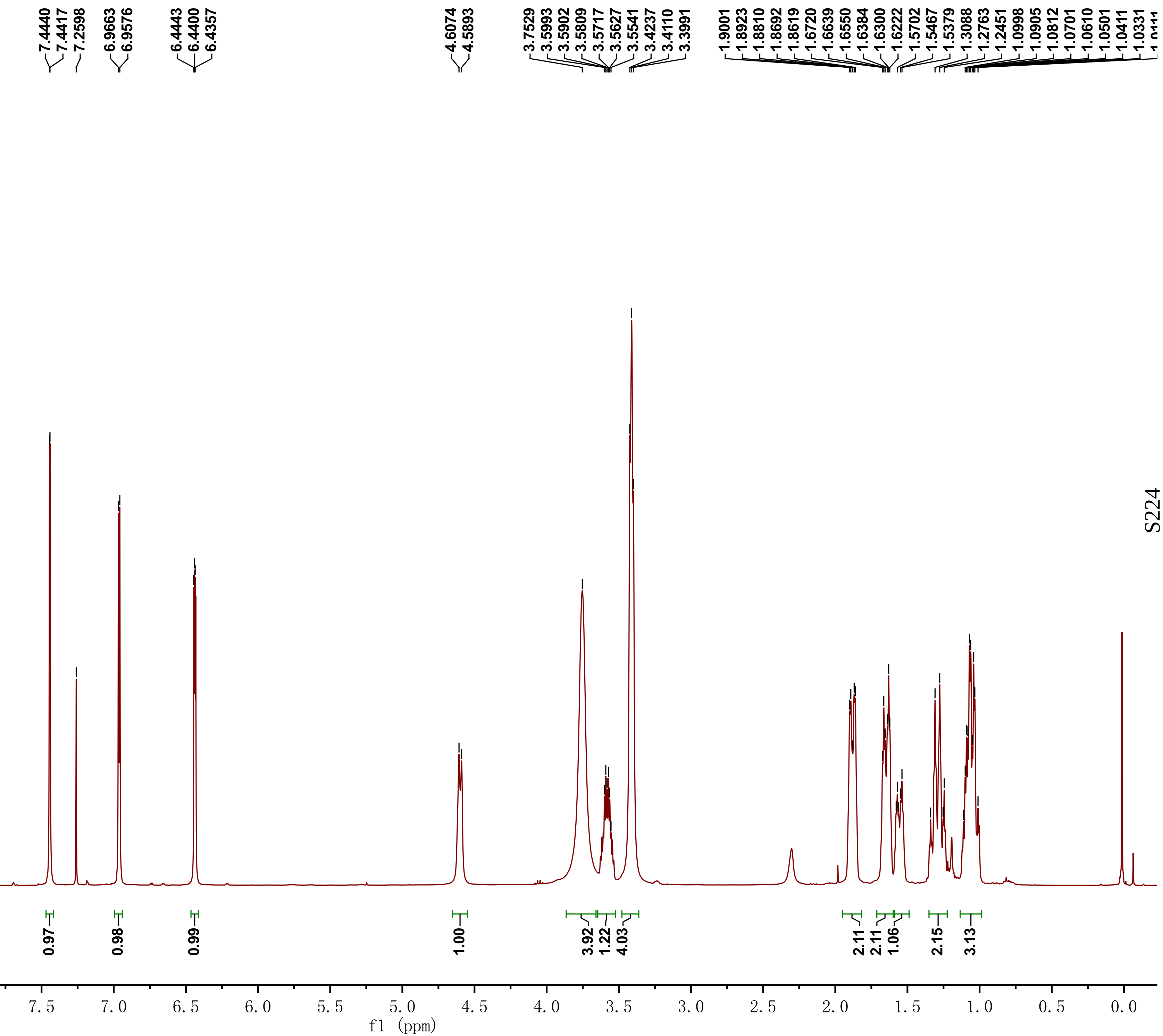

^{13}C NMR of P79
$CDCl_3$, 101 MHz

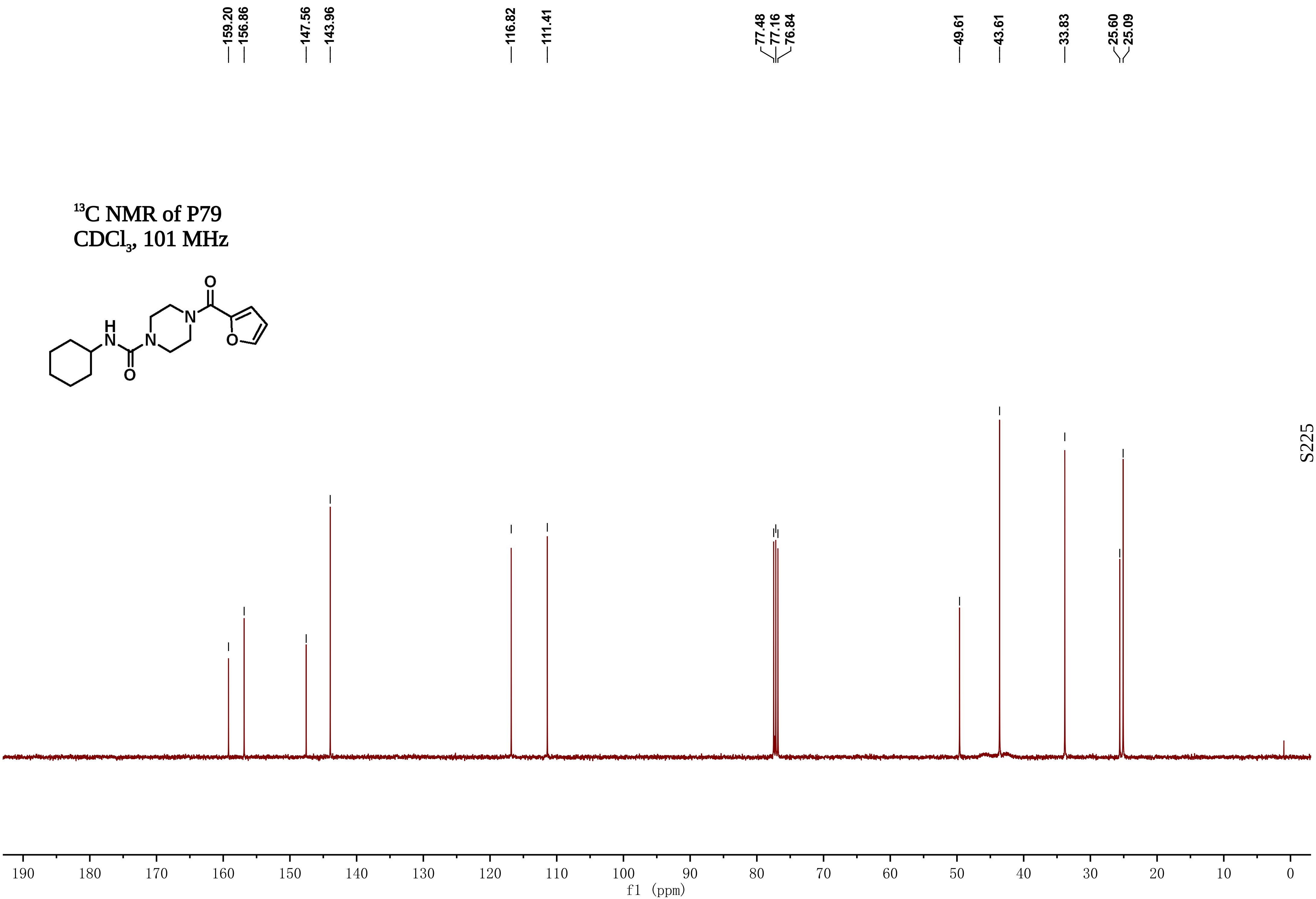

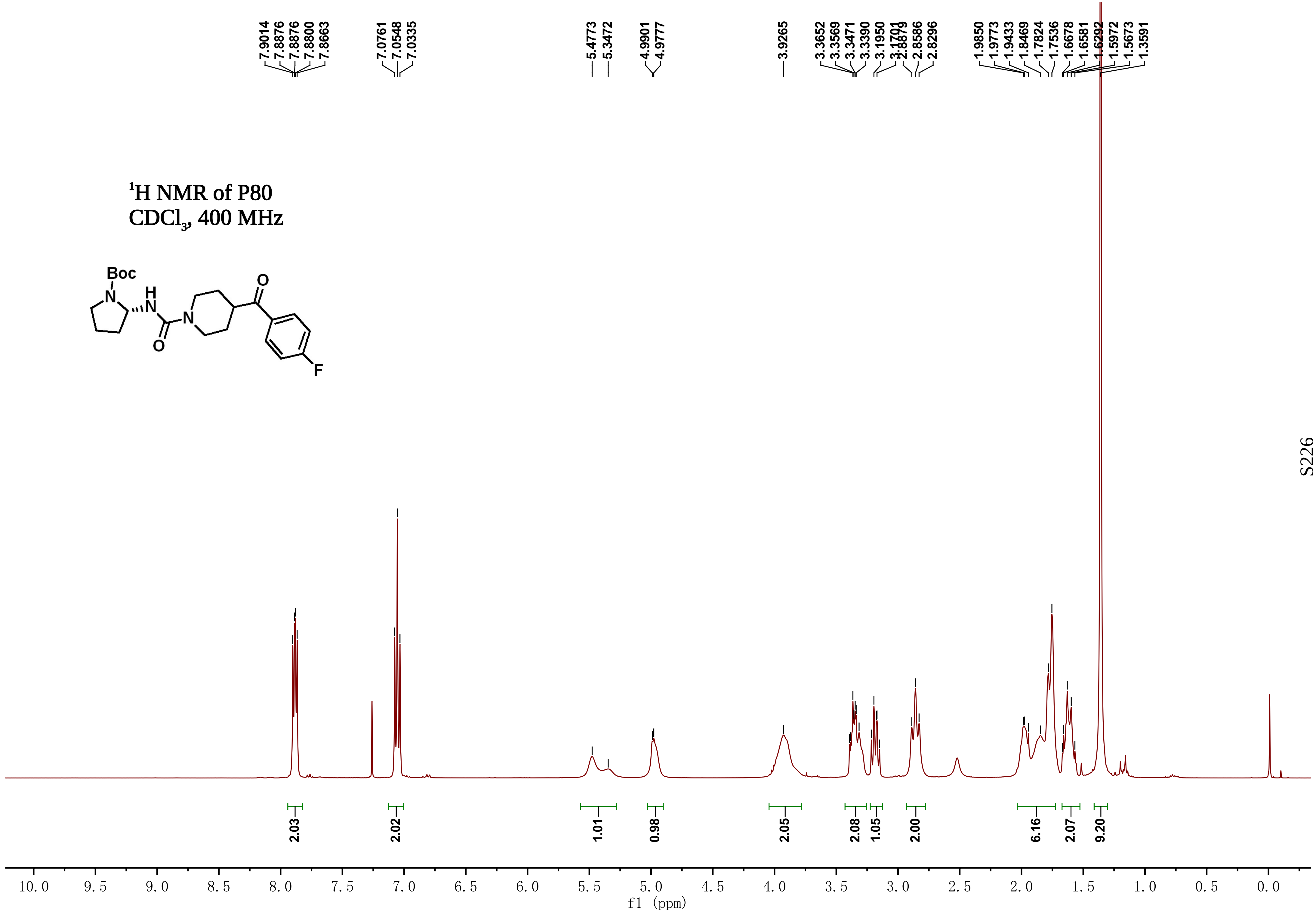

1H NMR of P80
CDCl3, 400 MHz
Boc
H
N
N
N
O
O
F
7.9014
7.8876
7.8876
7.8800
7.8663
7.0761
7.0548
7.0335
5.4773
5.3472
4.9901
4.9777
3.9265
3.3652
3.3569
3.3471
3.3390
3.1950
3.1701
2.8879
2.8586
2.8296
1.9850
1.9773
1.9433
1.8469
1.7824
1.7536
1.6678
1.6581
1.6292
1.5972
1.5673
1.3591
2.03
2.02
1.01
0.98
2.05
2.08
1.05
2.00
6.16
2.07
9.20
10.0
9.5
9.0
8.5
8.0
7.5
7.0
6.5
6.0
5.5
5.0
4.5
4.0
3.5
3.0
2.5
2.0
1.5
1.0
0.5
0.0
f1 (ppm)

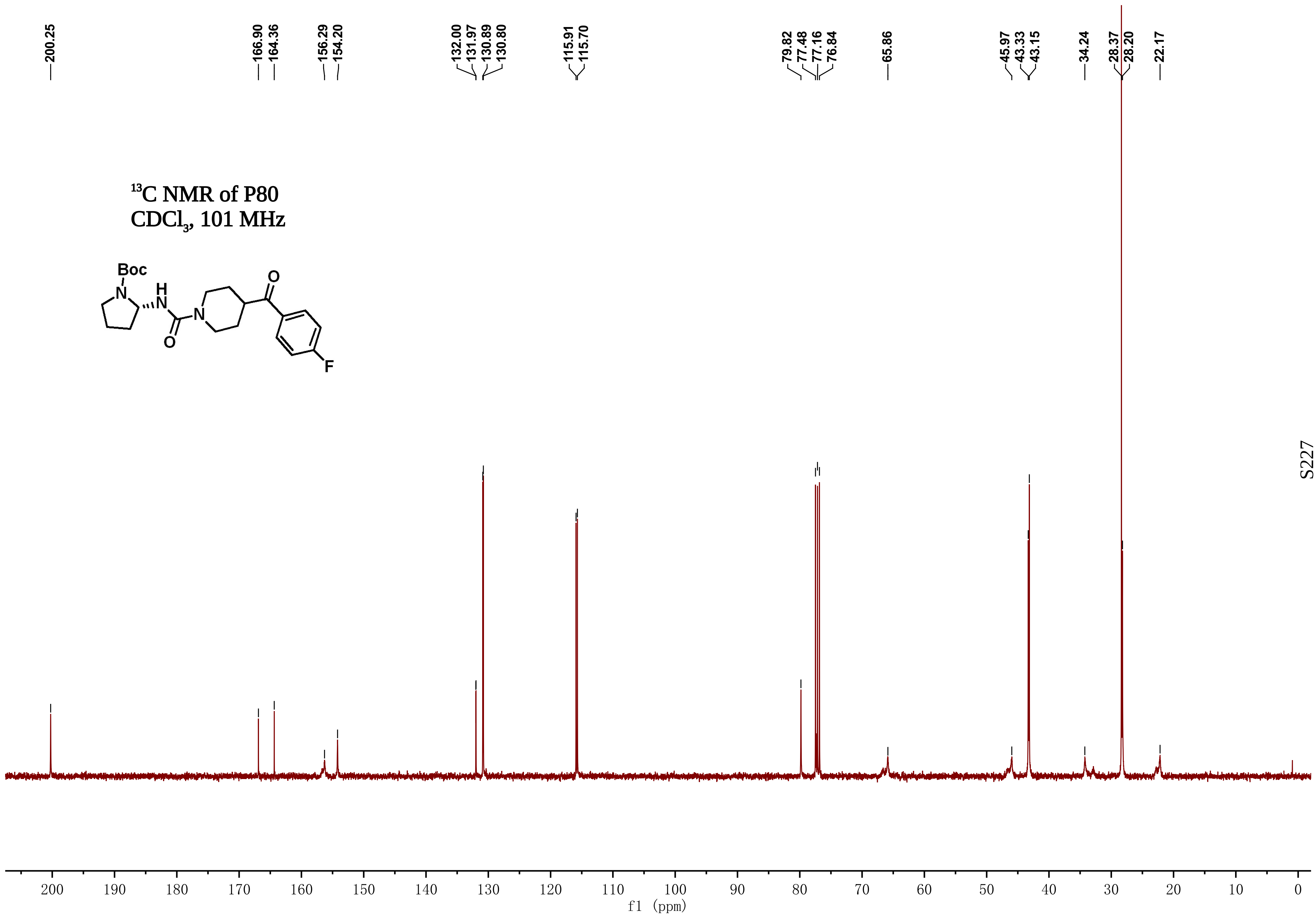

200.25
166.90
164.36
156.29
154.20
132.00
131.97
130.89
130.80
115.91
115.70
79.82
77.48
77.16
76.84
65.86
45.97
43.33
43.15
34.24
28.37
28.20
22.17
^{13}C NMR of P80
$CDCl_3$, 101 MHz
Boc
N
H
N
O
N
O
F
200 190 180 170 160 150 140 130 120 110 100 90 80 70 60 50 40 30 20 10 0
f1 (ppm)

^{19}F NMR of P80
$CDCl_3$, 376 MHz

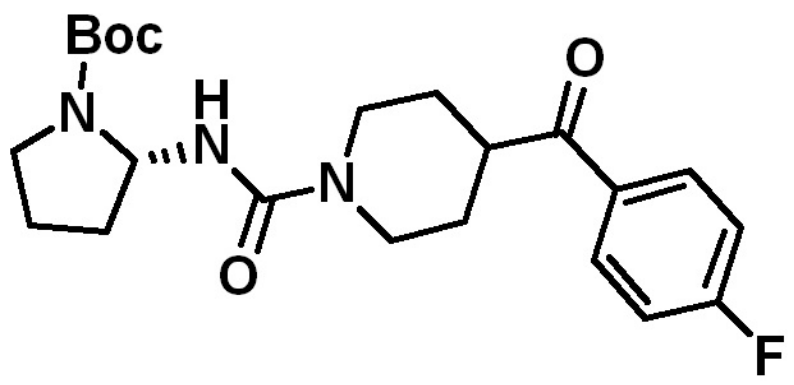

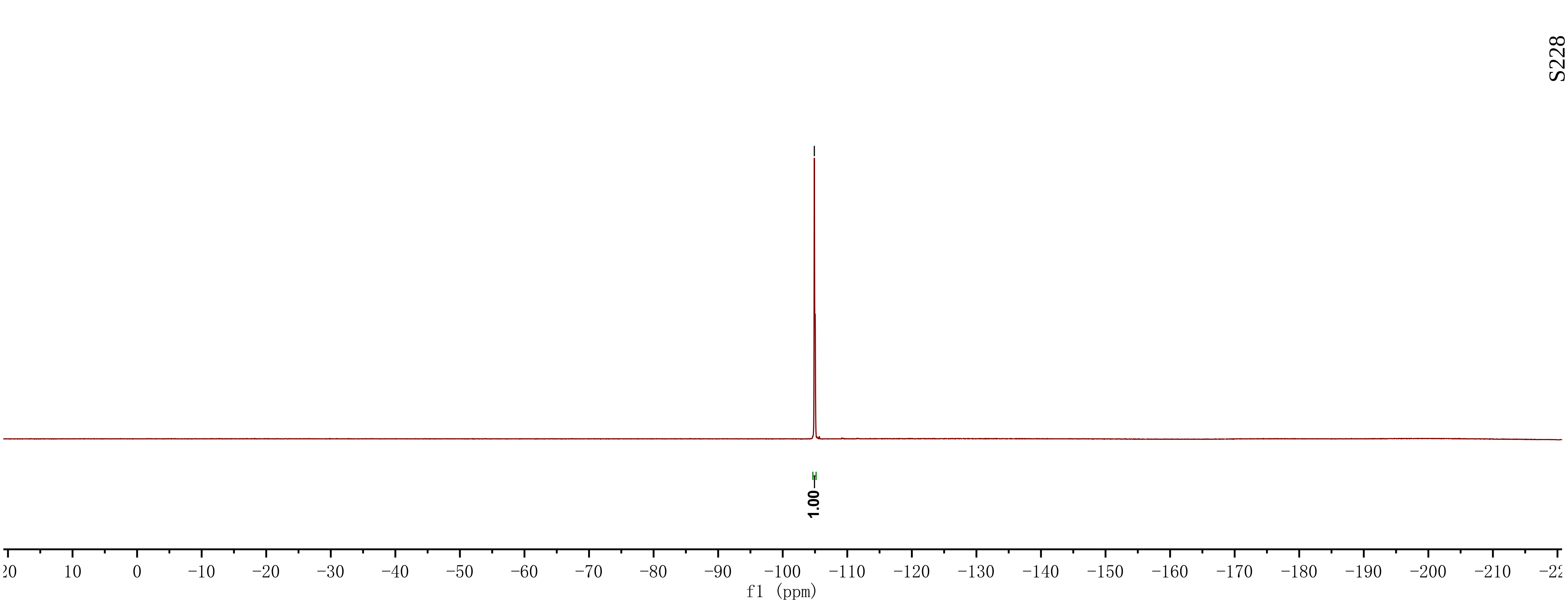

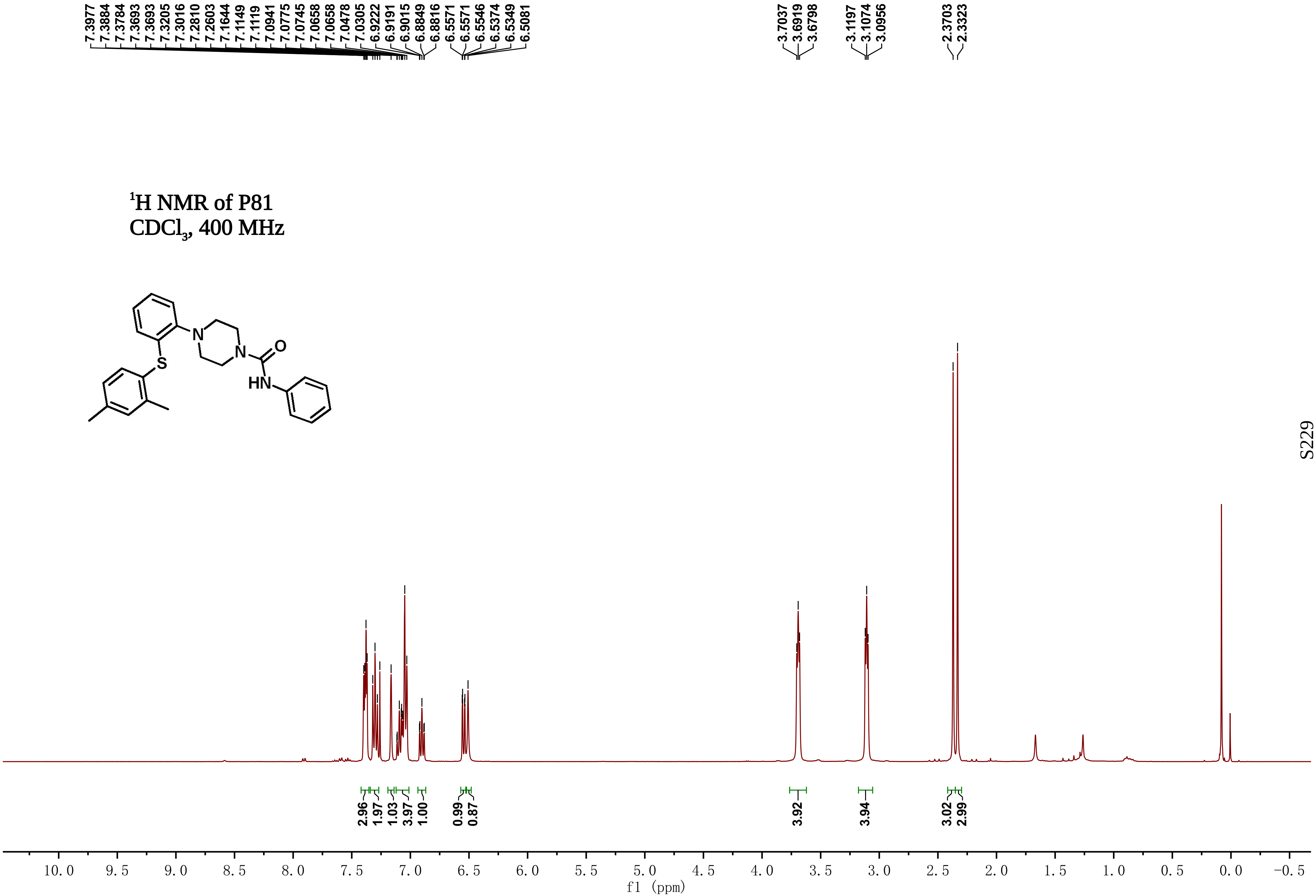

^{1}H NMR of P81
$CDCl_3$, 400 MHz
N
N
O
S
HN
7.3977
7.3884
7.3784
7.3693
7.3693
7.3205
7.3016
7.2810
7.2603
7.1644
7.1149
7.1119
7.0941
7.0775
7.0745
7.0658
7.0658
7.0478
7.0305
6.9222
6.9191
6.9015
6.8849
6.8816
6.5571
6.5571
6.5546
6.5374
6.5349
6.5081
3.7037
3.6919
3.6798
3.1197
3.1074
3.0956
2.3703
2.3323
2.96
1.97
1.03
3.97
1.00
0.99
0.87
3.92
3.94
3.02
2.99
10.0 9.5 9.0 8.5 8.0 7.5 7.0 6.5 6.0 5.5 5.0 4.5 4.0 3.5 3.0 2.5 2.0 1.5 1.0 0.5 0.0 -0.5
f1 (ppm)

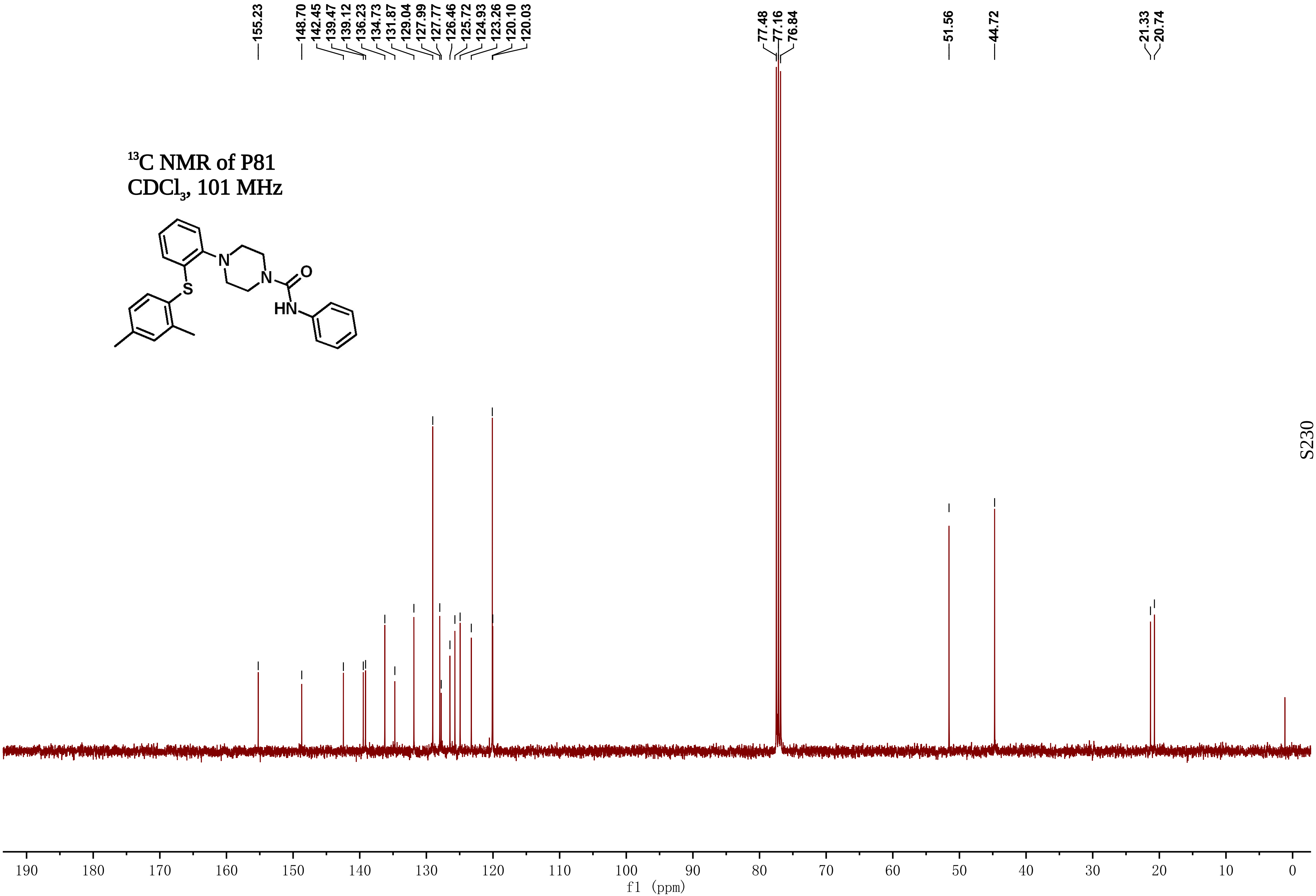

155.23
148.70
142.45
139.47
139.12
136.23
134.73
131.87
129.04
127.99
127.77
126.46
125.72
124.93
123.26
120.10
120.03
77.48
77.16
76.84
51.56
44.72
21.33
20.74
13C NMR of P81
CDCl3, 101 MHz
S
N
N
O
HN
190 180 170 160 150 140 130 120 110 100 90 80 70 60 50 40 30 20 10 0
f1 (ppm)

^{1}H NMR of P82
$CDCl_3$, 400 MHz

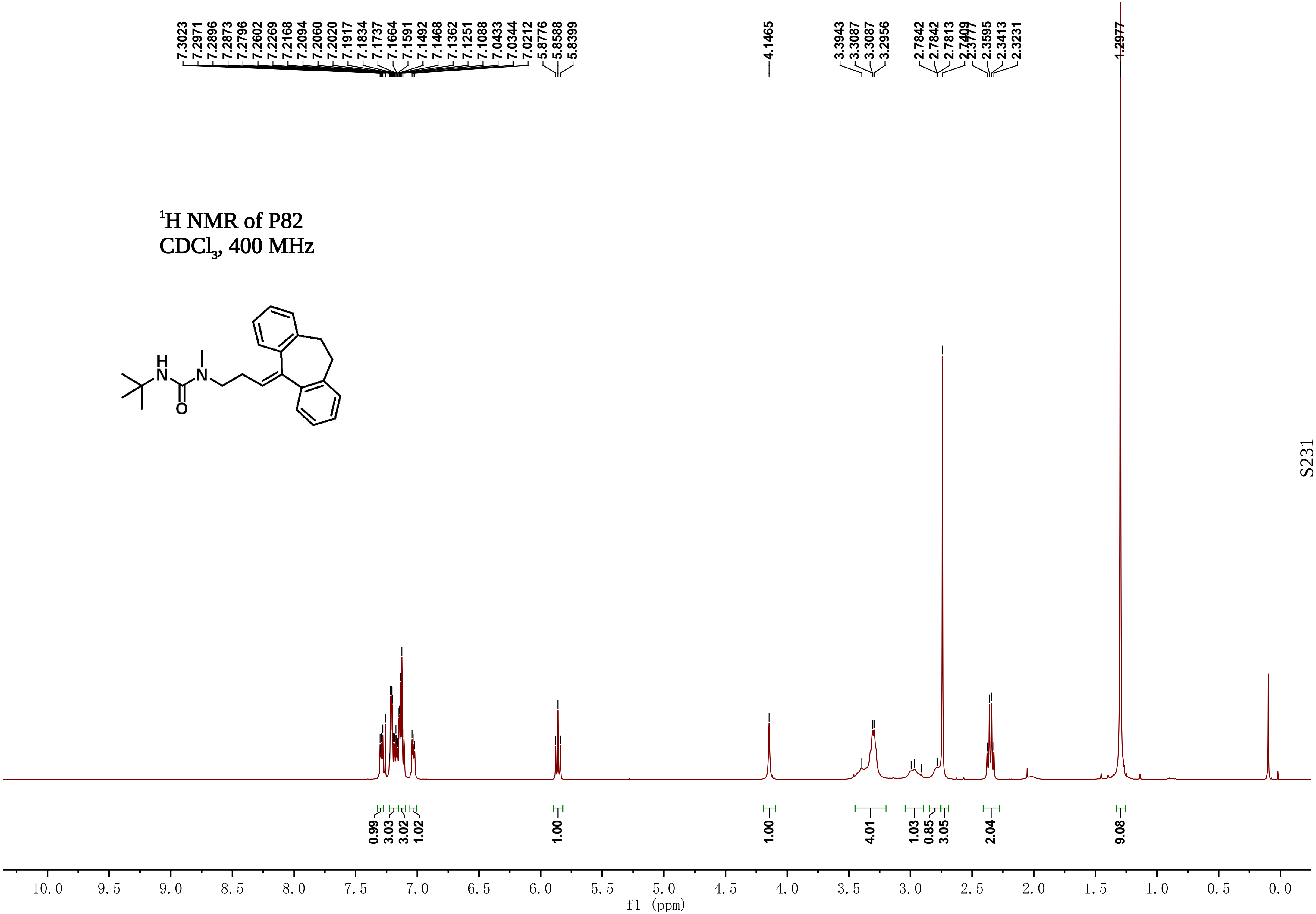

^{13}C NMR of P82

$CDCl_3$, 101 MHz

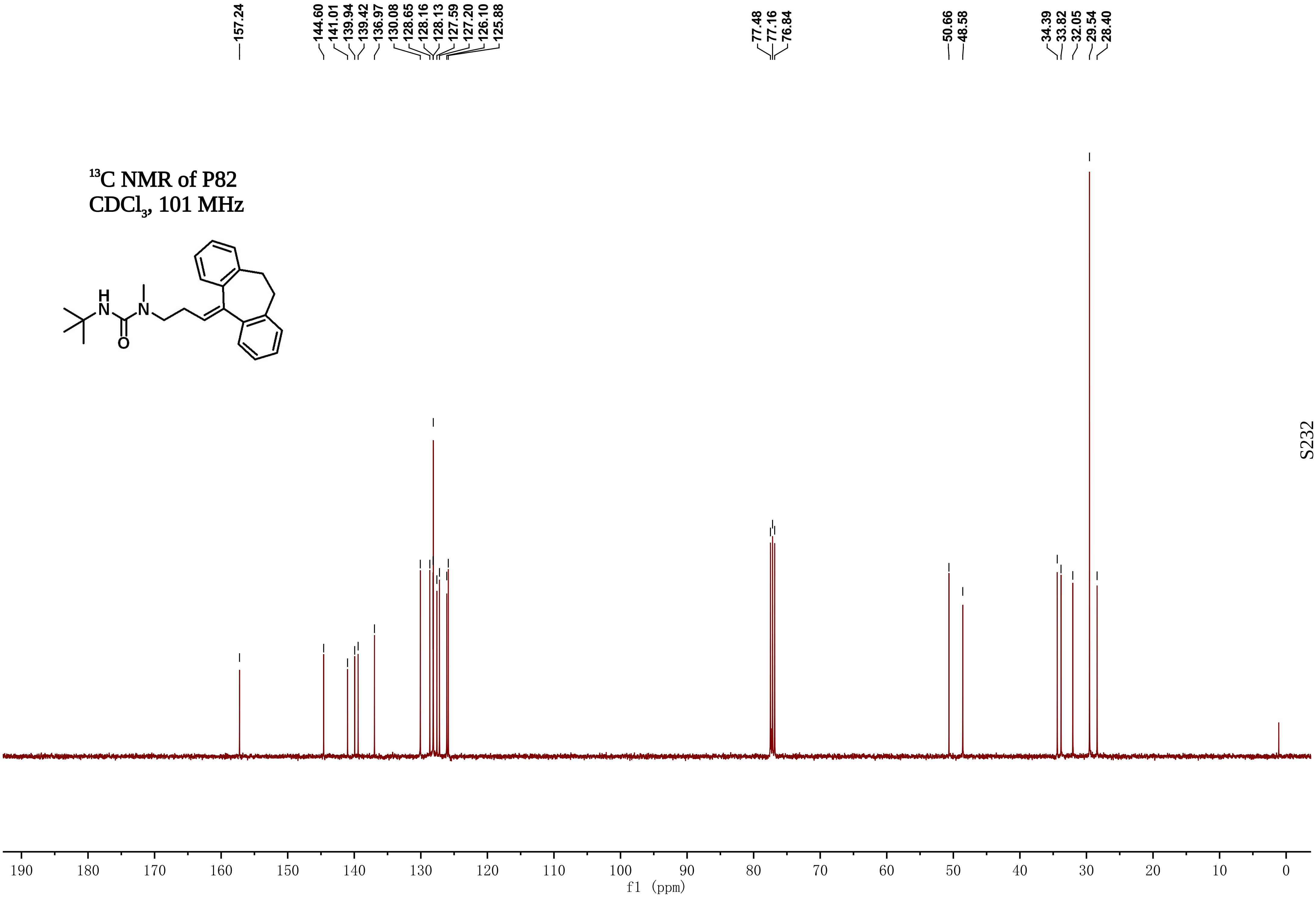

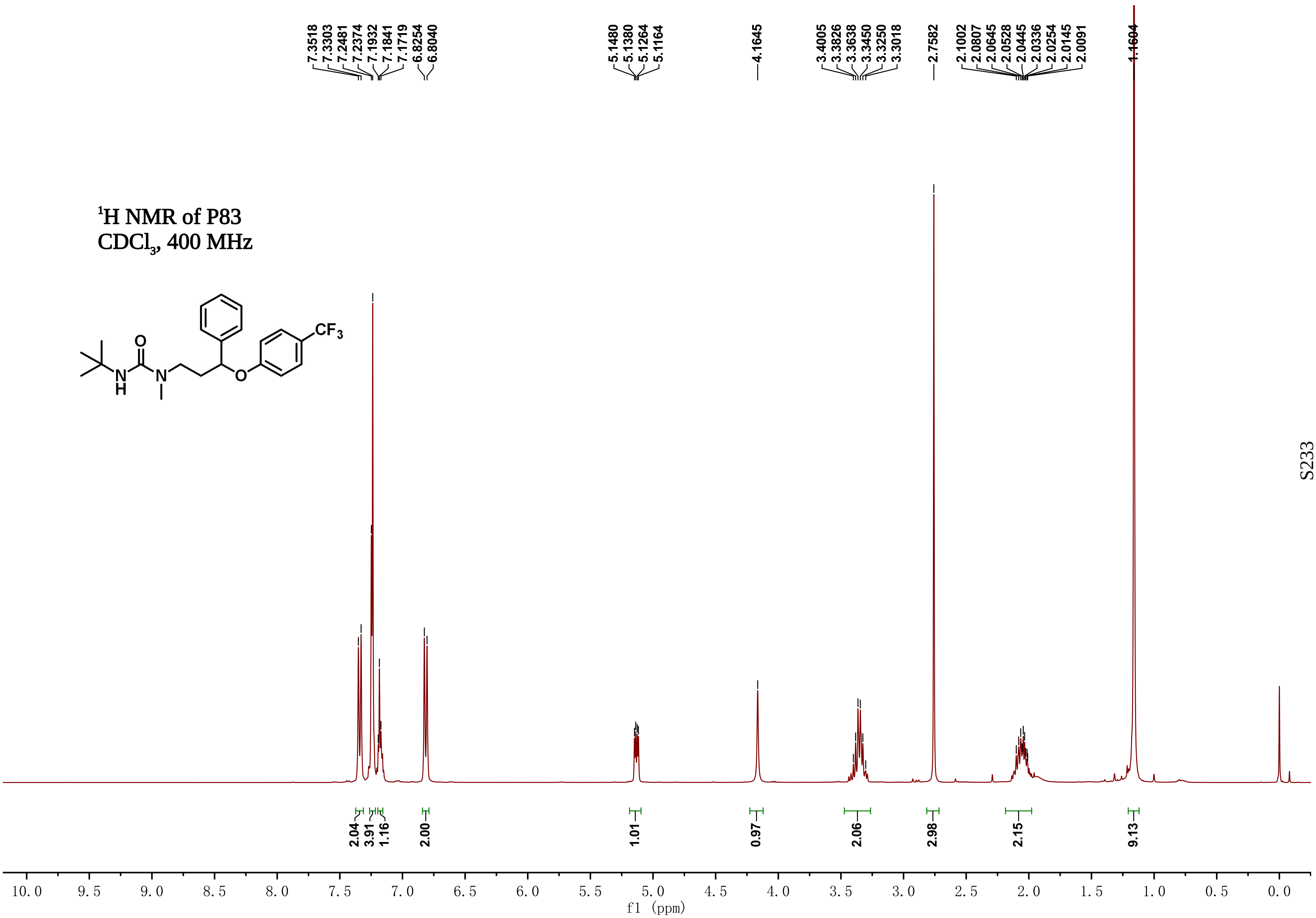

7.3518
7.3303
7.2481
7.2374
7.1932
7.1841
7.1719
6.8254
6.8040
5.1480
5.1380
5.1264
5.1164
4.1645
3.4005
3.3826
3.3638
3.3450
3.3250
3.3018
2.7582
2.1002
2.0807
2.0645
2.0528
2.0445
2.0336
2.0254
2.0145
2.0091
1.1604
1H NMR of P83
CDCl3, 400 MHz
O
N
H
N
O
CF3
2.04
3.91
1.16
2.00
1.01
0.97
2.06
2.98
2.15
9.13
10.0
9.5
9.0
8.5
8.0
7.5
7.0
6.5
6.0
5.5
5.0
4.5
4.0
3.5
3.0
2.5
2.0
1.5
1.0
0.5
0.0
f1 (ppm)

^{13}C NMR of P83
$CDCl_3$, 101 MHz

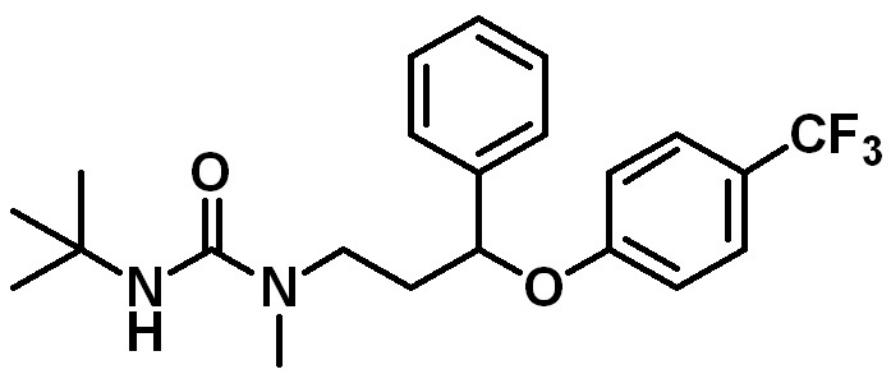

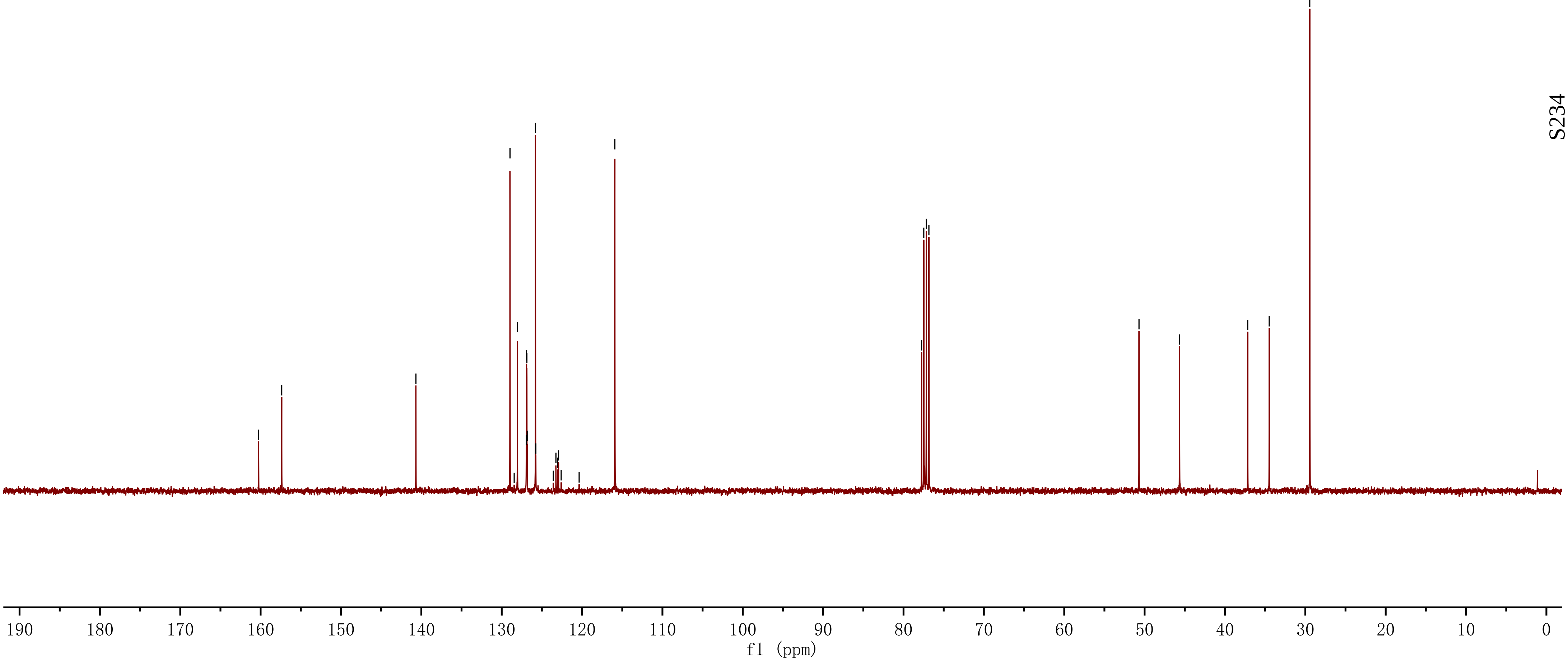

^{19}F NMR of P83
$CDCl_3$, 376 MHz

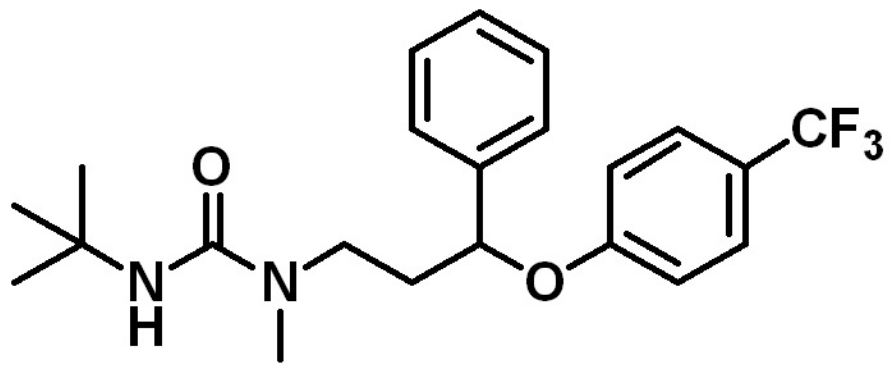

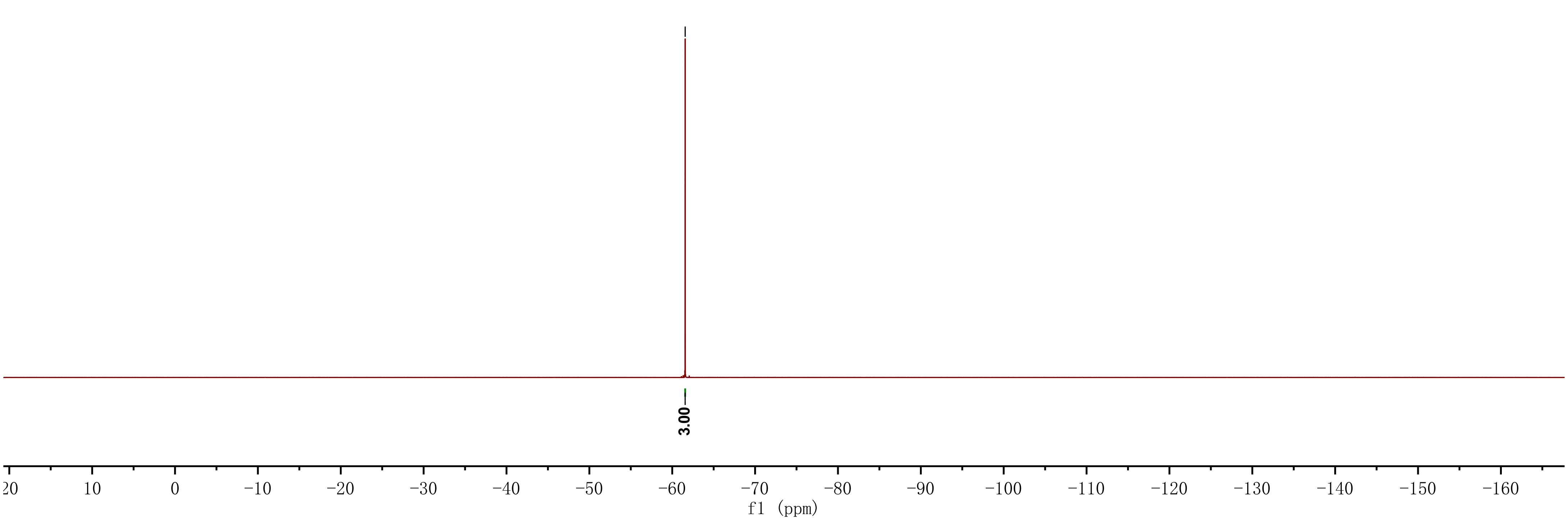

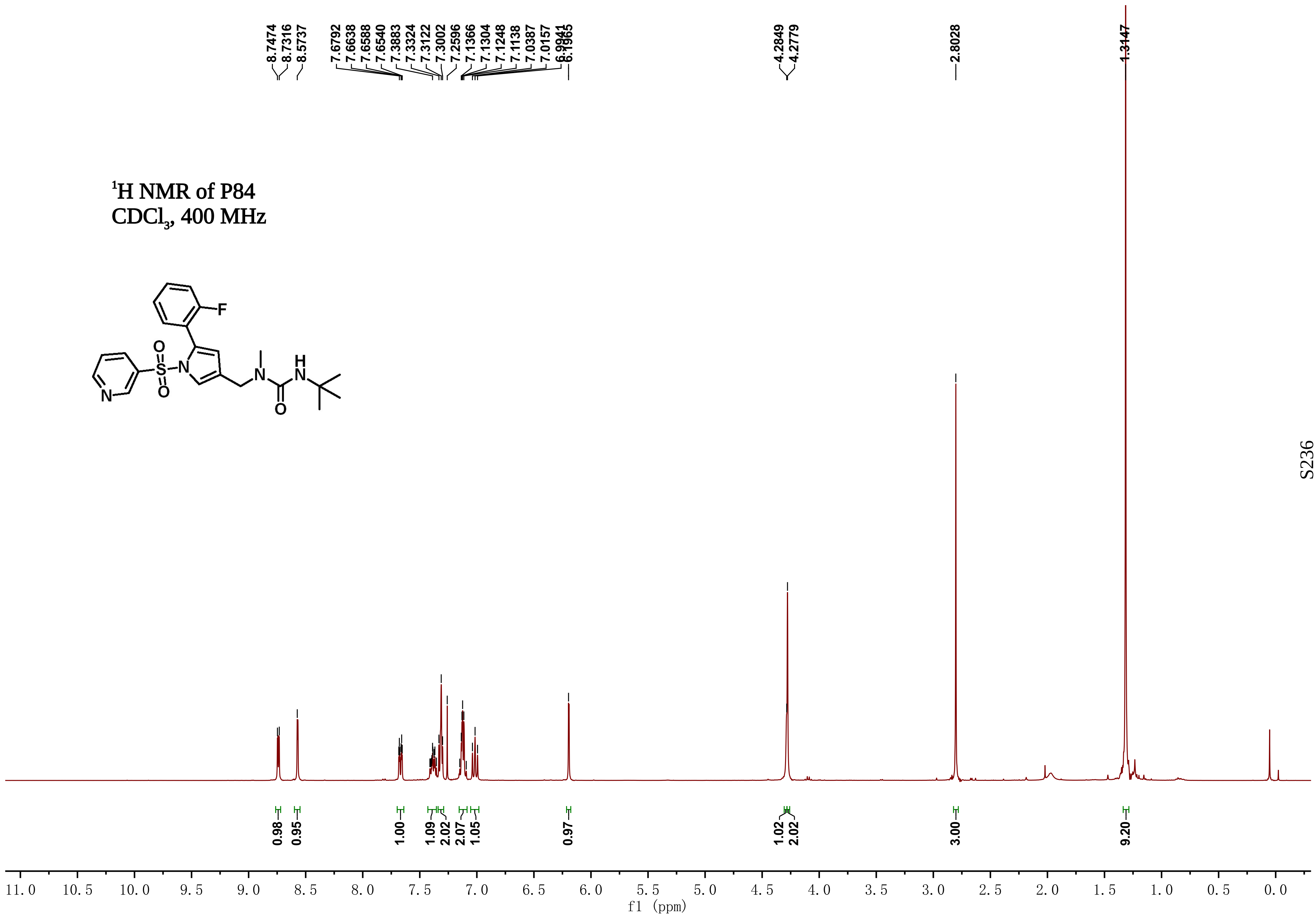

^{1}H NMR of P84
$CDCl_3$, 400 MHz

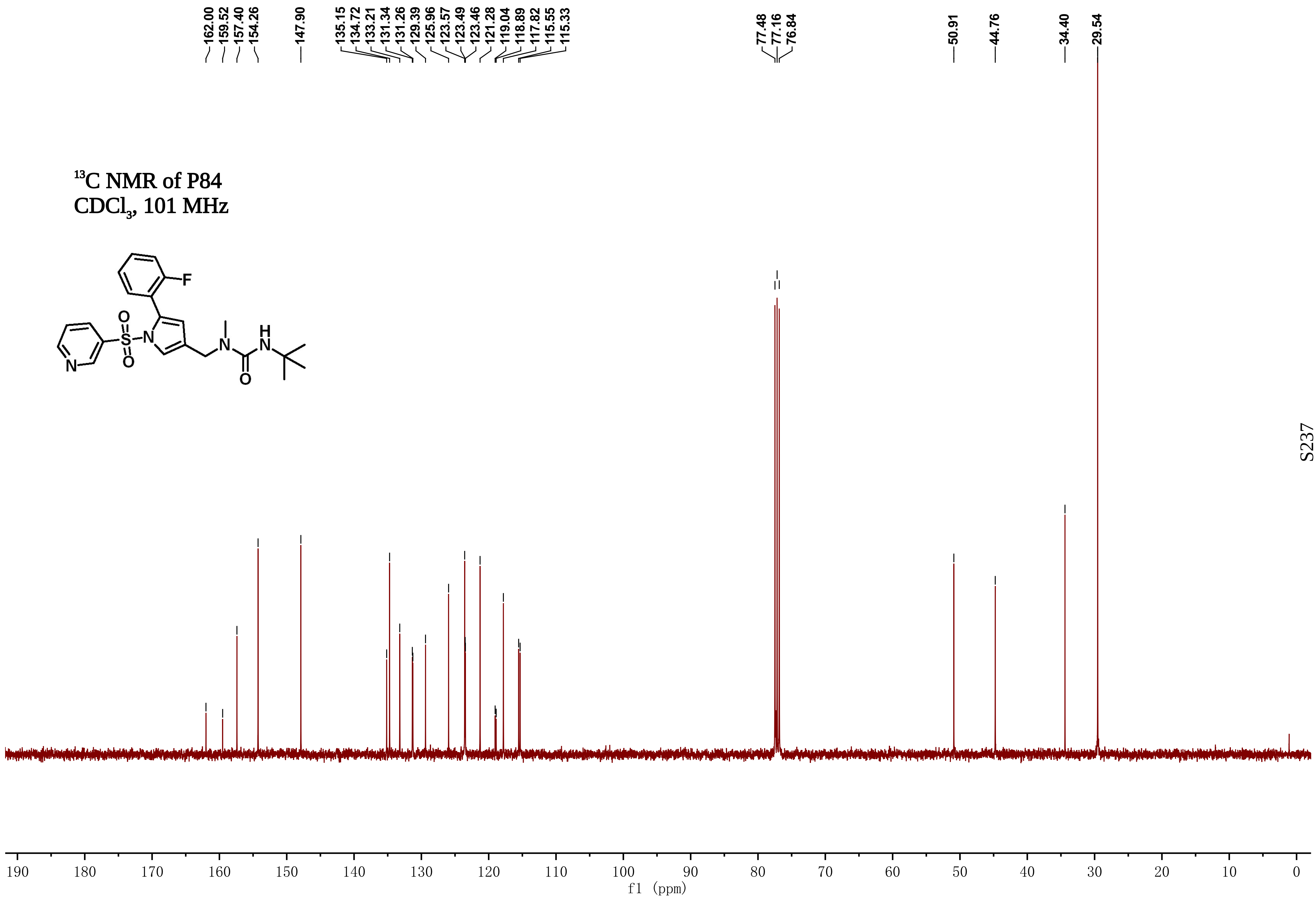

162.00
159.52
157.40
154.26
147.90
135.15
134.72
133.21
131.34
131.26
129.39
125.96
123.57
123.49
123.46
121.28
119.04
118.89
117.82
115.55
115.33
77.48
77.16
76.84
50.91
44.76
34.40
29.54
¹³C NMR of P84
CDCl₃, 101 MHz
F
O
S
N
O
N
N
H
N
O
190 180 170 160 150 140 130 120 110 100 90 80 70 60 50 40 30 20 10 0
f1 (ppm)

^{19}F NMR of P84
$CDCl_3$, 376 MHz

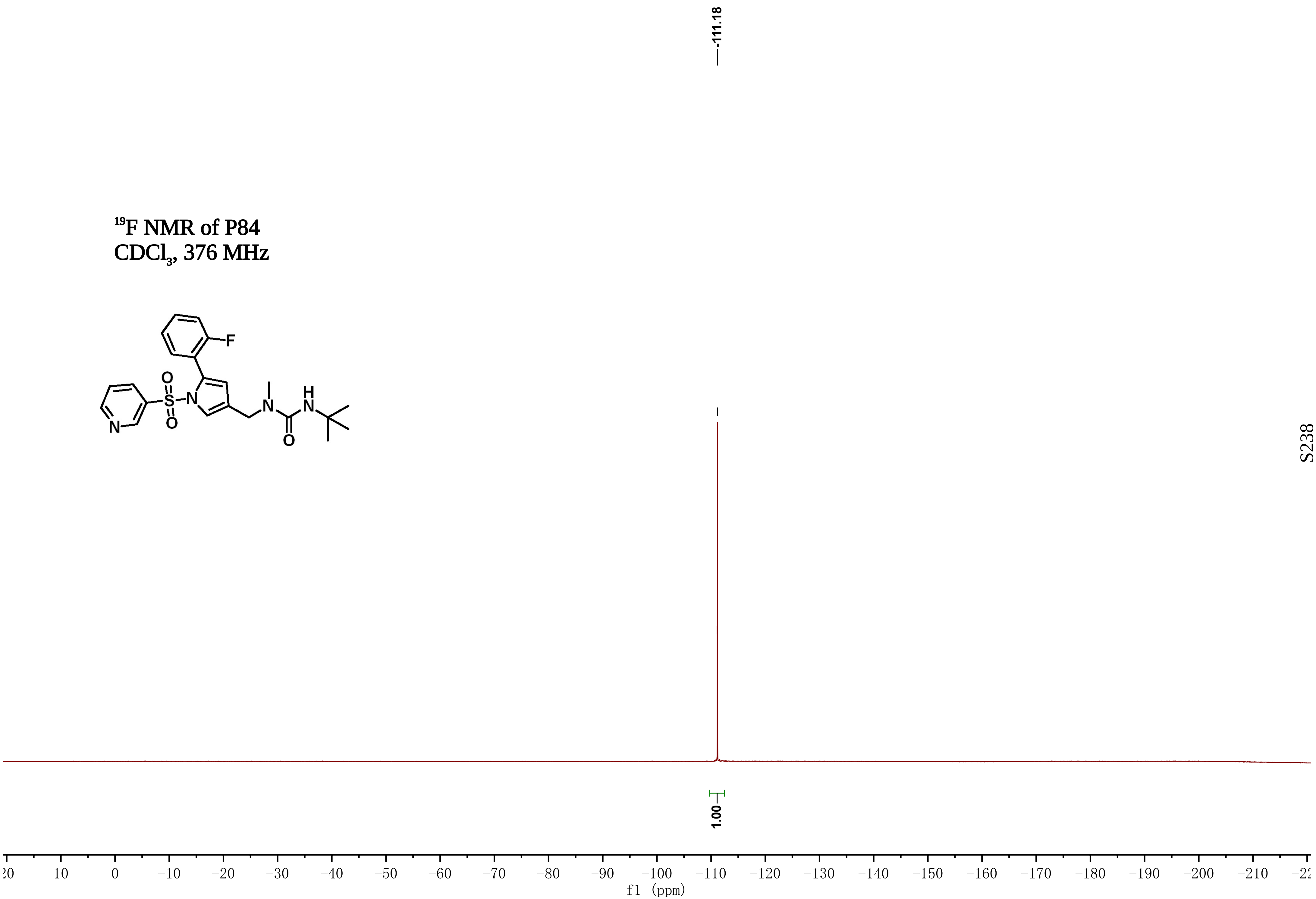

^{1}H NMR of P85
$CDCl_3$, 400 MHz

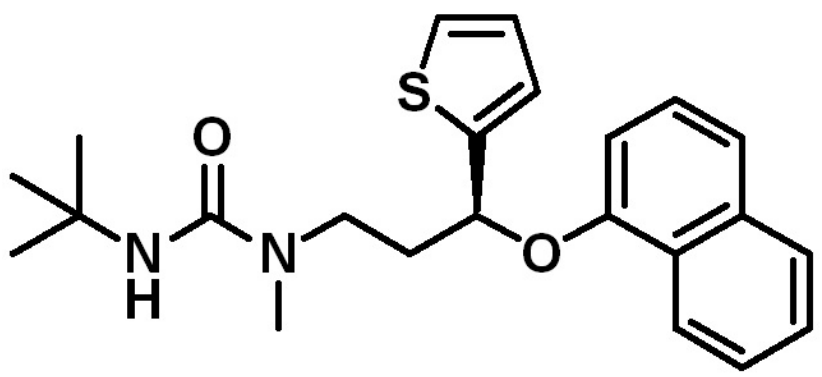

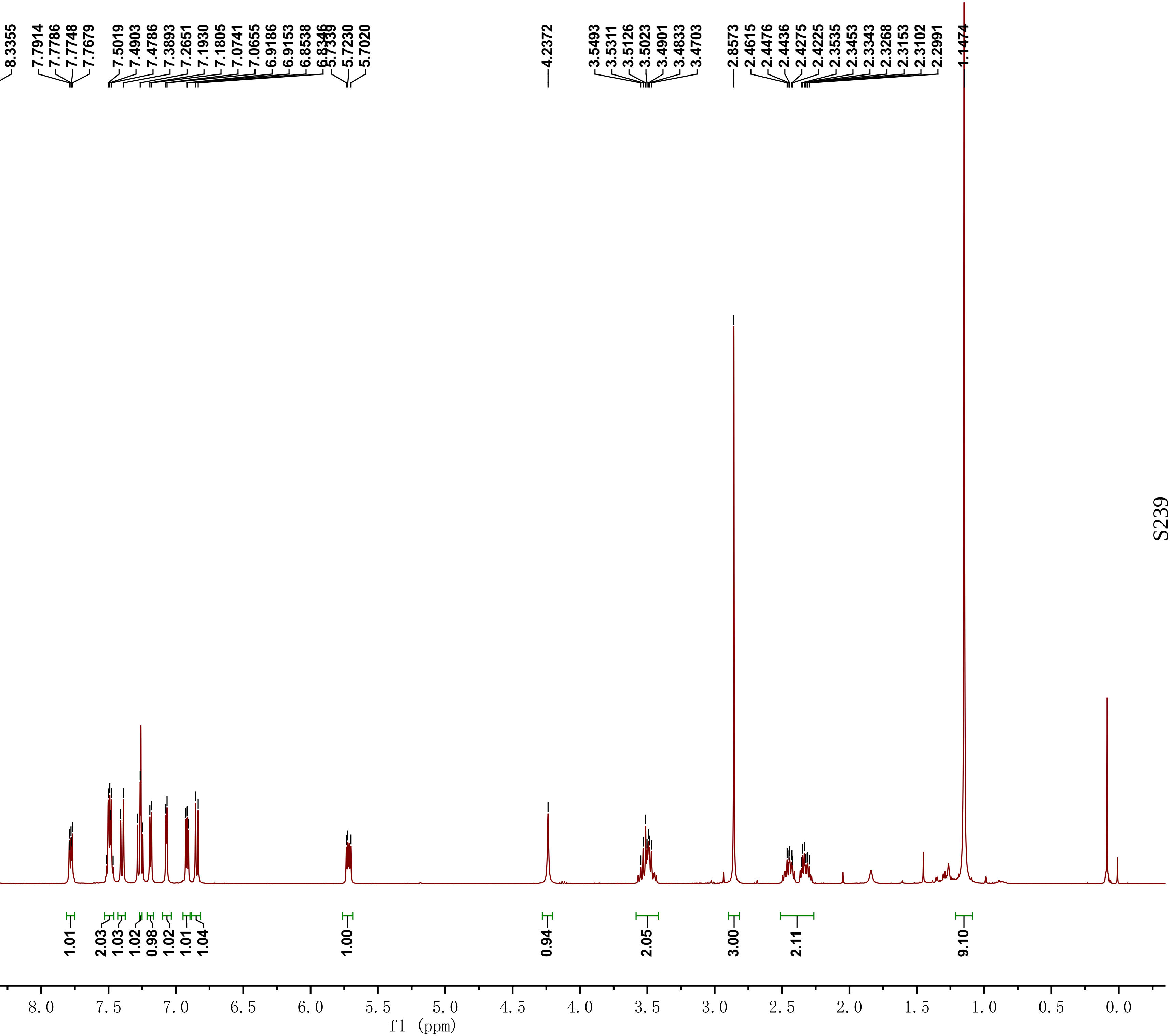

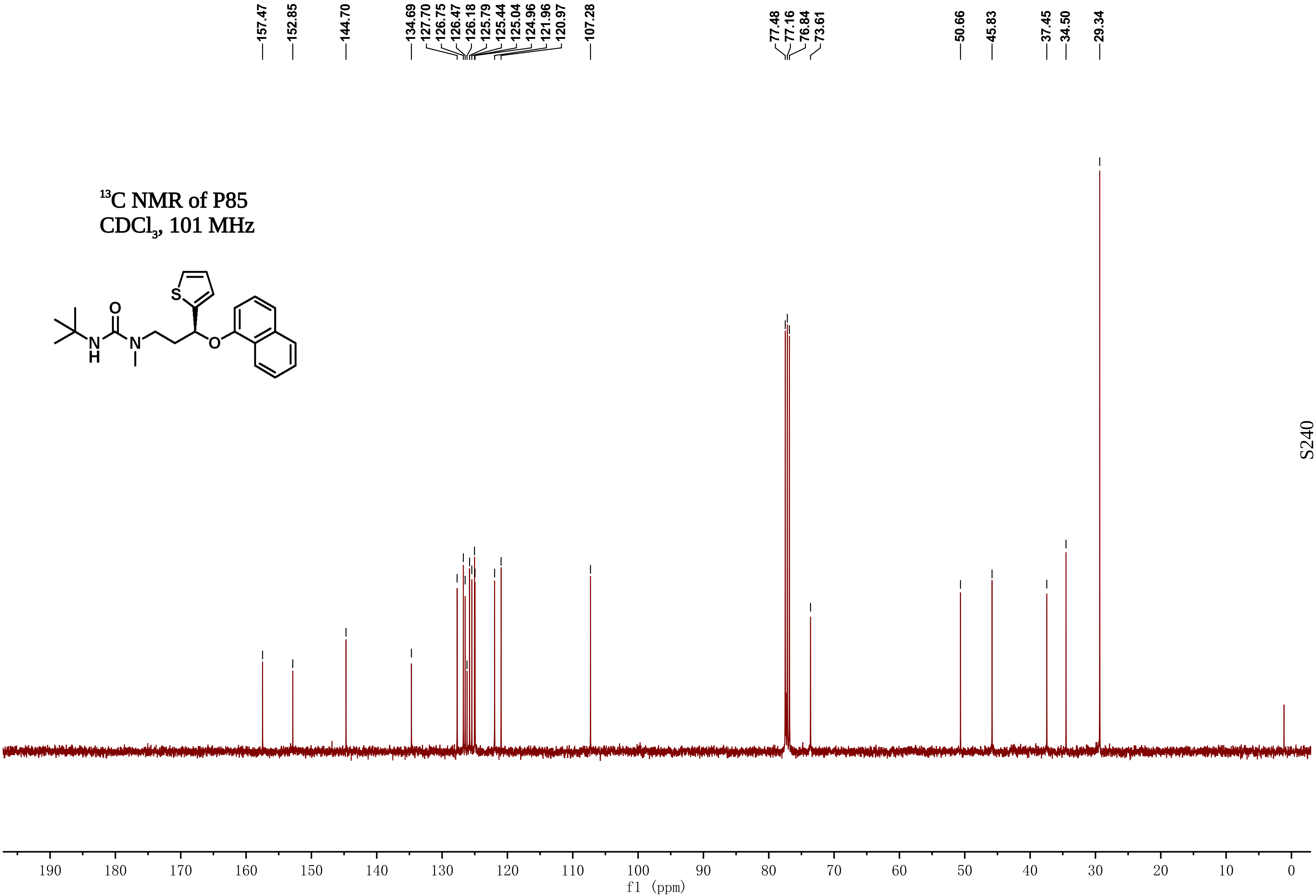

157.47
152.85
144.70
134.69
127.70
126.75
126.47
126.18
125.79
125.44
125.04
124.96
121.96
120.97
107.28
77.48
77.16
76.84
73.61
50.66
45.83
37.45
34.50
29.34
13C NMR of P85
CDCl3, 101 MHz
O
N
H
N
S
O
190 180 170 160 150 140 130 120 110 100 90 80 70 60 50 40 30 20 10 0
f1 (ppm)

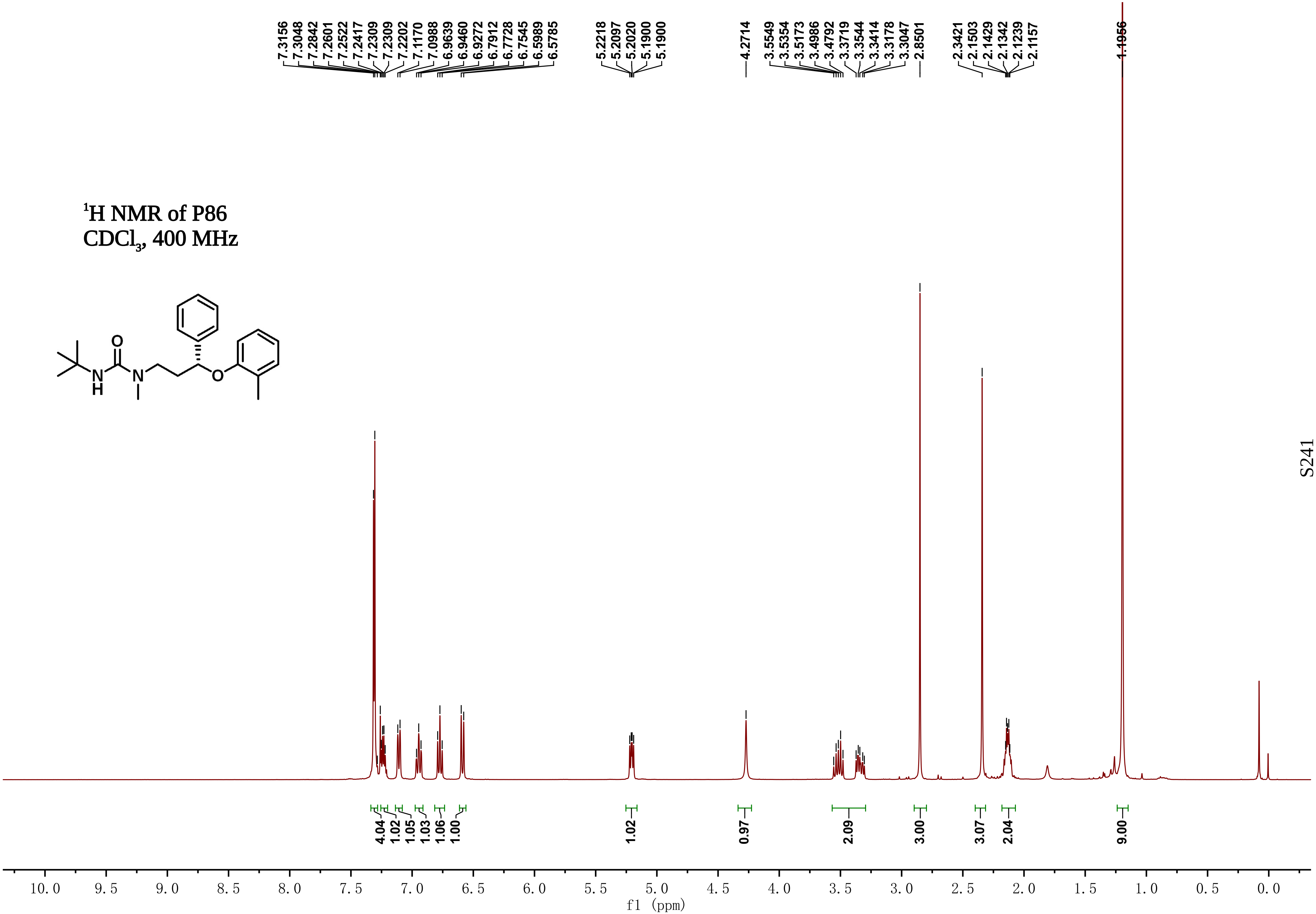

7.3156
7.3048
7.2842
7.2601
7.2522
7.2417
7.2309
7.2309
7.2202
7.1170
7.0988
6.9639
6.9460
6.9272
6.7912
6.7728
6.7545
6.5989
6.5785
5.2218
5.2097
5.2020
5.1900
5.1900
4.2714
3.5549
3.5354
3.5173
3.4986
3.4792
3.3719
3.3544
3.3414
3.3178
3.3047
2.8501
2.3421
2.1503
2.1429
2.1342
2.1239
2.1157
1.1956
1H NMR of P86
CDCl3, 400 MHz
O
N
H
N
O
4.04
1.02
1.05
1.03
1.06
1.00
1.02
0.97
2.09
3.00
3.07
2.04
9.00
10.0
9.5
9.0
8.5
8.0
7.5
7.0
6.5
6.0
5.5
5.0
4.5
4.0
3.5
3.0
2.5
2.0
1.5
1.0
0.5
0.0
f1 (ppm)

^{13}C NMR of P86
$CDCl_3$, 101 MHz

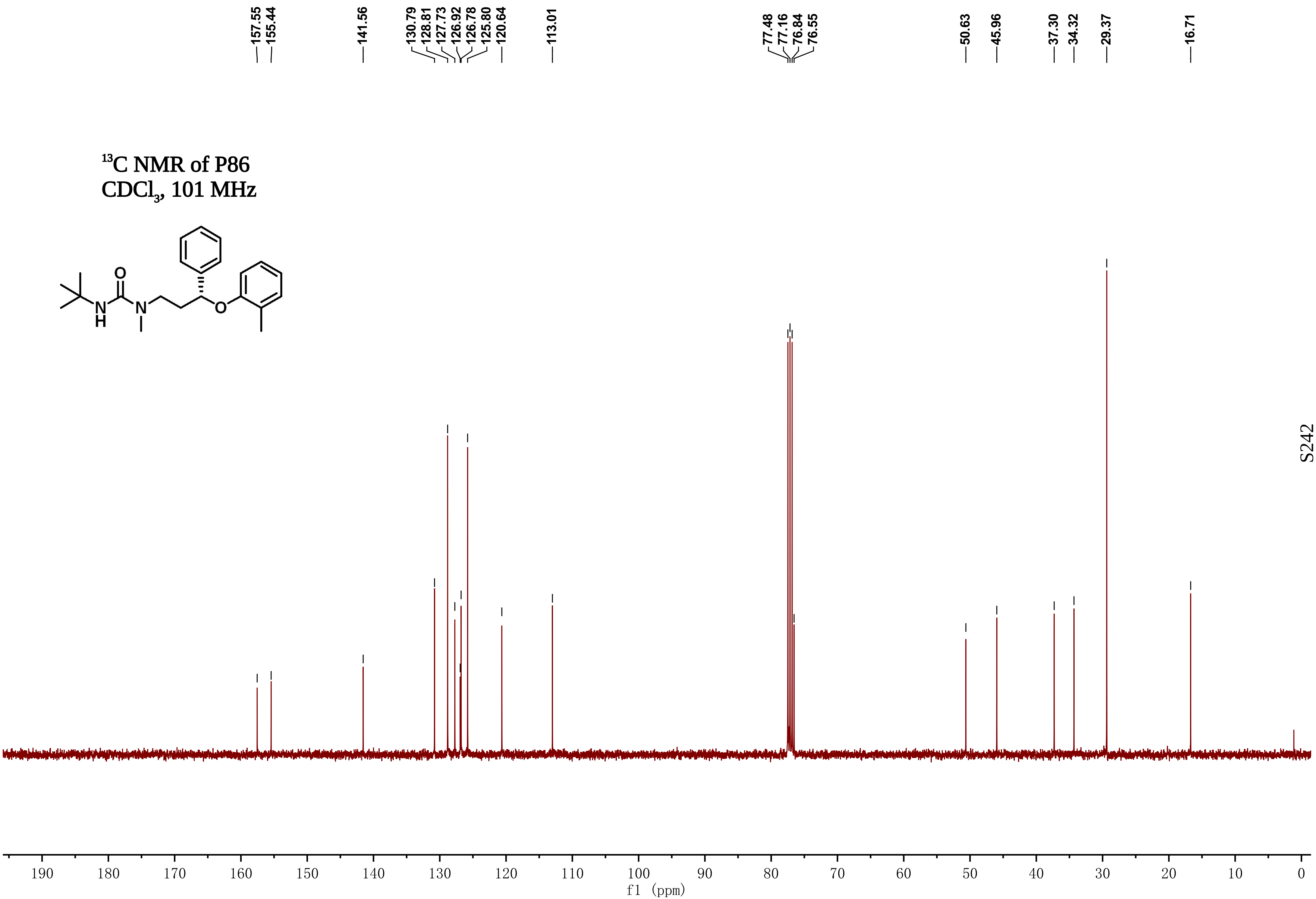

^{1}H NMR of P87
$CDCl_3$, 400 MHz

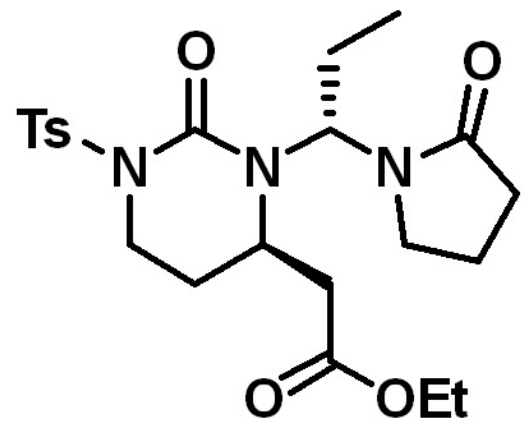

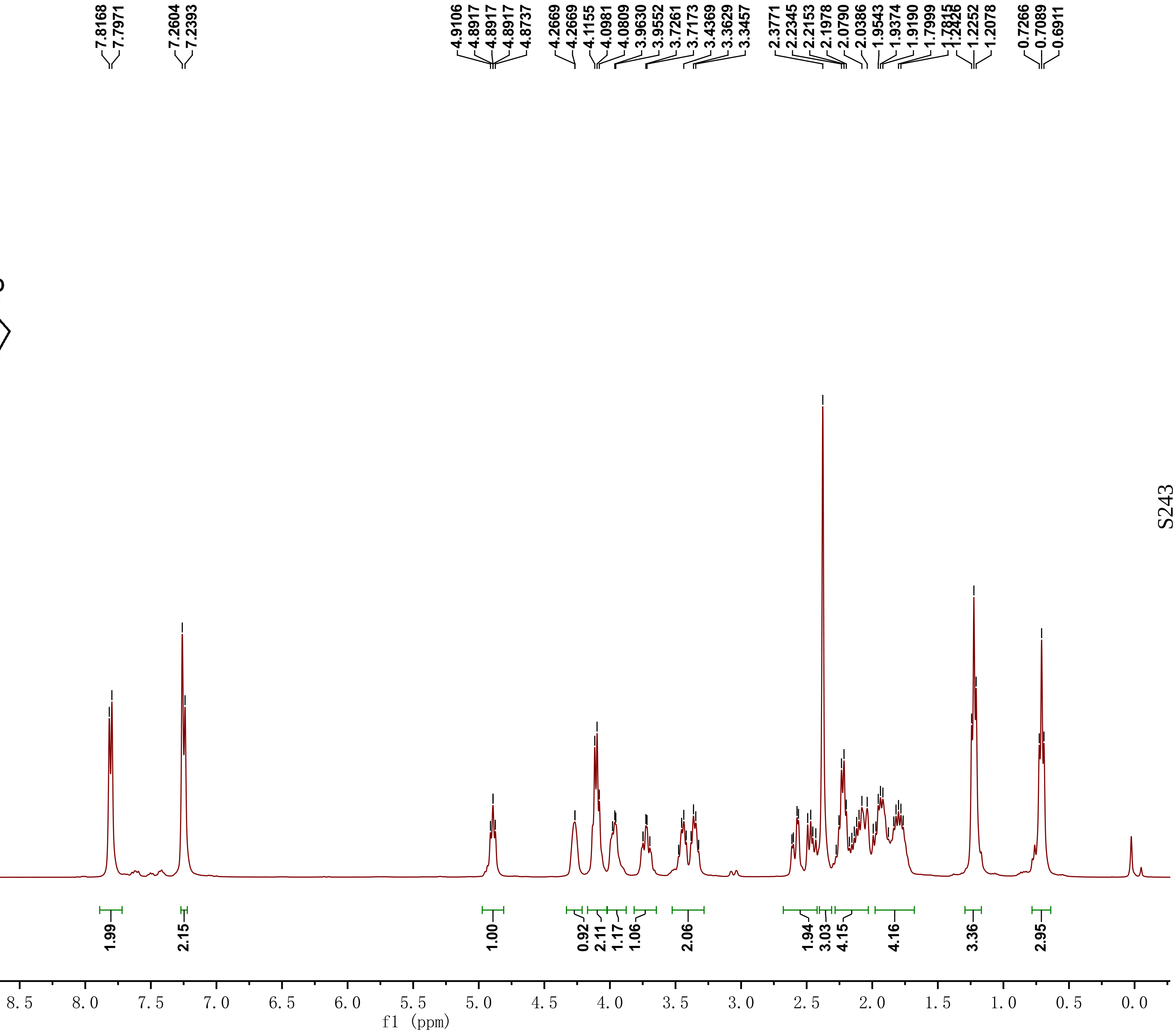

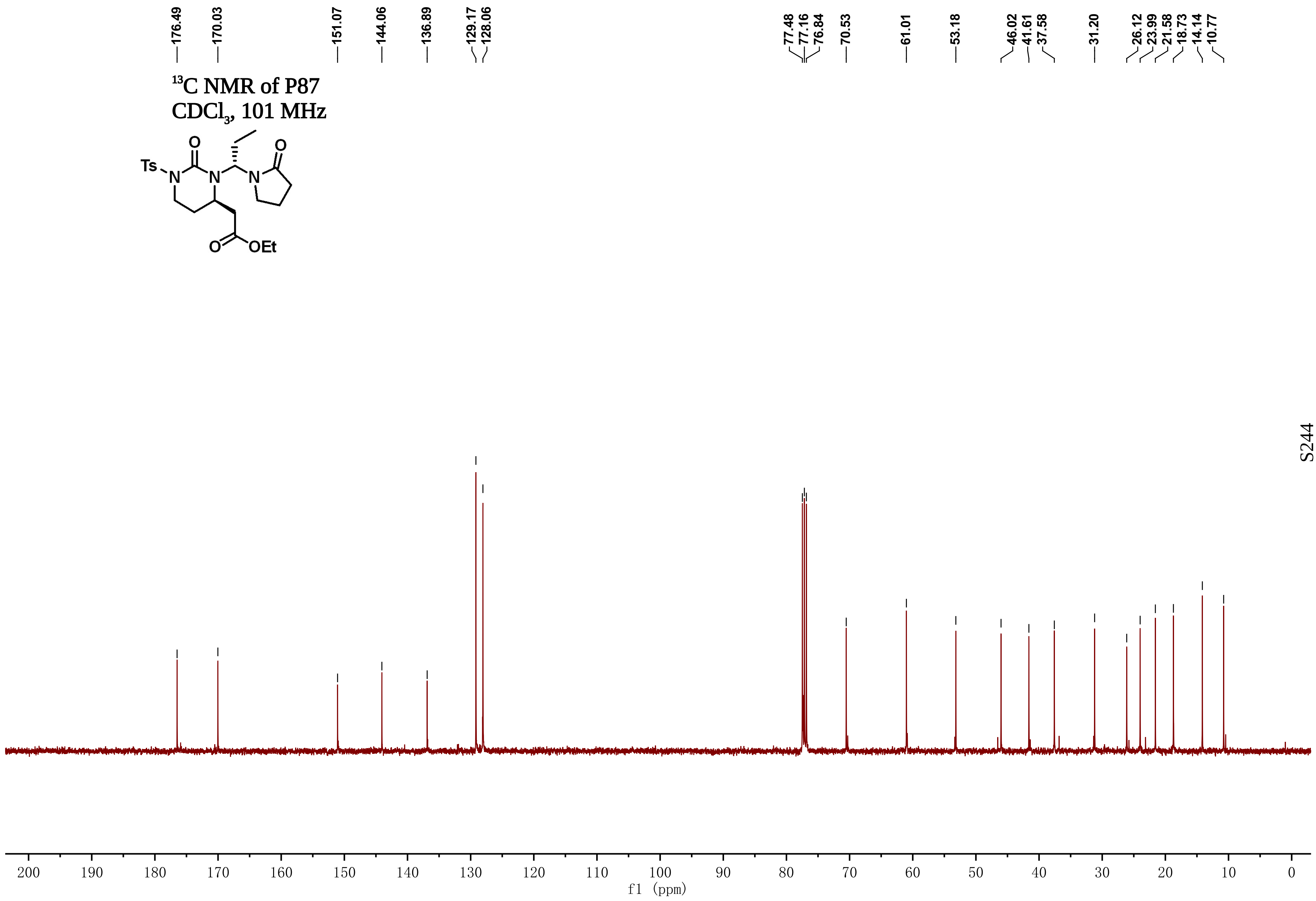

176.49
170.03
151.07
144.06
136.89
129.17
128.06
77.48
77.16
76.84
70.53
61.01
53.18
46.02
41.61
37.58
31.20
26.12
23.99
21.58
18.73
14.14
10.77
^{13}C NMR of P87
$CDCl_3$, 101 MHz
Ts
N
O
N
N
O
O
OEt
200 190 180 170 160 150 140 130 120 110 100 90 80 70 60 50 40 30 20 10 0
f1 (ppm)

^{1}H NMR of P88
$CDCl_3$, 400 MHz

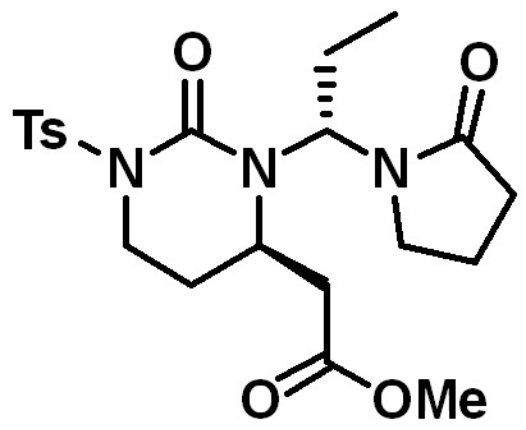

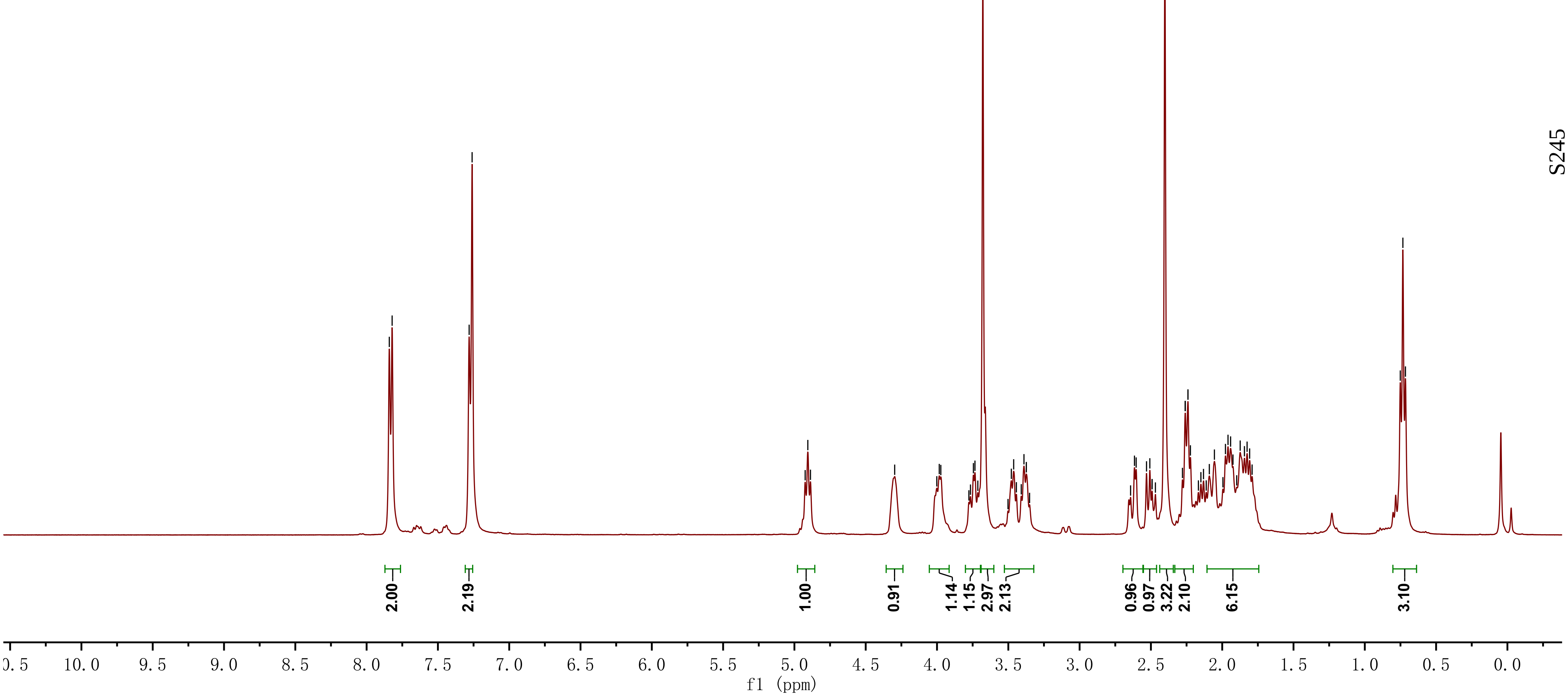

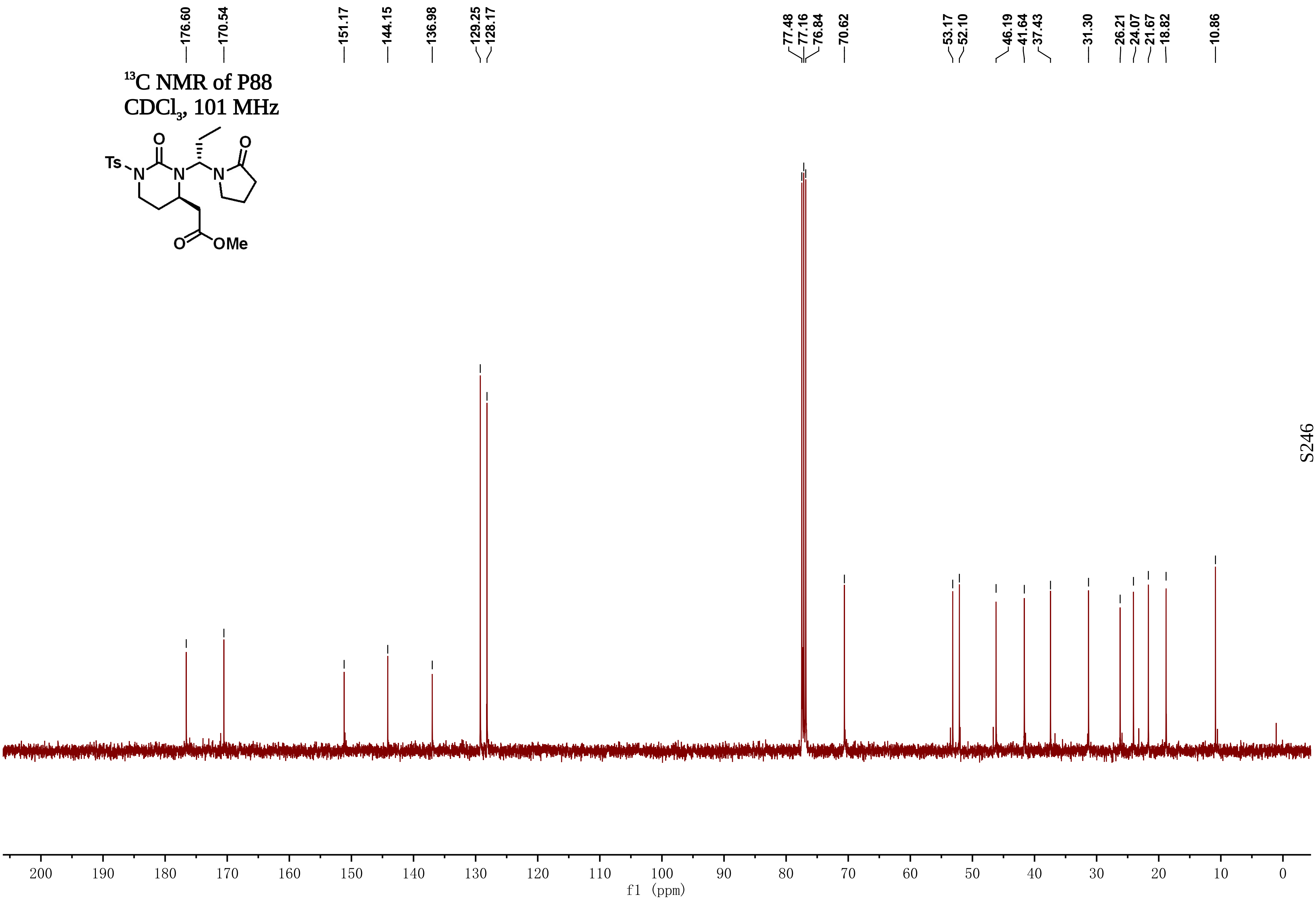

176.60
170.54
151.17
144.15
136.98
129.25
128.17
77.48
77.16
76.84
70.62
53.17
52.10
46.19
41.64
37.43
31.30
26.21
24.07
21.67
18.82
10.86
^{13}C NMR of P88
$CDCl_3$, 101 MHz
Ts
N
O
N
N
O
O
OMe
200 190 180 170 160 150 140 130 120 110 100 90 80 70 60 50 40 30 20 10 0
f1 (ppm)

^{1}H NMR of P89
$CDCl_3$, 400 MHz

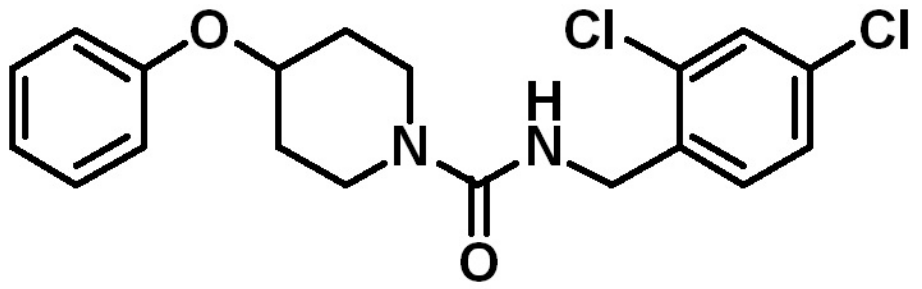

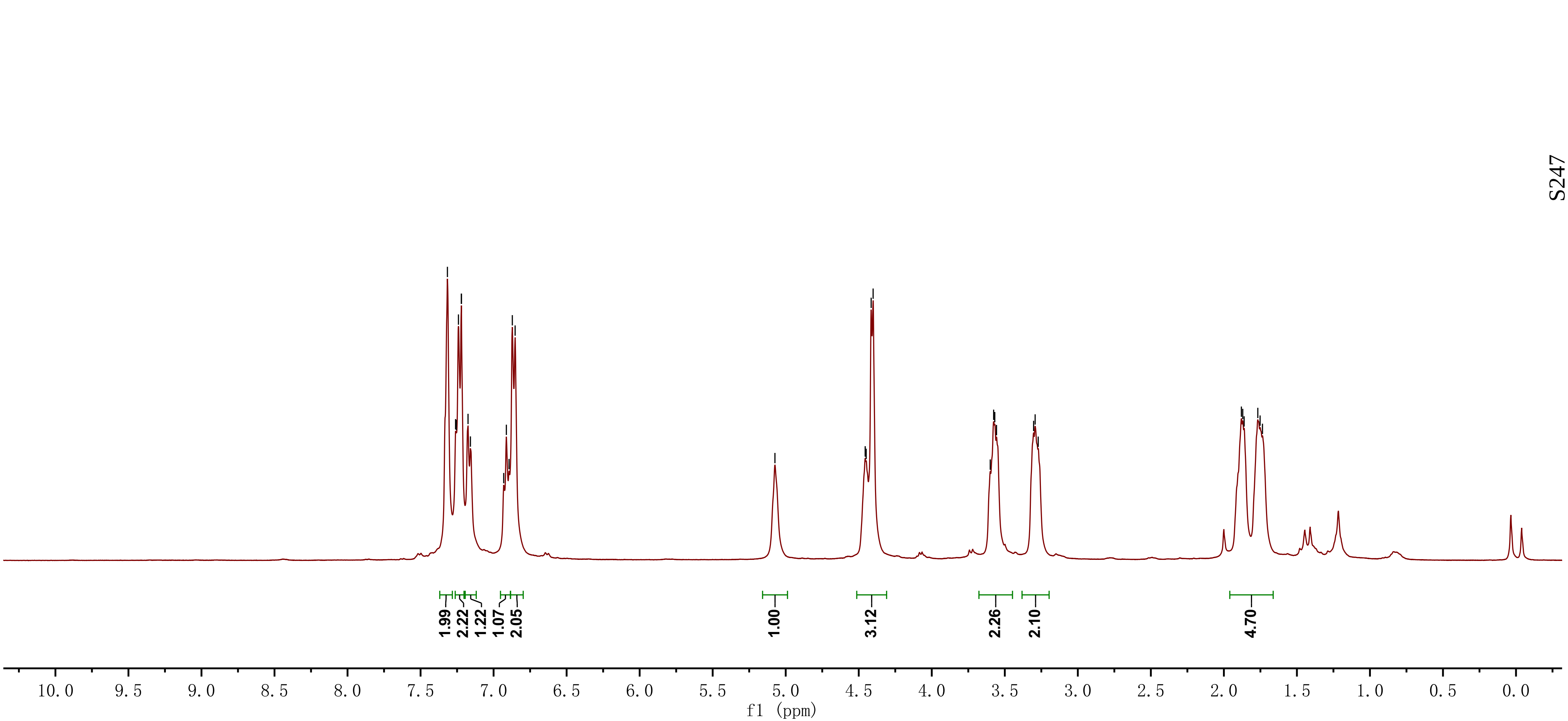

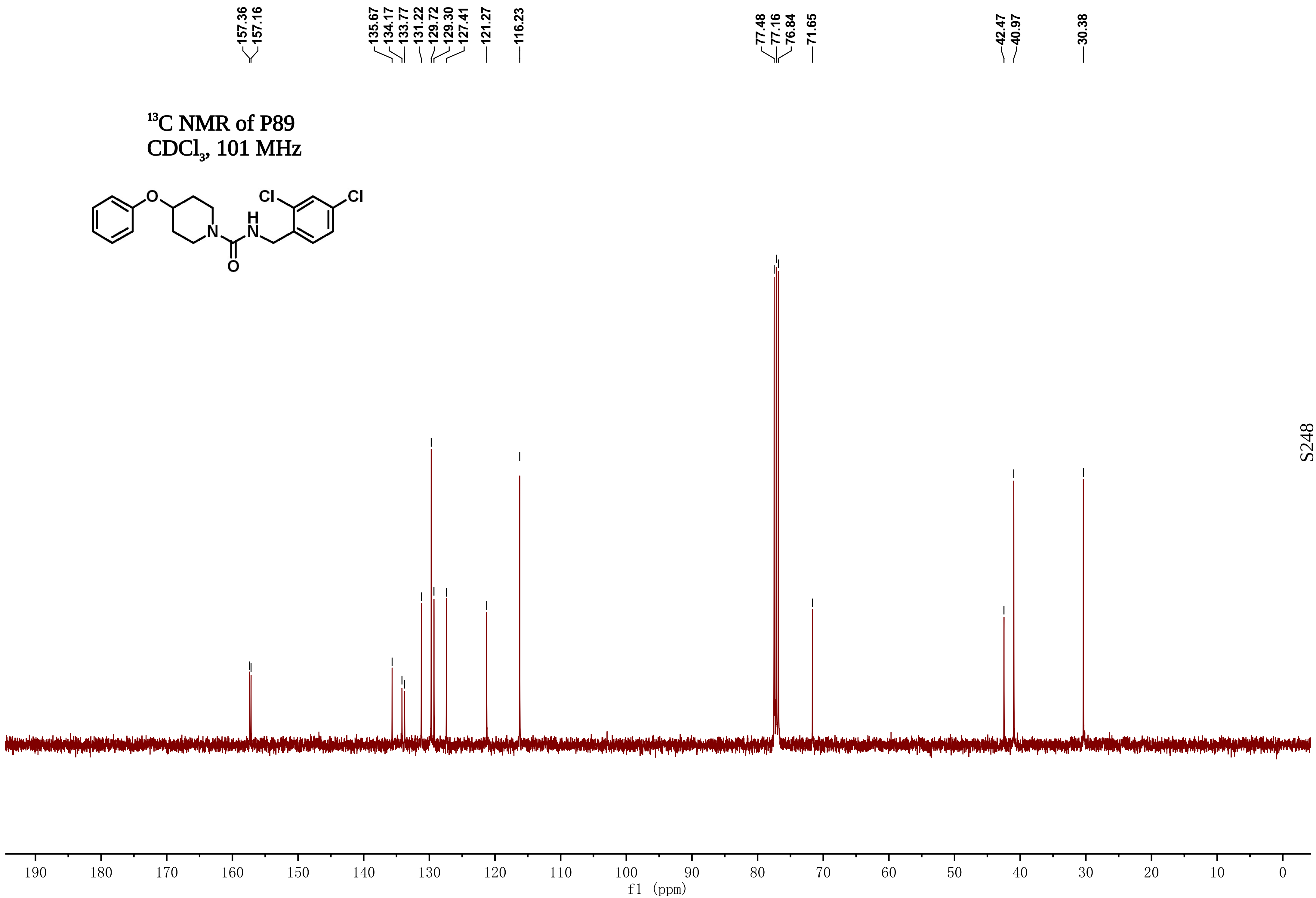
157.36
157.16
135.67
134.17
133.77
131.22
129.72
129.30
127.41
121.27
116.23
77.48
77.16
76.84
71.65
42.47
40.97
30.38
13C NMR of P89
CDCl3, 101 MHz
O
N
H
N
O
Cl
Cl
190 180 170 160 150 140 130 120 110 100 90 80 70 60 50 40 30 20 10 0
f1 (ppm)

^{1}H NMR of P90
$CDCl_3$, 400 MHz

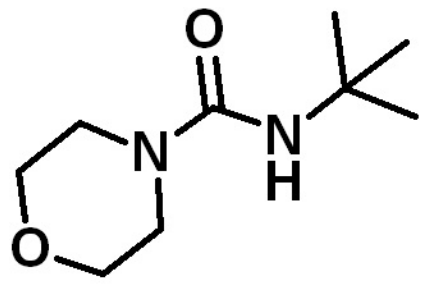

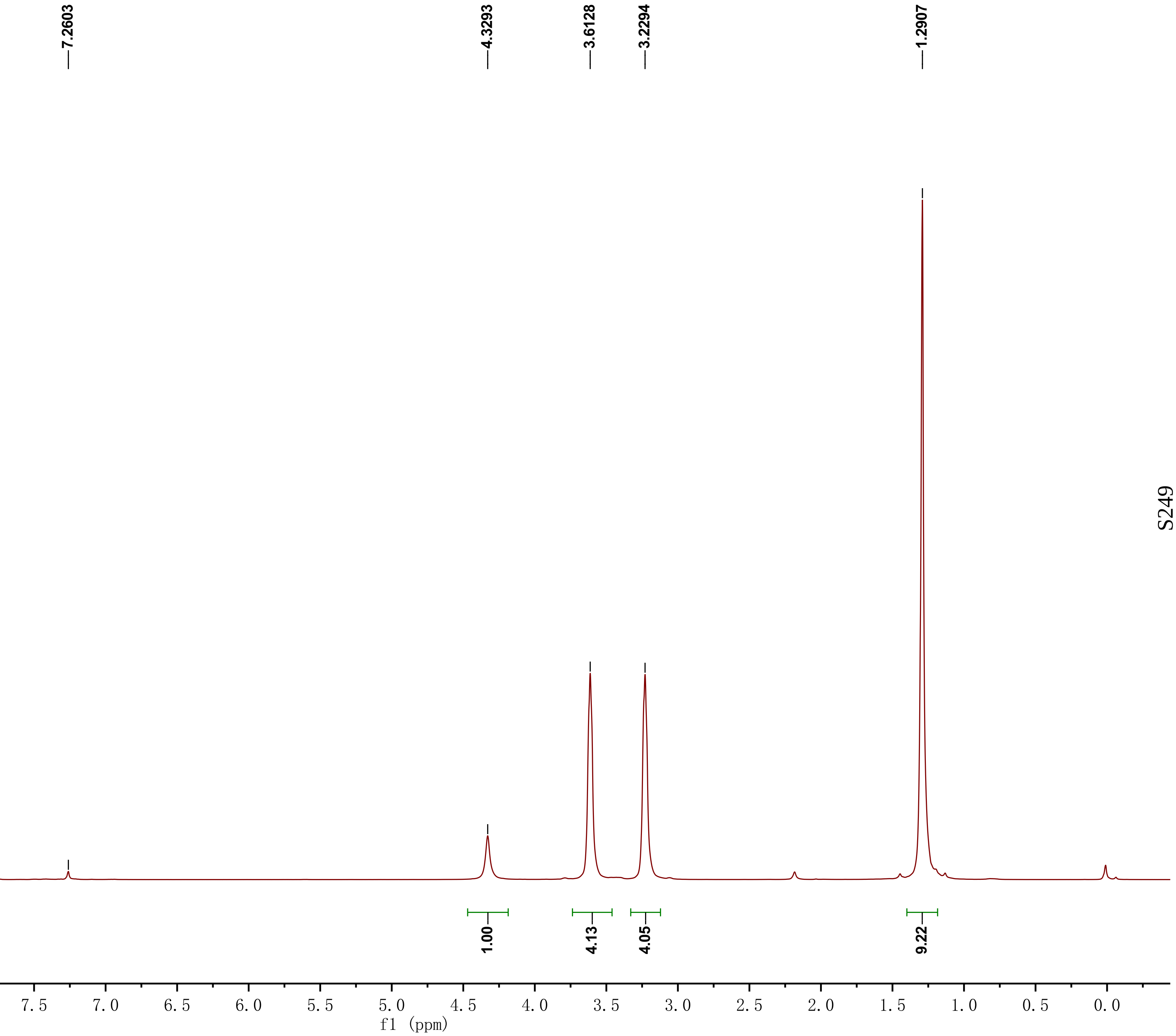

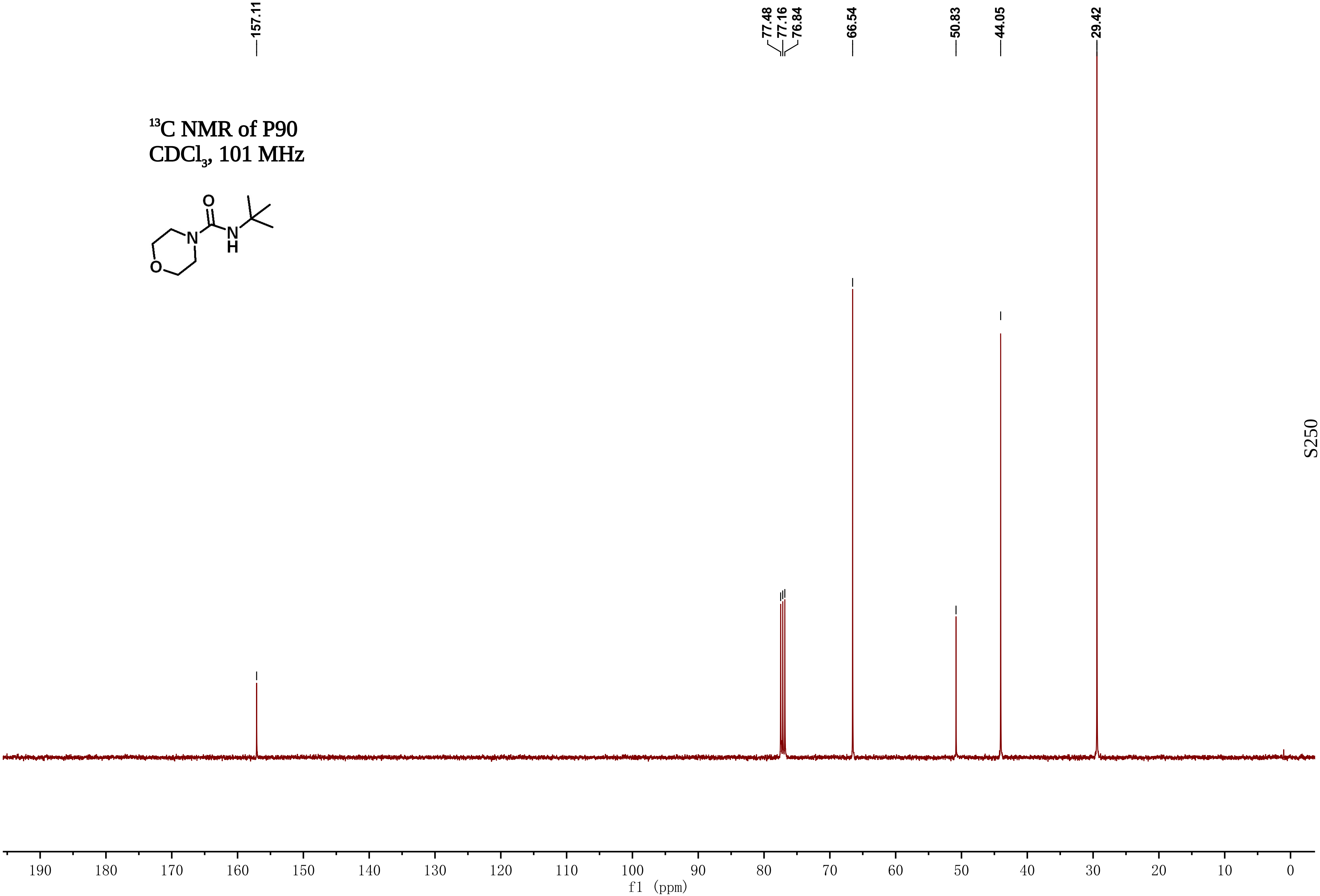

157.11
77.48
77.16
76.84
66.54
50.83
44.05
29.42
^{13}C NMR of P90
$CDCl_3$, 101 MHz
O
N
N
H
O
190 180 170 160 150 140 130 120 110 100 90 80 70 60 50 40 30 20 10 0
f1 (ppm)

^{1}H NMR of P91
CD_3OD, 400 MHz

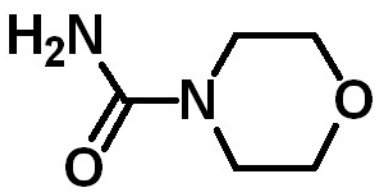

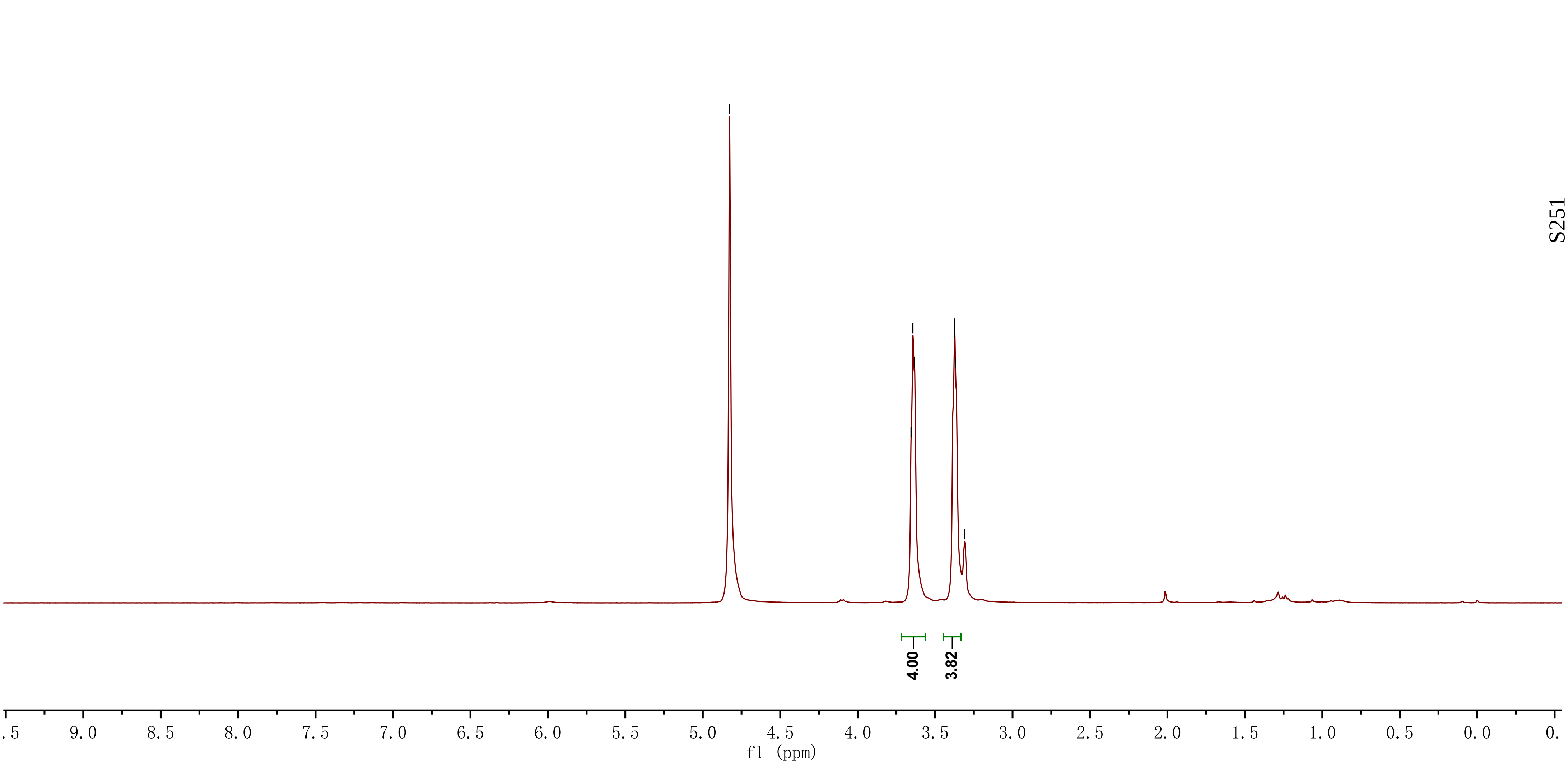

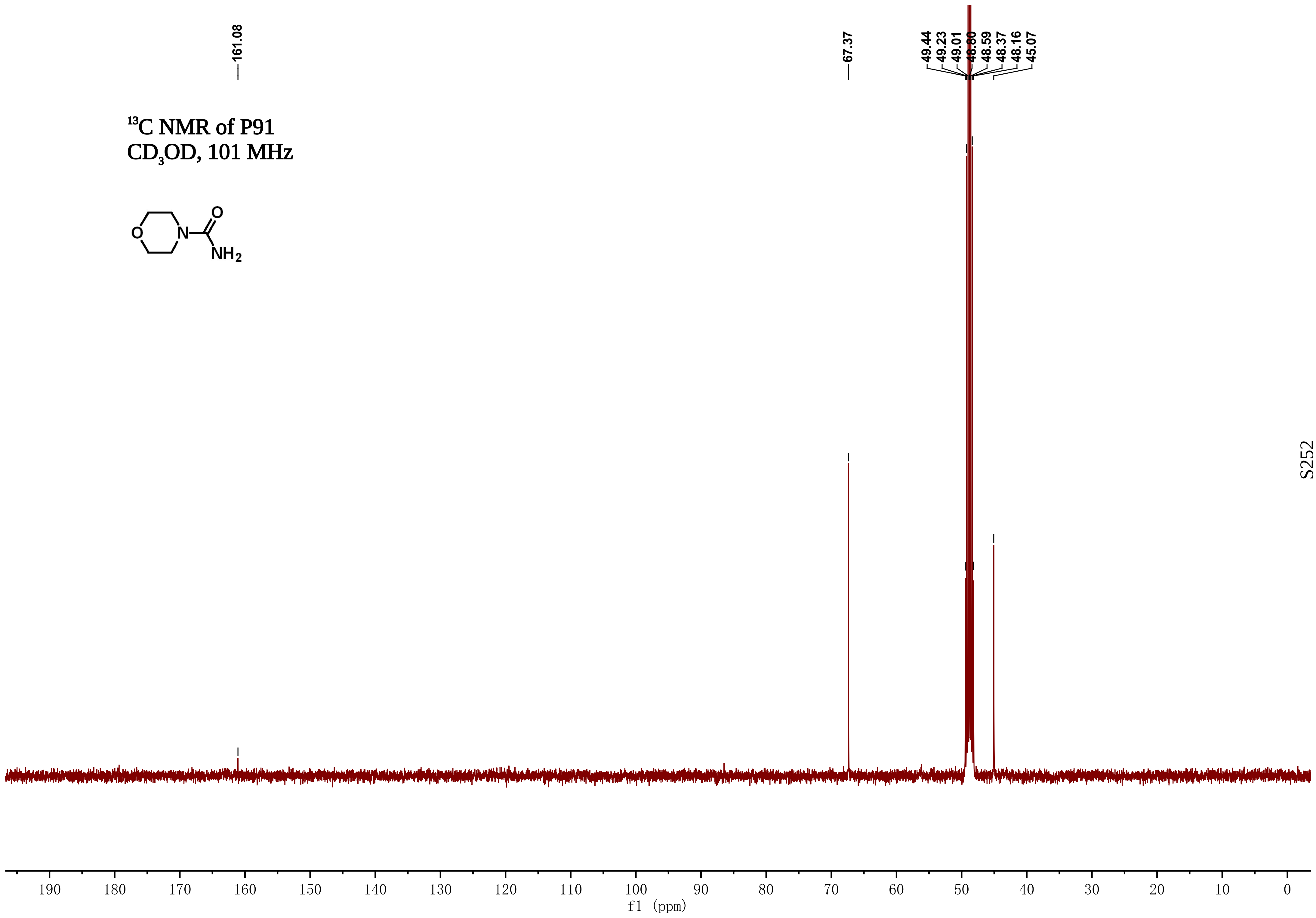

^{13}C NMR of P91
CD$_3$OD, 101 MHz
161.08
67.37
49.44
49.23
49.01
48.80
48.59
48.37
48.16
45.07
O
N
NH$_2$
190 180 170 160 150 140 130 120 110 100 90 80 70 60 50 40 30 20 10 0
f1 (ppm)

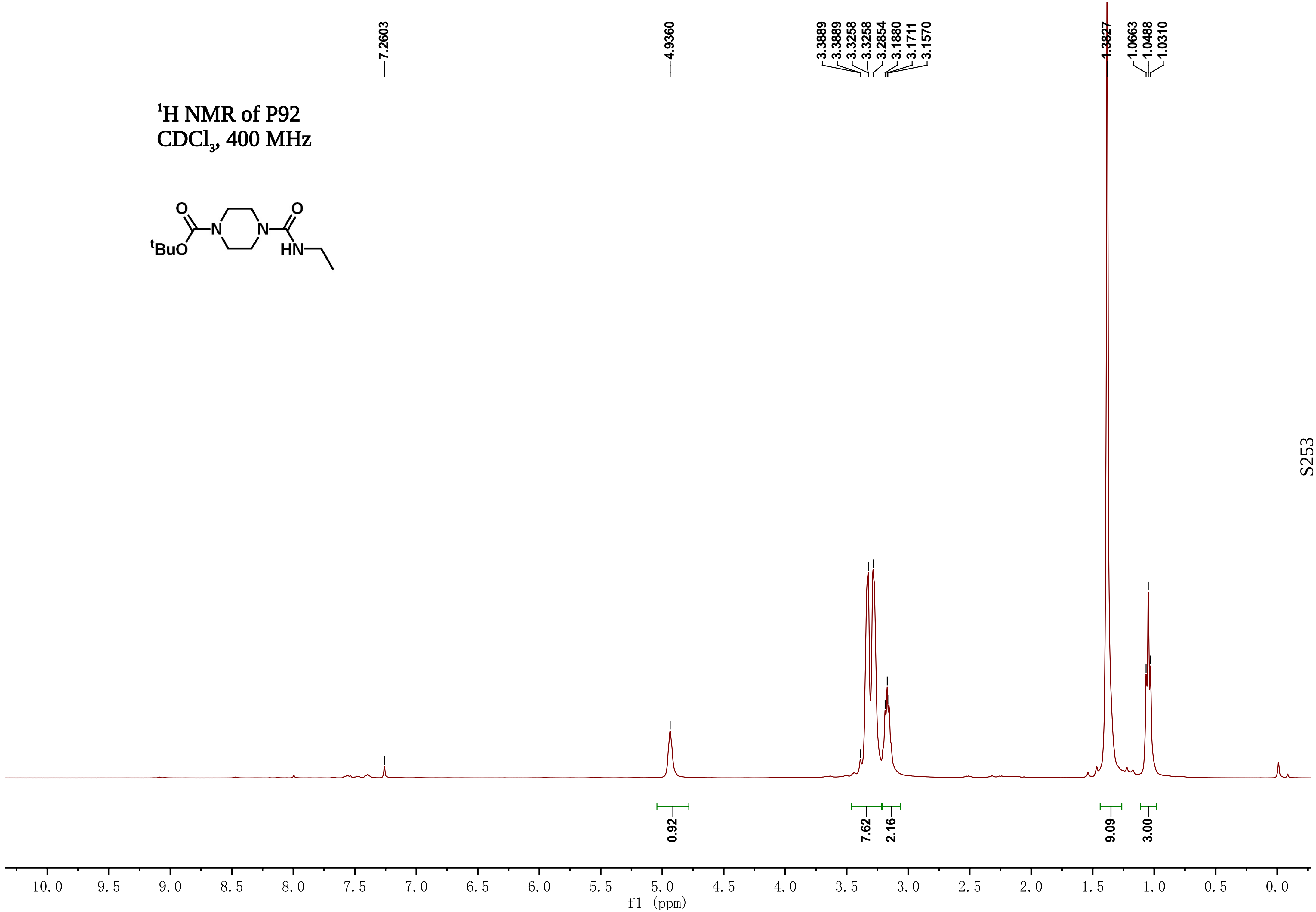

^{1}H NMR of P92
$CDCl_3$, 400 MHz
O
O
N
N
^{t}BuO
HN
7.2603
4.9360
3.3889
3.3889
3.3258
3.3258
3.2854
3.1880
3.1711
3.1570
1.3827
1.0663
1.0488
1.0310
0.92
7.62
2.16
9.09
3.00
10.0
9.5
9.0
8.5
8.0
7.5
7.0
6.5
6.0
5.5
5.0
4.5
4.0
3.5
3.0
2.5
2.0
1.5
1.0
0.5
0.0
f1 (ppm)

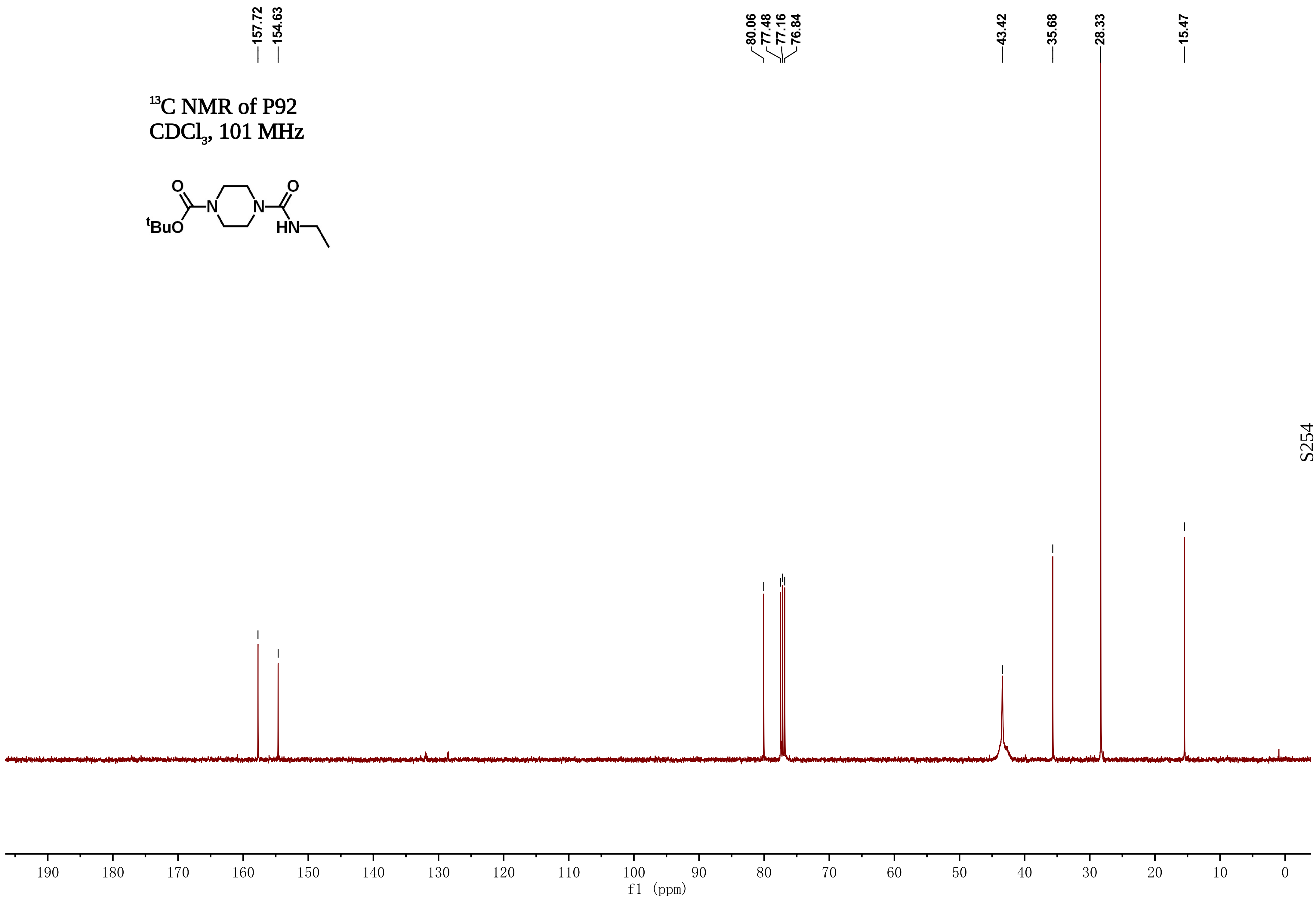

157.72
154.63
80.06
77.48
77.16
76.84
43.42
35.68
28.33
15.47
13C NMR of P92
CDCl3, 101 MHz
O
N
N
O
tBuO
HN
190
180
170
160
150
140
130
120
110
100
90
80
70
60
50
40
30
20
10
0
f1 (ppm)

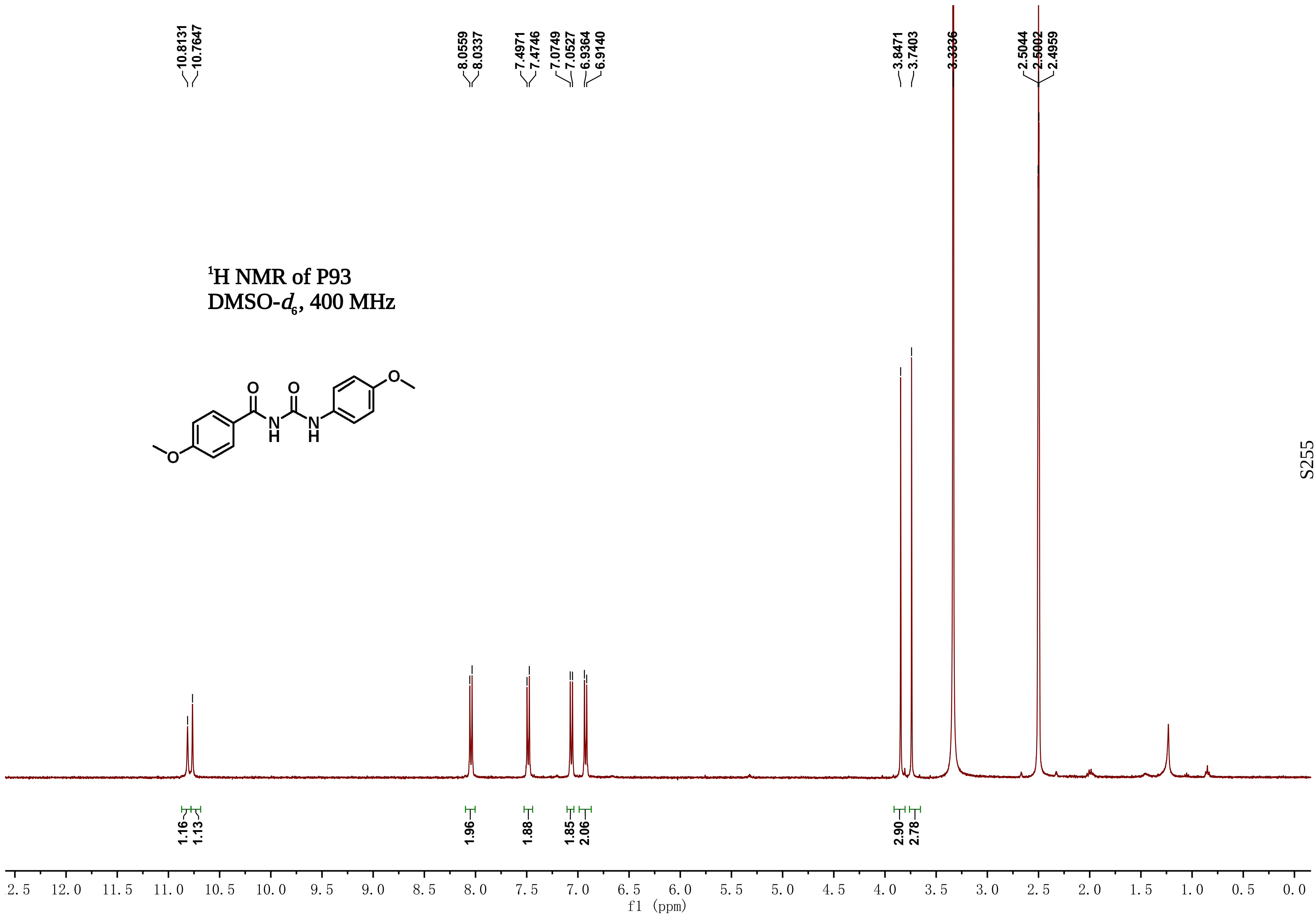
10.8131
10.7647
8.0559
8.0337
7.4971
7.4746
7.0749
7.0527
6.9364
6.9140
3.8471
3.7403
3.3336
2.5044
2.5002
2.4959
1H NMR of P93
DMSO-d6, 400 MHz
O
O
N
H
N
H
O
O
1.16
1.13
1.96
1.88
1.85
2.06
2.90
2.78
12.5 12.0 11.5 11.0 10.5 10.0 9.5 9.0 8.5 8.0 7.5 7.0 6.5 6.0 5.5 5.0 4.5 4.0 3.5 3.0 2.5 2.0 1.5 1.0 0.5 0.0
f1 (ppm)

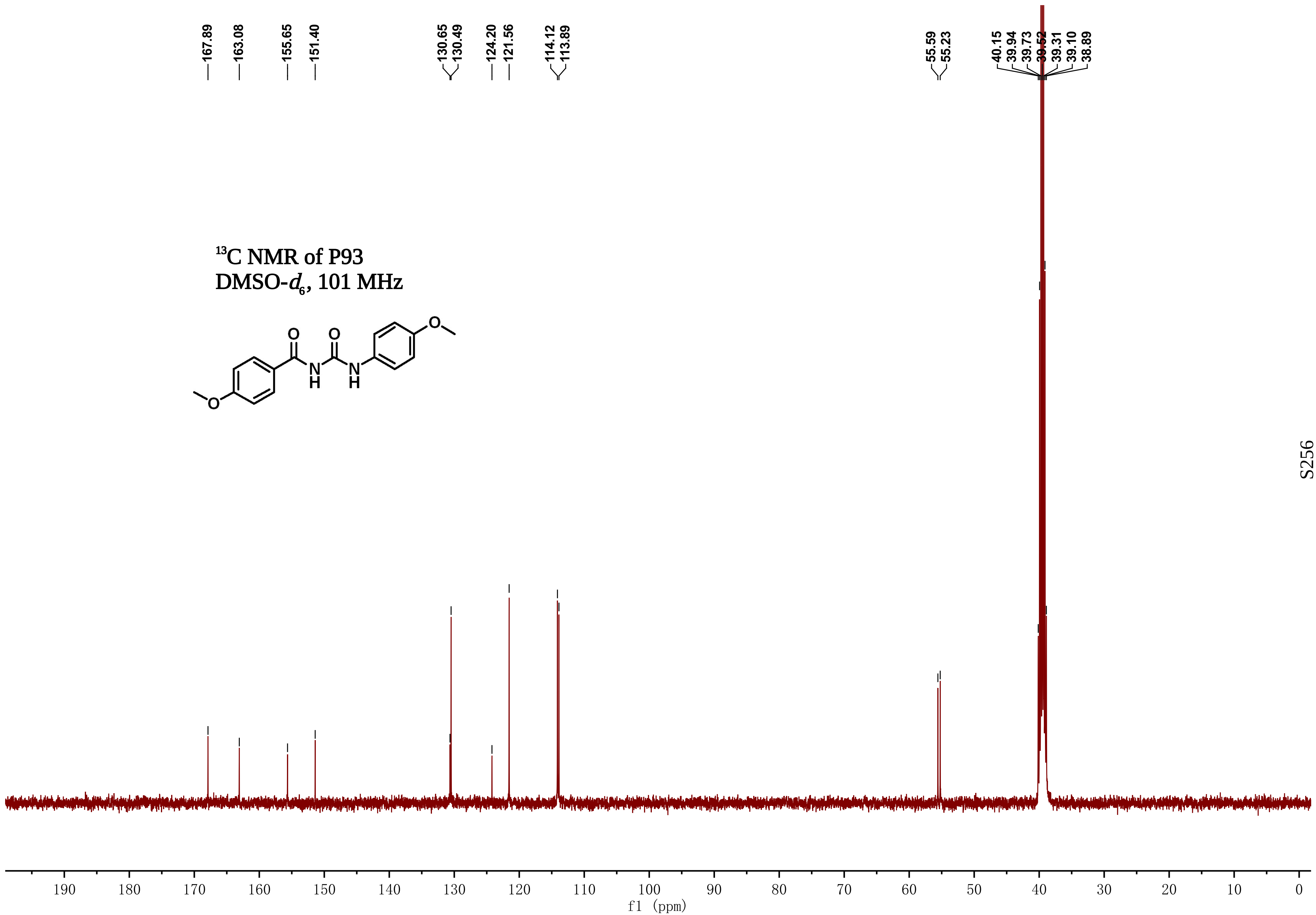
167.89
163.08
155.65
151.40
130.65
130.49
124.20
121.56
114.12
113.89
55.59
55.23
40.15
39.94
39.73
39.52
39.31
39.10
38.89
^{13}C NMR of P93
DMSO-d_6, 101 MHz
O
O
N
H
N
H
O
O
190
180
170
160
150
140
130
120
110
100
90
80
70
60
50
40
30
20
10
0
f1 (ppm)

^{1}H NMR of P94
$CDCl_3$, 400 MHz

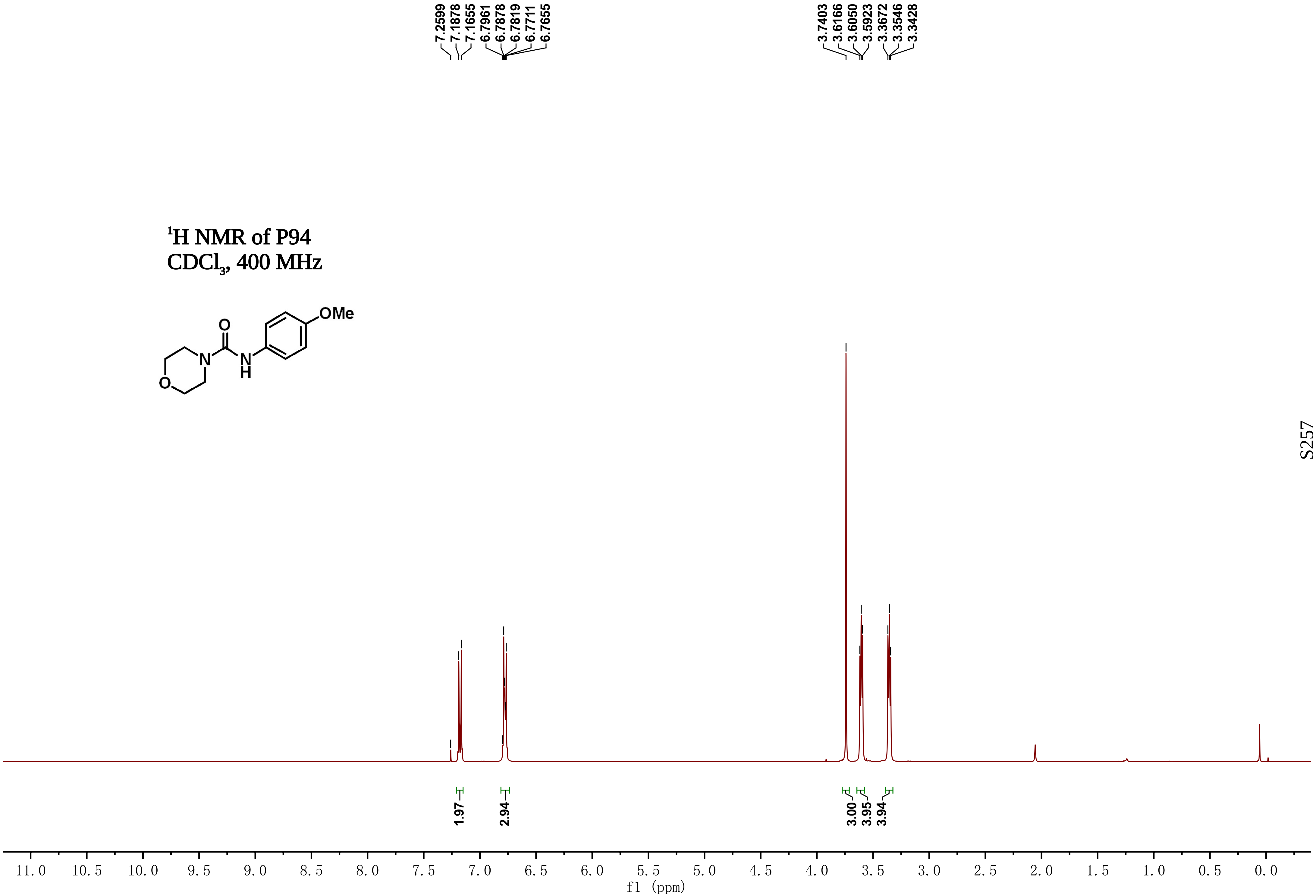

^{13}C NMR of P94
$CDCl_3$, 101 MHz

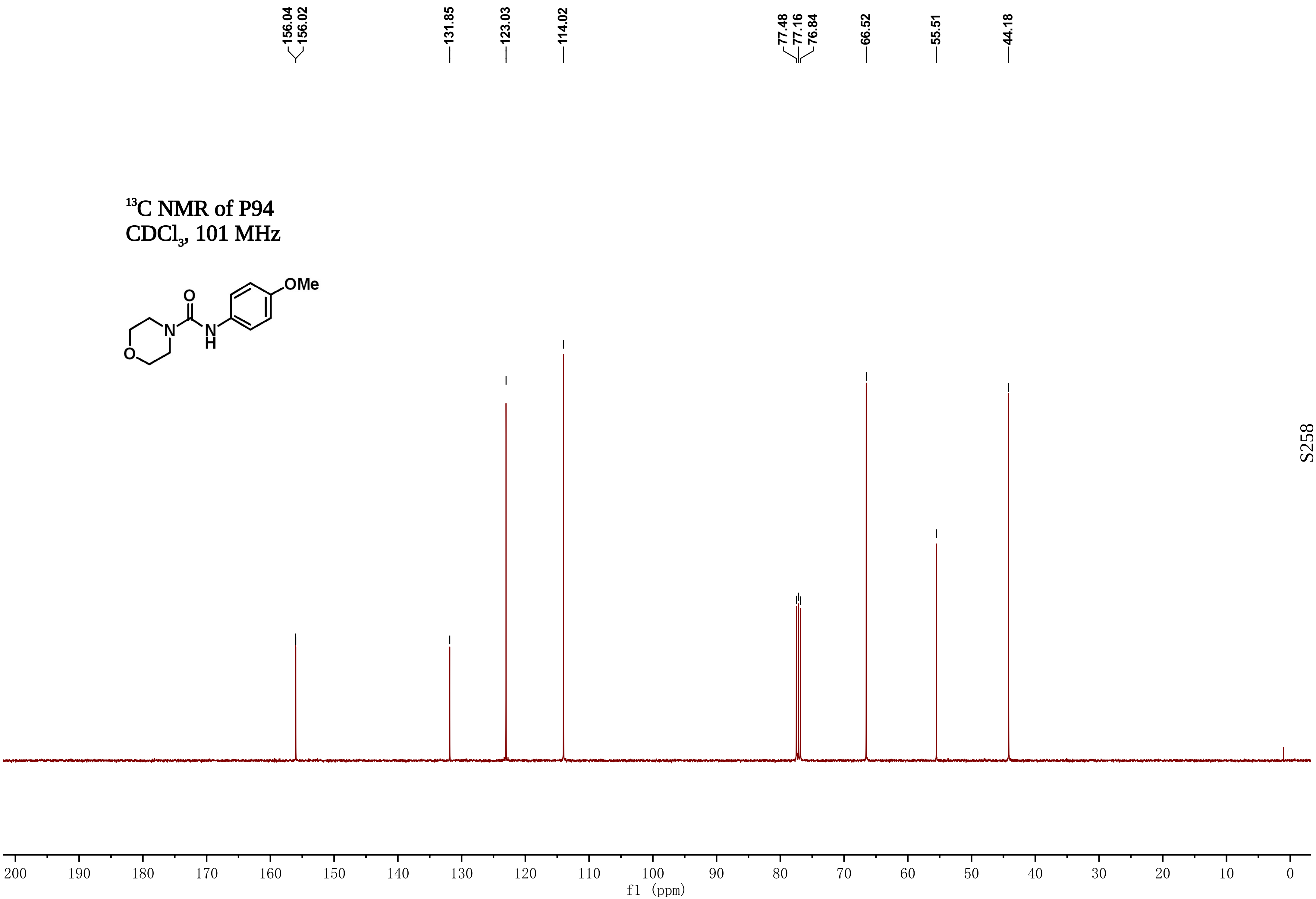

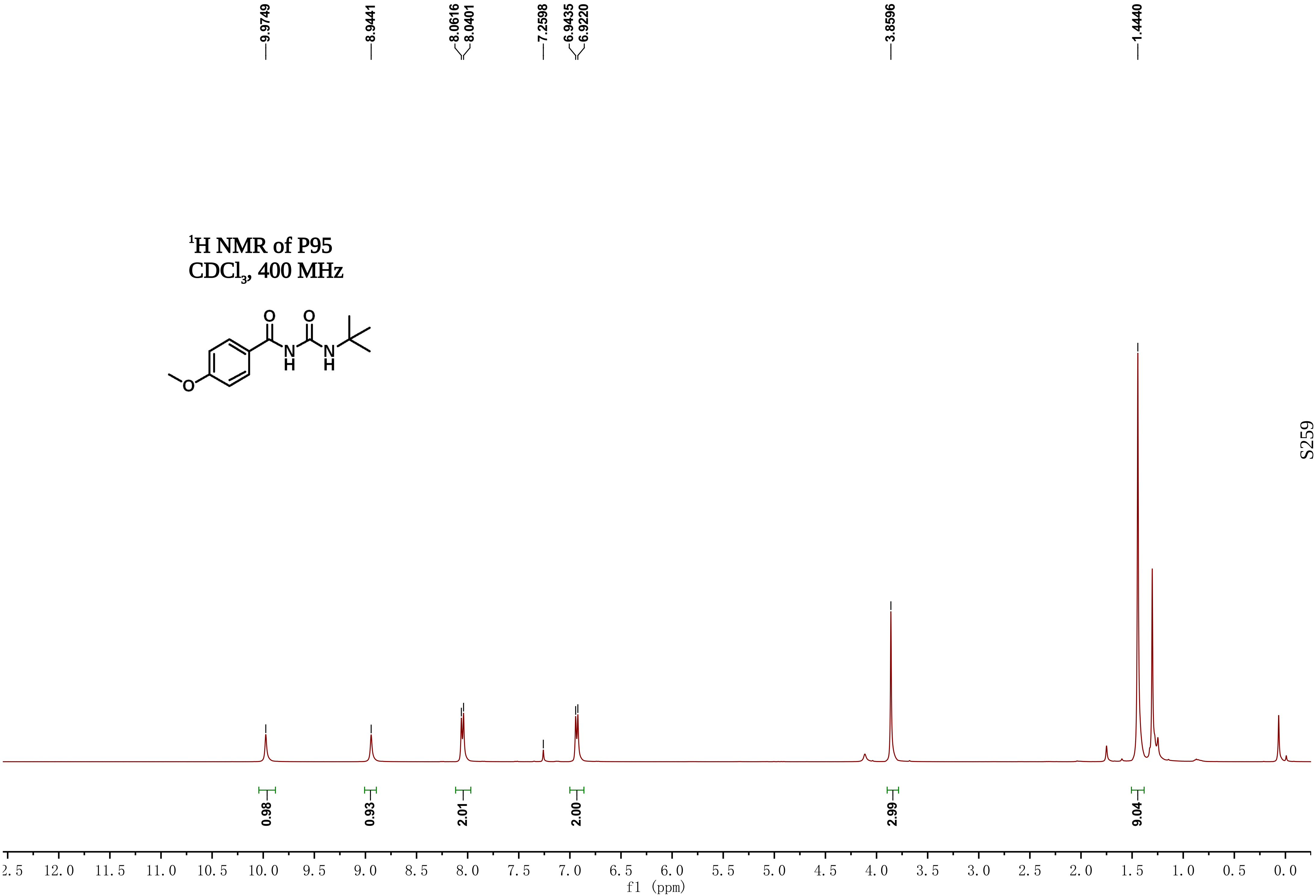

9.9749
8.9441
8.0616
8.0401
7.2598
6.9435
6.9220
3.8596
1.4440
^{1}H NMR of P95
$CDCl_3$, 400 MHz
O
O
N
H
N
H
O
0.98
0.93
2.01
2.00
2.99
9.04
12.5 12.0 11.5 11.0 10.5 10.0 9.5 9.0 8.5 8.0 7.5 7.0 6.5 6.0 5.5 5.0 4.5 4.0 3.5 3.0 2.5 2.0 1.5 1.0 0.5 0.0
f1 (ppm)

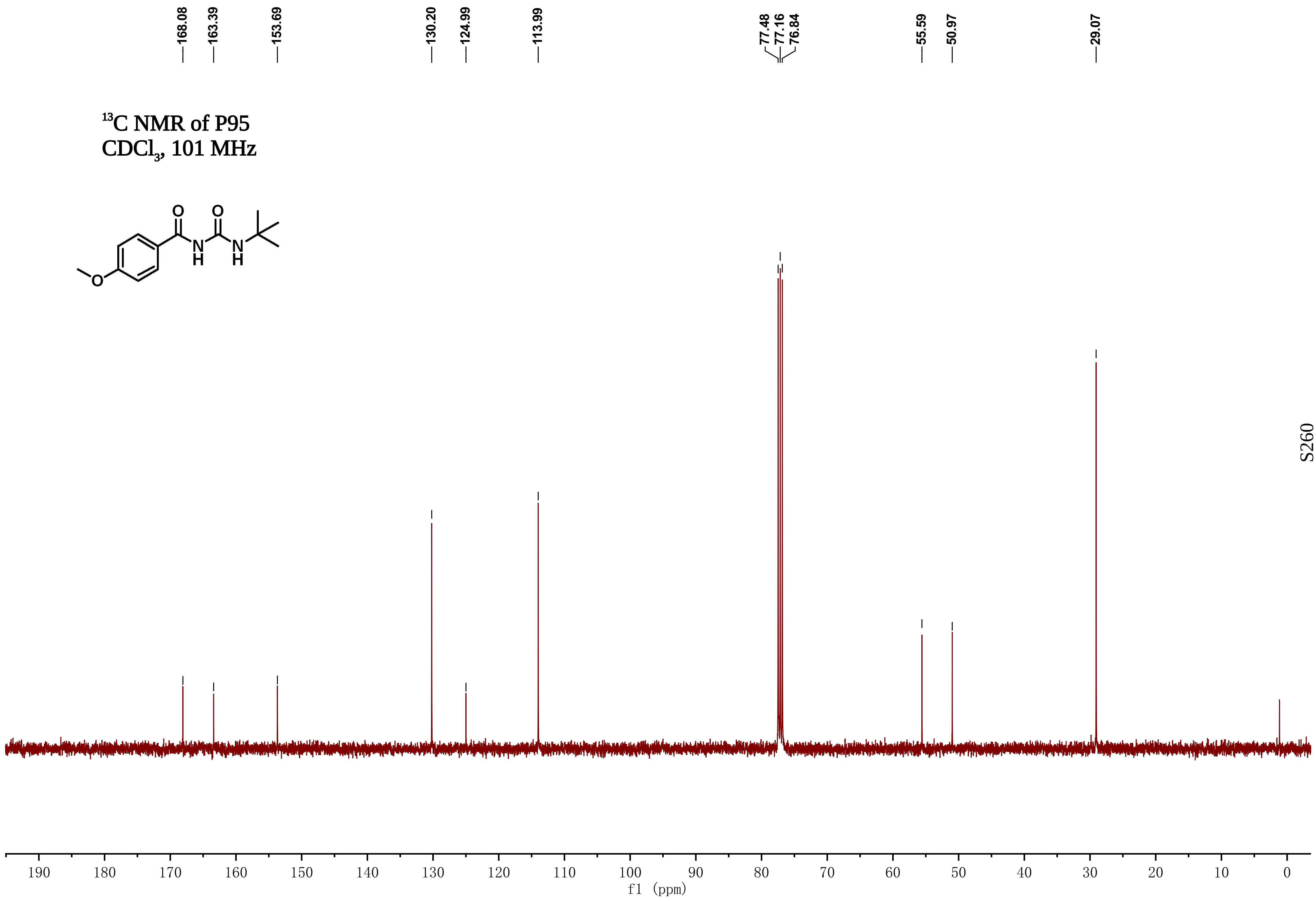

168.08
163.39
153.69
130.20
124.99
113.99
77.48
77.16
76.84
55.59
50.97
29.07
13C NMR of P95
CDCl3, 101 MHz
O
O
N
H
N
H
O
190
180
170
160
150
140
130
120
110
100
90
80
70
60
50
40
30
20
10
0
f1 (ppm)

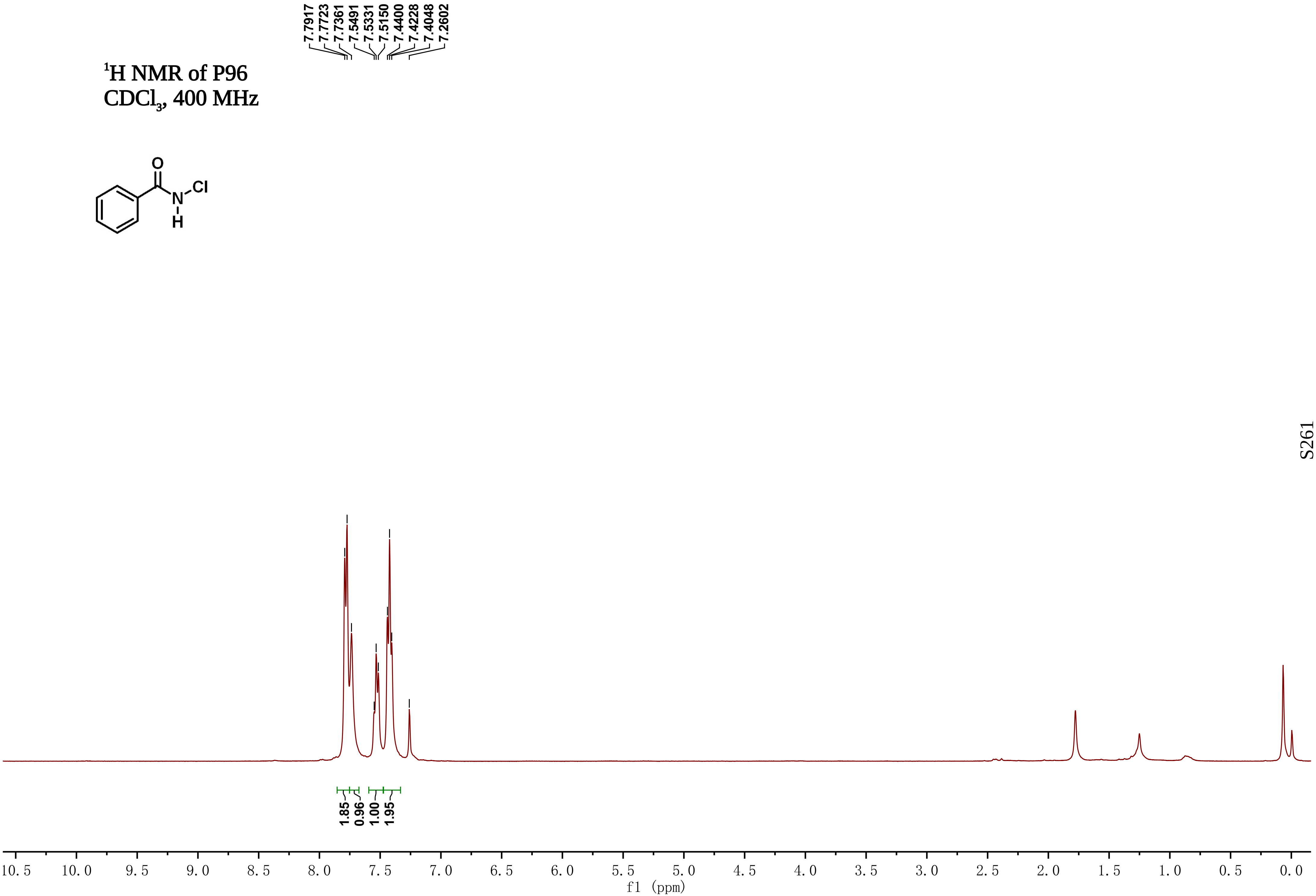

1H NMR of P96
CDCl3, 400 MHz
O
N
Cl
H
7.7917
7.7723
7.7361
7.5491
7.5331
7.5150
7.4400
7.4228
7.4048
7.2602
1.85
0.96
1.00
1.95
10.5
10.0
9.5
9.0
8.5
8.0
7.5
7.0
6.5
6.0
5.5
5.0
4.5
4.0
3.5
3.0
2.5
2.0
1.5
1.0
0.5
0.0
f1 (ppm)

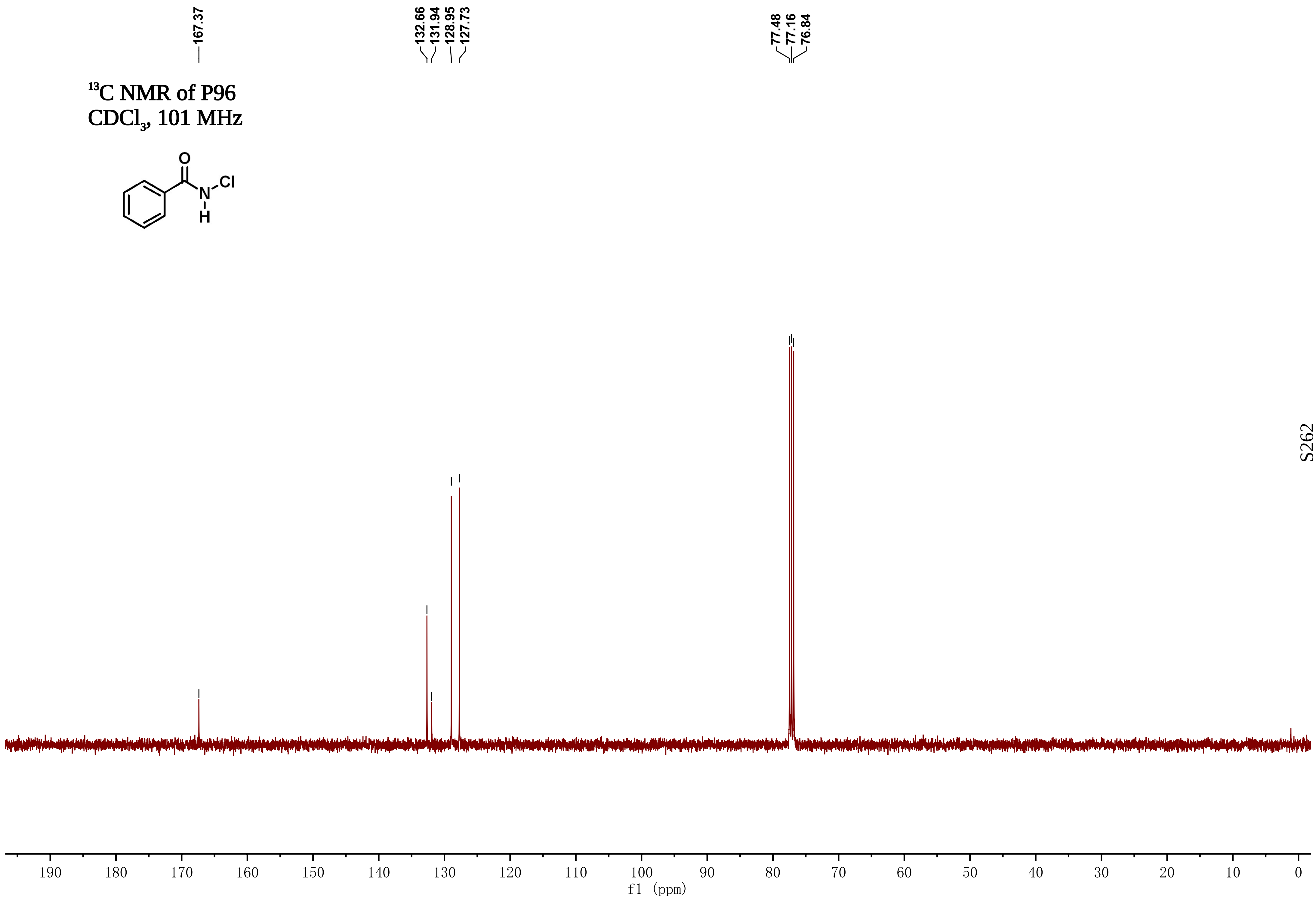
167.37
132.66
131.94
128.95
127.73
77.48
77.16
76.84
^{13}C NMR of P96
$CDCl_3$, 101 MHz
O
N
Cl
H
190 180 170 160 150 140 130 120 110 100 90 80 70 60 50 40 30 20 10 0
f1 (ppm)

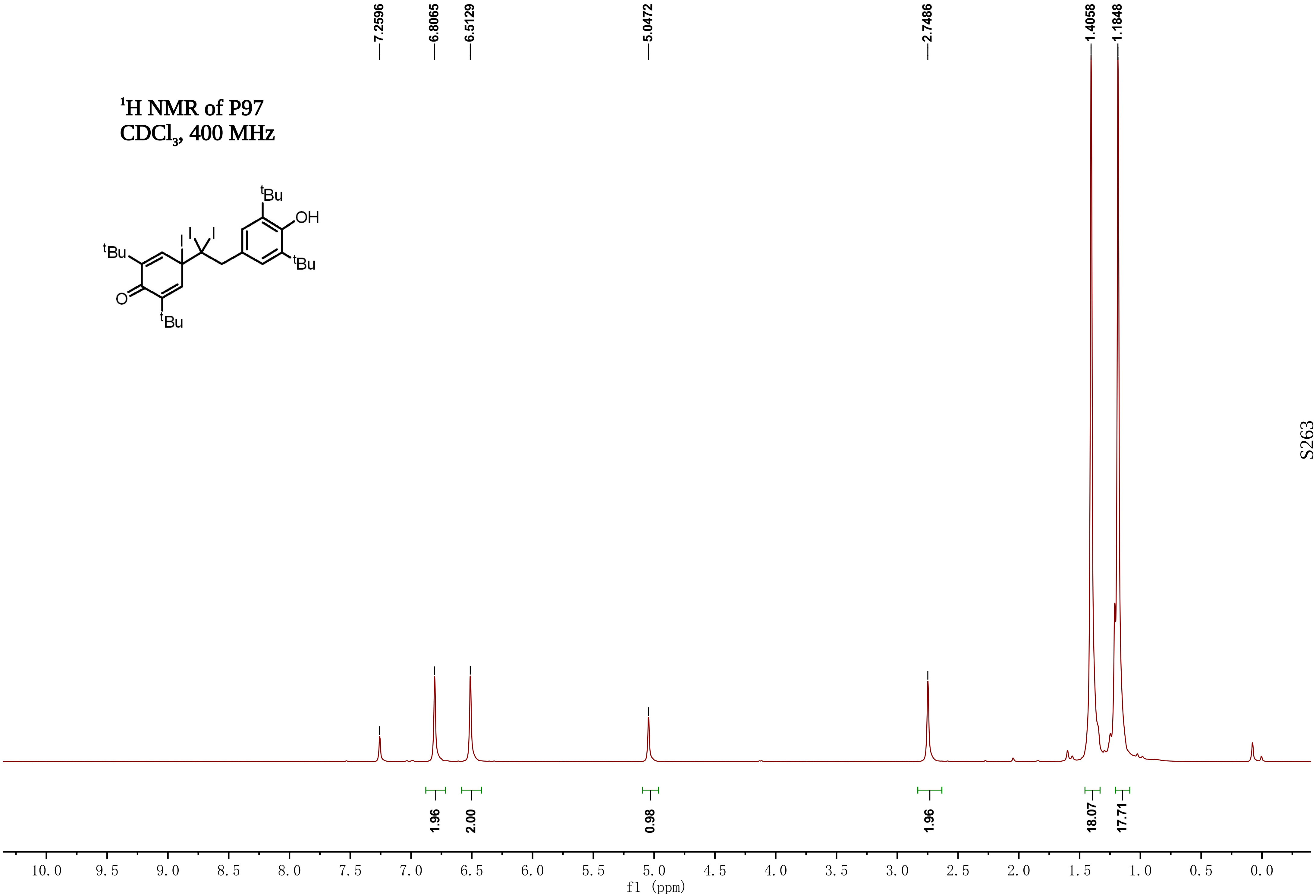

1H NMR of P97
CDCl3, 400 MHz
tBu
OH
tBu
tBu
tBu
O
I
I
I
7.2596
6.8065
6.5129
5.0472
2.7486
1.4058
1.1848
1.96
2.00
0.98
1.96
18.07
17.71
10.0
9.5
9.0
8.5
8.0
7.5
7.0
6.5
6.0
5.5
5.0
4.5
4.0
3.5
3.0
2.5
2.0
1.5
1.0
0.5
0.0
f1 (ppm)

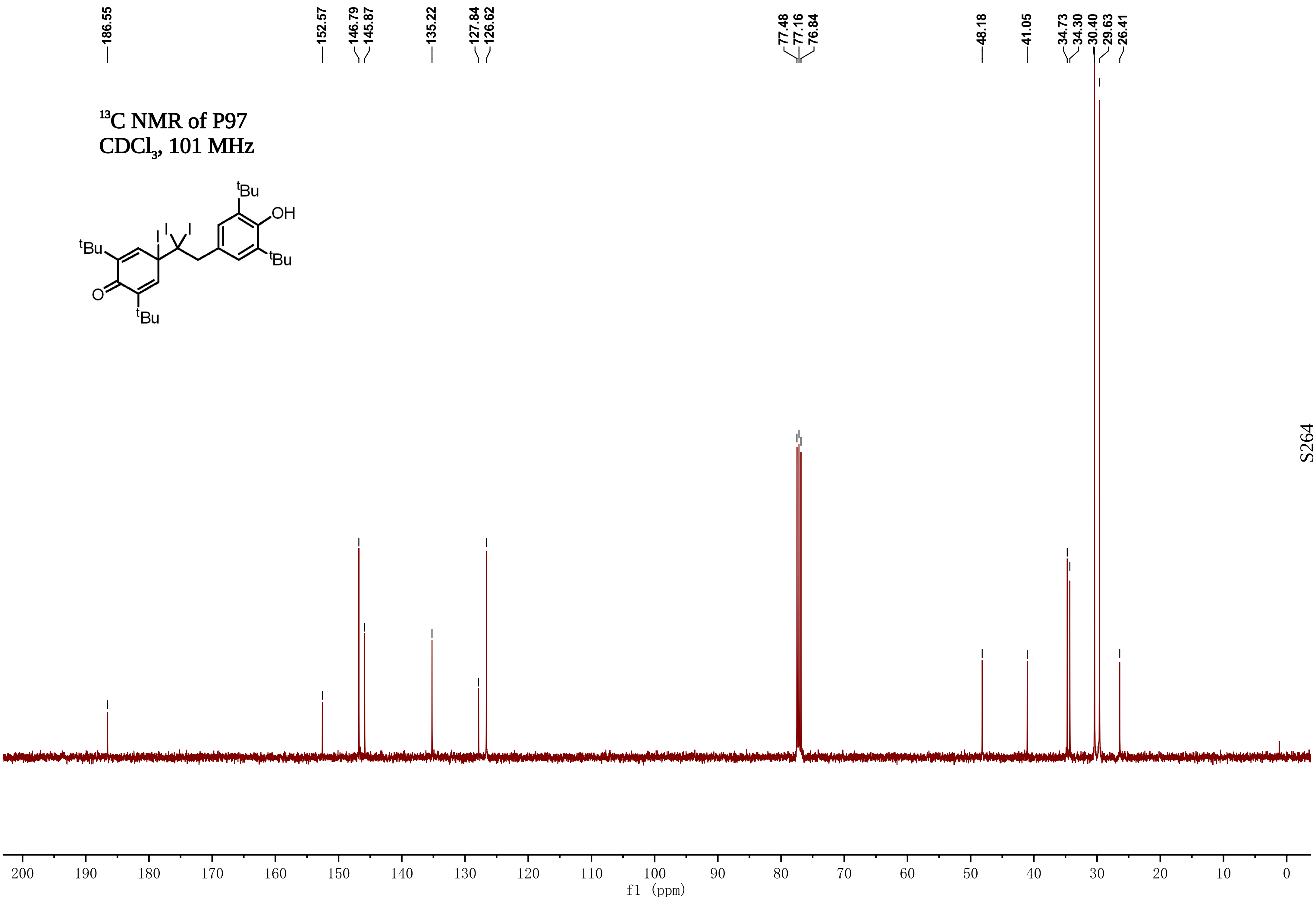
186.55
152.57
146.79
145.87
135.22
127.84
126.62
77.48
77.16
76.84
48.18
41.05
34.73
34.30
30.40
29.63
26.41
13C NMR of P97
CDCl3, 101 MHz
tBu
OH
I
I
I
tBu
tBu
O
tBu
200
190
180
170
160
150
140
130
120
110
100
90
80
70
60
50
40
30
20
10
0
f1 (ppm)

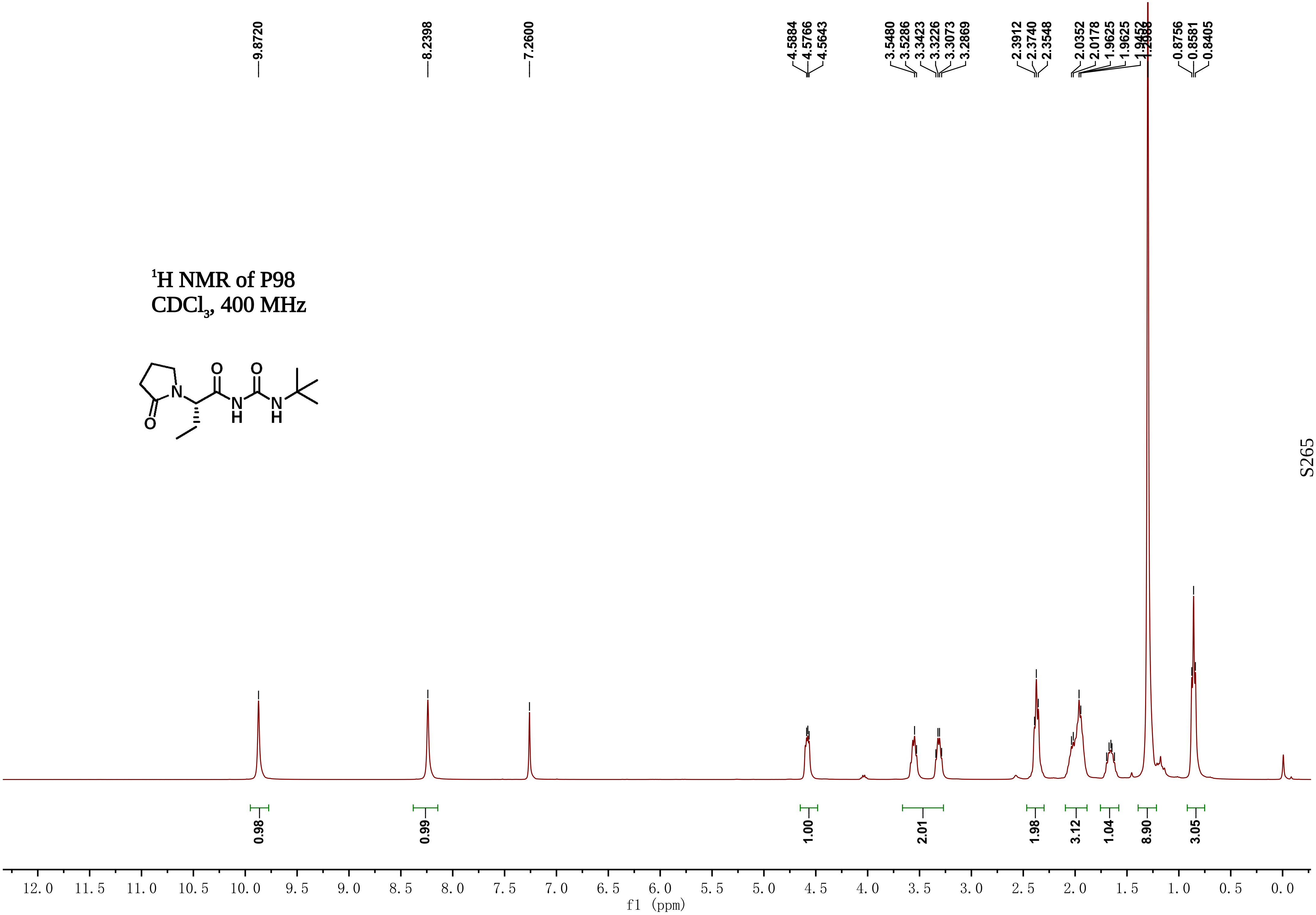

1H NMR of P98
CDCl3, 400 MHz
9.8720
8.2398
7.2600
4.5884
4.5766
4.5643
3.5480
3.5286
3.3423
3.3226
3.3073
3.2869
2.3912
2.3740
2.3548
2.0352
2.0178
1.9625
1.9625
1.9452
0.8756
0.8581
0.8405
0.98
0.99
1.00
2.01
1.98
3.12
1.04
8.90
3.05
12.0 11.5 11.0 10.5 10.0 9.5 9.0 8.5 8.0 7.5 7.0 6.5 6.0 5.5 5.0 4.5 4.0 3.5 3.0 2.5 2.0 1.5 1.0 0.5 0.0
f1 (ppm)

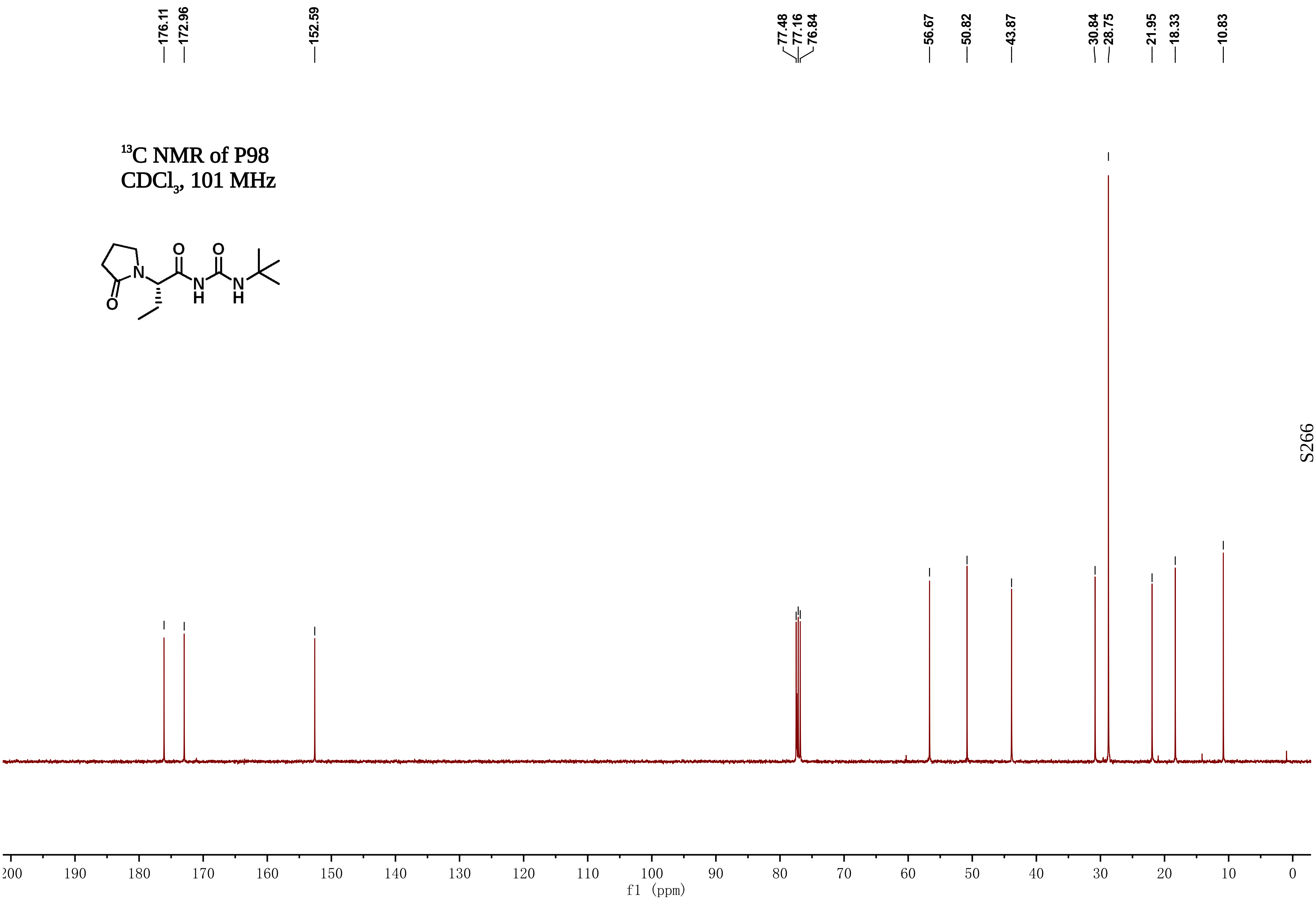

176.11
172.96
152.59
77.48
77.16
76.84
56.67
50.82
43.87
30.84
28.75
21.95
18.33
10.83
^{13}C NMR of P98
$CDCl_3$, 101 MHz
O
O
N
N
H
N
H
O
200
190
180
170
160
150
140
130
120
110
100
90
80
70
60
50
40
30
20
10
0
f1 (ppm)
